# The Human Blood Transcriptome in a Large Population Cohort and Its Relation to Aging and Health

**DOI:** 10.3389/fdata.2020.548873

**Published:** 2020-10-30

**Authors:** Maria Schmidt, Lydia Hopp, Arsen Arakelyan, Holger Kirsten, Christoph Engel, Kerstin Wirkner, Knut Krohn, Ralph Burkhardt, Joachim Thiery, Markus Loeffler, Henry Loeffler-Wirth, Hans Binder

**Affiliations:** ^1^IZBI, Interdisciplinary Centre for Bioinformatics, Universität Leipzig, Leipzig, Germany; ^2^BIG, Group of Bioinformatics, Institute of Molecular Biology, National Academy of Sciences, Yerevan, Armenia; ^3^IMISE, Institute for Medical Informatics, Statistics and Epidemiology, University of Leipzig, Leipzig, Germany; ^4^Leipzig Research Centre for Civilization Diseases, University of Leipzig, Leipzig, Germany; ^5^Institute of Laboratory Medicine, Clinical Chemistry and Molecular Diagnostics, University of Leipzig, Leipzig, Germany

**Keywords:** self-organizing maps, omics and phenotype integration, age, lifestyle and obesity, gene expression, immune response, subtypes

## Abstract

**Background:** The blood transcriptome is expected to provide a detailed picture of an organism's physiological state with potential outcomes for applications in medical diagnostics and molecular and epidemiological research. We here present the analysis of blood specimens of 3,388 adult individuals, together with phenotype characteristics such as disease history, medication status, lifestyle factors, and body mass index (BMI). The size and heterogeneity of this data challenges analytics in terms of dimension reduction, knowledge mining, feature extraction, and data integration.

**Methods:** Self-organizing maps (SOM)-machine learning was applied to study transcriptional states on a population-wide scale. This method permits a detailed description and visualization of the molecular heterogeneity of transcriptomes and of their association with different phenotypic features.

**Results:** The diversity of transcriptomes is described by personalized SOM-portraits, which specify the samples in terms of modules of co-expressed genes of different functional context. We identified two major blood transcriptome types where type 1 was found more in men, the elderly, and overweight people and it upregulated genes associated with inflammation and increased heme metabolism, while type 2 was predominantly found in women, younger, and normal weight participants and it was associated with activated immune responses, transcriptional, ribosomal, mitochondrial, and telomere-maintenance cell-functions. We find a striking overlap of signatures shared by multiple diseases, aging, and obesity driven by an underlying common pattern, which was associated with the immune response and the increase of inflammatory processes.

**Conclusions:** Machine learning applications for large and heterogeneous omics data provide a holistic view on the diversity of the human blood transcriptome. It provides a tool for comparative analyses of transcriptional signatures and of associated phenotypes in population studies and medical applications.

## Introduction

Blood is the pipeline of the human organism's physiology. The accessibility and minimal invasiveness during sampling has made it a feasible resource in scientific research and clinical diagnostics as they could replace more invasive and risky tests (Sohn, 2017). Because of utility and simplicity, blood transcriptome investigations on genome-wide scales have gained in popularity over the past few years. They were applied in a medical context for characterizing diseases such as ischemic stroke (Baird et al., 2015), Alzheimer's disease (Rembach et al., 2013), epilepsy (Karsten et al., 2011), sepsis (Davenport et al., 2016; Burnham et al., 2017; Scicluna et al., 2017; Hopp et al., 2018b); in pharmacogenomics (Burczynski and Dorner, 2006) and marker search (Hanash et al., 2011); and also in epidemiological investigations on aging (Peters et al., 2015), obesity status (Johannsen et al., 2010; Homuth et al., 2015), lifestyle factors such as smoking and alcohol consumption (Dumeaux et al., 2010), special nutrition (Burton et al., 2018), and in immune system characterization (Chaussabel et al., 2010) (see Chaussabel, 2015 and references cited therein for a broad literature survey). Most of these studies comprise of relatively small sample sizes of dozens to a few hundred individuals and they focus on selected diseases thus enabling only limited views on the variability of transcriptomic states and the mutual associations with health phenotypes in a broader context.

We here present the systematic analysis of the transcriptomes obtained from whole peripheral blood specimens of more than 3,000 adult individuals collected as part of the LIFE (-adult) study at the Leipzig Research Center for Civilization Diseases. This project conducted one of the largest cross-sectional population studies in Germany focusing on extensive phenotyping of urban individuals from Leipzig city in order to discover the interplay between molecular, environmental, and lifestyle factors and their impact on the health status of the population (Loeffler et al., 2015). The large number of phenotype characteristics collected in LIFE in parallel to blood samples from the same participants such as disease history, medication status, lifestyle factors, and body mass index (BMI) offers the option to study their mutual associations for women and men over an age range from about 40 to 80 years (Loeffler et al., 2015) (Table 1).

**Table 1:** Participant's characteristics of the LIFE-adult study used in this publication for association with the blood transcriptome (see also Supplementary Table 1 in Supplementary File 1 for further details).

**Features**		**Men**		**Women**		**Comment**
Number of participants^a^		1,618		1,510		
Age (mean ± SD)		58.1 ± 12.4		59 ± 13		Years
Smoker/Ex-smoker		1,000 ^e)^		701		
<30 g alcohol per day		633		218		
**Features**	**Symbol**	**# men**	**Mean age (±** **SD)**	**# women**	**Mean age (±** **SD)**	**Description (BMI in units of kg/m**^**2**^**)**
BMI status	uwt	14	39 ± 9	42	46 ± 10	Underweight BMI <18.5
	nwt	375	53 ± 15	492	54 ± 12	Normal weight 18.5 < BMI <25
	Pre obese	590	60 ± 12	443	60 ± 12	25 < BMI <30
	Obese	411	63 ± 11	311	61 ± 11	30 < BMI
**Features**						
Blood Count^b^	Basophils; eosinophils, erythrocytes; hematocrit; hemoglobin; leucocytes; lymphocytes; mean corpuscular hemoglobin; mean platelet volume; monocytes; neutrophils; reticulocytes; platelets					
Blood Serum markers	Human serum C-reactive protein; ferritin; transferrin; cystatin C					
Medication^c^	Alimentary tract and metabolism; blood and blood forming organs; cardiovascular system; dermatologicals; genitourinary system and sex hormones; systemic hormonal preparations, excl. sex hormones and insulins; anti-infective for systemic use; antineoplastic and immuno-modulating agents; muscular-skeletal system; nervous system; antiparasitic products, insecticides, and repellents; respiratory system; sensory organs; various					
Disease history^d^	Angina pectoris; arthrosis; asthma; cancer; cataract; depression; diabetes; glaucoma; gout; heart attack; hepatitis; hyperlipidemia; hypertension; hzoster; rheuma; sepsis; thyroid					

a For the detailed description of the LIFE-adult study see (Loeffler et al., 2015).

b *Analyses using clinical laboratory (Loeffler et al., 2015)*.

c Medications taken within the last 5 days before the LIFE-core program visit. Medication was classified according to Anatomical Therapeutic Chemicals (ATCs) indexing, https://www.whocc.no~/atc_ddd_index/).

d *Disease history of the participants was assessed in questionnaires (Loeffler et al., 2015)*.

**Figure 1:**
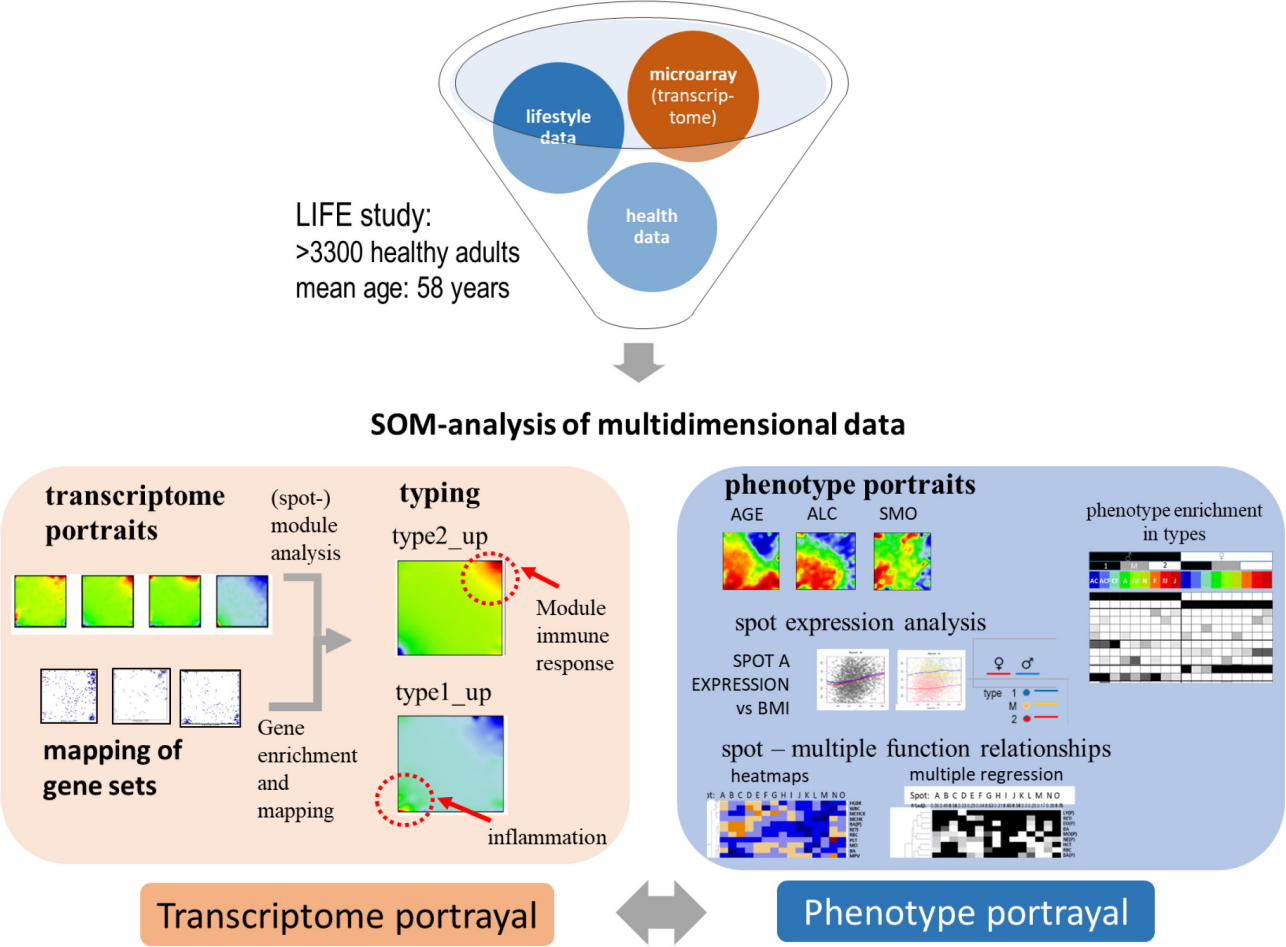
Schematic overview about portrayal approach applied in this study: subject-matched transcriptome and “phenotype” data of the LIFE-adult study were analyzed using SOM machine learning to obtain “personalized” transcriptome portraits. They were used for classification, gene-module extraction, and functional interpretation. Phenotype features such as blood cell counts or BMI provided phenotype (vs.-transcriptome correlation) portraits. For phenotypic features analyzed in this study see Table 1.

Our study aims at characterizing the diversity of transcriptional states of the blood transcriptome and their impact in terms of cellular functions and at studying associations with age and health-related features, so-called phenotypes, such as obesity, smoking, disease history, and medication status. From a methodical point of view, integrative analysis of molecular “omics” features and of phenotypes challenges the computational analysis framework (de Meulder et al., 2018). We have previously developed an omics “portrayal” methodology based on self-organizing maps (SOM) machine learning which takes into account the multidimensional nature of gene regulation and pursues a modular view on co-expression, reduces dimensionality, and supports visual perception by delivering “personalized,” case-specific transcriptome portraits (Wirth et al., 2011; Binder and Wirth, 2014). This method has been applied to a series of data types and diseases (Hopp et al., 2015b; Kunz et al., 2018; Bilz et al., 2019; Loeffler-Wirth et al., 2019; Nikoghosyan et al., 2019), among them a study on the blood transcriptomes of sepsis patients framed with pneumonia (Hopp et al., 2018b). In this publication we extend this approach to a much larger data set comprising the blood transcriptomes of thousands of nominally healthy individuals and of associated phenotype data. Figure 1 provides a schematic overview: SOM-portrayal permits a detailed description and visualization of the molecular heterogeneity of transcriptional states and of their association with different phenotypes. Our approach is expected to provide a detailed view of the blood transcriptome of a healthy population as a function of age, sex, and obesity status. It provides a methodical framework applicable to large data sets in the context of personalized medicine with potential impact for applications in medical diagnostics and molecular and epidemiological research.

## Materials and Methods

### LIFE-Adult Study and Phenotype Characteristics

The LIFE (-adult) study performed extensive phenotyping of more than 10,000 urban individuals from Leipzig city (Loeffler et al., 2015). The study was approved by the ethics board of the Medical Faculty of the University of Leipzig. In this publication we analyzed transcriptomic data of whole peripheral blood (WPB) samples, which were obtained from 3,388 adult participants of the study. They roughly divide equally into women and men covering an age range between about 20 and 80 years with a strong bias toward elderly persons (Table 1). The LIFE-adult study overall collected a broad survey of more than 20,000 lifestyle and health items (see Loeffler et al., 2015 for details). We made use of selected lifestyle characteristics of the participants such as smoking behavior and alcohol consumption, medication according to ATCs (Anatomical Therapeutic Chemicals) indexing and disease history of the participants collected via questionnaires, blood count data from clinical laboratory including selected serum markers, and body mass index (BMI) (Table 1 and Supplementary Table 1 for details). A list of items and abbreviations used is provided as Glossary in Supplementary File 1.

### Blood Transcriptome Sampling, Microarray Measurements, and Data Preprocessing

We made use of pre-processed gene expression data extracted from WPB samples of individuals as provided by the LIFE database. Participant's recruitment, blood collection, storage and mRNA preparation, microarray measurements, and primary data pre-processing was realized by different groups of the LIFE center (Loeffler et al., 2015). WPB was collected in tempus blood RNA tubes (ThermoFisher, Waltham, MA, USA) and stored at −80°C until further processing. RNA was isolated and then hybridized to Illumina HT-12 v4 Expression BeadChips (Illumina, San Diego, CA, USA) and measured on an Illumina HiScan device. Raw probe level data were extracted using Illumina GenomeStudio and then further pre-processing including batch correction, outlier and missing value removal, log-transformation, quantile normalization, and centralization of the expression value of each gene using an in-house pipeline as described in detail in Supplementary Methods (Supplementary File 1) was undertaken. The final transcriptome data consists of more than 48,000 probe IDs including the expression values of 19,049 genes for each of the individuals.

### Self-Organizing Maps (SOM) Transcriptome Portrayal

Pre-processed expression values were analyzed using the oposSOM pipeline, available as the R-package “oposSOM” (Löffler-Wirth et al., 2015). It uses SOM neuronal network machine learning to translate the high-dimensional expression data of *N* = 19,049 gene transcripts into K = 10,000 metagene expression data per individual (Wirth et al., 2011, 2012). Each metagene represents a “micro”-cluster of co-expressed genes showing mutually similar expression profiles across the samples. Metagenes were arranged in a 100 × 100 two-dimensional grid coordinate system and colored according to their expression level for each sample thus providing a “personalized” image of the blood transcriptome of each individual studied (Supplementary Figure 1A, for Supplemenatry Figures see Supplementary File 1). Size of the SOM was chosen to be virtually insensitive for a downstream analysis task regarding, e.g., the number of spots based on previous systematic adjustments of the method (Binder and Wirth, 2014). Mean portraits of transcriptome classes (see below) were calculated by averaging metagene expression values over all portraits of the respective group. Default color scale (red to blue for maximum to minimum expression, respectively) of the portraits uses log-expression values of the metagenes (Wirth et al., 2011). The diversity of the sample portraits was visualized using a graph representation called a “correlation network” as implemented in “oposSOM” (Löffler-Wirth et al., 2015). Downstream analysis of the SOM-portraits then provides quantitative features such as modules and lists of co-regulated genes and information about gene functions using enrichment techniques (see next subsections) together with statistical evaluation as described previously (Wirth et al., 2011, 2012) and implemented in the oposSOM software (Löffler-Wirth et al., 2015).

### “Spot” Clustering of Co-expressed Genes and Stratification of Samples

Metagenes of similar profiles clustered together forming “spot-like” red and blue areas of over- and under-expression in the portraits due to the self-organizing properties of the SOM. Each of the spots represents a cluster of mutually correlated genes (Supplementary Figure 1B). The spots were detected using a distance-metrics criterion making use of *Euclidean* distance between neighboring metagenes, where metagenes of maximum mutual distances form closed, halo-like lines around the “spots” (Vesanto, 1999) (see D-map in Supplementary Figure 1B for illustration). The spot expression patterns obtained represents a characteristic fingerprint of each particular sample. Lists of genes included in each of the spot modules and lists of enriched gene sets were provided as Supplementary Excel tables together with statistical information (Supplementary Tables 2–4 in Supplementary File 1 and Supplementary Files 3, 4, respectively). The overall collection of spot-modules detected are major nodes of the co-expression network derived from the sample series (see the spot correlation and implication networks in Supplementary Figures 1B,C, respectively). Spot selection criteria were developed and described previously (Wirth et al., 2011, 2012; Binder and Wirth, 2014) and applied and proven in numerous publications to provide reasonable results (Binder et al., 2014, 2017; Cakir et al., 2014, 2017; Hopp et al., 2015b,c; Hopp et al., 2018a,b; Gerber et al., 2017; Kunz et al., 2018; Arakelyan et al., 2019; Loeffler-Wirth et al., 2019; Nikoghosyan et al., 2019). Based on the spots detected in the transcriptome portraits we stratified the samples into appropriate groups. First, the portraits were divided into 33 so-called combinatorial pattern-types (cPATs), each defined by a certain unique combination of over-expressed spots as described recently (Loeffler-Wirth et al., 2019) (Supplementary Figure 2A). Using the cPATs we estimated the tentative number of groups (Supplementary Figures 2A–C) and used them subsequently in a K-means clustering run, which stratifies the portraits into three major transcriptome types and nine subtypes (STs, Supplementary Figures 2A–C). The transcriptome strata were further characterized by detailed statistics about spot appearance (Supplementary Figures 2C,D) and verified by random splits of the cohort into training and verification subsets, resampling, and subsequent classification using support vector machine (Supplementary Figure 3).

### Function Mining

We applied a gene set analysis to the lists of genes located in each of the spot modules to discover their functional context using a right-tailed Fisher's exact test as described previously (Wirth et al., 2012). In addition, the gene set enrichment z-score (GSZ) was used to evaluate the impact of the gene sets in the different transcriptomic strata (Wirth et al., 2012). The GSZ-metrics considers the mean expression of the gene set normalized by its variance, i.e., it provides high values for homogeneous gene sets reflecting the activation of biological functions with high relevance for the respective transcriptional states. Gene set maps complement this analysis by visualizing the position of the gene of a set within the SOM grid. According to their degree of accumulation in or near the spots, one can deduce their potential functional context (Wirth et al., 2011).

### Phenotype Portrayal

Phenotype information of the participants comprises their blood cell and marker counts, BMI and information about their lifestyle (smoking and alcohol consumption), and medication and disease history (Table 1 and Supplementary Table 1). The enrichment of categorical phenotypic characteristics in each of the transcriptomic classes (types and subtypes) were estimated using a one-tailed Fisher's exact test and visualized as enrichment heatmaps. Phenotype-to-metagene correlation maps were generated by correlating each of the phenotype parameter-profiles over all participants with each of the metagene expression profiles. For categorical phenotypes, correlation maps were obtained by calculating the point biserial correlation between the expression profile of each metagene and the respective phenotype profile. Point serial correlation de facto provides the difference of portraits between blood transcriptomes showing the respective phenotype and all others. The matrix of correlation coefficients obtained was visualized in the SOM-grid as “phenotype” portraits using a red-to-blue (maximum-to-minimum correlation) color-code. The metagene of maximum correlation coefficient was marked in the SOM-grid of a phenotype overview map. Expression of each of the spots was fitted using multiple regression with the phenotype values of the participants of each of the categories as variables. Standardized regression coefficients and their *p*-values were then visualized as heatmaps (Supplementary File).

### Availability of Data and Software

Processed transcriptomic data of this study are available as “SOM-data” via the Leipzig Health Atlas under the link https://www.health-atlas.de/data_files/76?version=1 and https://www.health-atlas.de/som_browser/201611_LIFE_Transcriptome/Summary.html (pdf and html reports). Data can be interactively discovered using the oposSOM browser functionality available under https://www.izbi.uni-leipzig.de/opossom-browser/ and https://apps.health-atlas.de/opossom-browser/?dataset=6. Raw expression data and participants information can be requested from the LIFE Consortium (www.life.uni-leipzig.de/en/). The oposSOM program (Löffler-Wirth et al., 2015) is available under https://rdrr.io/github/hloefflerwirth/oposSOM/.

## Results

### The Blood Transcriptome Splits Into Three Types

**Figure 2:**
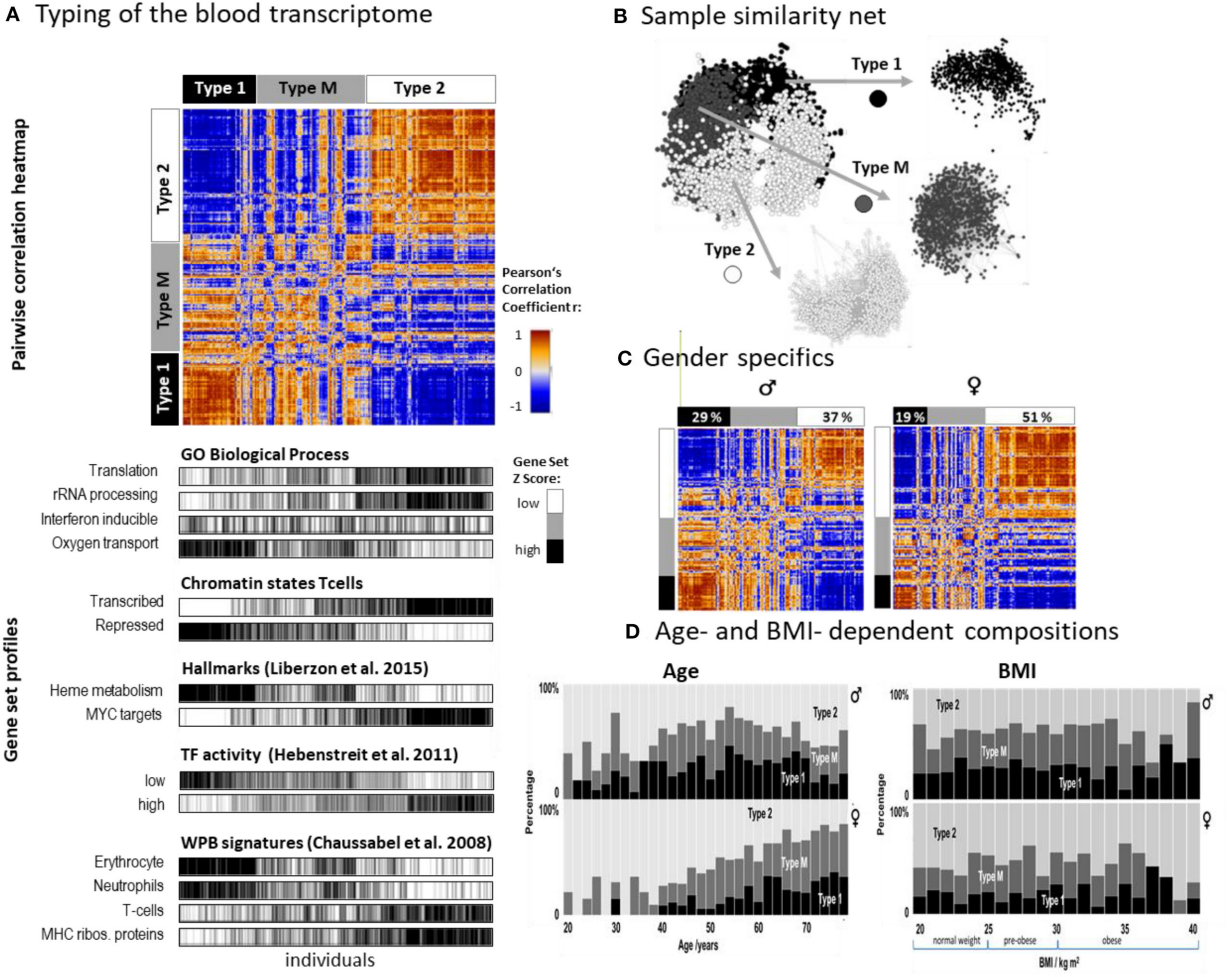
Stratification of the blood transcriptome into three types: **(A)** The pairwise correlation heatmap divides the samples into three transcriptome types according to the mutual correlations between their SOM expression patterns. The part below associates the samples of all three types to selected functional categories by means of gene set overexpression. The samples are sorted into clusters according to different combinatorial pattern types (cPATs) which gives rise to the striped patterns in the map (see below). **(B)** Network presentation of the similarity relations illustrates that the samples of type 1 and type 2 form almost separate data clouds while type M overlaps with both. **(C)** Stratification of the samples into women and men shows essentially similar correlation heatmaps and thus similar expression patterns. Women however are more frequently found in type 2. **(D)** The composition of types changes with age in a gender specific way but does not with BMI (body mass index). The percentage of type 2 women permanently decreases with age while the relative amount of type 2 men older than 55 years again increases.

SOM analysis provided one portrait for each of the 3,388 LIFE-adult participant's WPB transcriptomes (Supplementary File 2 and Supplementary Figure 1A). For the stratification of samples we made use of the so-called combinatorial spot patterns approach (cPATs, see also next subsection), which largely reduces the dimension of the data, and subsequent clustering as described in detail previously (Loeffler-Wirth et al., 2019), in the methods part and in Supplementary Figure 2. The associated cluster tree is shown in Supplementary Figure 2A and Figure 2A. Overall, we identified three major strata of transcriptomes called type 1, type 2, and type M (q = 0.003, Anova; classification error: 10% of samples after resampling and SVM-based re-classification, see Supplementary Figure 3B). The pairwise correlation map illustrates the similarities between the types in terms of Pearson's correlation coefficients between the expression portraits (Figure 2A). Type 1 and type 2 show pronounced anti-correlated expression portraits while type M forms an intermediate group. The network presentation reveals that WPB transcriptomes of type 1 and type 2 split into separate clusters while type M samples overlap between them (Figure 2B). The functional context of activated genes were estimated using gene set analysis (Figure 2A, part below). Type 1 was associated with functional categories related to oxygen transport, heme metabolism, neutrophil accumulation, and repressed chromatin states of T cells while the type 2 group was related to immune response, transcriptional activity, T cell accumulation, and active chromatin states (see below). A higher percentage of men were found in type 1 (29% vs. 19% for women) while this reverses for type 2 (percentage of women: 37% vs. 51%; Figure 2C). Type 1 was more populated with elderly persons compared with type 2, while the distribution with age was different between women and men (Figure 2D). The composition of types for women changed virtually monotonously with a steadily increasing percentage of type 1 in contrast to men, who showed a maximum of type composition in the age range of 50–55 years. Note also that the age dependence of type M more resembled that of type 1 than that of type 2 which suggests a functional correspondence between types M and 1 (see below). The type-composition of men and women was virtually independent of BMI (body mass index) except for very obese persons (BMI > 35 kg/m^2^) which seemed to be more present in type 1 transcriptomes (Figure 2D).

Taken together, we identified two major blood transcriptome types and an intermediate type partly resembling type 1. Type 1 included more men, elderly participants, and upregulated genes associated with inflammation and increased heme metabolism, while type 2 included more women and younger participants. It was associated with activated immune responses and transcriptional activity. The composition of types changes in a gender- and age-specific fashion.

### A Modular Map of Gene Activation

**Figure 3:**
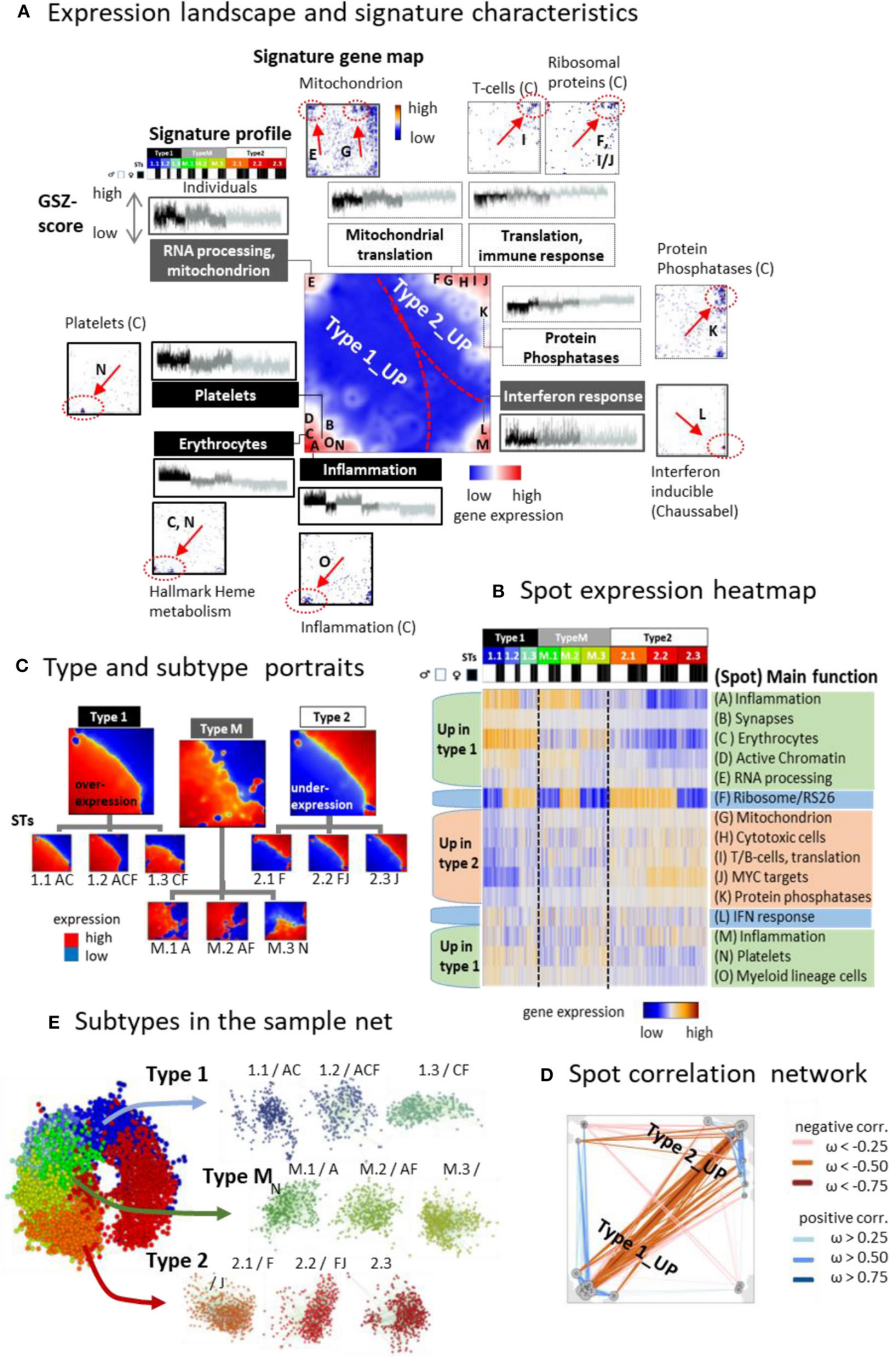
The landscape of the blood transcriptome: **(A)** The spot overexpression summary map shown in the center of **(A)** provides an overview about spot-clusters of co-expressed genes labeled with capital letters A–M. The map divides into areas, which contain spots upregulated predominantly in type 1 and type 2 transcriptomes, respectively. A selection of gene sets illustrates the functional context of the spots by showing their profiles and gene maps. These maps indicated the location of the genes of the respective gene set by dots. Their accumulation in and near spot areas are shown by arrows and dashed ellipses (see also Table S2). The expression profiles of the gene sets reveal their distinct up and downregulation in the different sample types. **(B)** Profiles of the spots are shown as heatmaps together with their major functional context (see also Table S 2). **(C)** The correlation map indicates positive and negative correlations between the spots by red and blue lines, respectively. Correlations were calculated using the weighted overlap measure (Hopp et al., 2013). **(D)** Mean portraits of types and subtypes (STs) reveal the mutual up and downregulation of genes located in different parts of the map. **(E)** Samples of different STs accumulate in well-separated data clouds in the similarity net.

Clusters of genes with correlated expression profiles appear as red spot-like areas in the transcriptomic portraits, which indicate their overexpression in the respective samples (Supplementary Figure 1A). Overall we identified 13 such major overexpression spots and labeled them with capital letters A–M (Figure 3A, for spot lists of genes see Supplementary Table 3 and Supplementary File 3 and for enriched gene sets Supplementary File 4). It roughly divides into two major areas containing spots predominantly upregulated either in type 1 (and partly also type M) or type 2 samples, respectively, and a third area with mixed spot assignment as illustrated by mean portraits of the transcriptomic types (Figure 3B), the spot profiles (Figure 3C and Supplementary Figure 4), and their correlation network (Figure 3D). Gene maps indicate the positions of genes taken from selected functional gene sets within the SOM grid of metagenes (Figure 3A). For example, genes upregulated in erythrocytes and platelets accumulate in spots C and N (up in type 1), respectively, while genes associated with mitochondrial function and RNA processing are found in spot E and G. Signature genes of T cells and of ribosomal function accumulate in and near spots I and J (up in type 2). Spot H accumulates the signature of CD4 cytotoxic T lymphocytes (CTLs) including the marker genes GZMA and PRF1, which were recently found to be associated with extreme longevity (Hashimoto et al., 2019). Genes with functions in interferon (IFN) response accumulate in spot L without preferential upregulation in one of the three types. Differential gene expression analysis between the types revealed a considerably larger number of genes upregulated in type 1 compared with type 2 (Supplementary Figure 5).

Typically, each of the individual sample portraits show more than one spot, which reflects the parallel activation of different transcriptional programs and/or their mutual couplings. We subsume frequently observed combinations of expressed spots as so-called combinatorial pattern types (cPATs) using a method described previously (Loeffler-Wirth et al., 2019). Overall we identified 33 cPATs, which were then used to sub-stratify each of the major transcriptomic types into three subtypes (STs, annotated by 1.1, 1.2, 1.3, M.1, M.2, M.3, and 2.1, 2.2, 2.3, respectively) differing in their mean expression portraits (Figure 3D) and spot expression (Figure 3B and Supplementary Figure 2). Part of the spot profiles show marked expression differences between the STs (e.g., spots A, B, D, F) while others change continuously (e.g., spots H- J). Most of the spots upregulate either in type 1 or 2 samples. Interestingly, spot F enriching genes encoding ribosomal subunit S26 proteins showed specific expression patterns with strong upregulation in part of STs without preference to either type 1 or type 2. Spot co-occurrence analysis indicates that adjacent spots are often observed together, but also spots from different areas can co-occur, especially in samples of type M, which supports their intermediate position between type 1 and type 2. Part of the STs are dominated by samples expressing only one spot while others, especially of type M, show a broader distribution owing to more heterogeneous expression patterns (Supplementary Figure 2C). The sample similarity net indicates that most samples of the different STs accumulate into well-localized clouds reflecting their mutual similarity (Figure 3E and Supplementary Figure 2F). The ST-composition is virtually age-independent except ST 1.1, which collects an increasing percentage of men and women at an age above 65 years (Supplementary Figure 6). In summary, the diversity of transcriptional states can be described by the combinatorics of about one dozen modules of co-expressed genes of different functional context, which decompose each of the transcriptional types into three subtypes.

### Footprints of Functions: Cellular Programs, Infections, Telomeres, and Epigenetics

**Figure 4:**
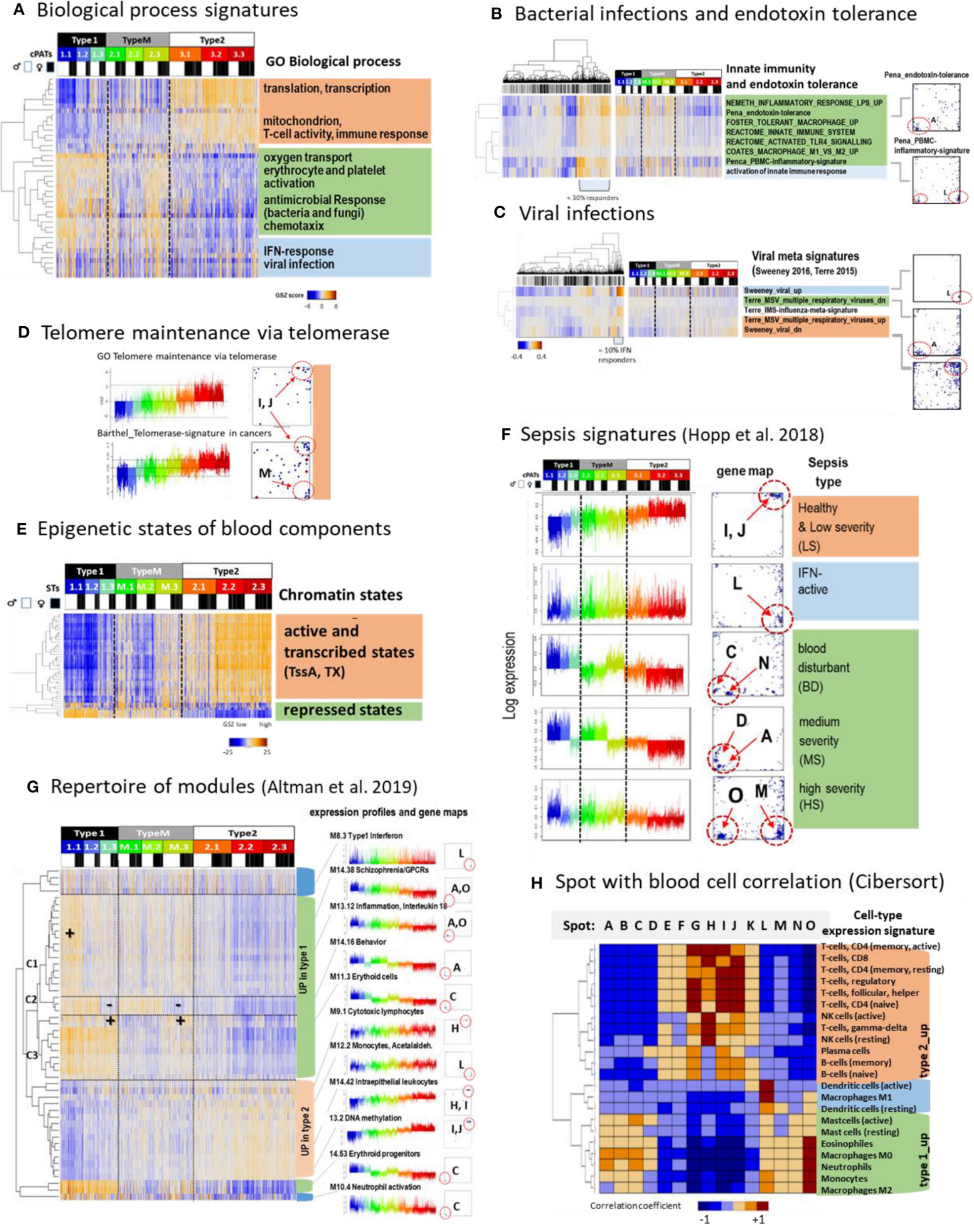
Functional characteristics and previous signatures of the blood transcriptome: **(A)** Signatures of the GO-term biological process (BP) roughly group into processes upregulated in type 1 (green), type 2 (apricot color), and in samples showing high expression in all types (blue) (see also Supplementary Figure 7). **(B)** Gene signatures associated with bacterial (Néemeth et al., 2003; Foster et al., 2007; Coates et al., 2008; Pena et al., 2014) and **(C)**, with viral infections (Andres-Terre et al., 2015; Sweeney et al., 2016) associate with distinct spots in the SOM and profiles. Clustering of samples reveals that about 30%/13% of specimens had elevated levels of transcriptomic footprints of bacterial/viral infections. **(D)** Signatures of telomere length maintenance (TM) taken from GO-repository (Subramanian et al., 2005) and a meta-analysis of cancer induced TM (Barthel et al., 2017). Both signatures activate in type 2 (spots I, J) where the cancer-derived signature also contained genes located in spot M associated with inflammation (see also Supplementary Figure 9). **(E)** Expression of genes assigned to different epigenetic states of healthy blood cells (see Roadmap Epigenomics Consortium et al., 2015) reflects that transcriptionally active states (active promoters and transcribed genes) upregulate predominantly in type 2 while repressed states become upregulated in type 1 that suggests their reprogramming into active ones (see also Supplementary Figure 10). **(F)** Expression signatures extracted from the blood transcriptomes of sepsis patients framed within community acquired pneumonia (CAP) (Hopp et al., 2018b) also express clear footprints in the LIFE-adult cohort. **(G)** A repertoire of functionally annotated transcriptional modules (Altman et al., 2019) reveals agreement with our typing scheme. The heatmap of the 50 most variant modules indicates concerted regulation with our types where modules overexpressed in type 1 further split into 3 clusters C1–C3 associating with specific ST expression patterns. Interestingly, modules which upregulate in type 2 show a less diverse cluster structure (see also Supplementary Figure 14). **(H)** Correlation between spot expression profiles and signature expression of a collection of 22 blood cell compounds taken from Cibersort (Newman et al., 2015). They upregulate either in type 1 or type 2 blood transcriptomes or resemble the IFN-response characteristics (dendritic cells and macrophages M1). These patterns are commonly found also for collections of other signatures thus reflecting major features of the intrinsic modular structure of the blood transcriptome (see Supplementary Figure 15). GSZ-patterns differences (red vs. blue) refer to *q* < 10^−3^ (Anova, **B–E**) and spot differences (**F**, *p* < 10^−9^, *t*-test).

Next, we performed functional analysis of the transcriptome strata using gene sets taken from the functional categories “biological process” (Subramanian et al., 2005) (Figure 4A), “hallmarks of cancer” offering disease characteristics in a more general context (Liberzon et al., 2015) (Supplementary Figure 8), “telomere maintenance” (Barthel et al., 2017), and “epigenetic states” (Figures 4A–E). Telomere expression signatures were chosen because mean telomere length in blood cells is associated with lifestyle and disease characteristics. In human leukocytes it negatively correlates with lifespan and BMI (Rode et al., 2015; Gielen et al., 2018) and it associates with heart diseases, type 2 diabetes, cancer (Oeseburg et al., 2010; Haycock et al., 2014; Polonis et al., 2019), lifestyle factors (Townsend et al., 2016), diet (Leung et al., 2018), and psychological stress (Epel and Prather, 2018). Hence, we are interested whether genes with telomere functions activate differently in the transcriptomic types or not. Moreover, such expression changes might reflect changed chromatin organization leading to altered cell function in type 1 compared with type 2 as discussed, e.g., as epigenetic mechanisms accompanying aging (Ciccarone et al., 2018) and inflammation (Busslinger and Tarakhovsky, 2014; Daniel et al., 2018; Ray and Yung, 2018; Lorente-Sorolla et al., 2019) and are associated with changes of DNA-methylation and histone-marks governing gene activity.

Profiling function-signatures splits them into two major clusters either upregulated in type 1 (marked with green color in the figures) or type 2 (apricot color), respectively. Gene signatures taken from the gene ontology category “biological process” reveal that type 2 associates with the activation of cell cycling, MYC-target genes, oxidative phosphorylation (oxphos), while inflammation, hypoxia, coagulation, reactive-oxygen species, and the pathway signaling of TNFalpha-, TGFbeta-, PI3K-Akt-MTOR-, and IL6-JAK-Stat3 activate in type 1. A third cluster (blue color) accumulates signatures related to interferon (IFN) response, which eventually suggests an association with viral infections (Figure 4A). We analyzed expression signatures derived recently to differentiate between bacterial and viral infections (Néemeth et al., 2003; Foster et al., 2007; Coates et al., 2008; Pena et al., 2014; Andres-Terre et al., 2015; Sweeney et al., 2016) (Figures 4B,C, respectively). The former signatures associated with the “inflammatory” spots A, O, and M, which were upregulated in type 1 samples. In contrast, viral signature genes accumulated strongly in the IFN-response spot L, which was found upregulated in about 10% of all samples. Next, we studied genes which are involved in telomere length maintenance (TM) via activation of telomerase. TM-genes were more active in type 2 transcriptomes, which suggests that they strongly counteracted telomere shortening in younger (and healthier) individuals (Figure 4D). TM expression was associated with cell cycle activity, starvation, oxidative stress, aging, DNA-methylation, and other functions related to spots I and J indicating mutual coupling between TM and our transcriptome types (see also Supplementary Figure 9).

Next we analyzed the expression sets of genes assigned to distinct chromatin states in blood cells under healthy conditions, among them T-, B-,and T-regulatory-cells (Figure 4E and Supplementary Figure 10). States involving genes with an active promoter (TssA) and a completed transcription (Tx) were expected to show high expression, while repressed promoter states were expected to show low expression levels. This relation was indeed observed in type 2 transcriptomes, however it reversed in type 1. This reversal suggests de-repression of nominally repressed states and repression of active states in type 1 transcriptomes by epigenetic chromatin re-modeling. We recently demonstrated that differentiation and adjustment of cellular programs are governed by subtle cooperation of transcription factor (TF-) networks and epigenetics, e.g., via regulation of the polycomb repressive complex 2 (PRC2) and its targets (Thalheim et al., 2018). We found that signatures related to TF-networks regulate cell function requiring relatively high expression levels of their major regulatory genes such as cell cycle, oxphos, and transcription predominantly in type 2 transcriptomes (Supplementary Figure 11). On the contrary, repressive epigenetic signatures related to PRC2 function, repressive histone (H3K27me3) marks, and DNA-methylation antagonistically changed compared with those of the TF-networks. Interestingly, these profiles show moderate and low expression levels according to the accumulation of their signature genes in the central region of the map. On the other hand, we found an asymmetry of differentially regulated genes and functions, namely a markedly larger number of genes (Supplementary Figure 5) and spot-modules (see below) which upregulated in type 1. It suggests a more distributed and heterogeneous network of transcriptional regulation under epigenetic control in type 1. In summary, type 2 transcriptomes were associated with cell cycle, oxphos-metabolism, telomere maintenance, and immune system activity regulated mainly via transcription factor networks, which become repressed in type 1 transcriptomes in parallel with epigenetic de-repression of inflammatory cellular programs including responses to infections.

### Previous Gene Expression Signatures of the Blood Transcriptome

Next, we analyzed a series of expression signatures taken from previous, independent studies of blood transcriptomes (Chaussabel et al., 2008; Peters et al., 2015; Hopp et al., 2018b; Altman et al., 2019) in our data to assign previous functional annotations, to draw parallels between blood transcriptomes of healthy and diseased individuals, and to also verify our data and classification scheme in the light of independent data. Modules of co-regulated genes taken from Chaussabel et al. (2008) well-agreed with our spot clusters and further specified functional interpretation in terms of associated blood compounds such as cytotoxic plasma-, T and B cells (upregulated in type 2) and erythrocytes, platelets, neutrophils, and cells of myeloid lineage (up in type 1) (Figure 3A and Supplementary Figure 12). Another study extracted aging signatures of the blood transcriptome (Peters et al., 2015). Genes of decreasing expression (“age_dn”) accumulated near spots I and J (up in type 2) while genes of increasing expression (age_up) were found in wider areas around spots A, M, and H (up in type 1) (Supplementary Figure 13). This asymmetry of the numbers of spots suggests that age_up involves a more heterogeneous collection of molecular mechanisms than age_dn (see below), which is also supported by the larger number of genes differentially upregulated (Supplementary Figure 5). Another set of signatures was obtained recently in a study of the blood transcriptomes collected from patients of sepsis framed with CAP (community acquired pneumonia) (Hopp et al., 2018b) (Figure 4F). These signatures surprisingly corresponded to signatures of nominally healthy individuals, e.g., patients with less severe CAP show signatures of type 2 transcriptomes, and while more severe CAP cases show type 1 transcriptomes associating partly with the activation of inflammatory and endotoxin tolerance characteristics (Hopp et al., 2018b).

Next, we made use of a repertoire of 382 functionally annotated expression modules extracted from a recent meta-analysis of the blood transcriptomes of 16 disease and physiological states (Altman et al., 2019) (Figure 4G and Supplementary Figure 14). Clustering of these signatures sub-stratified them into three of type 1-like clusters which were strongly affected by spot O (C1 in Figure 4G), A (C2), or C (C3), respectively. Their profiles resemble those of the different severe CAP transcriptomes and reflect inflammatory signatures, which are modulated by increased and decreased erythrocyte (spot C) and thrombocyte (spot N) activation patterns, respectively. Further, the 382 modules provided a rich repertoire of functional annotations, which supported the interpretation of our data (see example profiles in Figure 4G and Supplementary File 5 for the full set of profiles). For example, age_dn modules agreed with DNA-methylation signatures in the blood. Methylation of CpG's in the promoters or enhancers upon aging obviously repressed the transcription of the respective downstream gene (see also Supplementary Figure 1), which is in agreement with the finding that altered methylation sites enrich in aging genes (Peters et al., 2015). Moreover, we found strong enrichment of 91 of these modules in at least one of our spots (Supplementary Figure 14A). Hence, the spots provided a sort of basis set of co-regulated genes, which further expanded into a rich collection of functional annotations of different categories via a multitude of combinations as considered by our cPATs (see above).

Correlation analysis of different previous blood signature sets (Chaussabel et al., 2008; Newman et al., 2015; Peters et al., 2015; Hopp et al., 2018b; Altman et al., 2019) and our spot profiles provide very similar patterns in support of the assumption of a common modular structure of the blood transcriptome (Figure 4H and Supplementary Figure 15). Particularly, the independently obtained signatures split into two groups either positively correlating with our spot-signatures upregulated in type 1 or positively correlating with our spot-signatures upregulated in type 2 transcriptomes, respectively. Importantly, this result reflects the strikingly similar characteristics of the blood transcriptomes as seen by independent studies and verifies our blood types in the light of independent data sets.

In summary, the comparison of previous blood signatures with our data show that our spot-modules represent a sort of minimum set describing co-expression of the blood transcriptome. It expands into a rich collection of functional annotations including molecular mechanisms, cellular programs, and cell types but also lifestyle factors, diseases, and aging effects and, finally, it verifies our blood types using independent data.

### Blood Cell Signatures and Seasonal Effects

Gene sets implemented in blood cell deconvolution algorithms such as Cibersort (Newman et al., 2015) show the characteristic correlation patterns also observed in the other blood signatures (compare Figure 4H and Supplementary Figure 16). They link the expression patterns of 22 blood cell types with our spot profiles. Elevated expression (and cell fractions, Supplementary Figure 17) of monocytes, neutrophils, and eosinophils was observed in type 1 transcriptomes while overall expression of T and B cells were upregulated in type 2. Expression of M1 macrophages and dendritic cells associate with the IFN-response signature (spot L). Furthermore, signatures of monocytes, M0, and M2 macrophages were also enriched in spot L, however in combination with the inflammatory spot O, supporting the pro-inflammatory impact of these cells.

Recent studies report seasonal changes of gene expression of the blood transcriptome and of blood cell counts (de Jong et al., 2014; Goldinger et al., 2015). We found a slight shift of transcriptome characteristics toward type 1 in winter compared with summer, both for men and women (Supplementary Figure 18). It was characterized by increased expression levels of inflammation (spot A) and erythrocyte expression (spot C) and counts and decreased levels of thrombocyte characteristics (spot N) and reticulocyte and eosinophil counts (Supplementary Table 5 in Supplementary File 1). Overall, the seasonal changes of type compositions were relatively small (<3% in men and 1% in women) and were not considered further.

### Phenotype Portrayal: Blood Cell Counts, Lifestyle, Medication, and Disease History

**Figure 5:**
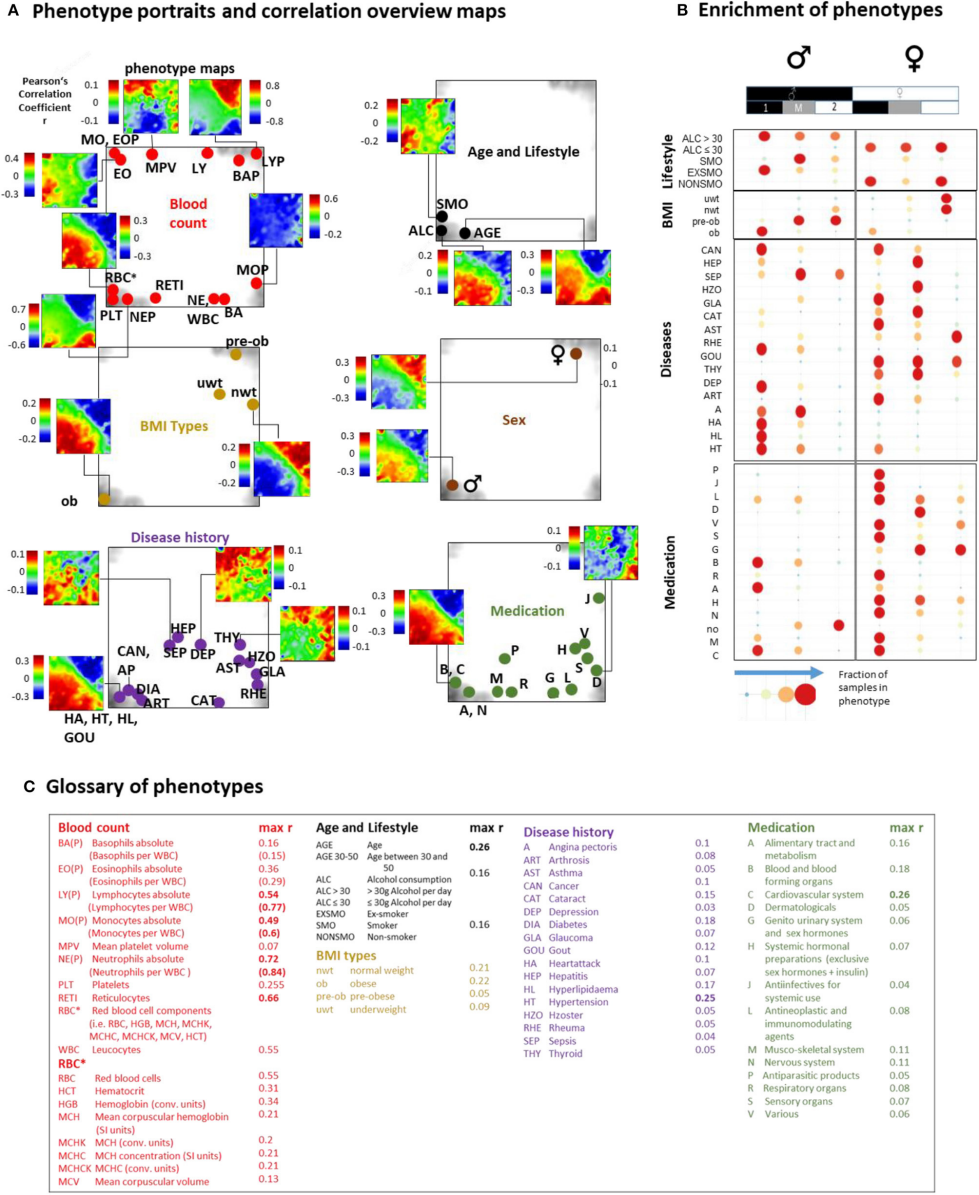
Association of selected features (phenotypes) with the transcriptome landscape of blood: **(A)** Phenotype (correlation) portraits visualize the correlation between metagene expression profiles and the profiles of selected phenotypes in a red-to-blue color scale. The correlation overview maps for each of the categories mark the metagene of maximum correlation coefficient for each of the phenotypes studied [see the legend in **(C)** and also Table S 1 for assignment of phenotypes]. **(B)** Distribution of samples of each of the phenotypes among the transcriptome types. Obese men and men consuming alcohol (>30 g/day) accumulate (red circles) in type 1 transcriptomes. Also participants with different disease histories enrich in type 1 in a gender-specific fashion while medication is most prevalent in women of type 1. Type 2 and type M refer to underweight and normal weight women and to smoking men, respectively. Enrichment of blood count data is provided in Supplementary Figure 20. Correlation maps and further details are presented for each of the phenotype categories in Supplementary Figures 21–25.

Previous blood transcriptome studies also extracted gene signatures which were associated with health-related features such as BMI (body mass index) and smoking status and also with the development of different diseases such as heart failure (Tan et al., 2002), dental caries (McLachlan et al., 2005), schizophrenia, and neoplasms (Altman et al., 2019). We find that they predominantly upregulate in type 1 transcriptomes showing characteristics of aging and/or inflammation (Supplementary Figure 19). The LIFE-adult study provided a series of features characterizing health and lifestyle of the participants in terms of the so-called phenotypes (Supplementary Table 1). We associated them with the blood transcriptomes in a participant-matched fashion using phenotype portraits, which typically showed areas of positive (colored in red) and negative (in blue) correlation between phenotype features and expression profiles in the transcriptome landscape with metagene resolution (Figure 5A, and for details Supplementary Figures 21–25). For example, phenotype associations with expression patterns of type 1 (red in the lower left part of the map) or type 2 (red in the upper right part) can be distinguished. In addition, overview maps were generated for each of the phenotype categories, which mark the metagene of maximum (and minimum) correlation for each of the phenotypes studied. The enrichment of phenotypes was evaluated in terms of the distribution of cases among the transcriptome types (Figure 5C, for enrichment significance evaluation using Fishers exact test see Supplementary Figures 21D–25D).

We found that most blood count data correlate either with type 1 (e.g., erythrocytes, reticulocytes, platelets, neutrophils) or type 2 (lymphocytes) transcriptomes in agreement with the blood cell transcriptomes analyzed above. Smokers, alcohol consumers (>30 g/day), obese and elderly people, men, and participants taking different categories of medication according to the ATC (Anatomical Therapeutic Chemicals) classification and also participants with different self-reported lifetime diseases show preferences for type 1 (and partly type M) transcriptomes while younger, under- and normal-weight participants, women, and non-consumers of medication associated preferentially with type 2. The degree of correlation with metagene expression was markedly higher for blood counts compared with the other phenotypes (Figure 5C).

Part of the blood count portraits indicated fingerprint-like correlation patterns specific for the different blood compounds (Figures 5A,B, Supplementary Figures 20, 21, and Figure 4H). The portraits of the phenotypes of the other categories partly resembled those of blood counts, this way reflecting close association between them. For example, the “aging” portrait (visualizing the correlation between age and transcriptome) can be understood as the superposition of the red blood cell (RBC)- and neutrophil (NE)-phenotype portraits indicating the increased levels of RBC and NE in elderly people (see next subsection). The “alcohol consumption” portrait also resembled the RBC-portrait while smoking revealed an eosinophil (EO)-like pattern. Increased eosinophil counts in smokers associated with lung function were reported for humans (Jensen et al., 1998; Higuchi et al., 2016) and in mouse models (Botelho et al., 2011).

Part of the medication and disease history portraits can be interpreted similarly. Namely they reflect the fact that increased usage of medication and incidences for diseases are more prevalent in elderly people (see the mean age data of each of the phenotypes listed in Supplementary Table 1) and consequently were associated with increased RBC- and NE-levels and decreased lymphocyte (LY) counts (Supplementary Figures 24, 25).

Other phenotype portraits, e.g., those of different age ranges (see next subsection) and of different medications, cannot be simply interpreted as composites of the blood count portraits. For a more detailed view we performed correlation and multiple regression analysis to estimate the particular effect of phenotypes on spot expression (Supplementary Figures 21C–F, 25C–F). We found a close relationship between high correlation coefficients and significant contributions of phenotype-coefficients (*p* < 10^−6^) especially for spots located in the lower left and upper right corners of the map. These refer, first of all, to age, obesity, gender, RBC, and white blood cell (WBC) counts, and LY, medications of the groups C (cardiovascular system) and B (blood forming organs) and the previous diseases HL (hyperlipidemia), DIA (diabetes), HT (hypertension), and CAN (cancer).

In summary, phenotype portrayal visualizes fine structures of the effect of health and lifestyle factors on the blood transcriptome. They reflect alterations of blood cell composition and presumably also the specifics of the transcriptional programs activated in the different cells. The transcriptome types (and subtypes) resolve the heterogeneity of blood transcriptomes while the spot modules provide a metric for its quantification. Overall, the phenotype portraits enable an intuitive, perception-based interpretation in terms of function and mutual associations between the different features.

### Portrayal of Aging

**Figure 6:**
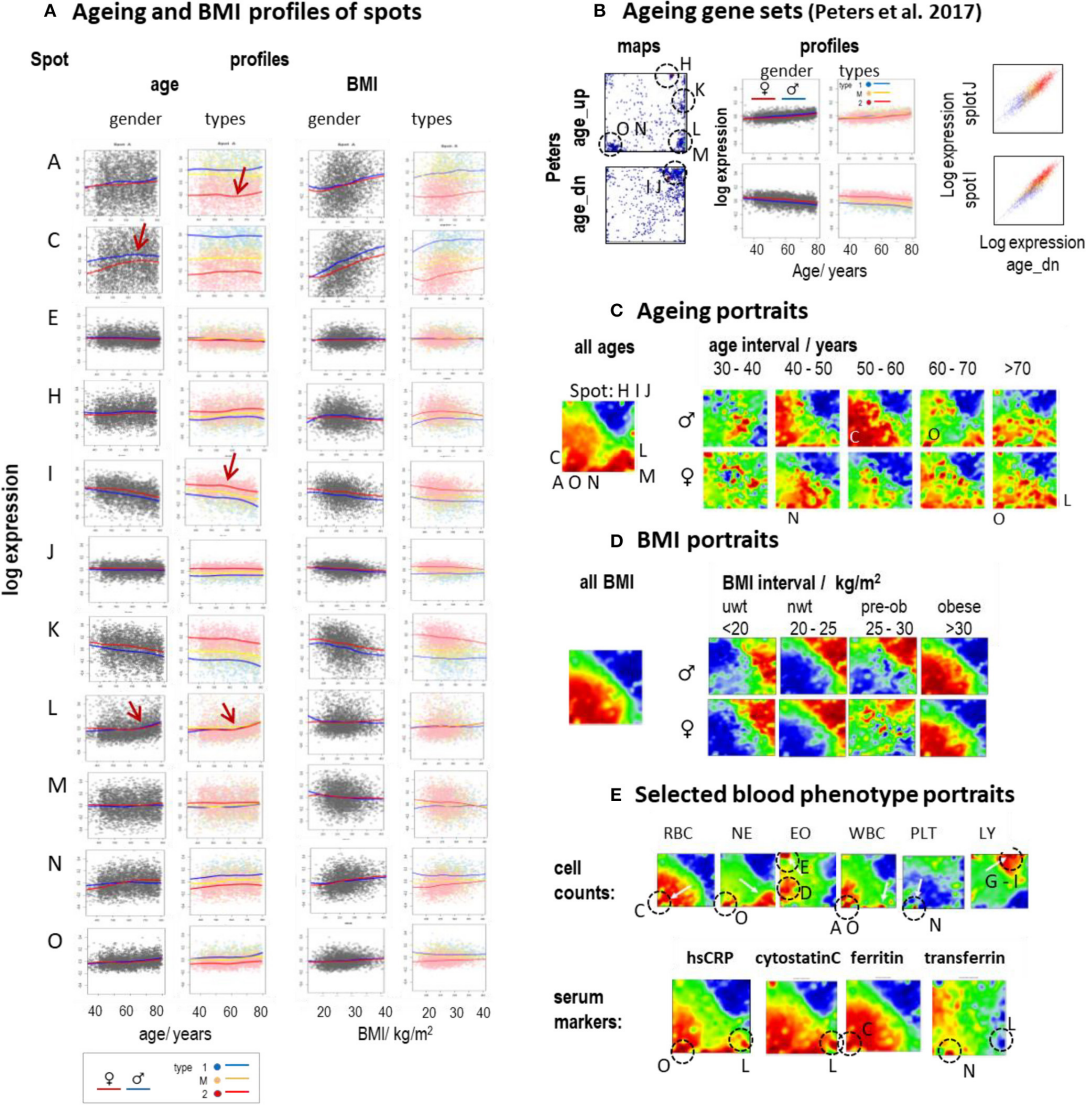
Aging and BMI characteristics of the blood transcriptome: **(A)** Expression of selected spots as a function of age and BMI. Separate LOESS (local weighted scatterplot smoothing) fits for women and men (red and blue curves) and for types 1, M, and 2 visualize mean spot expressions as a function of age and BMI. The course is mostly non-linear and change slope at different turning points (see arrows). **(B)** Genes from previous aging sets (Peters et al., 2015) spread either over a heterogeneous (age_up) or a more homogeneous (age_dn) distribution of spots (letters and dashed circles). The latter set correlates with the expression of spots I and J (correlation plots at the right). **(C)** The overall aging portrait (of correlations between the ages of the participants and the transcriptome “metagene” landscape) roughly can be understood as superposition of (increasing with age in red) RBC- and NE-like fingerprints and (decreasing with age in blue) LY-patterns. Stratification into decade intervals shows that NE-fingerprints apply mainly to elderly people (>60 years) while elevation of RBC is relevant mostly for participants younger than 60 years. **(D)** Portraits of different BMI strata reveal the continuous change from type 2 (under- and normal-weight participants) to type 1 (obese) transcriptomes. **(E)** Selected blood cell count and serum marker portraits reveal fingerprint-like patterns. Characteristic “landmark” spots are indicated by dashed circles and the respective spot letters.

Aging and alterations of the BMI are accompanied by changes of the composition of transcriptome types in a gender-specific fashion (Figure 2D). Functional analysis shows that expression of type 1_up transcriptomes gains with age while the expression of type 2_up decays on average (see the plots of age-ranked samples in Supplementary Figures 7–13, all showing enrichment of type 2 transcriptomes at younger ages and of type 1 transcriptomes at higher ages). Plots of spot expression as a function of age and BMI reveal further details (Figure 6A). Spot expressions related to red blood (spot C) and platelet (spot N) characteristics increase as a function of age and BMI with differences between the mean LOESS-curves for men and women (compare the red and blue curves) in correspondence with the blood count data (Supplementary Figure 20). In turn, the expression curves of spots related to immunity (I and J) decay with age and BMI in a nearly sex-independent fashion. On the other hand, the curves show similar courses at different levels for the transcriptomic types, which suggests type-independent aging tendencies. The aging curves are partly non-linear where the slopes get steeper for ages above 55–60 years (e.g., for spot A and I, indicative for inflammation and immune response, respectively) or above 65–70 years (spot L, IFN response), which suggests altered mechanisms in elderly people above certain age thresholds. Importantly, individual expression values of the spots show high variance about the LOESS-curves largely exceeding the mean changes observed over the age range studied between 40 and 80 years. This result suggests that the inter-individual variability of the activity of underlying molecular programs exceeds the intra-individual changes upon aging. Recent longitudinal follow-up studies on different molecular markers indeed show that inter-individual age-dependencies strongly scatter about the mean aging curve and presumably better describe aging trends than the overall curve (Alpert et al., 2019; Ahadi et al., 2020).

Note also that the scattering of individual values about the LOESS-curve is larger for spots showing increasing expression with age (e.g., spots A, C, and N) than for spots of decaying mean expression (e.g., spots I, J) which is in parallel with the larger heterogeneity of associated processes (see below). Gene maps of previous aging signatures (Peters et al., 2015) also revealed an asymmetrical distribution of aging_up and aging_dn genes (Figure 6B). The latter ones accumulated within a narrow area in and around spots I and J in the right upper corner of the map giving rise to strong correlations between signatures' expression and that of these spots. Deactivation of associated cellular functions such as immune response, telomere maintenance, and/or ribosomal and mitochondrial activities with age obviously proceed homogenously, presumably driven via mechanisms such as DNA hyper-methylation (Supplementary Figure 14). In contrast, aging_up genes distributed much more heterogeneously between different spot-regions where each of them showed a specific profile of expression gaining with age (see curves of spots A, O, N, M, L, and H in Figure 6A). Aging is obviously accompanied or even driven by the activation of a multitude of inflammatory mechanisms involving different molecular and cellular components (see spot characteristics), which combine in a patient-specific fashion giving rise to a relatively heterogeneous aging_up signature.

The mean aging portrait (“all ages” in Figure 6C) corresponds to the distribution of aging_up and aging_dn genes of the aging signature (Peters et al., 2015) (compare the respective gene set maps with the red and blue areas in Figure 6B, respectively). Moreover, the aging portrait can be roughly interpreted by the superposition of increasing RBC- and NE-like (positive correlation in red, see Glossary below and in Supplementary File 1) and decaying LY-like (negative correlation in blue) contributions (compare with the cell count portraits in Figure 6E) in agreement with the increase/decrease of the expression of the respective landmark spots C, O, and I, J, respectively. Inspection of gender- and age (decade)-stratified portraits revealed that elderly women and men (>60 years) are similarly affected by an increase of NE- and IFN-related (found especially for subtype M.3) characteristics while the RBC-like pattern (typical for subtype 1.3) is more pronounced for mid-aged men (40–60 years). Hence, mean spot signatures show either increasing or decreasing expression with age where the former was associated with inflammatory processes, red blood cell transcriptional characteristics, de-repression of epigenetically repressed cellular programs, and a higher variability of individual data compared with the decaying curves, which, in turn, associated with decaying immune response and telomere maintenance.

### Obesity and Serum Markers

The mean BMI-portrait (“all BMI” in Figure 6D) shows characteristics of type 1 transcriptomes without the NE-like patterns and the elevated expression of spot L (IFN-response) observed in the respective aging portrait. Interestingly, the BMI-stratified portraits “switch” from type 2 into type 1 for obese women and men (BMI > 30 kg/m^2^), due to gained (positive) correlations between BMI and inflammatory (spot A), RBC- (spot C), and platelet (spot N) characteristics, on one hand, and decaying immune response (spots I, J) expression signatures on the other one. Interestingly, this behavior is possibly associated with the so-called obesity-paradox claiming that an intermediate BMI about 25 kg/m^2^ is associated with minimum health risk (Wild and Byrne, 2016) and thus switches from positive to a negative effect of increasing BMI on health.

For further comparison, we generated phenotype (correlation) portraits of four selected serum protein markers (Figure 6E). The portraits of hsCRP (human serum C-reactive protein) and of cytostatin C reflect footprints of inflammation (spot O) and IFN-response (spot L) in the blood transcriptome were associated with NE-like patterns of the blood counts. The portrait of ferritin closely resembled that of RBC reflecting correspondence between the level of stored iron and erythrocyte expression (spot C). The transferrin portrait revealed a different patterns associating with the diminished spots O (inflammation) and especially L (IFN-response) and the enhanced spot N (thrombocytes), possibly due to the role of platelets in iron transport (Brieland et al., 1989). In summary, aging and obesity associate with characteristic alterations of the blood transcriptome reflecting a fine interplay between inflammatory and iron physiology as mediated by molecular (as IFN-response), cellular (e.g., WBC and RBC), and serum protein compounds.

## Discussion

We “portrayed” the diversity of the blood transcriptome of a cohort of more than 3,000 nominally healthy adult individuals included in the Leipzig Health “LIFE-adult” Study in terms of intuitive SOM-images and classified them into three major transcriptome types. The expression patterns decomposed into a minimum set of modules of co-regulated genes. Their functional impact can be interpreted based on the results of previous blood transcriptome studies. Finally, we associated the blood transcriptomes with a series of phenotype-features collected in the study for the same participants such as age, obesity-status, blood cell count, disease history, and medication by means of phenotype portraits. Overall, machine learning provided a comprehensive characterization of the diversity of the blood transcriptome taking into account the whole spectrum of transcriptional states on a population-wide scale in the context of health and lifestyle factors.

Overall, the strength of the study consisted in the large and novel set of molecular and associated phenotype data and in the comprehensive description of the blood transcriptome in terms of a holistic approach, which extracts, describes, and visualizes the multidimensional relationships between intrinsic modes of variation and their associations with health and lifestyle factors. Its limitations, on the other hand, can be seen in the fact that the visualization capabilities partly mask the evaluation of rigor and stringency in comparing different conditions, which require separate ways of presentation. Another limitation is the solely cross-sectional design, which impedes full entanglement of relations between individual and population-averaged trends.

### SOM-Portrayal Reduces Dimensions of the Blood Transcriptome

Dimension-reduction and feature extraction are important issues in high-throughput data analysis (Binder et al., 2015; de Meulder et al., 2018). Our machine-learning approach reduces the dimensionality of data into a handful transcriptome types and subtypes (Binder and Wirth, 2014). Their expression patterns were governed by about one dozen expression (spot-) modules in close correspondence and agreement with previous signatures of the blood transcriptome (Chaussabel et al., 2008; Peters et al., 2015; Hopp et al., 2018b; Altman et al., 2019). Moreover, data portrayal transforms high-dimensional data landscapes into easy-to-interpret images. Their visual inspection strongly supports analytic tasks on different levels of stratification ranging from individual “personalized” to subtype- and type-averaged expression portraits. Our study thus provided a sort of album of transcriptomic “faces” of the LIFE participants (Supplementary File 2). Importantly, the phenotype portrayal projects low dimensional features such as age or BMI onto the high-dimensional transcriptome landscape, which generates highly granular correlation images serving as a “fingerprint” of the respective phenotype.

**Figure 7:**
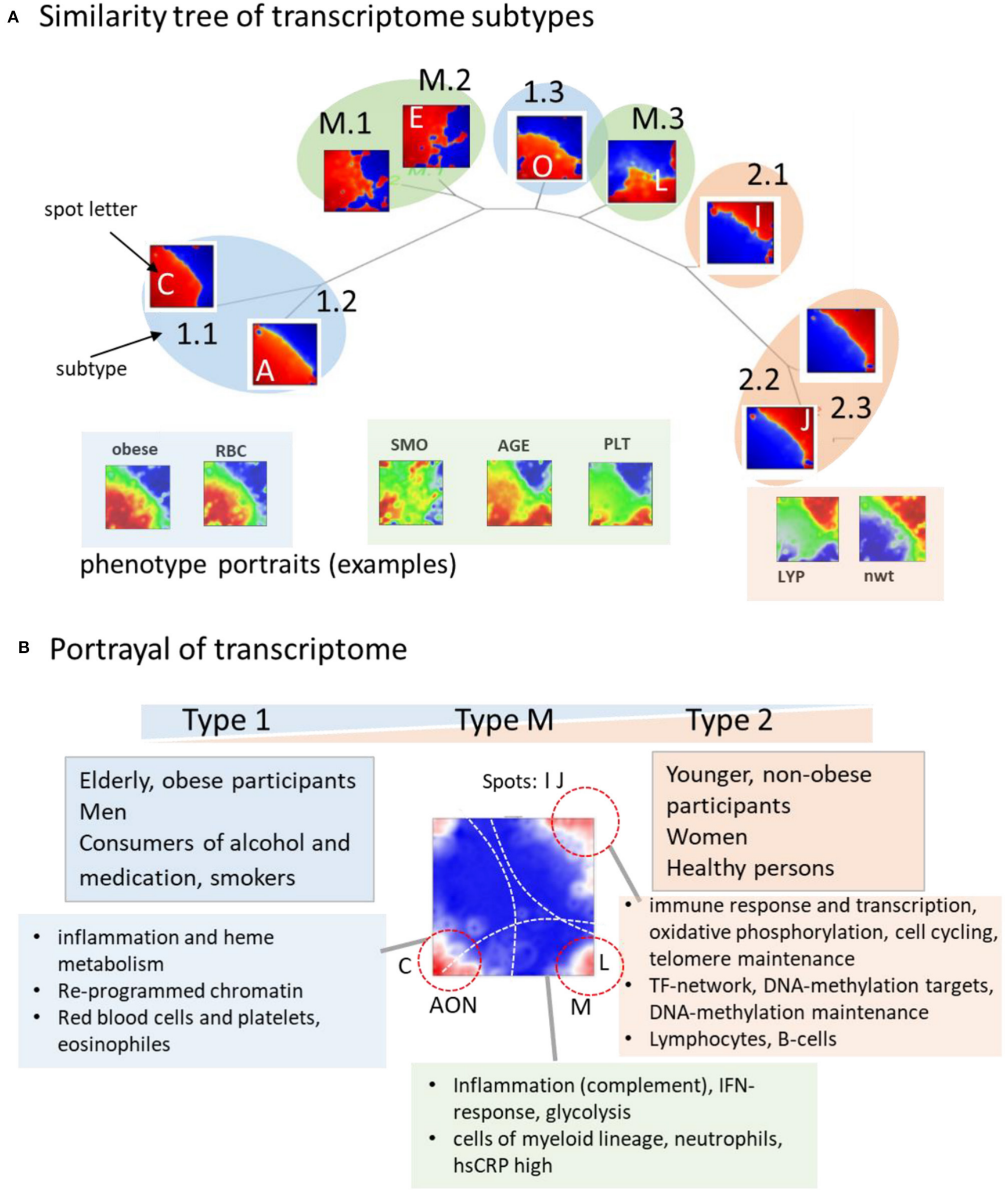
Portrayal of the blood transcriptome: **(A)** The similarity tree reflects a virtually linear arrangement of subtypes due to a continuum of transcriptional states ranging from type 1 to type 2. Selected phenotype portraits illustrate correlation of the respective features with the transcriptomes of different types. **(B)** Main functions and phenotypes associating with the transcriptome types.

The tree in Figure 7A illustrates the similarities between the subtype portraits, which are virtually linearly arranged along a common backbone. The portraits at the left and right margins (type 1-vs.-type 2) differ mainly in the antagonistic expression of genes located in opposite corners of their portraits. Our analysis thus uncovered a striking simplicity of the transcriptome at the coarsest level of approximation. It reflects characteristic alterations of transcriptional programs referring to different cell components, namely a decrease in signatures of myeloid-lineage cells and an increase of signatures of lymphocytes from the left to the right. The transcriptional (spot-) modules diversify these basic patterns in a subtype-specific fashion. Namely it indicates continuous expression change along the subtypes related to immune response (spots I, J) and cytotoxic cells (H) with potential impact for longevity, and, in addition, also subtype-specific expression related to erythrocytes and platelets (C, N) giving rise to gender-specific differences. A third category shows the activation patterns spread over all subtypes related to IFN-response reflecting partly viral infections. It increases, on average, in elderly people especially above 65 years.

### Footprints of Aging, Telomere Maintenance, and Epigenetics

On a cross sectional population scale our data provide information about aging between mid-life (30–50 years) and elderly (70–80 years) women and men. In addition to the systematic changes of inflammation characteristics and immune response, aging relates to epigenetic factors and to telomere length dynamics (Figure 7B). Telomeres serving as protective nucleoprotein structures that cap the ends of chromosomes shorten systematically with age in result of repeated cell divisions (Mather et al., 2010). Telomere maintenance mechanisms counteract this process and thus their activation can be indicative for counteracting cell aging (Shawi and Autexier, 2008; Codd et al., 2013). We found that the expression of genes involved in the telomerase-maintenance pathway (Nersisyan et al., 2019) were more active in type 2 transcriptomes, which associated with younger and healthy individuals. The drop of telomere maintenance activity in type 1 transcriptomes indicates that aging and the worsening of health status is associated with the weakening of telomere maintenance, which associates with the shortening of leukocyte telomere lengths in the course of age-related diseases (Oeseburg et al., 2010; Haycock et al., 2014). Moreover, decay of telomere length with age (Lapham et al., 2015) resembles the decay of the amount of type 2 transcriptomes with age. Women, having a higher fraction of type 2 transcriptomes with activated telomere maintenance mechanisms possess on average longer telomeres than men (Gardner et al., 2014; Lapham et al., 2015). Overall, a cell's ability to maintain telomeres is associated with better immune responsibility and a general health constitution especially in younger, non-obese, non-smoking, and non-alcohol consuming people.

Our analysis also emphasizes the importance of epigenetic mechanisms, particularly of chromatin (re-) organization for changes of the blood transcriptome. We found a pronounced mutual switching between type 1 and type 2 transcriptomes using gene expression of nominally repressed and activated chromatin states in blood cells as an indicator of gene activity. This result suggests that part of active states in type 2 become repressed in type 1 and vice versa, that part of repressed states in type 2 become activated in type 1. Hence, part of the expression changes observed were associated with changed chromatin organization leading to altered cell function as discussed in the context of aging (Ciccarone et al., 2018) and inflammation (Busslinger and Tarakhovsky, 2014; Daniel et al., 2018; Ray and Yung, 2018; Lorente-Sorolla et al., 2019). DNA-methylation is typically linked to chromatin states by different mechanisms (Hopp et al., 2015a,c). Indeed, DNA-methylation signatures change expression in parallel with the chromatin state signatures particularly between type 1 and type 2 transcriptomes. The DNA-methylation maintenance methyltranferase DNMT1 is part of the type2_up (spot J) signature showing decaying expression with age and correlating with the DNA-methylation signature (Supplementary Figure 14). This finding supports previous assumptions that aging methylation signatures, so-called DNA-methylation clocks, reflect the activity of the epigenetic maintenance system (Horvath, 2013). Note however, that there is only weak correlation between DNA-methylation and transcriptome age predictors, which were obtained independently (Peters et al., 2015). Transcriptomic and the epigenetic predictors describe probably different aspects of biological aging. One possible reason can be seen in the fact that transcriptomic and epigenetic mechanisms partly decouple upon aging in a similar way as reported for cancer development (Hopp et al., 2018a; Binder et al., 2019) and cell differentiation (Thalheim et al., 2018). Coupled transcription, DNA-methylation, and telomere length epidemiological studies are required to better disentangle the relationship between these features of the blood transcriptome (Bell et al., 2019).

Transcriptome typing and modularization describes the effect of age and BMI on the blood transcriptome, and in a wider context, on a human's physiology via association with lifestyle characteristics. The percentage of type 1 transcriptomes in the population relating to inflammation gains with age and, to a less degree, with BMI in a non-linear, gender-specific fashion. It is known that obesity is associated with leukocytosis representing a state of chronic low-grade inflammation (Herishanu et al., 2006; Johannsen et al., 2010), which, in turn is considered a driver of many age-related disorders (inflammo-aging) (Wu et al., 2015). We found a striking overlap of signatures shared by multiple diseases, aging, and obesity driven by an underlying common pattern in agreement with (Wang et al., 2016). We also found an agreement with the blood transcriptomes of patients suffering from severe sepsis framed by community acquired pneumonia (Hopp et al., 2018b), which revealed tree axes of variation, namely an inflammatory-vs.-immune response one (endotoxin tolerance, cytotoxic cells), a “blood-disturbance” axis including mostly erythrocyte and thrombocyte characteristics, and the IFN-response axis. They combine in different relations where the number of states is higher in type 1 compared with type 2 transcriptomes. This asymmetry reflects multi-factorial activation mechanisms potentially accompanying aging, disease development, and unhealthy lifestyle factors such as smoking and alcohol consumption (see Peters et al., 2015 and Supplementary Figure 13). On the other hand, these results suggest that the diversity of the blood transcriptome is governed by a relatively high inter-individual variability along these axes on a first level. Age- and lifestyle-related systematic trends form a second layer, which is further modulated by the actual health (or disease) status of the individuals, e.g., in the case of severe sepsis by the strong activation of inflammatory signatures (Hopp et al., 2018b). Recent longitudinal studies revealed that individuals are more similar to their own expression profiles later in life than to profiles of other individuals of their own age (Alpert et al., 2019; Balliu et al., 2019; Ahadi et al., 2020). Individual aging patterns, so-called “ageotypes” can be defined on the basis of molecular pathways that changed over time in a given individual reflecting personal aging as a result of personal lifestyle and medical history (Ahadi et al., 2020). Longitudinal follow-up studies over different age ranges are required to study individual “life-courses” of the blood transcriptome and their impact for lifetime-risk prediction.

## Conclusions

Machine learning offers a promising option to analyze omics data sets in the epidemiological context. We characterized the human blood in terms of transcriptome types and functional gene modules and their association with health-, lifestyle- and age-related phenotypes. It has impacts for future applications for diagnosis and prognosis via the refinement of existing and the development of novel predictors for age, lifestyle, and disease outcomes. The individual portrayal of transcriptomes and of their associations with phenotype features in terms of easy-to-interpret images offers perspectives for visual perception-based personalized diagnostics. Large scale longitudinal studies and paired transcriptome-epigenome investigations are needed to better understand lifetime courses, causal relationships, and mechanisms of (epi-)genomic regulation.

## Data Availability Statement

The data that support the findings of this study are available from the LIFE center but restrictions apply to the availability of these data, which were used under license for the current study, and so are not publicly available. Data are however available from the authors upon reasonable request and with permission of LIFE. Secondary data are available as SOM-data via the Leipzig Health Atlas under the link https://www.health-atlas.de/data_files/76?version=1 and https://www.health-atlas.de/som_browser/201611_LIFE_Transcriptome/Summary.html (pdf and html reports). Data can be interactively discovered using the oposSOM browser functionality available under https://www.izbi.uni-leipzig.de/opossom-browser/ and https://apps.health-atlas.de/opossom-browser/?dataset=5.

## Ethics Statement

The studies involving human participants were reviewed and approved by ethics board of the Medical Faculty of the University of Leipzig. The patients/participants provided their written informed consent to participate in this study.

## Author Contributions

HB and HL-W: conceived the study. MS and HB: wrote this paper. MS, LH, HB, and HL-W: performed analysis. HL-W, MS, and AA: downstream analysis methods development. HK: preprocessing of transcriptomics data. KW, CE, RB, and KK: collection and curation of phenotype data. ML and JT: coordinators of LIFE research center. All authors read and approved the final manuscript.

## Conflict of Interest

The authors declare that the research was conducted in the absence of any commercial or financial relationships that could be construed as a potential conflict of interest.

## Glossary

A: Alimentary tract and metabolism
AP: Angina pectoris
ALC > 30: Participants consuming more than 30g alcohol per day
ALC ≤ 30: Participants consuming less than 30g alcohol per day
ALC: Alcohol consumption
ART: Arthrosis
AST: Asthma
ATC: Anatomical Therapeutic Chemical classification system of medication
B: Blood and blood forming organs
BA: Basophils absolute (10^9^/l)
BAP: Basophils (%)
BD: Sepsis type “blood disturbant”
BMI: Body mass index
BP: GO-term “biological process”
C: Cardiovascular system
CAN: Cancer
CAP: Community acquired pneumonia
CAT: Cataract
cPAT: Combinatorial pattern type
CTL: Cytotoxic T lymphocytes
D: Dermatologics
DEP: Depression
DIA: Diabetes
DNMT1: DNA-methylation maintenance methyltranferase
EO: Eosinophils absolute (10^9^/l)
EOP: Eosinophils (%)
EXSMO: Ex-smoker
G: Genitourinary system and sex hormones
GLA: Glaucoma
GO: Gene ontology
GOU: Gout
GSZ: Gene set enrichment z-score
H: Systemic hormonal preparations, excl. sex hormones and insulins
HA: Heart attack
HCT: Hematocrit (l/l)
HEP: Hepatitis
HGB: Hemoglobin (SI units, mmol/l)
HGBK: Hemoglobin (conv. units, g/dl)
HL: Hyper-lipidemia
HS: Sepsis type “high severity”
hsCRP: Human serum C-reactive protein
HT: Hypertension
HZO: Hzoster
IFN: Interferon
J: Anti-infective for systemic use
L: Antineoplastic and immunomodulating agents
LIFE(-adult): Leipzig Research Center for Civilization Diseases
LOESS: Locally estimated scatterplot smoothing
LS: Sepsis type “healthy and low severity”
LY: Lymphocytes absolute (10^9^/l)
LYP: Lymphocytes (%)
M: Muscular-skeletal system
MCH: Mean corpuscular hemoglobin (SI units, fmol)
MCHC: Mean corpuscular hemoglobin concentration (SI units, mmol/l)
MCHCK: Mean corpuscular hemoglobin concentration (conv. units, g/dl)
MCHK: Mean corpuscular hemoglobin (conv. units, pg)
MCV: Mean corpuscular volume (fl)
MO: Monocytes absolute (10^9^/l)
MOP: Monocytes (%)
MPV: Mean platelet volume (fl)
MS: Sepsis type “medium severity”
N: Nervous system
NE: Neutrophils absolute (10^9^/l)
NEP: Neutrophils (%)
NONSMO: Non-smoker
nwt: Normal weight
ob: Obese
P: Antiparasitic products, insecticides, and repellents
PLT: Platelets (10^9^/l)
PRC2: Polycomb repressive complex 2
pre-ob: Pre-obese
R: Respiratory system
RBC: Erythrocytes (10^12^/l)
RETI: Reticulocytes (/1000)
RHE: Rheuma
S: Sensory organs
SEP: Sepsis
SMO: Smoker
SMO: Smoking
SOM: Self-organizing maps
ST: Subtype
TF: Transcription factor
THY: Thyroid
TM: Telomere length maintenance
TssA: Genes with active promoter
Tx: Genes with completed transcription
uwt: Underweight
V: Various
WBC: Leucocytes (10^9^/l)
WPB: Whole peripheral blood.
