# Supplementary material for: The Human Blood Transcriptome in a Large Population Cohort and Its Relation to Aging and Health
Source: Front Big Data. 2020 Oct 30;3:548873. doi: 10.3389/fdata.2020.548873 (PMC7931910; doi:10.3389/fdata.2020.548873)
Supplement: Supplementary file 1 [file Data_Sheet_1.pdf]

Supplementary File 1:
Glossary, Supplementary Methods,Supplementary Figures S1 – S22 and Supplementary Tables S1 – S5

Machine learning-based analysis of the human blood transcriptome in a population cohort and its relation to ageing and health

Maria Schmidt [1], Lydia Hopp [1], Arsen Arakelyan [2], Holger Kirsten [3,4], Christoph Engel [3,4], Kerstin Wirkner [3,4], Knut Krohn [4,5], Ralph Burkhardt [4,5], Joachim Thiery [4,5], Markus Loeffler[1,3,4], Henry Loeffler-Wirth [1§], Hans Binder [1,4§*]

1 IZBI, Interdisciplinary Centre for Bioinformatics, Universität Leipzig, Härtelstr. 16 – 18, 04107 Leipzig, Germany

2 BIG, Group of Bioinformatics, Institute of Molecular Biology, National Academy of Sciences, Yerevan, Armenia

3 IMISE, Institute for Medical Informatics, Statistics and Epidemiology; University of Leipzig; Leipzig; Germany

4 LIFE, Leipzig Research Centre for Civilization Diseases; University of Leipzig; Leipzig; Germany

5 Institute of Laboratory Medicine, Clinical Chemistry and Molecular Diagnostics, University of Leipzig, Leipzig, Germany

* Author to whom correspondence should be addressed;
§ shared senior authorship

Content

1. Glossary 2
2. Supplementary Methods 4
   2.1. Preparation of blood mRNA and preprocessing of microarray data 4
3. Supplementary Figures 5
   3.1. SOM portrayal and spot patterns 5
   3.2. Subtyping and spot profiles 7
   3.3. Functional characteristics 12
   3.4. Previous signatures of the blood transcriptome and blood cell deconvolution 15
   3.5. Phenotype characteristics and associations with the blood transcriptome 22
4. Supplementary Tables 30
5. References 40

1. Glossary

A	Alimentary tract and metabolism
AP	Angina pectoris
ALC > 30	Participants consuming more than 30g alcohol per day
ALC ≤ 30	Participants consuming ess than 30g alcohol per day
ALC	Alcohol consumption
ART	Arthrosis
AST	Asthma
ATC	Anatomical Therapeutic Chemical Classification System of medication
B	Blood and blood forming organs
BA	Basophils absolute (10^9/l)
BAP	Basophils (%)
BD	Sepsis type „Blood disturbant“
BMI	Body mass index
BP	GO-term „Biological process“
C	Cardiovascular system
CAN	Cancer
CAP	Community Aquired Pneumonia
CAT	Cataract
cPAT	Combinatorial pattern type
CTL	Cytotoxic T lymphocytes
D	Dermatologics
DEP	Depression
DIA	Diabetes
DNMT1	DNA-methylation maintenance methyltranferase
EO	Eosinophils absolute (10^9/l)
EOP	Eosinophils (%)
EXSMO	Ex-smoker
G	Genitourinary system and sex hormones
GLA	Glaucoma
GO	Gene Ontology
GOU	Gout
GSZ	Gene set enrichment z-score
H	Systemic hormonal preparations, excl. sex hormones and insulins
HA	Heart attack
HCT	Hematocrit (l/l)
HEP	Hepatitis
HGB	Hemoglobin (SI units, mmol/l)
HGBK	Hemoglobin (conv. units, g/dl)
HL	Hyper-lipidaemia
HS	Sepsis type „High severety“
hsCRP	human serum C-reactive protein
HT	Hypertension
HZO	Hzoster
IFN	Interferon
J	Anti-infective for systemic use

L	Antineoplastic and immunomodulating agents
LIFE(-adult)	Leipzig Research Center for Civilization Diseases
LOESS	locally estimated scatterplot smoothing
LS	Sepsis type „Healthy & Low severety“
LY	Lymphocytes absolute (10^9/l)
LYP	Lymphocytes (%)
M	Muscular-skeletal system
MCH	Mean corpuscular hemoglobin (SI units, fmol)
MCHC	Mean corpuscular hemoglobin concentration (SI units, mmol/l)
MCHCK	Mean corpuscular hemoglobin concentration (conv. units, g/dl)
MCHK	Mean corpuscular hemoglobin (conv. units, pg)
MCV	Mean corpuscular volume (fl)
MO	Monocytes absolute (10^9/l)
MOP	Monocytes (%)
MPV	Mean platelet volume (fl)
MS	Sepsis type „Medium severety“
N	Nervous system
NE	Neutrophils absolute (10^9/l)
NEP	Neutrophils (%)
NONSMO	Non-smoker
nwt	Normal weight
ob	Obese
P	Antiparasitic products, insecticides and repellents
PLT	Platelets (10^9/l)
PRC2	Polycomb repressive complex 2
pre-ob	Pre-obese
R	Respiratory system
RBC	Erythrocytes (10^{12}/l)
RETI	Reticulocytes (/1000)
RHE	Rheuma
S	Sensory organs
SEP	Sepsis
SMO	Smoker
SMO	Smoking
SOM	Self-organizing maps
ST	Subtype
TF	Transcription factor
THY	Thyroid
TM	Telomere length maintenance
TssA	Genes with active promoter
Tx	Genes with completed transcription
uwt	Underweight
V	Various
WBC	Leucocytes (10^9/l)
WPB	Whole peripheral blood

2. Supplementary Methods

2.1. Preparation of blood mRNA and preprocessing of microarray data

Holger Kirsten

IMISE, Institute for Medical Informatics, Statistics and Epidemiology; University of Leipzig; Leipzig; Germany

In this study we used preprocessed gene expression data as provided by the LIFE data base. The process from participant recruitment, blood collection, storage and mRNA preparation, microarray measurements and primary data preprocessing was realized by different groups of the LIFE center. Accordingly, whole blood was collected in Tempus Blood RNA Tubes (LIFE Technologies) and relocated to -80 °C before further processing. Isolated RNA was processed and hybridized to Illumina HT-12 v4 Expression BeadChips (Illumina, San Diego, CA, USA) and measured on the Illumina HiScan. Raw data of all 47,323 probes was extracted by Illumina GenomeStudio in all 3,489 included individuals. Data was further processed within R version 3.1.0/Bioconductor (1). 0.4% of all samples showing an extreme number of expressed genes (defined as median ± 3 interquartile ranges (IQR) of the cohort's values) were excluded. Transcripts not found to be expressed according to Illumina's internal cut-off as implemented in Bioconductor package "lumi" $p \leq 0.05$ in at least 5% of all samples were not further considered in the analysis (2). Expression data was quantile normalized and log2-transformed (Schmid et al., 2010). For further outlier detection, the Euclidian distance was calculated between all individuals and an artificial individual having average expression levels of all transcripts after removing 10% samples farthest away from the average of all samples (implemented in the R / Bioconductor package lumi (2). 0.2% of the initially included samples with a distance larger than median + 3 IQR were filtered out. Furthermore, for each sample a combined quantitative measure combining quality control features available for HT-12 v4 was defined. This included perfect-match and miss-match control probes, control probes present at different concentrations, mean of negative control probes, mean of house-keeping genes, number of expressed genes, mean signal strength of biotin-control-probes, and ERCC-spike in probes (3). The Mahalanobis-distance between all samples and an artificial individual average value for these quality control features was calculated using R/Bioconductor package mdqc (4). Following, 2.3% of the included samples with a distance larger than median + 3 IQR were filtered out. Transcript levels were corrected for the known batch effect due to being processed on the same expression chip using an empirical Bayes method (5). Success of adjustment was verified using ANOVA for both, the expression chip used as well as the processing batch. Mapping of genes corresponding to expression probes and assignment of gene names was done using a remapping approach (6) applying gene-information of the Entrez gene database of the National Center for Biotechnology Information (NCBI, http://www.ncbi.nlm.nih.gov/gene) using illuminaHumanv4.db_1.14.0 (hg19). After further filtering based on a quality score of at least level "good" from this remapping approach (6) a total of 19,049 valid gene-expression probes were available in the 3,388 valid individuals.

3. Supplementary Figures

3.1. SOM portrayal and spot patterns

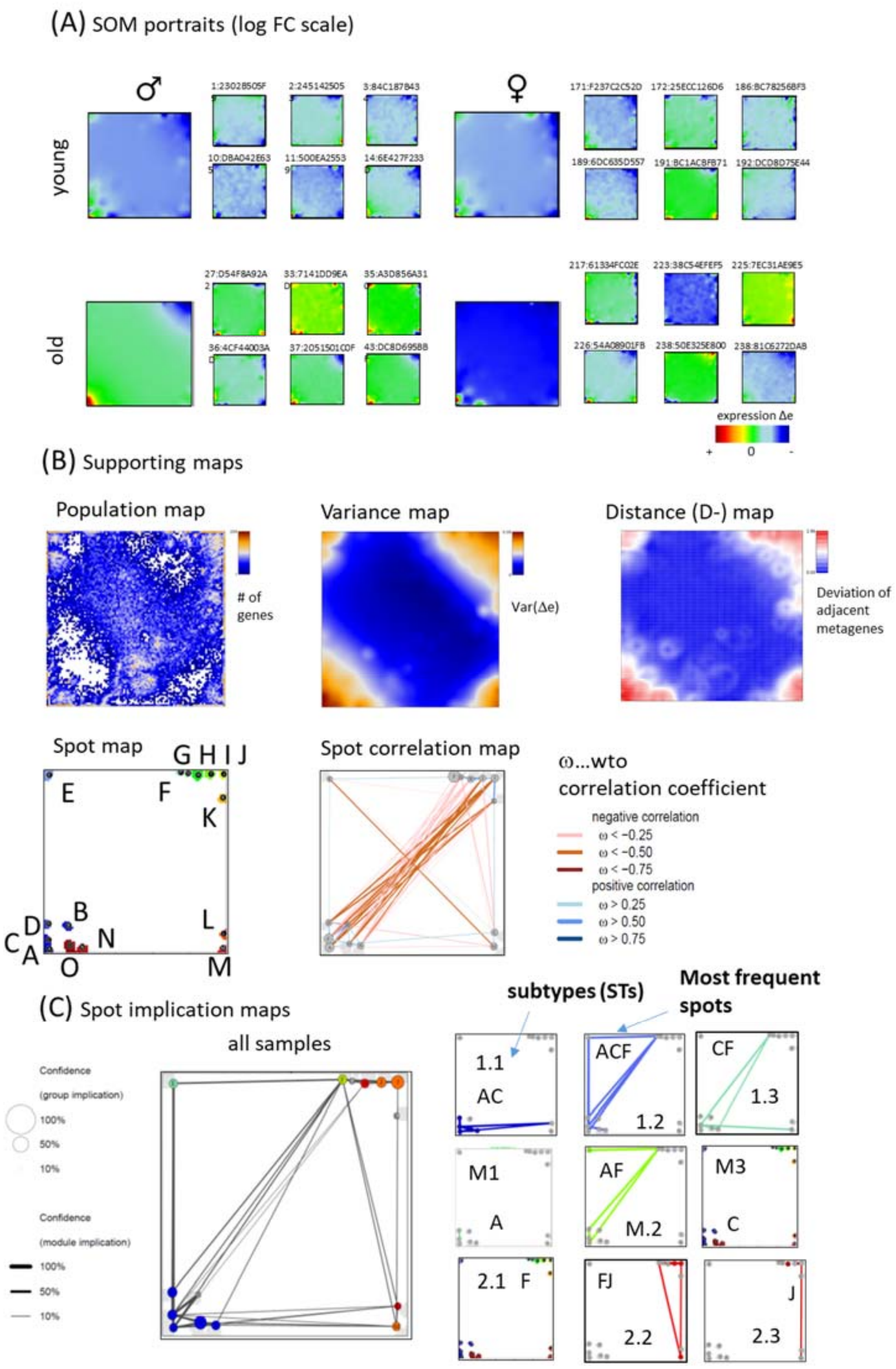

Figure S 1: SOM portrayal of blood transcriptomes, spot selection and spot combinatorics: (A) Mean expression portraits of men (left part) and women (right part) younger (part above) and older (part below) than 50 years, respectively. Examples of individual expression portraits take from the respective strata are shown in smaller size. (B) Supporting maps of the blood transcriptome (see also (7) for methods description): The population map visualizes the number of genes in each of the metagenes. It ranges from empty metagenes (white, no gene), via blue

(one gene) and gray to a maximum of more than 200 single genes per metagene (maroon color). Genes accumulate especially in the left lower and right upper corners of the map. The variance map visualizes the variance of metagene expression. Highly variant metagenes accumulate near the corners of the map (maroon color), especially in the left lower and right upper corners. The D-map visualizes the Euclidian distance between the profiles of adjacent metagenes, which is small in the areas of high metagene variance (red color) and larger in the area of invariant metagenes (blue areas). Both type of regions are separated by white 'envelopes', which mark the transitions between different shapes of profiles showing local maxima of mutual distance. These areas of different profile types are subsumed as 'spots' using in addition an overexpression criterion of their mean metagene expression (>90% percentile of all metagene expression values in a selected sample). Note also that areas of different profiles are separated often by regions of empty metagenes because SOM training stretches the distance metrics in these areas (see the white regions in the population map). The spot map summarizes the spots areas obtained after segmentation of the map using the 90 percentile criterion. The spots were labelled with capital letters A – O. The spot correlation map connects spots showing positive (blue) or negative (red) correlations using the weighted topological overlap (wto) metrics (8). Especially spots connected along the positive diagonal are mutually anti-correlated. (C) Spot implication analysis calculates the probability of concerted spot appearance in the individual portraits (see (9) for details). Spots observed frequently together are connected by lines. The larger map on the left shows all implications observed while the smaller maps on the right show the implications observed in selected subtypes (STs, see also Figure S 2). The STs associate with specific patterns of mutual co-occurrence of spots.

3.2. Subtyping and spot profiles

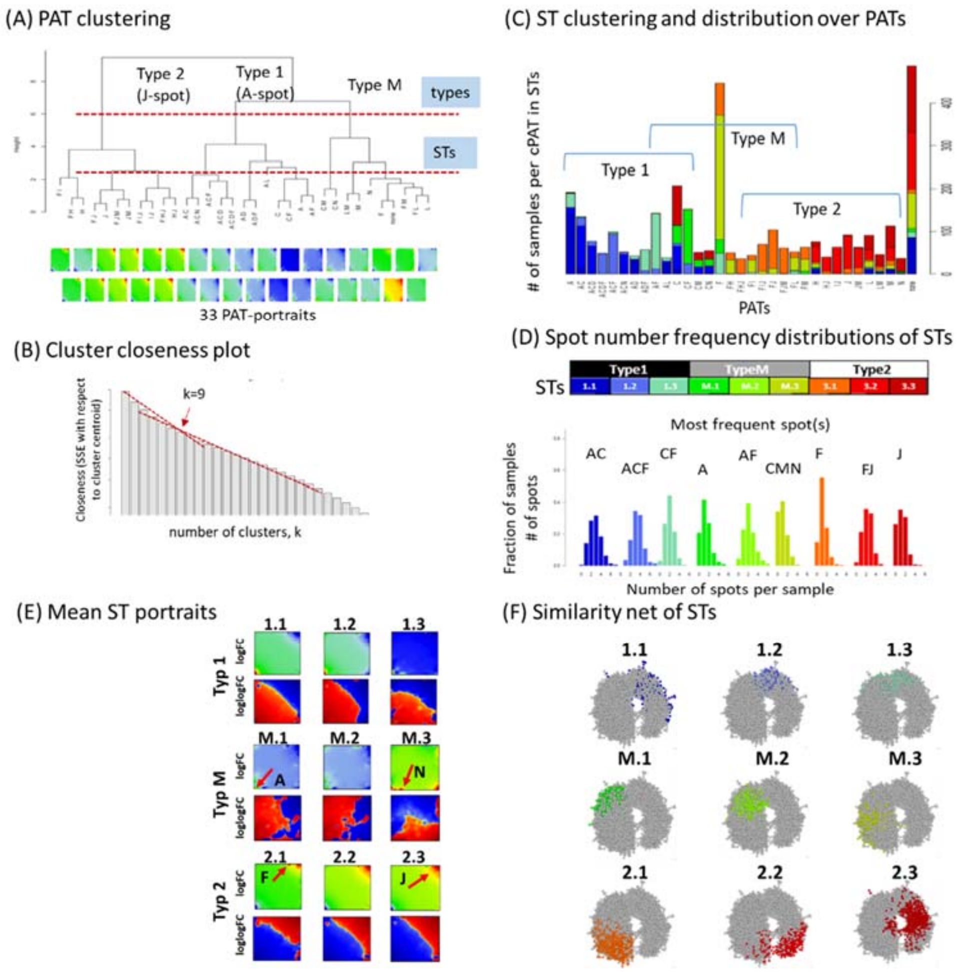

Figure S 2: Typing of transcriptome samples and subtype characteristics: (A) All 3,388 transcriptomic portraits were stratified into 33 PATs (pattern types, see also {Loeffler-Wirth, 2019 #3362}). Each PAT is defined by a unique spot patterns, i.e. the unique combinatorial co-appearance of spots as assigned by the spot letters (see also Figure S 1C). Their portraits group into three major clusters (assigned as 'types') and 9 more granular subtype-level clusters (STs) using two different distances in the hierarchical clustering tree as marked by the two horizontal dashed lines, respectively. (B) Cluster closeness is calculated as the mean squared distance between all cluster members and the cluster centroid. They are plotted as a function of the number of clusters used in supervised K-means clustering of PATs. The logged closeness decays virtually linearly showing a breakpoint of changing slope near k=9 which provides reasonable granularities for the stratification of samples into subtypes. (C) Based on this estimation of reasonable cluster numbers we performed a new supervised K-means clustering run, which stratifies all transcriptome portraits into nine subtypes (STs) and three transcriptome types, where three STs aggregate together into each of the transcriptome types. Each stacked bar plot shows distribution of the respective PAT over STs. The ST- distribution among PATs shows enrichment of spot A expressing PATs in type 1, spot J containing PATs in type 2 and spot C expressing PATs in type M. (D) The spot number frequency distributions counts samples from each ST showing 1, 2, 3 etc. spots. Part of the STs are dominated by patterns expressing only two spots while others express typically three or more spots in parallel. The broader distribution of spot numbers in the latter STs reflects a more heterogeneous expression landscape compared with STs which are dominated by two spots only. (E) Mean expression portraits of the STs are shown using two different scales: In log-scale the logged metagene expression values are color-coded from blue-to-red. It sharply identifies spot areas of strong over- and underexpression, respectively. The log-log scale provides a 'more flat' expression-landscape which splits into areas of over (red) and under (blue) expression (7). This color scale reveals larger areas of over- and under-expression in a type- and ST-

specific fashion. (F) The samples of each ST accumulate in certain regions of the similar network, which reflects the mutual similarities of the included individual portraits.

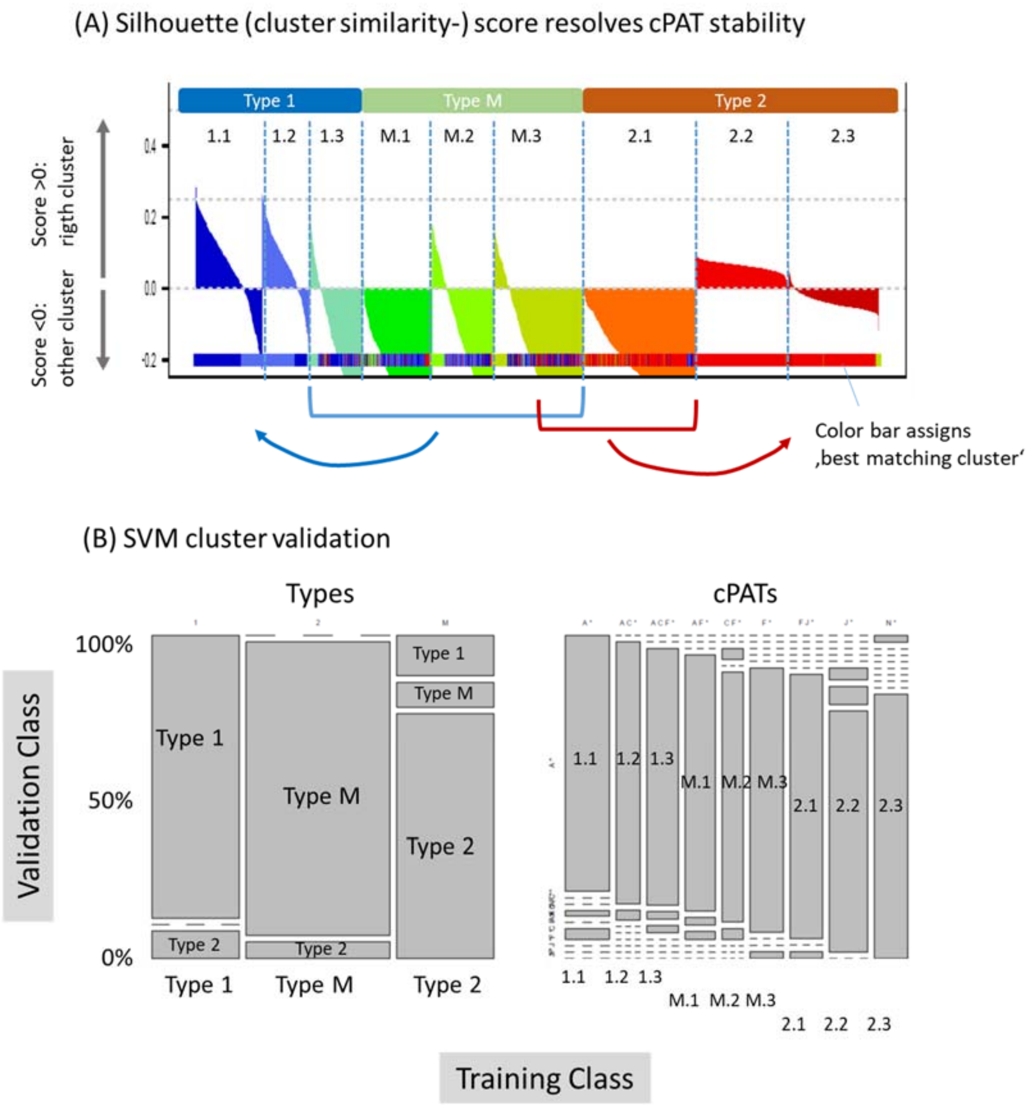

Figure S 3: Cluster stability and verification of stratification of samples: (A) The silhouette score (ssc) estimates 'best matching' cluster for each transcriptome sample. It is calculated as the difference of the Euclidian distances between all metagenes of the sample of interest and the respective cluster centroid or the next-nearest centroid of another cluster {Kunz, 2018 #3359}. The ssc-value is positive if the sample is in the cluster of minimum Euclidian distance and it is negative if the Euclidian distance to another cluster is minimum. The color bar below the silhouette plot assigns the cluster of minimum distance. The plot considers cPAT resolution of stratification. One sees that Type 1 and type M samples are stable within type 1, while type 2 samples are stable within type 2 cluster. Note that clustering as described in Figure S 2 is based on PATs while the ssc uses metagene data. The silhouette analysis thus confirms stability of type 1 and 2 clusters. (B) Training-versus-verification data clustering: The sample were randomly split into training (90%) and verification (10%) cohort. Then a Support Vector Machine (SVM, R-package 'e1071') was trained according to type (or cPAT, left and right part of the figure, respectively) stratifications using training data. Subsequently verification data were classified by means of SVM. Resampling (10x) provided 89% to 92% (92% to 95%) properly verified types (cPATs) assignments. Class discovery (training of SVM) and classification (of verification data) were based on spot data used for PAT assignments. Hence, stratification into types and cPATs is stable for roughly 90% of samples.

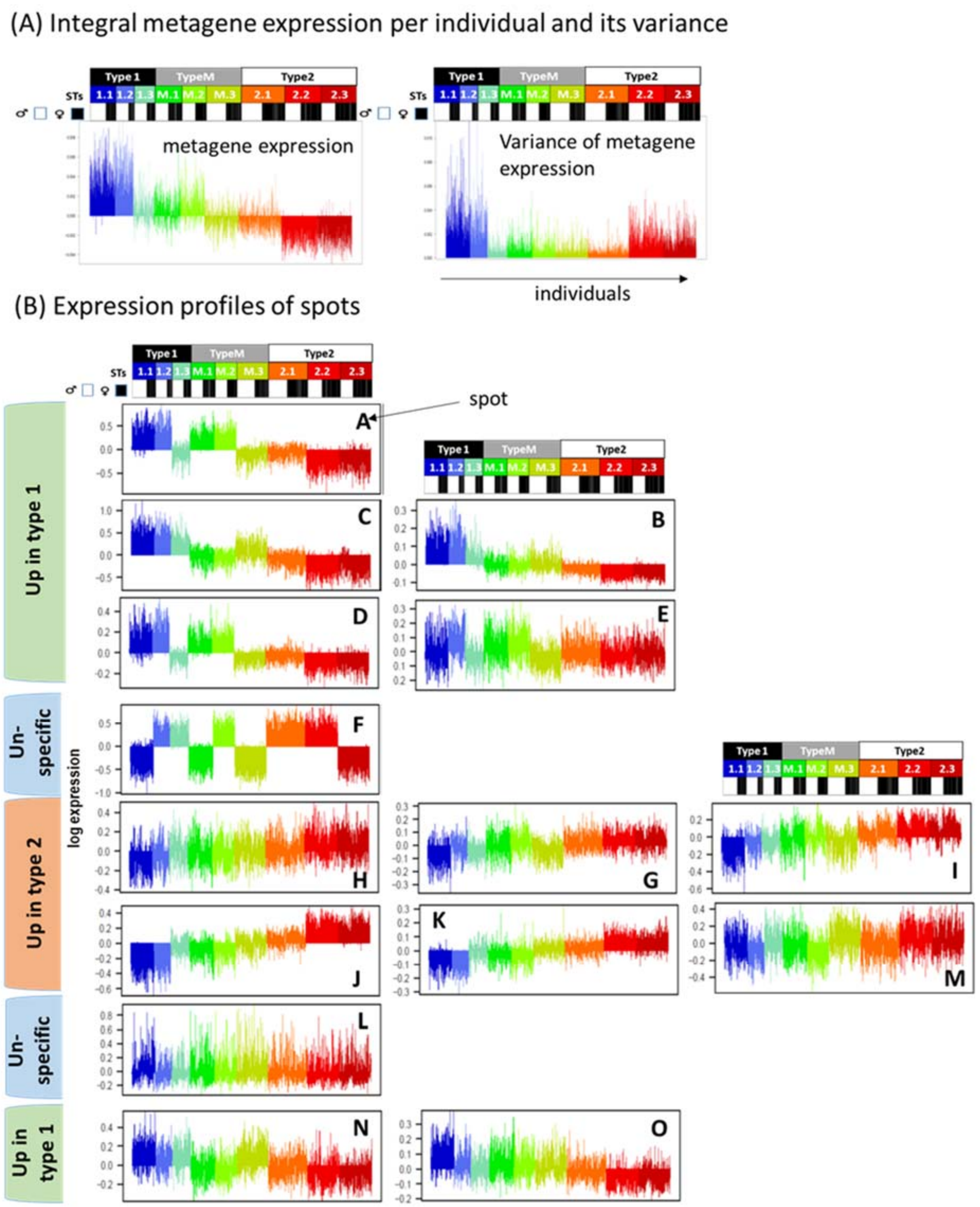

Figure S 4: Expression profiles: (A) Integral expression of all metagenes per samples and its variance. Asymmetry of metagene expression between type 1 (upregulated) and type 2 (downregulated) samples reflects larger net expression changes in type 1 compared with type 2. (B) Expression profiles of spots A –O detected in the transcriptomic landscape of the blood. The spots roughly group into profiles mainly upregulated in type 1 (7 spots, see colored marks at the left), in type 2 (6 spots) and two spots showing no clear preference for one of the types. Hereby spot F (enriching function 'transcription') reveals strong differential regulation between the subtypes (STs), while spot L (IFN-response) shows spiked upregulation of samples roughly equally distributed over the subtypes. Gene lists for each of the spots are provided as supplementary file 3.

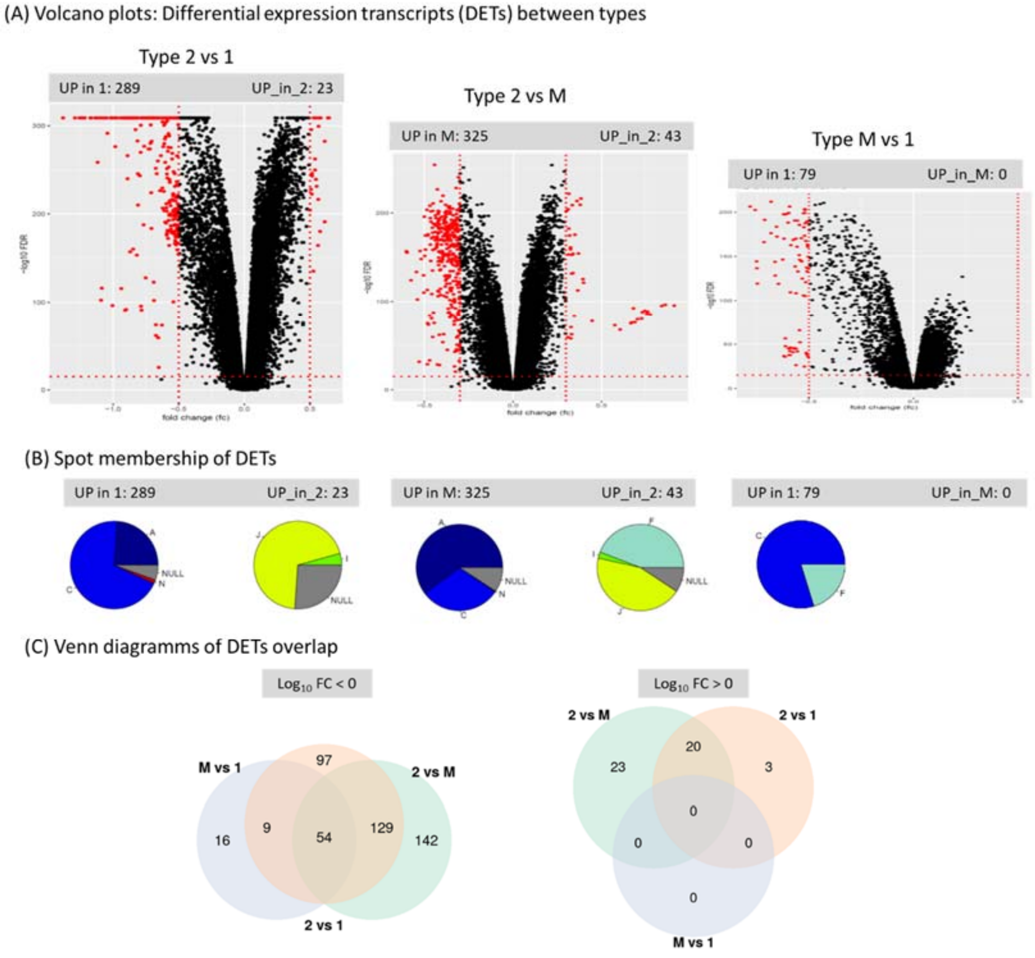

Figure S 5: Differential expression analysis between transcriptome types. (A) Volcano plots show false discovery rates (FDR) as a function of logarithmic fold changes ($\log_{10}$fc) for pairwise type-comparisons. Red dots mark transcripts meeting the condition $|\log_{10}fc| > 0.5$ *AND* $\log_{10}FDR < -16$. FDR was obtained using adjusted t-test p-values and fdr-tool {Strimmer, 2008 #1067}. One finds an asymmetrical differential expression patterns: The number of type 1_up (and type M_up) genes exceeds the number of type 2_up roughly by a number of ten. It shows that upregulation of type 1 overall accompanies by larger expression changes. (B) The pie-charts indicate the spot-membership of 'red' transcripts ('NULL' means no spot-membership). Type 1_up (and type M_up) genes are mainly members of spots A (inflammation) and spot C (erythrocytes) while type 2_up genes are mainly members of spot J (MYC-targets, translation) and, to a less degree, spot I. (C) Overlap between differently regulated genes. Type 1_up genes relative to type 2 and M overlap to about 50%.

Importantly type 1_up (and type M_up) transcriptome refers mainly to epigenetically de-repressed genes while the type 2_up transcriptome includes preferentially genes of active epigenetic states. Hence, the asymmetry of gene numbers means that a relative low number of genes from active chromatin states (e.g., MYC-targets) in type 2 are replaced by wide upregulation of de-repressed genes in type 1 transcriptomes reflecting an asymmetrical interplay between transcription factor and epigenetic networks.

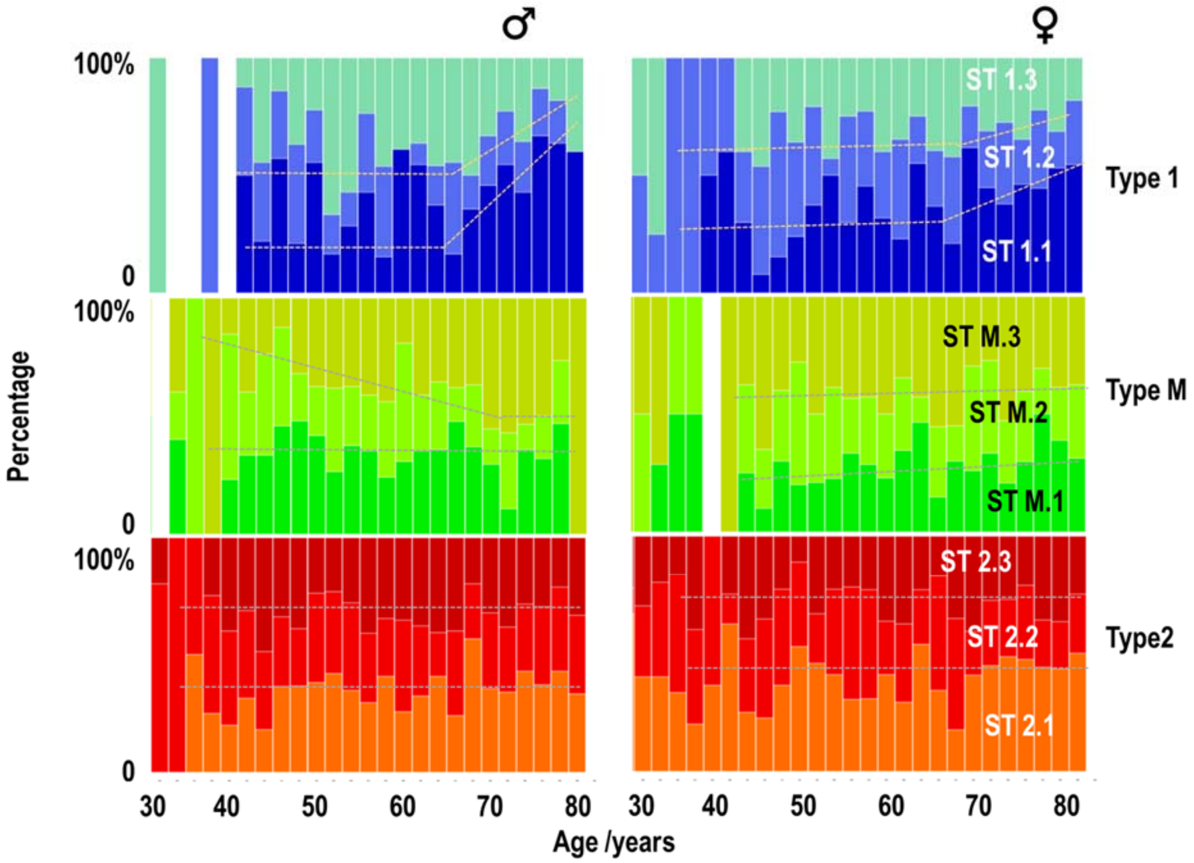

Figure S 6. Percentages of samples in subtypes (STs) in each of the transcriptome types as a function of age of the participants. The percentage of ST 1.1 in type 1 increases among men and women older than 65 years while percentage of 1.3 decreases. ST1.1 collects inflammatory phenotypes. The percentage of M.3 increases with age for men but not for women. The ST- composition of type 2 is virtually independent of age. The dotted lines serve as guide for the eyes to visualized the ageing trend.

3.3. Functional characteristics

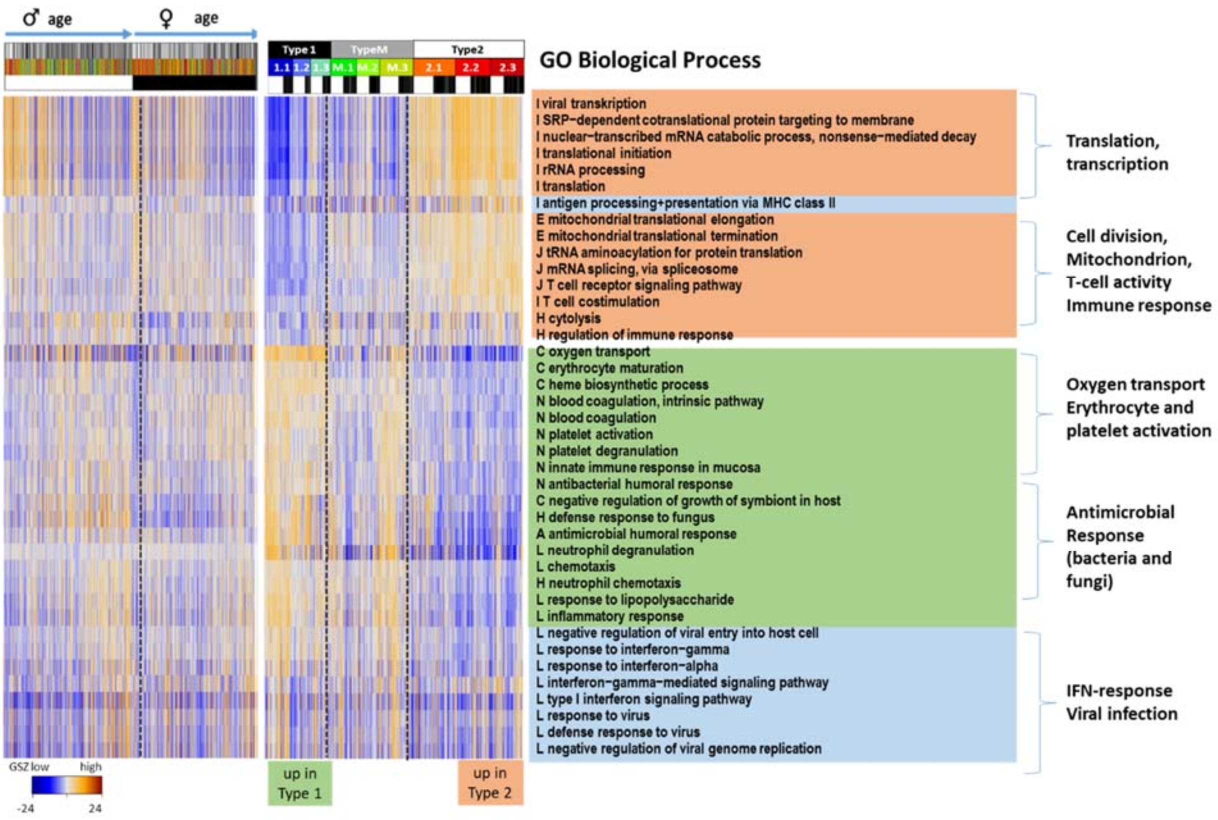

Figure S 7**:** Functional analysis using gene sets of GO biological process: The heatmap virtually divides into three clusters of genes sets upregulated predominantly either in type 1 (green) or in type 2 (apricot color) samples or in samples of all types (blue). The samples are ordered either with increasing age separately for men and women (left part) or according to their type, and ST memberships as indicated by the sample codes above the heatmaps. Type 2 transcriptomes tend to be upregulated in younger participants while type 1 upregulate predominantly in elderly participants.

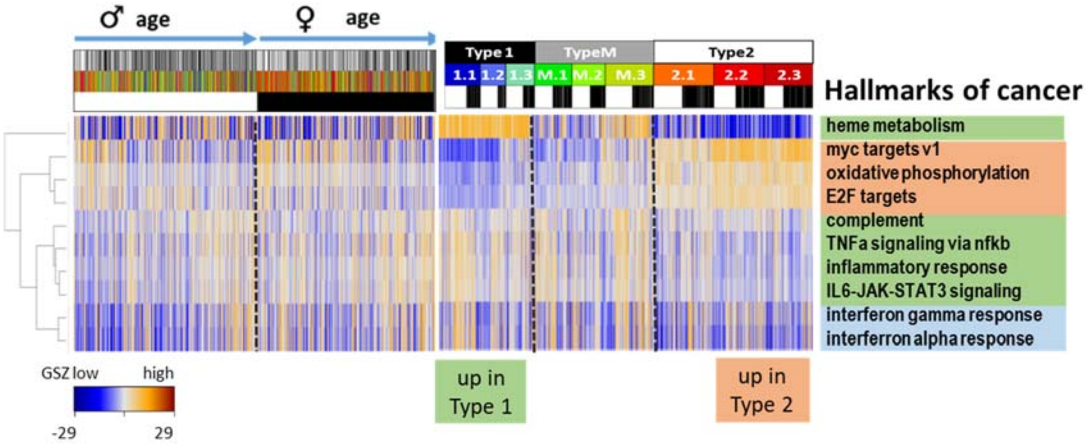

Figure S 8: Functional analysis using gene sets ‘hallmarks of cancer’ (10). See legend of Figure S 7 for details. Note that the ‘hallmarks’ describe general functional aspects with disease-related impact beyond cancer.

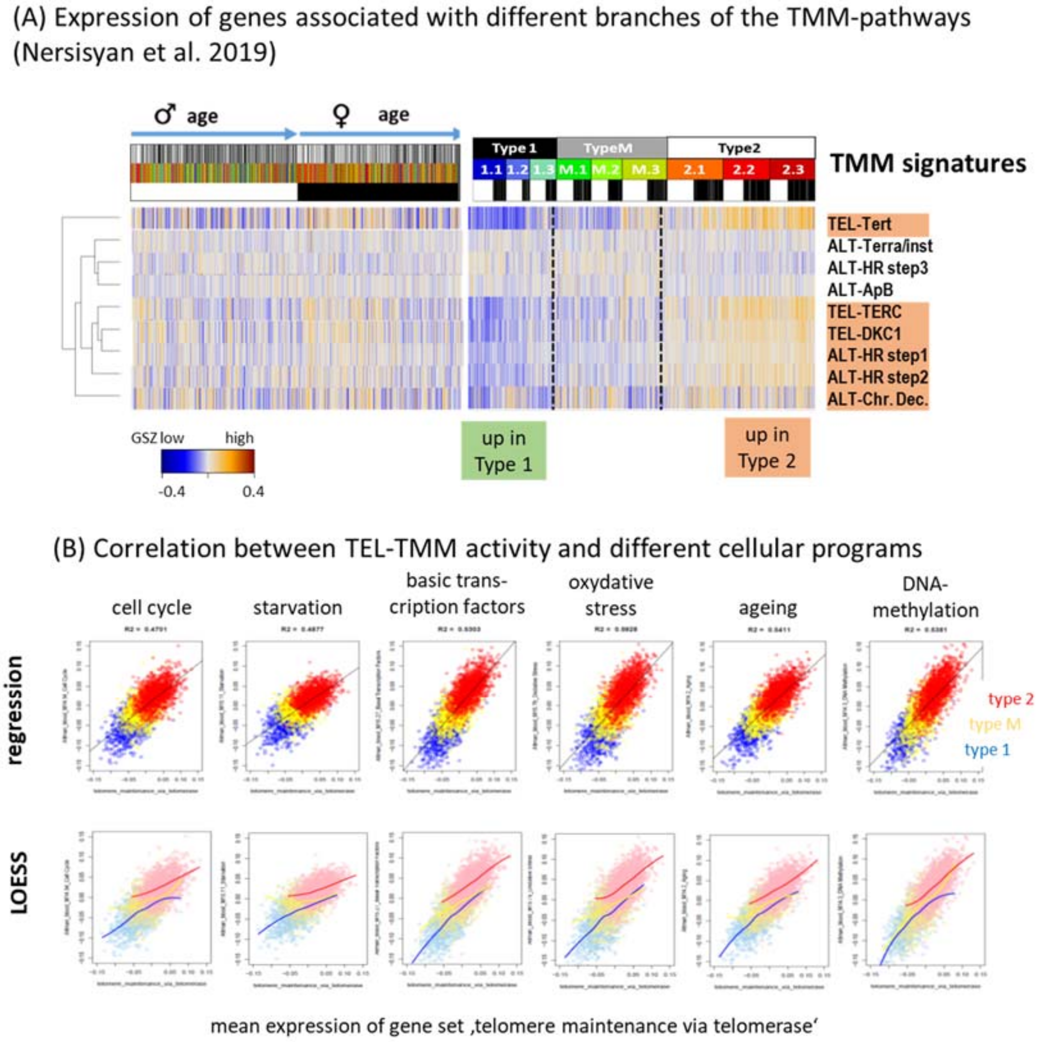

Figure S 9: Telomere length maintenance (TMM) in the blood transcriptome: (A) TMM-pathway signatures taken from (11) estimate expression level of different branches of the TEL(omerase)- and ALT(ernative)-TMM pathways. TEL-TMM strongly change with the transcriptome types while ALT-TMM remains virtually invariant. (B) Correlation plots of mean expression of the gene set ‘GO telomere maintenance via telomerase’ and the mean expression of gene sets referring to different cellular processes. The regression coefficients change between r=0. 47 and 0.59. The colours assign samples of type 1, M and 2. Separate LOESS fits were performed independently for the three transcriptome types. Overall, TEL-activity linearly correlates with the processes considered where type 2 transcriptomes show largest expression.

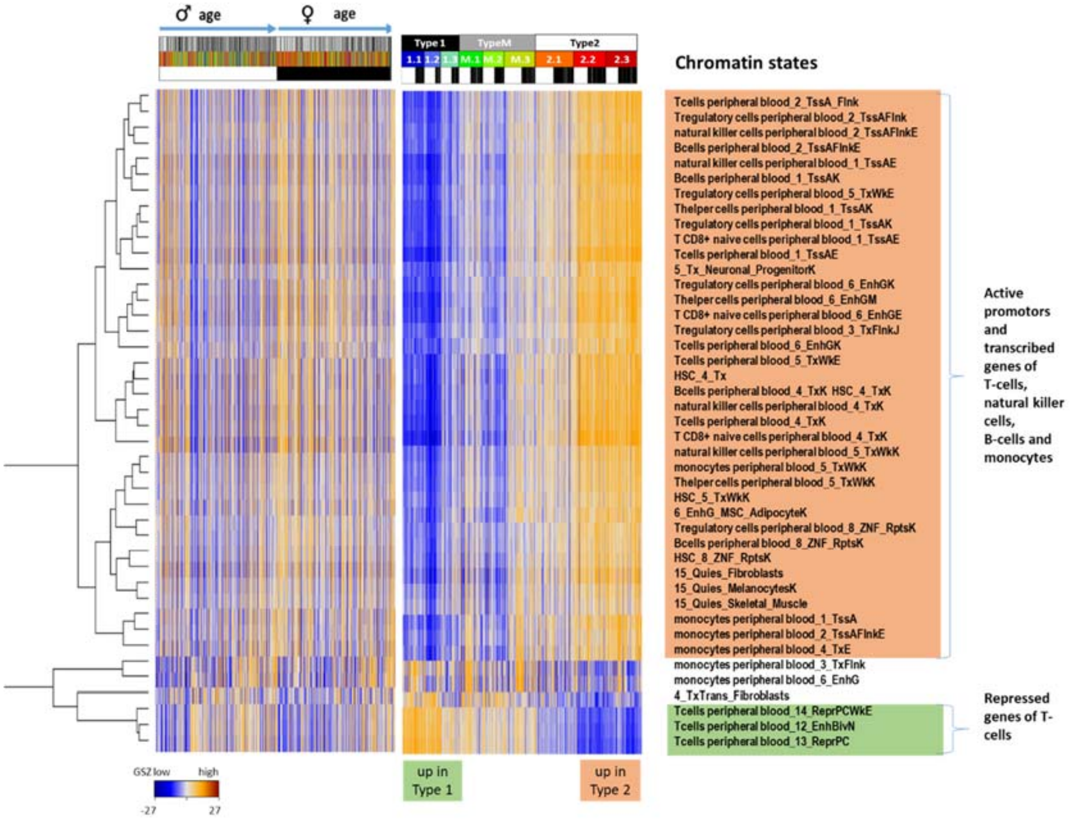

Figure S 10: Gene signatures of a series of chromatin states in different blood cell types (T-cells, T-regulatory cells, natural killer cells, B-cells) and fibroblasts taken from (12) mapped onto transcriptome types. Genes in 'active promoters' (TSSA) and 'transcribed (Txn) states show high expression in type 2 samples while repressed (ReprPC) in T cells upregulate in type 1 transcriptomes. High expression of repressed states suggests their de-repression in type 1 samples by chromatin remodeling. Repression acts via polycomb repressive complex 2 (PRC2) and H3K27me3 (triple methylated lysine side chains at position 27 of histone 3 compound of nucleosomes).

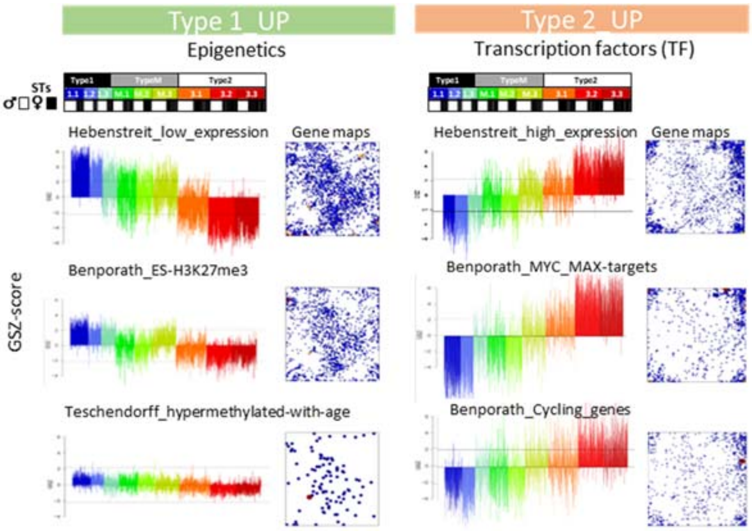

Figure S 11: Gene sets related to the activity of transcription factors (TF) and states driven by epigenetic mechanism such as specific histone modifications . Their expression profiles (and gene maps) support the notion that the former one's associate predominantly with activation in type 1 and the second one with activation in type 2 transcriptomes. Notably, epigenetically related genes accumulate in the central area of the map which collects genes of medium and low expression levels. Gene sets were taken from (13-15).

3.4. Previous signatures of the blood transcriptome and blood cell deconvolution

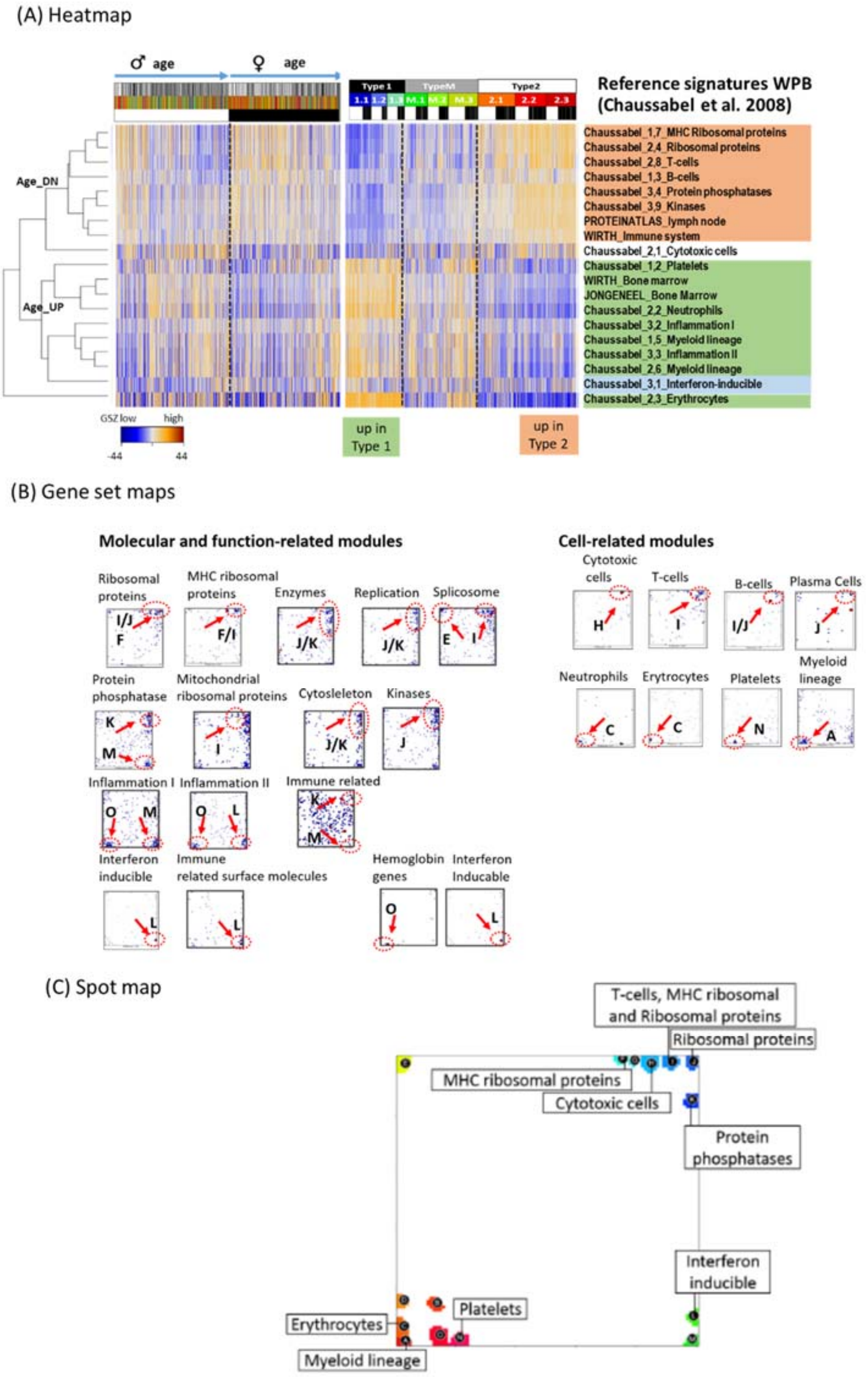

Figure S 12: Functional analysis using gene sets 'whole periphal blood signatures' (16). (A) Heatmap of the GSZ-scores of the gene setsSee legend of Figure S 7 for assignments (part A). (B) Gene sets maps visualize the positions of the member genes of the set in the SOM. Most of them accumulate in or near the spots identified in the expression maps (see red dashed elipses and the letters for spot assignment). (C) The spot map summarizes the functional assignments of the spots using these blood transcriptome signatures.

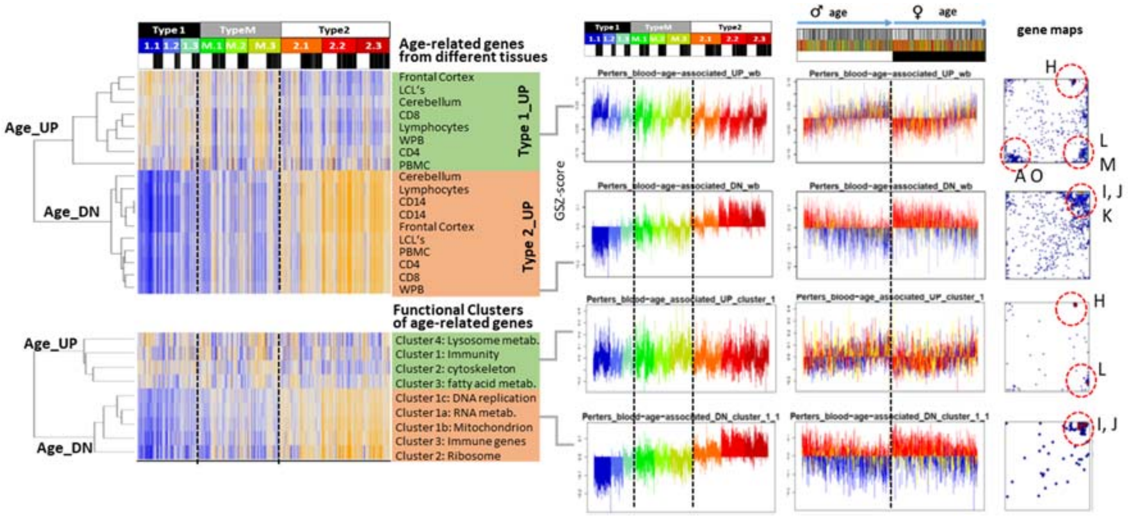

Figure S 13: Functional analysis using age-related gene sets based on a meta-analysis of whole-blood gene expression data (17). The heatmaps show gene sets referring to different tissues/cell compounds used for comparison (upper part) and to different functional gene clusters identified in the WPB-transcriptome (part below). Expression profiles were shown for selected sets which either up- or down-regulate with age (right part). The samples were sorted either according to their type and ST memberships or with increasing age. Overall the profiles divide into two major clusters either upregulated in type 1 or type 2 blood transcriptomes, where however 'type 2_UP' profiles more clearly express differences between the types than 'type 1_UP' profiles. These characteristics of the profiles associate with localized accumulation of the genes in the right upper corner of the map ('type 2_UP', spots I, J and K) while genes of the 'type 1_UP' sets widely distribute among different spot areas. In other words, the typing of the blood transcriptome is asymmetrical with respect to genes whose expression decays or increases with age. Genes of decaying expression upon ageing map predominantly into a few spot modules of coexpressed genes thus reflecting a virtually parallel relationship between ageing and the overall covariance structure of the transcriptome. In contrast, genes showing increasing expression upon ageing distribute over a series of spots with diverse expression type-characteristics which reflect a mutually multidimensional relationship presumably indicating a multifactoral causality.

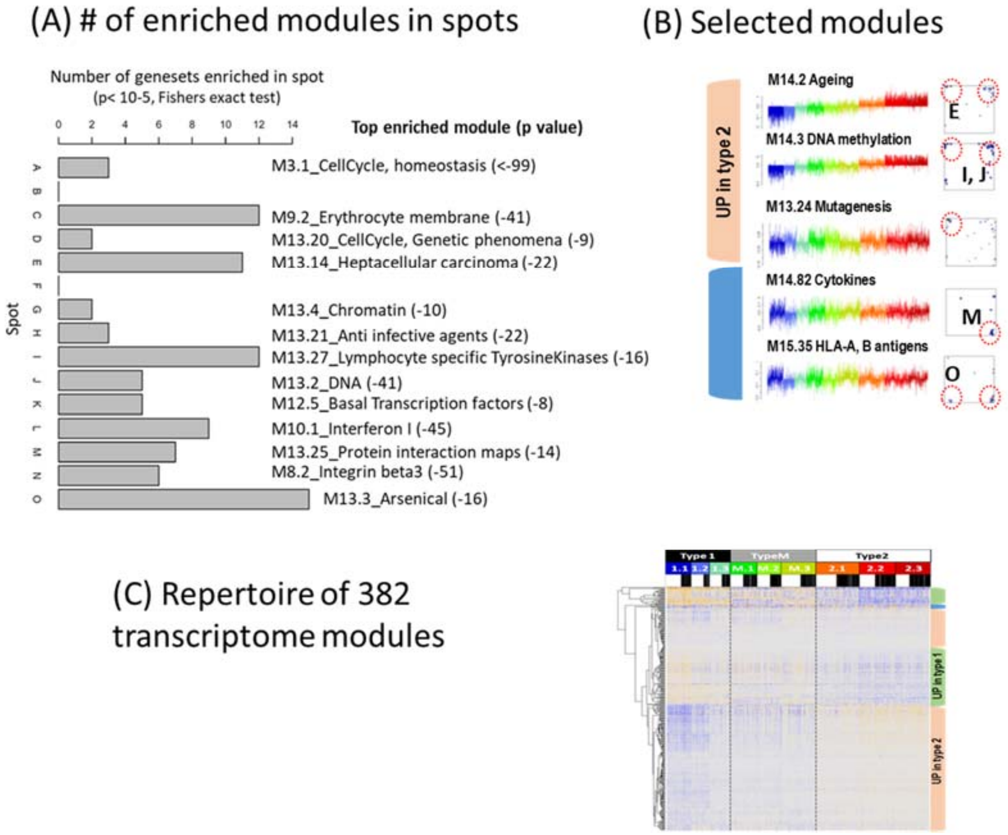

Figure S 14: Transcriptome analysis using a repertoire of 382 functionally annotated transcriptional modules (18) reveals agreement with our typing scheme: (A) Number of modules showing enrichment in the spots (with p< 10^{-5}, Fishers exact test). Spots O (MAPK signaling), C (erythrocytes) and I (T-cells) accumulate 12 or more modules, which reflects their association with a multitude of functions. Overall 91 modules were found enriched in at least one of the spots. (B) Profiles and gene maps of modules showing enrichment in spots E or M. Modules related to DNA-methylation or ageing show virtually identical characteristics. Spot M associates with HLA- antigens and cytokines. (C) Clustering of all 382 modules reveals a larger number of modules upregulated in type 2 than in type 1.

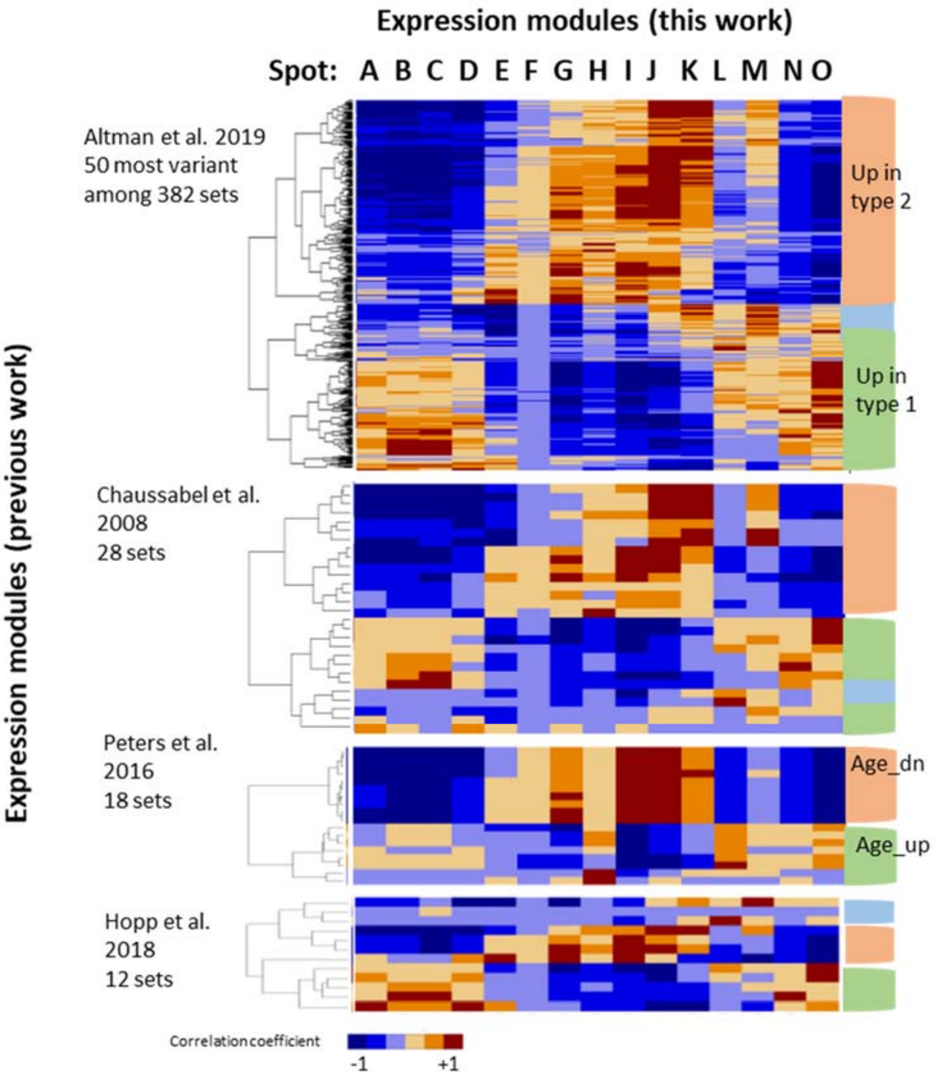

Figure S 15: Correlation between previous blood transcriptome signatures (16-19) and the spot profiles identified in the blood transcriptome in this work. All collections of signatures split into very similar clusters of spot-patterns (compare the brown and blue areas in the heatmaps) reflecting marked correlations and anti-correlations referring to type-specific up- and down-regulation and also spot-specific subclusters. This common co-variance structure reflects the intrinsic 'eigen'-modes of co-regulated genes in the blood transcriptome as extracted by the spots. It is also evident in other categories of signatures such as that for blood cells (see Figure S 16C). Profiles are assigned in Figure S 12 (16), Figure S 13 (18) and Figure S 14.

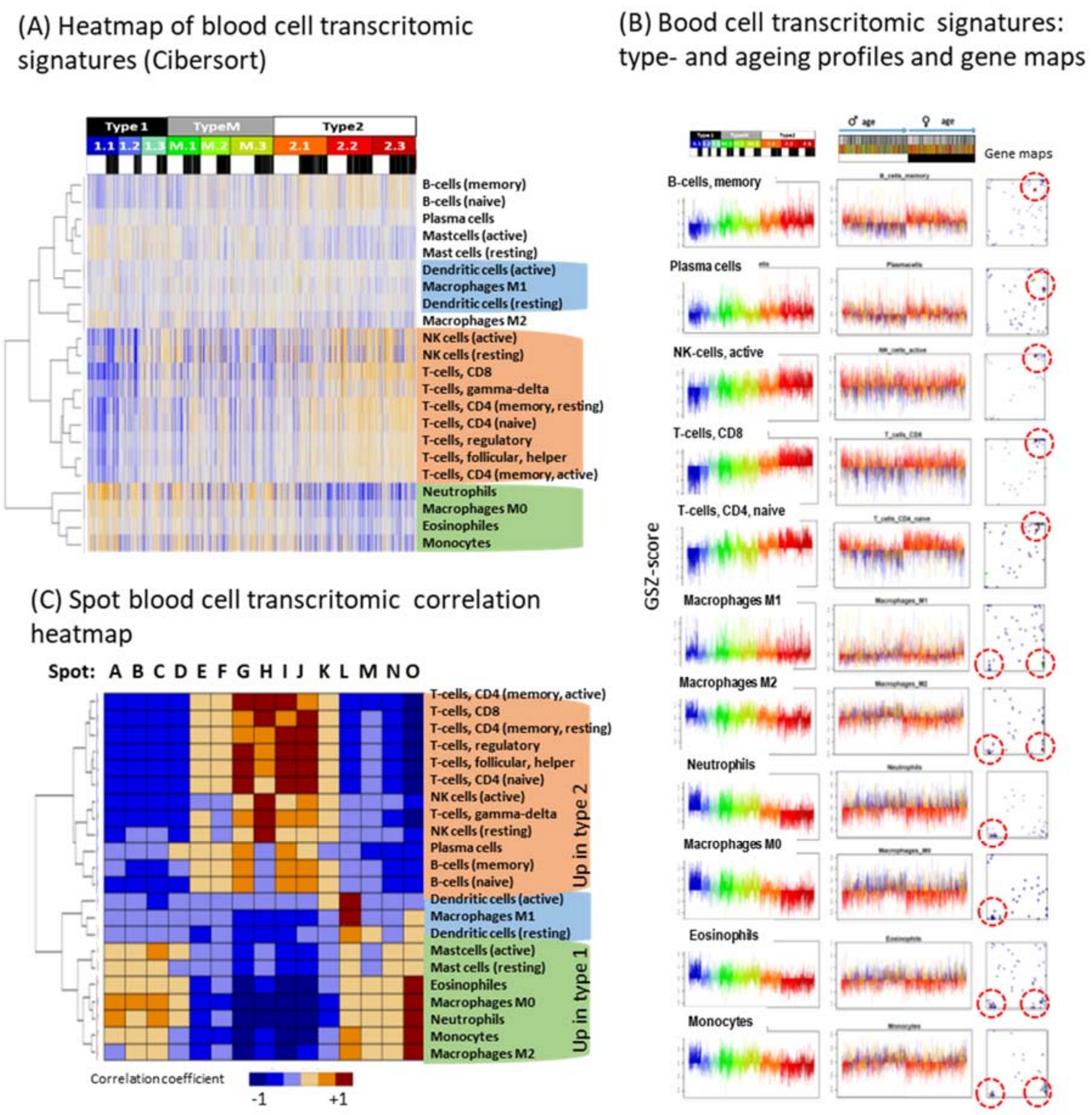

Figure S 16: Expression signatures of 22 types of blood cells taken from Cibersort (20). The GSZ-heatmap (A) and the profiles of selected cell types (B) reveals type and subtype specific upregulation in the blood transcriptome. Notably, ST1.1 and M.1 expression associates with neutrophils, eosinophils and monocytes (recall that ST1.3 and M.3 associate with erythroid cells and thrombocytes, see above). The gene maps reveal accumulation of the signature genes in distinct spot areas as indicated by red circles. (C) The correlation heatmap between the spot and signature profiles relates spot expression to the cell types. It clearly shows a type-specific spot expression patterns either upregulated in type 1 or 2. In addition one sees a cell type specific fine structure of spot expression. Expression of dendritic cells and macrophages M1 associate with spot L and thus with the IFN-response related to viral infections.

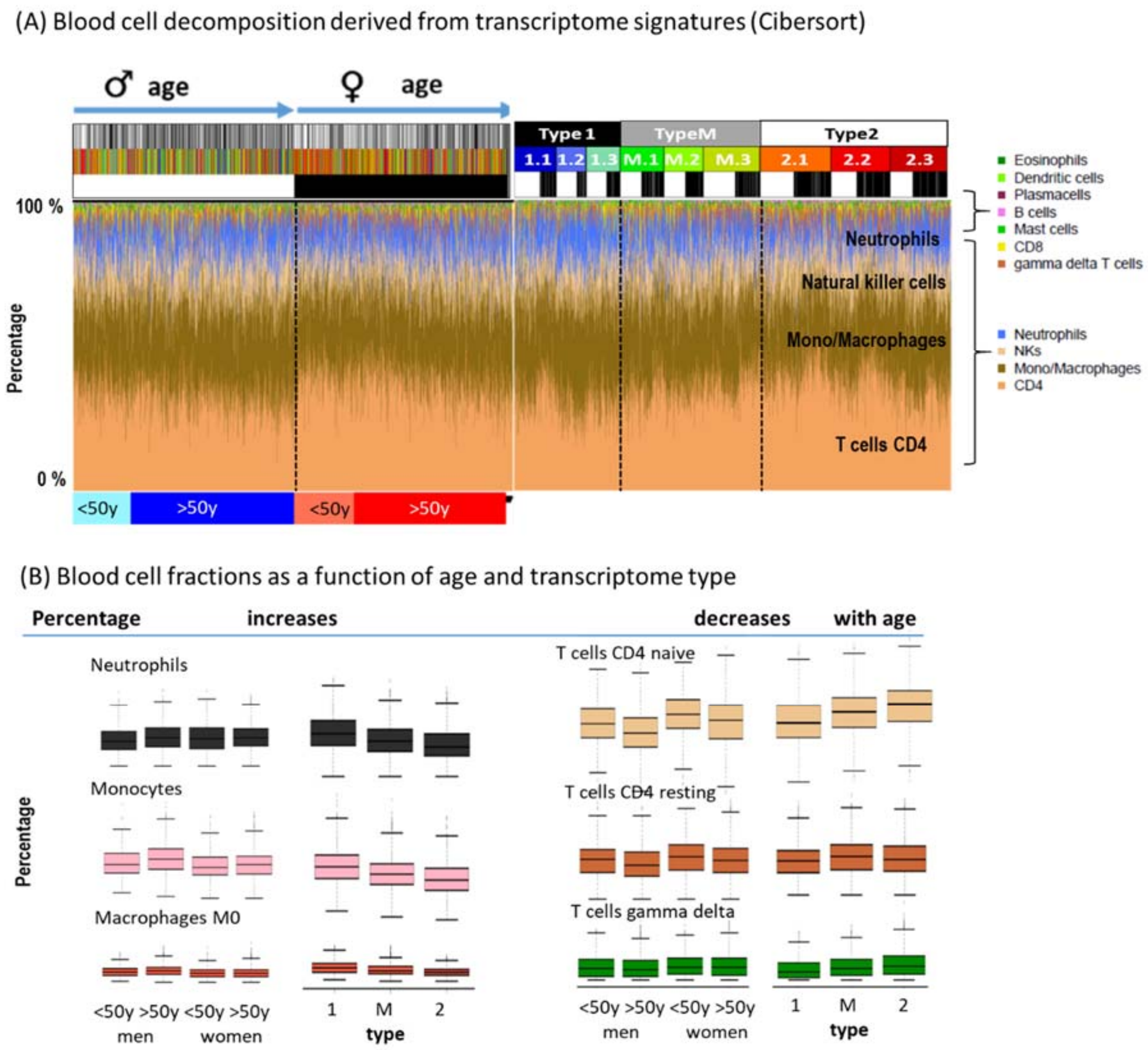

Figure S 17: Relative cell counts (in units of percentage) of the 22 cell types considered in Cibersort (20). Most abundant cell types are CD4 T-cells, monocytes/macrophages, natural killer cells and neutrophils. Relative cell counts change between subtypes in parallel to the respective expression levels (Figure S 16 A and B).

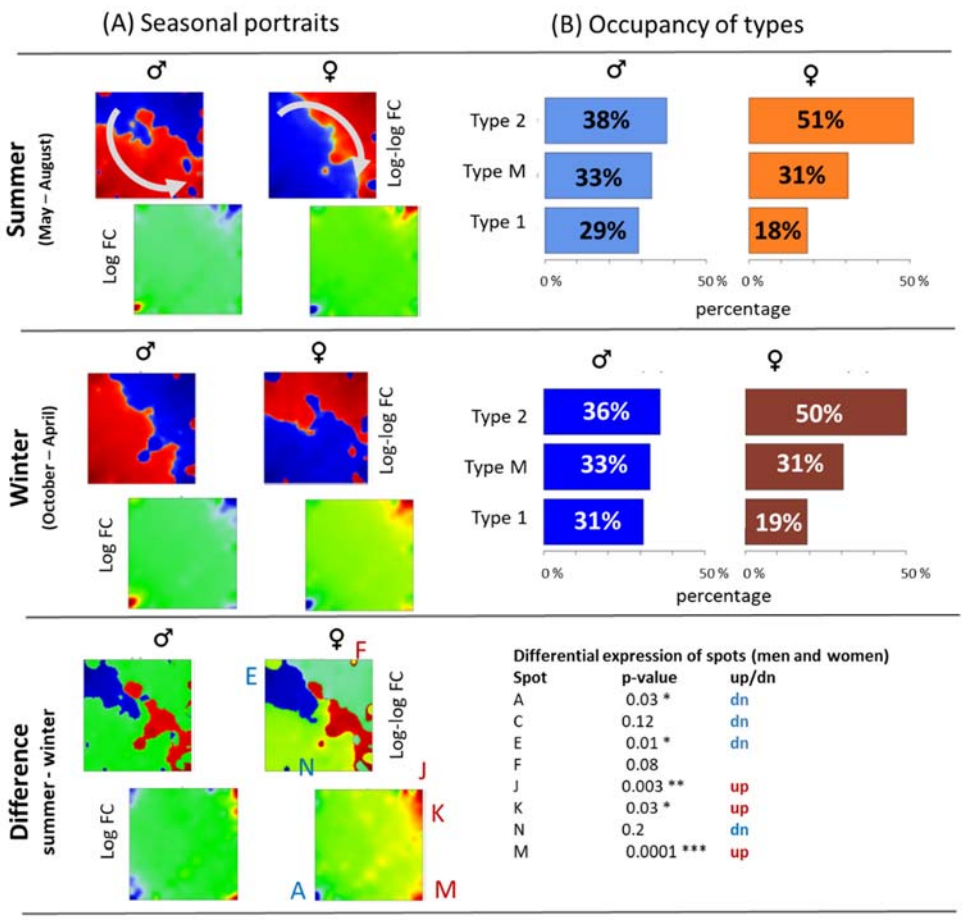

Figure S 18: Seasonal (summer versus winter) effects on the transcriptomes of women and men: (A) Seasonal portraits (in log FC and log-log FC scales) are calculated as mean portraits averaged over all individuals whose blood samples were collected between May and August (summer) and October and April (winter), respectively. The portraits show the sex-specifics of the transcriptomes. The log-log FC scale better resolves subtle seasonal changes as indicated by the arrows, which can be rationalyzed by the gender-specifics of the transcriptomes and a virtually very similar seasonal effect on women's and men's transcriptomes. The difference portraits indicate higher levels of type 2 (especially spots J, K, and M) in summer and increased levels of type 1 (especially of spots A, C, N and E) in winter, respectively (see t-test p-values of differential spot expression). These changes associate with the alteration of blood counts of reticulocytes, basophils and erythrocytes (Table S 5). (B) Distribution of blood transcriptomes over types only weakly change by 1- 3 percent between summer and winter.

3.5. Phenotype characteristics and associations with the blood transcriptome

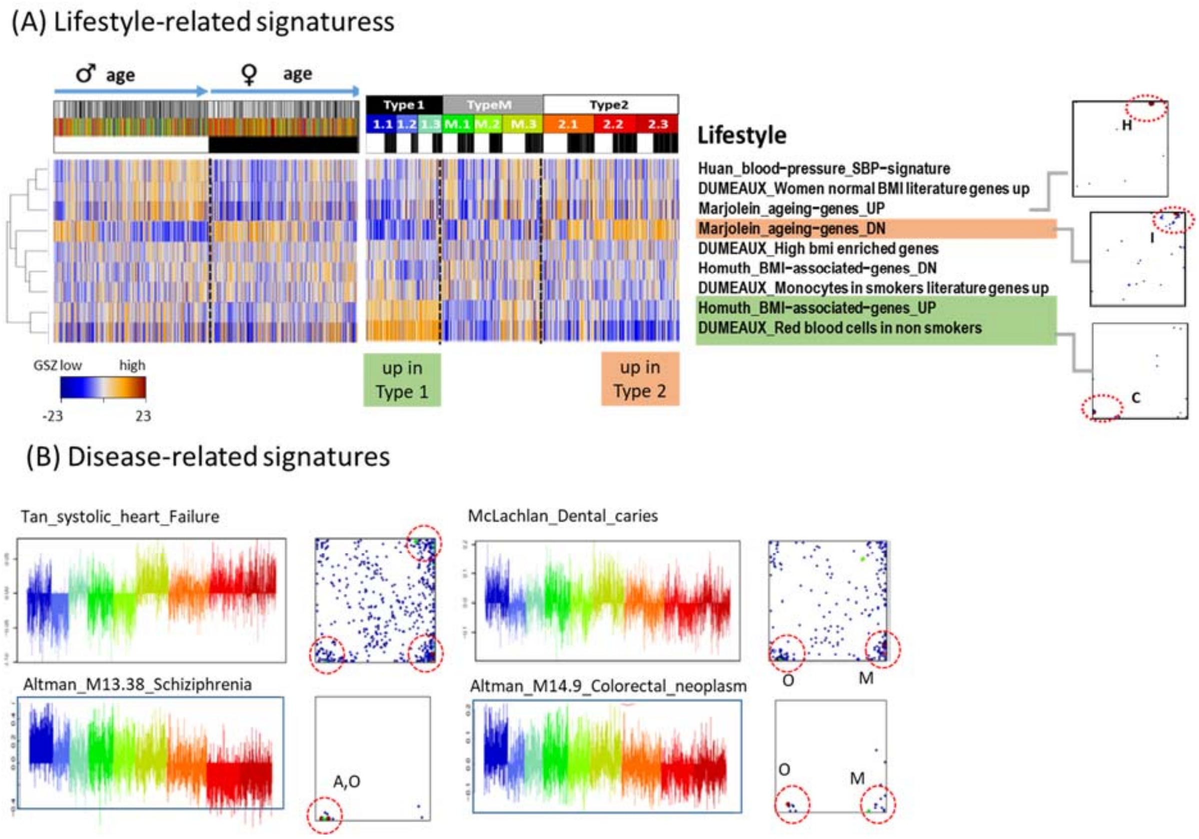

Figure S 19: Lifestyle-, age- and disease-related signatures: (17, 21-23) (18, 24, 25) reveal profiles and gene maps resembling inflammatory and/or blood-disturbant signatures.

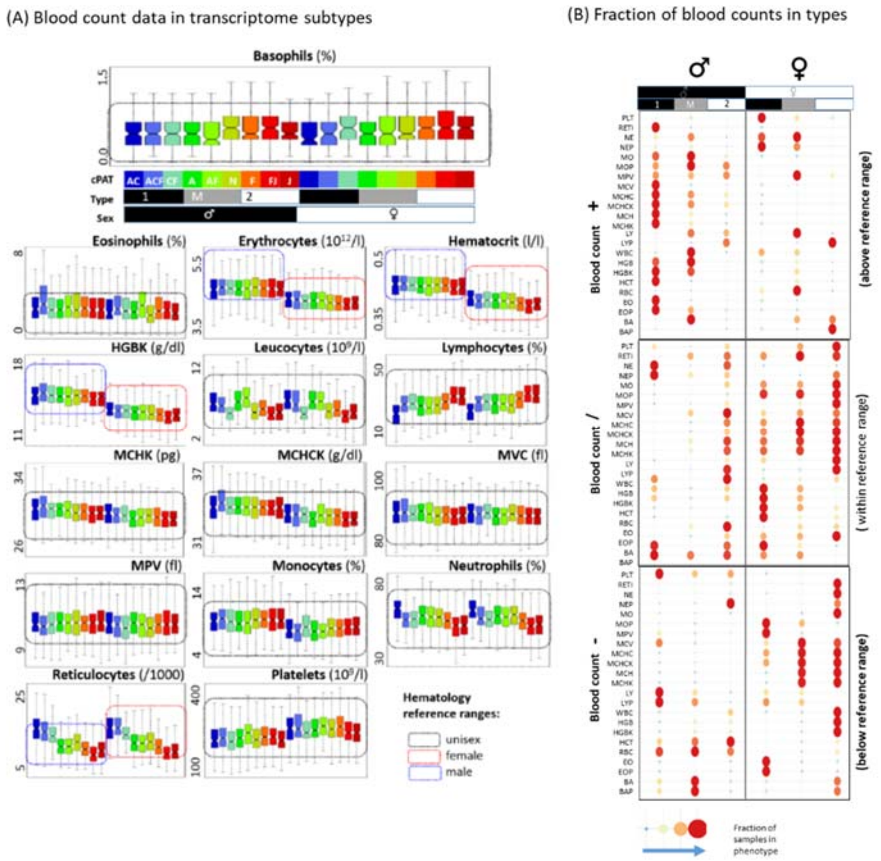

Figure S 20: Blood count data of the LIFE-transcriptome cohort stratified for gender and transcriptome subtypes/types. (A) Boxplots of blood count levels as a function of subtypes. See the larger plot above for assignments. Reference ranges were shown as frames in each of the plots where the upper and lower edges limit the respective range. Erythrocytes and hematocrit levels are different for men and women as considered by gender-specific reference ranges. Reticulocyte-counts strongly vary between the subtypes being maximum in ST 1.1 and 1.2 and minimum in ST 2.2 and 2.3 for men and women as well. Lymphocytes show the reverse relation with maximum counts in ST 2.2 and 2.3. Leucocytes, in turn, reveal large count levels in M.1 and M.2. (B) Enrichment of blood counts above, within and below the respective reference ranges among the samples of the different transcriptome types. Blood counts within the reference ranges are typical for type 2 transcriptomes of men and transcriptomes of all types for women. Blood counts above the reference range are found preferentially in type 1 (and type M) men while blood counts below the reference range enrich in type 2 transcriptomes of women. Hence, overall one finds a shift from type 1 to type 2 transcriptomes for men with enhanced blood counts and women with decreased blood counts. These differences reflect a sexual dimorphism also evident in transcriptomic data.

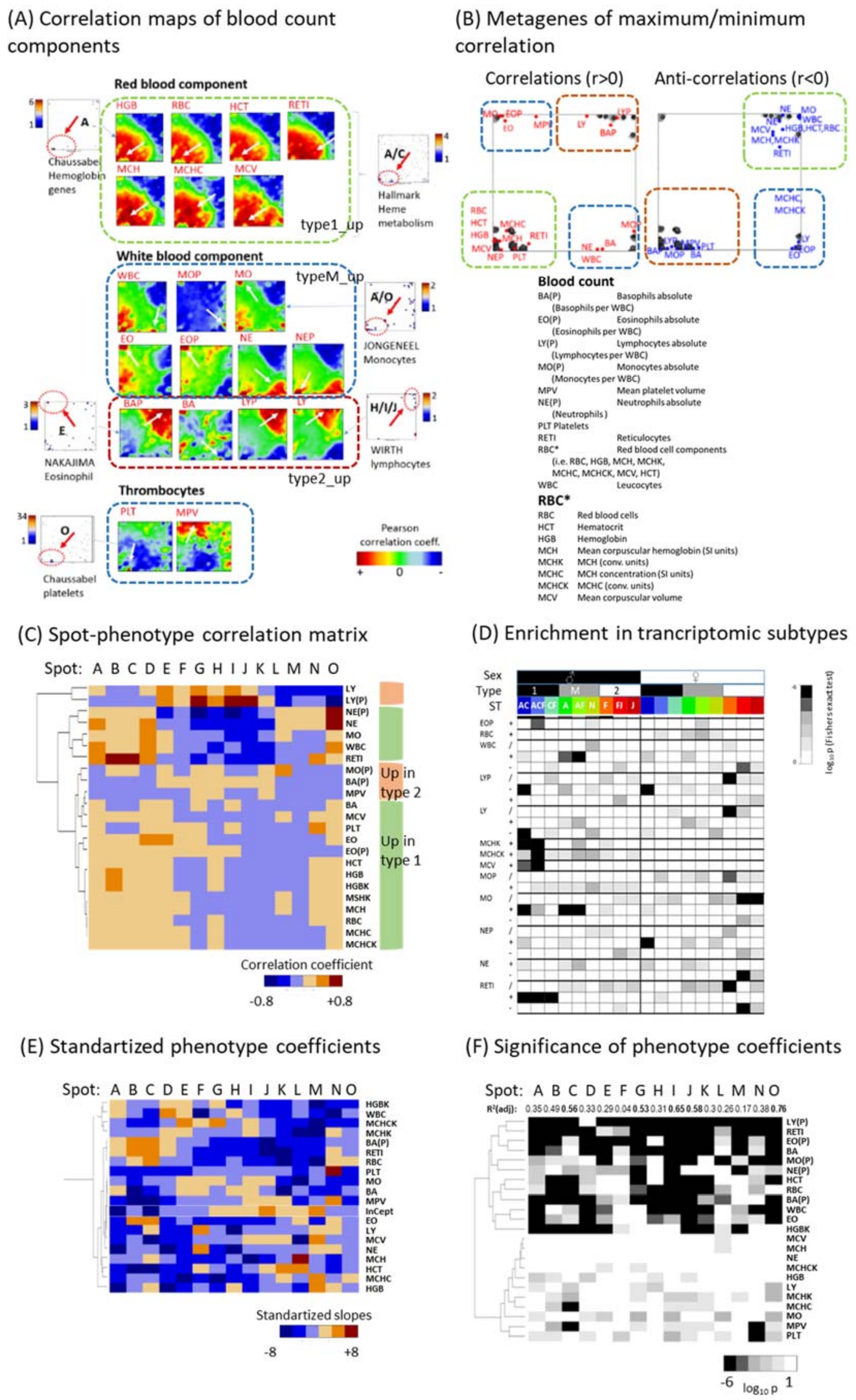
(A) Correlation maps of blood count components
Red blood component
HGB
RBC
HCT
RETI
MCH
MCHC
MCV
type1_up
Chaussabel Hemoglobin genes
Hallmark Heme metabolism
White blood component
WBC
MOP
MO
typeM_up
EO
EOP
NE
NEP
JONGENEEL Monocytes
BAP
BA
LYP
LY
NAKAJIMA Eosinophil
WIRTH lymphocytes
type2_up
Thrombocytes
PLT
MPV
Chaussabel platelets
Pearson correlation coeff.
(B) Metagenes of maximum/minimum correlation
Correlations (r>0)
Anti-correlations (r<0)
Blood count
BA(P) Basophils absolute (Basophils per WBC)
EO(P) Eosinophils absolute (Eosinophils per WBC)
LY(P) Lymphocytes absolute (Lymphocytes per WBC)
MO(P) Monocytes absolute (Monocytes per WBC)
MPV Mean platelet volume
NE(P) Neutrophils absolute (Neutrophils)
PLT Platelets
RETI Reticulocytes
RBC* Red blood cell components (i.e. RBC, HGB, MCH, MCHK, MCHC, MCHCK, MCV, HCT)
WBC Leucocytes
RBC*
RBC Red blood cells
HCT Hematocrit
HGB Hemoglobin
MCH Mean corpuscular hemoglobin (SI units)
MCHK MCH (conv. units)
MCHC MCH concentration (SI units)
MCHCK MCHC (conv. units)
MCV Mean corpuscular volume
(C) Spot-phenotype correlation matrix
Spot: A B C D E F G H I J K L M N O
Up in type 2
Up in type 1
Correlation coefficient
-0.8
+0.8
(D) Enrichment in trancriptomic subtypes
Sex
Type
ST
log10 p (Fishers exact test)
(E) Standartized phenotype coefficients
Spot: A B C D E F G H I J K L M N O
Standartized slopes
-8
+8
(F) Significance of phenotype coefficients
Spot: A B C D E F G H I J K L M N O
R²(adj): 0.35 0.49 0.56 0.33 0.29 0.04 0.53 0.31 0.65 0.58 0.3 0.26 0.17 0.38 0.76
-6
log10 p
1

Figure S 21: Association of blood count data with the blood transcriptome. (A) Correlation maps indicate correlations between blood count components with the transcriptome landscape. Red blood components associate predominantly with type 1 transcriptomes, while white blood components and platelets show more diverse association patterns. Lymphocytes and neutrophiles associate with type 2 or type M transcriptomes as indicated by the frames. Areas of strongest correlation are indicated by white arrows in the correlation maps. They largely agree with previous gene expression signatures (see gene maps: red arrows and red circles indicate areas of accumulation of genes and Figure S 12). (B) Metagenes of maximum (positive) and minimum (negative) correlation coefficients are shown for each blood component by a dot. The patterns obtained accumulates most spots in the opposite corners of the map which assign to upregulation of type 1 and type 2 genes, respectively (see the dashed frames). Interestingly, the anti-correlations patterns of these dots more clearly agrees with type 1 and type 2 transcriptomes than the patterns of positive correlations. Note also that absolute and relative (%) units partly provide different positions for the same blood component. Absolute measures more adequately relate to expression data, due to their absolute scale. (C) Correlation coefficients between spot expression profiles and blood count profiles are shown as heatmap. Regulation of spots and blood counts is visualized in green (up in type 1) and apricot (up in type 2). (D) Enrichment of blood counts in transcriptomic subtypes for men (left part) and women (right). Counts falling into (symbol '/'), falling below ('-') or exceeding ('+') the reference range are considered separately (see Figure S 20). Accordingly, type 1 transcriptomes enrich men with reticulocyte and hemoglobin (MCHK, different units) counts exceeding the reference range. (E) Multiple linear regression of the blood count profiles of each of the spot expression profiles. The heatmap visualizes matrix of standartized slope-coefficients of each of the blood counts. (F) Significance of slope coefficients (p-value) is shown as heatmap: Reticulocytes and lymphocytes significantly ($p < 10^{-6}$, black) contribute to virtually all spots while platelets affect solely spot N. Virtually all significant blood counts locate their metagenes of maximum/minimum correlation in the corners of the map as indicated by the dashed frames in part B of the figure.

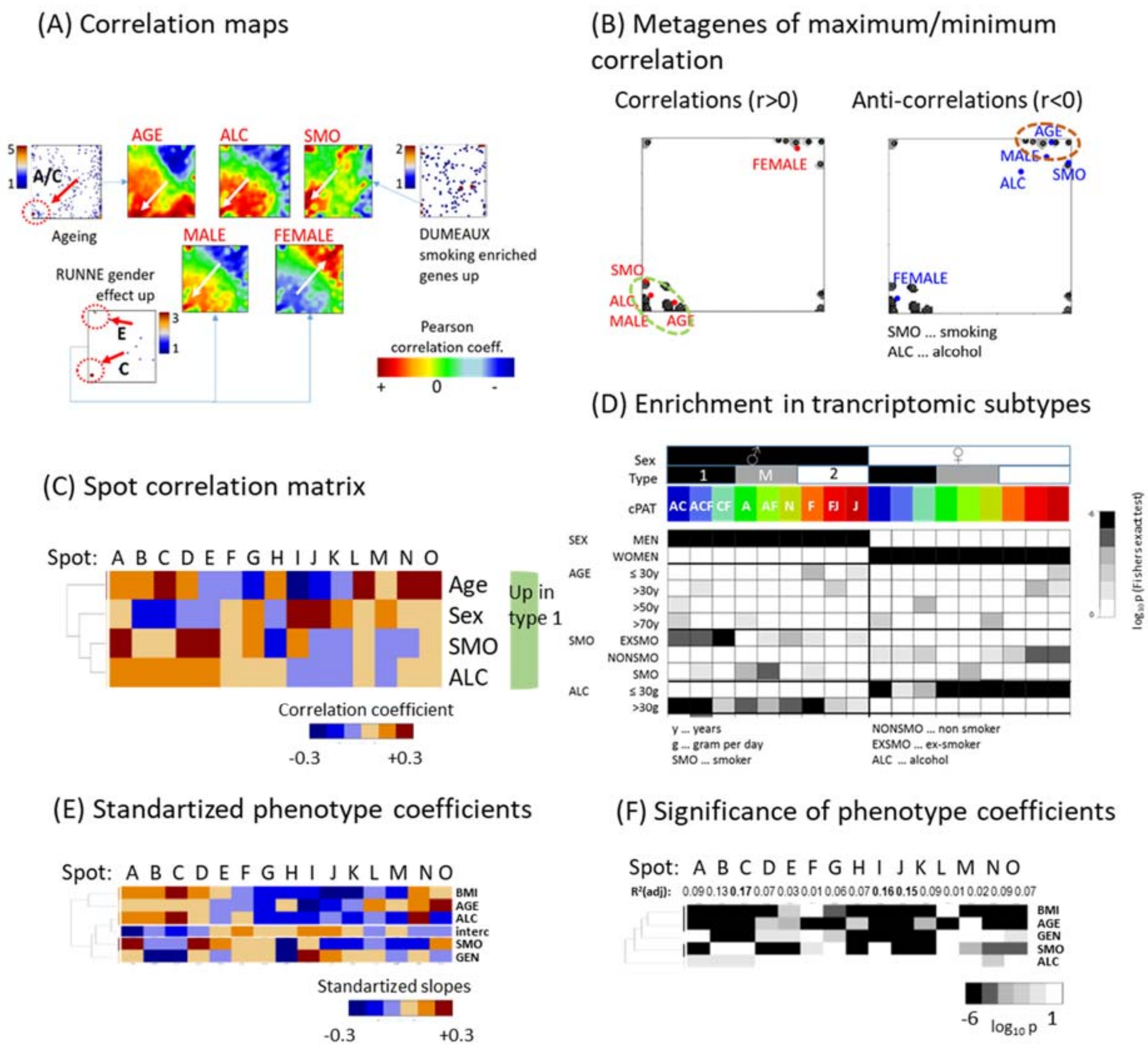

Figure S 22: Association of age, sex, alcohol consumption (more than 30 g/day) and smoking status (smokers) with the blood transcriptome. See legend of Figure S 21 and Table S 1 for assignments. The red correlation patterns in the phenotype portrait of ageing well agree with the distribution of age_up genes shown in Figure S 13. Age, smoking and alcohol consumption associate with type 1 transcriptomes, however with subtle specifics of the different phenotypes as indicated by the position of metagenes of maximum/minimum correlation (part B), by the spot-phenotype correlation patterns and regression coefficients (E). Alcohol consumption (>30 g/day) associates with men, mainly of type 1 (part D) and virtually non-drinking women mainly with type M and 2.

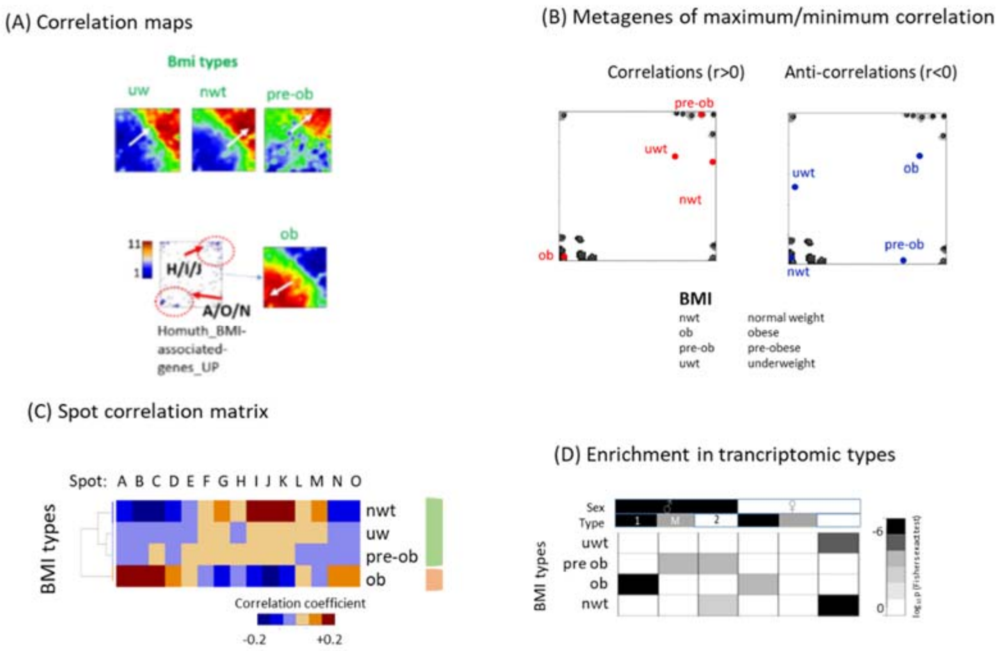

Figure S 23: Association of obesity classes and body types with the blood transcriptome. (A) Obese types associate with type 1 in agreement with a previous BMI signature (23)(see gene set map). Obese and/or most male body types (M5 and F3) associate with type 1 transcriptomes while women more associate with type 2. M1 and M2 collecting mostly younger and medium age men show correlations near spot E and with spot L (IFN response).

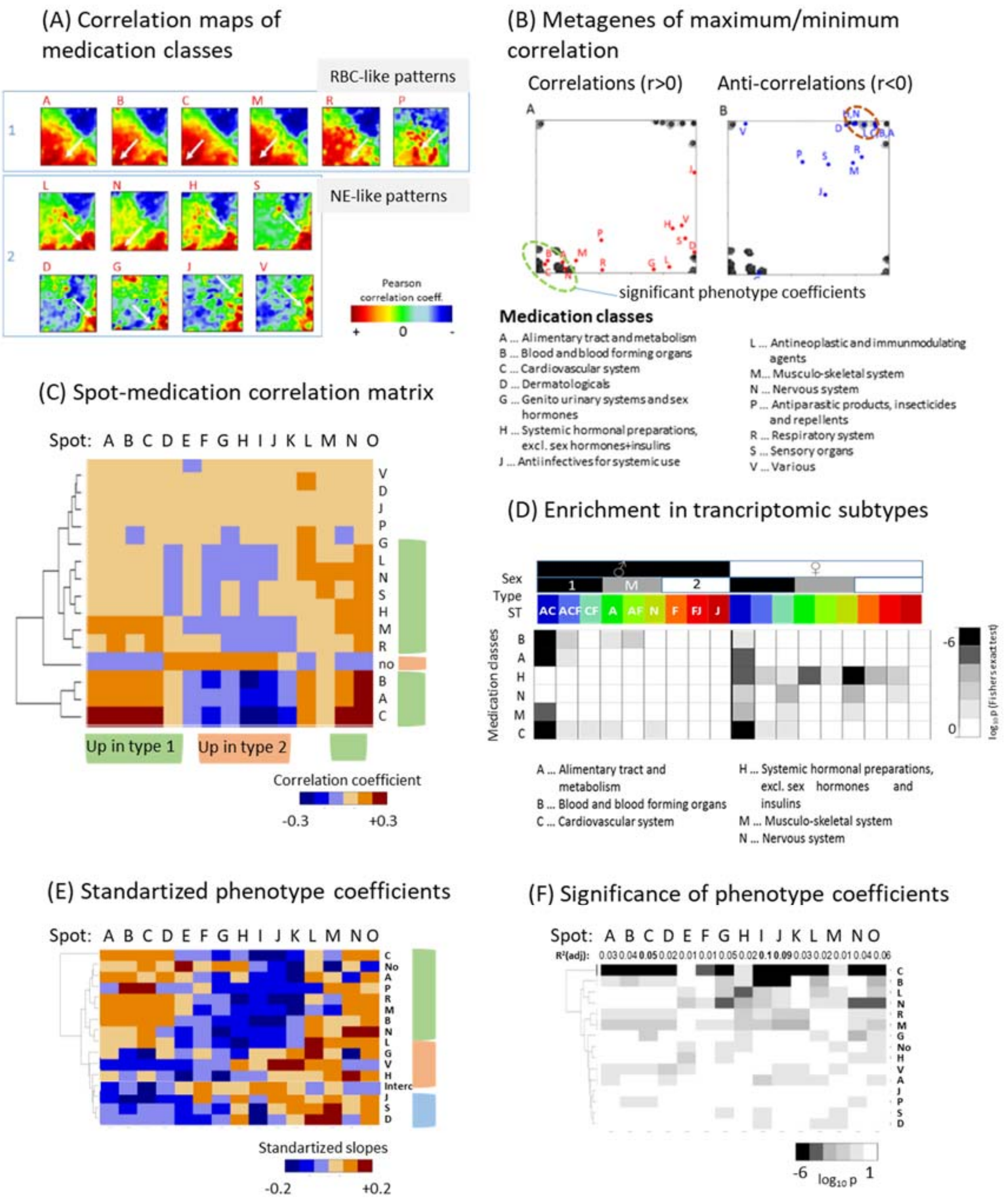

Figure S 24: Association of medication with the blood transcriptome (see Figure S 21 and Table S 1 for description and assignments). The phenotype maps resemble those of red blood components (RBC-like patterns) or those of neutrophils (NE-like patterns) or they show red areas in and around spots M, L and K along the right border of the portraits (part A). RBC- and NE-like patterns suggest association of elevated levels of these blood counts in the participants taking the respective drugs. The latter type of patterns is obviously not covered by blood count patterns. Medications (especially, C, B, L and N) with significant (log p < -6) phenotype coefficients are paralleled by maximum correlation with spots in the left lower (r >0) and right upper (r< 0) corners of the map.

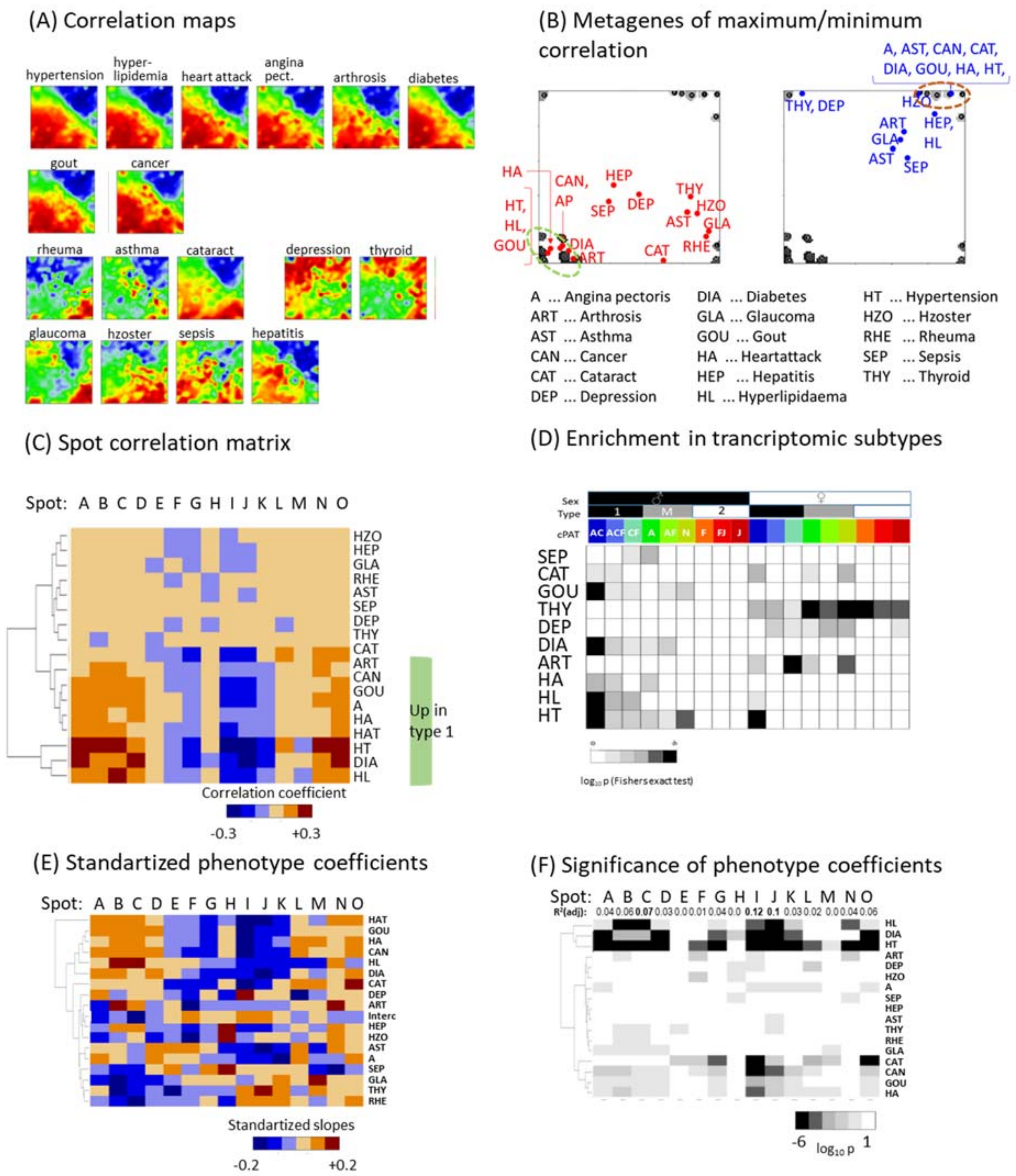

Figure S 25: Association of the disease histories with the blood transcriptome (see Figure S 21 and Table S 1 for description and assignments). As for medications, phenotype portraits of part of diseases are of the RBC- (first two rows in part A) and NE-types, while others show a more specific correlation patterns. Diseases with significant phenotype coefficients accumulate theirs metagenes of maximum correlation in the lower left corner of the map.

4. Supplementary Tables

Table S 1: Participant's characteristics of the LIFE ADULT study used in this publication for association with the blood transcriptome

Features		Men		Women		Comment
**Number of participants** [a)]		1,618		1,510		
**Age (mean ± SD)**		58.1 ± 12.4		59 ± 13		years
**Smoker/Ex-smoker**		**1,000**[e)]		701		±
**<30g alcohol per day**		**633**		218		
**Blood Count** [b)]	BA	0.034 ± 0.02		0.036 ± 0.02		Basophils absolute (10^9/l)
	BAP	0.58 ± 0.32		0.62 ± 0.36		Basophils (%)
	EO	0.18 ± 0.12		0.16 ± 0.12		Eosinophils absolute (10^9/l)
	EOP	3.0 ± 1.8		2.8 ± 1.9		Eosinophils (%)
	RBC	**4.83 ± 0.38**		4.45 ± 0.33		Erythrocytes (10^12/l)
	HCT	**0.429 ± 0.028**		0.393 ± 0.026		Hematocrit (l/l)
	HGBK	**14.7 ± 1.1**		13.2 ± 0.9		Hemoglobin (conv. units, g/dl)
	HGB	**9.13 ± 0.68**		8.21 ± 0.59		Hemoglobin (SI units, mmol/l)
	WBC	6.1 ± 1.8		6.0± 1.7		Leucocytes (109/l)
	LYP	29.5 ± 8.0		31.1 ± 7.9		Lymphocytes (%)
	LY	1.77 ± 0.69		1.81 ± 0.55		Lymphocytes absolute (109/l)
	MCHK	30.5 ± 1.6		29.8 ± 1.6		Mean corpuscular hemoglobin (conv. units, pg)
	MCH	1.90 ± 0.10		1.85 ± 0.10		Mean corpuscular hemoglobin (SI units, fmol)
	MCHCK	34.3 ± 1.0		33.6 ± 0.9		Mean corpuscular hemoglobin concentration (conv. units, g/dl)
	MCHC	21.31 ± 0.63		20.86 ± 0.56		Mean corpuscular hemoglobin concentration (SI units, mmol/l)
	MCV	88.9 ± 4.1		88.5 ± 4.0		Mean corpuscular volume (fl)
	MPV	10.6± 0.9		10.6 ± 0.9		Mean platelet volume (fl)
	MOP	8.8 ± 2.1		7.7 ± 2.0		Monocytes (%)
	MO	**0.53 ± 0.17**		0.45 ± 0.14		Monocytes absolute (109/l)
	NEP	58.0 ± 8.6		57.7 ± 8.7		Neutrophils (%)
	NE	3.6 ± 1.35		3.5 ± 1.37		Neutrophils absolute (109/l)
	RETI	11.6 ± 3.8		11.4 ± 3.7		Reticulocytes (/1000)
	PLT	226 ± 54		256 ± 55		Platelets (109/l)
**Blood Serum markers**	hsCRP	2.57 ± 4.6		3.03 ± 5.0		mg/l human serum C-reactive protein
	ferritin	**261± 179**		128 ± 192		ng/ml
	Transferritin	2.56 ± 0.39		2.57 ± 0.4		g/l
	cystatinC	1.0 ± 0.3		1.0 ± 0.2		mg/l
		**# men**	**mean age**	**# women**	**mean age**	
**BMI status**	uwt	14	39±9	**42**	46±10	underweight BMI < 18.5 kg/m^2)
	nwt	375	53±15	**492**	54±12	normal weight 18.5 < BMI < 25
	pre obese	**590**	60±12	443	60±12	25 < BMI < 30
	obese	**411**	63±11	311	61±11	30 < BMI
**Medication**[d)]	A	152	67±9	130	56±12	Alimentary tract and metabolism
	B	**75**	64±11	37	64±11	Blood and blood forming organs

	C	457	60±15	363	68±10	Cardiovascular system
	D	13	69±8	**27**	65±10	Dermatologicals
	G	50	70±8	**133**	60±13	Genitourinary system and sex hormones
	H	47	58±11	**141**	50±12	Systemic hormonal preparations, excl. sex hormones and insulins
	J	5	66±9	10	61±11	Anti-infective for systemic use
	L	5	67±10	10	60±12	Antineoplastic and immuno-modulating agents
	M	86	65±10	**105**	58±10	Muscular-skeletal system
	N	89	68±8	**134**	61±12	Nervous system
	P	0	63±10	0	61±11	Antiparasitic products, insecticides and repellents
	R	71	62±11	61	59±22	Respiratory system
	S	28	63±11	25	58±12	Sensory organs
	V	45	63±12	45	66±10	Various
**Disease history [e)]**	A(ngina pectoris)	**86**	67±9	47	69±8	Angina pectoris
	Art(hrosis)	340	64±11	**507**	64±10	Arthrosis
	Ast(hma)	113	60±15	146	57±12	Asthma
	Can(cer)	188	69±8	184	63±11	Cancer
	Cat(aract)	153	70±8	169	71±7	Cataract
	Dep(ression)	104	58±11	**205**	58±11	Depression
	Dia(betes)	**242**	66±9	135	67±9	Diabetes
	Glau(coma)	83	67±10	90	66±10	Glaucoma
	Gou(t)	**189**	65±10	74	66±11	Gout
	HA(eart attack)	**75**	68±8	24	68±9	Heart attack
	Hep(atitis)	165	63±10	173	64±10	Hepatitis
	HL(yper-lipidaemia)	616	62±11	500	63±11	Hyper-lipidaemia
	HT(ypertension)	962	63±11	744	64±10	Hypertension
	Hzo(ster)	210	63±12	269	63±11	Hzoster
	Rhe(uma)	66	66±10	106	61±12	Rheuma
	Sep(sis)	**120**	62±12	71	61±11	Sepsis
	Thy(roid)	221	63±11	**668**	59±11	Thyroid

a) For a detailed description of the LIFE-adult study see (26)

b) Analyses using clinical laboratory

c) Body types were derived from 3D laser scanning data using machine learning as described previously (27)

d) Considers medications taken within the last 7 days before the LIFE-core program visit (26). Medication was classified according to Anatomical Therapeutic Chemicals (ATCs) indexing, https://www.whocc.no /atc_ddd_index/)

e) Disease history of the participants was assessed in questionnaires (26).

f) larger values indicating sex-differences are shown in bold letters

Table S 2: Spot modules of co-expressed genes in the blood transcriptome: Enrichment was calculated using Fisher's Exact Test. In brackets there are the exponent of the p-values. The Associated Phenotypes column lists all phenotypes with maximal positive (+) or negative (-) correlation values between the overall metagene expression and the respective phenotype variable. See **Figure 1** and Table S 1 for assignment of phenotypes.

Spot	Short name<br># of genes	Top gene sets (exponent p-value)	Phenotype signatures	Associated Phenotypes
A	inflammation<br>332	LU_EZH2-targets_DN (-49), Chaussabel_2.7-unknown-funktion (-5), endodeoxyribonuclease activity (-5)		Neutrophils, Obesity (+)<br>Basophils, Lymphocytes (-)
B	Nicotin receptor<br>208	Neuromuscular synaptic transmission (-4) Reactome_rora_activates_circadian_expression (-4), ligand−gated ion channel activity (-3)		
C	Erythrocytes<br>362	Chaussabel_erythrocytes (-87), Hallmark_heme_metabolism (-67), Chaussabel_neutrophils (-16), oxygen transporter activity (-8), erythrocyte differentiation (-7)	Homuth_BMI−associated −genes_UP (-22) DUMEAUX_Red blood cells in non smokers literature genes up (-16)	Red blood cell components, Alcohol (+)
D	Active chromatin<br>238	LU_EZH2-targets_DN (-26), negative regulation of NF−kappaB transcription factor activity (-4)		
N	Platelets<br>213	Chaussabel_1.2-platelets (-99), Raghavacha_platelet_specific_genes (-59) Reactome_hemostasis (-18), Reactome_platelet_activation_signaling_and_aggregation (-17), platelet degranulation (-14), blood coagulation (-8)		Age (+)<br>Basophils, Platelets (-)
O	Inflammation<br>387	Chromatin states (-27), Theilgaard_neutrophil_at_skin_wound (-18), Protein binding (-9), Cytoplasm (-8)	Homuth_BMI−associated −genes_DN (-12)	Platelets, Age (+)<br>Mean platelet volume (-)
E	RNA processing, mitochondrion<br>556	Active and transcribed chromatin states (-99), LU_EZH2-targets-UP (-17), Mitochondrion, mitochondrial translation (≥-15), Chaussabel-3.7-splicosome (-13), Poly(A) RNA binding (-10), DNA_repair (-8)		Eosinophils, Monocytes (+)
F	Ribosome<br>44	Chaussabel-1.7-_MHC Ribosomal proteins (-5), ribosomal protein S26		
G	Mitochondrion<br>150	Mitochondrial translation active and transcribed states (-14), Mitochondrion (-14),		
H	Cytotoxic cells<br>278	Chaussabel_2.1-cytotoxic cells (-87), Regulation of immune response (-19), Wirth_immune system (-17), Li_induced_t_to_natural_killer (-14), Cellular defense response (-8), Immune response (-7), Cytolysis (-7)	Marjolein_ageing−genes_UP (-21) DUMEAUX_Women normal BMI literature genes up (-8) Huan_blood−pressure_SBP−signature (-7) DUMEAUX_Exercising non smoker literature enriched genes (-6)	Lymphocytes (+)<br>Neutrophils (-)

I	Translation, inflammation 421	Chaussabel_2.8_T-cells (-38), Chaussabel_1.7-MHC ribosomal proteins (-22), Chaussabel_2.4-Ribosomal proteins (-19), SRP−dependent cotranslational protein targeting to membrane (-17), rRNA processing (-15), Translation (-12), Chaussabel_1.3-B-cells (-9)	Marjolein_ageing−genes_DN (-18)	Pre-obesity (+)<br>Age (-)
J	MYC targets 326	Pujana_BRCA1_PCC_network (-30), Pujana_CHEK2_PCC_network (-24), poly(A) RNA binding (-19), Chaussabel_2.4_ribosomal proteins (-16), Hallmark_MYC-targets_v1 (-15)		Monocytes, Leucocytes (-)
K	Protein phosphatases 326	Active Chromatin states (-69), SHEN_SMARCA2_TARGETS_UP (-25), protein binding (-18), Chaussabel_3.4-Protein phosphatases (-16)		Smoking (-)
L	Interferon response 207	Chaussabel_3.1-interferon inducible (-99), Hallmarks interferon gamma&alpha response Interferon response and signaling (-79), Dauer_Stat3-targets_DN (-47), NYUTTEN_EZH2-targets_UP (-40) Defense response to virus (-37), type I interferon signaling pathway (-37)	DUMEAUX_Monocytes in smokers literature genes up (-5)	Lymphocytes (-)
M	Cytosol 262	Protein binding (-20), Cytosol, Exosomes (-13), Membrane (-12), WIRTH_Immune system (-11)	Homuth_BMI−associated−genes_DN (-8)	Eosinophils (-)
O	Myeloid lineage cells	THEILGAARD_NEUTROPHIL_AT_SKIN_WOUND_DN (-18), Chaussabel_2.6-myeloid_ilineage (-9), Chaussabel_inflammationI (-8),	Homuth_BMI−associated−genes_DN (-12)	

Table S 3: Top genes of the spot-modules (ranked with decreasing Pearsons correlation coefficient with mean spot expression profile, r). For full lists of genes see Supplementary File 3.

Spot	Top 10 genes [a)] Symbol (r)	Name
A	IL17RD (0.97)	interleukin 17 receptor D
	ALPP (0.97)	alkaline phosphatase, placental
	DMC1 (0.97)	DNA meiotic recombinase 1
	CSF2RA (0.97)	colony stimulating factor 2 receptor alpha subunit
	FAM175A (0.96)	family with sequence similarity 175 member A
	DTWD2 (0.96)	DTW domain containing 2
	SERF2 (0.96)	small EDRK-rich factor 2
	CCDC125 (0.96)	coiled-coil domain containing 125
	RHBDL2 (0.96)	rhomboid like 2
C	SELENBP1 (0.93)	selenium binding protein 1
	GMPR (0.93)	guanosine monophosphate reductase
	SLC6A10P (0.93)	solute carrier family 6 member 10, pseudogene
	DMTN (0.93)	dematin actin binding protein
	FAM83F (0.93)	family with sequence similarity 83 member F
	AHSP (0.92)	alpha hemoglobin stabilizing protein
	EPB42 (0.92)	erythrocyte membrane protein band 4.2
	HBD (0.92)	hemoglobin subunit delta
	SEMA6B (0.92)	semaphorin 6B
	TPRA1 (0.92)	transmembrane protein adipocyte associated 1
	SPRYD3 (0.92)	SPRY domain containing 3
D	GALNT3 (0.91)	glutathione peroxidase 8
	DCAF11 (0.91)	DDB1 and CUL4 associated factor 11
	DOPEY2 (0.89)	dopey family member 2
	ZNF860 (0.89)	zinc finger protein 860
	PIGX (0.89)	phosphatidylinositol glycan anchor biosynthesis class X
	SLC5A8 (0.89)	solute carrier family 5 member 8
	PPM1B (0.89)	protein phosphatase, Mg2+/Mn2+ dependent 1B
	SPC24 (0.89)	SPC24, NDC80 kinetochore complex component
	PPIAL4G (0.89)	peptidylprolyl isomerase A like 4G
E	FAAP100 (0.85)	Fanconi anemia core complex associated protein 100
	GPS1 (0.85)	G protein pathway suppressor 1
	NDUFS7 (0.84)	NADH:ubiquinone oxidoreductase core subunit S7
	WDR46 (0.84)	WD repeat domain 46
	PMPCA (0.84)	peptidase, mitochondrial processing alpha subunit
	ARL2 (0.85)	ADP ribosylation factor like GTPase 2
	IMP4 (0.85)	IMP4 homolog, U3 small nucleolar ribonucleoprotein
	RANGAP1 (0.85)	Ran GTPase activating protein 1
	POLRMT (0.85)	RNA polymerase mitochondrial
	DHX37 (0.85)	DEAH-box helicase 37
G	MRM3 (0.82)	mitochondrial rRNA methyltransferase 3
	ZC3HC1 (0.81)	zinc finger C3HC-type containing 1
	JMJD8 (0.8)	jumonji domain containing 8
	UBXN1 (0.79)	UBX domain protein 1
	COA3 (0.78)	cytochrome c oxidase assembly factor 3
	AFG3L2 (0.78)	AFG3 like matrix AAA peptidase subunit 2
	C8orf33 (0.78)	chromosome 8 open reading frame 33
	CNOT11 (0.77)	CCR4-NOT transcription complex subunit 11
	KDM1A (0.77)	lysine demethylase 1A

**H**	GZMA (0.93)	granzyme A
	PPP2R2B (0.91)	protein phosphatase 2 regulatory subunit Bbeta
	NKG7 (0.9)	natural killer cell granule protein 7
	ADGRG1 (0.89)	adhesion G protein-coupled receptor G1
	TGFBR3 (0.89)	transforming growth factor beta receptor 3
	NCALD (0.88)	neurocalcin delta
	PRF1 (0.87)	perforin 1
	FGFBP2 (0.86)	fibroblast growth factor binding protein 2
	RUNX3 (0.86)	runt related transcription factor 3
**I**	ABLIM1 (0.88)	actin binding LIM protein 1
	OCIAD2 (0.87)	OCIA domain containing 2
	CCR7 (0.85)	C-C motif chemokine receptor 7
	FAM102A (0.85)	family with sequence similarity 102 member A
	C12orf57 (0.85)	chromosome 12 open reading frame 57
	VEGFB (0.85)	vascular endothelial growth factor B
	IMPDH2 (0.85)	inosine monophosphate dehydrogenase 2
	UXT (0.85)	ubiquitously expressed prefoldin like chaperone
	SAE1 (0.85)	SUMO1 activating enzyme subunit 1
	POLR1E (0.85)	RNA polymerase I subunit E
	CD27 (0.84)	CD27 molecule
**J**	RPL15 (0.91)	ribosomal protein L15
	URI1 (0.91)	URI1, prefoldin like chaperone
	EIF4B (0.91)	eukaryotic translation initiation factor 4B
	RPL22 (0.9)	ribosomal protein L22
	ETS1 (0.88)	ETS proto-oncogene 1, transcription factor
	DOCK10 (0.88)	dedicator of cytokinesis 10
	FAM60A (0.88)	family with sequence similarity 60 member A
	MRPL3 (0.88)	mitochondrial ribosomal protein L3
	HNRNPR (0.88)	heterogeneous nuclear ribonucleoprotein R
	ZNF22 (0.88)	zinc finger protein 22
	DNMT1 (0.81)	DNA-Methyltransferase 1
**K**	FNDC3A (0.81)	fibronectin type III domain containing 3A
	TMED7 (0.79)	transmembrane p24 trafficking protein 7
	IKZF5 (0.79)	IKAROS family zinc finger 5
	OXR1 (0.78)	oxidation resistance 1
	ECHDC1 (0.78)	ethylmalonyl-CoA decarboxylase 1
	ARID2 (0.78)	AT-rich interaction domain 2
	RBM7 (0.77)	RNA binding motif protein 7
	UBR5 (0.77)	ubiquitin protein ligase E3 component n-recognin 5
	LMBRD2 (0.77)	LMBR1 domain containing 2
	SDHD (0.76)	succinate dehydrogenase complex subunit D
**L**	RSAD2 (0.86)	radical S-adenosyl methionine domain containing 2
	IFIT3 (0.86)	interferon induced protein with tetratricopeptide repeats 3
	SERPING1 (0.85)	serpin family G member 1
	PARP9 (0.85)	poly(ADP-ribose) polymerase family member 9
	OAS3 (0.85)	2'-5'-oligoadenylate synthetase 3
	EPSTI1 (0.85)	epithelial stromal interaction 1
	SAMD9L (0.84)	sterile alpha motif domain containing 9 like
	IFIT1 (0.84)	interferon induced protein with tetratricopeptide repeats 1
	IFI44L (0.84)	interferon induced protein 44 like
	HERC5 (0.84)	HECT and RLD domain containing E3 ubiquitin protein ligase 5
**M**	CHIC2 (0.86)	cysteine rich hydrophobic domain 2
	AP1G1 (0.85)	adaptor related protein complex 1 gamma 1 subunit
	CORO1C (0.84)	coronin 1C
	ADAM10 (0.84)	ADAM metallopeptidase domain 10
	FEM1C (0.84)	fem-1 homolog C
	AP1G1 (0.84)	adaptor related protein complex 1 gamma 1 subunit
	COQ10B (0.83)	coenzyme Q10B
	ZC3HAV1 (0.82)	zinc finger CCCH-type containing, antiviral 1
	LAMP2 (0.82)	lysosomal associated membrane protein 2
	CASC4 (0.82)	cancer susceptibility 4

**N**	ESAM (0.91)	endothelial cell adhesion molecule
	GP9 (0.9)	glycoprotein IX platelet
	CTDSPL (0.9)	CTD small phosphatase like
	GNG11 (0.89)	G protein subunit gamma 11
	PTCRA (0.89)	pre T-cell antigen receptor alpha
	ITGB5 (0.89)	integrin subunit beta 5
	SH3BGRL2 (0.89)	SH3 domain binding glutamate rich protein like 2
	PDE5A (0.89)	phosphodiesterase 5A
	CTTN (0.89)	cortactin
	PTGS1 (0.88)	prostaglandin-endoperoxide synthase 1
**O**	MCL1 (0.65)	BCL2 family apoptosis regulator
	IQSEC1 (0.65)	IQ motif and Sec7 domain 1
	TSHZ3 (0.65)	teashirt zinc finger homeobox 3
	E2F3 (0.65)	E2F transcription factor 3
	MAK (0.65)	male germ cell associated kinase
	MAP3K5 (0.65)	mitogen-activated protein kinase kinase kinase 5
	RNF216P1 (0.65)	ring finger protein 216 pseudogene 1
	GPCPD1 (0.65)	glycerophosphocholine phosphodiesterase 1
	MAPK1 (0.65)	mitogen-activated protein kinase 1
	KATNBL1 (0.65)	katanin regulatory subunit B1 like 1

Table S 4: Enrichment of gene sets taken from the repertoire of functional modules of the blood transcriptome (18)

Spot	Module (enrichment p-value, Fishers exact test)
A	M3.1_Signs and Symptoms 1,00E-99<br>M8.1_Cyclic AMP 5,60E-35<br>M14.60_Behavior 2,90E-13
B	
C	M9.2_Erythrocyte Membrane 3,67E-41<br>M11.3_Erythroid Cells 9,93E-32<br>M13.30_Biotransformation 1,38E-24<br>M14.53_Megakaryocyte-Erythroid Progenitor Cells 7,87E-24<br>M15.53_Erythrocytes 3,17E-23<br>M15.74_Metabolic Networks and Pathways 1,59E-22<br>M12.11_Oxidoreductases Acting on Aldehyde or Oxo Group Donors 6,15E-22<br>M13.26_Specialty Uses of Chemicals 8,02E-21<br>M16.96_Platelet Activating Factor 8,74E-10<br>M11.2_RNA Transport 2,21E-09<br>M16.19_Trauma, Nervous System 3,76E-09<br>M16.34_Cyclic AMP-Dependent Protein Kinases 1,59E-08
D	M13.20_Genetic Phenomena 8,65E-09<br>M14.73_Biotransformation 2,98E-06
E	M13.4_Carcinoma, Hepatocellular 1,08E-22<br>M12.4_Chromatin 1,08E-15<br>M13.24_Mutagenesis 7,44E-12<br>M15.46_Metabolic Networks and Pathways 1,18E-11<br>M15.23_Biotransformation 4,94E-11<br>M14.25_Hepatitis C 8,85E-11<br>M13.29_Nucleic Acid Hybridization 7,46E-09<br>M13.10_Lead 9,60E-09<br>M15.39_Lymphotoxin-beta 2,86E-07<br>M15.15_Proteolysis 3,34E-06<br>M14.55_Transcription, Genetic 9,40E-06
F	
G	M12.4_Chromatin 3,53E-10<br>M13.4_Carcinoma, Hepatocellular 3,51E-09
H	M13.21_Anti-Infective Agents 1,06E-41<br>M9.1_Cytotoxins 3,16E-39<br>M14.42_Intraepithelial Lymphocytes 6,19E-07
I	M13.27_Lymphocyte Specific Protein Tyrosine Kinase p56(lck) 4,16E-16<br>M11.1_Chemical Phenomena 4,77E-13<br>M12.6_Protein Structure, Secondary 2,05E-12<br>M14.42_Intraepithelial Lymphocytes 8,45E-11<br>M12.8_Antibodies, Monoclonal, Murine-Derived 2,78E-09<br>M13.2_DNA 3,03E-07<br>M14.80_Biotransformation 3,12E-07<br>M15.16_Dendritic Cells 5,75E-07<br>M14.31_Mutant Proteins 5,15E-06<br>M14.49_Epigenesis, Genetic 5,70E-06<br>M13.4_Carcinoma, Hepatocellular 8,58E-06<br>M12.1_Mitochondria Apoptotic9,38E-06
J	M13.2_DNA 1,83E-41<br>M13.6_Apoptosis 5,42E-19

	M12.3_Cell Growth Processes 4,71E-10<br>M12.1_Mitochondria Apoptotic 9,08E-09<br>M13.27_Lymphocyte Specific Protein Tyrosine Kinase p56(lck) 1,52E-06
K	M12.5_Basal Transcription Factors 1,37E-08<br>M15.45_Platelet Membrane Glycoproteins 1,39E-08<br>M13.9_RNA Splicing 4,11E-08<br>M16.25_Alternative Splicing 2,77E-07<br>M15.44_RNA, Messenger 4,15E-06
L	M10.1_RIG-I 1,47E-45<br>M8.3_Transcriptome 8,04E-39<br>M13.17_STAT2 Transcription Factor 4,13E-38<br>M15.127_2,5-oligoadenylate 1,57E-16<br>M15.64_Tripartite Motif Proteins 6,98E-15<br>M12.2_Acetaldehyde 1,39E-11<br>M15.86_Metallothionein 4,20E-10<br>M13.1_Mitogen-Activated Protein Kinase 1 2,43E-07<br>M15.37_Free Radical Scavengers 2,26E-06
M	M13.25_Protein Interaction Maps 1,95E-14<br>M13.7_RNA Interference 2,99E-12<br>M13.19_Protein Domains 6,00E-11<br>M15.99_Protein Structure, Secondary 6,67E-09<br>M14.66_Caspase Cascade 4,65E-08<br>M14.27_Reverse Transcription 2,49E-07<br>M13.1_Mitogen-Activated Protein Kinase 1 3,11E-06
N	M8.2_Integrin beta3 7,74E-51<br>M10.3_Adhesiveness 3,94E-44<br>M14.59_Thromboplastin 7,41E-30<br>M16.64_Myosin Light Chains 2,31E-25<br>M14.39_beta-Galactosidase 6,05E-13<br>M14.81_SN12C Cells 3,19E-12
O	M13.3_Arsenicals 3,84E-16<br>M13.7_RNA Interference 1,99E-13<br>M15.43_Amyloidosis 2,78E-08<br>M13.1_Mitogen-Activated Protein Kinase 1 3,11E-08<br>M16.67_Herpes Simplex 3,82E-08<br>M14.7_Amino Acid Substitution 9,07E-08<br>M15.66_Endoribonucleases 4,43E-07<br>M15.14_Phosphatidylcholines 4,74E-07<br>M14.9_Colorectal Neoplasms 5,93E-07<br>M16.22_Metamorphosis, Biological 5,95E-07<br>M14.76_Hyaluronan Receptors 9,40E-07<br>M16.70_Gene Duplication 1,03E-06<br>M14.26_Hybrid Cells 1,39E-06<br>M13.16_Cryoprotective Agents 3,93E-06<br>M15.24_Methotrexate 5,78E-06

Table S 5: Seasonal (summer versus winter) changes of blood counts of the transcriptome subcohort of LIFE: p-values of differential count data (t-test) for women and men. Red coloring means higher blood count values in summer and blue in winter.

blood count	p-val male	female
HGBK	0.079	0.16
HGB	0.081	0.16
RBC	0.11	0.60
HCT	0.68	0.63
RETI	2.2e-16 ***	9.74e-12 ***
MCHK	1.89e-07 ***	1.97e-05 ***
MCH	8.89e-08 ***	1.89e-05 ***
MCHCK	5.61e-07 ***	0.02 *
MCHC	6.1e-7 ***	0.02 *
MCV	0.004 **	0.0007 ***
WBC	0.96	0.60
MOP	0.37	0.42
MO	0.32	0.84
EO	0.36	0.11
EOP	0.33	0.16
NE	0.69	0.96
NEP	0.18	0.35
BAP	6.2e-12 ***	1.41e-11 ***
BA	9.9e-10 ***	2.7e-12 ***
LYP	0.55	0.89
LY	0.46	0.34
PLT	0.99	0.38
MPV	0.016 *	0.50

5. References

1. Gentleman R, Carey V, Bates D, Bolstad B, Dettling M, Dudoit S, et al. Bioconductor: open software development for computational biology and bioinformatics. *Genome Biol* (2004) 5:R80. PubMed PMID: doi:10.1186/gb-2004-5-10-r80.

2. Du P, Kibbe WA, Lin SM. lumi: a pipeline for processing Illumina microarray. *Bioinformatics* (2008) 24(13):1547-8. doi: 10.1093/bioinformatics/btn224.

3. Jiang L, Schlesinger F, Davis CA, Zhang Y, Li R, Salit M, et al. Synthetic spike-in standards for RNA-seq experiments. *Genome Res* (2011) 21(9):1543-51. Epub 2011/08/04. doi: 10.1101/gr.121095.111. PubMed PMID: 21816910.

4. Cohen Freue GV, Hollander Z, Shen E, Zamar RH, Balshaw R, Scherer A, et al. MDQC: a new quality assessment method for microarrays based on quality control reports. *Bioinformatics* (2007) 23(23):3162-9. doi: 10.1093/bioinformatics/btm487.

5. Johnson WE, Li C, Rabinovic A. Adjusting batch effects in microarray expression data using empirical Bayes methods. *Biostat* (2006) 8(1):118-27. doi: 10.1093/biostatistics/kxj037.

6. Barbosa-Morais NL, Dunning MJ, Samarajiwa SA, Darot JFJ, Ritchie ME, Lynch AG, et al. A re-annotation pipeline for Illumina BeadArrays: improving the interpretation of gene expression data. *Nucl Acids Res* (2009) 38(3):e17-e. doi: 10.1093/nar/gkp942.

7. Wirth H, Löffler M, von Bergen M, Binder H. Expression cartography of human tissues using self organizing maps. *BMC Bioinformatics* (2011) 12(1):306. doi: 10.1186/1471-2105-12-306.

8. Hopp L, Wirth H, Fasold M, Binder H. Portraying the expression landscapes of cancer subtypes: A glioblastoma multiforme and prostate cancer case study. *Systems Biomedicine* (2013) 1(2):99-121.

9. Loeffler-Wirth H, Kreuz M, Hopp L, Arakelyan A, Haake A, Cogliatti SB, et al. A modular transcriptome map of mature B cell lymphomas. *Genome Medicine* (2019) 11(1):27. doi: 10.1186/s13073-019-0637-7.

10. Liberzon A, Birger C, Thorvaldsdóttir H, Ghandi M, Mesirov Jill P, Tamayo P. The Molecular Signatures Database Hallmark Gene Set Collection. *Cell Systems* (2015) 1(6):417-25.

11. Nersisyan L, Hopp L, Loeffler-Wirth H, Galle J, Loeffler M, Arakelyan A, et al. Telomere length maintenance and its transcriptional regulation in Lynch syndrome and sporadic colorectal carcinoma. *Frontiers in Oncology* (2019) in press.

12. Roadmap Epigenomics Consortium, Kundaje A, Meuleman W, Ernst J, Bilenky M, Yen A, et al. Integrative analysis of 111 reference human epigenomes. *Nature* (2015) 518(7539):317-30. doi: 10.1038/nature14248

http://www.nature.com/nature/journal/v518/n7539/abs/nature14248.html#supplementary-information.

13. Ben-Porath I, Thomson MW, Carey VJ, Ge R, Bell GW, Regev A, et al. An embryonic stem cell-like gene expression signature in poorly differentiated aggressive human tumors. *Nat Genet* (2008) 40(5):499-507. doi: http://www.nature.com/ng/journal/v40/n5/suppinfo/ng.127_S1.html.

14. Teschendorff AE, Menon U, Gentry-Maharaj A, Ramus SJ, Weisenberger DJ, Shen H, et al. Age-dependent DNA methylation of genes that are suppressed in stem cells is a hallmark of cancer. *Genome Res* (2010) 20. doi: 10.1101/gr.103606.109.

15. Hebenstreit D, Fang M, Gu M, Charoensawan V, van Oudenaarden A, Teichmann SA. RNA sequencing reveals two major classes of gene expression levels in metazoan cells. *Mol Syst Biol* (2011) 7. doi: http://www.nature.com/msb/journal/v7/n1/suppinfo/msb201128_S1.html.

16. Chaussabel D, Quinn C, Shen J, Patel P, Glaser C, Baldwin N, et al. A Modular Analysis Framework for Blood Genomics Studies: Application to Systemic Lupus Erythematosus. *Immunity* (2008) 29(1):150-64. doi: 10.1016/j.immuni.2008.05.012.

17. Peters MJ, Joehanes R, Pilling LC, Schurmann C, Conneely KN, Powell J, et al. The transcriptional landscape of age in human peripheral blood. *Nature Communications* (2015) 6:8570. doi: 10.1038/ncomms9570

https://www.nature.com/articles/ncomms9570#supplementary-information.

18. Altman MC, Rinchai D, Baldwin N, Whalen E, Garand M, Kabeer BA, et al. A Novel Repertoire of Blood Transcriptome Modules Based on Co-expression Patterns Across Sixteen Disease and Physiological States. *bioRxiv* (2019):525709. doi: 10.1101/525709.

19. Hopp L, Loeffler-Wirth H, Nersisyan L, Arakelyan A, Binder H. Footprints of Sepsis Framed Within Community Acquired Pneumonia in the Blood Transcriptome. *Frontiers in Immunology* (2018) 9(1620). doi: 10.3389/fimmu.2018.01620.

20. Newman AM, Liu CL, Green MR, Gentles AJ, Feng W, Xu Y, et al. Robust enumeration of cell subsets from tissue expression profiles. *Nature Methods* (2015) 12:453. doi: 10.1038/nmeth.3337

https://www.nature.com/articles/nmeth.3337#supplementary-information.

21. Dumeaux V, Olsen KS, Nuel G, Paulssen RH, Børresen-Dale A-L, Lund E. Deciphering Normal Blood Gene Expression Variation—The NOWAC Postgenome Study. *PLoS Genet* (2010) 6(3):e1000873. doi: 10.1371/journal.pgen.1000873.

22. Huan T, Esko T, Peters MJ, Pilling LC, Schramm K, Schurmann C, et al. A Meta-analysis of Gene Expression Signatures of Blood Pressure and Hypertension. *PLoS Genet* (2015) 11(3):e1005035. doi: 10.1371/journal.pgen.1005035.

23. Homuth G, Wahl S, Müller C, Schurmann C, Mäder U, Blankenberg S, et al. Extensive alterations of the whole-blood transcriptome are associated with body mass index: results of an mRNA profiling study involving two large population-based cohorts. *BMC Medical Genomics* (2015) 8:65. doi: 10.1186/s12920-015-0141-x. PubMed PMID: PMC4608219.

24. McLachlan JL, Smith AJ, Bujalska IJ, Cooper PR. Gene expression profiling of pulpal tissue reveals the molecular complexity of dental caries. *Biochimica et Biophysica Acta (BBA) - Molecular Basis of Disease* (2005) 1741(3):271-81. doi: https://doi.org/10.1016/j.bbadis.2005.03.007.

25. Tan F-L, Moravec CS, Li J, Apperson-Hansen C, McCarthy PM, Young JB, et al. The gene expression fingerprint of human heart failure. *Proceedings Of The National Academy Of Sciences Of The United States Of America* (2002) 99(17):11387-92. Epub 2002/08/12. doi: 10.1073/pnas.162370099. PubMed PMID: 12177426.

26. Loeffler M, Engel C, Ahnert P, Alfermann D, Arelin K, Baber R, et al. The LIFE-Adult-Study: objectives and design of a population-based cohort study with 10,000 deeply phenotyped adults in Germany. *BMC Public Health* (2015) 15:691. doi: 10.1186/s12889-015-1983-z. PubMed PMID: PMC4509697.

27. Löffler-Wirth H, Willscher E, Ahnert P, Wirkner K, Engel C, Loeffler M, et al. Novel Anthropometry Based on 3D-Bodyscans Applied to a Large Population Based Cohort. *PLOS one* (2016) 11(7):e0159887. doi: 10.1371/journal.pone.0159887.
