# Supplementary material for: The Human Blood Transcriptome in a Large Population Cohort and Its Relation to Aging and Health
Source: Front Big Data. 2020 Oct 30;3:548873. doi: 10.3389/fdata.2020.548873 (PMC7931910; doi:10.3389/fdata.2020.548873)

ST 1.1 / male

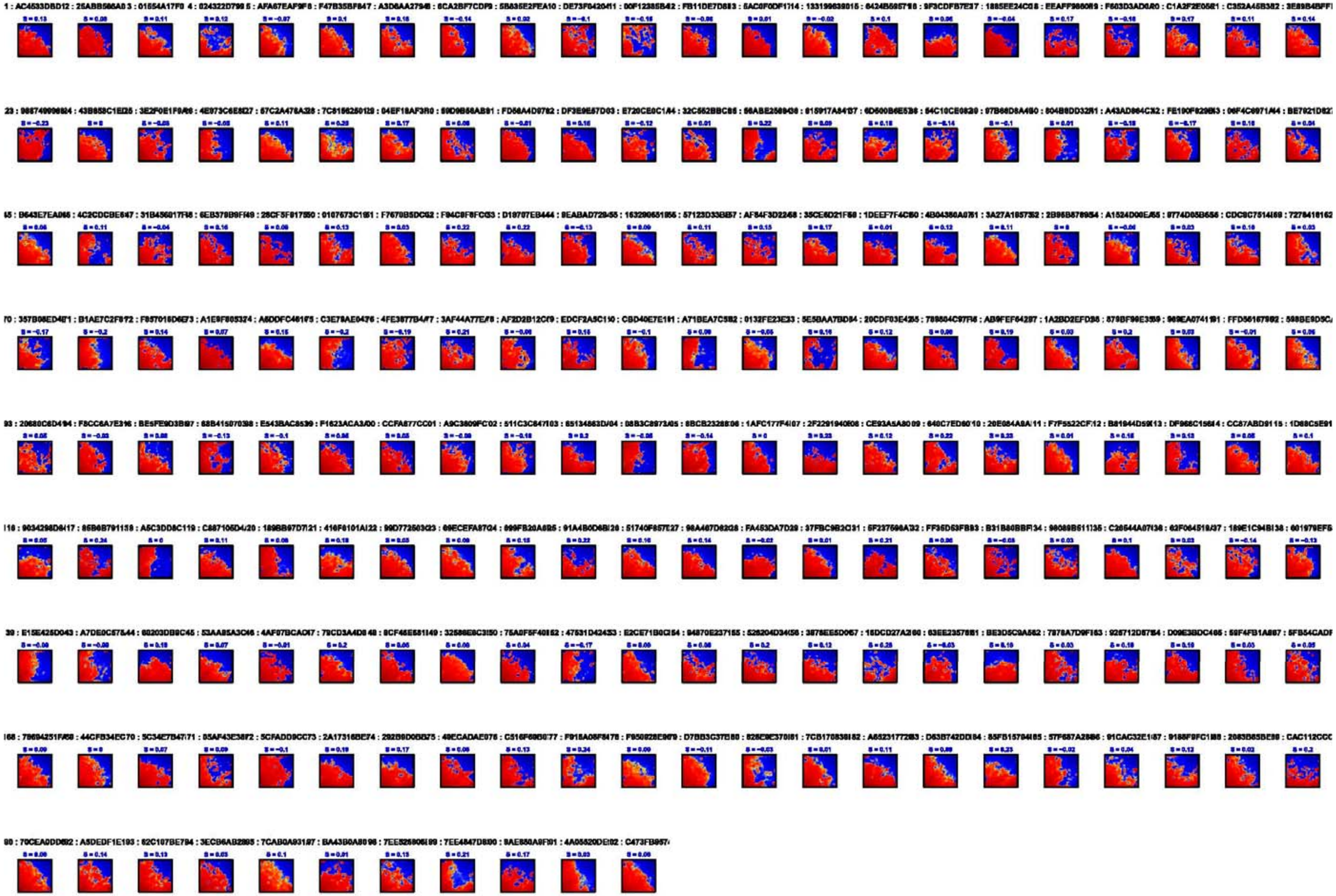

ST 1.1 / female

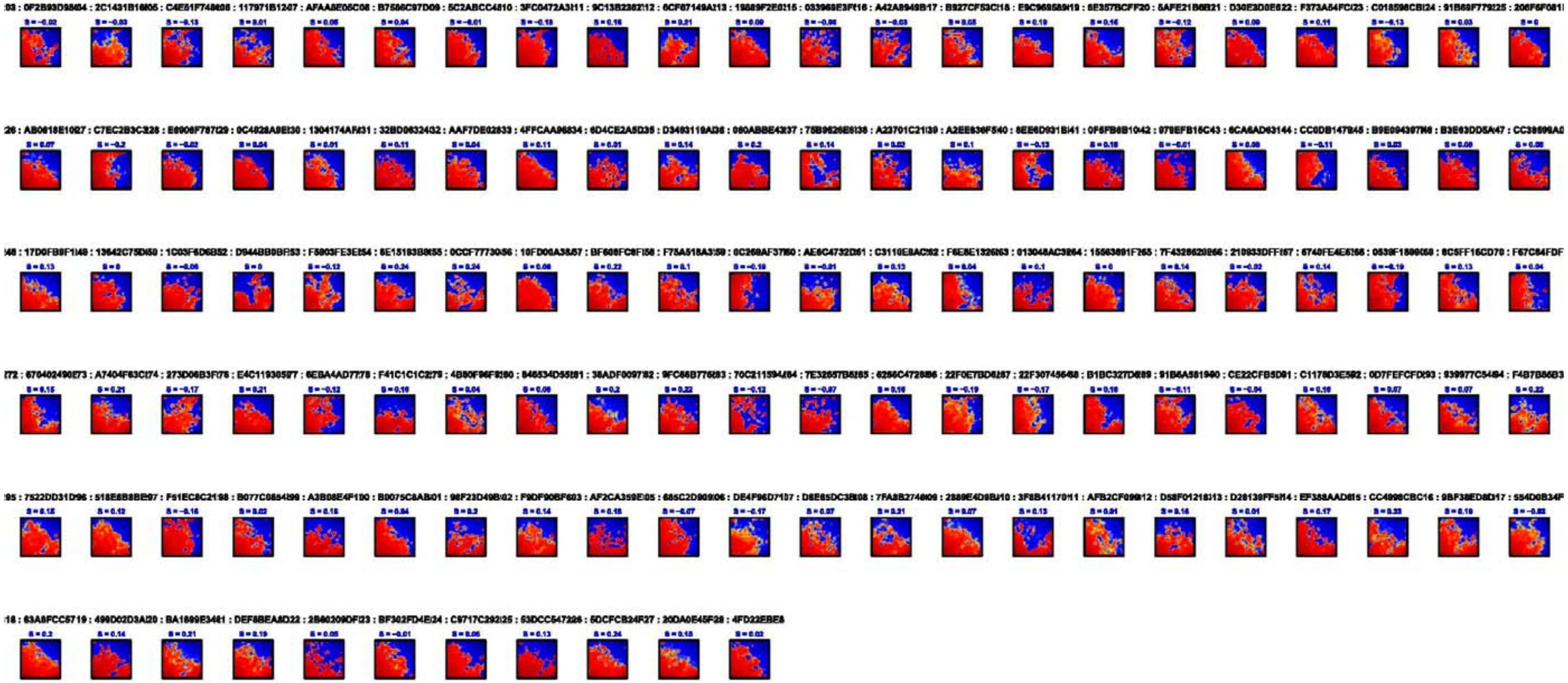

ST 1.2 / male

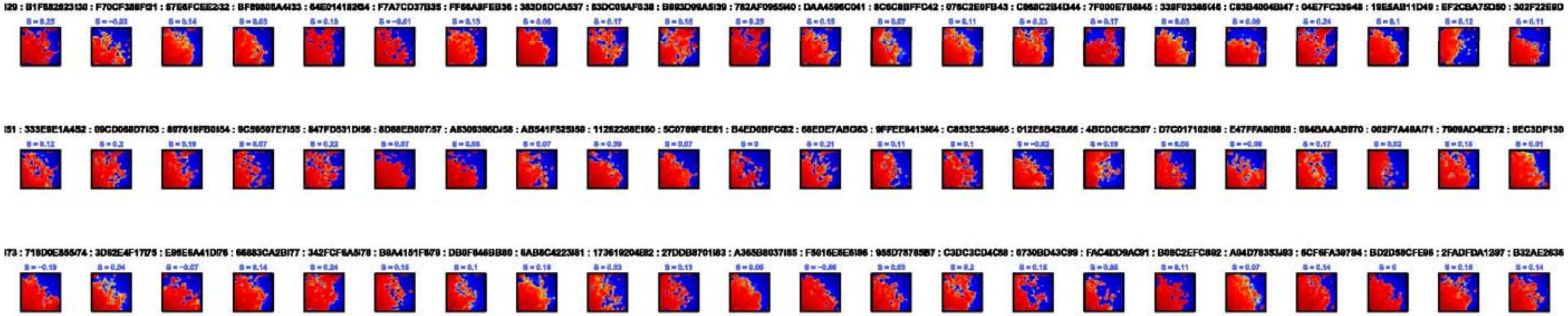

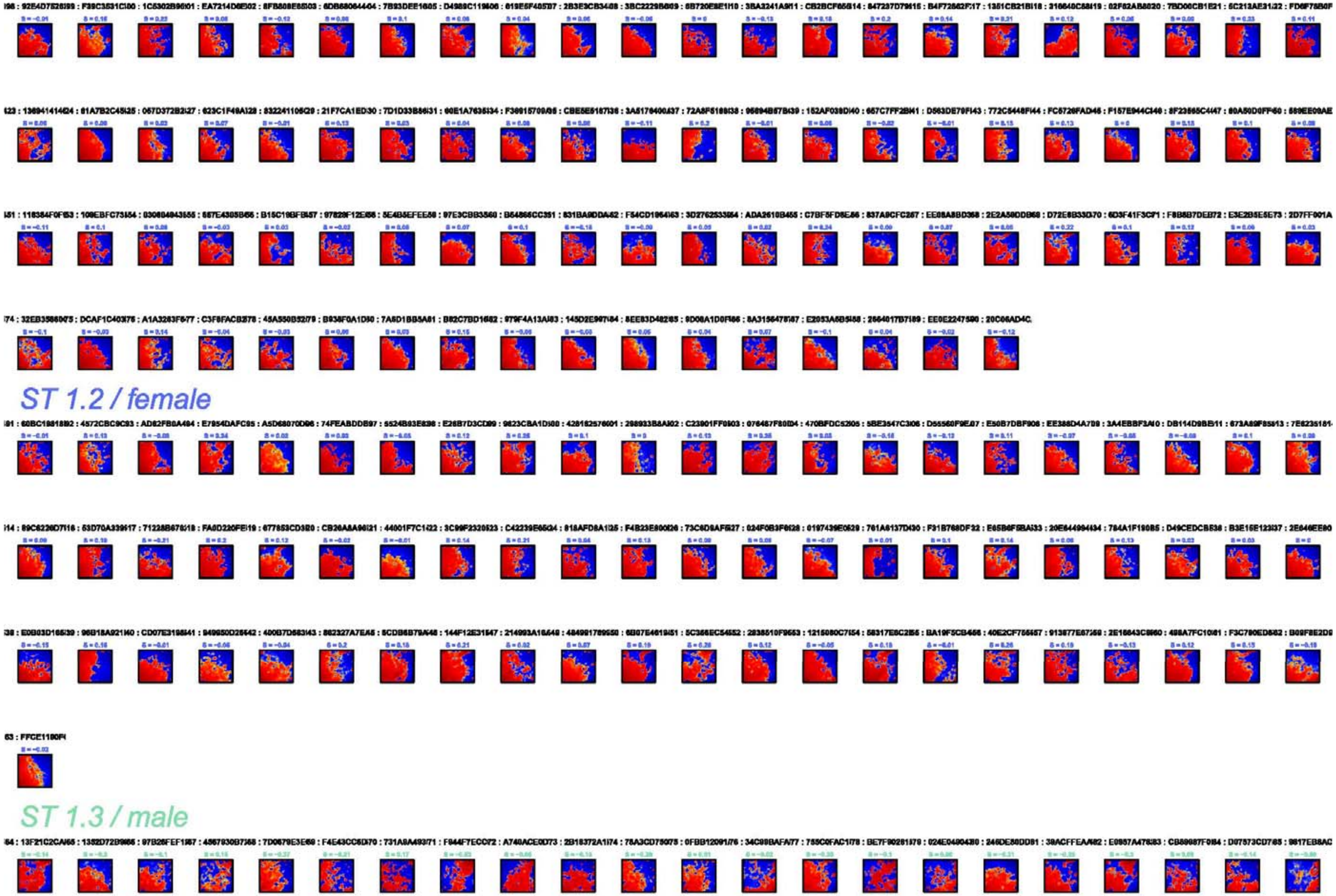
ST 1.2 / female
ST 1.3 / male

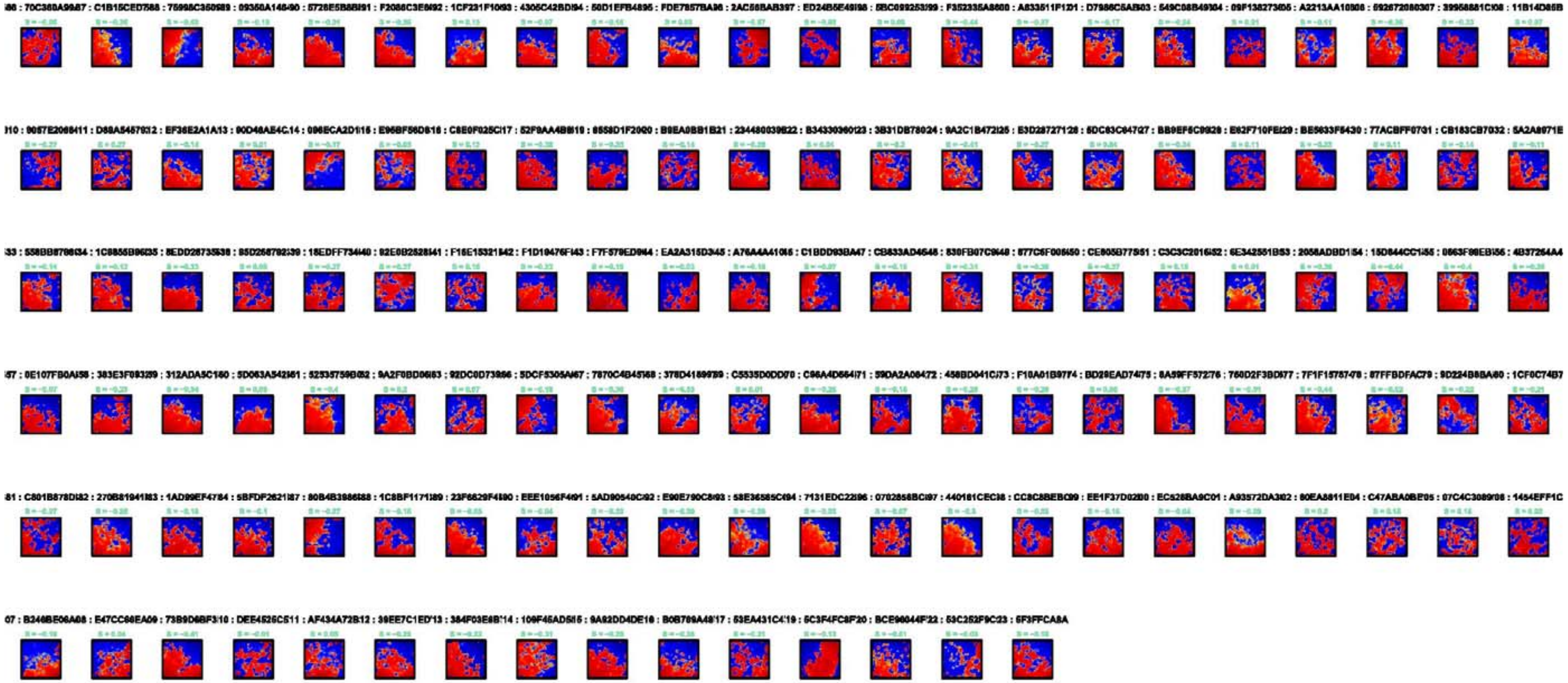

ST 1.3 / female

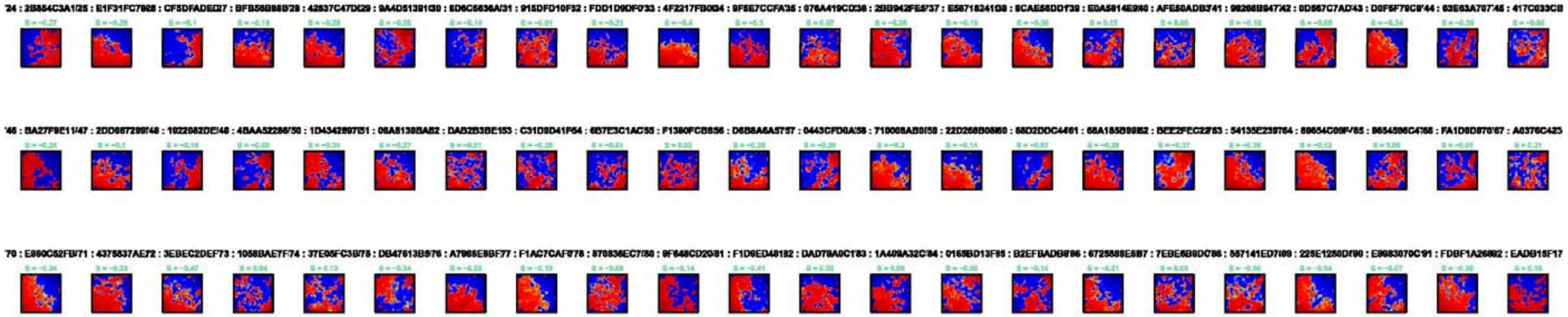

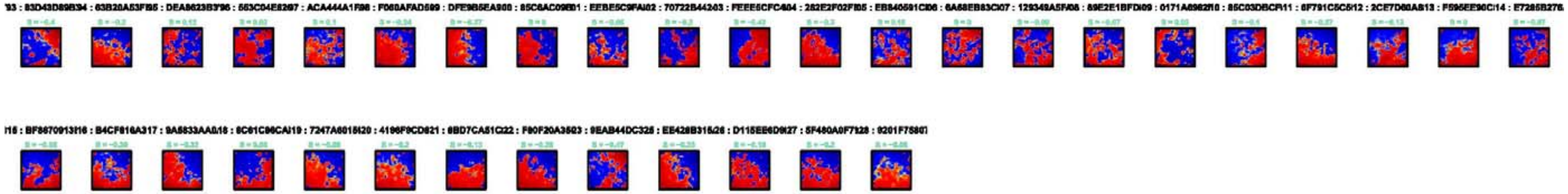

ST M.1 / male

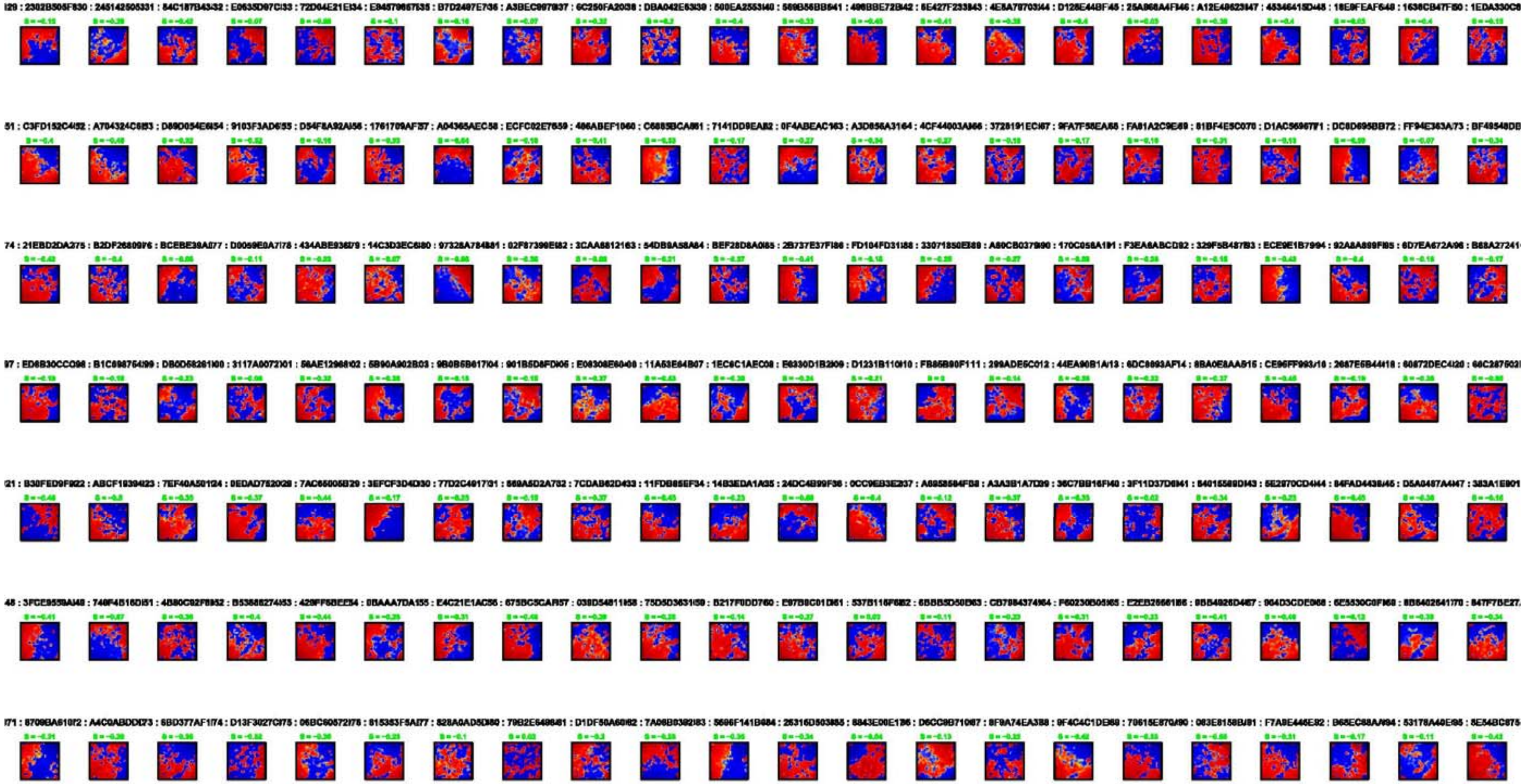

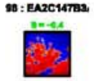

ST M.1 / female

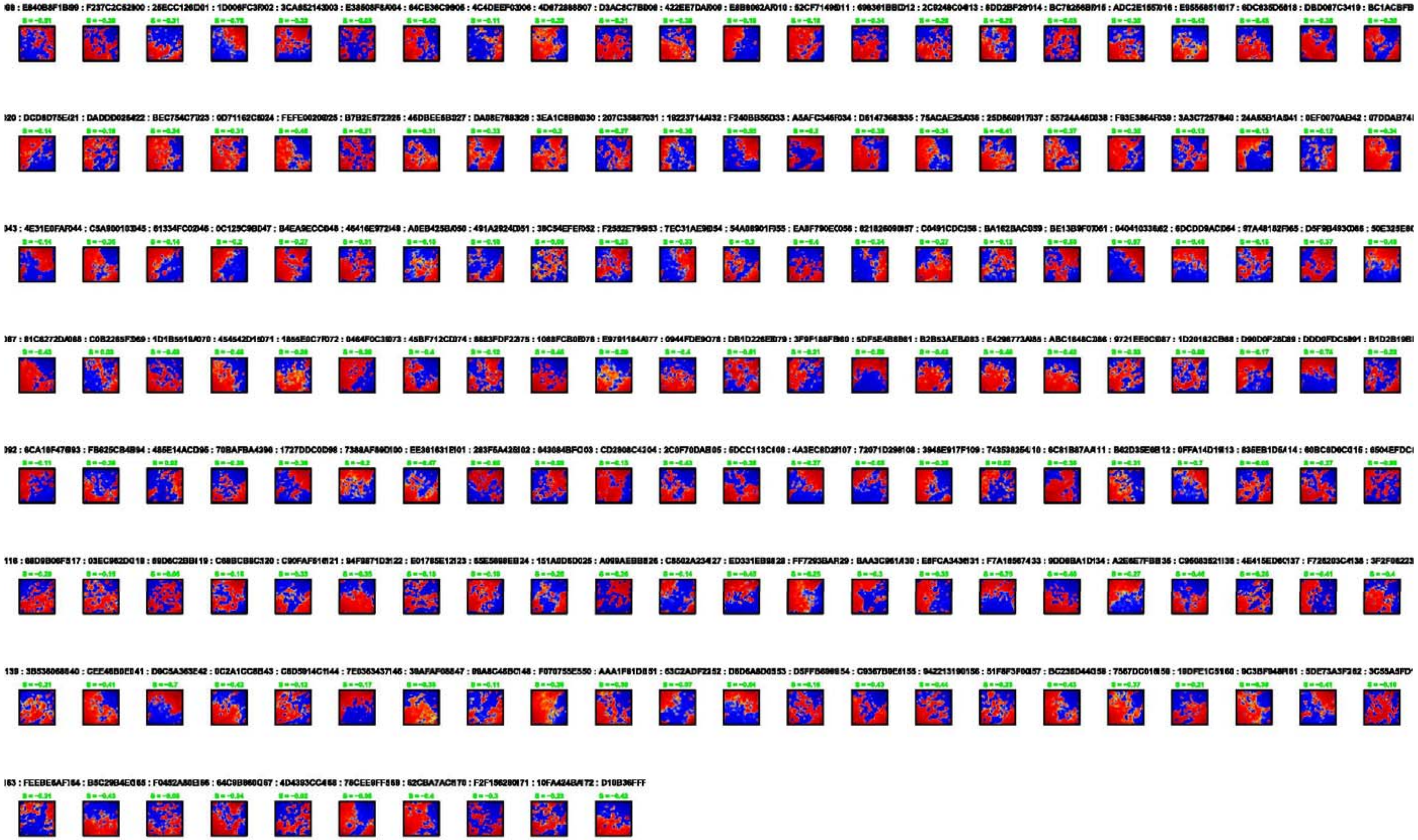

ST M.2 / male

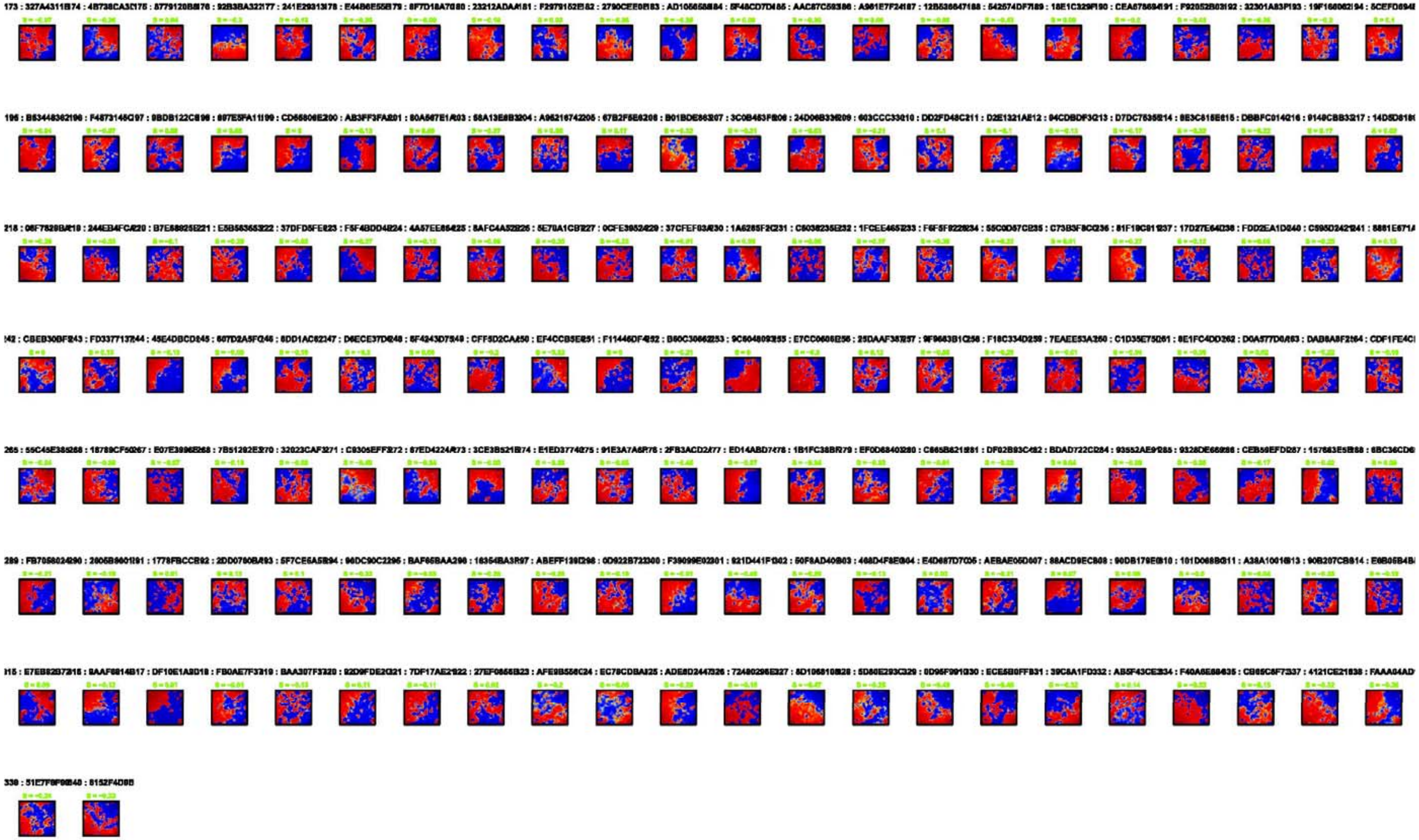

ST M.2 / female

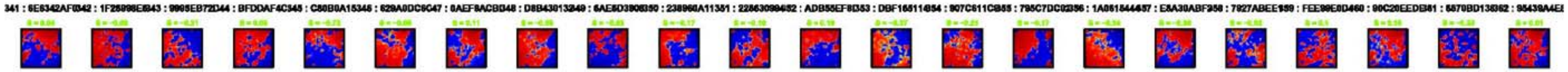

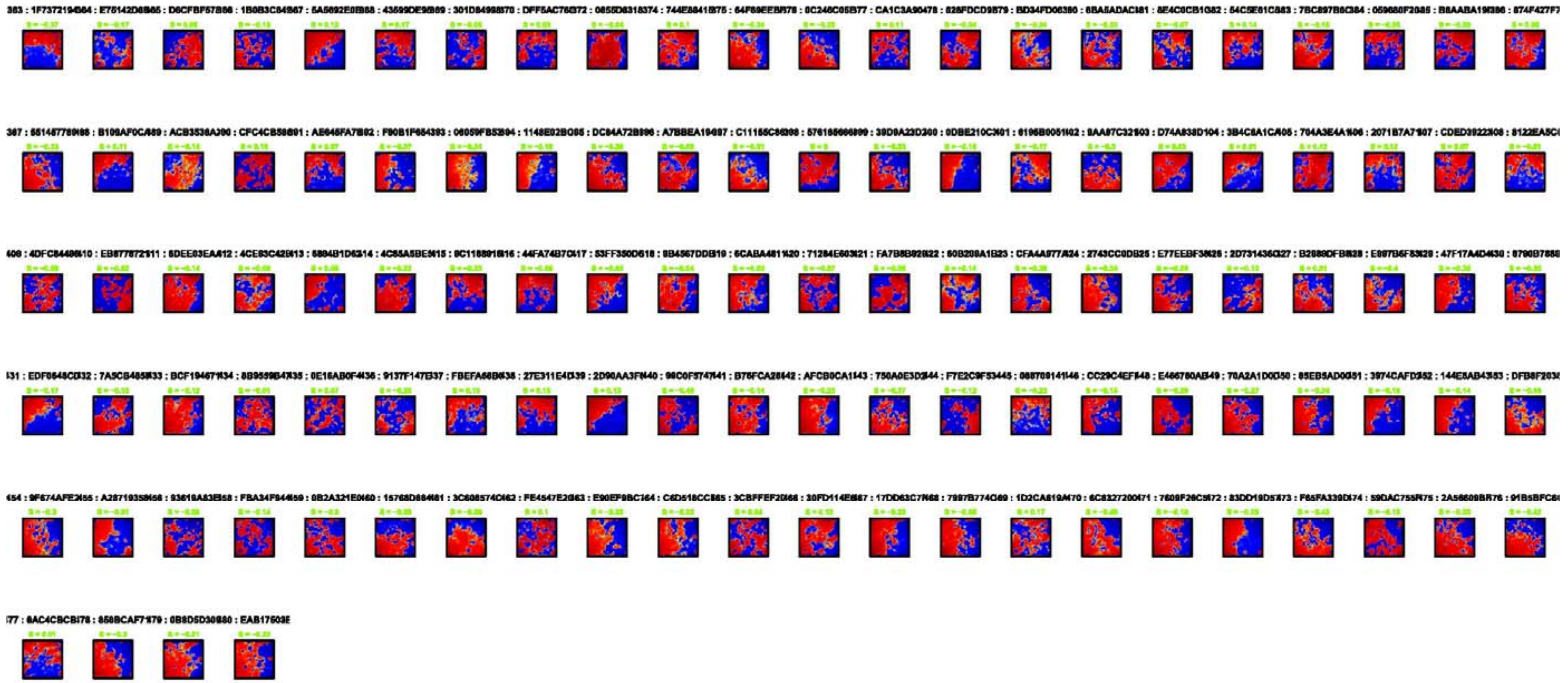

ST M.3 / male

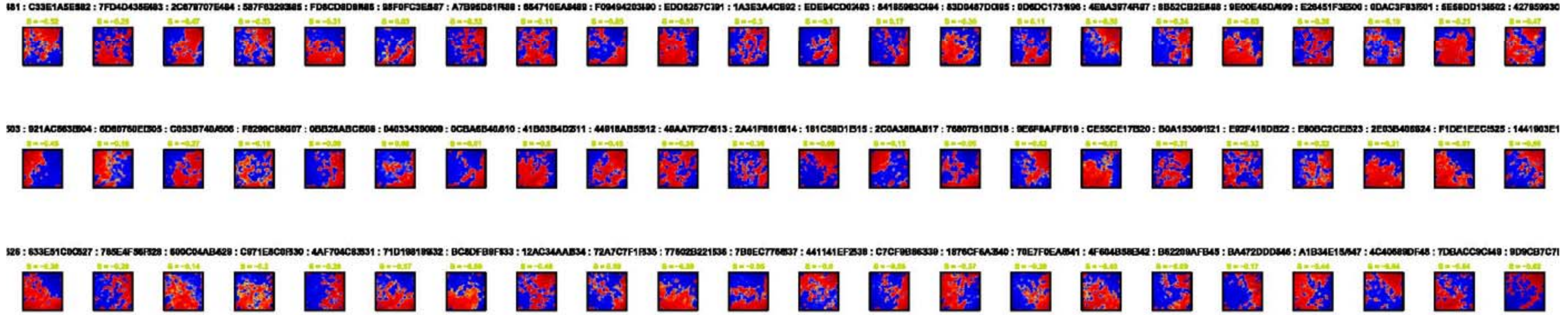

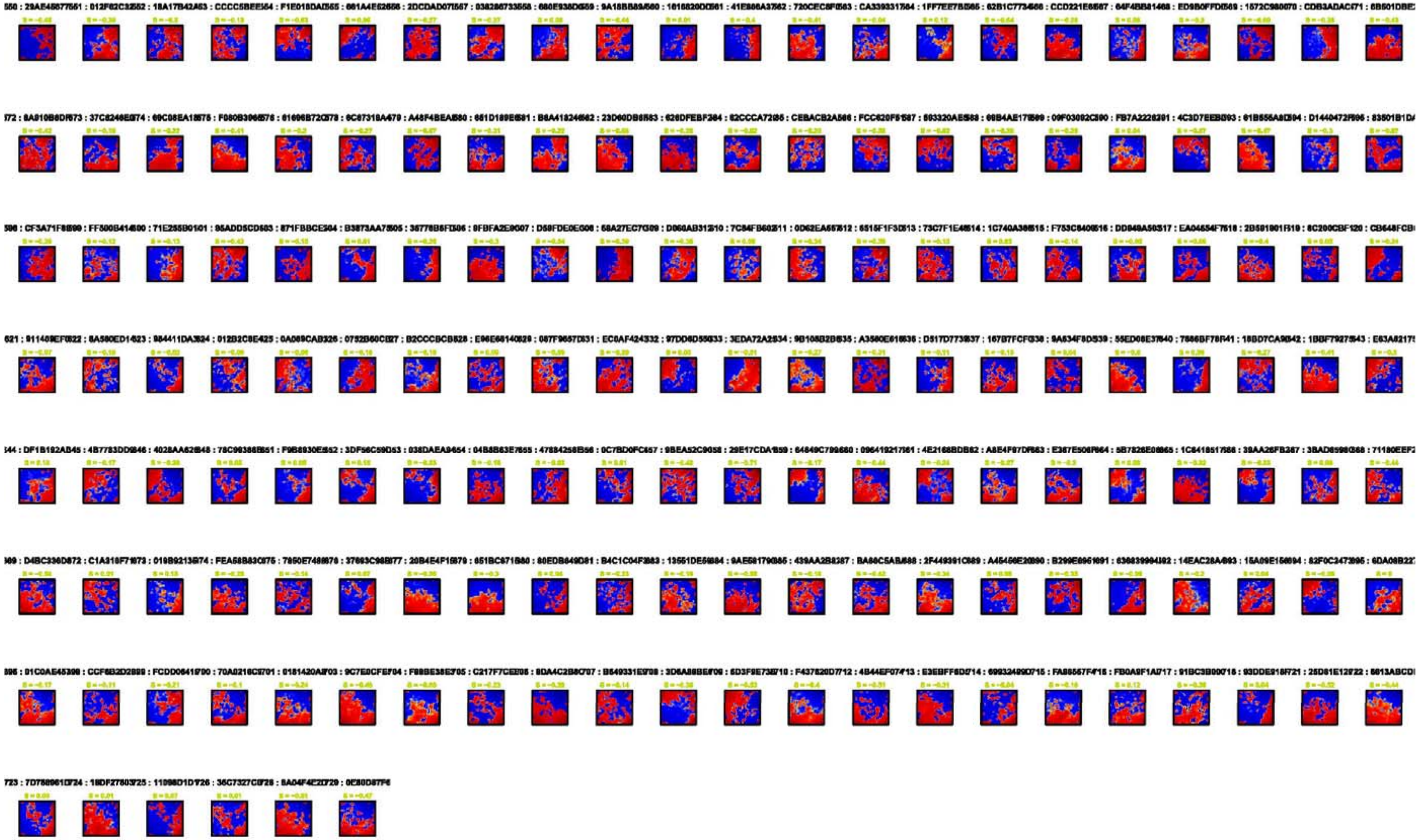

ST M.3 / female

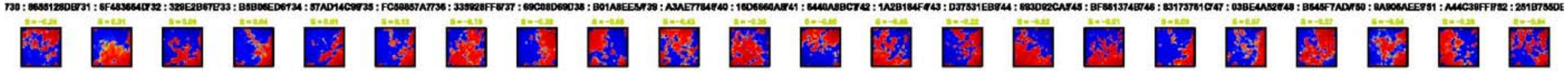

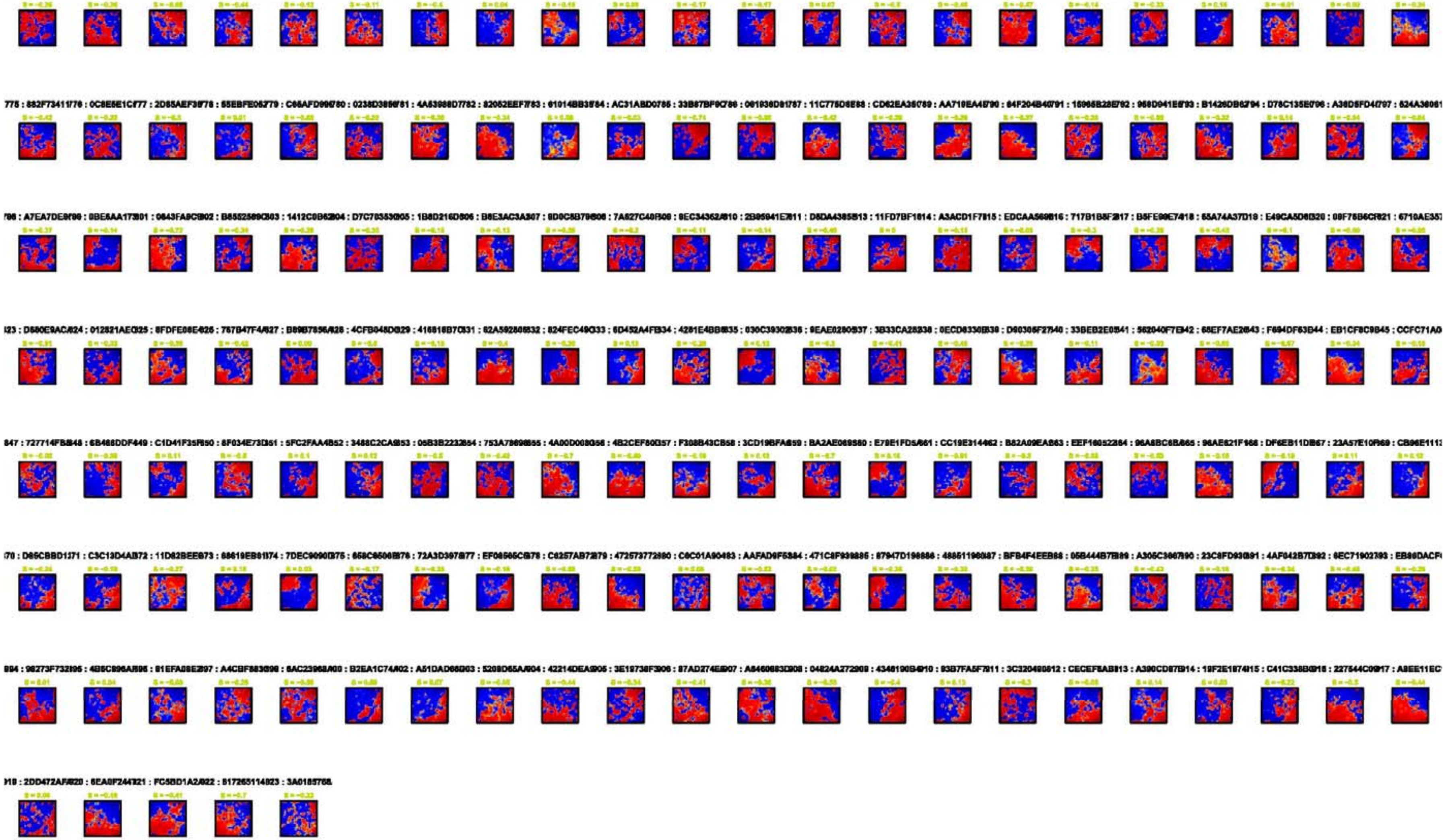

ST 2.1 / male

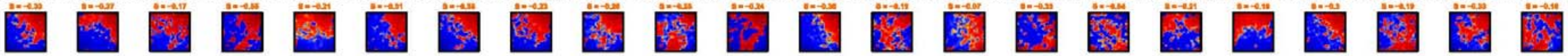

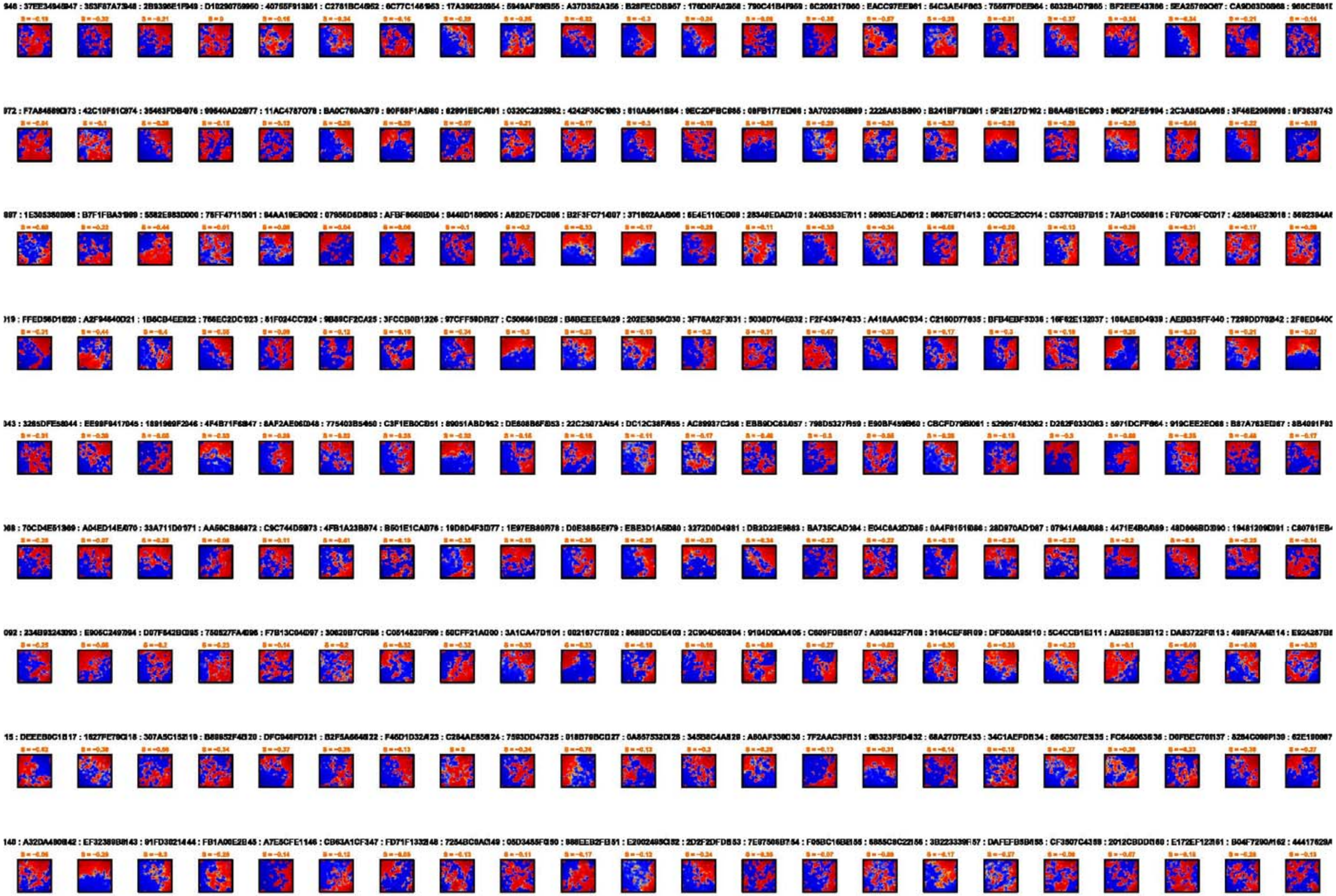

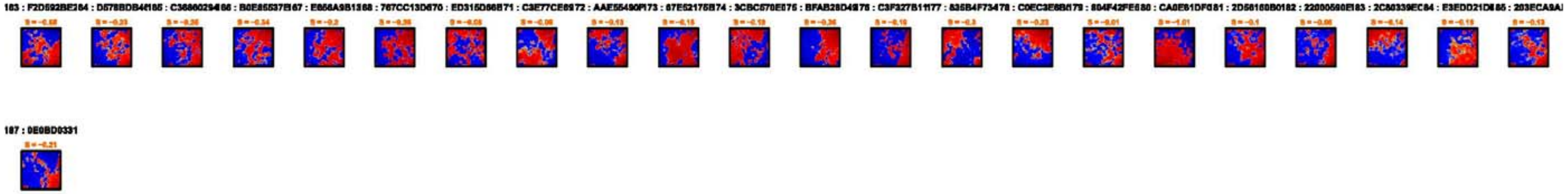

ST 2.1 / female

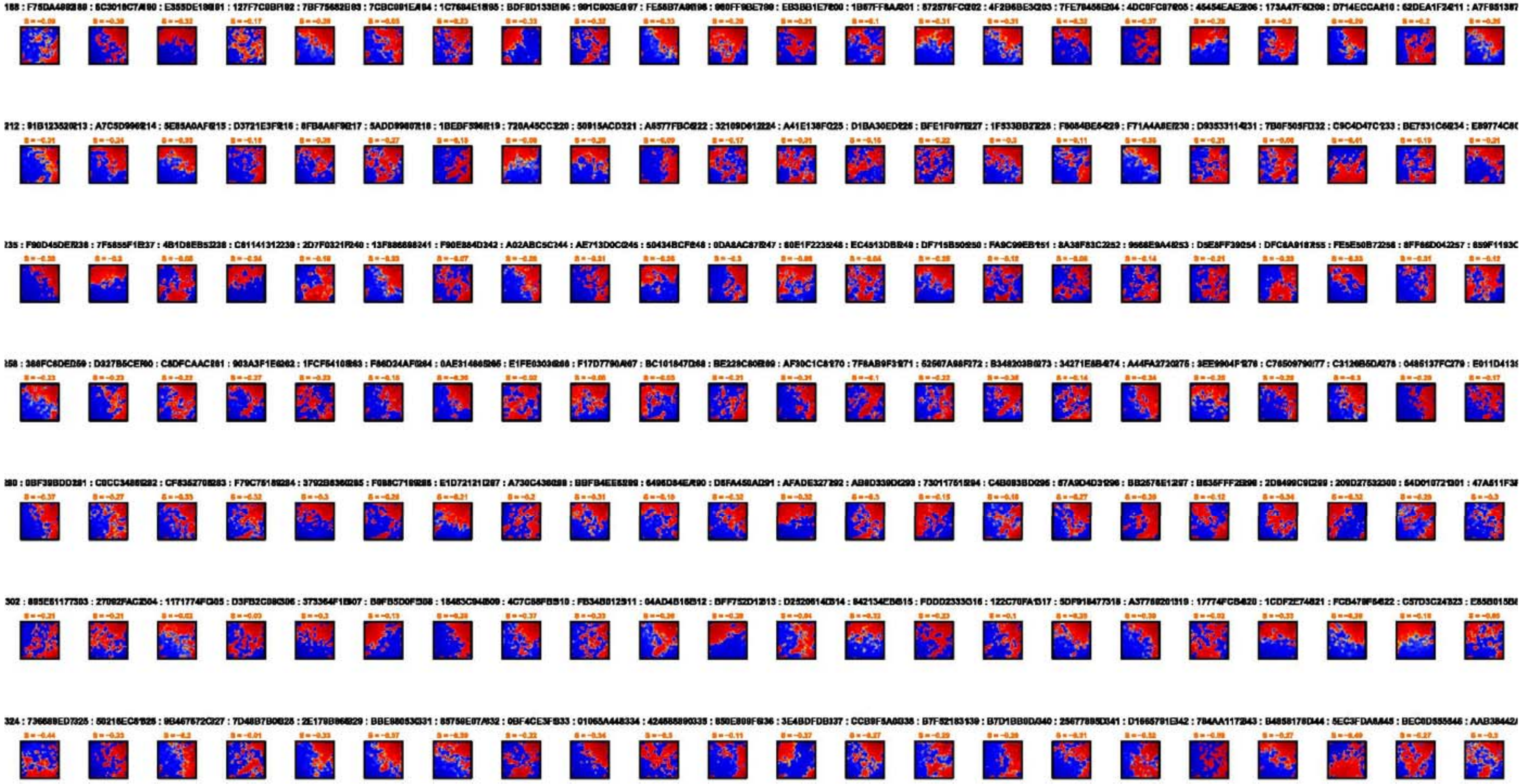

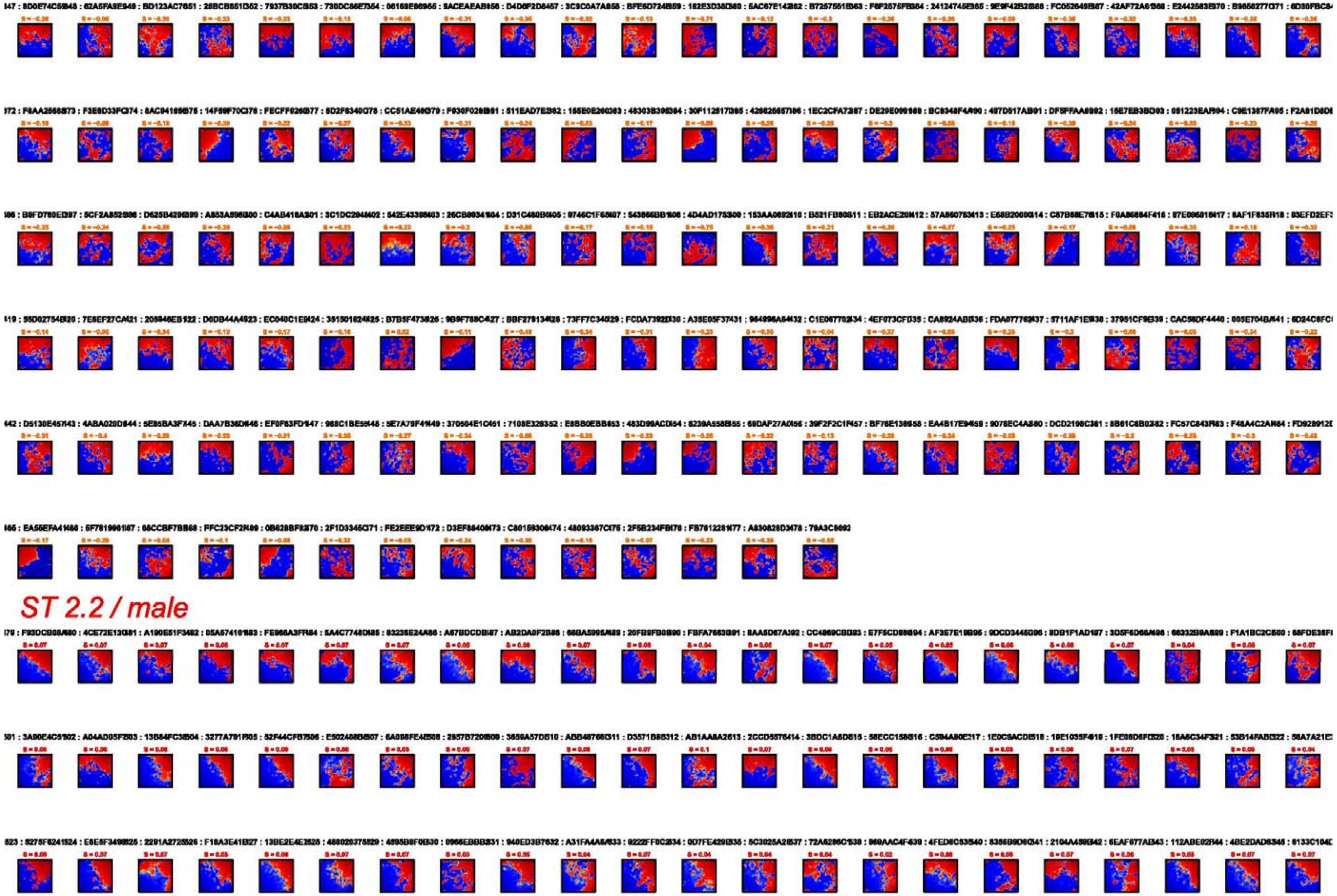
ST 2.2 / male

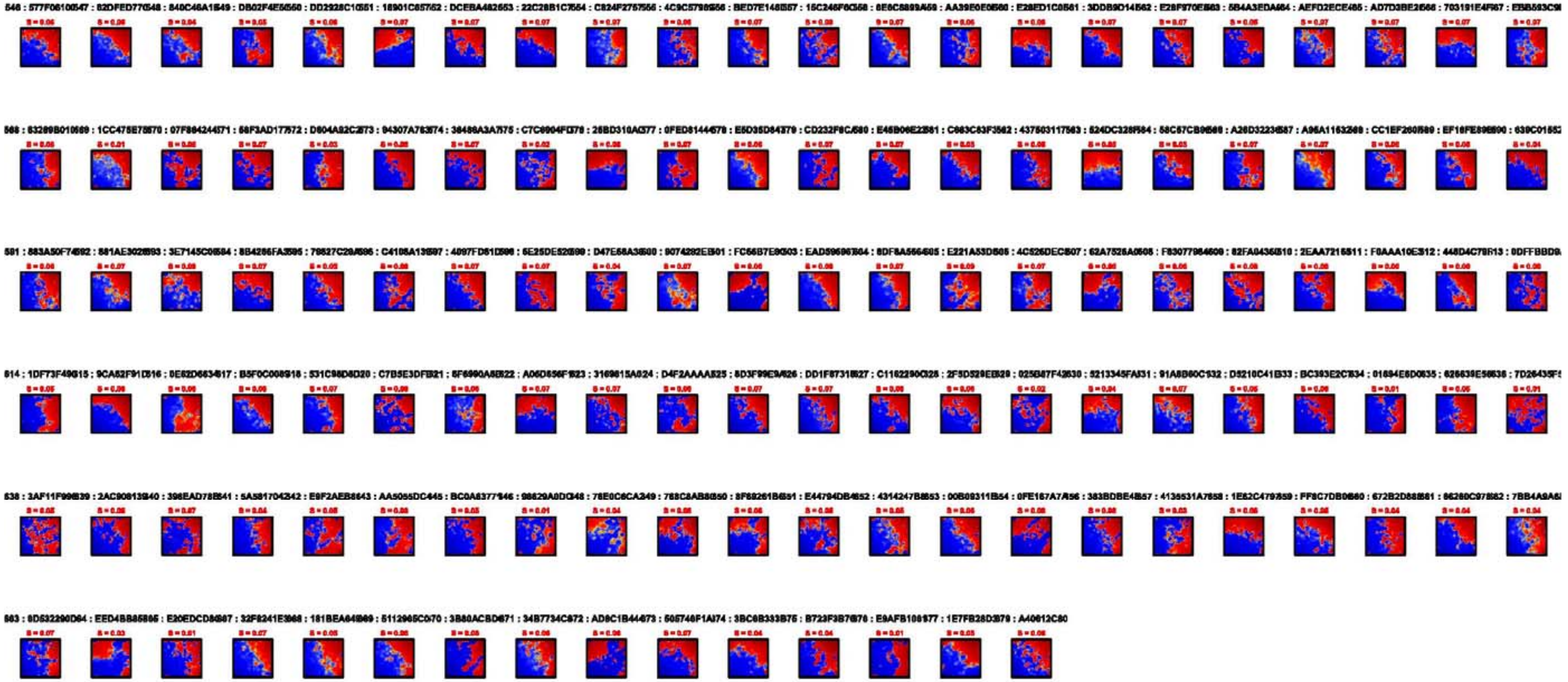

ST 2.2 / female

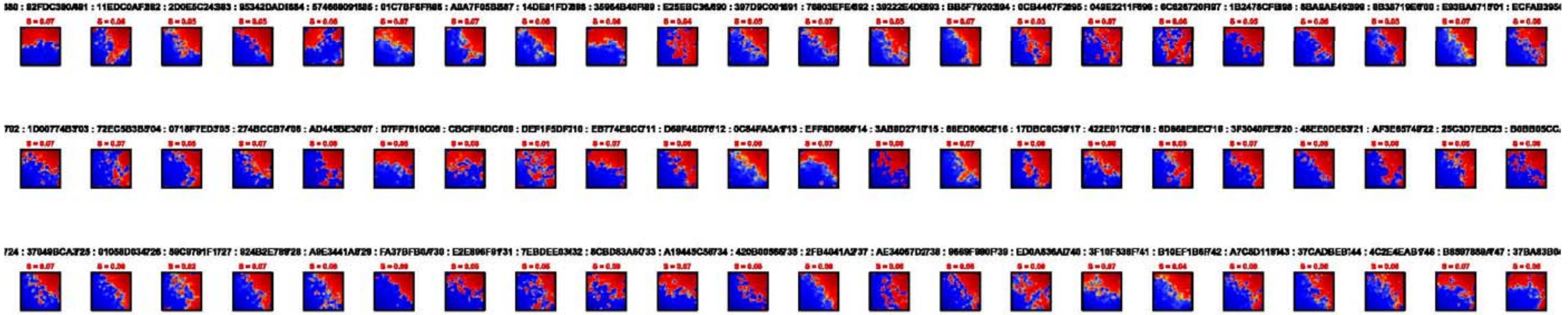

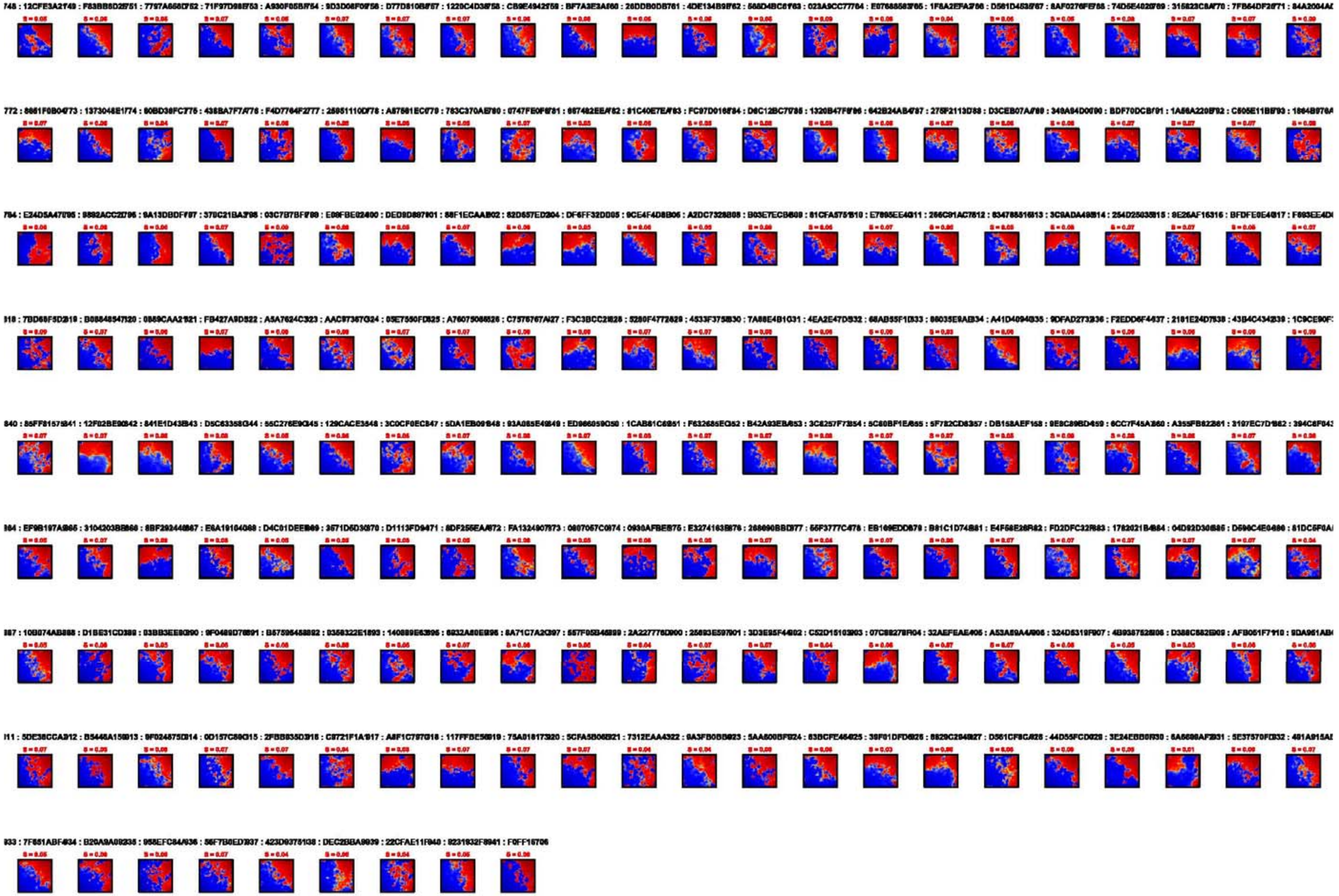

ST 2.3 / male

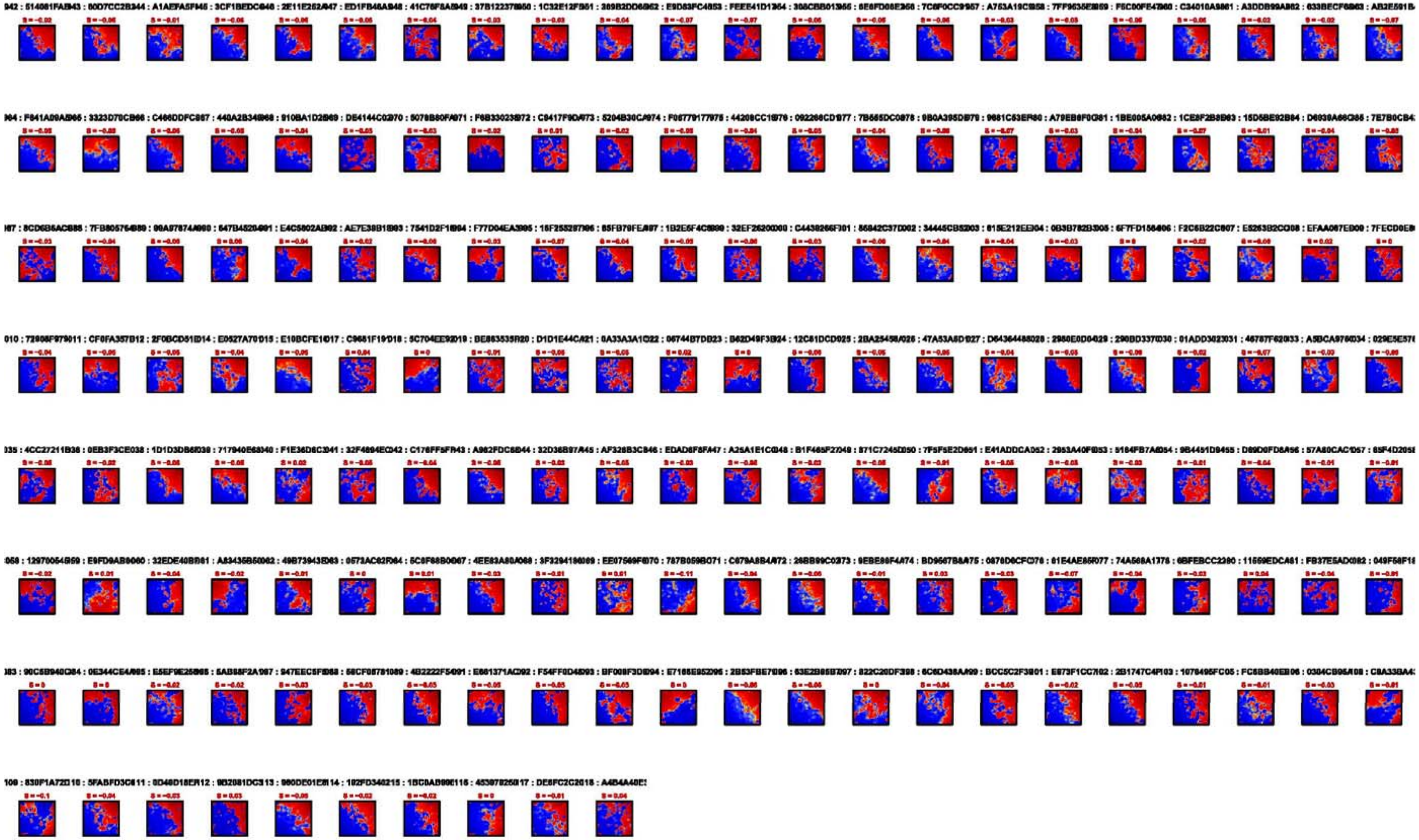

ST 2.3 / female

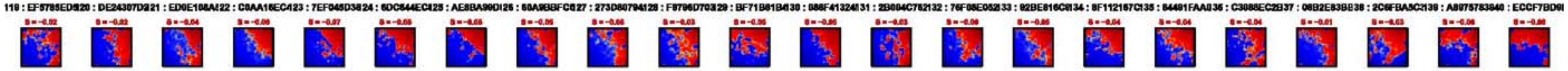

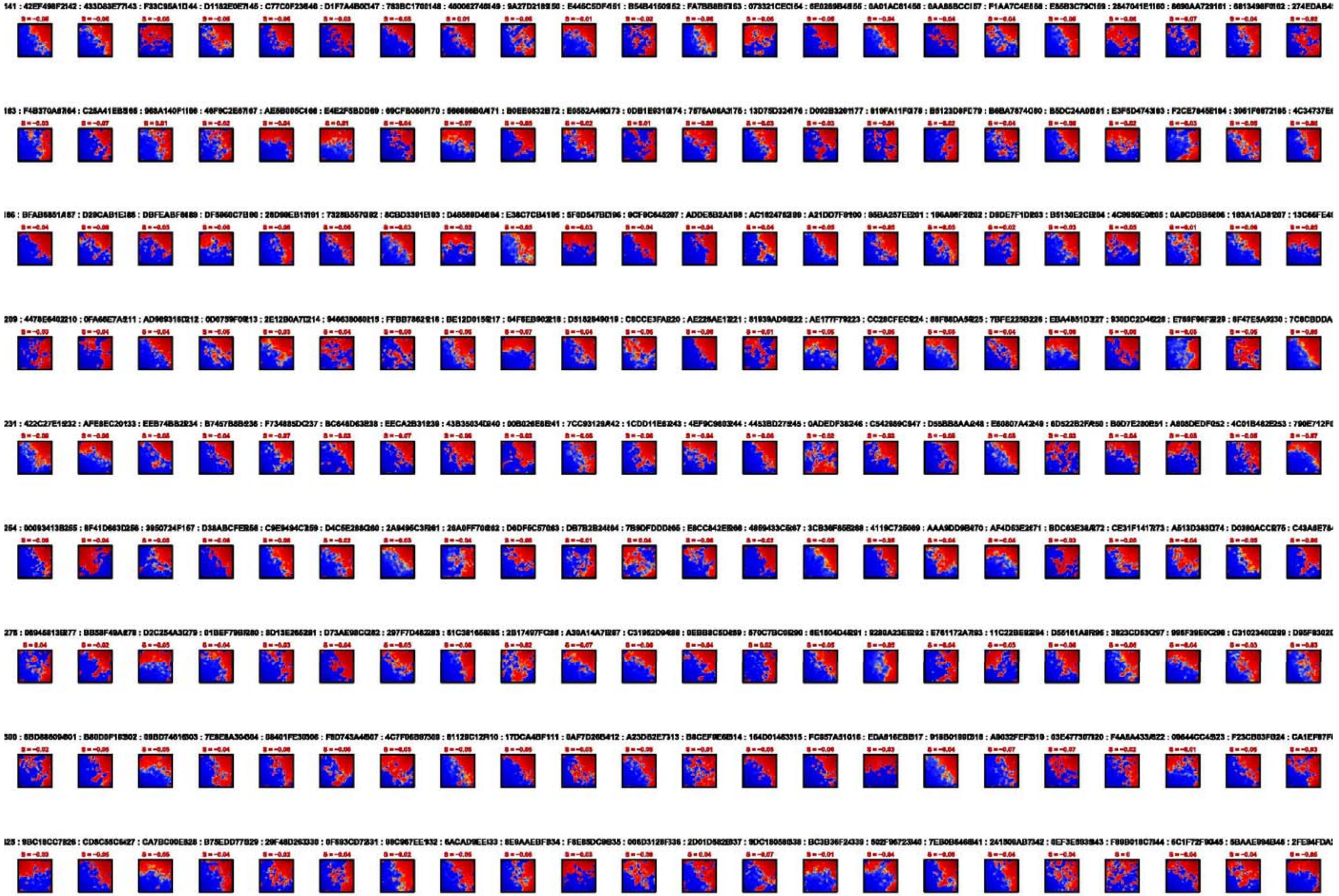

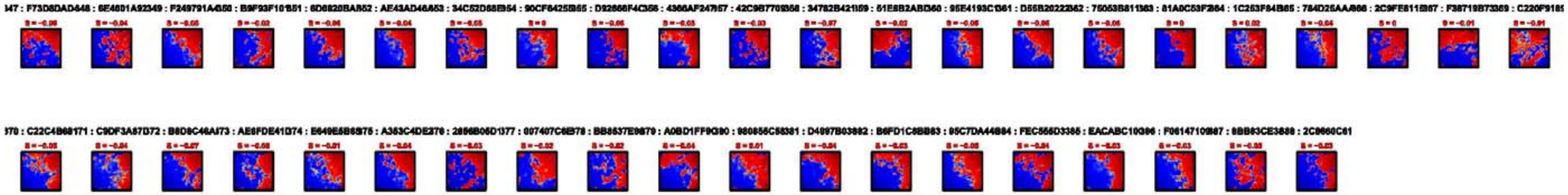

Supplement: Supplementary file 2 [file Data_Sheet_2.pdf]
