# Supplementary material for: The Human Blood Transcriptome in a Large Population Cohort and Its Relation to Aging and Health
Source: Front Big Data. 2020 Oct 30;3:548873. doi: 10.3389/fdata.2020.548873 (PMC7931910; doi:10.3389/fdata.2020.548873)

Altman_blood_M3.1_Signs and Symptoms

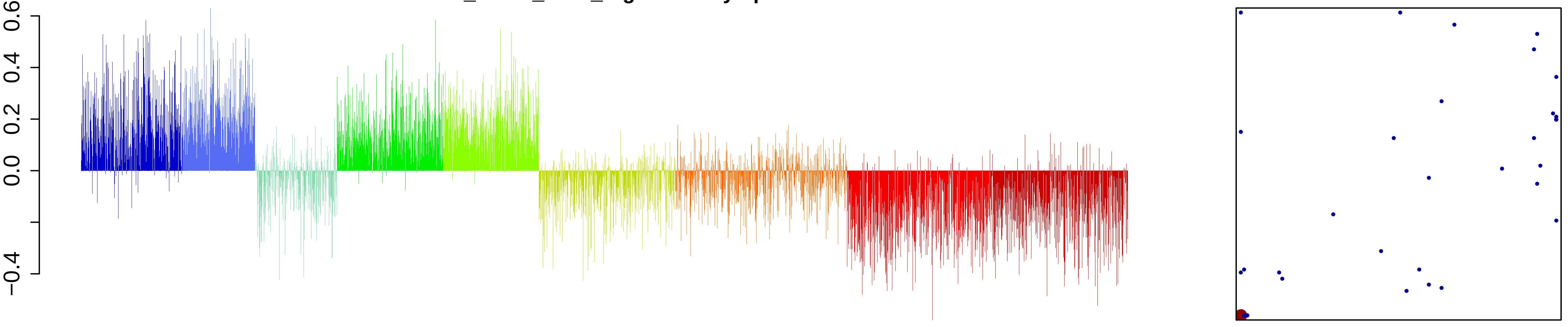

Altman_blood_M8.1_Cyclic AMP

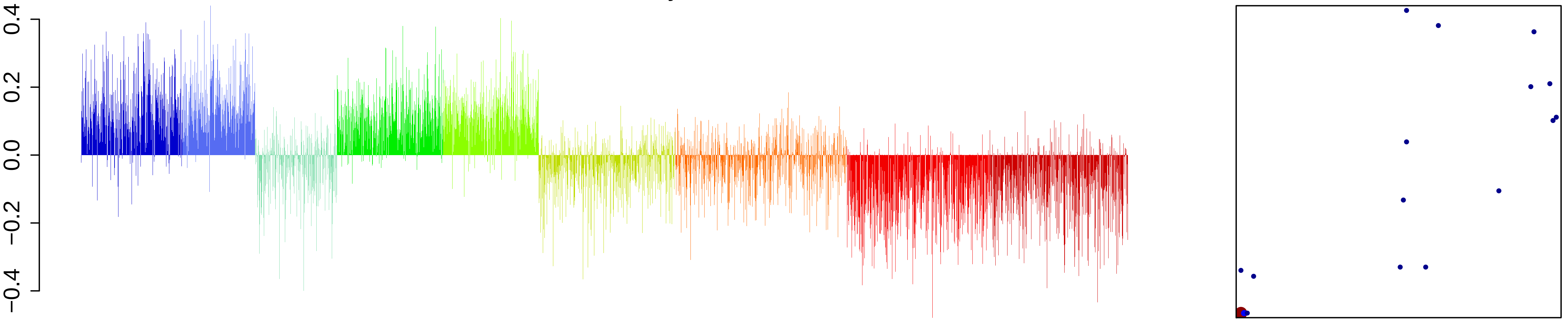

Altman_blood_M8.2_Integrin beta3

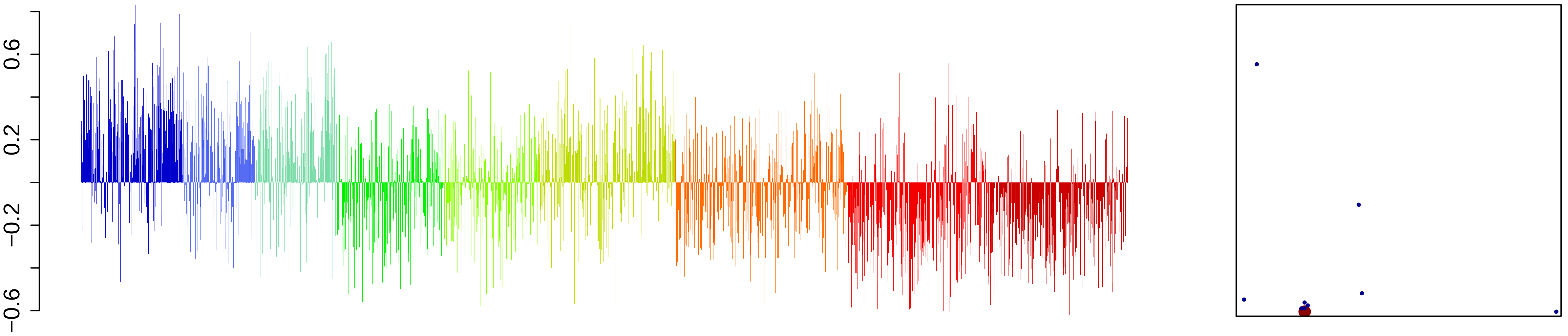

Altman_blood_M8.3_Transcriptome

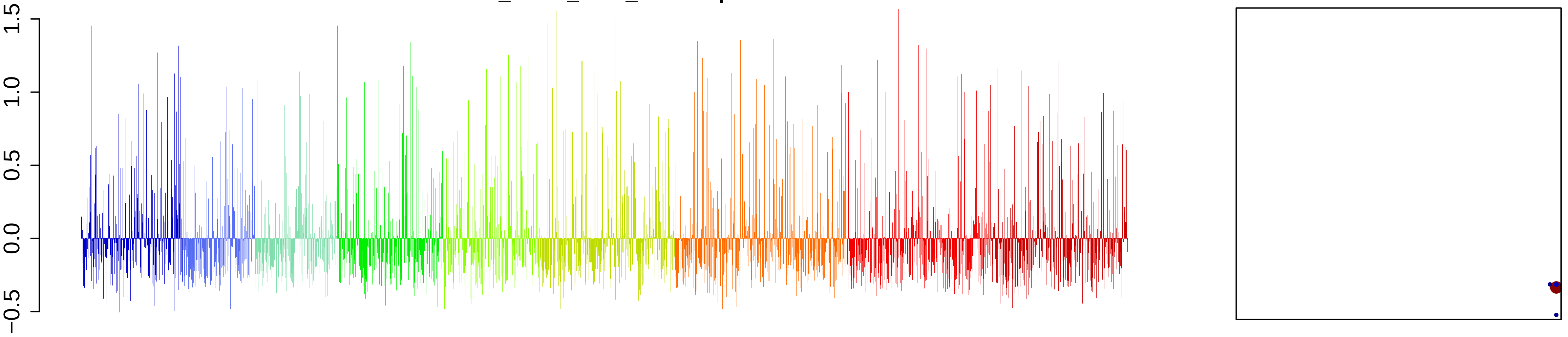

Altman_blood_M9.1_Cytotoxins

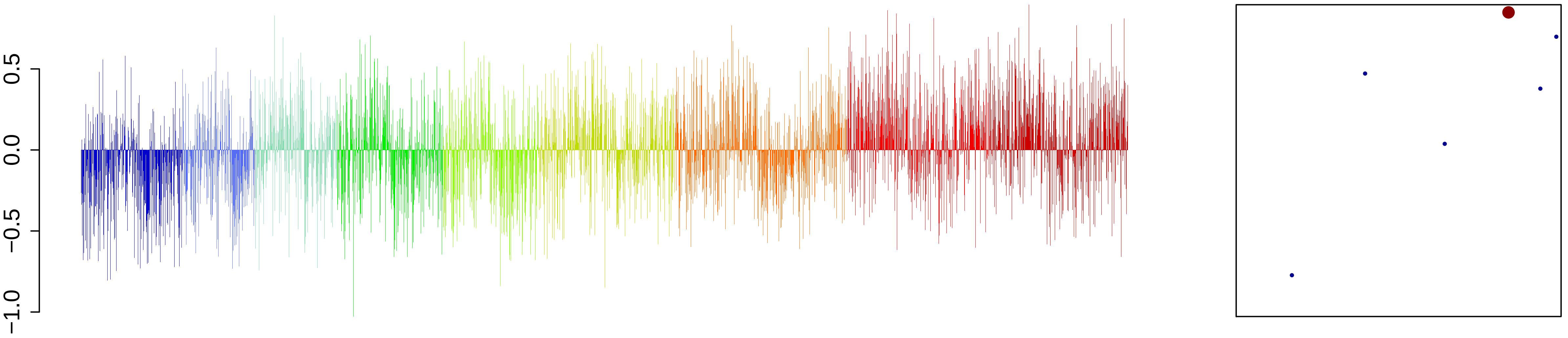

Altman_blood_M9.2_Erythrocyte Membrane

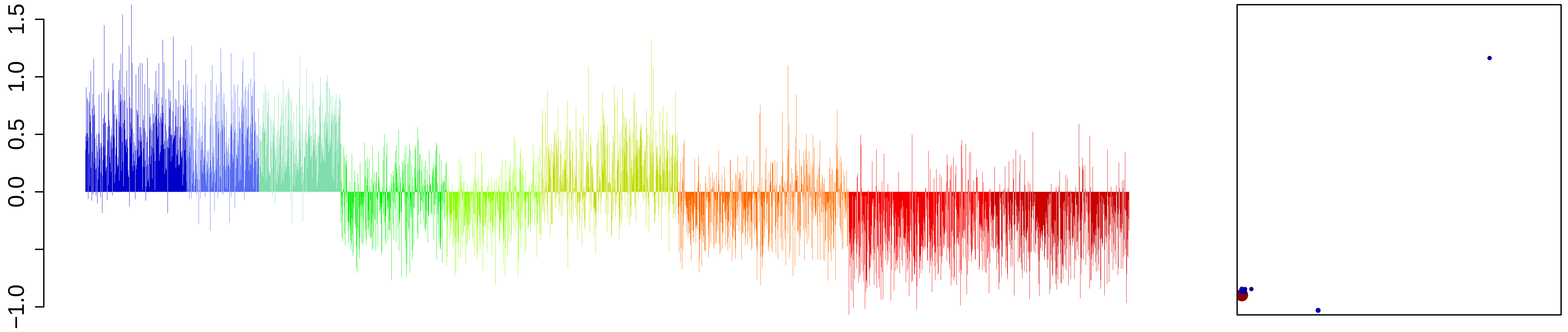

Altman_blood_M10.1_RIG−I

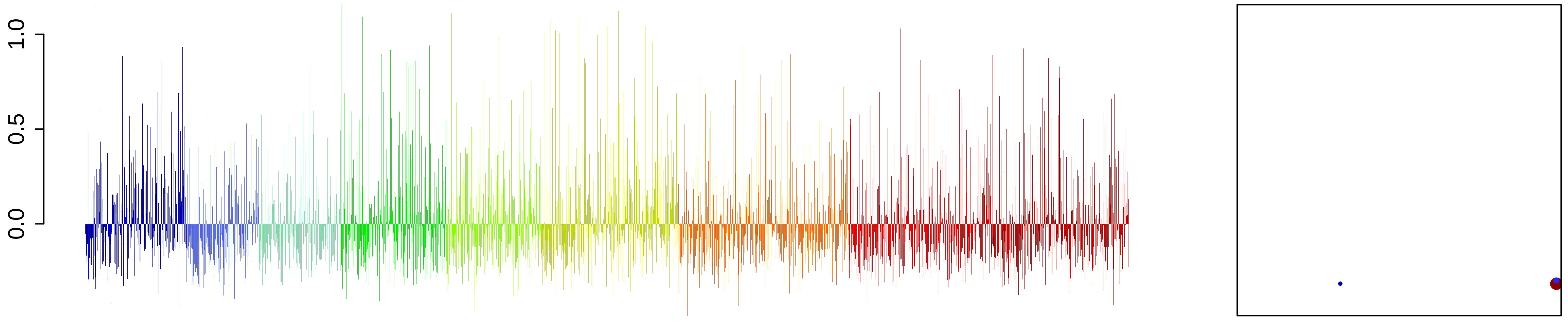

Altman_blood_M10.2_Hemoglobinopathies

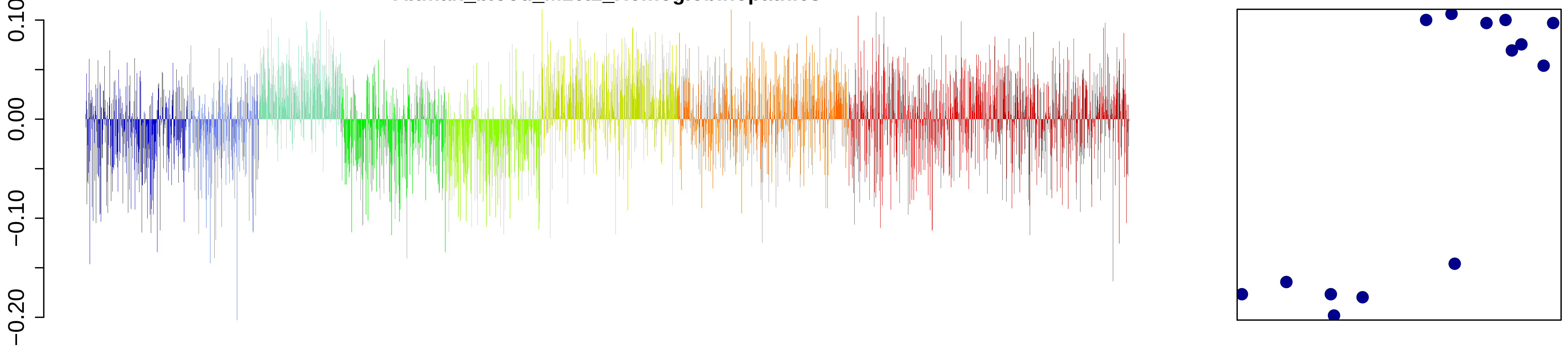

Altman_blood_M10.3_Adhesiveness

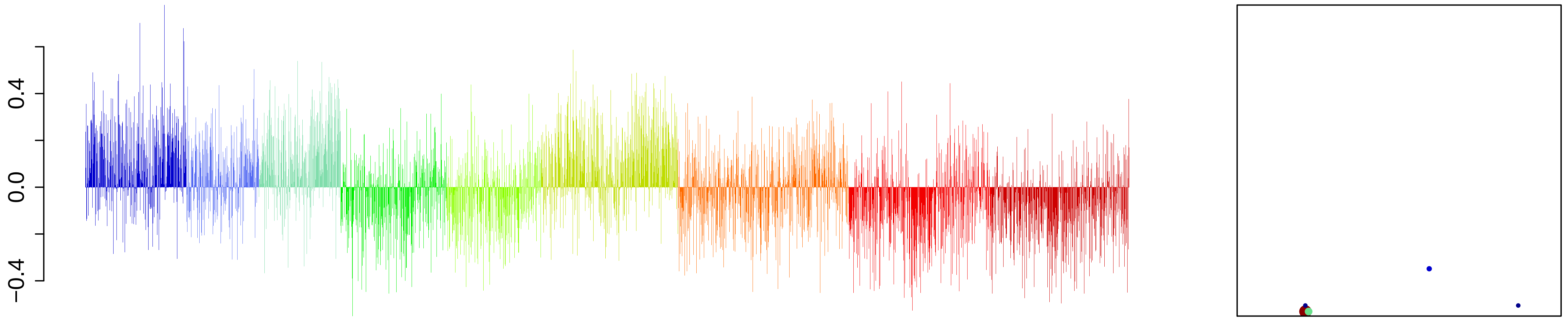

Altman_blood_M10.4_Cathepsin G

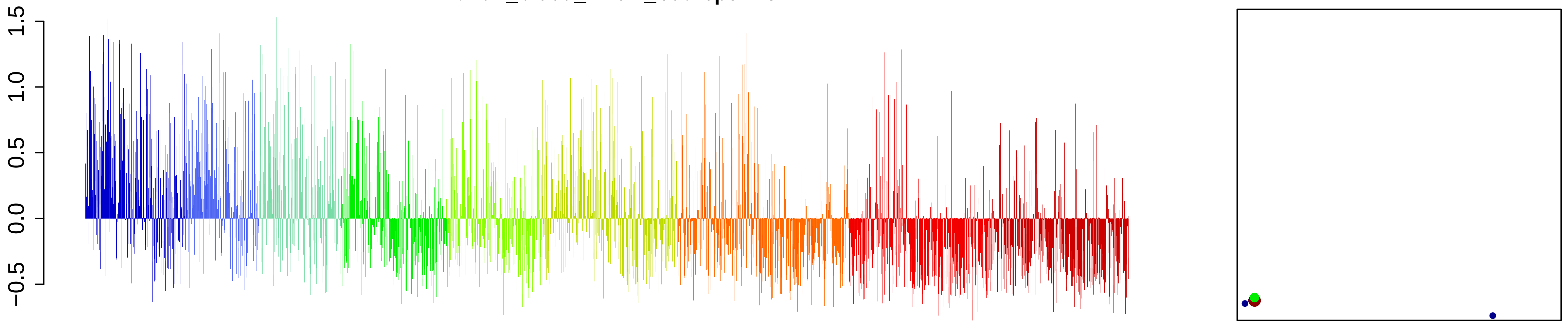

Altman_blood_M11.1_Chemical Phenomena

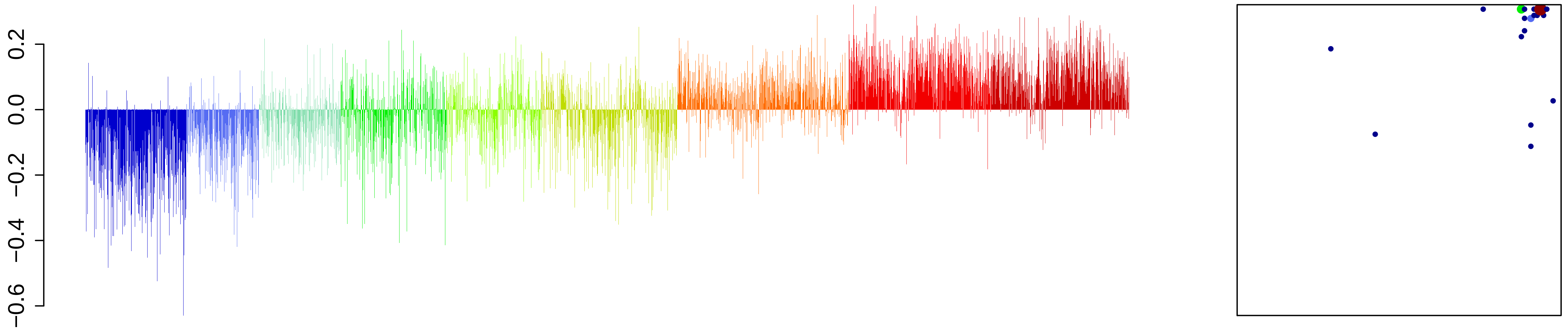

Altman_blood_M11.2_RNA Transport

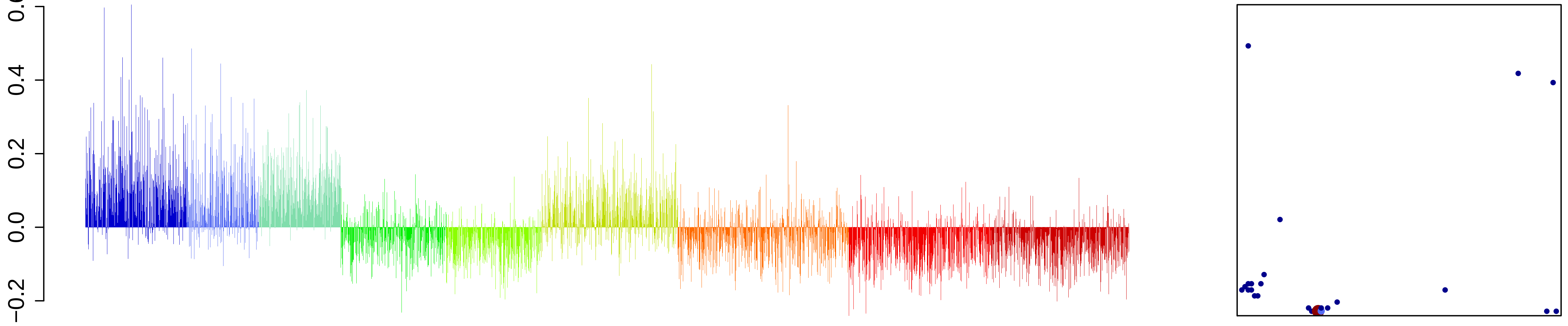

Altman_blood_M11.3_Erythroid Cells

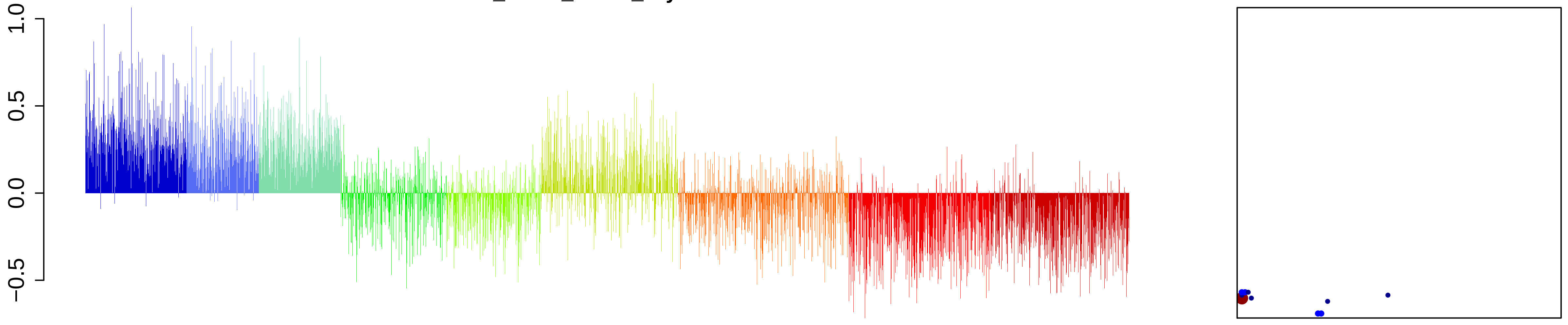

Altman_blood_M11.4_Metabolic Networks and Pathways

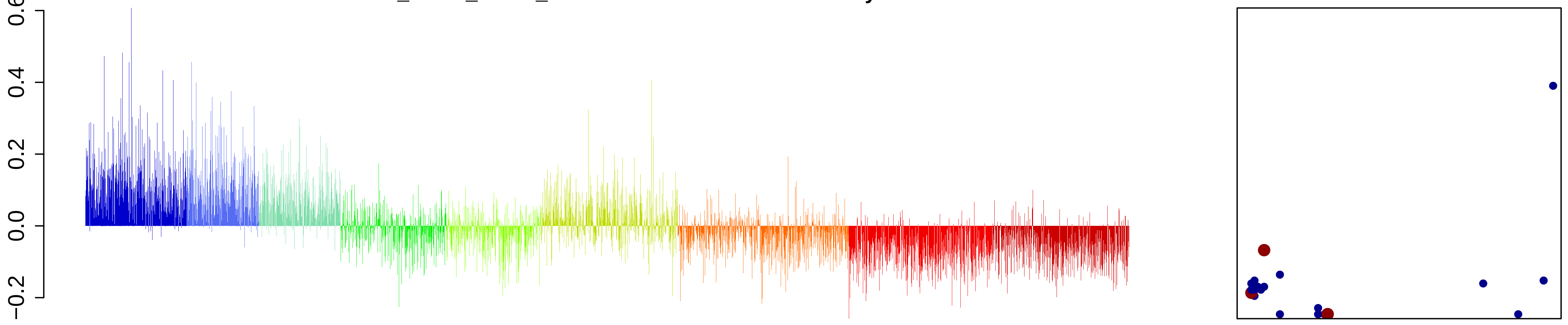

Altman_blood_M12.1_Mitochondria Apoptotic

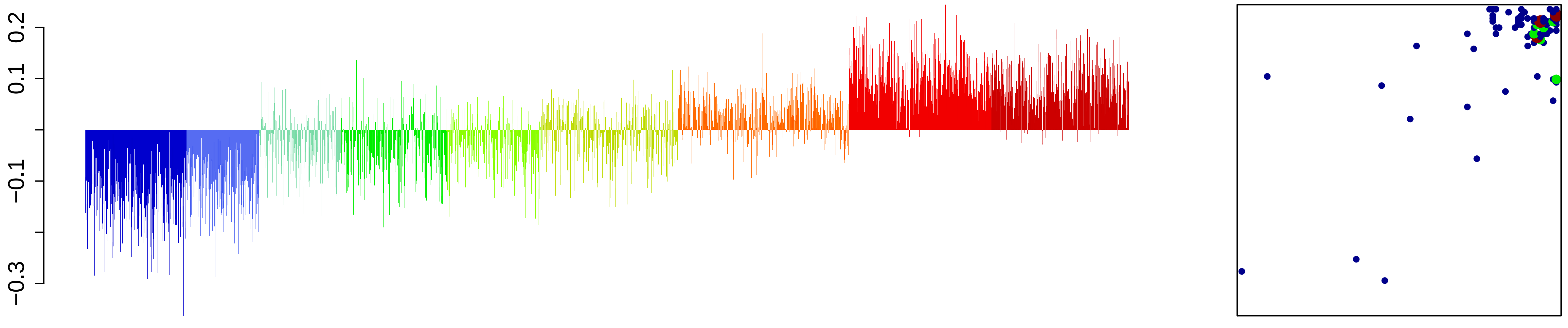

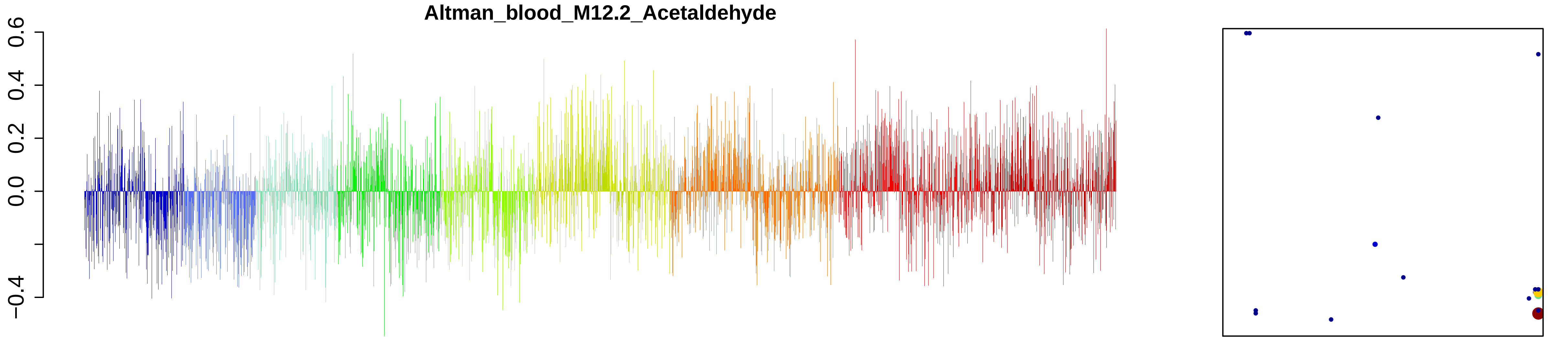
Altman_blood_M12.2_Acetaldehyde
0.6
0.4
0.2
0.0
−0.4

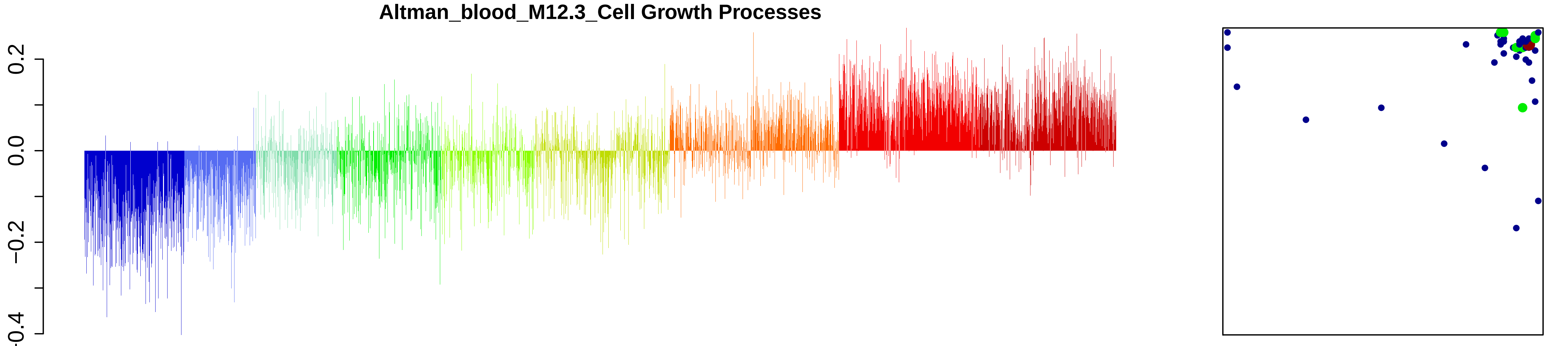
Altman_blood_M12.3_Cell Growth Processes
0.2
0.0
−0.2
−0.4

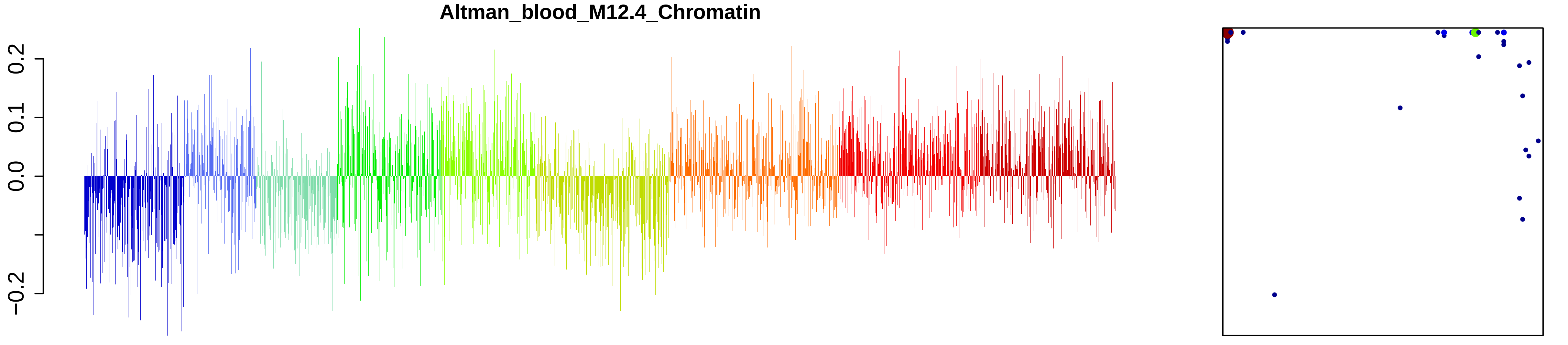
Altman_blood_M12.4_Chromatin
0.2
0.1
0.0
−0.2

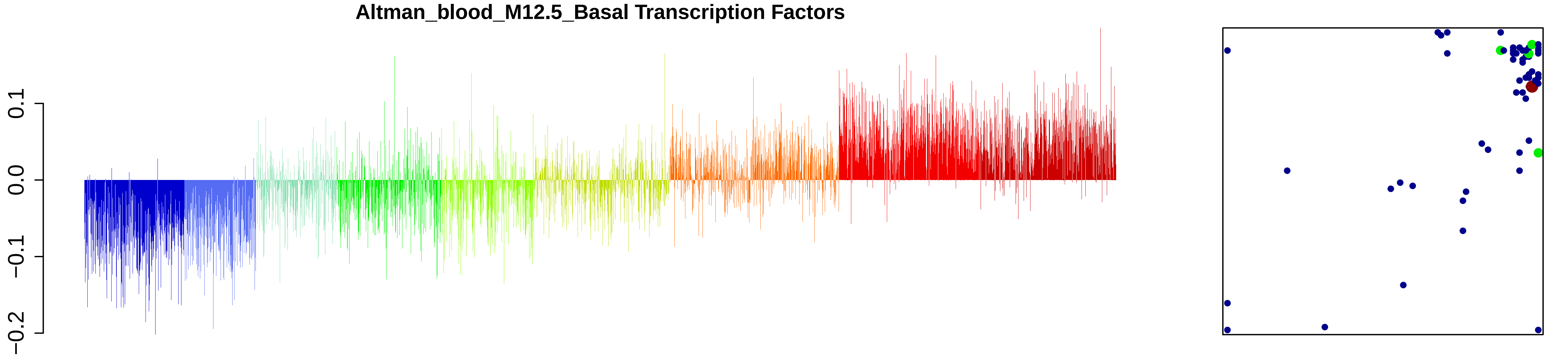
Altman_blood_M12.5_Basal Transcription Factors
0.1
0.0
−0.1
−0.2

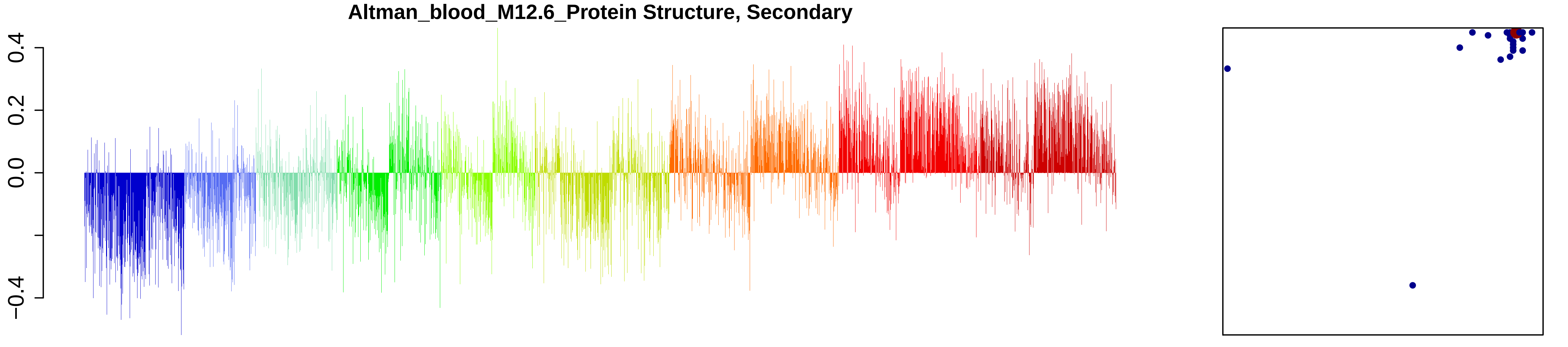
Altman_blood_M12.6_Protein Structure, Secondary
0.4
0.2
0.0
−0.4

Altman_blood_M12.7_Sequence Homology

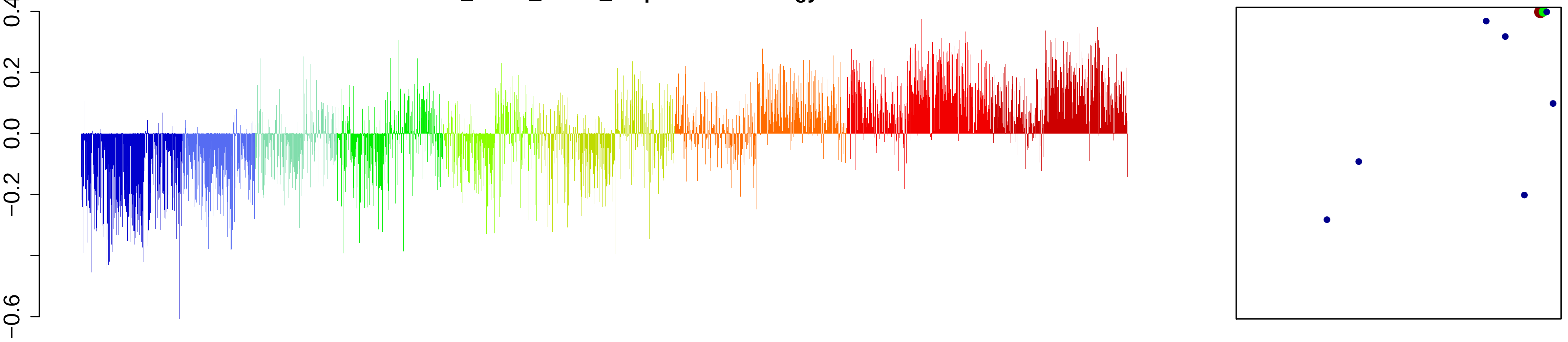

Altman_blood_M12.8_Antibodies, Monoclonal, Murine−Derived

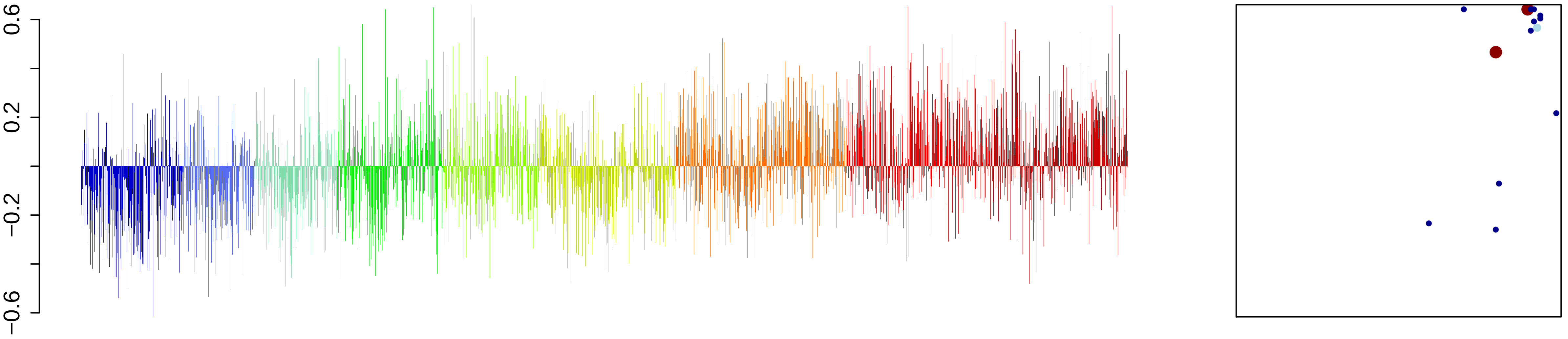

Altman_blood_M12.9_Cysteine

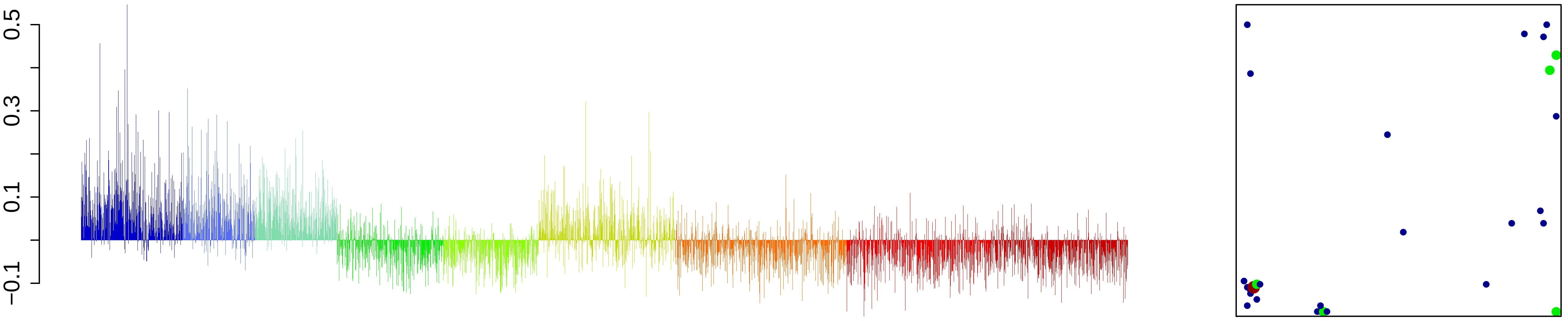

Altman_blood_M13.1_Mitogen−Activated Protein Kinase 1

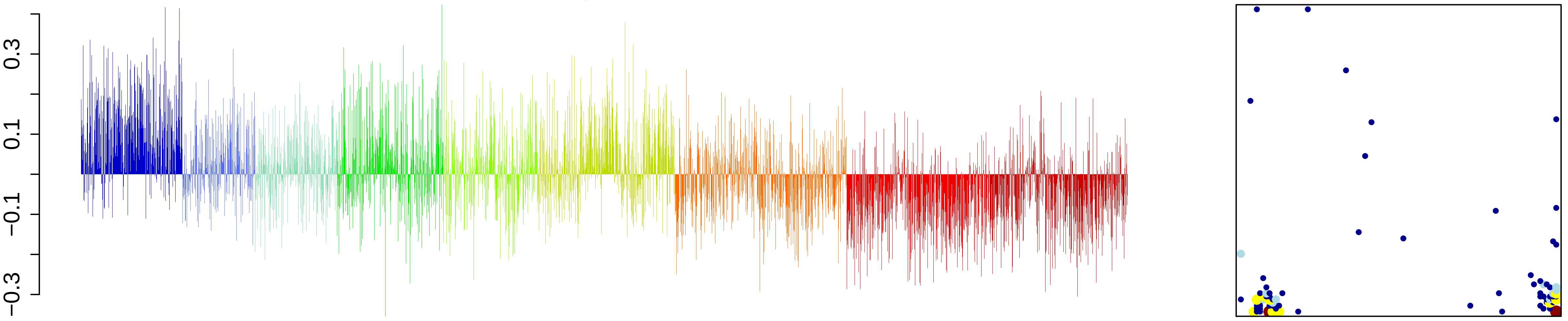

Altman_blood_M13.2_DNA

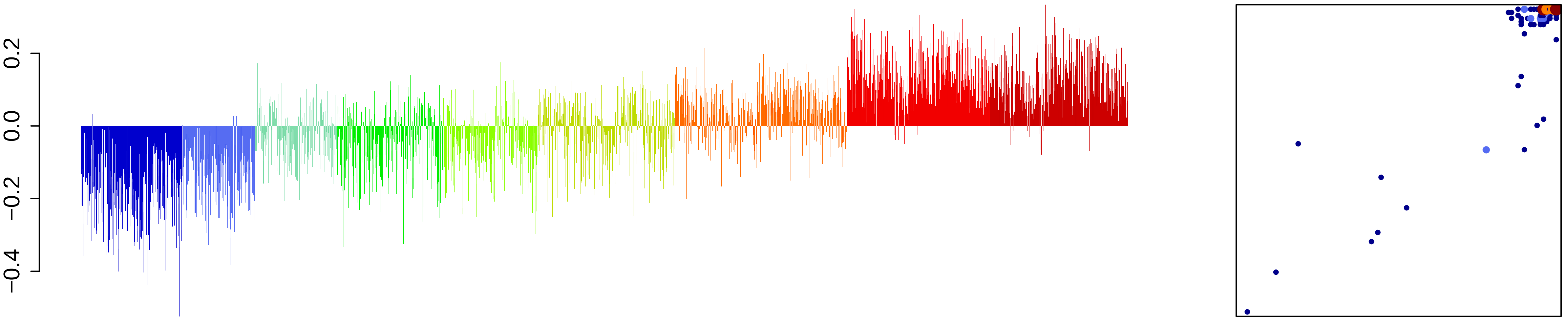

Altman_blood_M13.3_Arsenicals

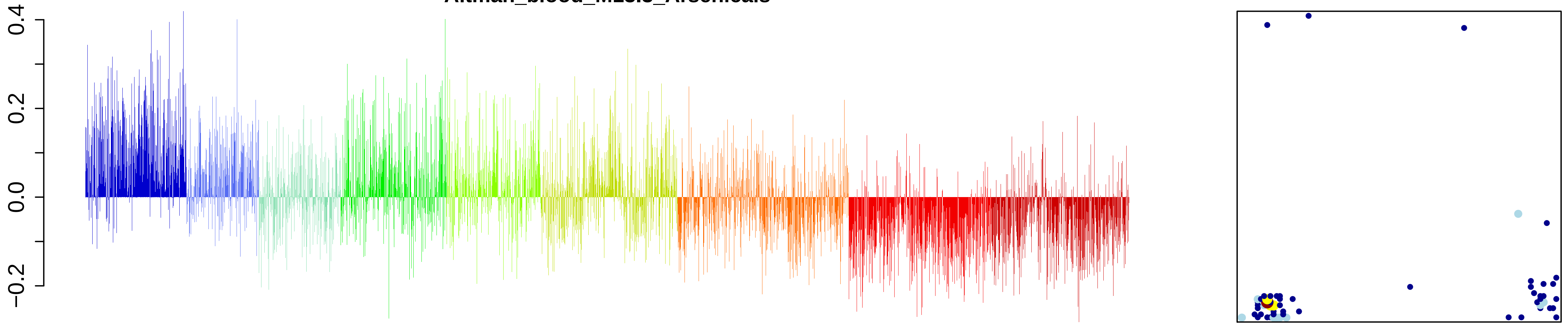

Altman_blood_M13.4_Carcinoma, Hepatocellular

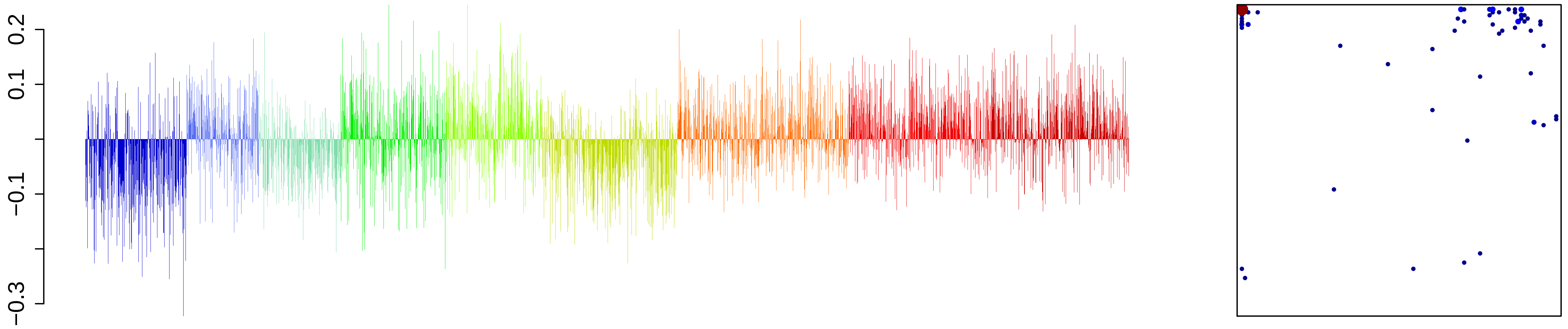

Altman_blood_M13.5_beta−Galactosidase

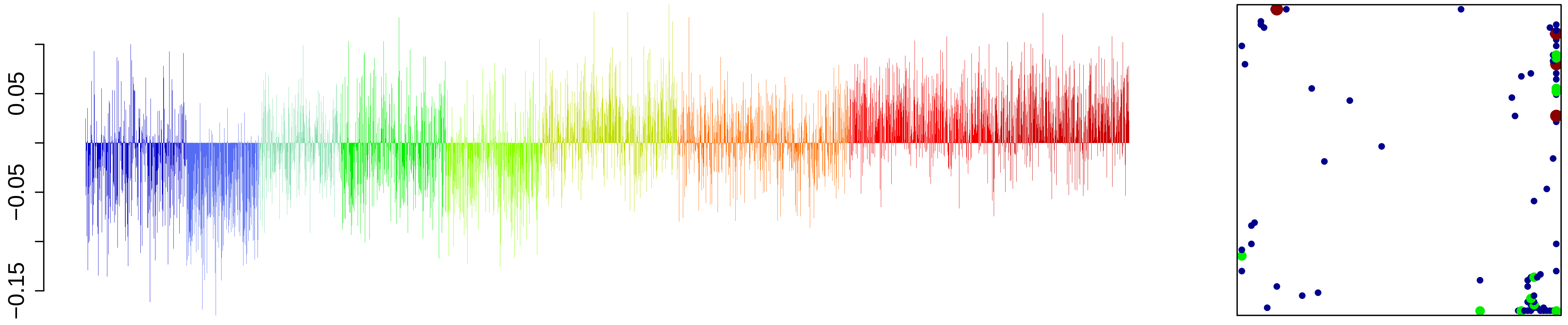

Altman_blood_M13.6_Apoptosis

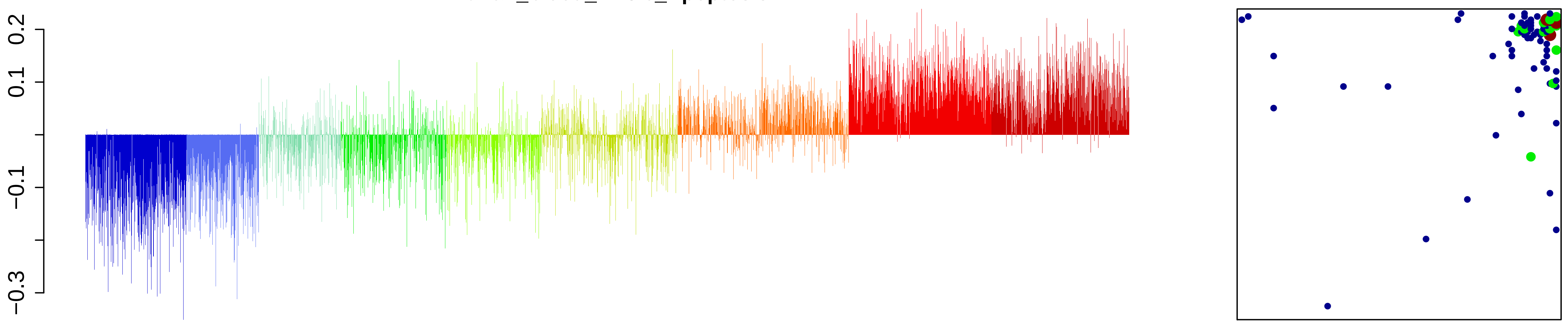

Altman_blood_M13.7_RNA Interference

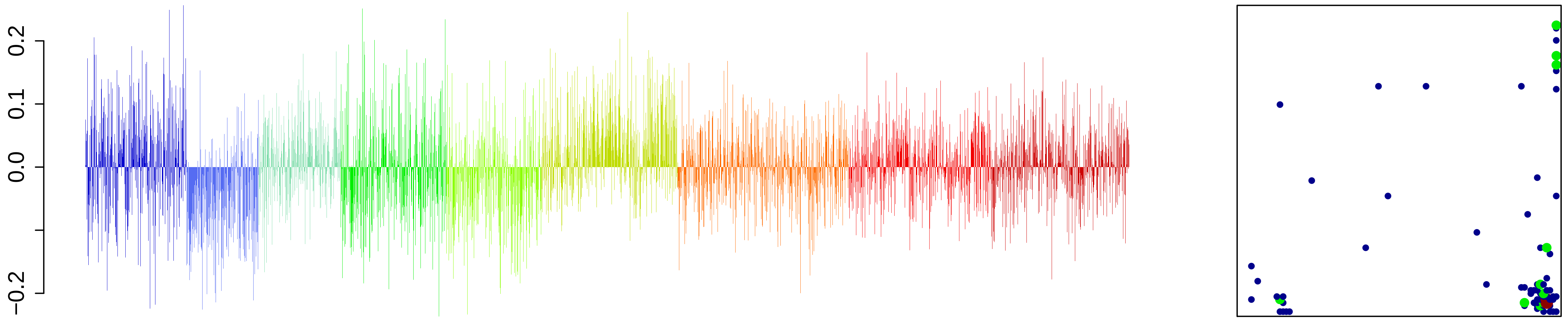

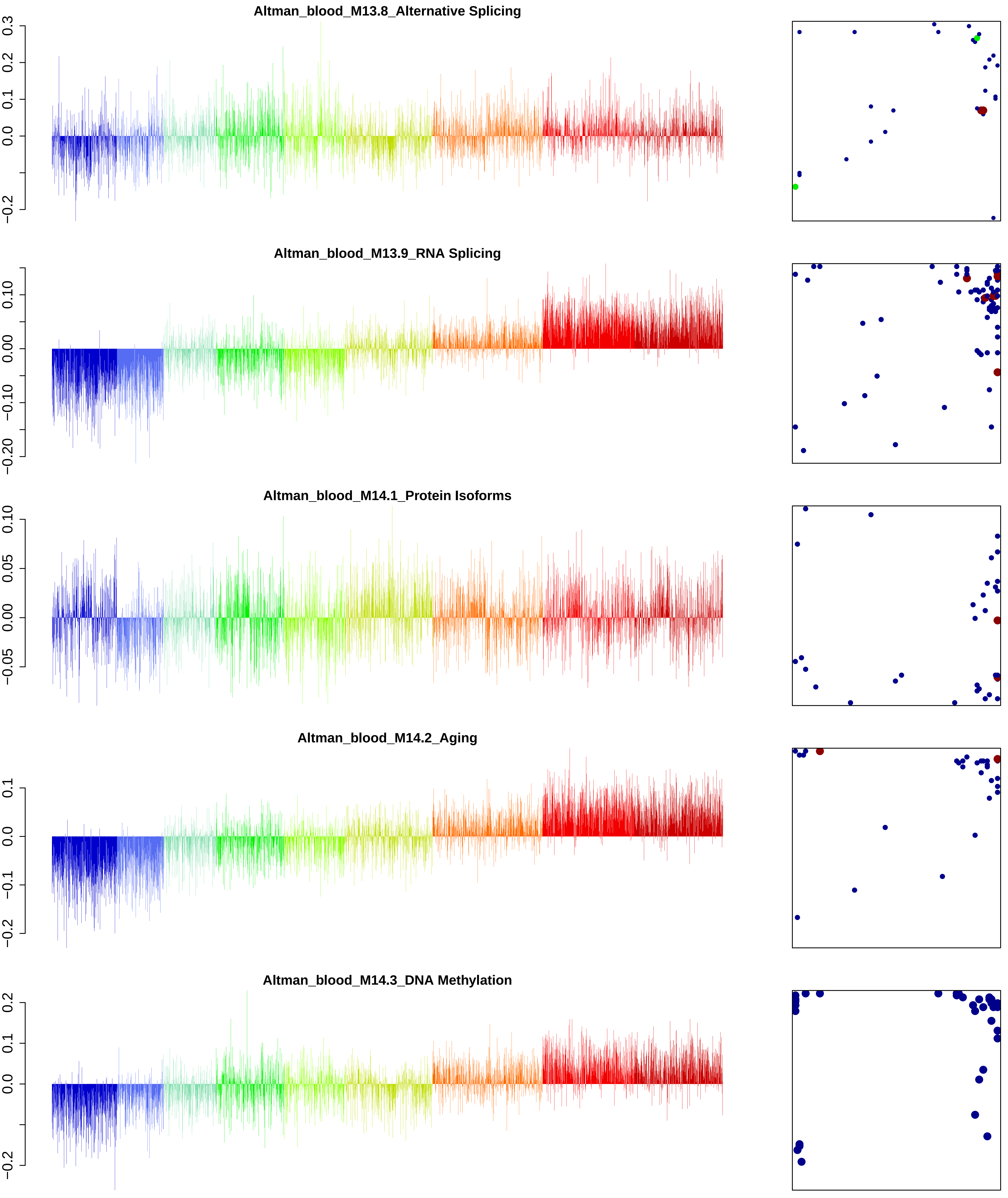
Altman_blood_M13.8_Alternative Splicing
0.3
0.2
0.1
0.0
−0.2
Altman_blood_M13.9_RNA Splicing
0.10
0.00
−0.10
−0.20
Altman_blood_M14.1_Protein Isoforms
0.10
0.05
0.00
−0.05
Altman_blood_M14.2_Aging
0.1
0.0
−0.1
−0.2
Altman_blood_M14.3_DNA Methylation
0.2
0.1
0.0
−0.2

Altman_blood_M14.4_Uracil

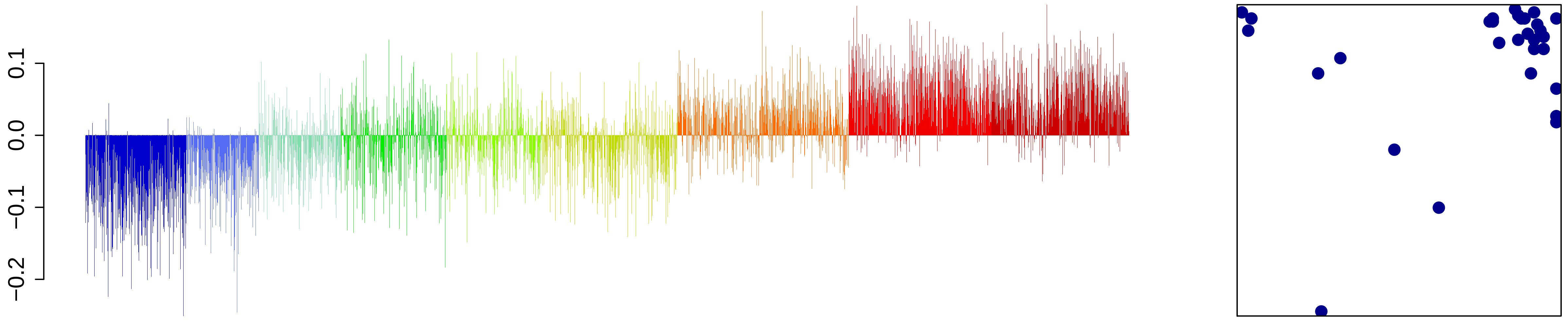

Altman_blood_M14.5_Phylogeny

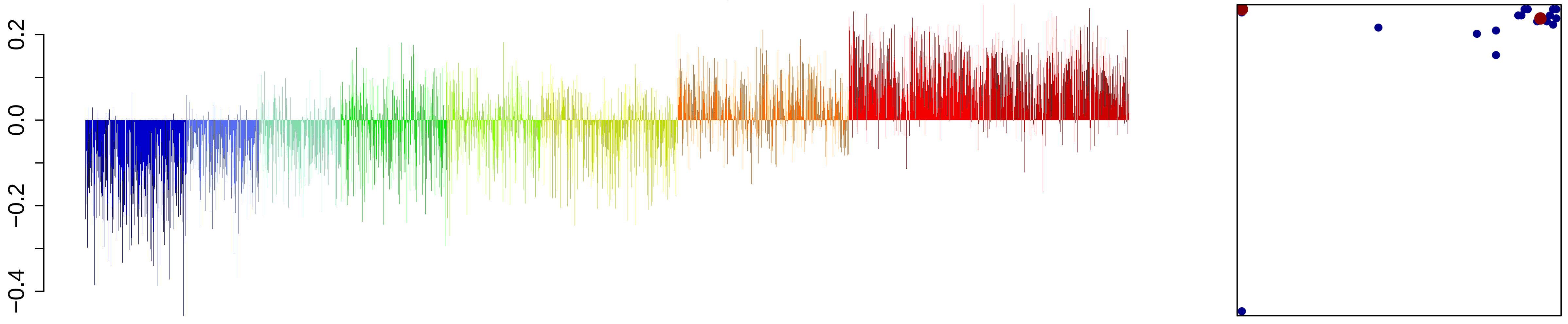

Altman_blood_M14.6_Protein Synthesis Inhibitors

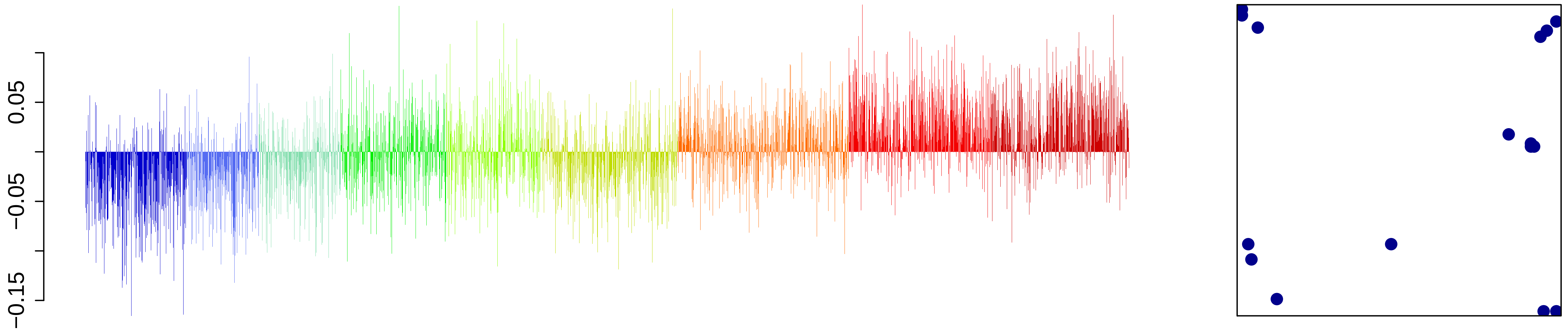

Altman_blood_M14.7_Amino Acid Substitution

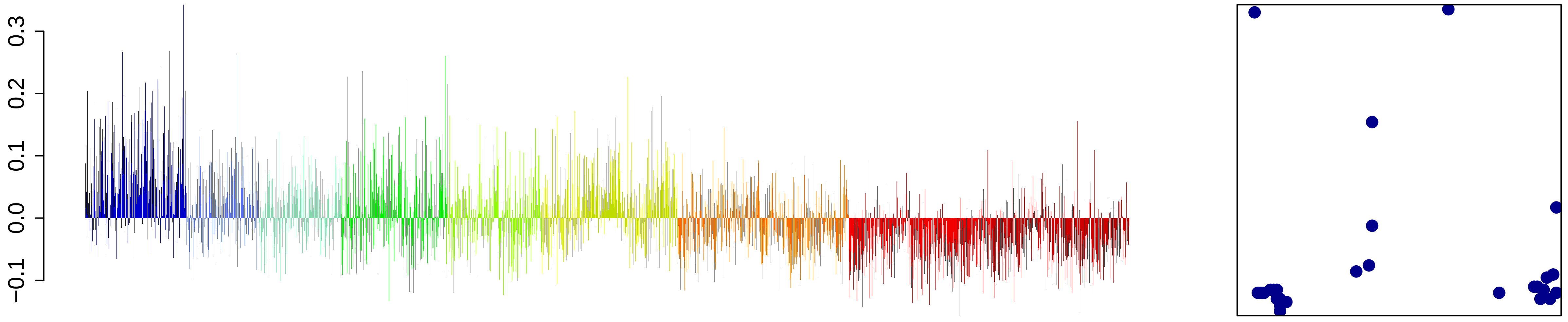

Altman_blood_M14.8_Flavoproteins

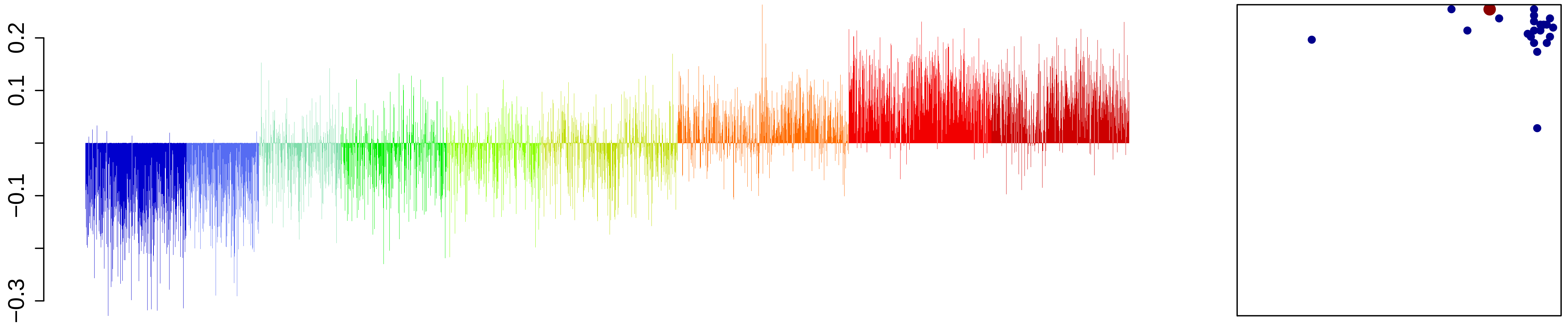

Altman_blood_M14.9_Colorectal Neoplasms

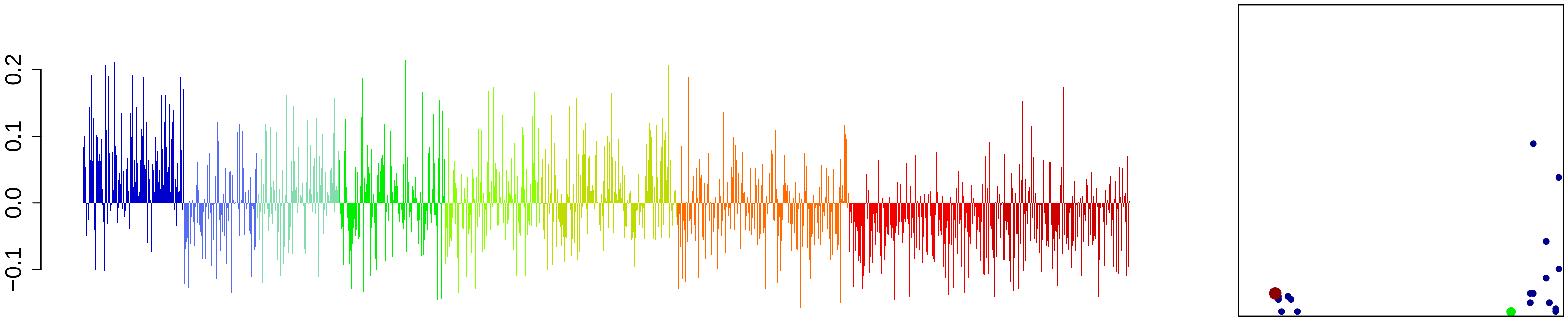

Altman_blood_M15.1_Genomic Instability

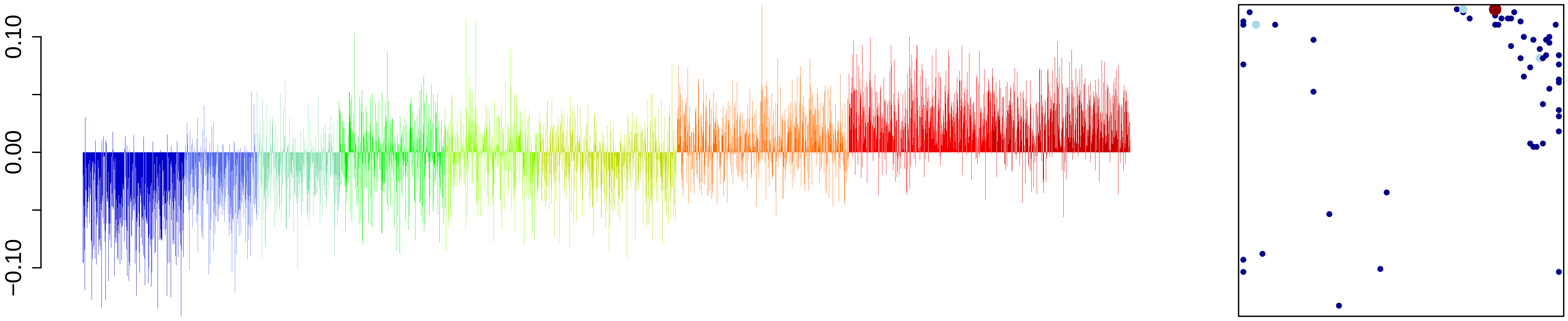

Altman_blood_M15.2_Neuroectodermal Tumors, Primitive

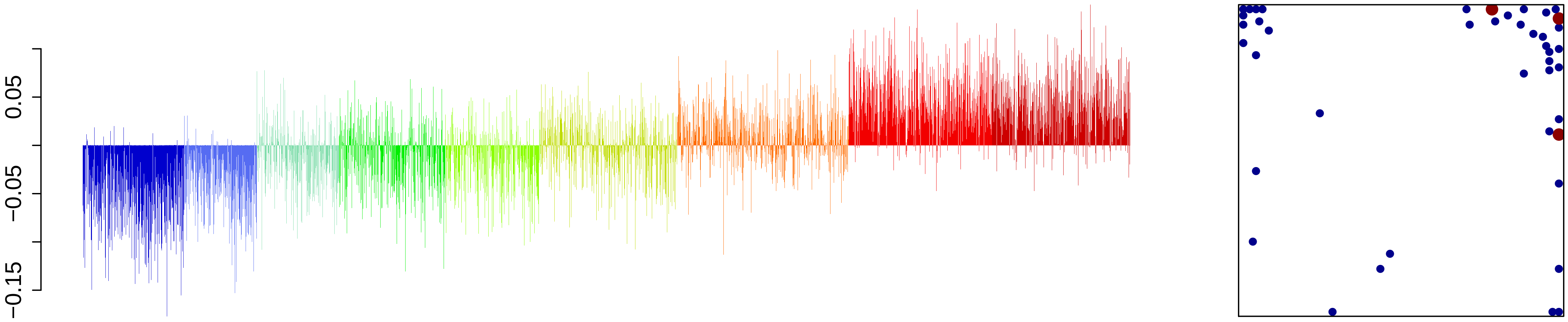

Altman_blood_M16.6_Alleles

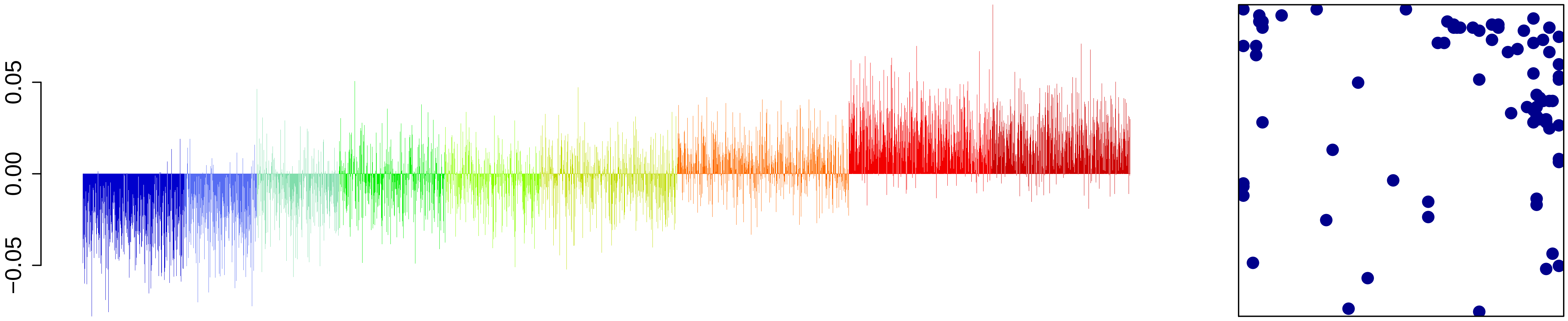

Altman_blood_M16.7_Embryo, Mammalian

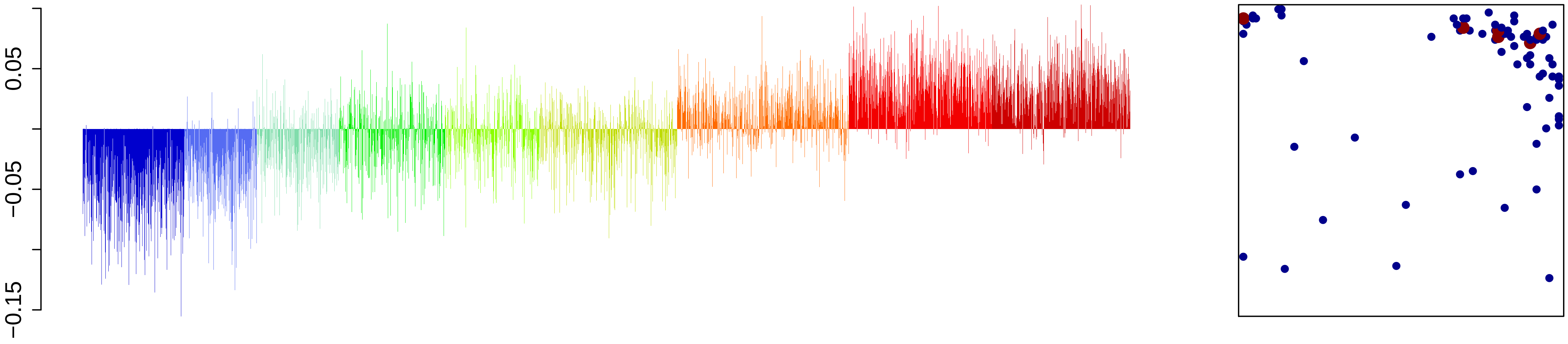

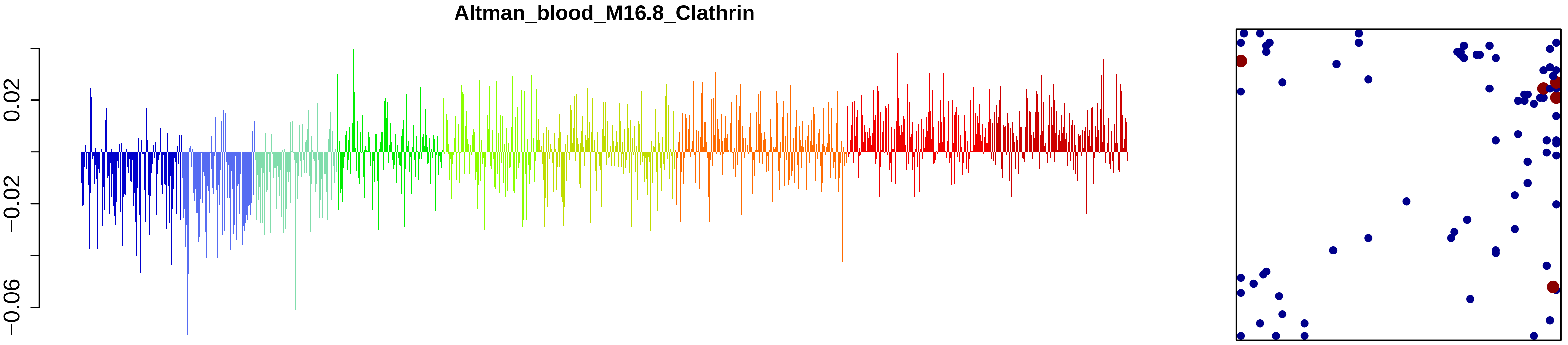
Altman_blood_M16.8_Clathrin
0.02
−0.02
−0.06

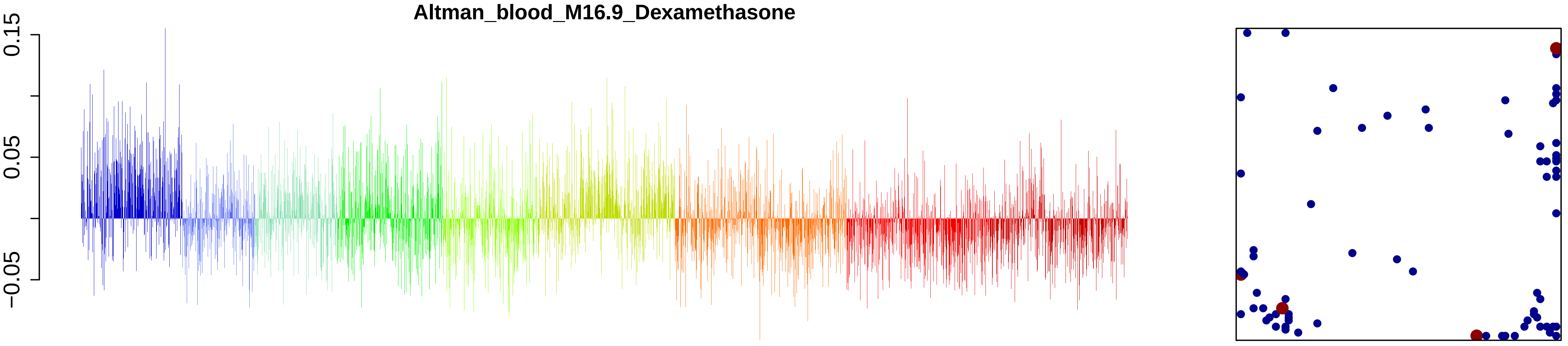
Altman_blood_M16.9_Dexamethasone
0.15
0.05
−0.05

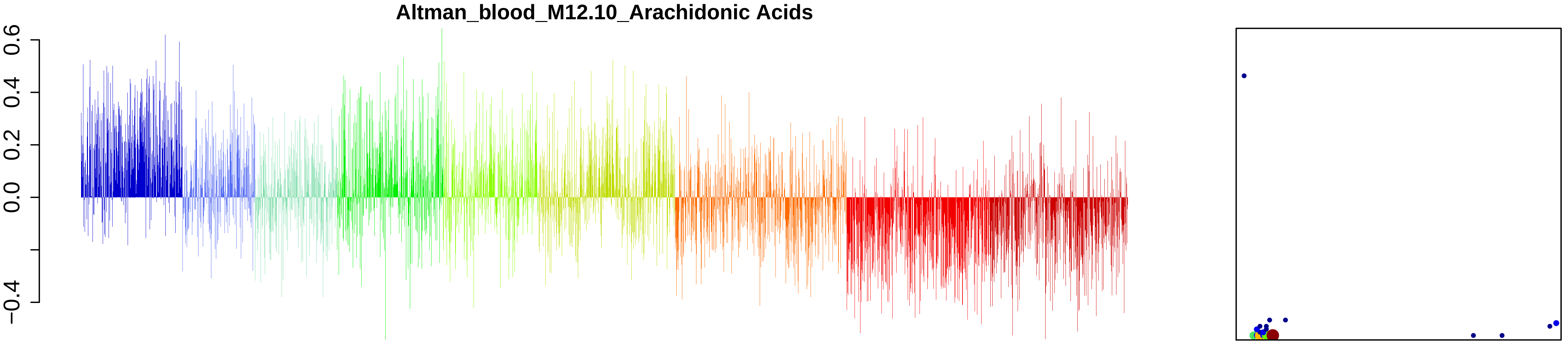
Altman_blood_M12.10_Arachidonic Acids
0.6
0.4
0.2
0.0
−0.4

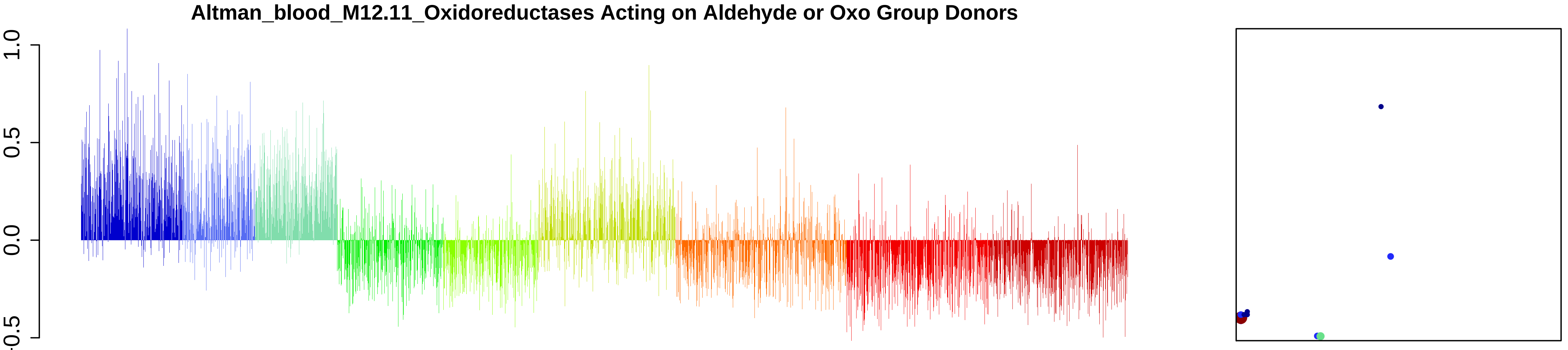
Altman_blood_M12.11_Oxidoreductases Acting on Aldehyde or Oxo Group Donors
1.0
0.5
0.0
−0.5

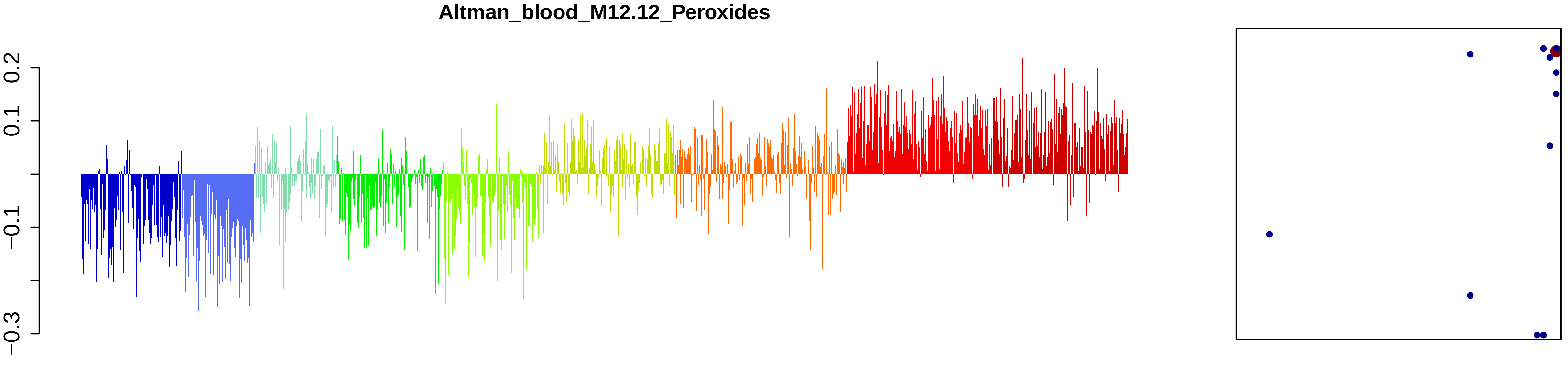
Altman_blood_M12.12_Peroxides
0.2
0.1
−0.1
−0.3

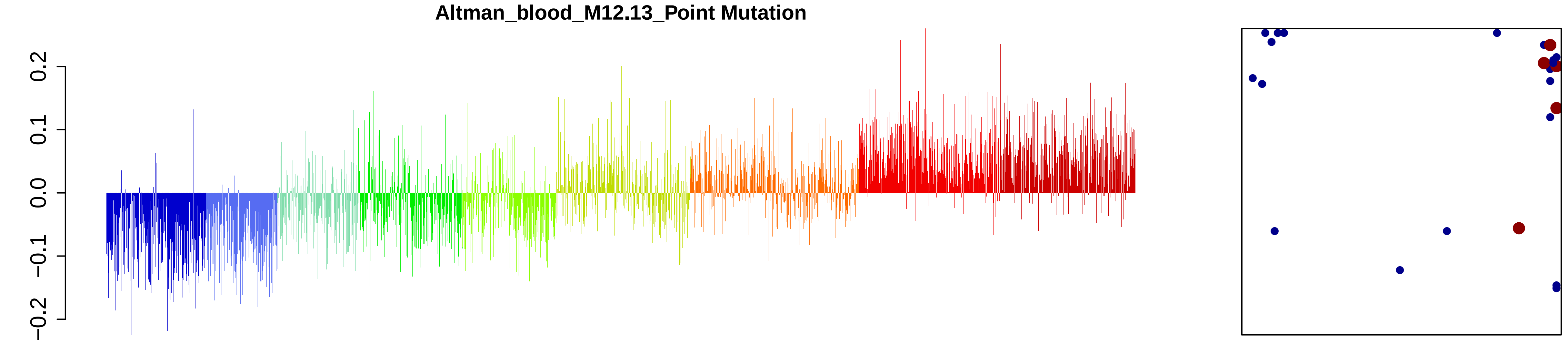
Altman_blood_M12.13_Point Mutation
0.2
0.1
0.0
−0.1
−0.2

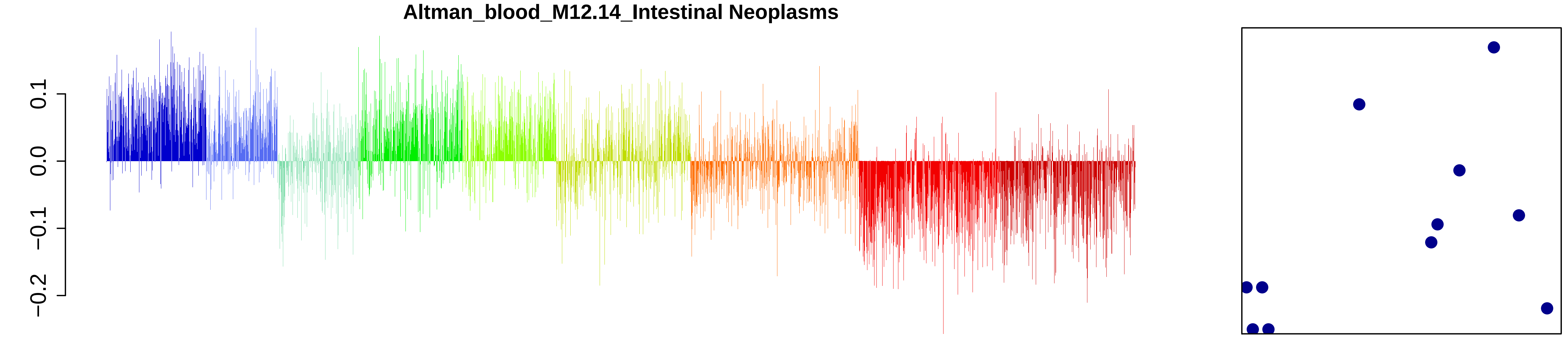
Altman_blood_M12.14_Intestinal Neoplasms
0.1
0.0
−0.1
−0.2

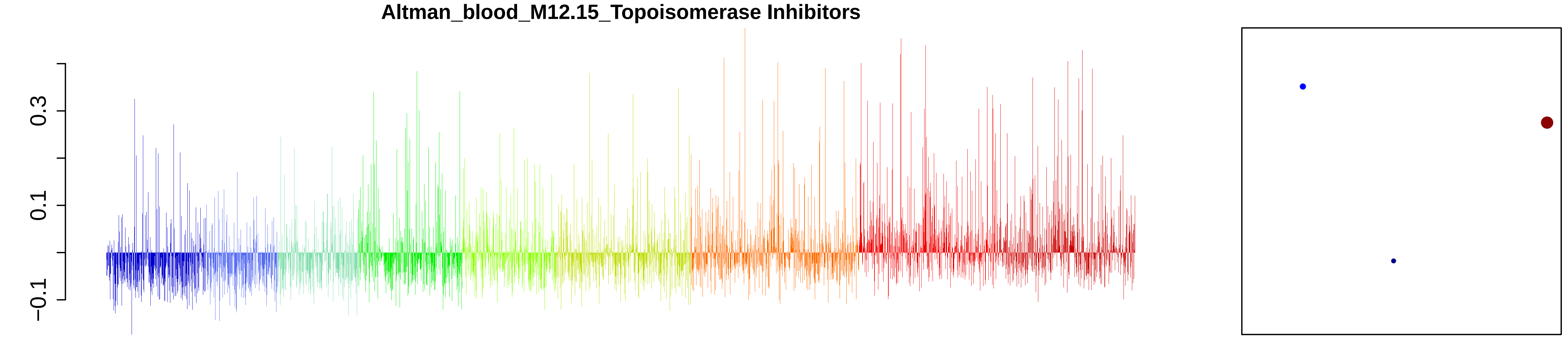
Altman_blood_M12.15_Topoisomerase Inhibitors
0.3
0.1
−0.1

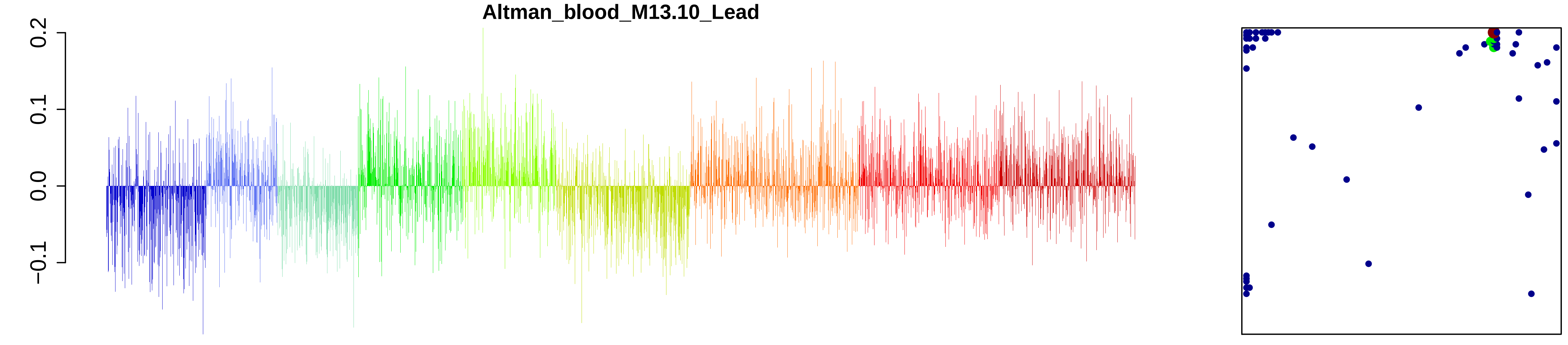
Altman_blood_M13.10_Lead
0.2
0.1
0.0
−0.1

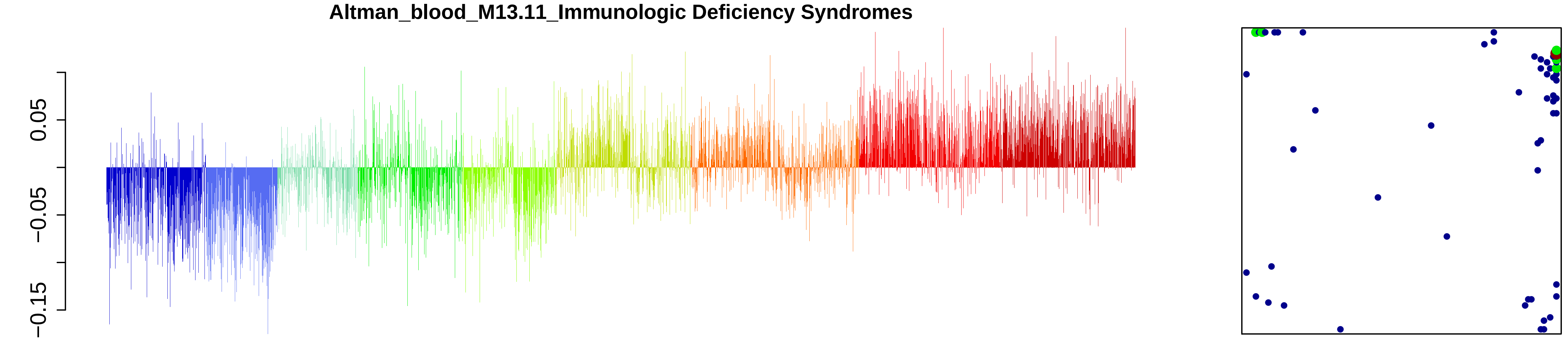
Altman_blood_M13.11_Immunologic Deficiency Syndromes
0.05
−0.05
−0.15

Altman_blood_M13.12_Receptors, Interleukin−18

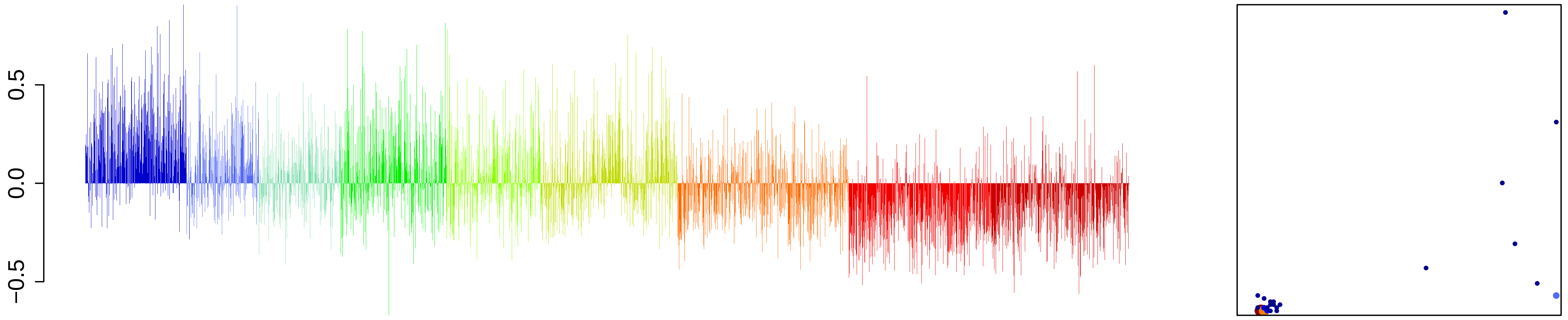

Altman_blood_M13.13_Heterogeneous−Nuclear Ribonucleoproteins

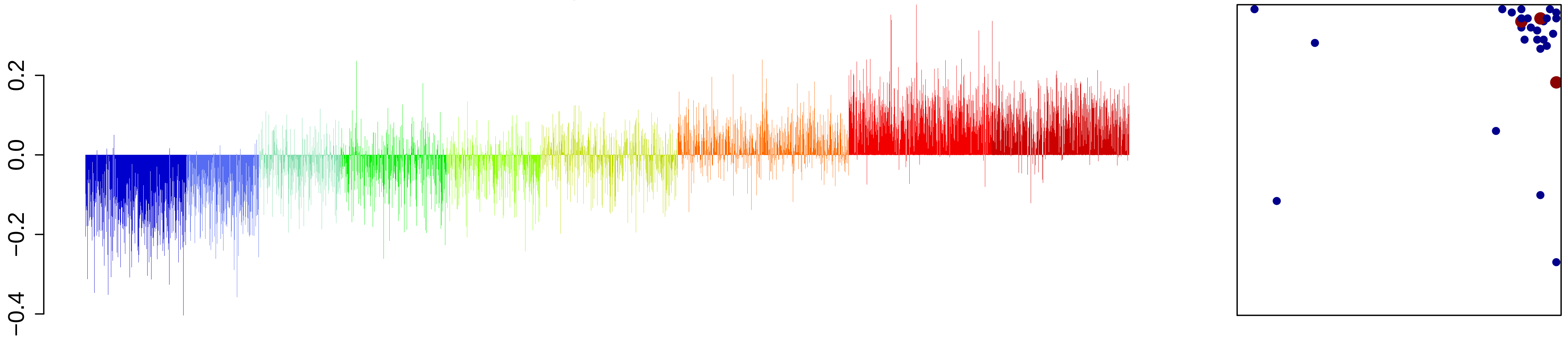

Altman_blood_M13.14_Piperidones

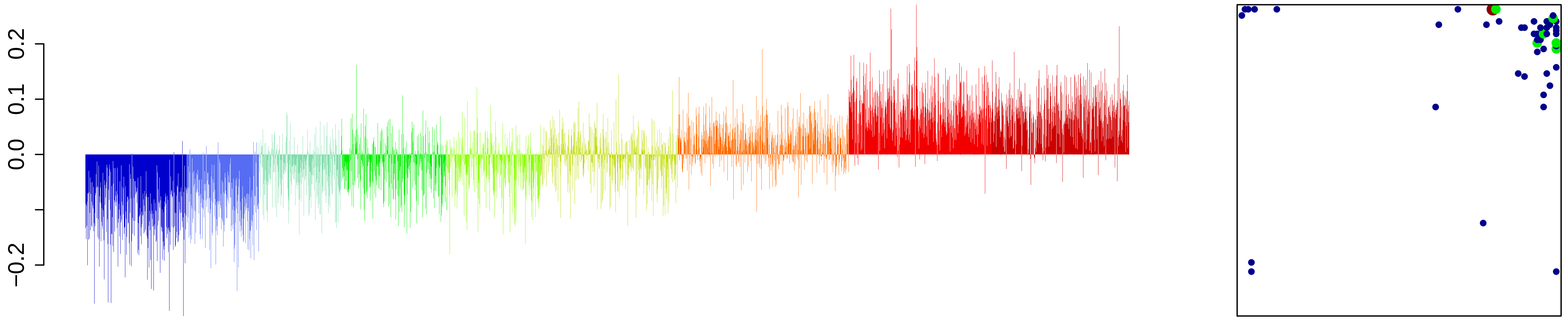

Altman_blood_M13.15_Protein Domains

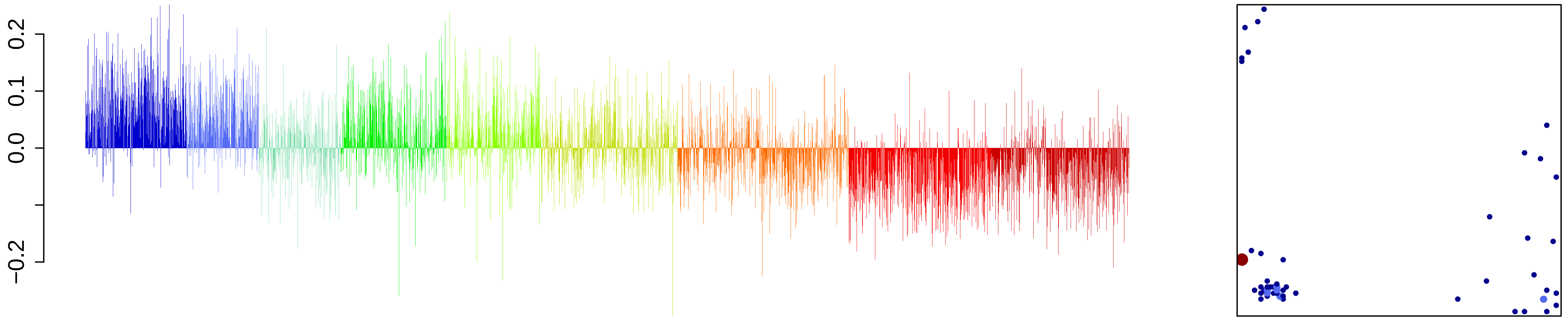

Altman_blood_M13.16_Cryoprotective Agents

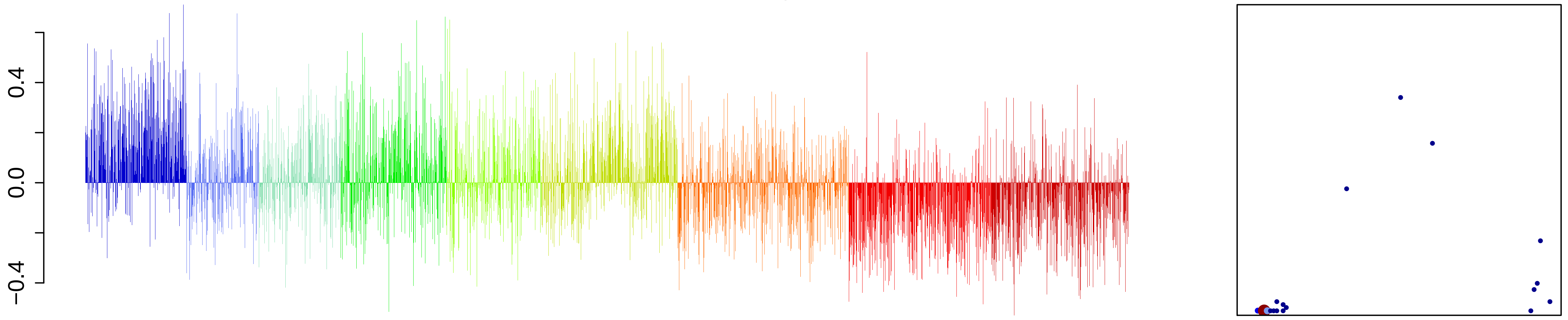

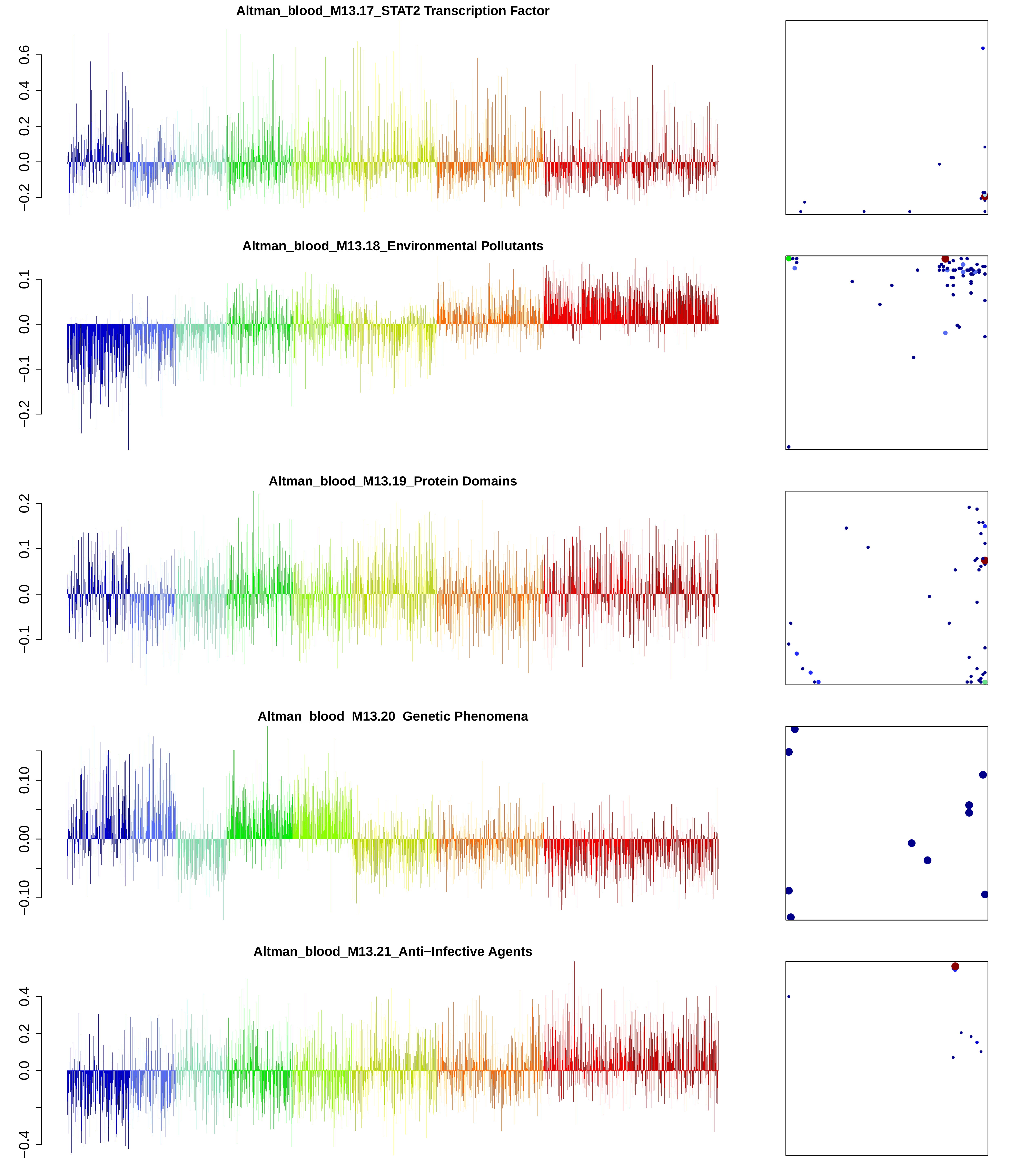
Altman_blood_M13.17_STAT2 Transcription Factor
−0.2
0.0
0.2
0.4
0.6
Altman_blood_M13.18_Environmental Pollutants
−0.2
−0.1
0.0
0.1
Altman_blood_M13.19_Protein Domains
−0.1
0.0
0.1
0.2
Altman_blood_M13.20_Genetic Phenomena
−0.10
0.00
0.10
Altman_blood_M13.21_Anti−Infective Agents
−0.4
0.0
0.2
0.4

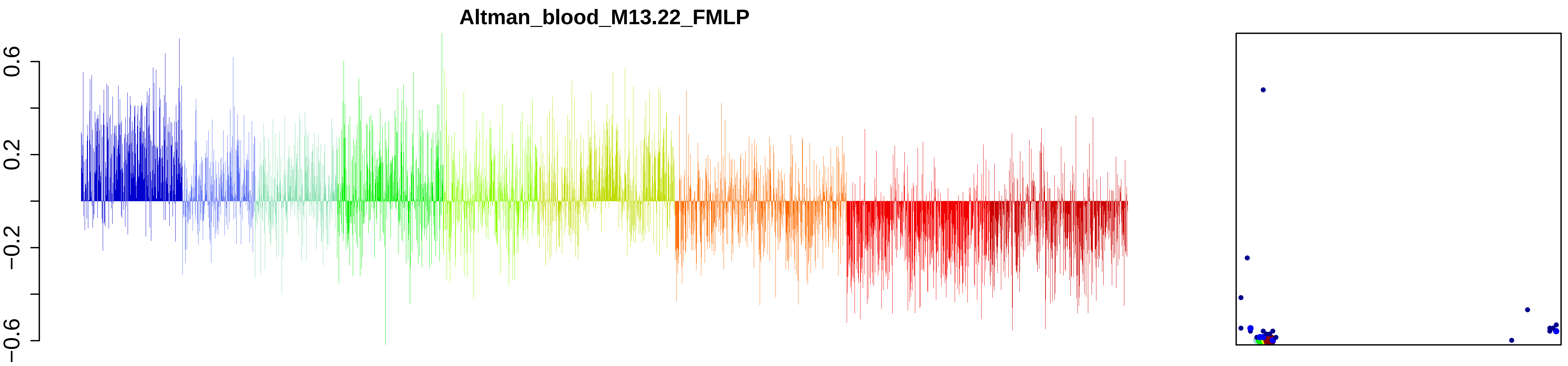
Altman_blood_M13.22_FMLP
0.6
0.2
−0.2
−0.6

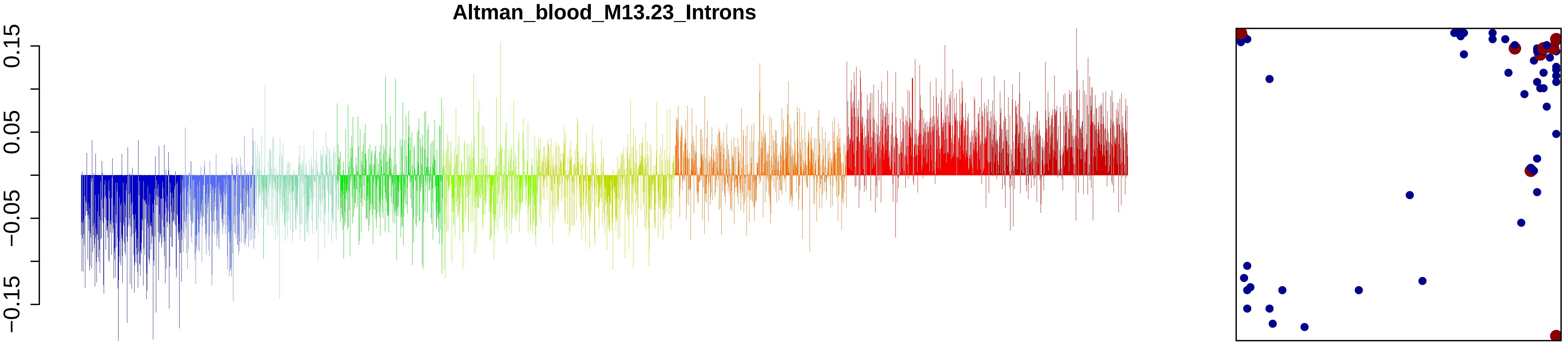
Altman_blood_M13.23_Introns
0.15
0.05
−0.05
−0.15

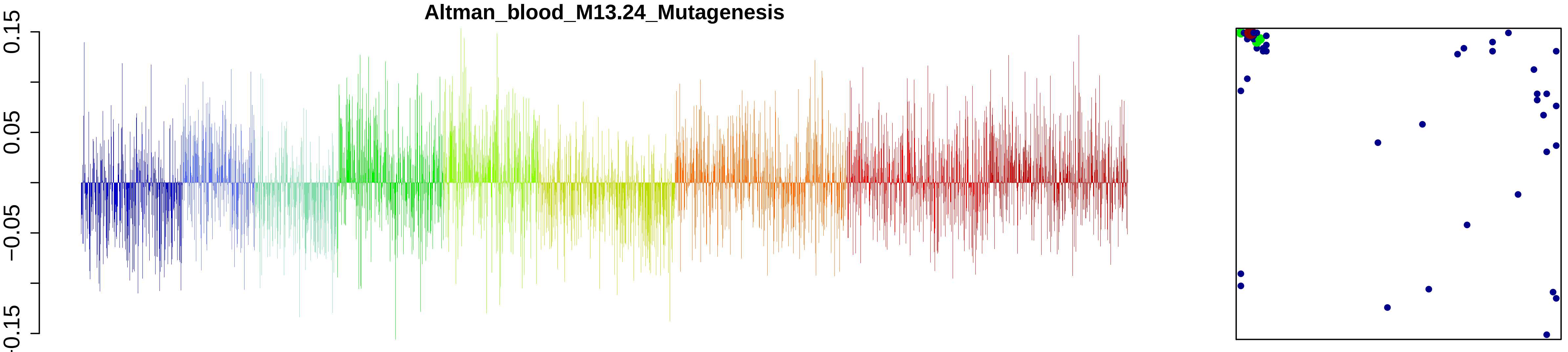
Altman_blood_M13.24_Mutagenesis
0.15
0.05
−0.05
−0.15

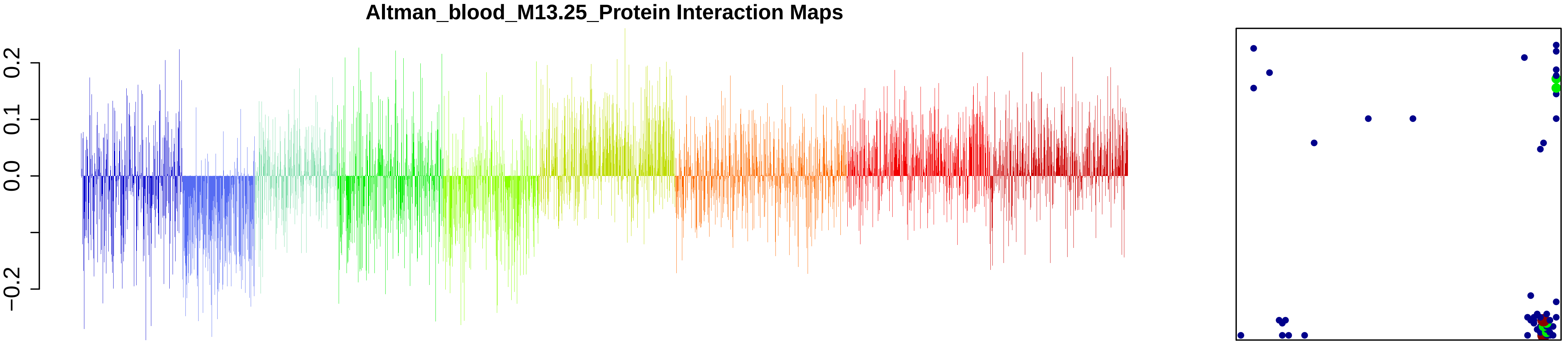
Altman_blood_M13.25_Protein Interaction Maps
0.2
0.1
0.0
−0.2

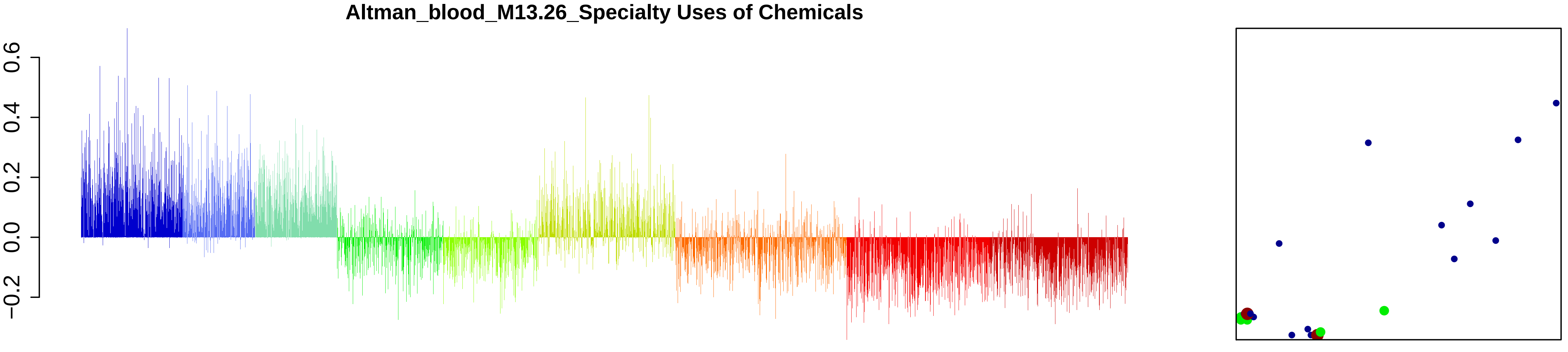
Altman_blood_M13.26_Specialty Uses of Chemicals
0.6
0.4
0.2
0.0
−0.2

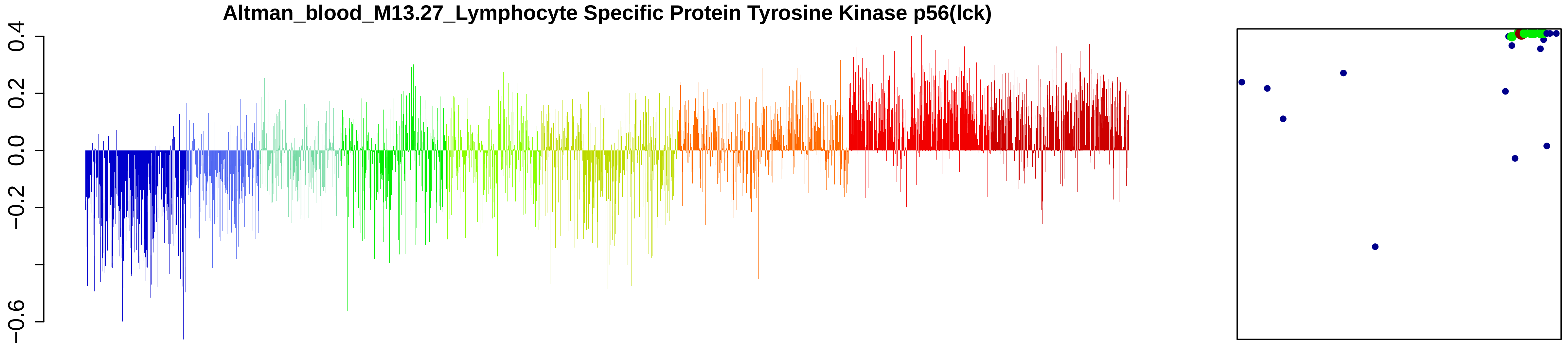
Altman_blood_M13.27_Lymphocyte Specific Protein Tyrosine Kinase p56(lck)
0.4
0.2
0.0
−0.2
−0.6

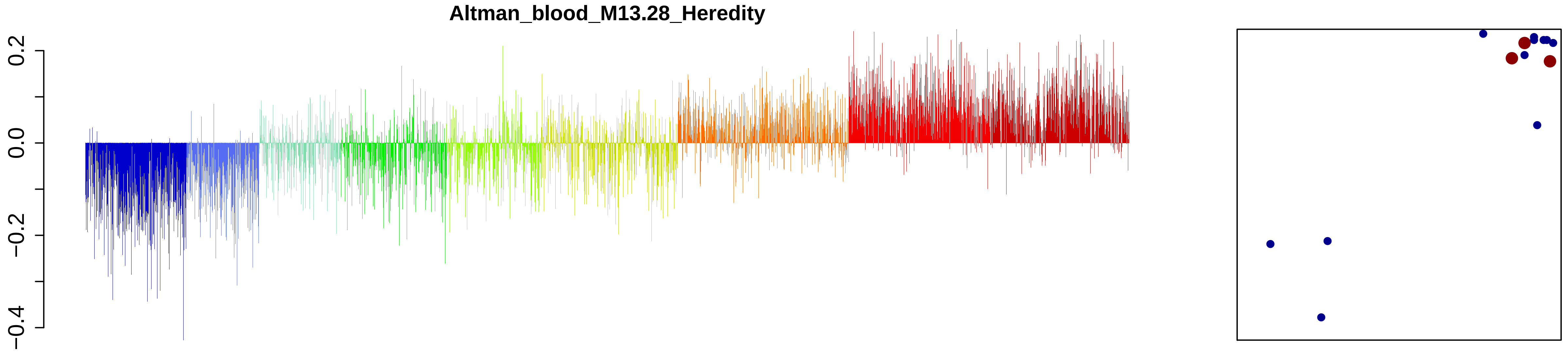
Altman_blood_M13.28_Heredity
0.2
0.0
−0.2
−0.4

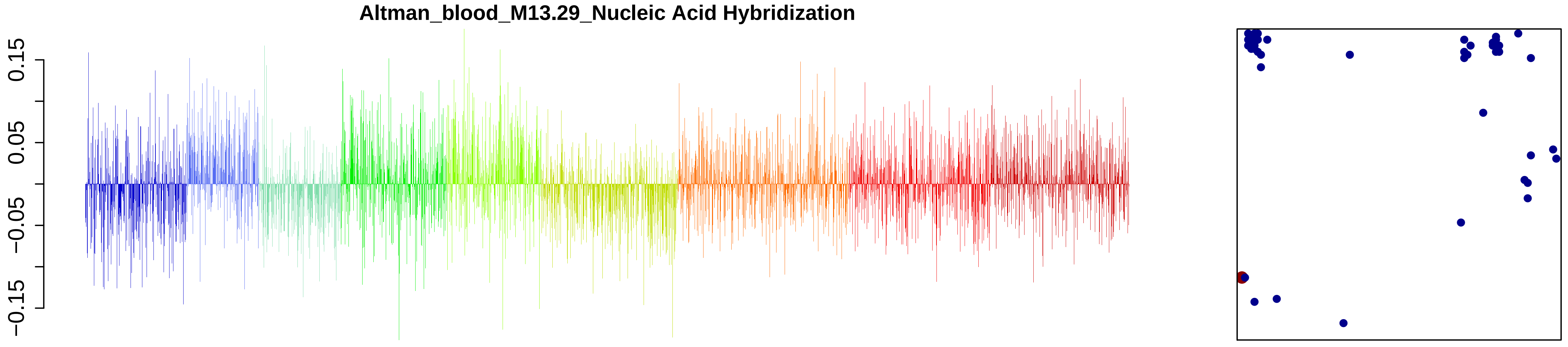
Altman_blood_M13.29_Nucleic Acid Hybridization
0.15
0.05
−0.05
−0.15

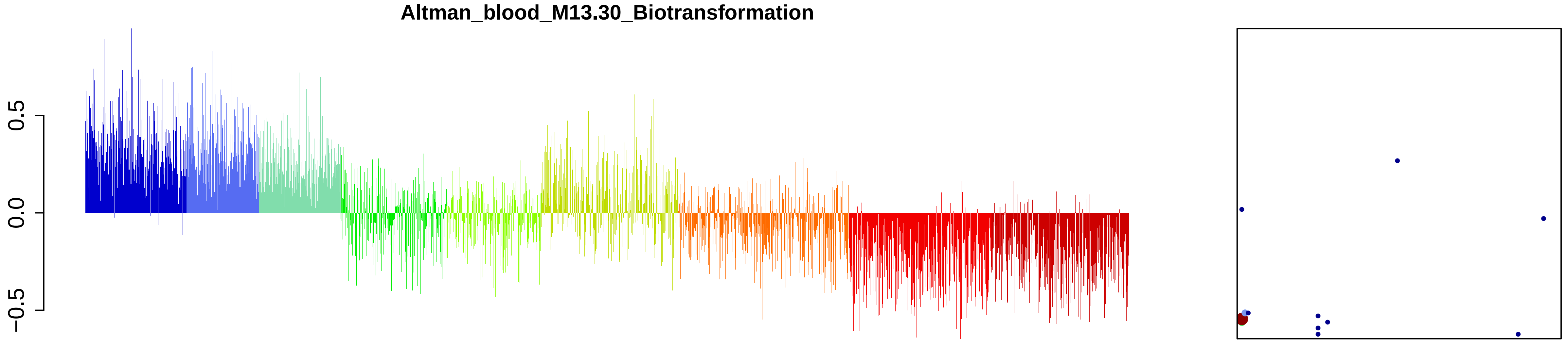
Altman_blood_M13.30_Biotransformation
0.5
0.0
−0.5

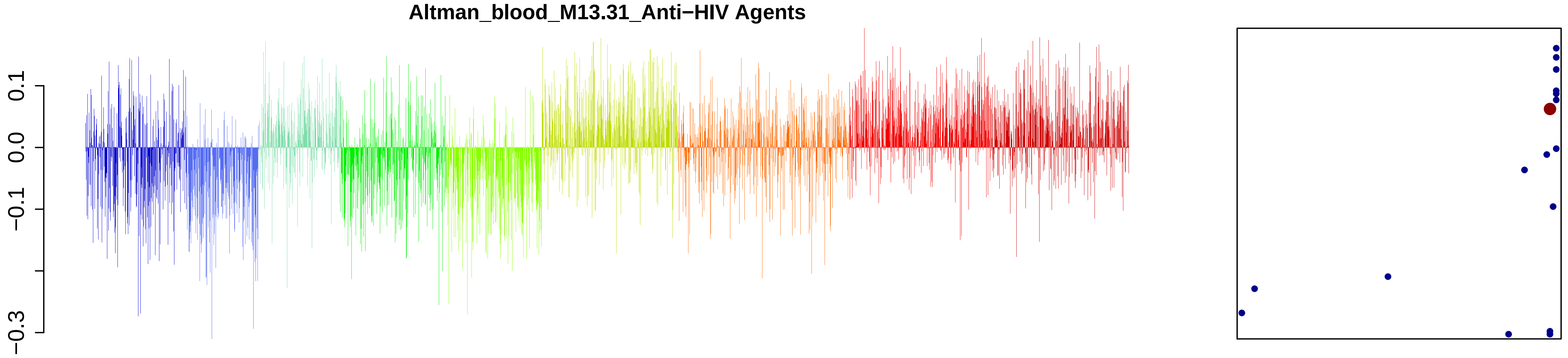
Altman_blood_M13.31_Anti−HIV Agents
0.1
0.0
−0.1
−0.3

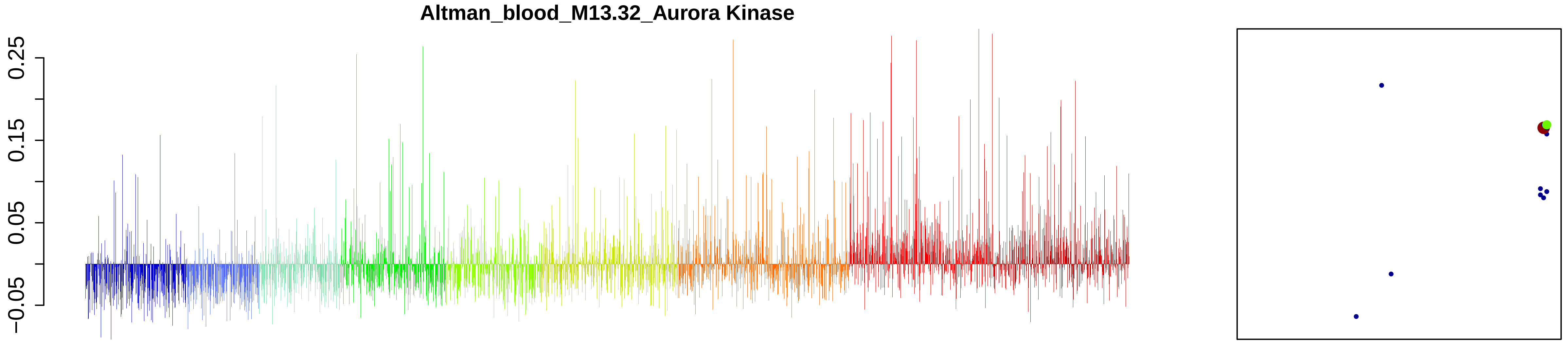
Altman_blood_M13.32_Aurora Kinase
0.25
0.15
0.05
−0.05

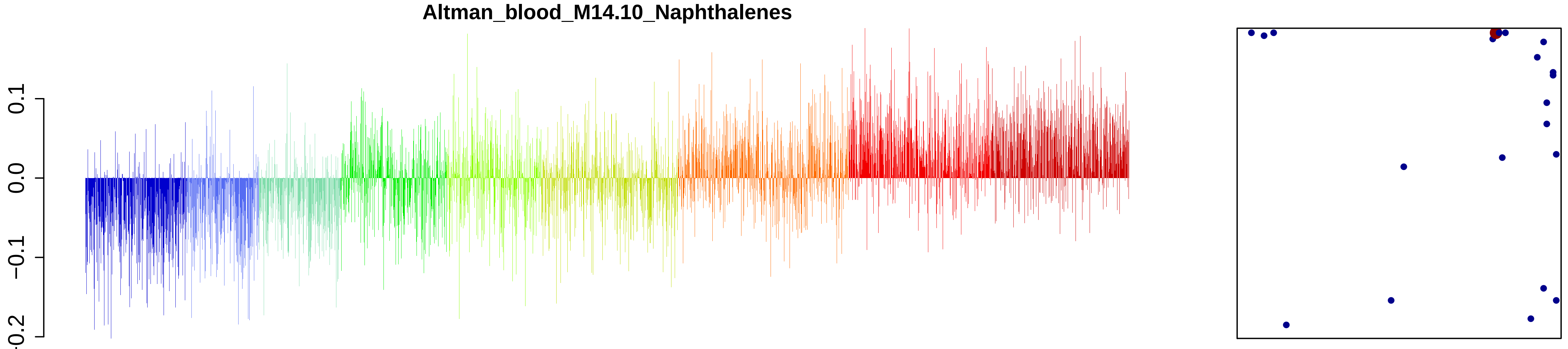
Altman_blood_M14.10_Naphthalenes
0.1
0.0
−0.1
−0.2

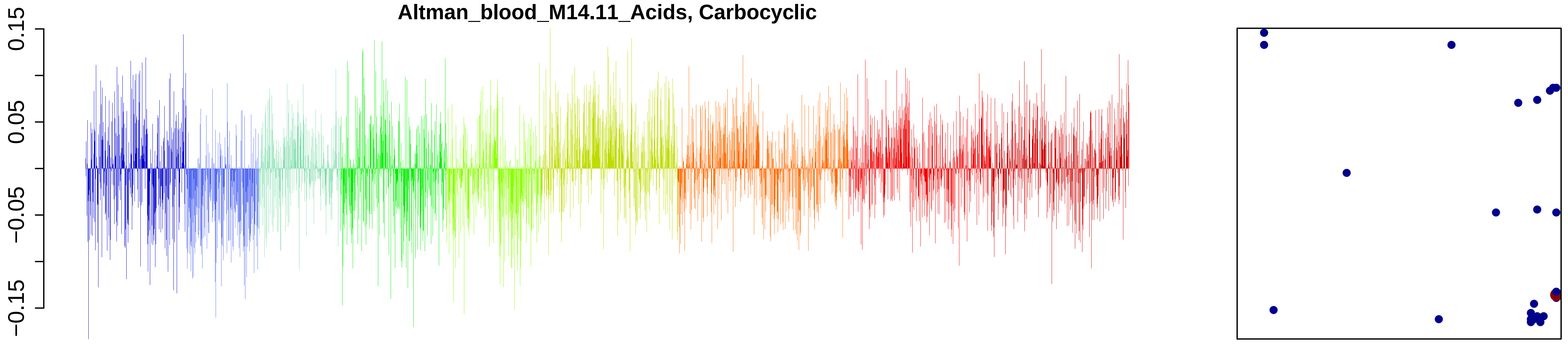
Altman_blood_M14.11_Acids, Carbocyclic
0.15
0.05
−0.05
−0.15

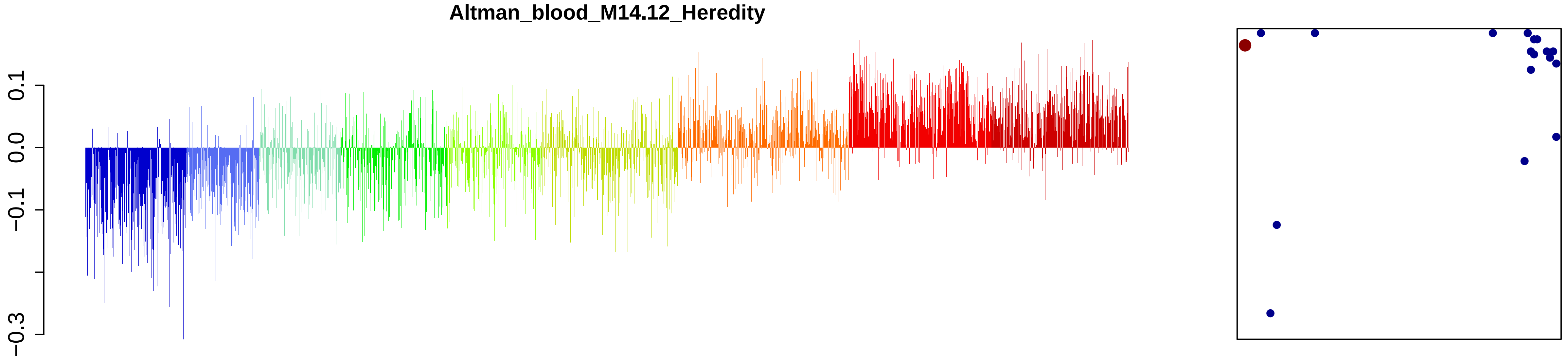
Altman_blood_M14.12_Heredity
0.1
0.0
−0.1
−0.3

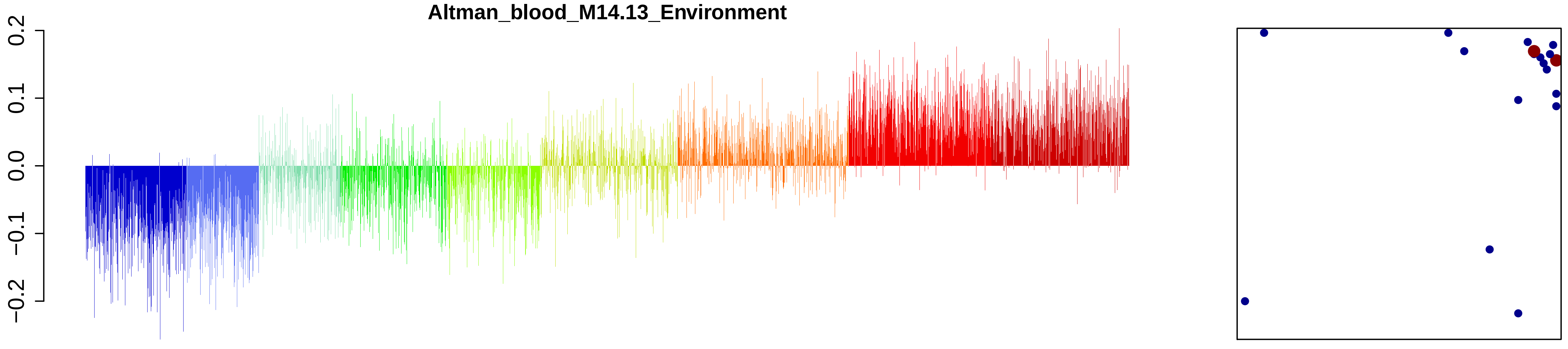
Altman_blood_M14.13_Environment
0.2
0.1
0.0
−0.1
−0.2

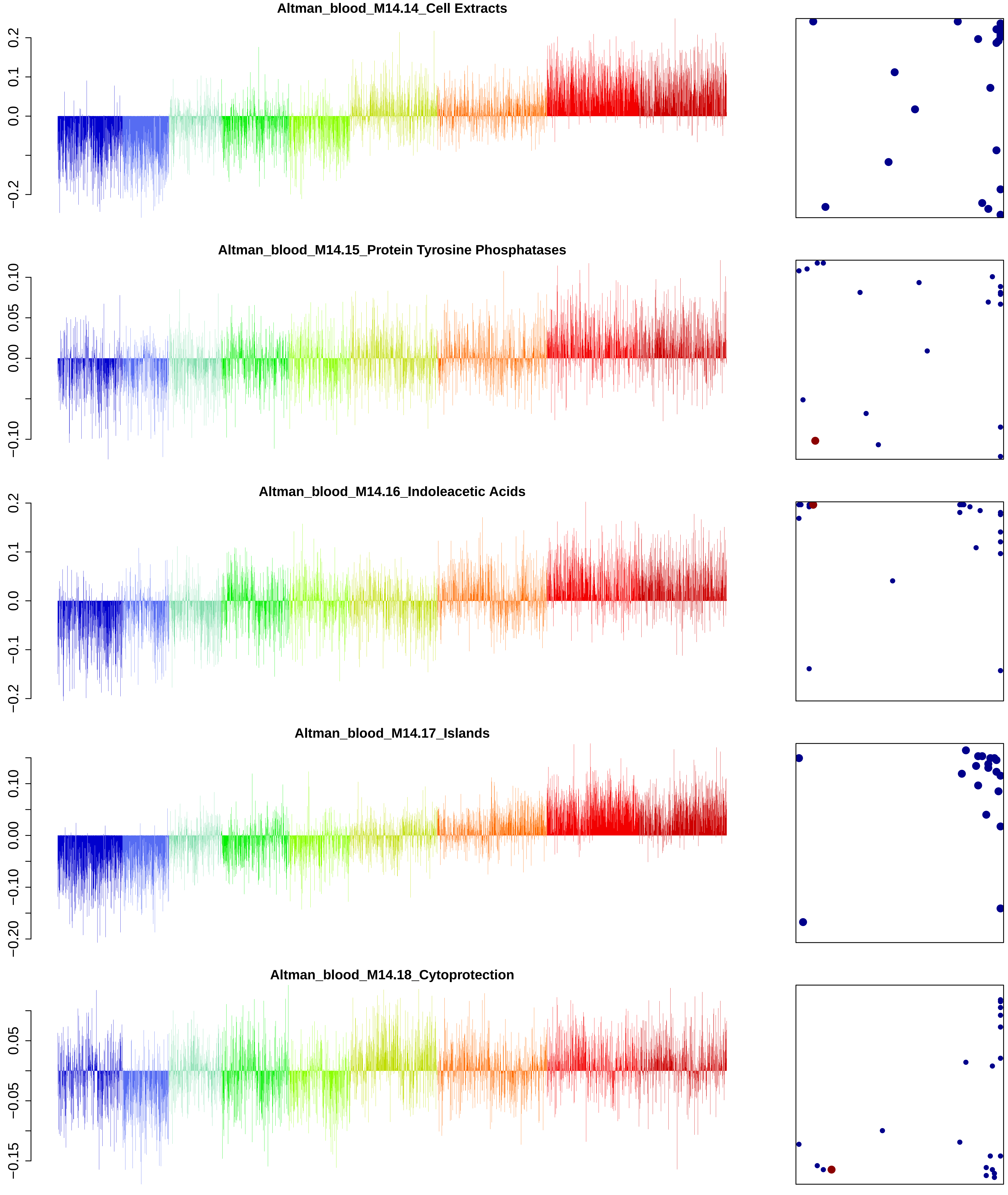

Altman_blood_M14.14_Cell Extracts
0.2
0.1
0.0
−0.2
Altman_blood_M14.15_Protein Tyrosine Phosphatases
0.10
0.05
0.00
−0.10
Altman_blood_M14.16_Indoleacetic Acids
0.2
0.1
0.0
−0.1
−0.2
Altman_blood_M14.17_Islands
0.10
0.00
−0.10
−0.20
Altman_blood_M14.18_Cytoprotection
0.05
−0.05
−0.15

Altman_blood_M14.19_Genes, Immediate−Early

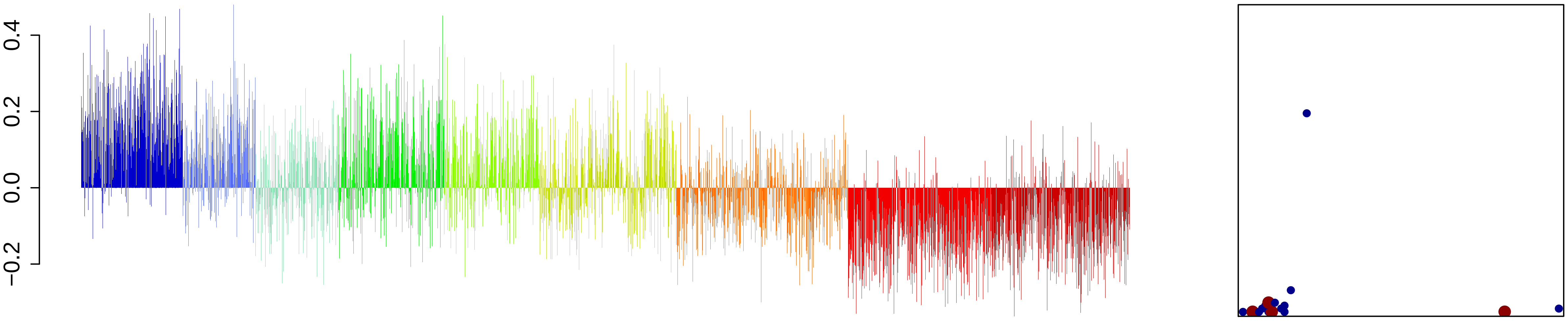

Altman_blood_M14.20_Specialty Uses of Chemicals

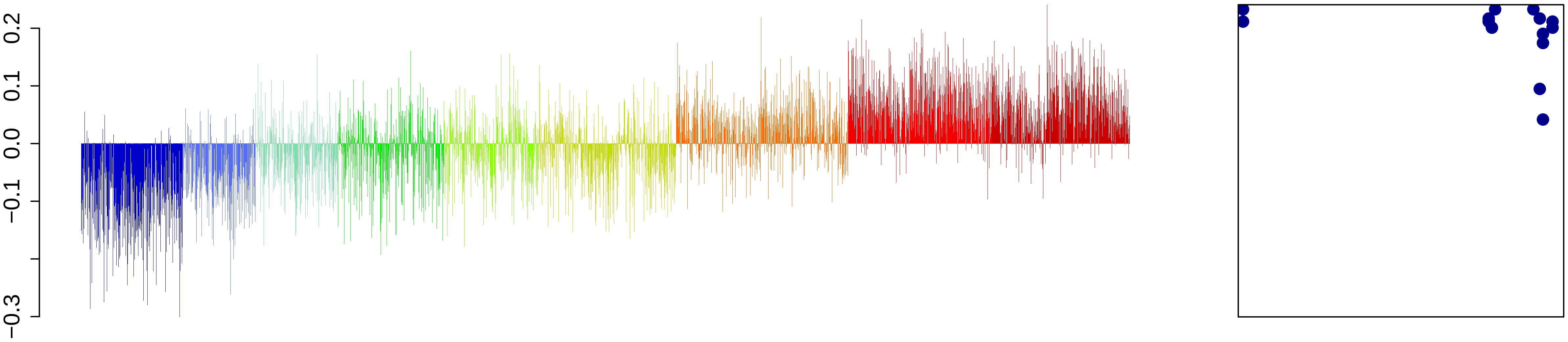

Altman_blood_M14.21_Behavior

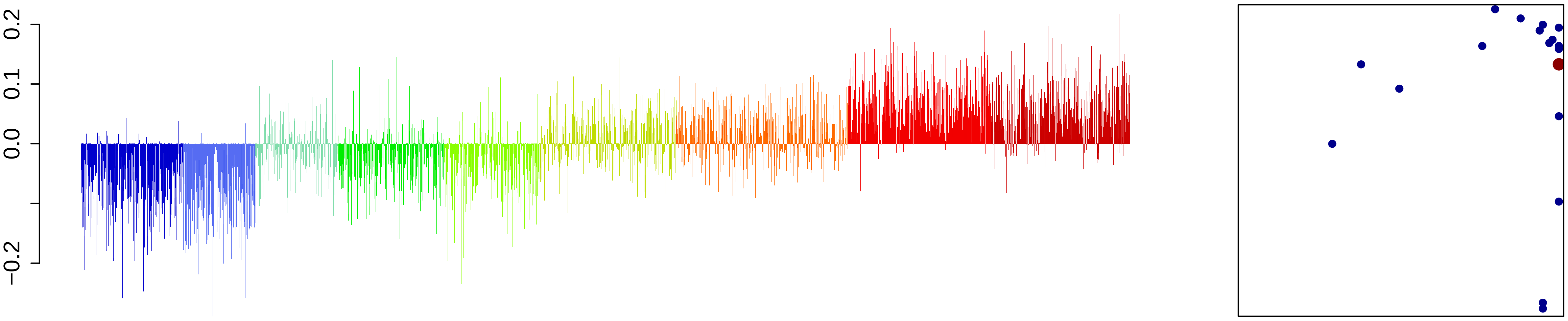

Altman_blood_M14.22_Giant Cells

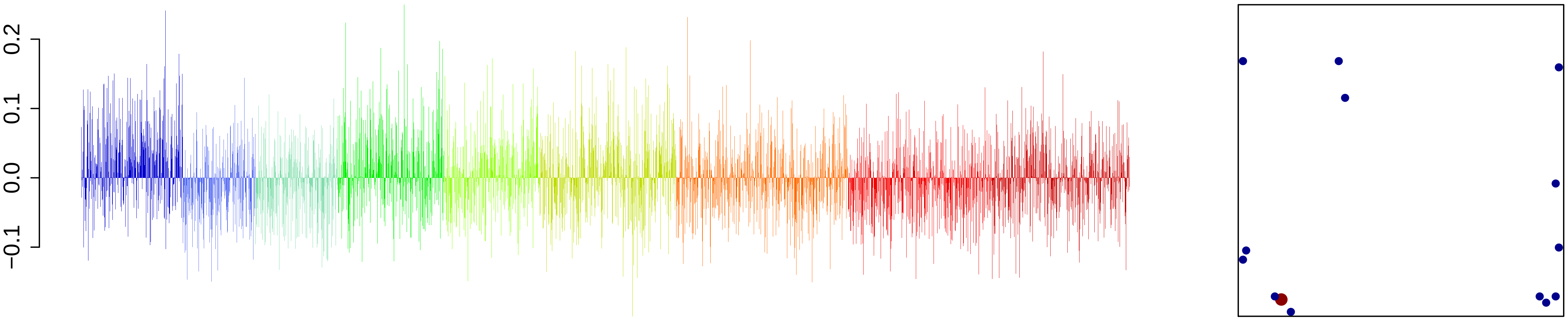

Altman_blood_M14.23_Genetic Phenomena

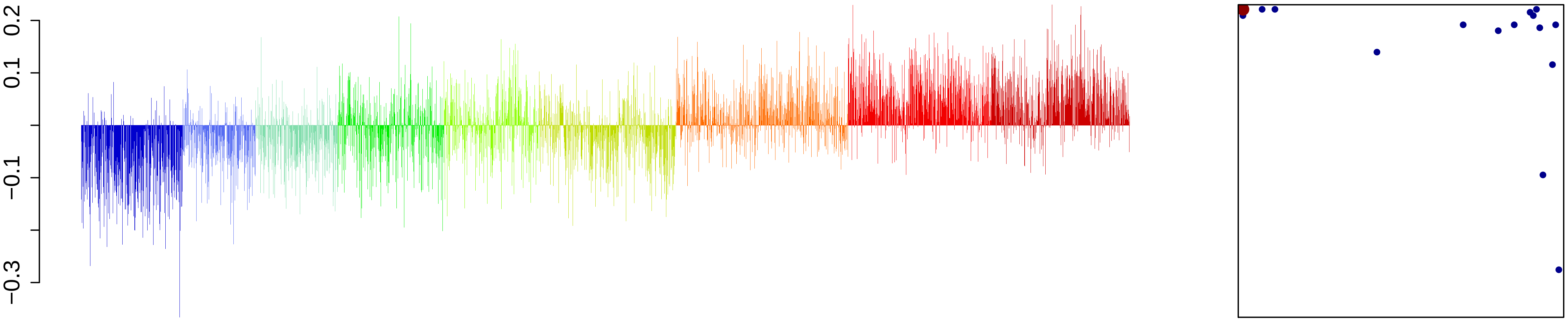

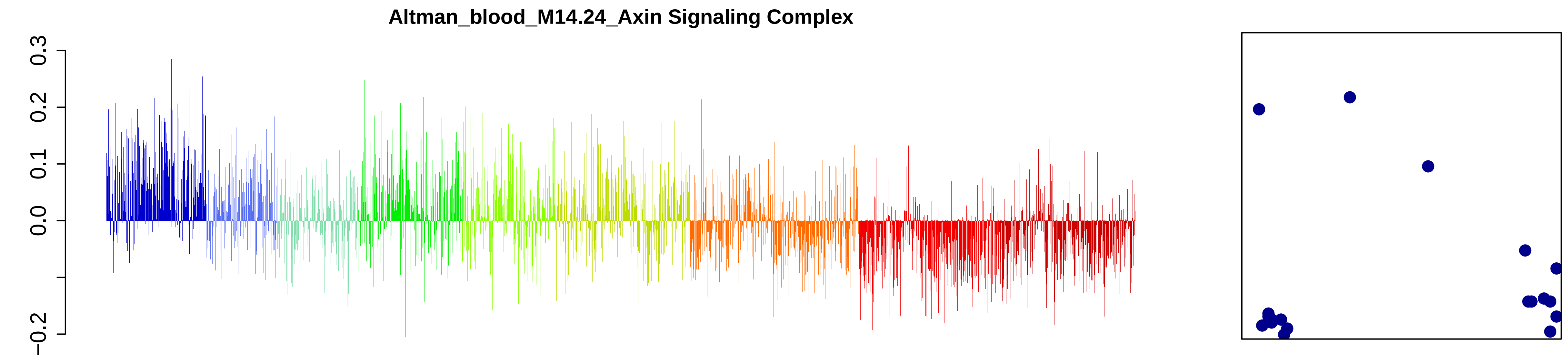
Altman_blood_M14.24_Axin Signaling Complex
0.3
0.2
0.1
0.0
−0.2

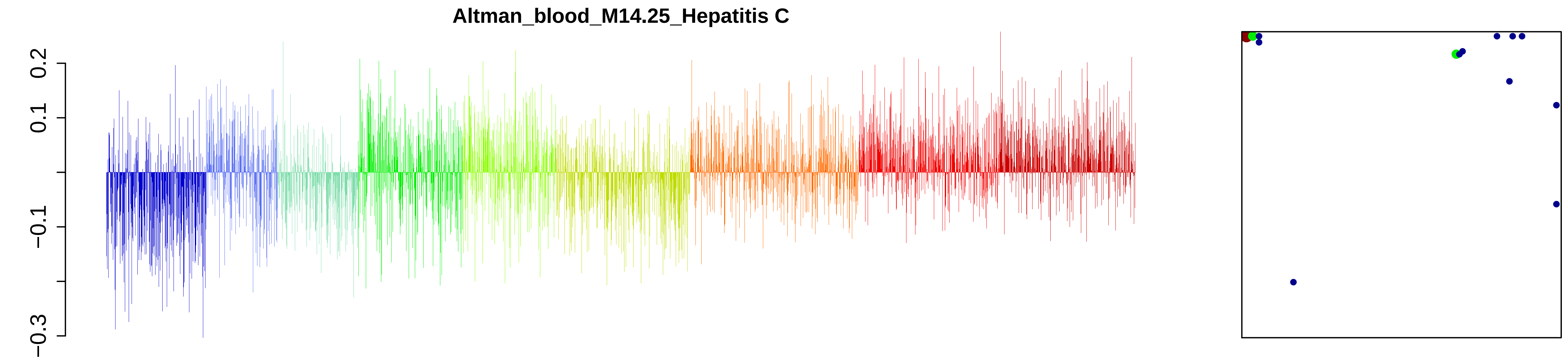
Altman_blood_M14.25_Hepatitis C
0.2
0.1
−0.1
−0.3

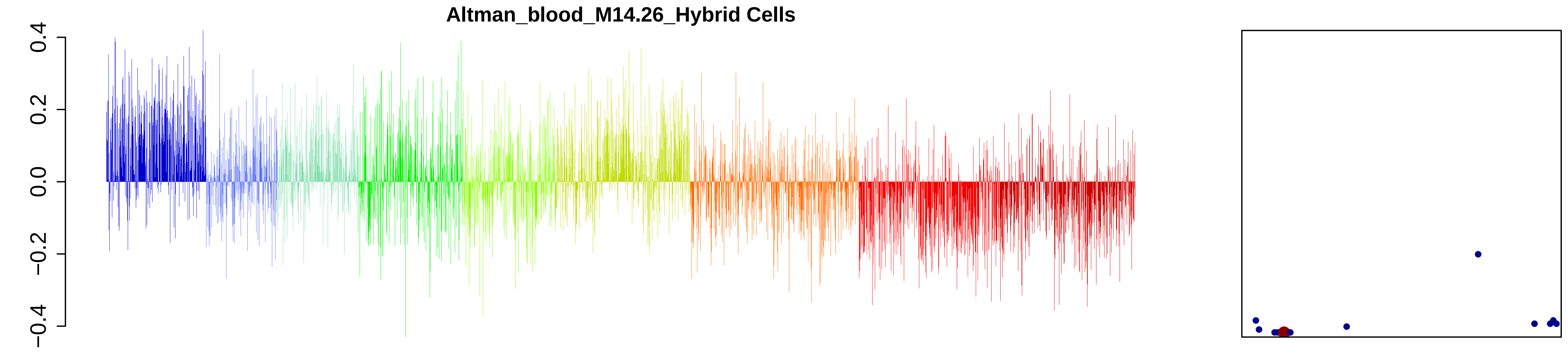
Altman_blood_M14.26_Hybrid Cells
0.4
0.2
0.0
−0.2
−0.4

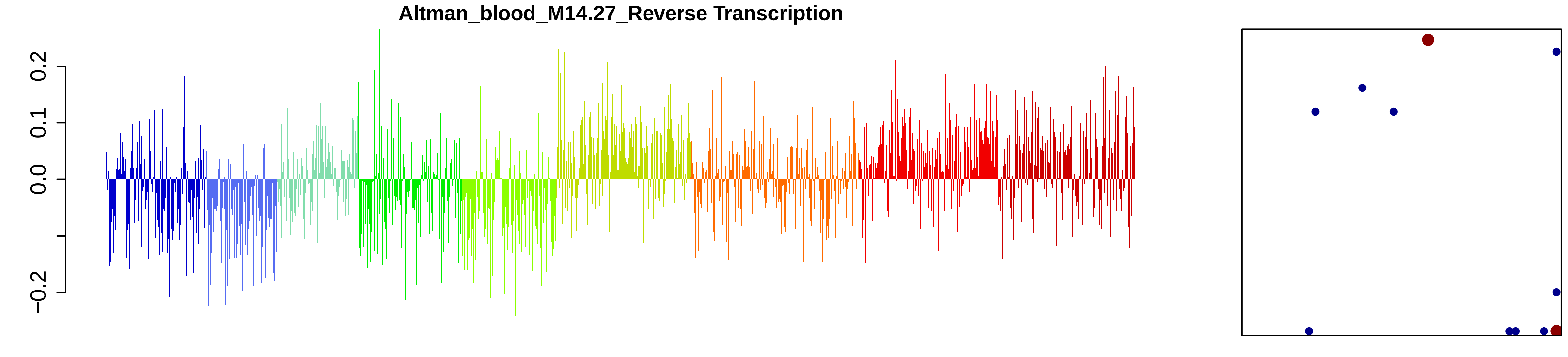
Altman_blood_M14.27_Reverse Transcription
0.2
0.1
0.0
−0.2

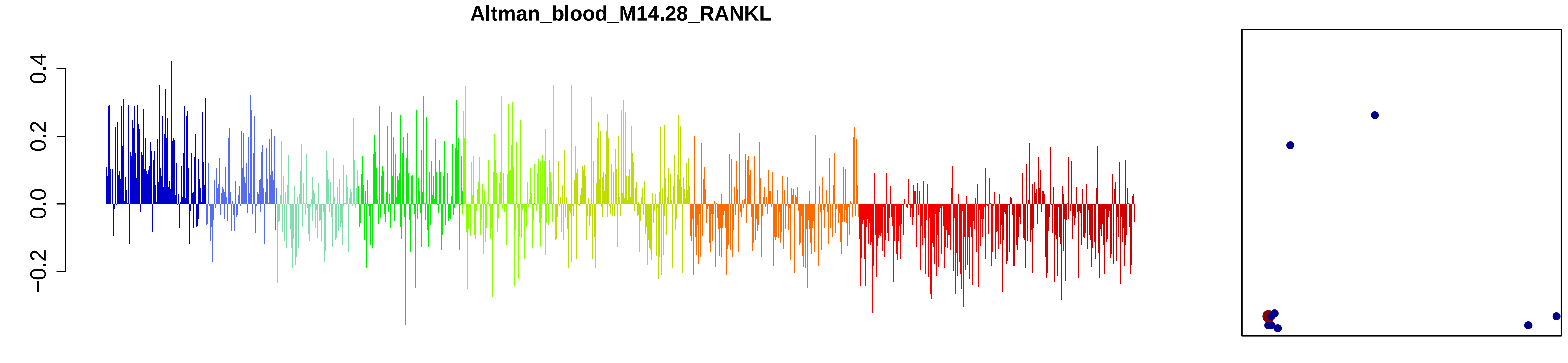
Altman_blood_M14.28_RANKL
0.4
0.2
0.0
−0.2

Altman_blood_M14.29_Genetic Code

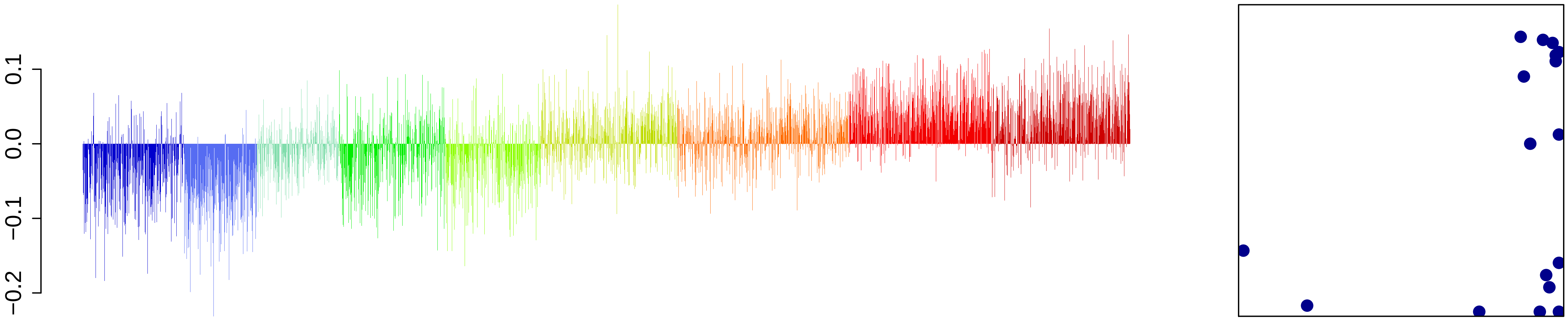

Altman_blood_M14.30_Iron−Binding Proteins

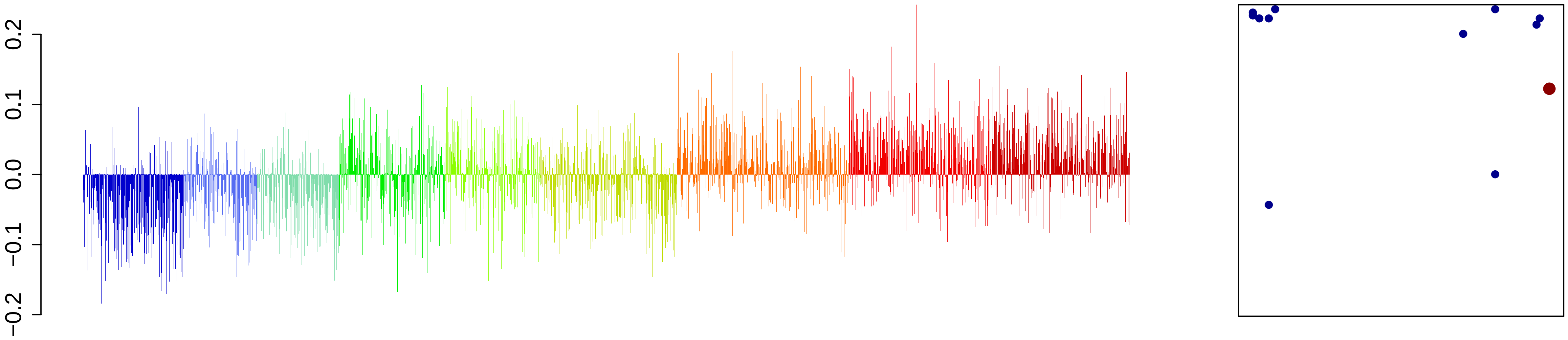

Altman_blood_M14.31_Mutant Proteins

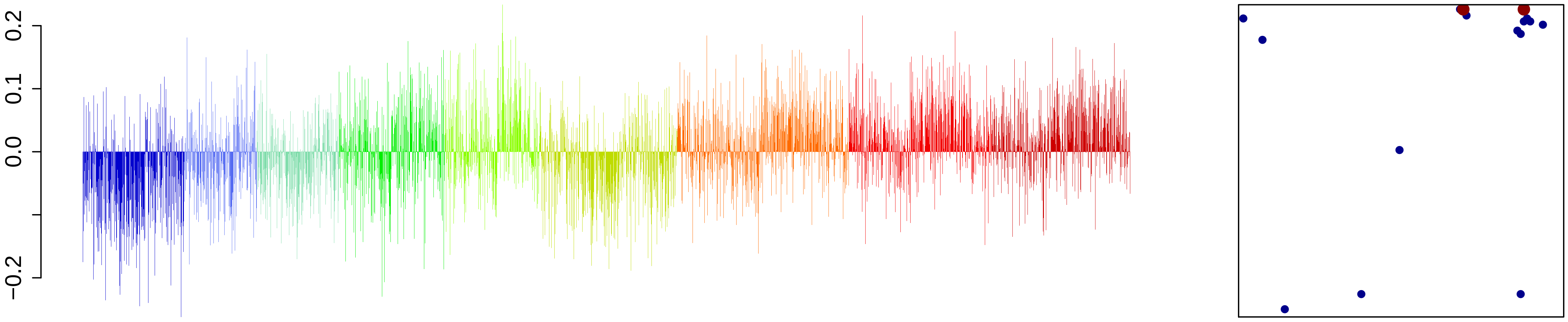

Altman_blood_M14.32_Macrolides

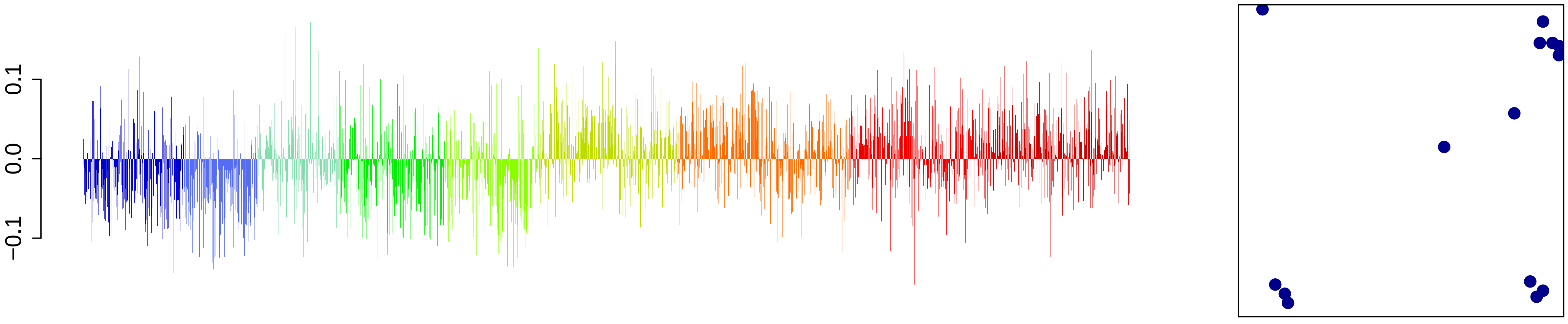

Altman_blood_M14.33_Cysteine Proteinase Inhibitors

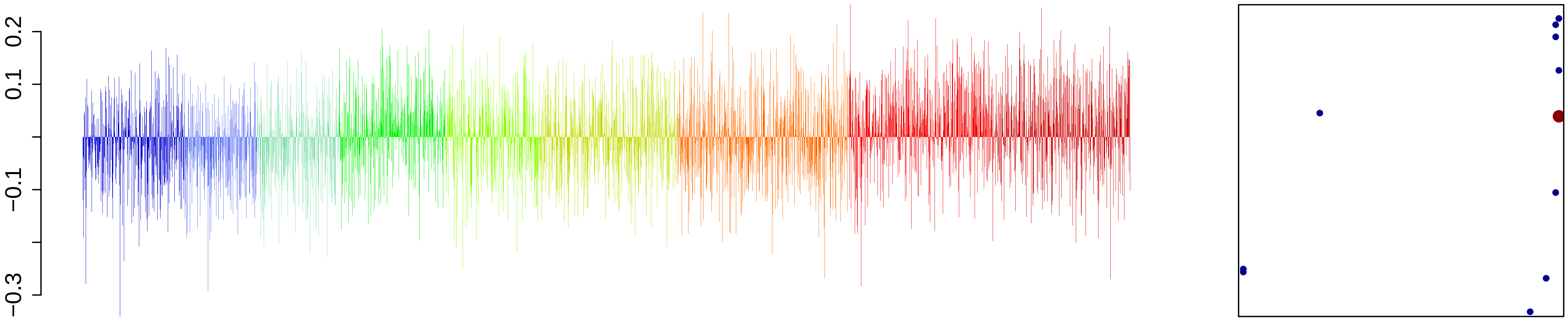

Altman_blood_M14.34_Pentosyltransferases

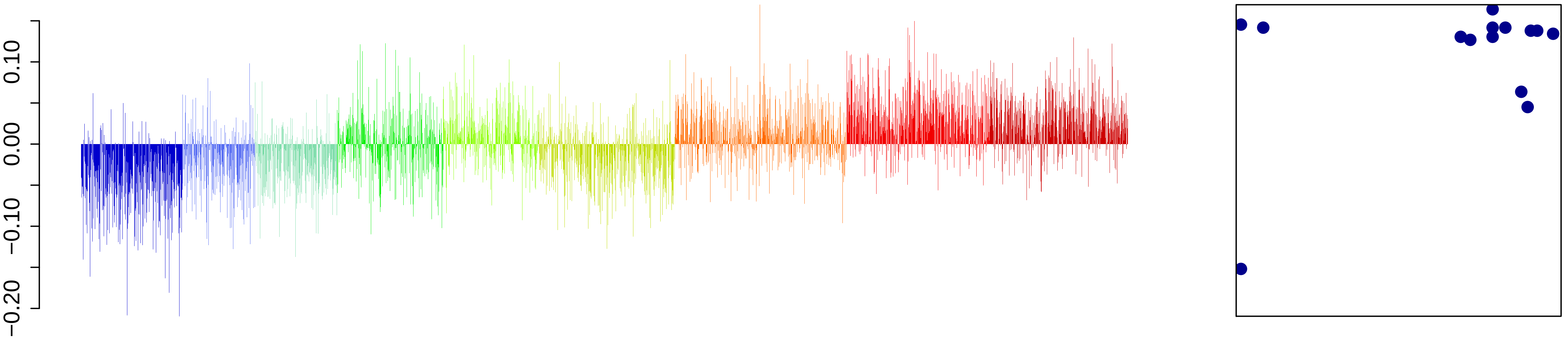

Altman_blood_M14.35_Biotransformation

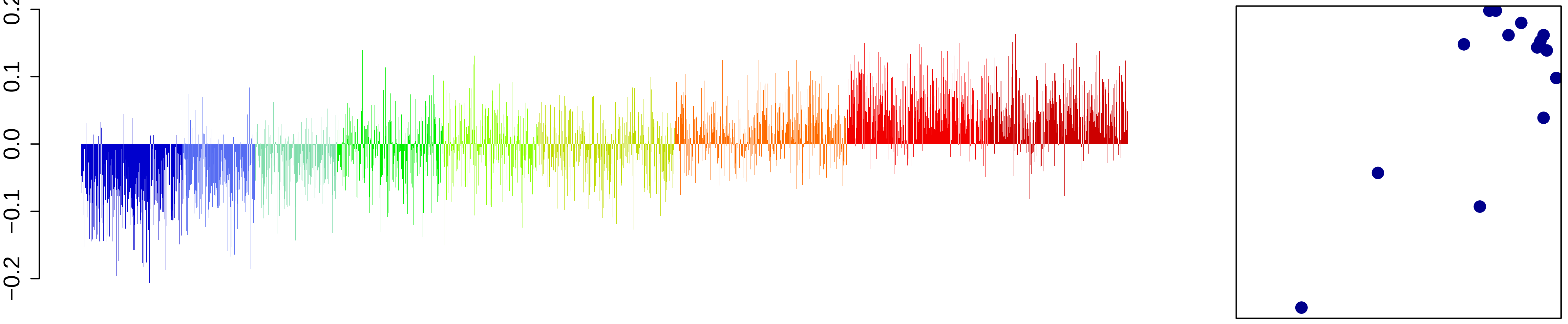

Altman_blood_M14.36_Ear

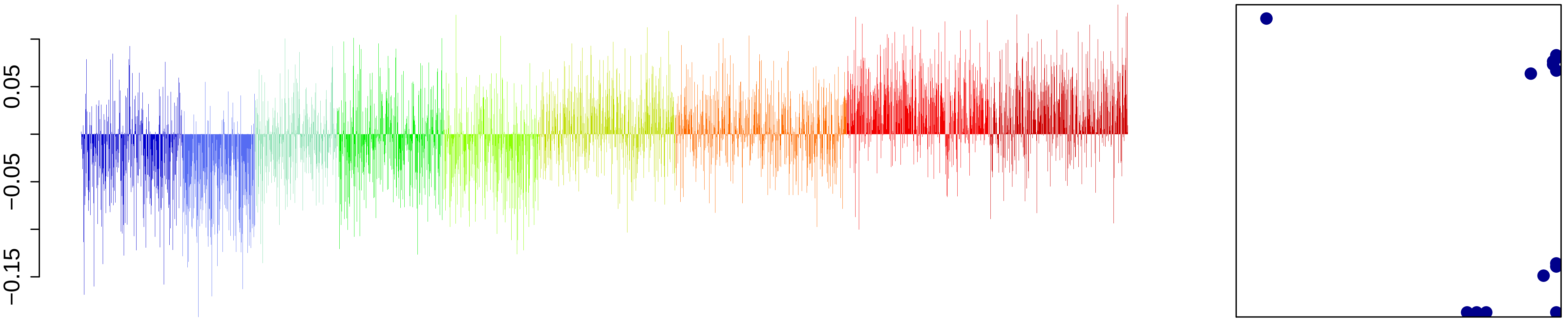

Altman_blood_M14.37_Polycyclic Compounds

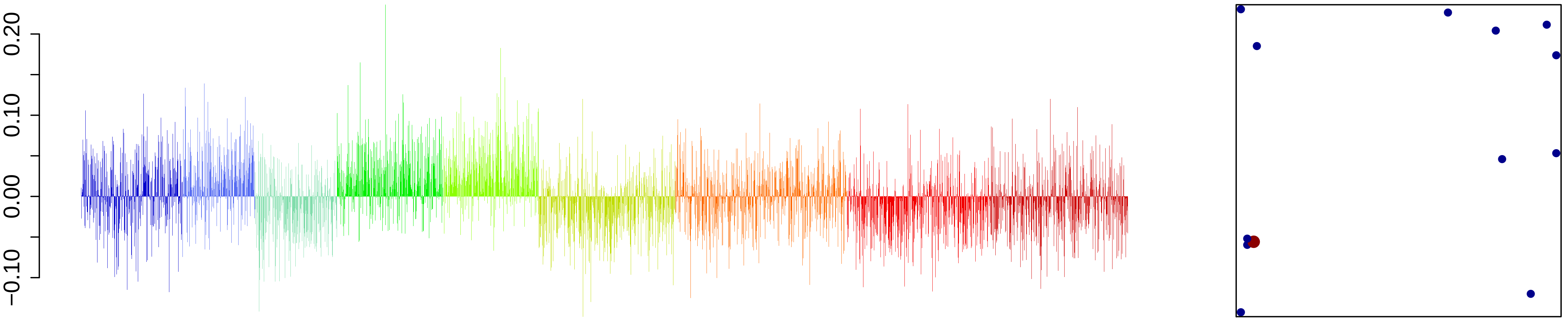

Altman_blood_M14.38_Schizophrenia

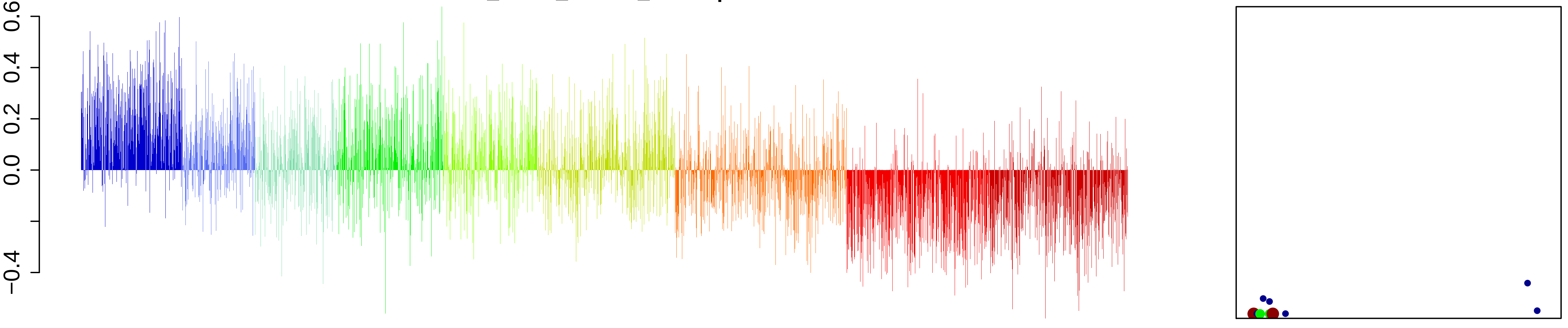

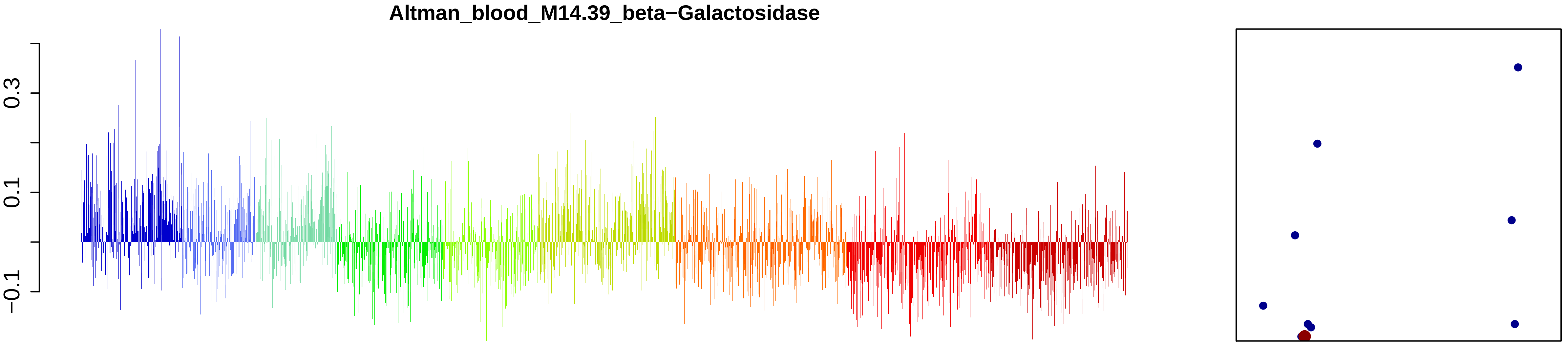
Altman_blood_M14.39_beta−Galactosidase
0.3
0.1
−0.1

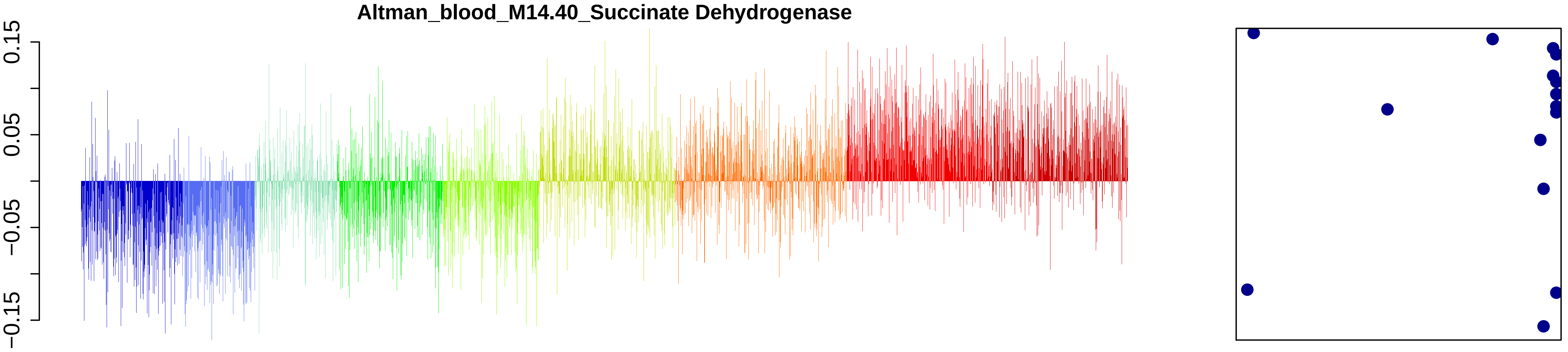
Altman_blood_M14.40_Succinate Dehydrogenase
0.15
0.05
−0.05
−0.15

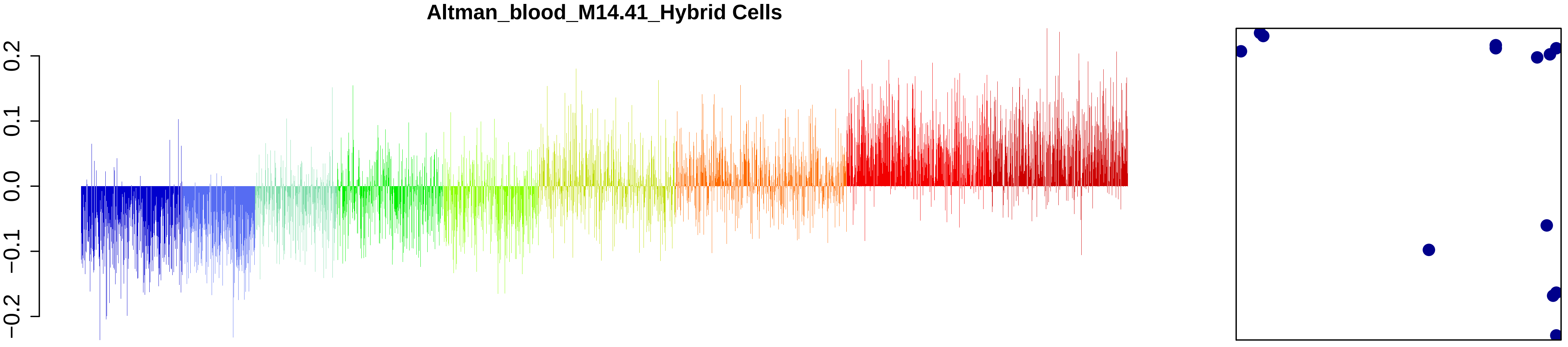
Altman_blood_M14.41_Hybrid Cells
0.2
0.1
0.0
−0.1
−0.2

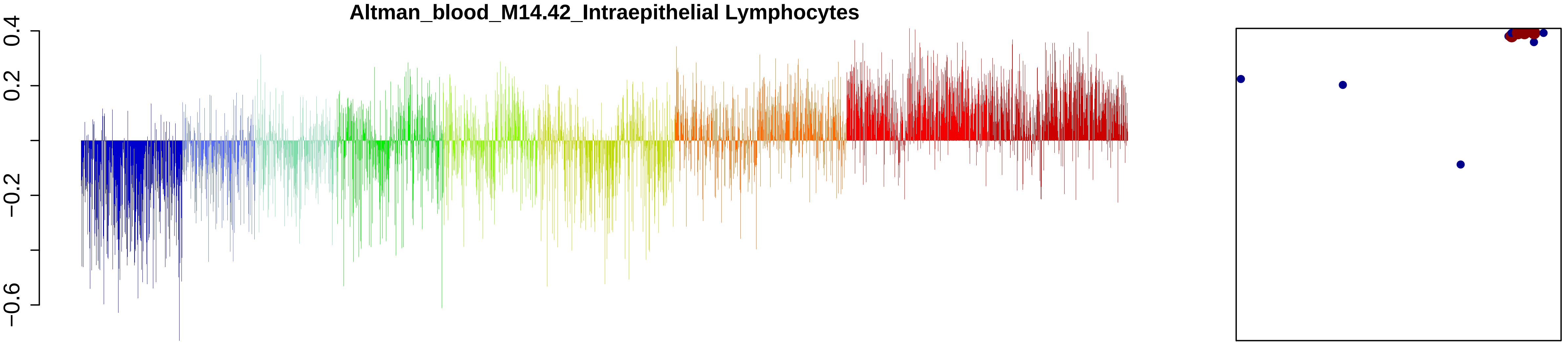
Altman_blood_M14.42_Intraepithelial Lymphocytes
0.4
0.2
−0.2
−0.6

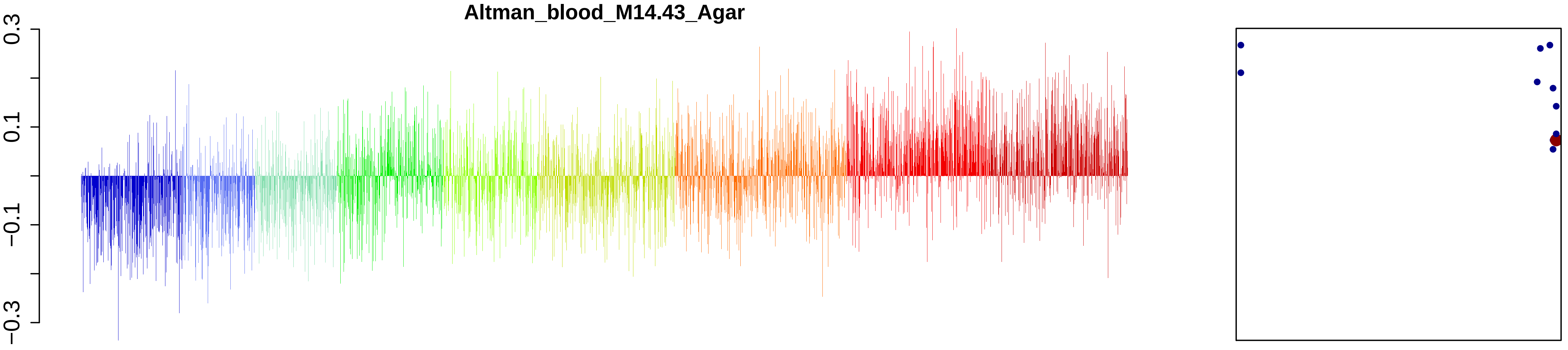
Altman_blood_M14.43_Agar
0.3
0.1
−0.1
−0.3

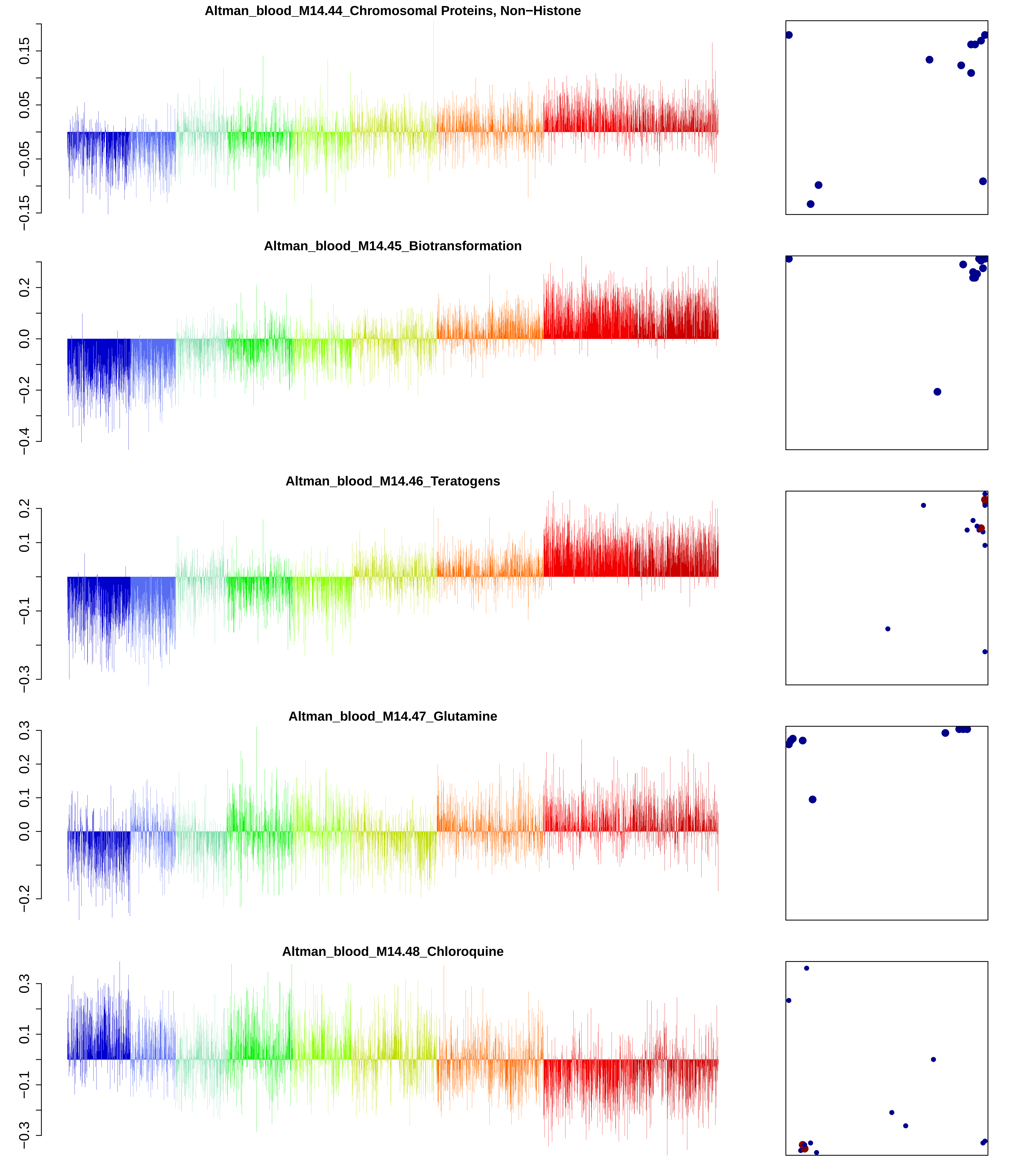
Altman_blood_M14.44_Chromosomal Proteins, Non−Histone
0.15
0.05
−0.05
−0.15
Altman_blood_M14.45_Biotransformation
0.2
0.0
−0.2
−0.4
Altman_blood_M14.46_Teratogens
0.2
0.1
−0.1
−0.3
Altman_blood_M14.47_Glutamine
0.3
0.2
0.1
0.0
−0.2
Altman_blood_M14.48_Chloroquine
0.3
0.1
−0.1
−0.3

Altman_blood_M14.49_Epigenesis, Genetic

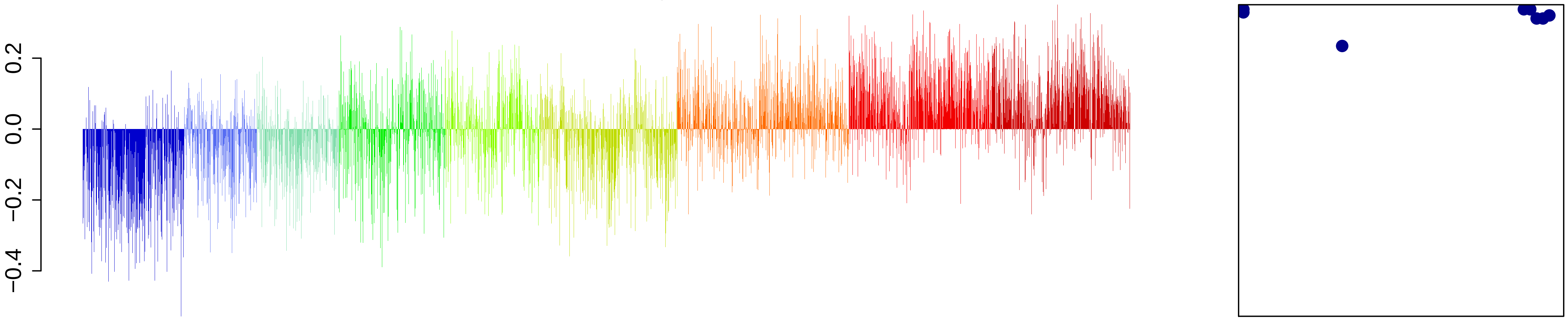

Altman_blood_M14.50_Junctional Adhesion Molecules

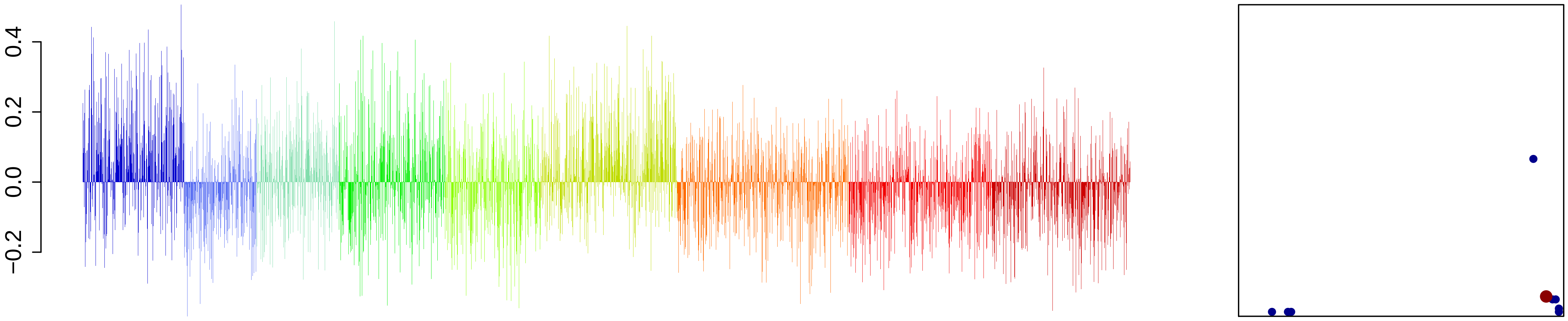

Altman_blood_M14.51_Peroxidase

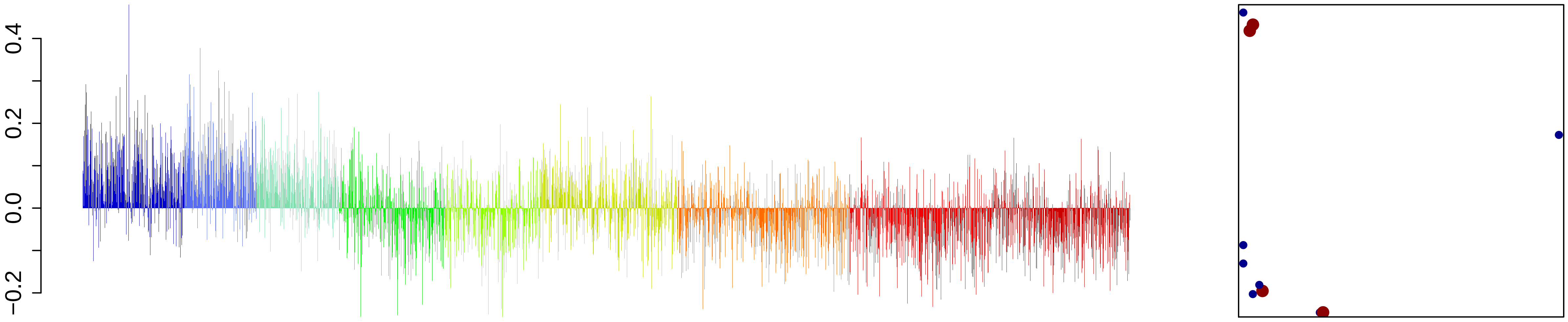

Altman_blood_M14.52_Lead

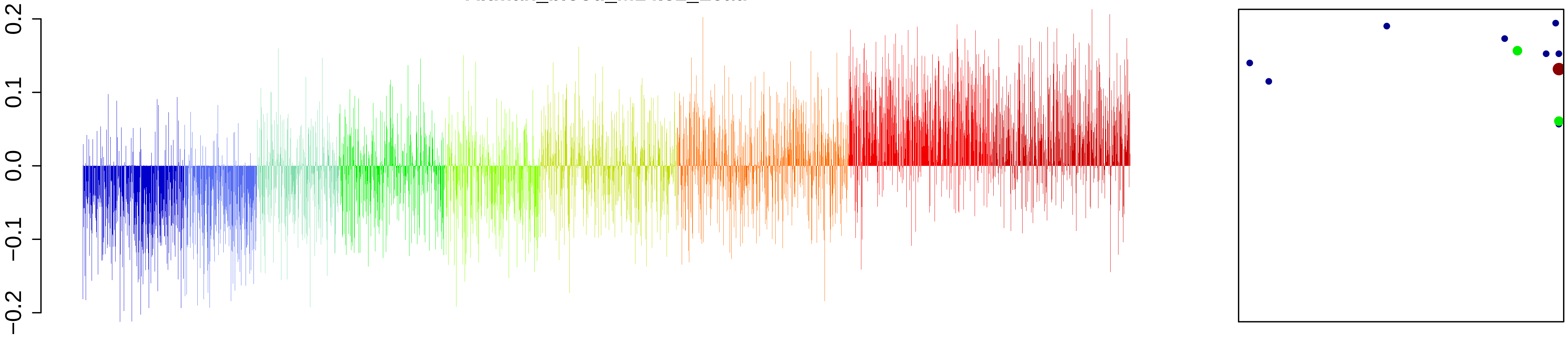

Altman_blood_M14.53_Megakaryocyte−Erythroid Progenitor Cells

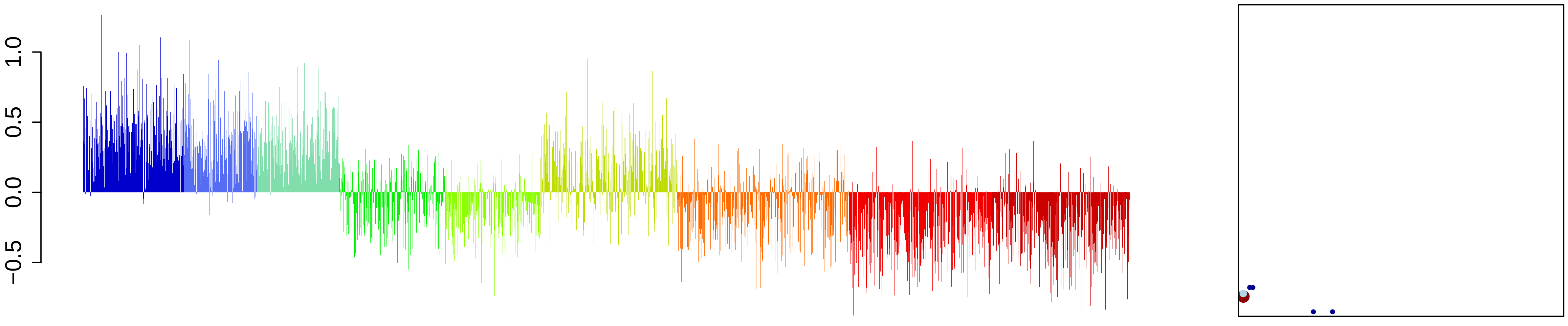

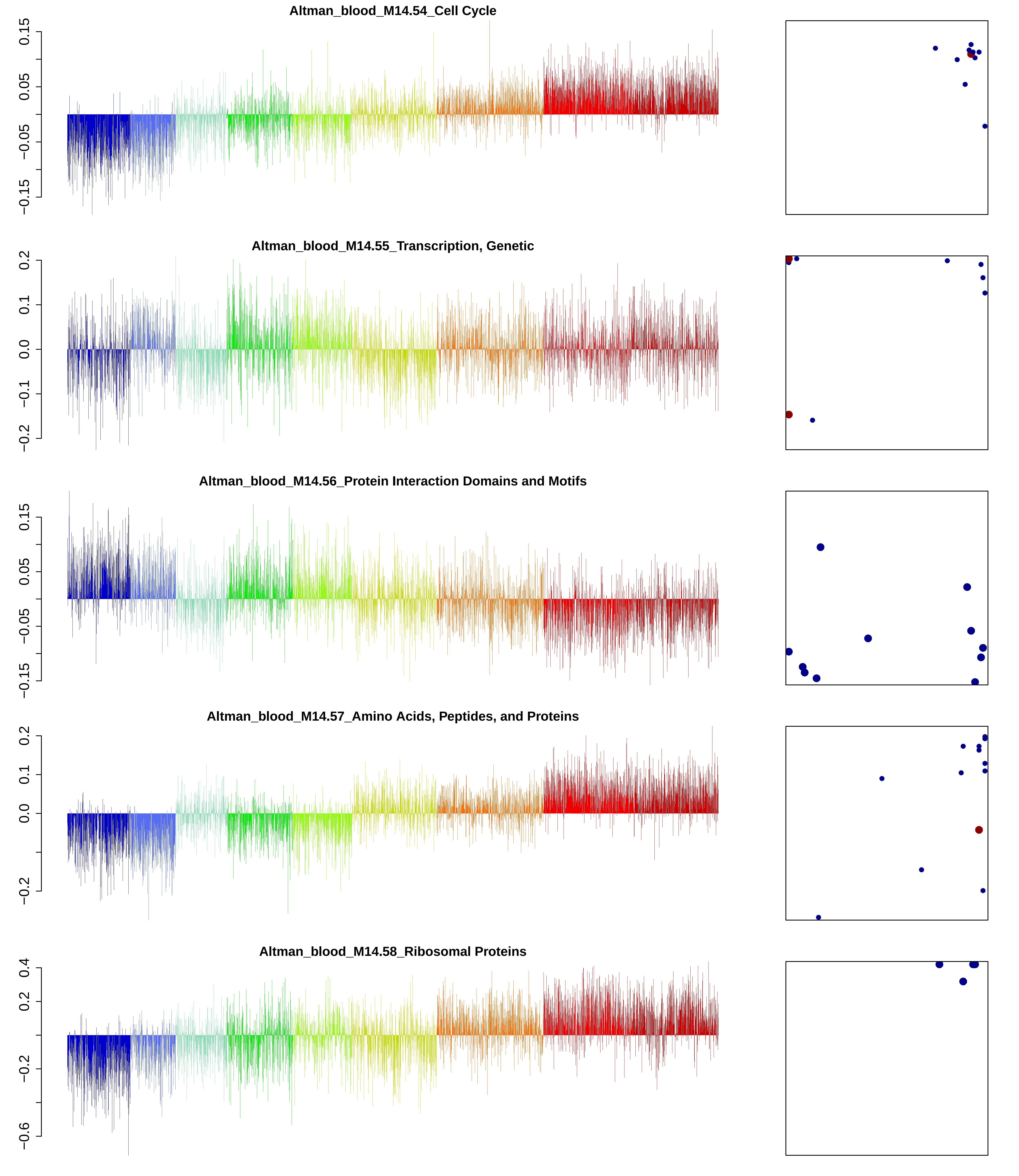
Altman_blood_M14.54_Cell Cycle
0.15
0.05
−0.05
−0.15
Altman_blood_M14.55_Transcription, Genetic
0.2
0.1
0.0
−0.1
−0.2
Altman_blood_M14.56_Protein Interaction Domains and Motifs
0.15
0.05
−0.05
−0.15
Altman_blood_M14.57_Amino Acids, Peptides, and Proteins
0.2
0.1
0.0
−0.2
Altman_blood_M14.58_Ribosomal Proteins
0.4
0.2
−0.2
−0.6

Altman_blood_M14.59_Thromboplastin

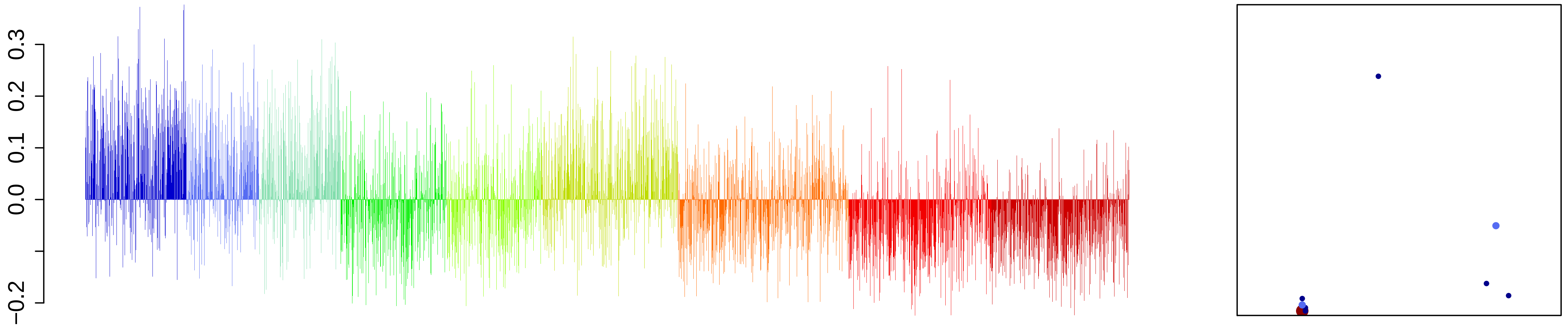

Altman_blood_M14.60_Behavior

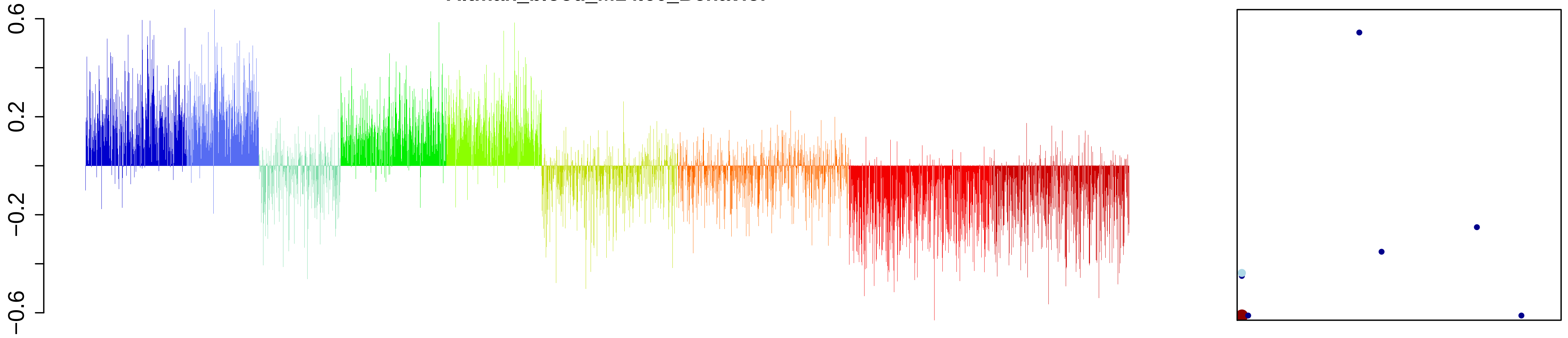

Altman_blood_M14.61_Sumoylation

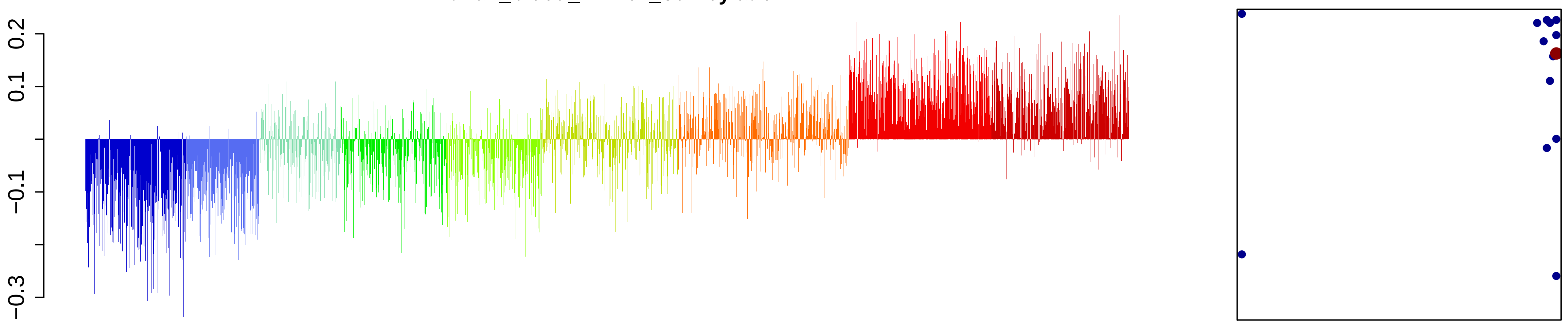

Altman_blood_M14.62_Neoplastic Stem Cells

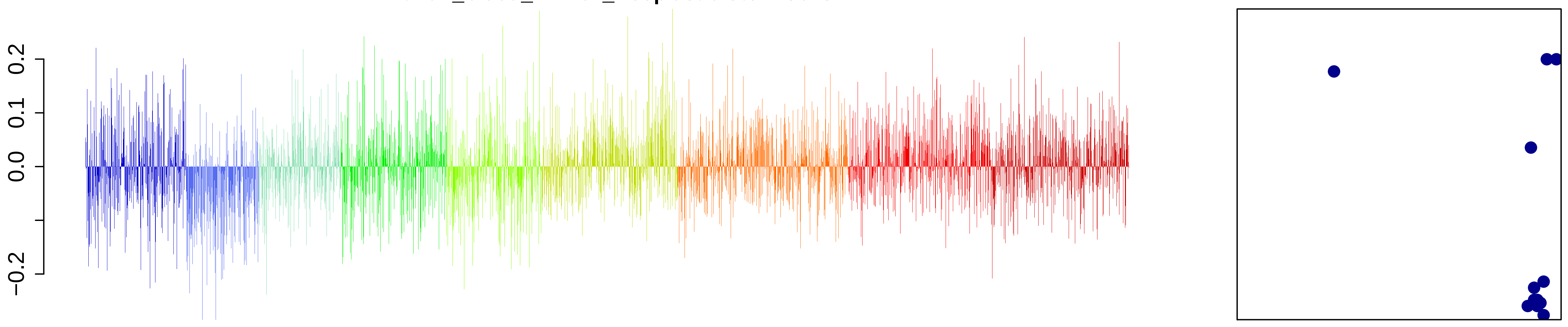

Altman_blood_M14.63_Multienzyme Complexes

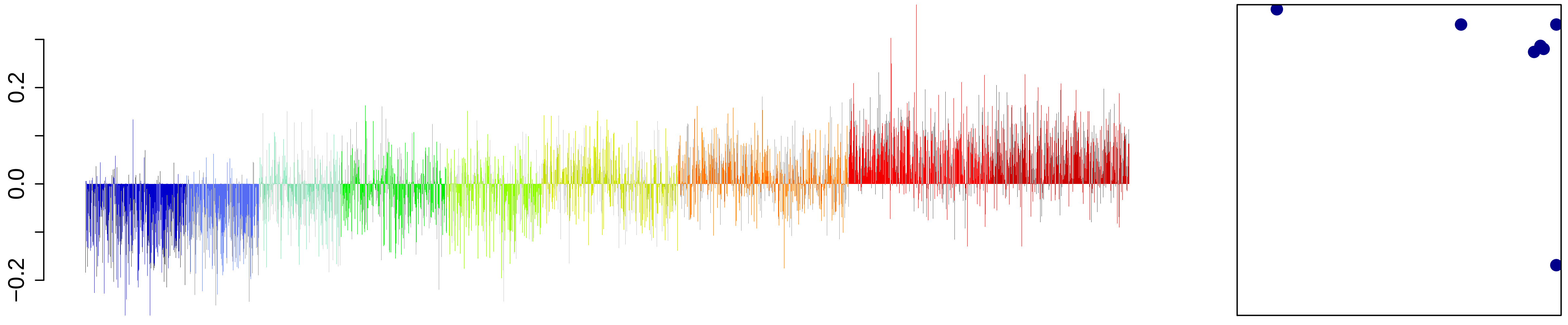

Altman_blood_M14.64_RNA Processing, Post−Transcriptional

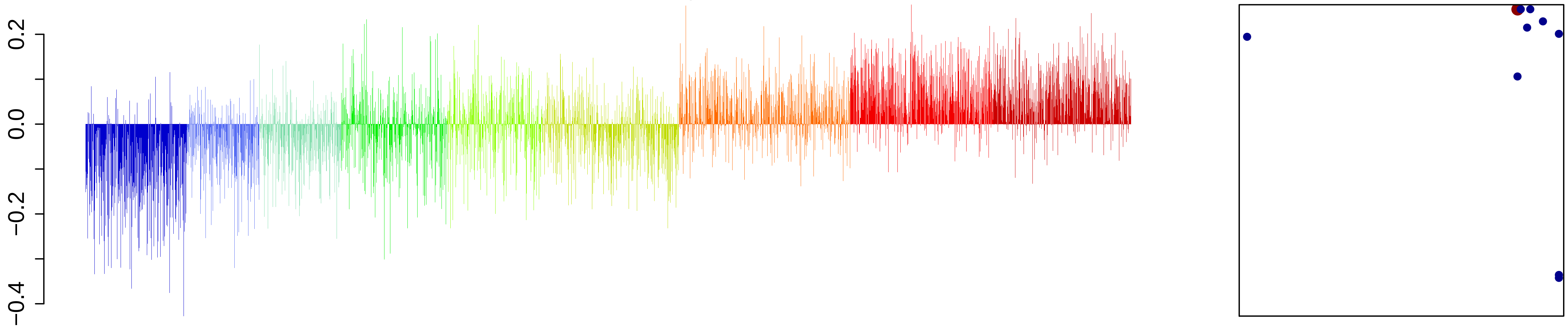

Altman_blood_M14.65_rab5 GTP−Binding Proteins

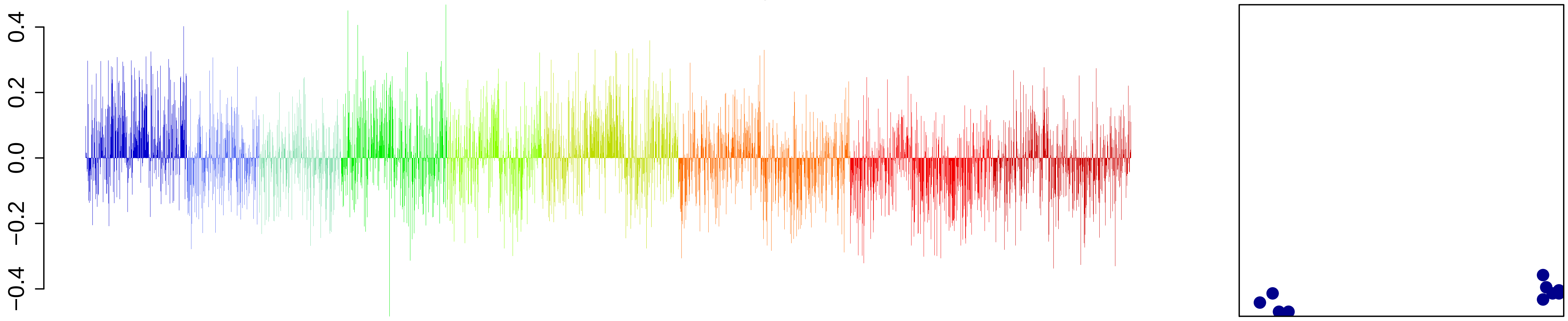

Altman_blood_M14.66_Caspase Cascade

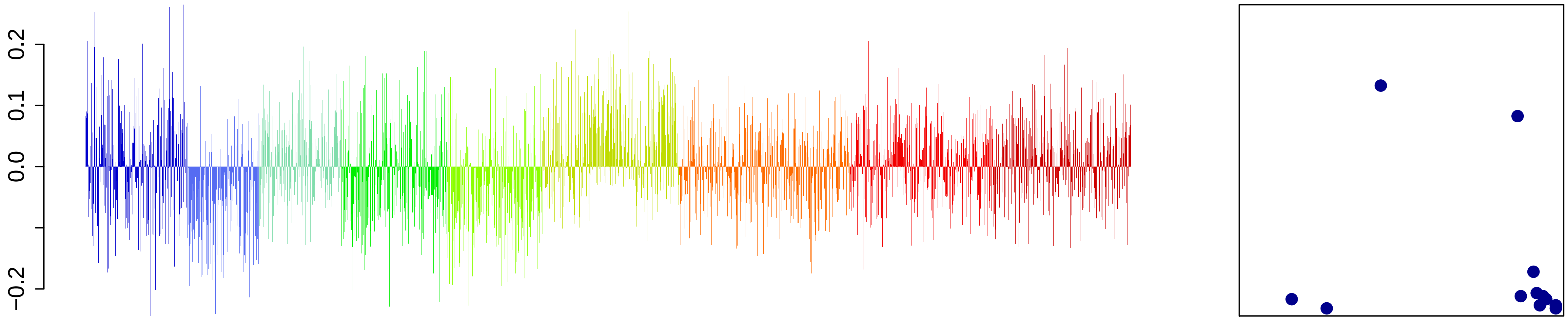

Altman_blood_M14.67_Luciferases

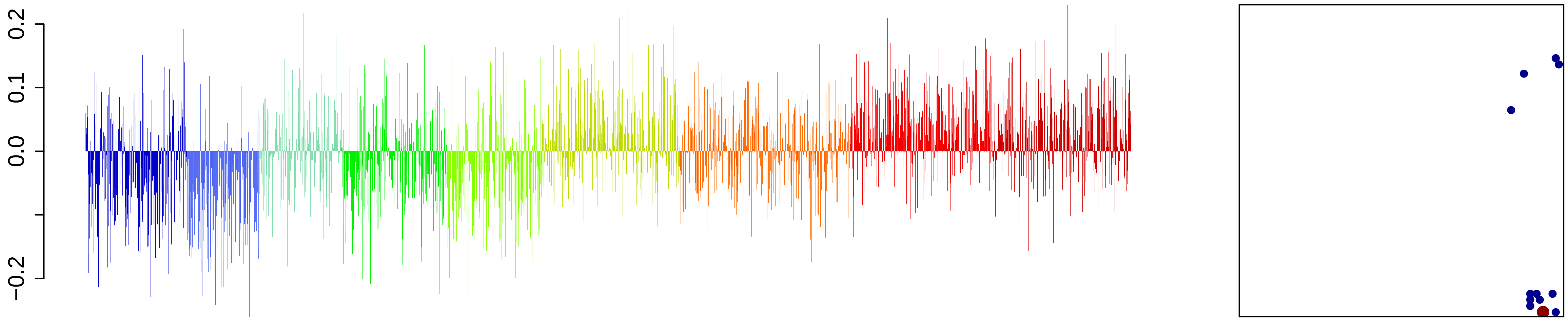

Altman_blood_M14.68_Clathrin

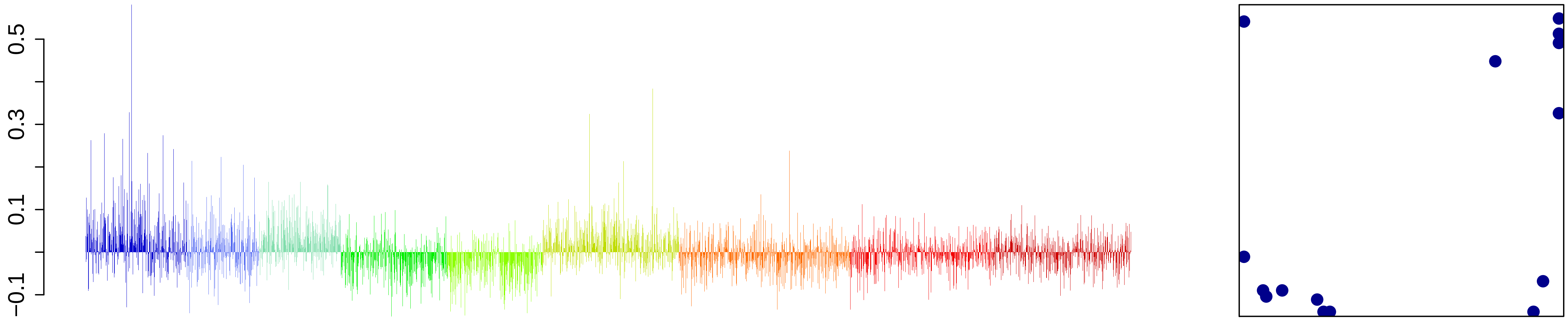

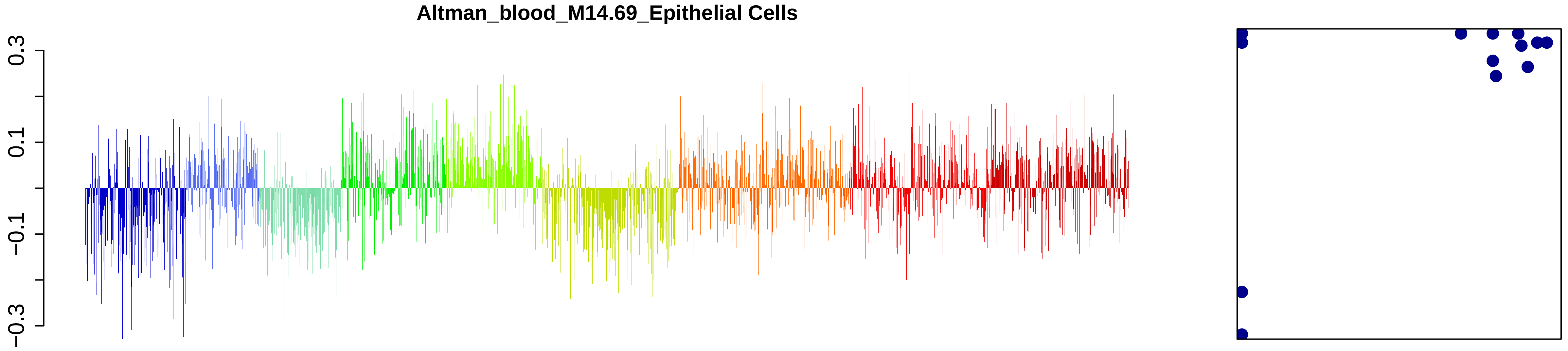
Altman_blood_M14.69_Epithelial Cells
0.3
0.1
−0.1
−0.3

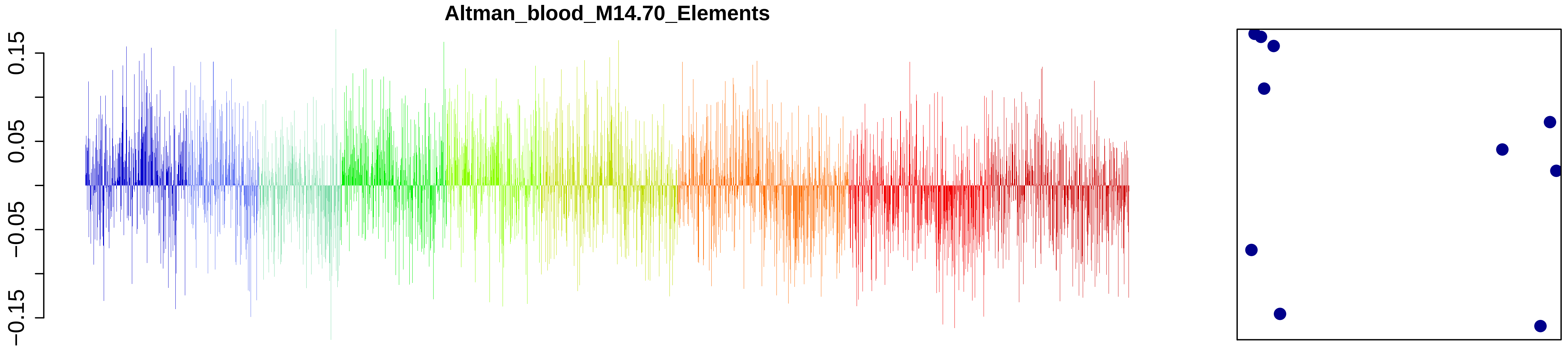
Altman_blood_M14.70_Elements
0.15
0.05
−0.05
−0.15

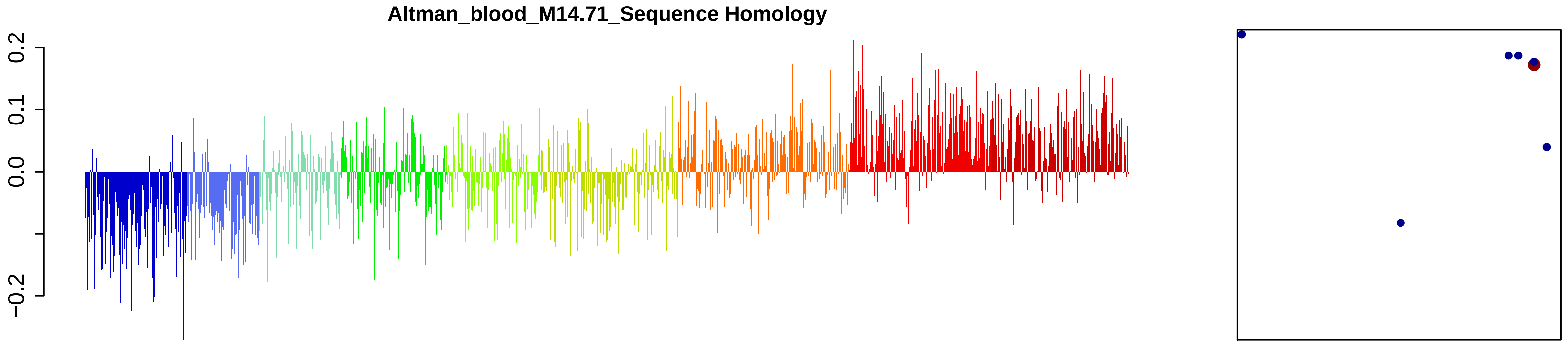
Altman_blood_M14.71_Sequence Homology
0.2
0.1
0.0
−0.2

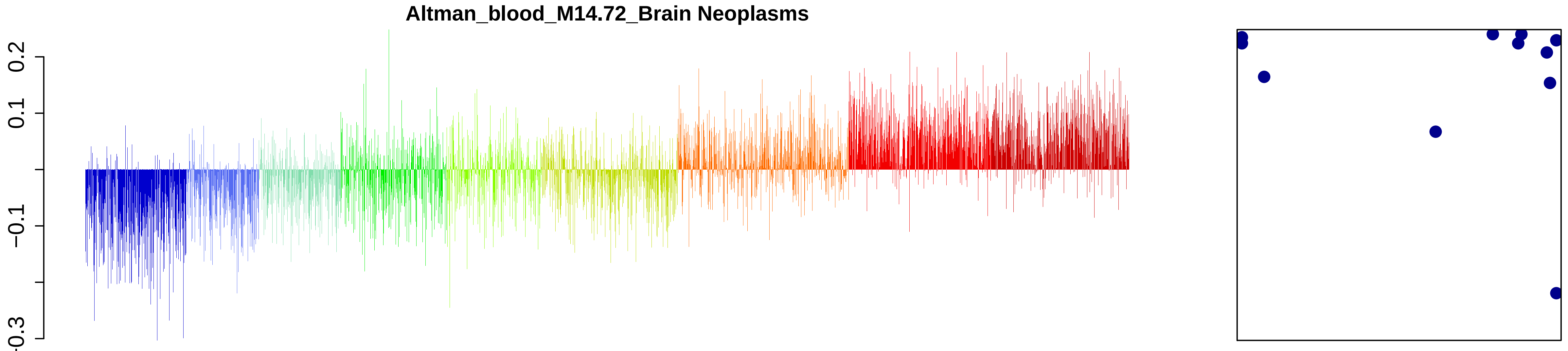
Altman_blood_M14.72_Brain Neoplasms
0.2
0.1
−0.1
−0.3

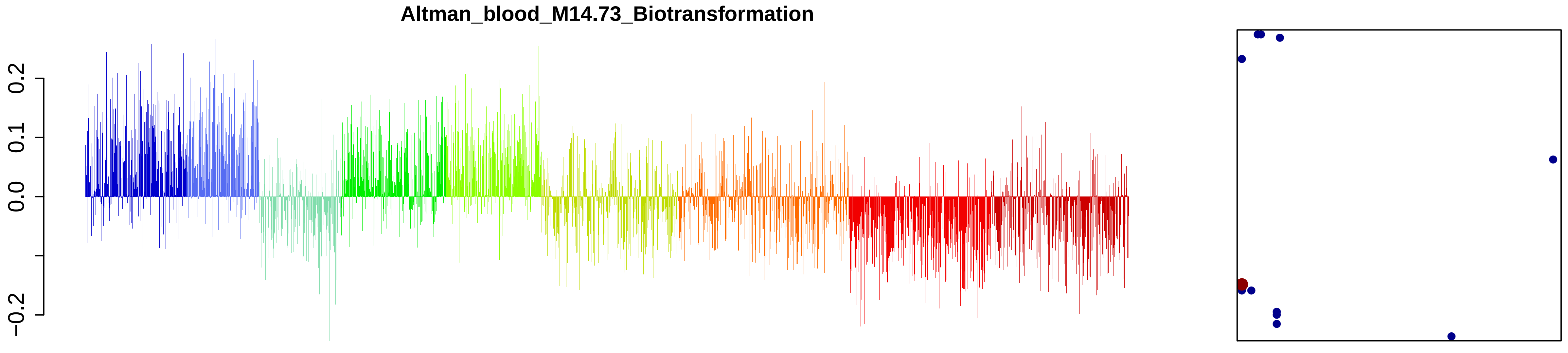
Altman_blood_M14.73_Biotransformation
0.2
0.1
0.0
−0.2

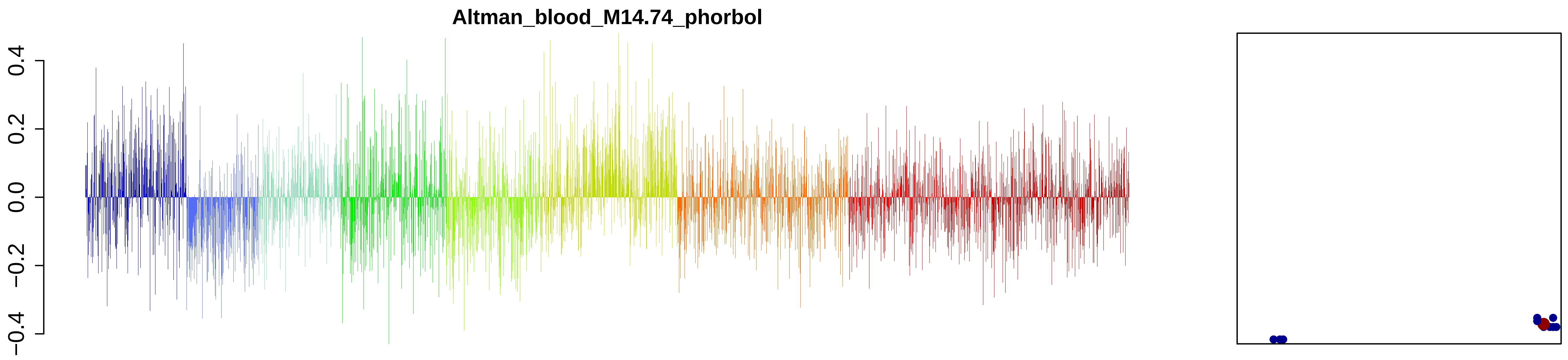

Altman_blood_M14.74_phorbol
0.4
0.2
0.0
−0.2
−0.4

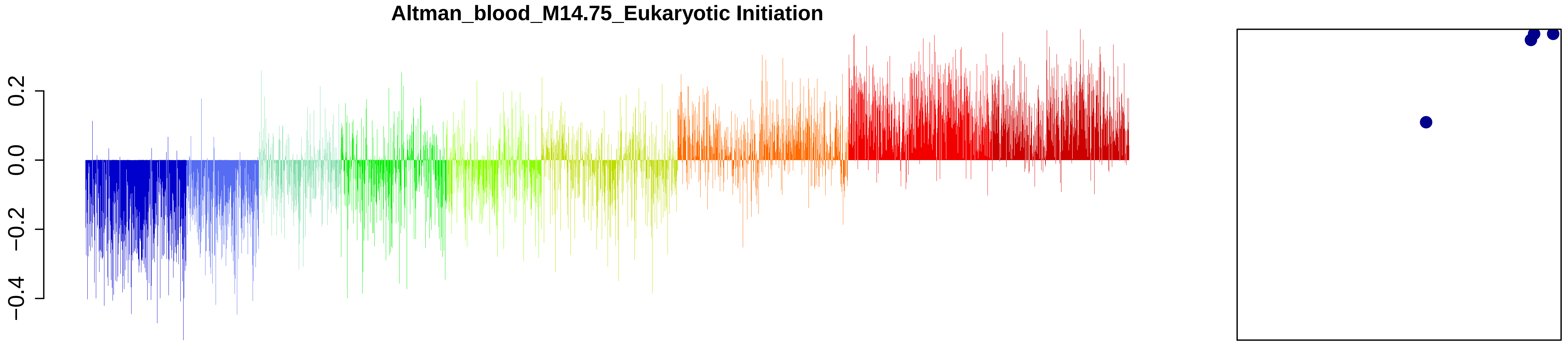

Altman_blood_M14.75_Eukaryotic Initiation
0.2
0.0
−0.2
−0.4

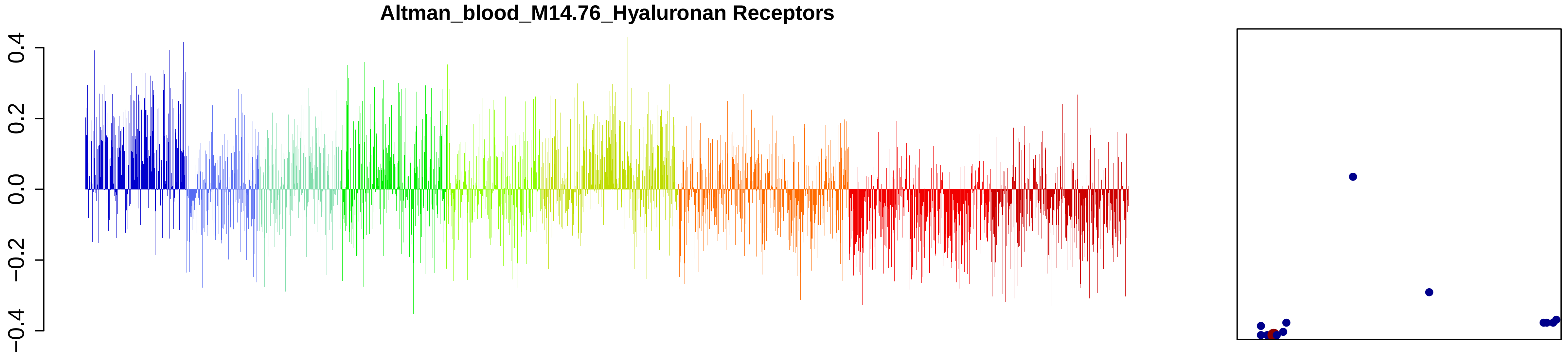

Altman_blood_M14.76_Hyaluronan Receptors
0.4
0.2
0.0
−0.2
−0.4

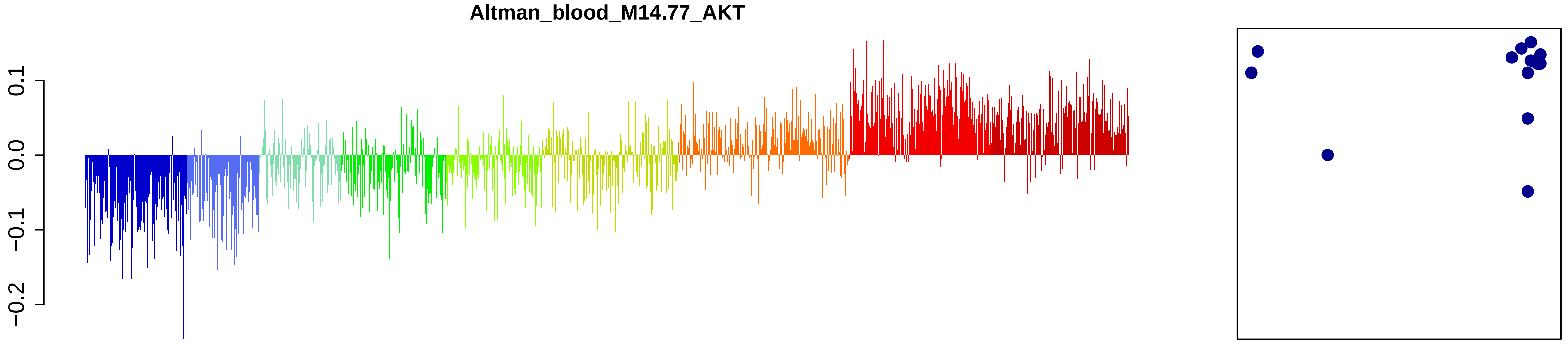

Altman_blood_M14.77_AKT
0.1
0.0
−0.1
−0.2

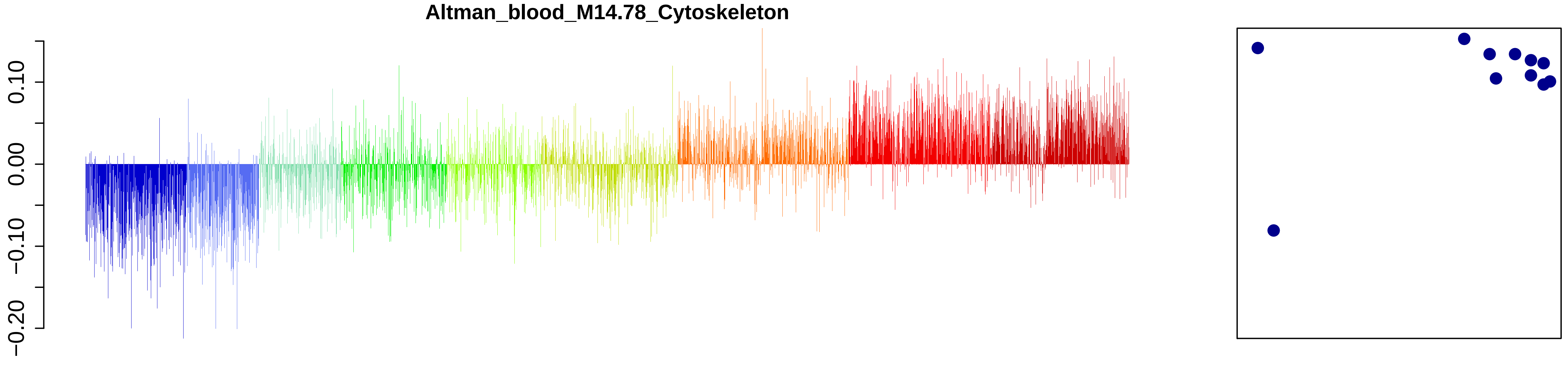

Altman_blood_M14.78_Cytoskeleton
0.10
0.00
−0.10
−0.20

Altman_blood_M14.79_Sequence Homology, Amino Acid

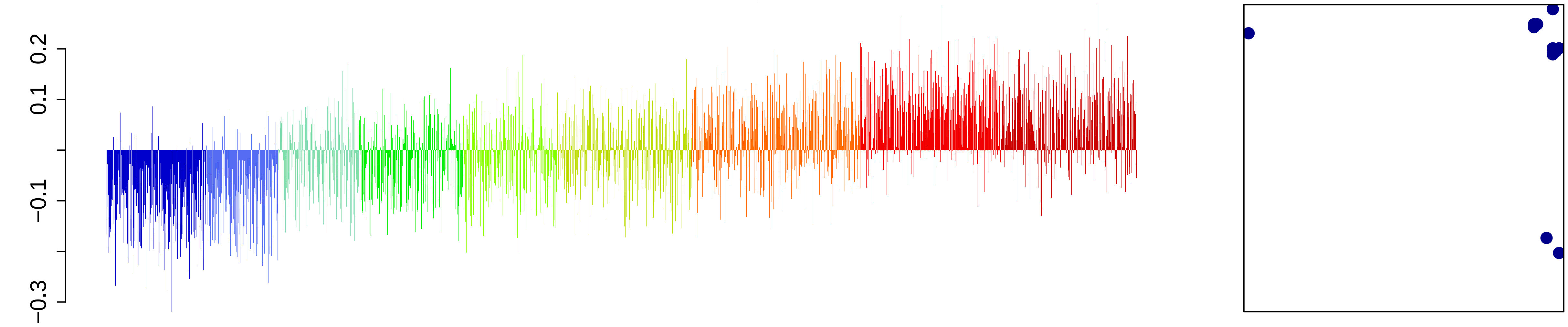

Altman_blood_M14.80_Biotransformation

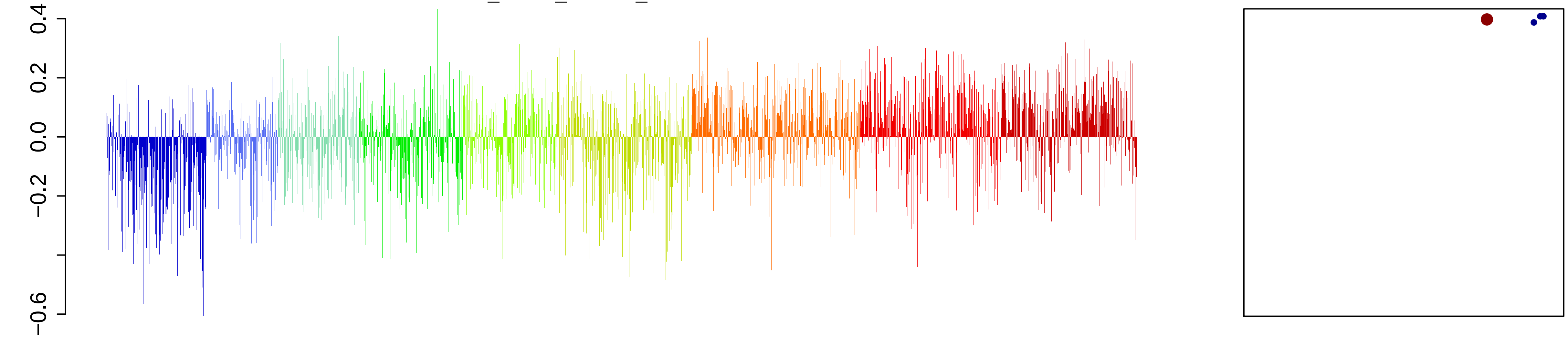

Altman_blood_M14.81_SN12C Cells

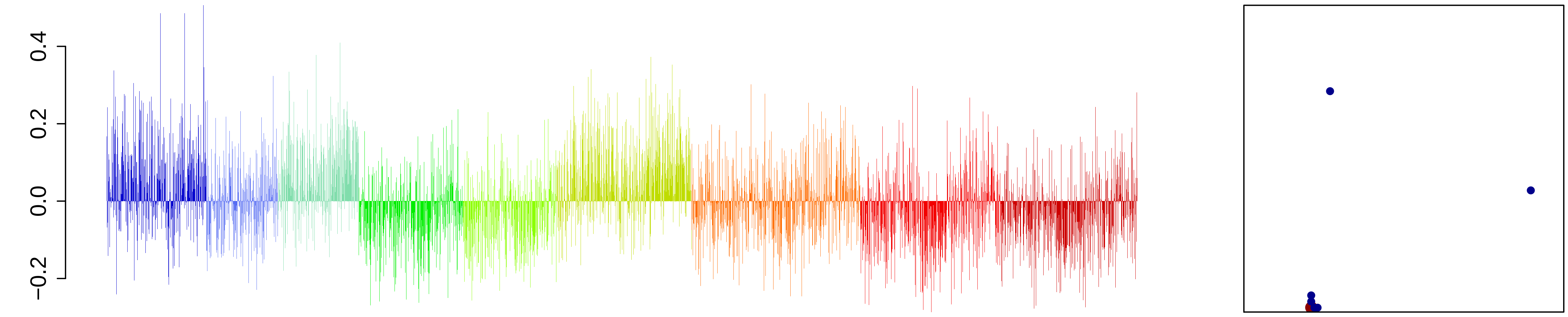

Altman_blood_M14.82_Rhombencephalon

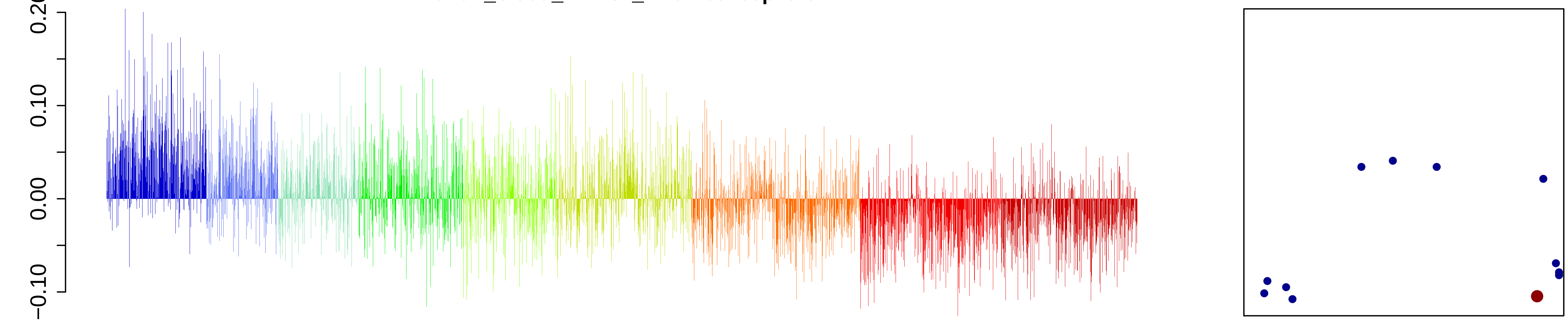

Altman_blood_M14.83_Histone Acetyltransferases

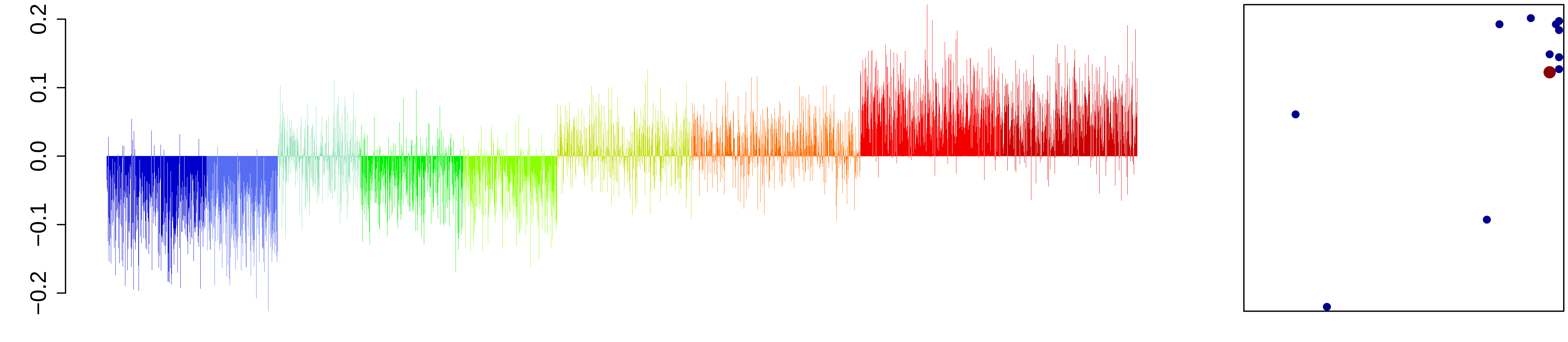

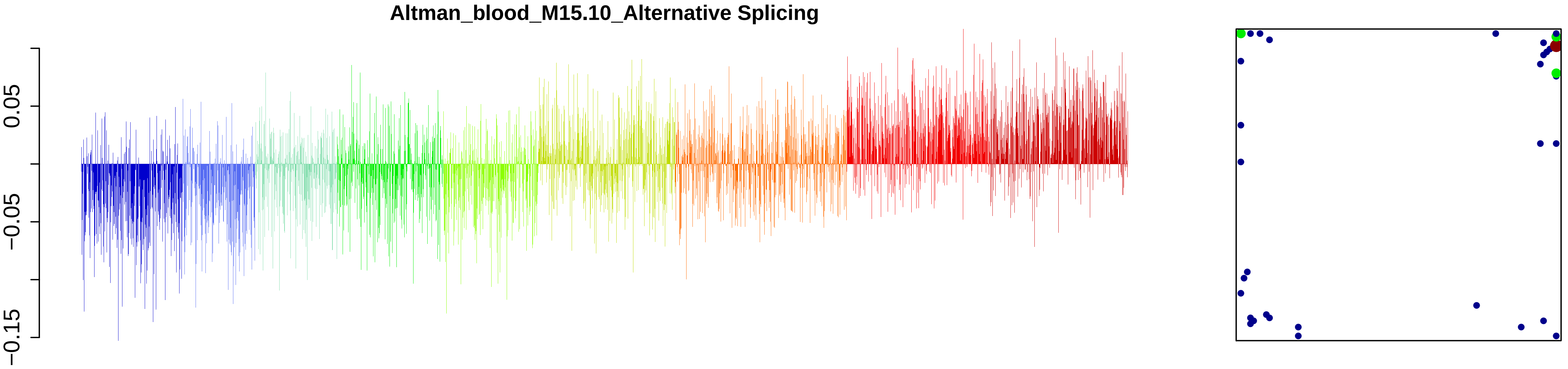
Altman_blood_M15.10_Alternative Splicing
0.05
−0.05
−0.15

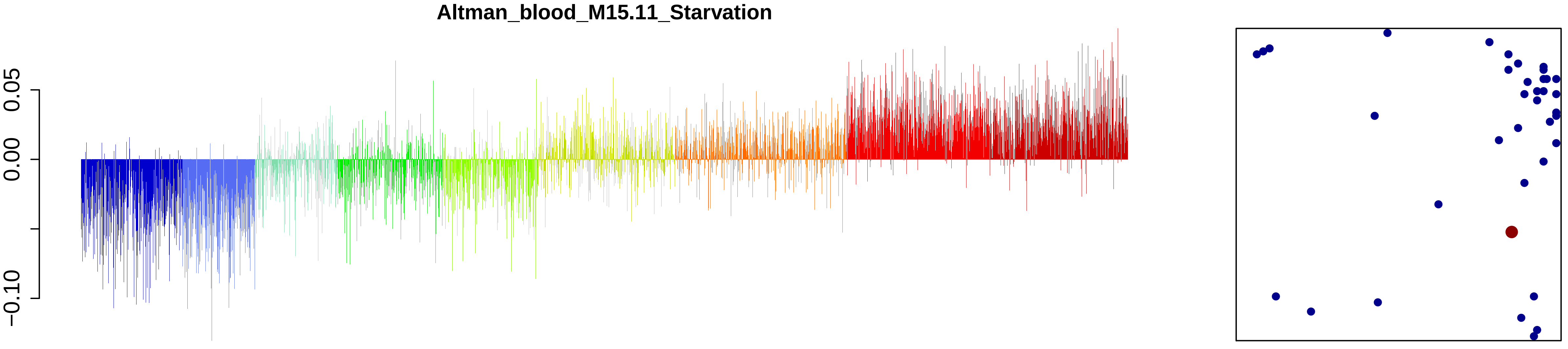
Altman_blood_M15.11_Starvation
0.05
0.00
−0.10

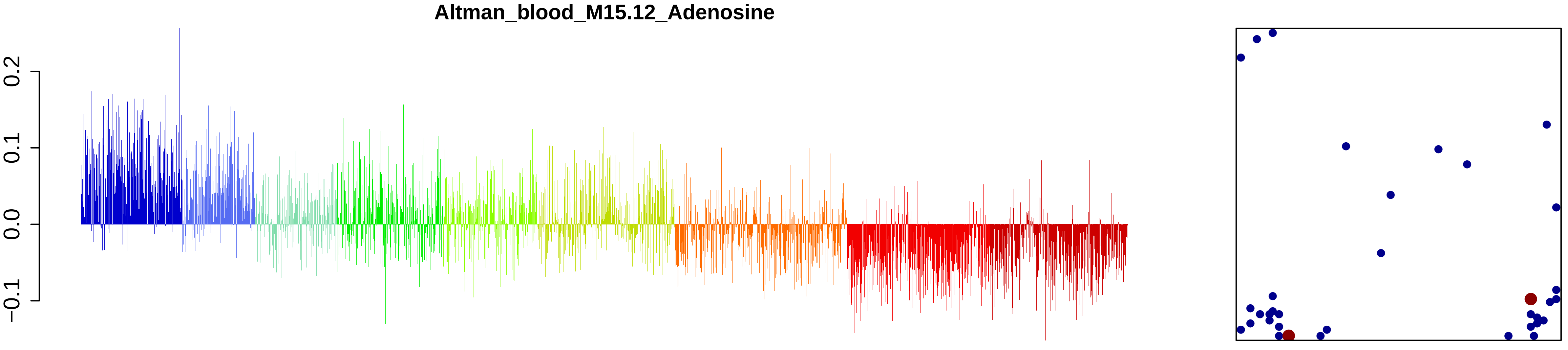
Altman_blood_M15.12_Adenosine
0.2
0.1
0.0
−0.1

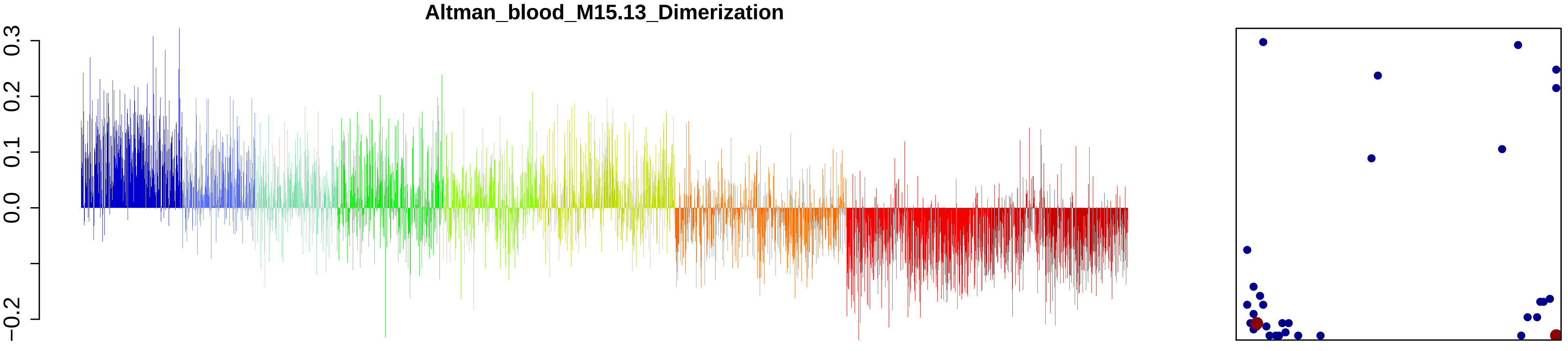
Altman_blood_M15.13_Dimerization
0.3
0.2
0.1
0.0
−0.2

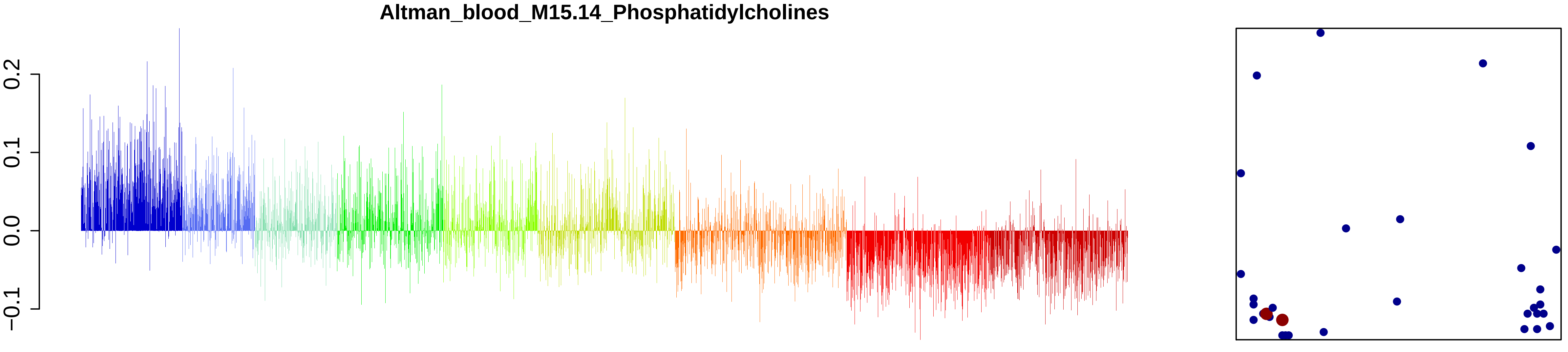
Altman_blood_M15.14_Phosphatidylcholines
0.2
0.1
0.0
−0.1

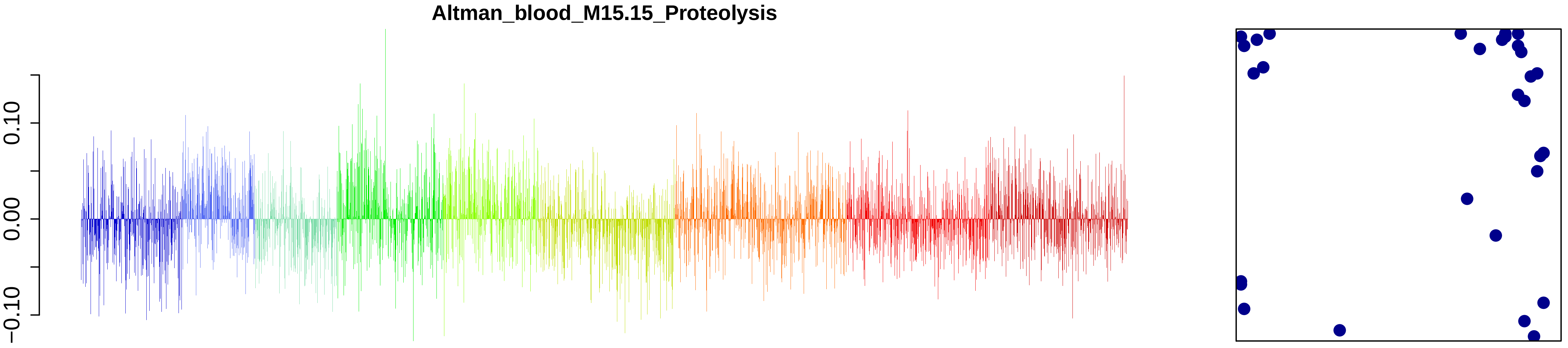
Altman_blood_M15.15_Proteolysis
0.10
0.00
−0.10

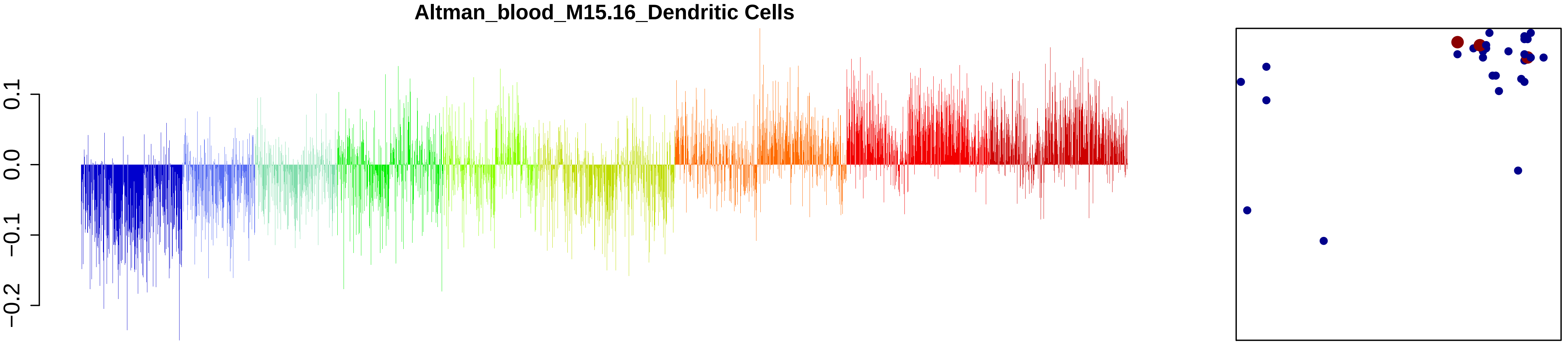
Altman_blood_M15.16_Dendritic Cells
0.1
0.0
−0.1
−0.2

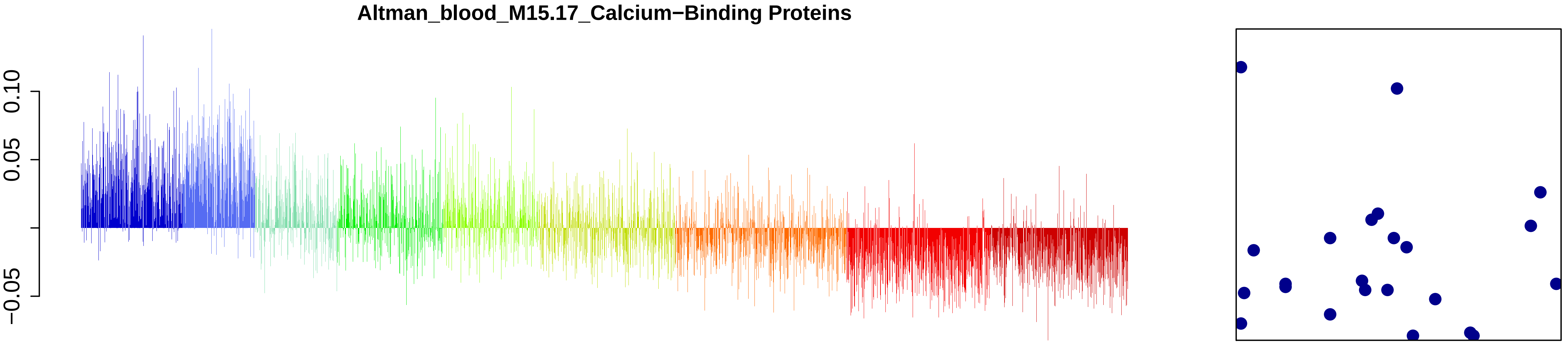
Altman_blood_M15.17_Calcium−Binding Proteins
0.10
0.05
−0.05

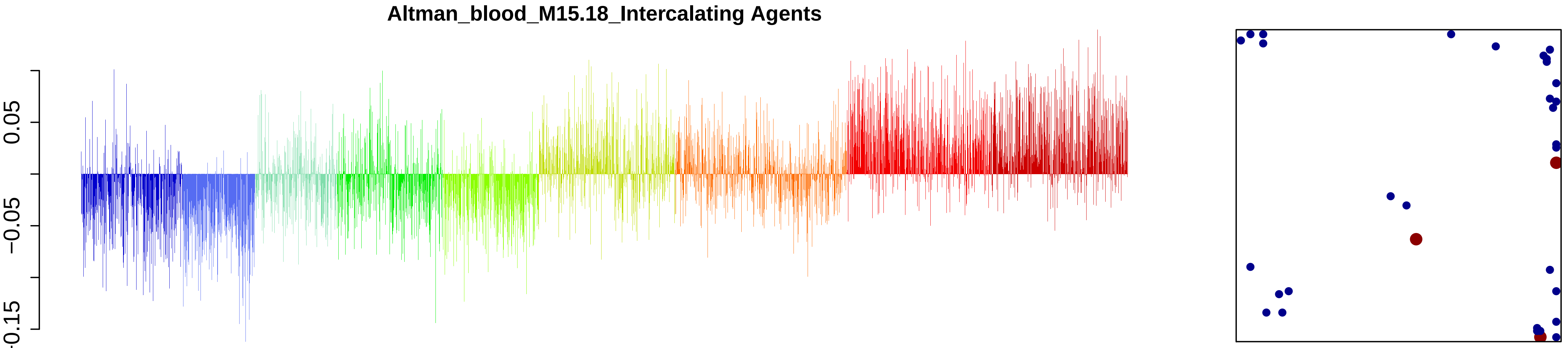
Altman_blood_M15.18_Intercalating Agents
0.05
−0.05
−0.15

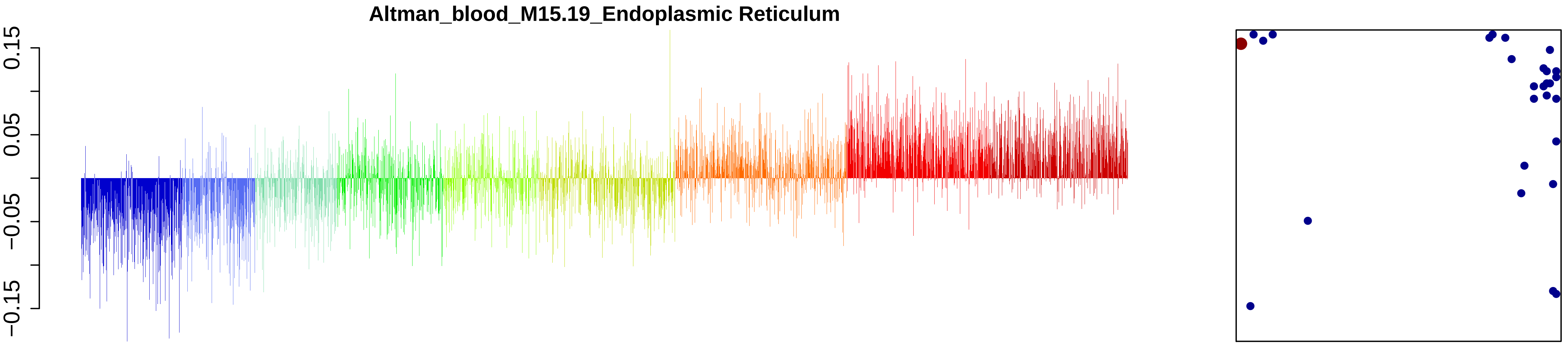
Altman_blood_M15.19_Endoplasmic Reticulum
0.15
0.05
−0.05
−0.15

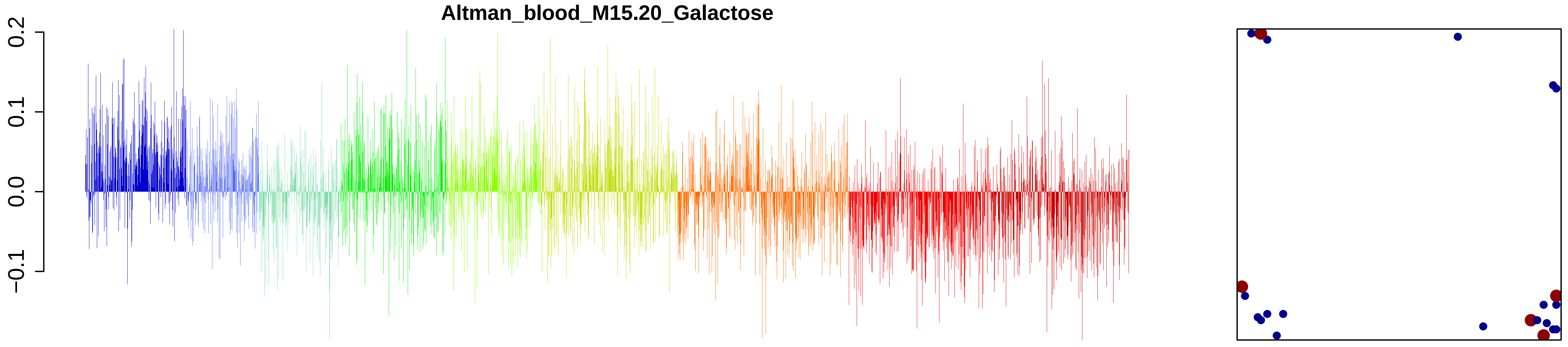
Altman_blood_M15.20_Galactose
0.2
0.1
0.0
−0.1

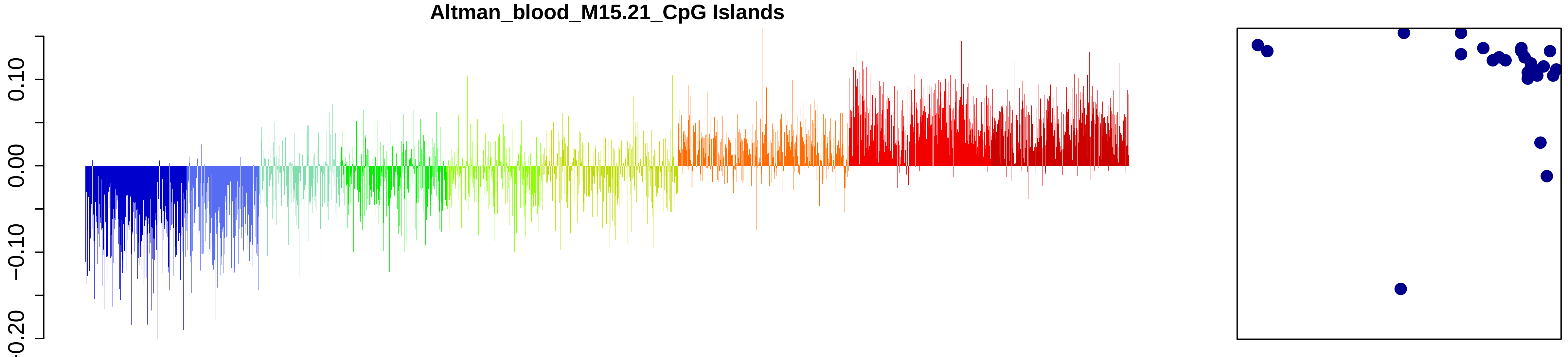
Altman_blood_M15.21_CpG Islands
0.10
0.00
−0.10
−0.20

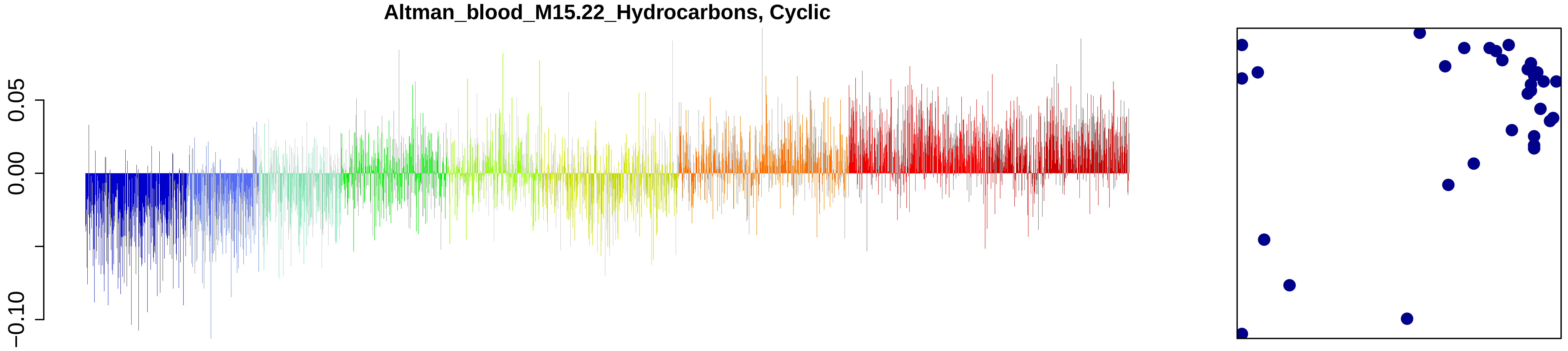
Altman_blood_M15.22_Hydrocarbons, Cyclic
0.05
0.00
−0.10

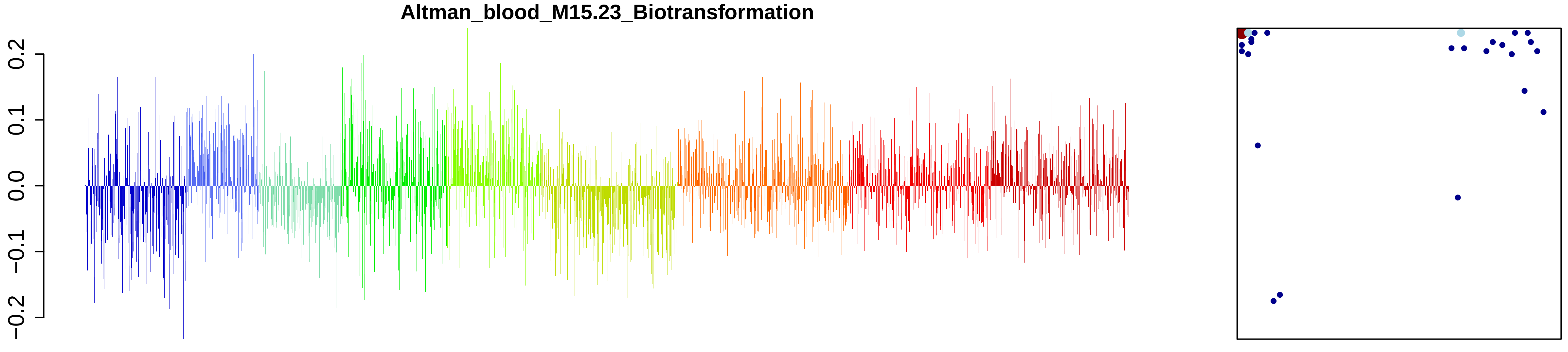
Altman_blood_M15.23_Biotransformation
0.2
0.1
0.0
−0.1
−0.2

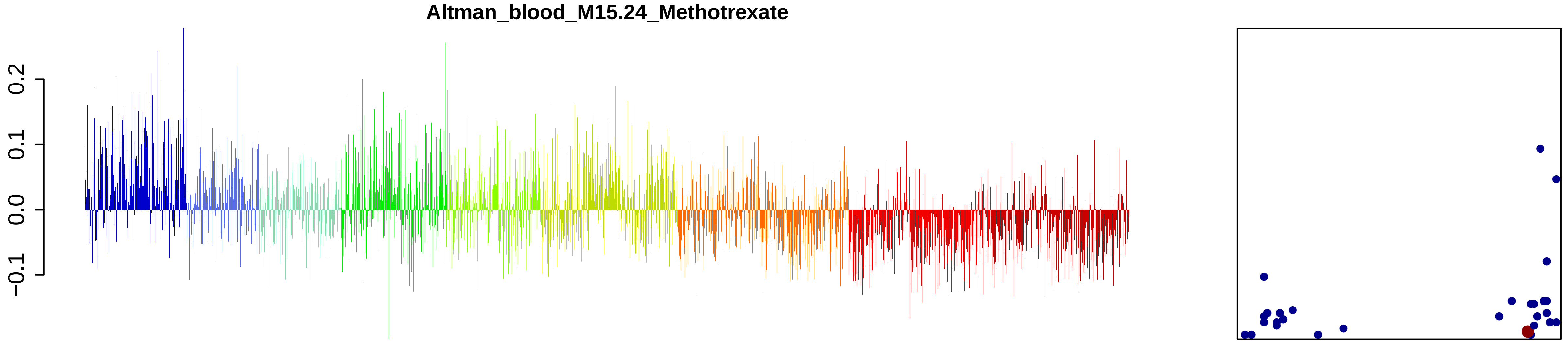
Altman_blood_M15.24_Methotrexate
0.2
0.1
0.0
−0.1

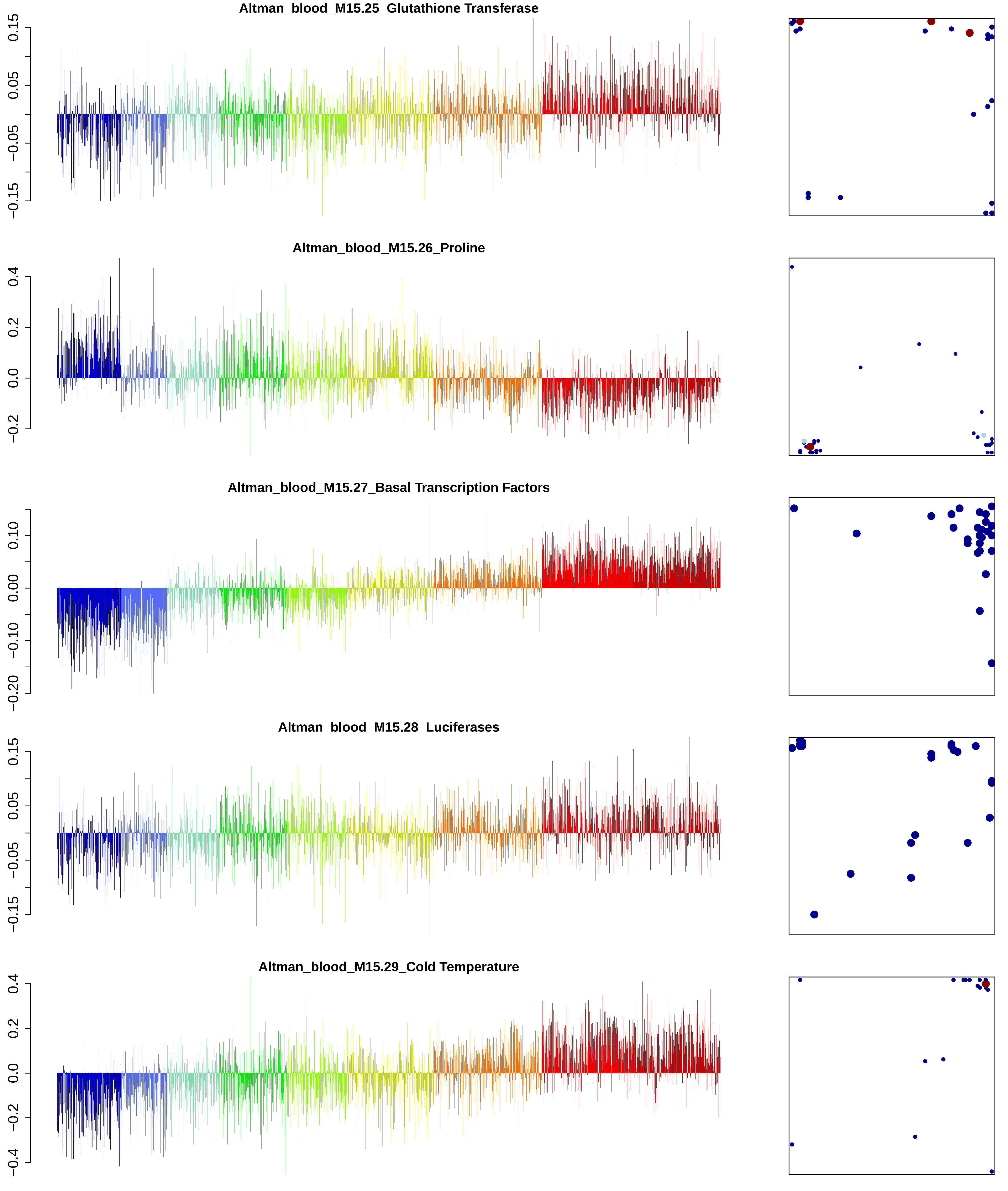

Altman_blood_M15.25_Glutathione Transferase
0.15
0.05
−0.05
−0.15
Altman_blood_M15.26_Proline
0.4
0.2
0.0
−0.2
Altman_blood_M15.27_Basal Transcription Factors
0.10
0.00
−0.10
−0.20
Altman_blood_M15.28_Luciferases
0.15
0.05
−0.05
−0.15
Altman_blood_M15.29_Cold Temperature
0.4
0.2
0.0
−0.2
−0.4

Altman_blood_M15.30_SMAD2

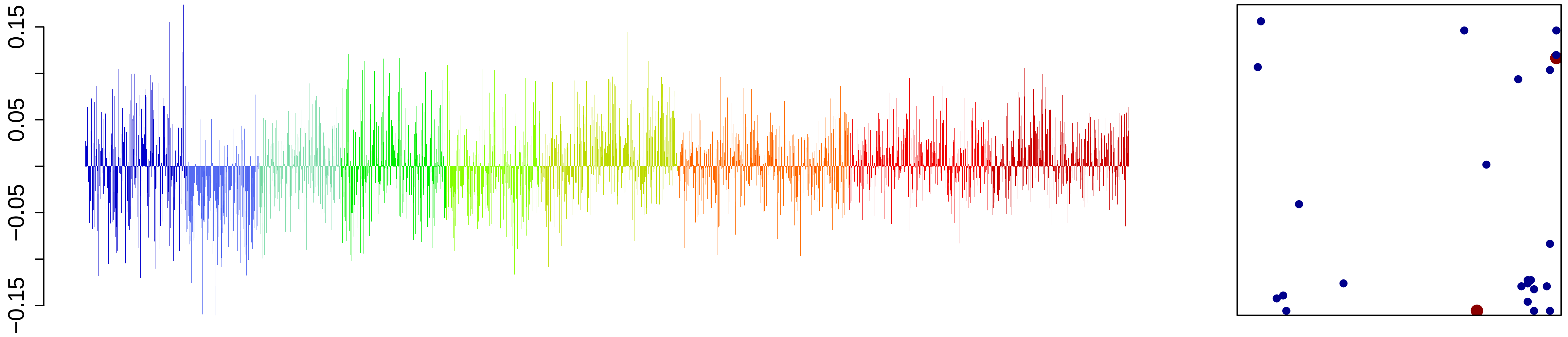

Altman_blood_M15.31_Antigens, Nuclear

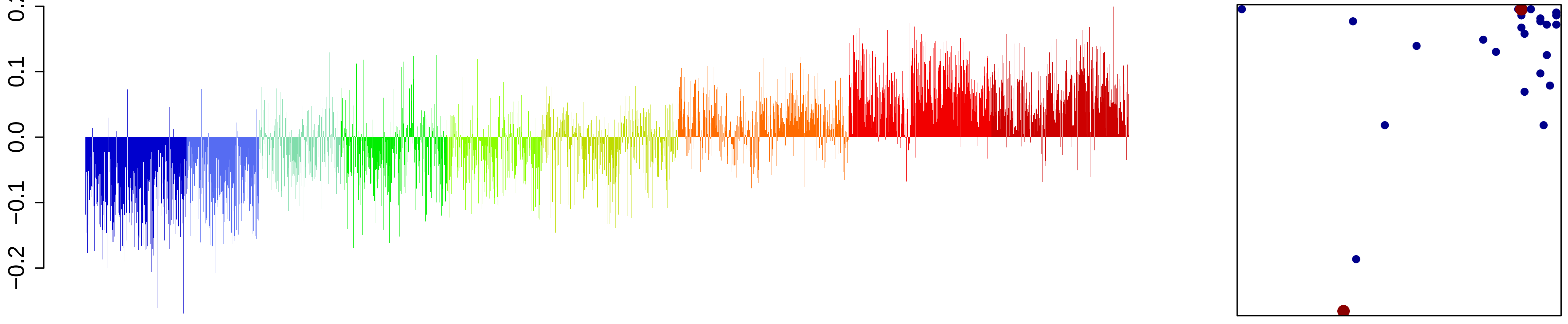

Altman_blood_M15.32_Therapeutic Uses

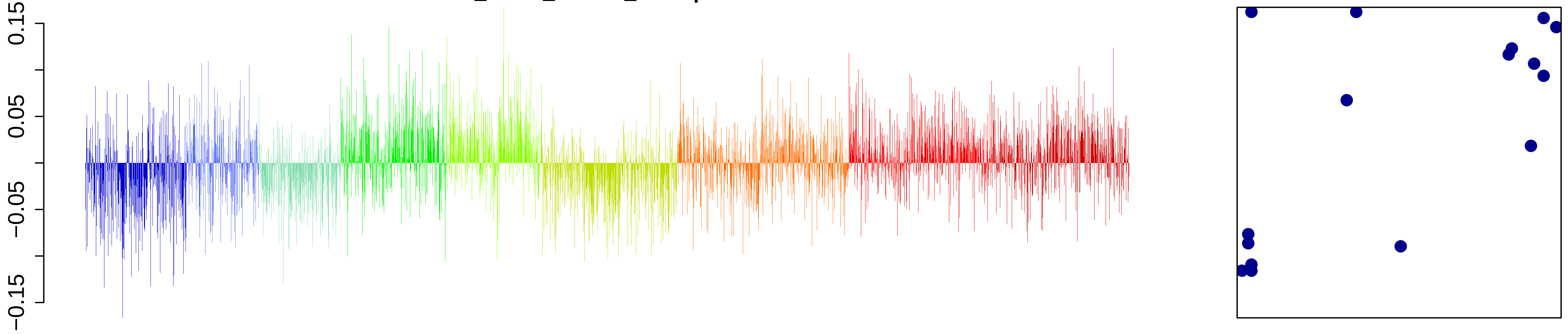

Altman_blood_M15.33_Connective Tissue Diseases

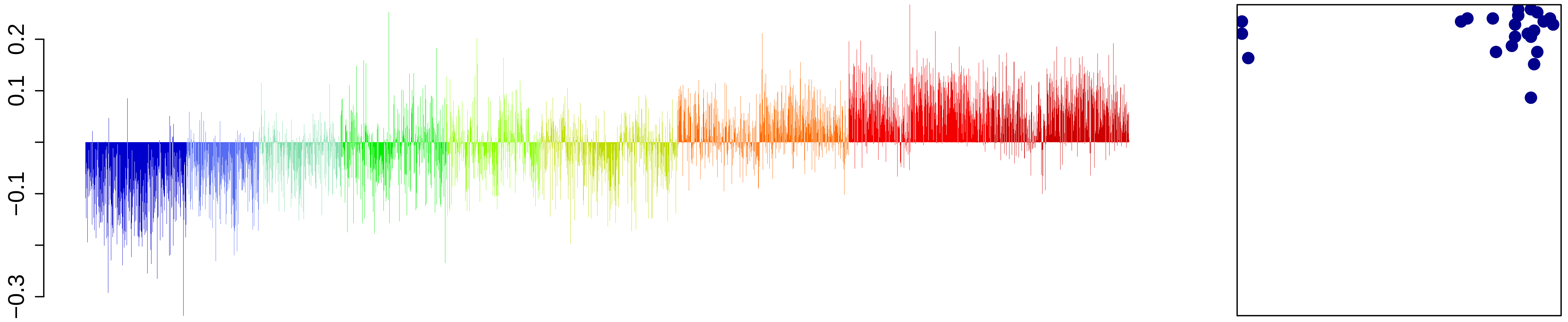

Altman_blood_M15.34_RNA Interference

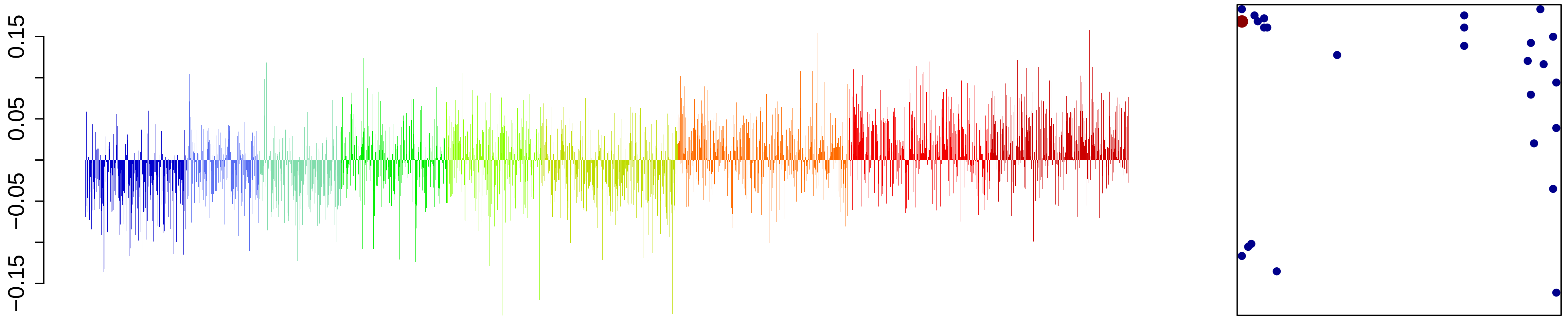

Altman_blood_M15.35_HLA−B Antigens

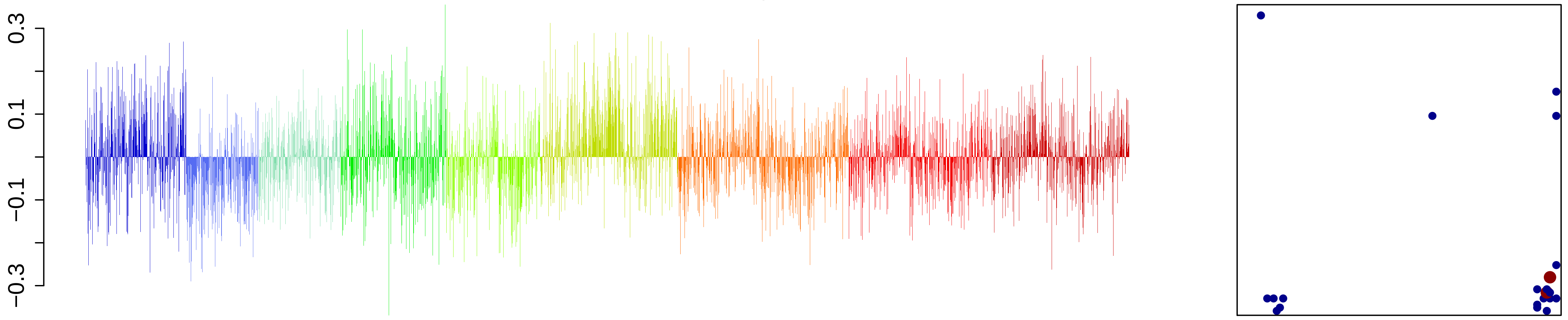

Altman_blood_M15.36_Biotransformation

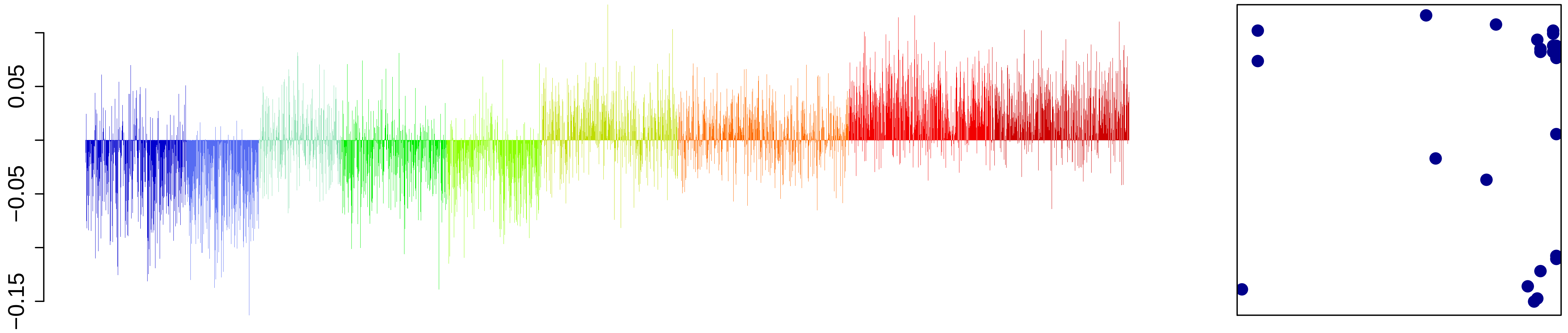

Altman_blood_M15.37_Free Radical Scavengers

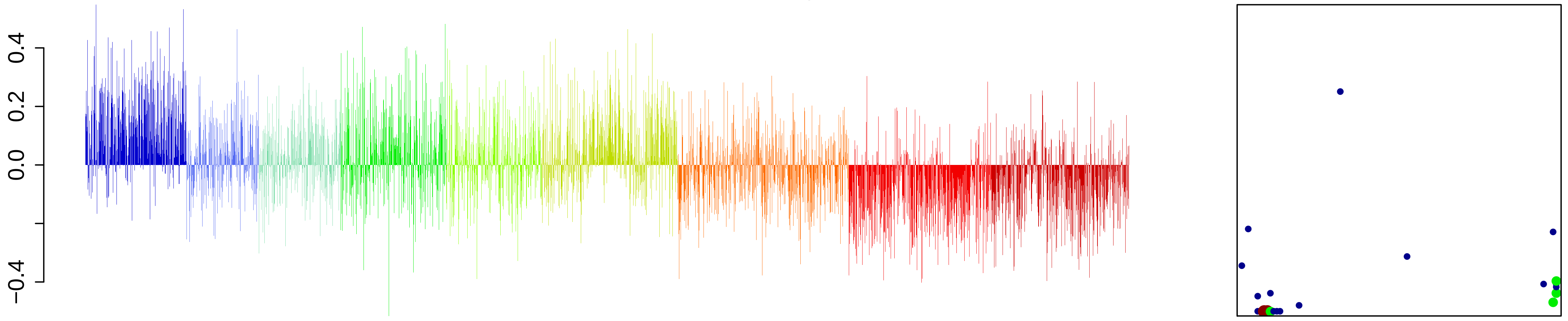

Altman_blood_M15.38_Signaling Lymphocytic Activation Molecule Family

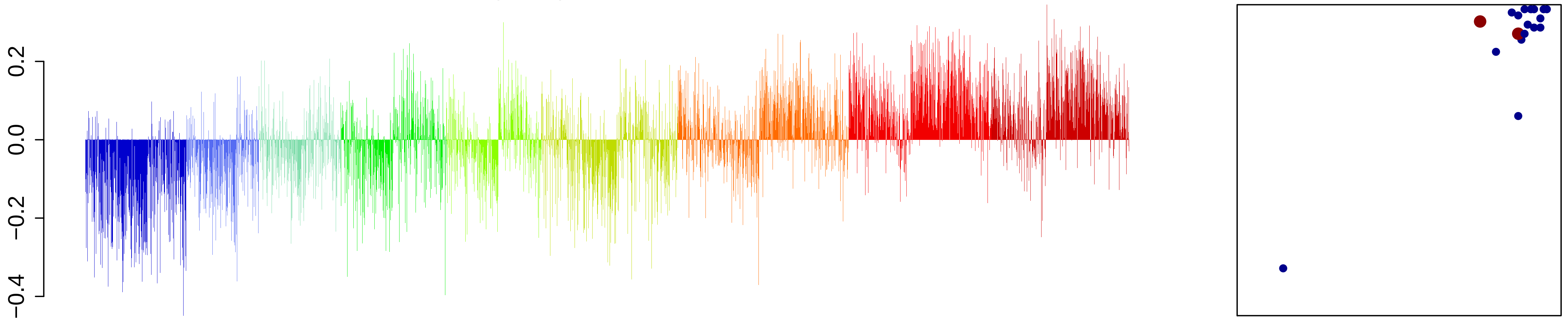

Altman_blood_M15.39_Lymphotoxin−beta

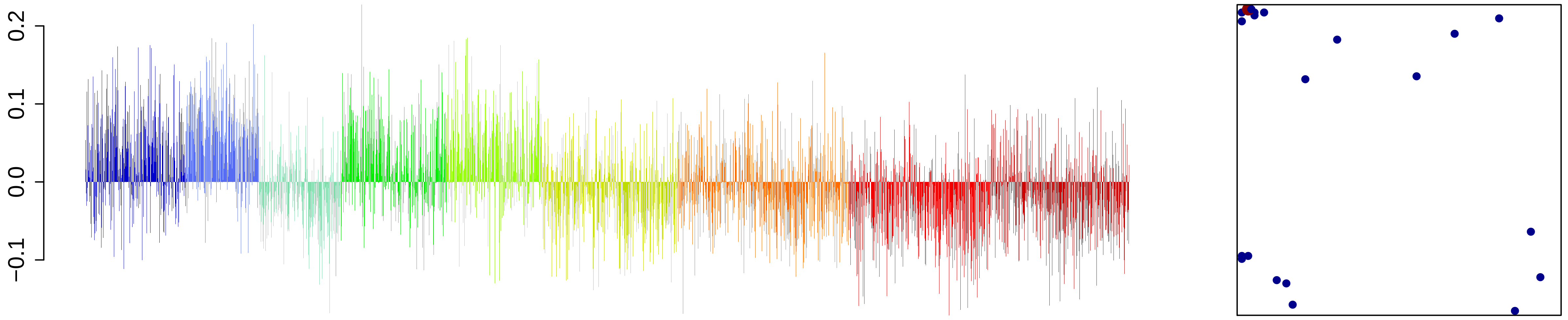

Altman_blood_M15.40_Biochemical Phenomena

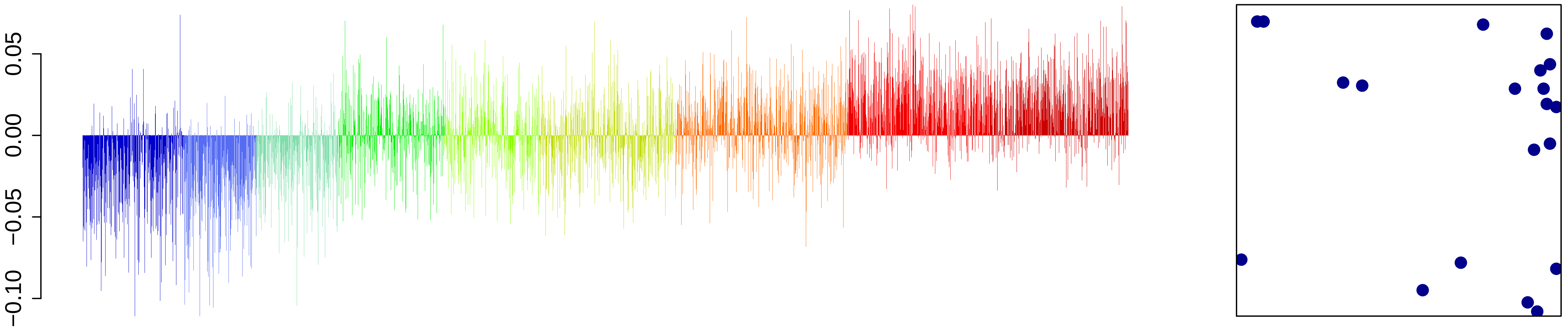

Altman_blood_M15.41_Antibodies

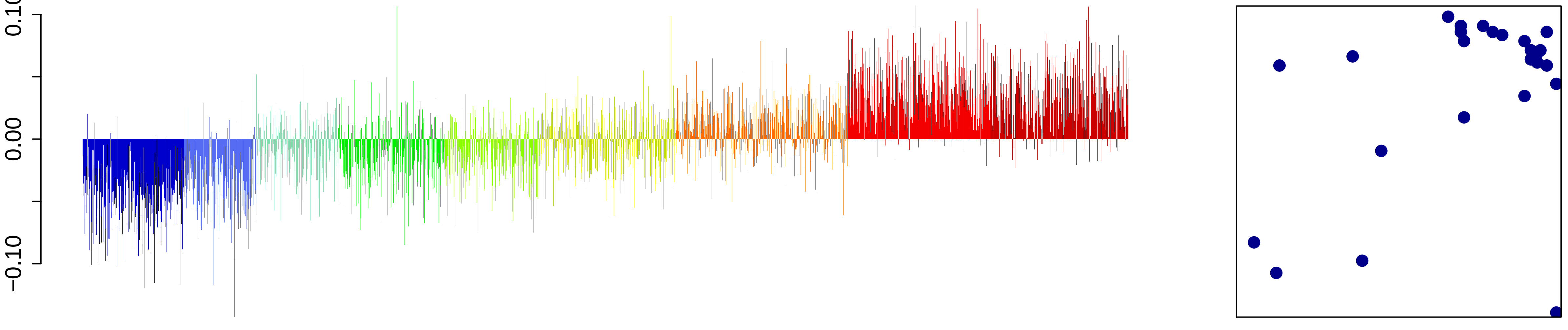

Altman_blood_M15.42_Oncogenes

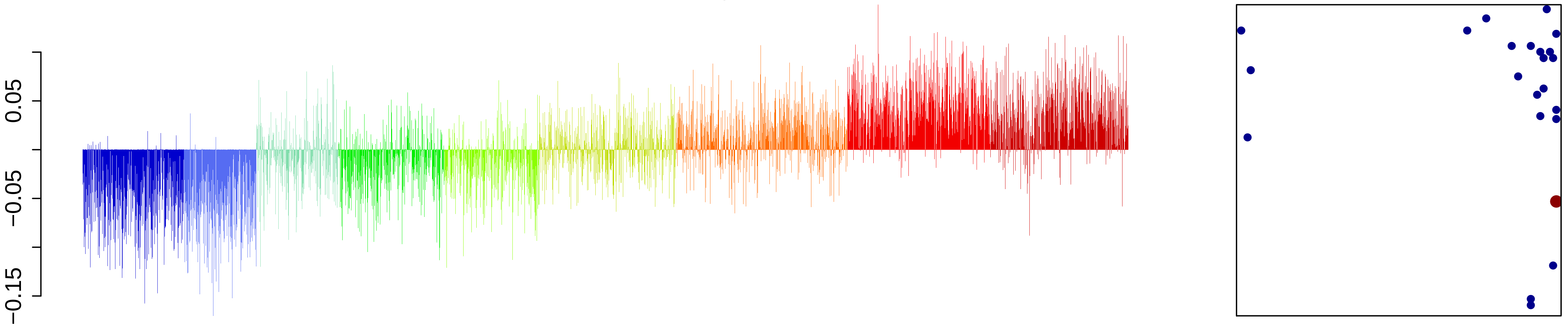

Altman_blood_M15.43_Amyloidosis

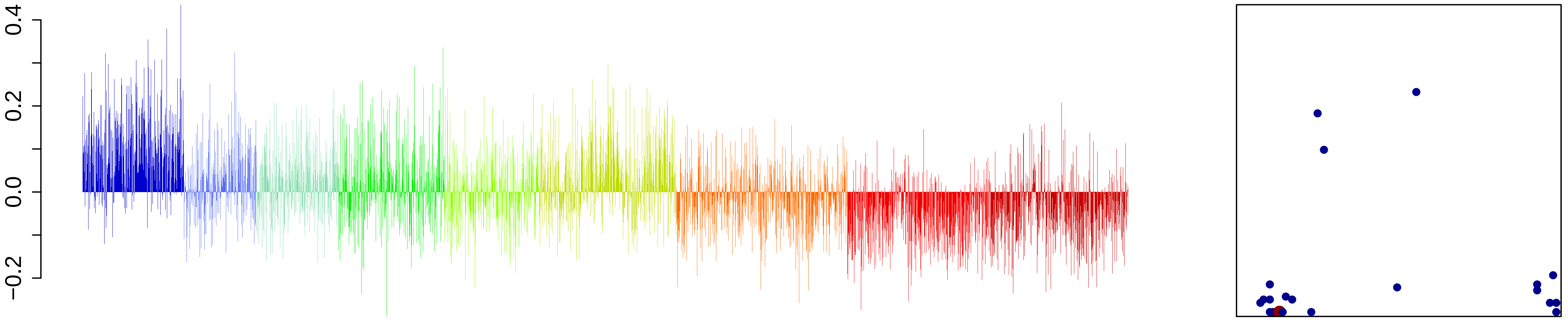

Altman_blood_M15.44_RNA, Messenger

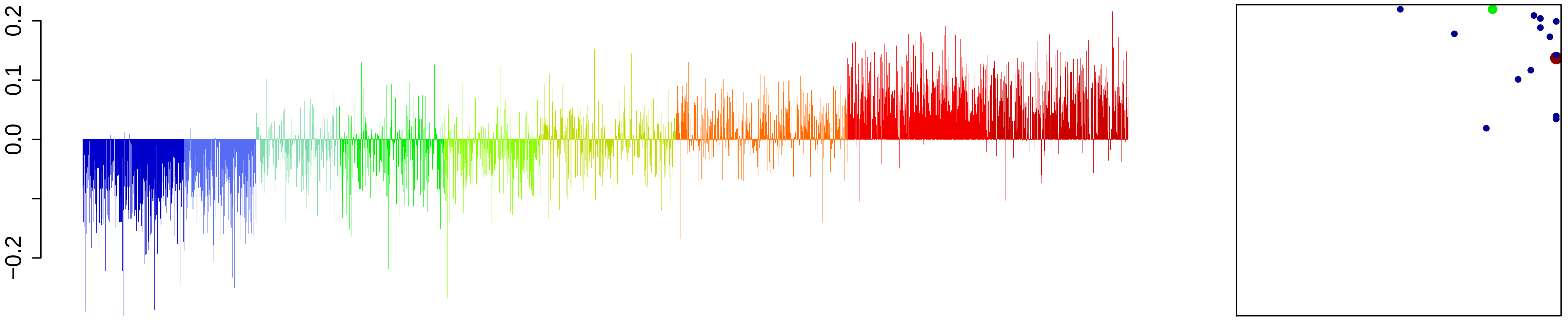

Altman_blood_M15.45_Platelet Membrane Glycoproteins

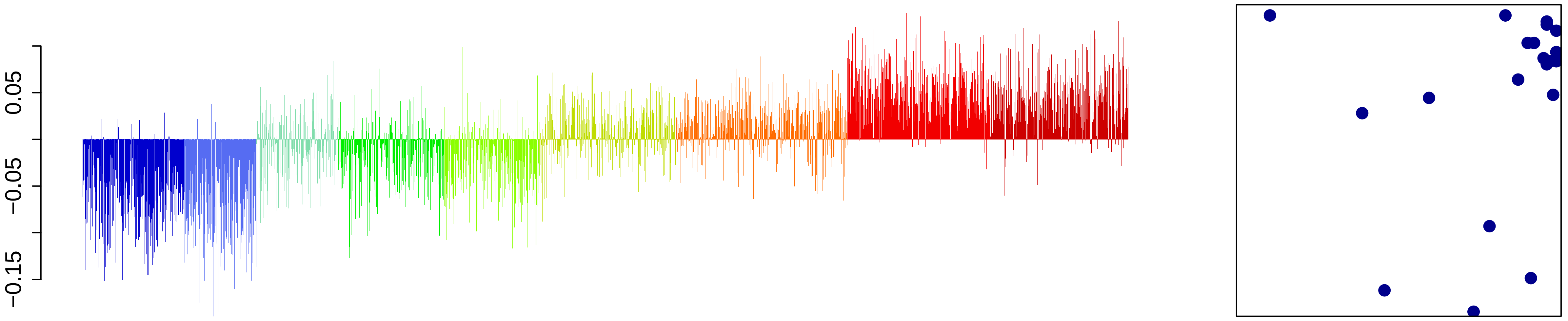

Altman_blood_M15.46_Metabolic Networks and Pathways

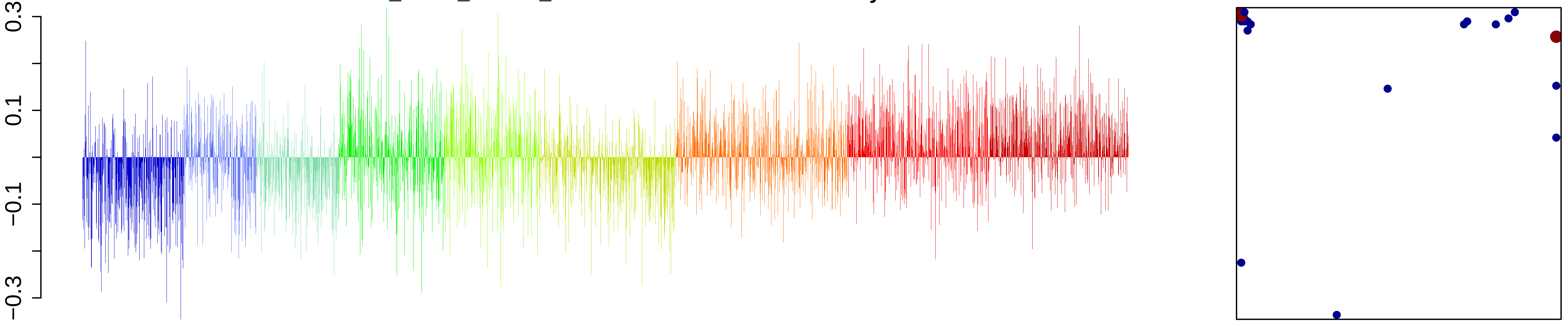

Altman_blood_M15.47_Genetic Loci

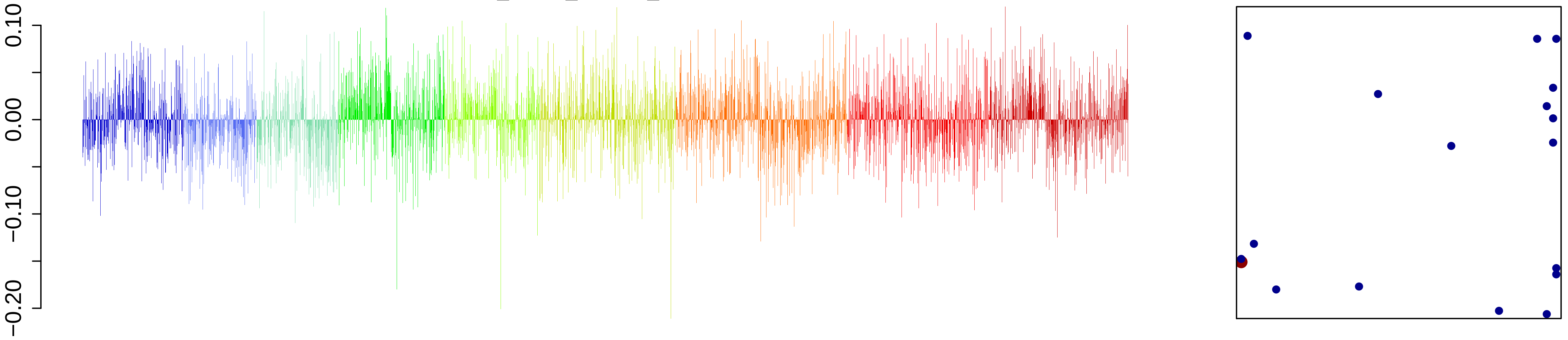

Altman_blood_M15.48_Cell Division

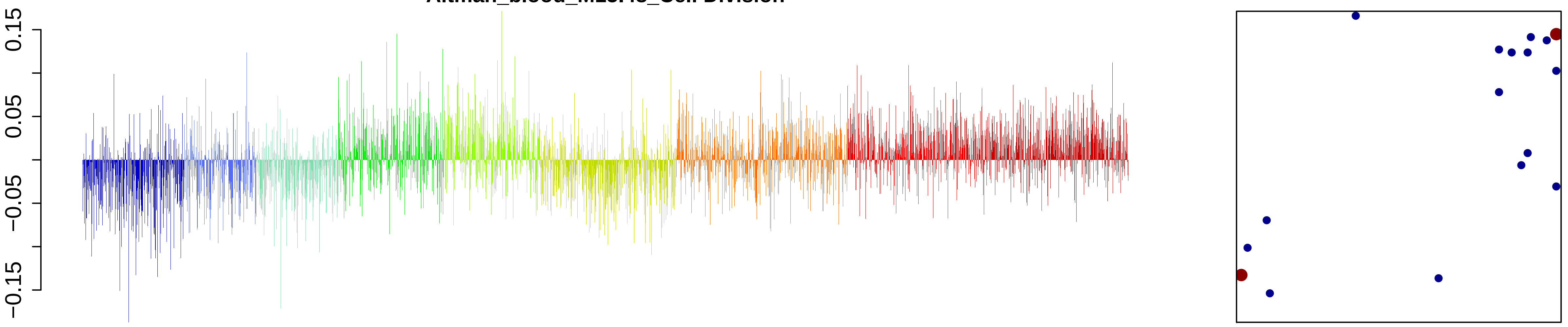

Altman_blood_M15.49_SN12C Cells

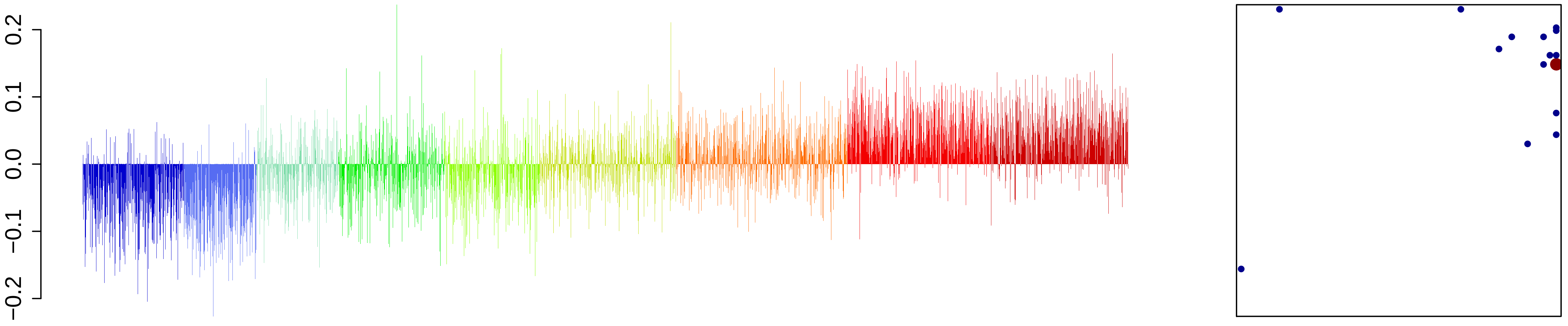

Altman_blood_M15.50_Arthropod Proteins

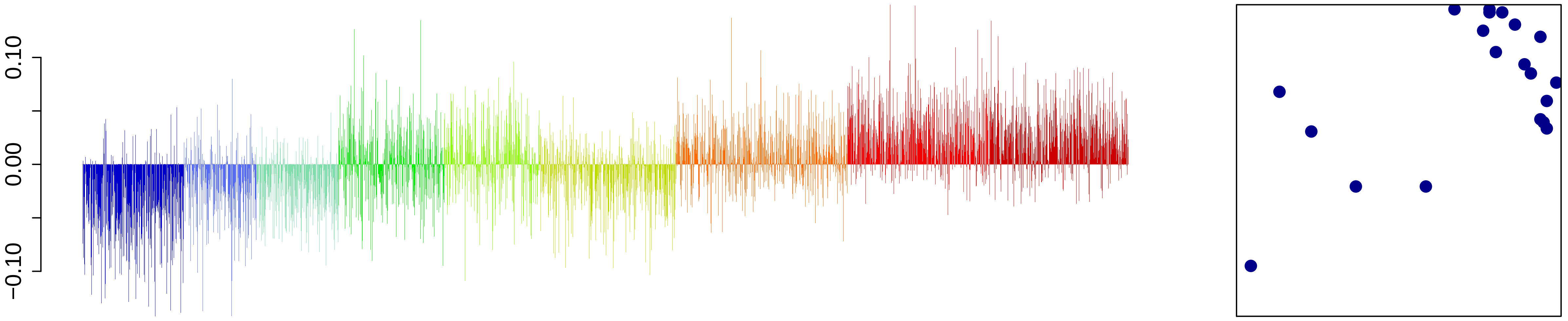

Altman_blood_M15.51_5 Untranslated Regions

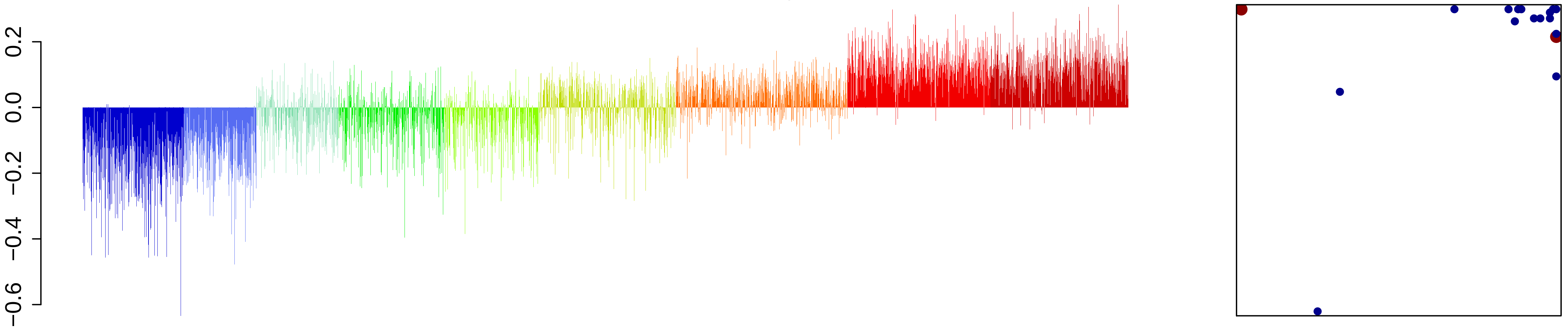

Altman_blood_M15.52_Biotransformation

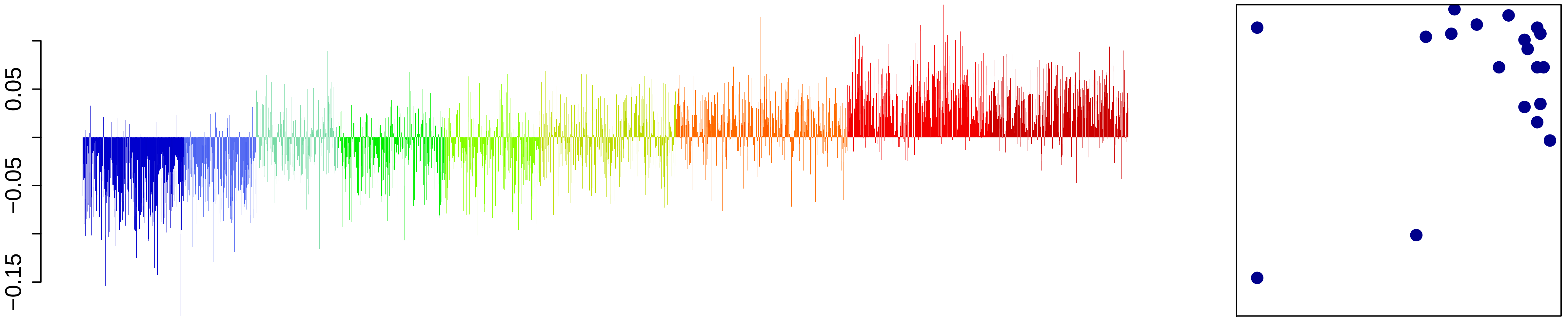

Altman_blood_M15.53_Erythrocytes

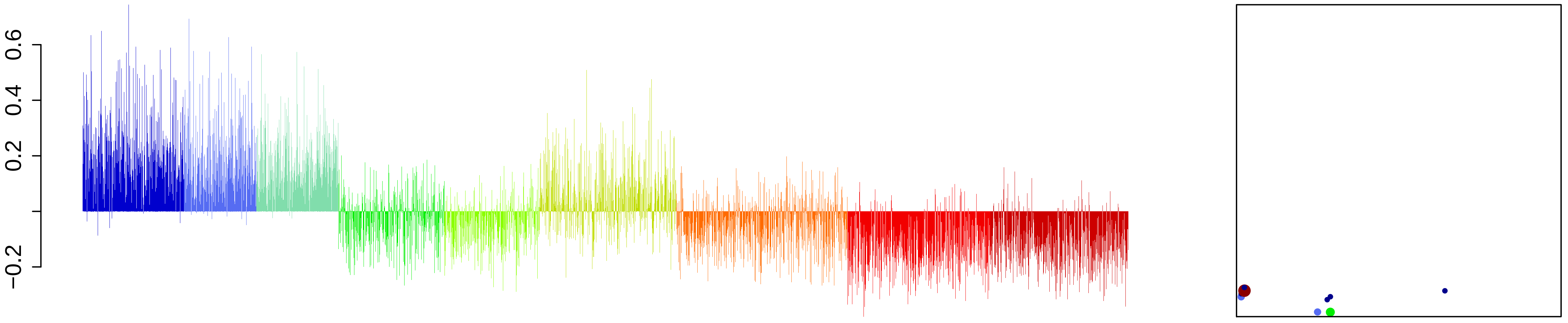

Altman_blood_M15.54_Ethanolamine

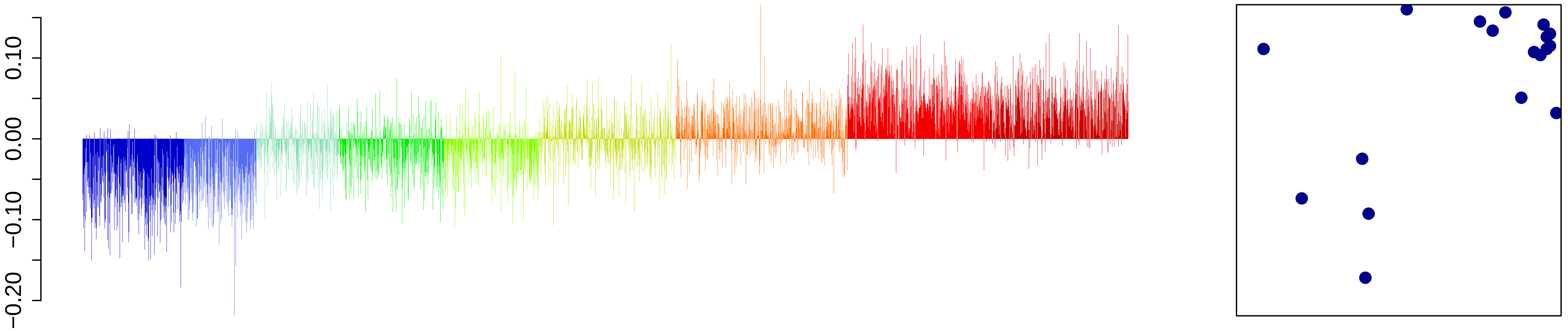

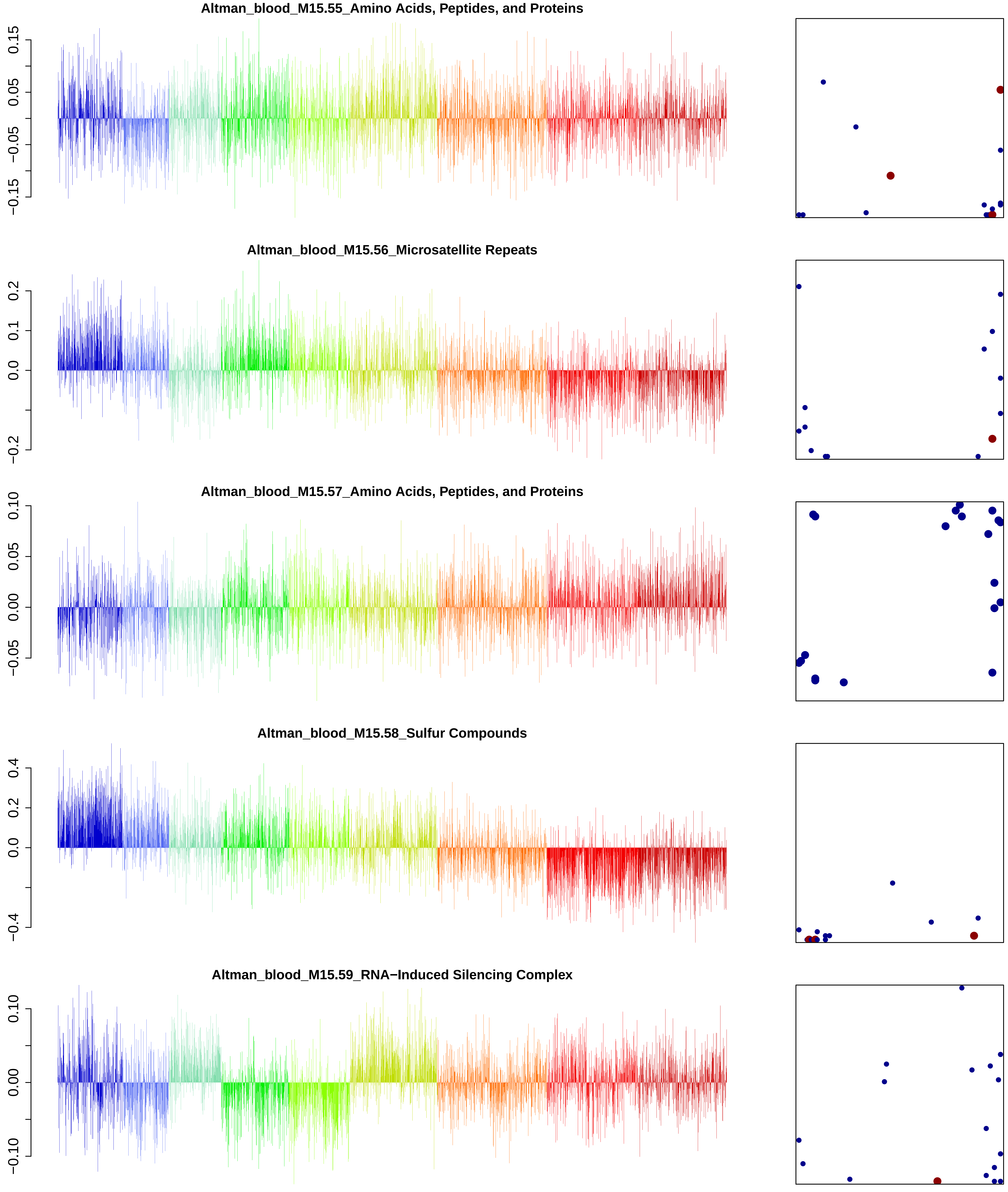
Altman_blood_M15.55_Amino Acids, Peptides, and Proteins
0.15
0.05
−0.05
−0.15
Altman_blood_M15.56_Microsatellite Repeats
0.2
0.1
0.0
−0.2
Altman_blood_M15.57_Amino Acids, Peptides, and Proteins
0.10
0.05
0.00
−0.05
Altman_blood_M15.58_Sulfur Compounds
0.4
0.2
0.0
−0.4
Altman_blood_M15.59_RNA−Induced Silencing Complex
0.10
0.00
−0.10

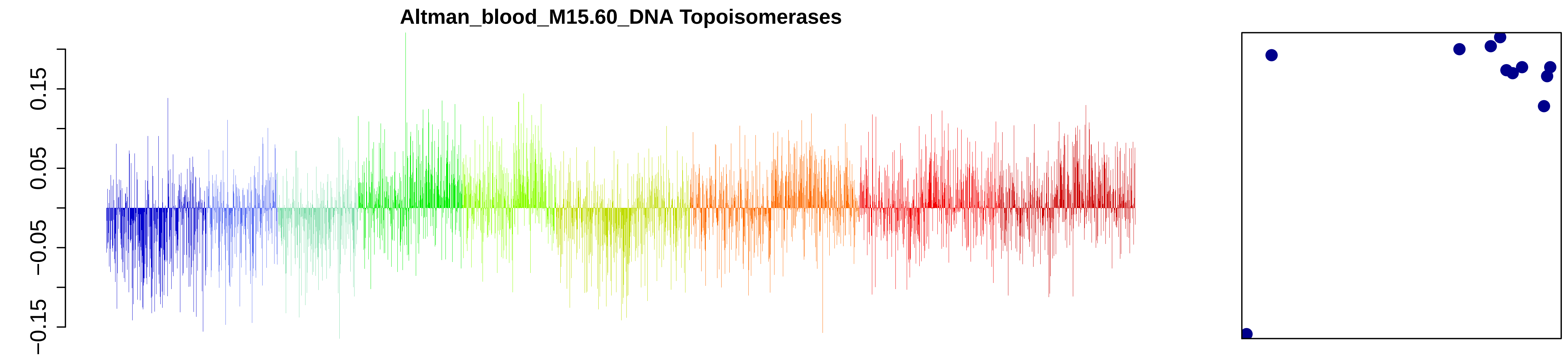
Altman_blood_M15.60_DNA Topoisomerases
0.15
0.05
−0.05
−0.15
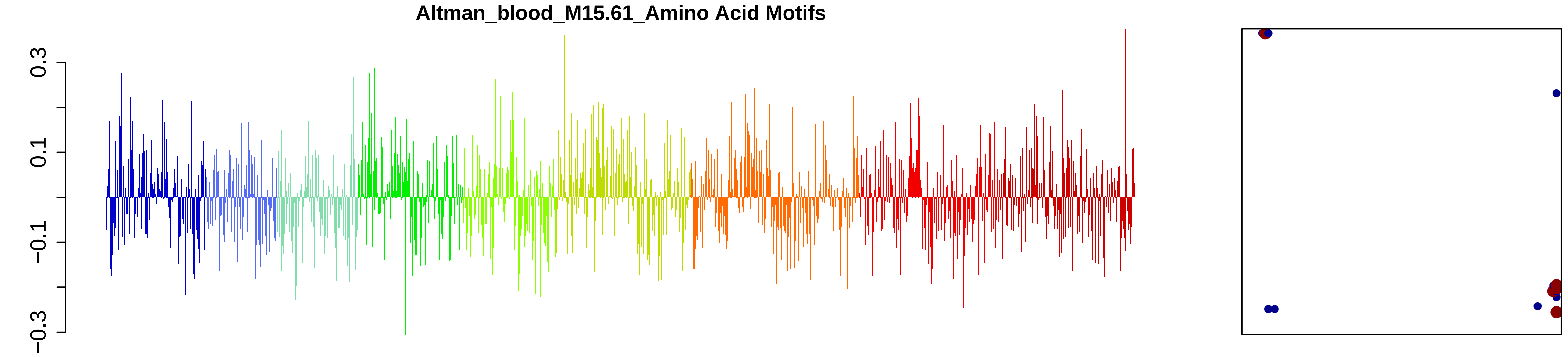
Altman_blood_M15.61_Amino Acid Motifs
0.3
0.1
−0.1
−0.3
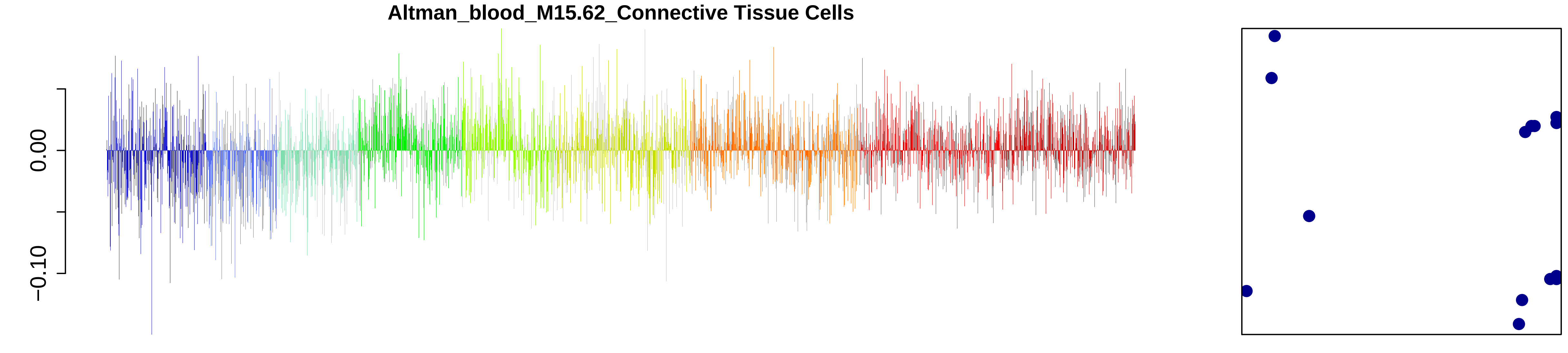
Altman_blood_M15.62_Connective Tissue Cells
0.00
−0.10
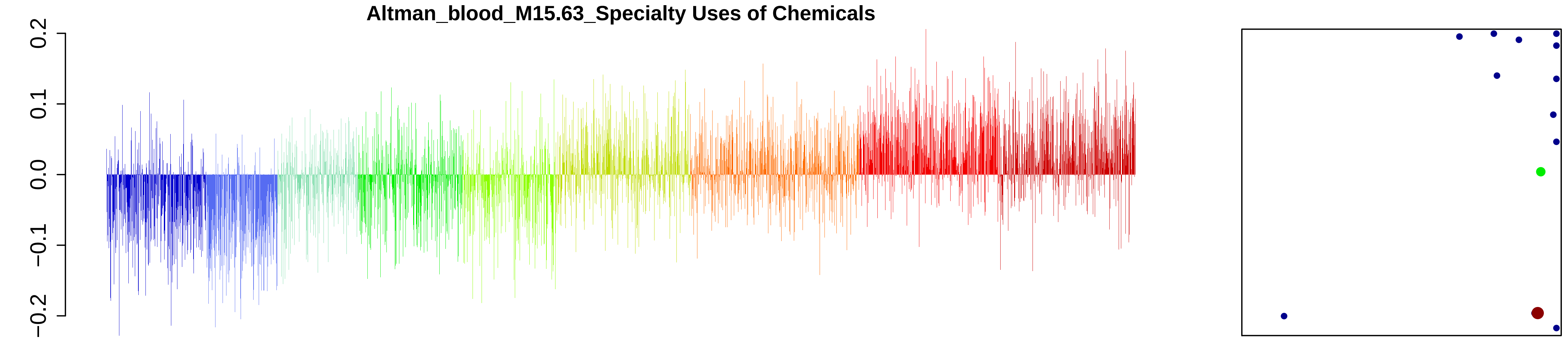
Altman_blood_M15.63_Specialty Uses of Chemicals
0.2
0.1
0.0
−0.1
−0.2
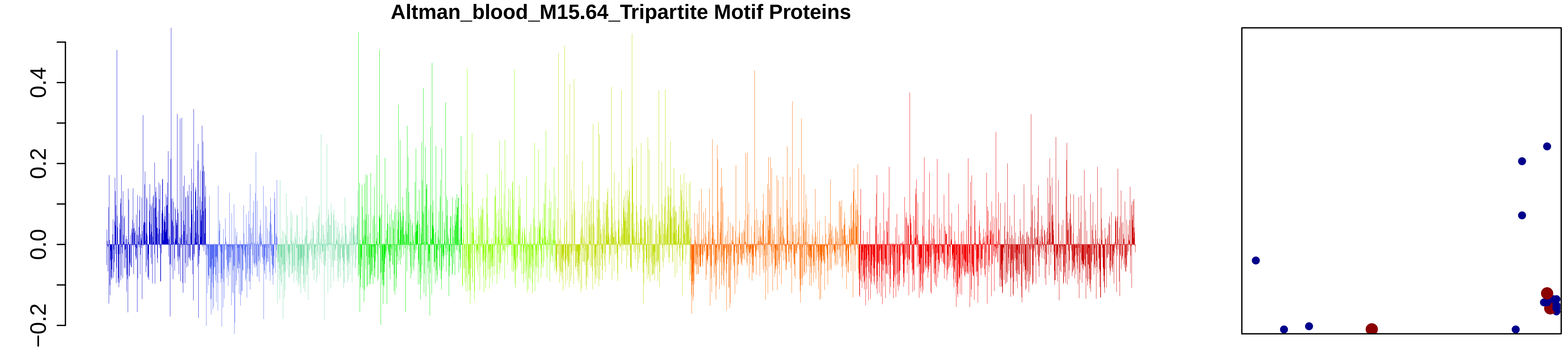
Altman_blood_M15.64_Tripartite Motif Proteins
0.4
0.2
0.0
−0.2

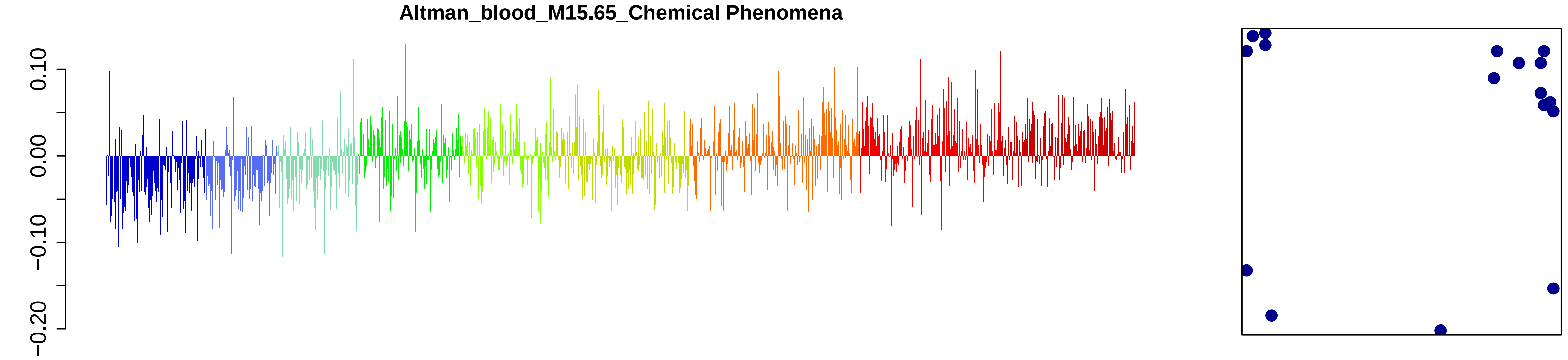
Altman_blood_M15.65_Chemical Phenomena
0.10
0.00
−0.10
−0.20

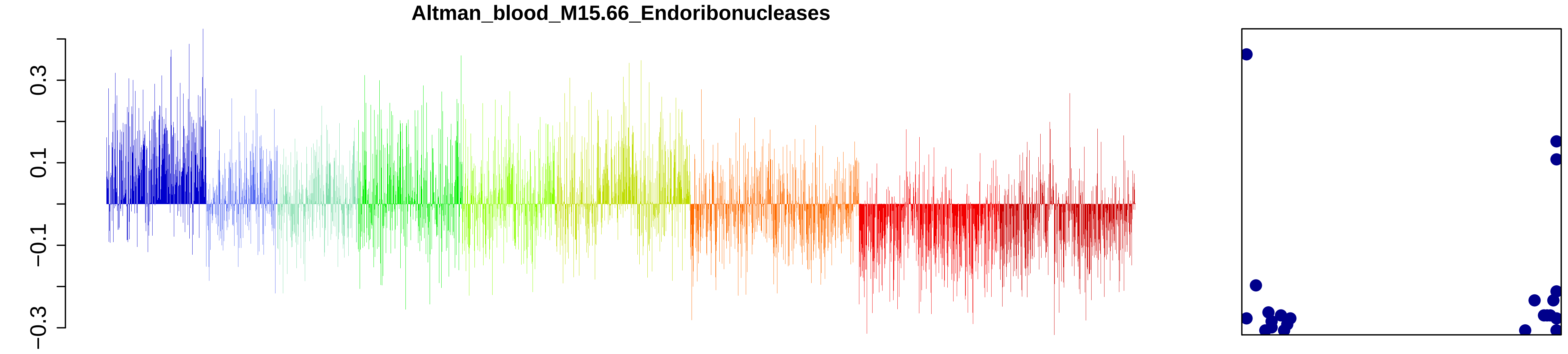
Altman_blood_M15.66_Endoribonucleases
0.3
0.1
−0.1
−0.3

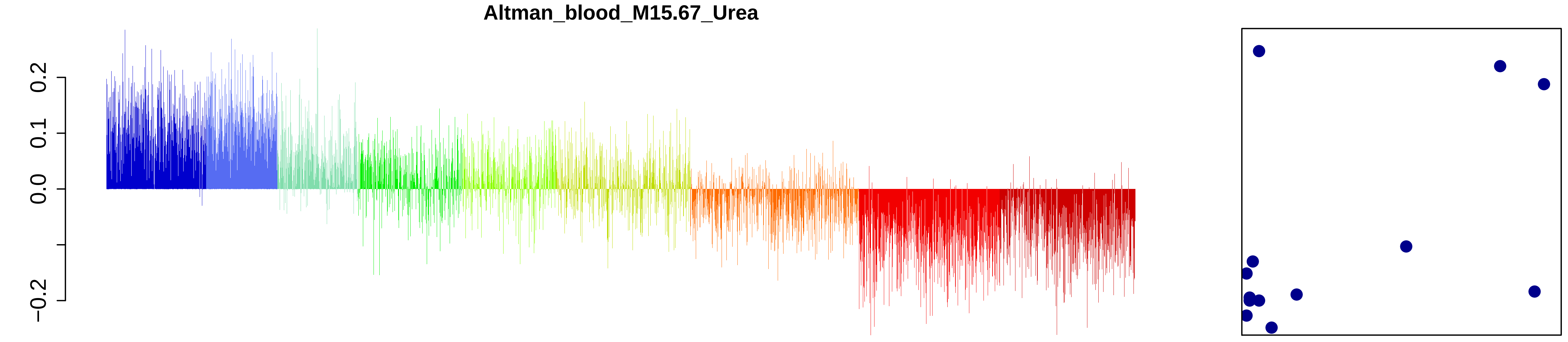
Altman_blood_M15.67_Urea
0.2
0.1
0.0
−0.2

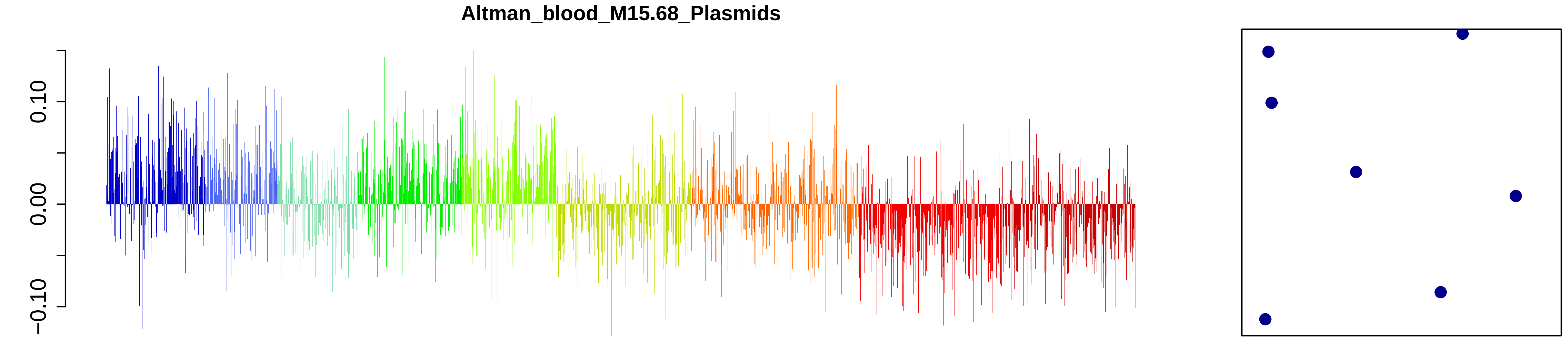
Altman_blood_M15.68_Plasmids
0.10
0.00
−0.10

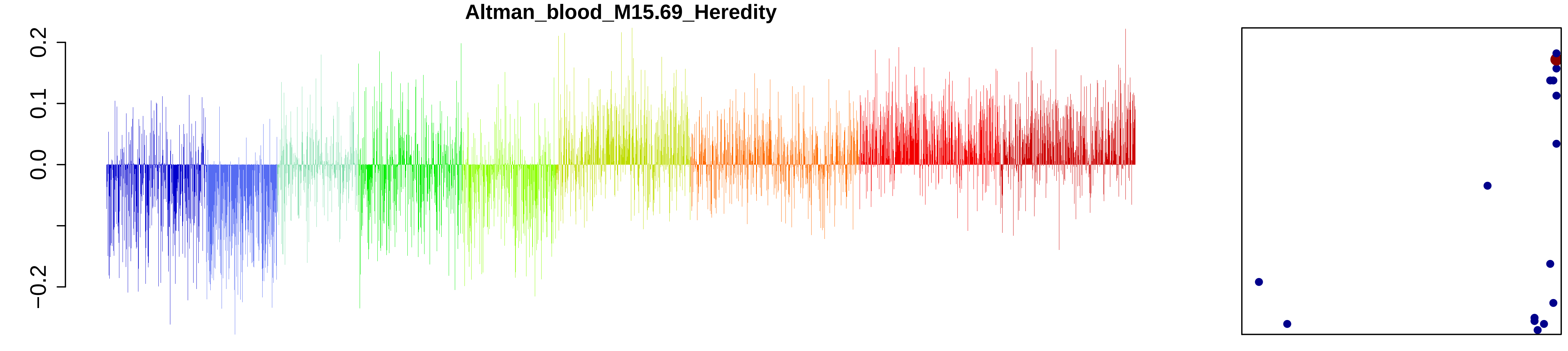
Altman_blood_M15.69_Heredity
0.2
0.1
0.0
−0.2

Altman_blood_M15.70_Efficiency

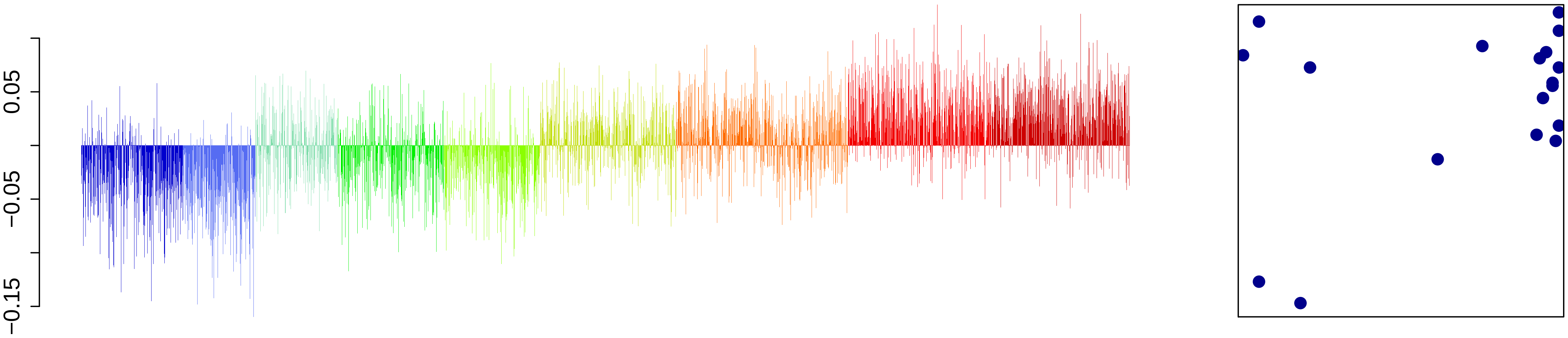

Altman_blood_M15.71_Amino Acids, Peptides, and Proteins

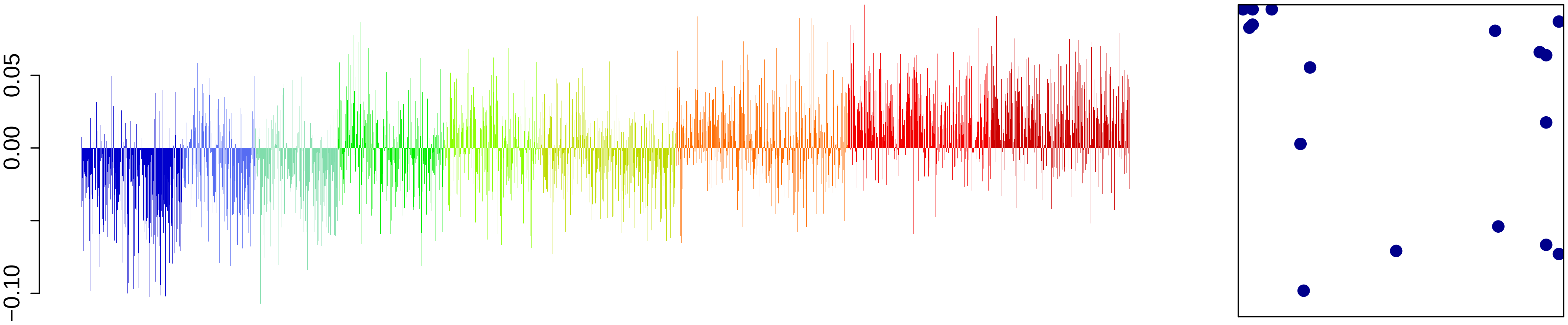

Altman_blood_M15.72_Heredity

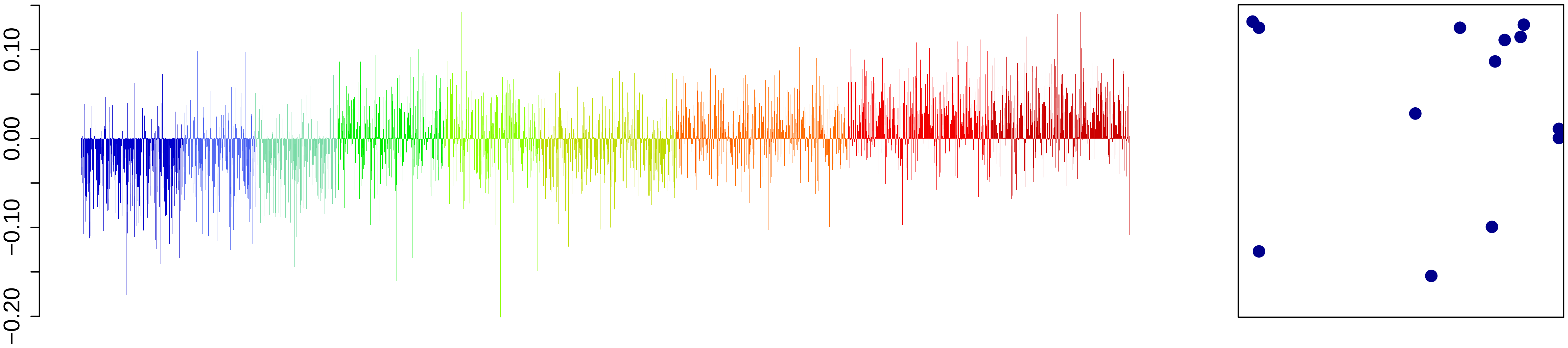

Altman_blood_M15.73_Cells, Cultured

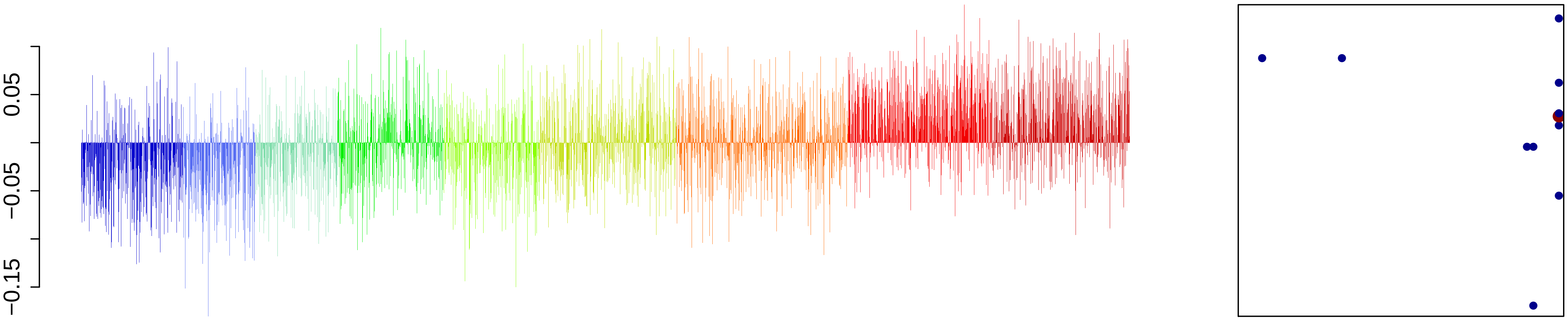

Altman_blood_M15.74_Metabolic Networks and Pathways

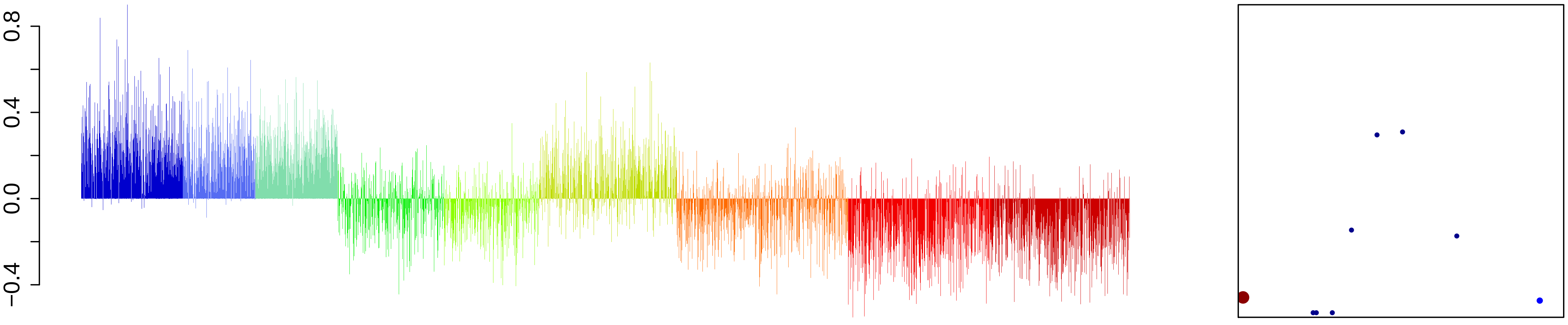

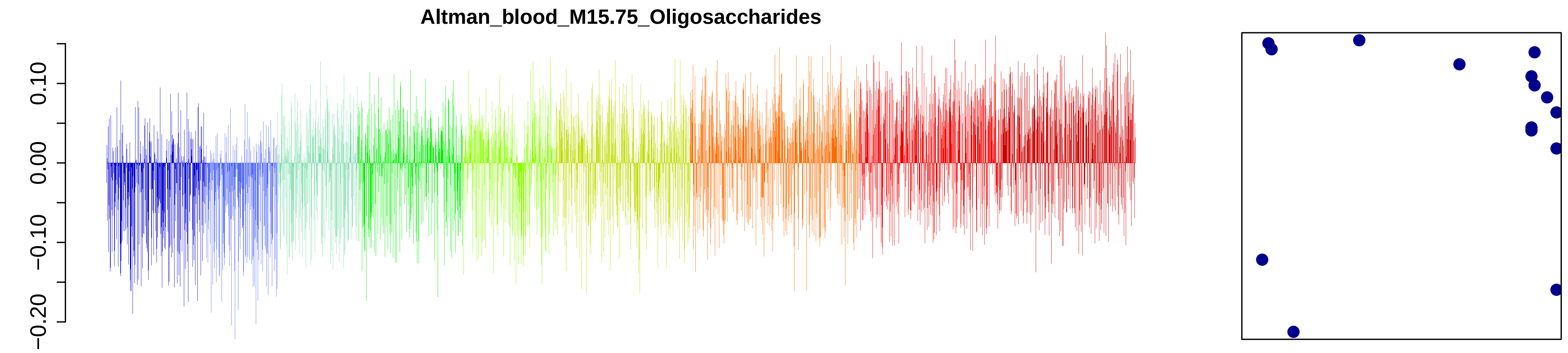
Altman_blood_M15.75_Oligosaccharides
0.10
0.00
−0.10
−0.20

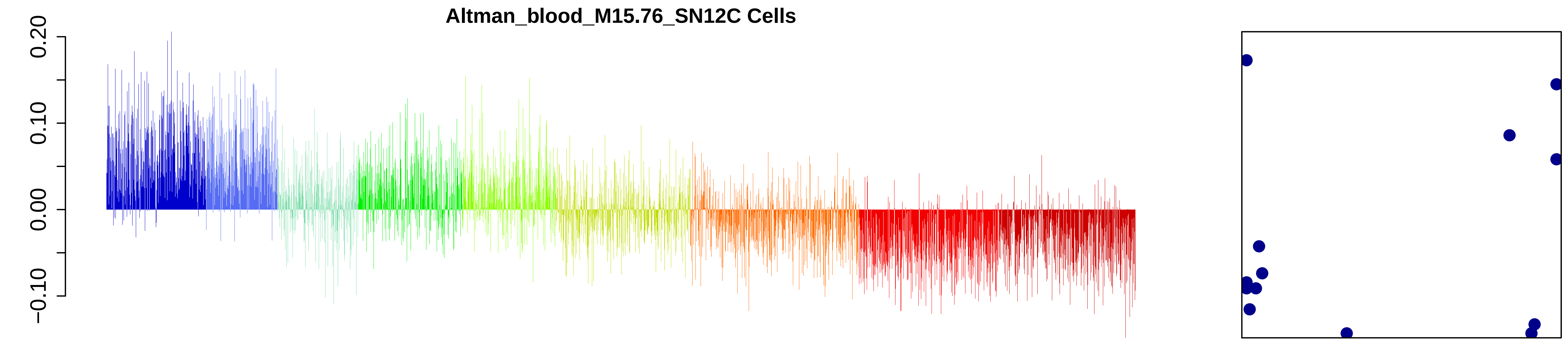
Altman_blood_M15.76_SN12C Cells
0.20
0.10
0.00
−0.10

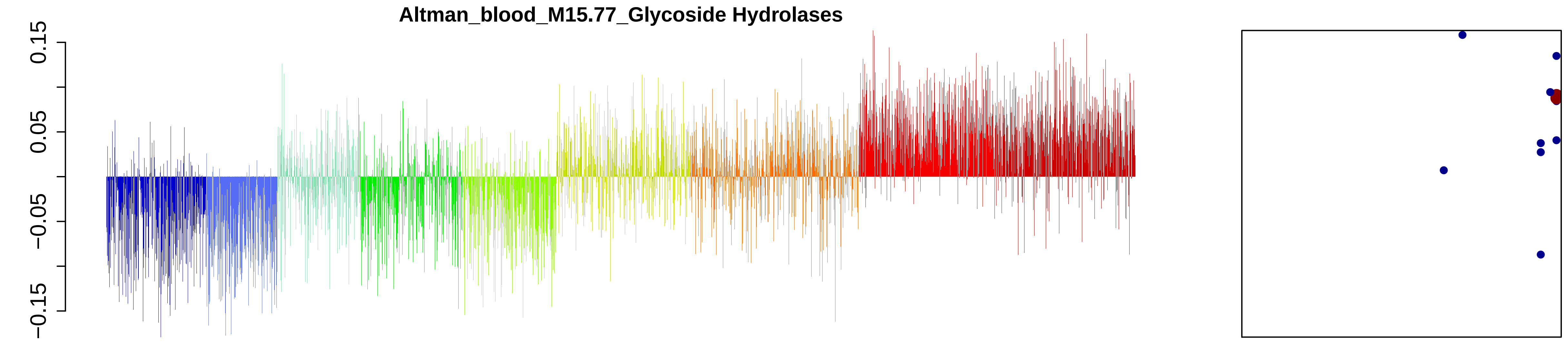
Altman_blood_M15.77_Glycoside Hydrolases
0.15
0.05
−0.05
−0.15

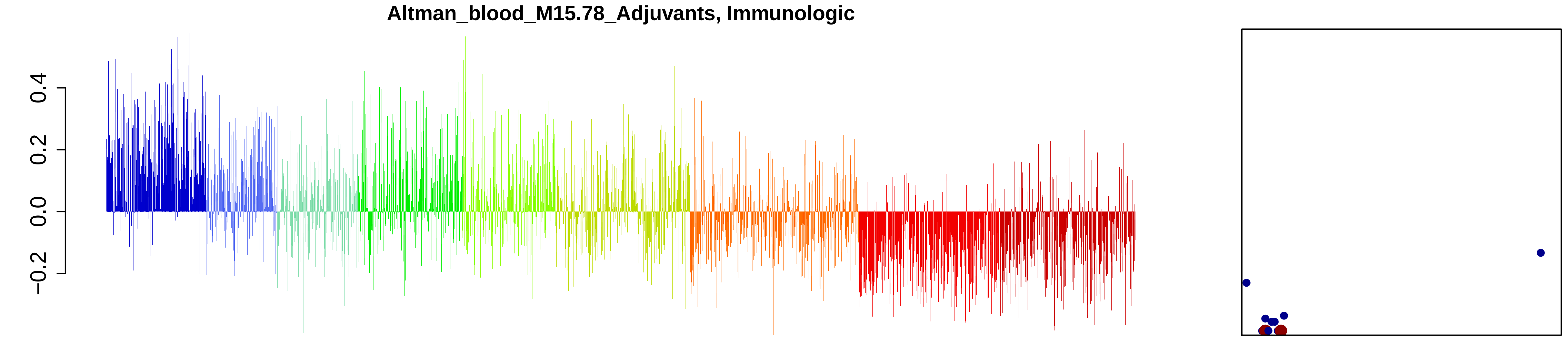
Altman_blood_M15.78_Adjuvants, Immunologic
0.4
0.2
0.0
−0.2

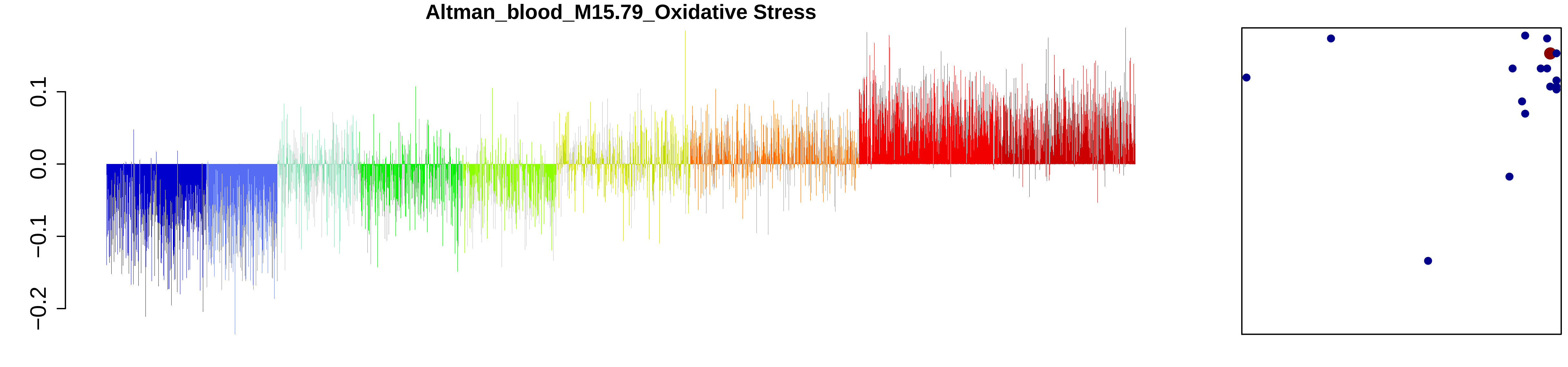
Altman_blood_M15.79_Oxidative Stress
0.1
0.0
−0.1
−0.2

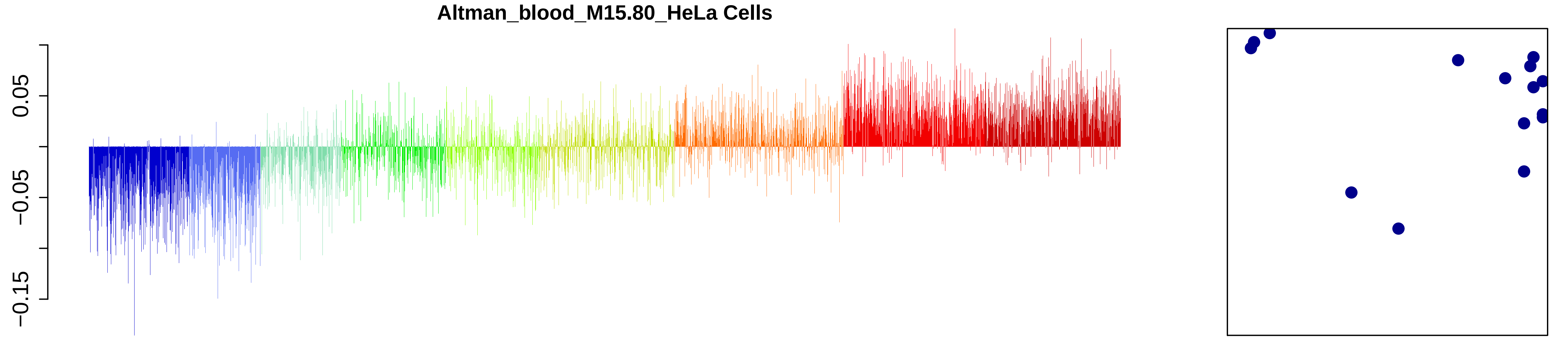
Altman_blood_M15.80_HeLa Cells
0.05
−0.05
−0.15

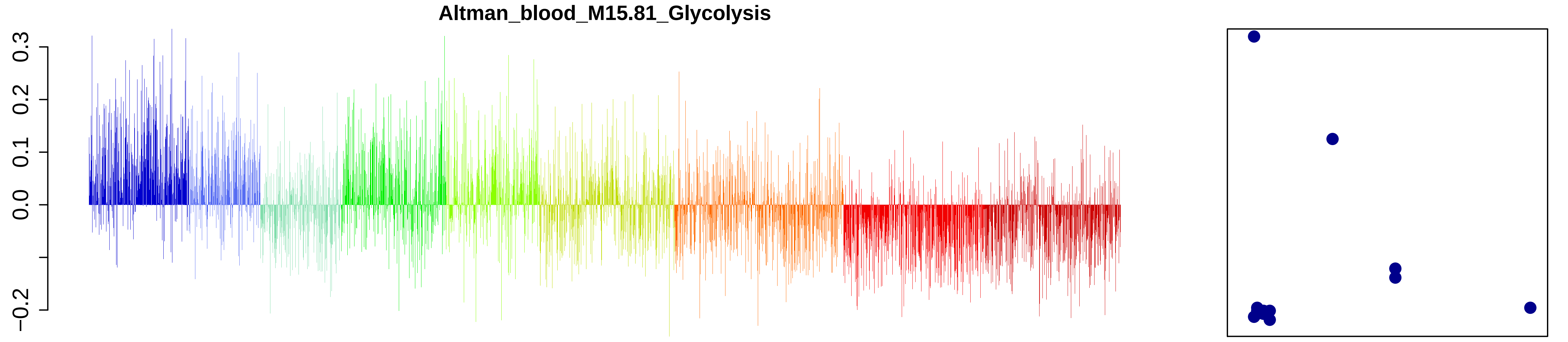
Altman_blood_M15.81_Glycolysis
0.3
0.2
0.1
0.0
−0.2

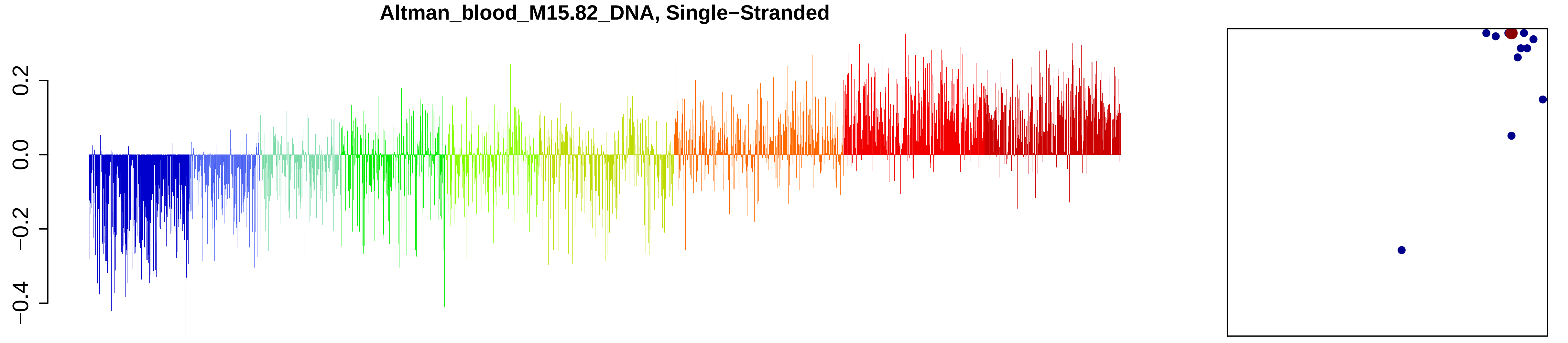
Altman_blood_M15.82_DNA, Single−Stranded
0.2
0.0
−0.2
−0.4

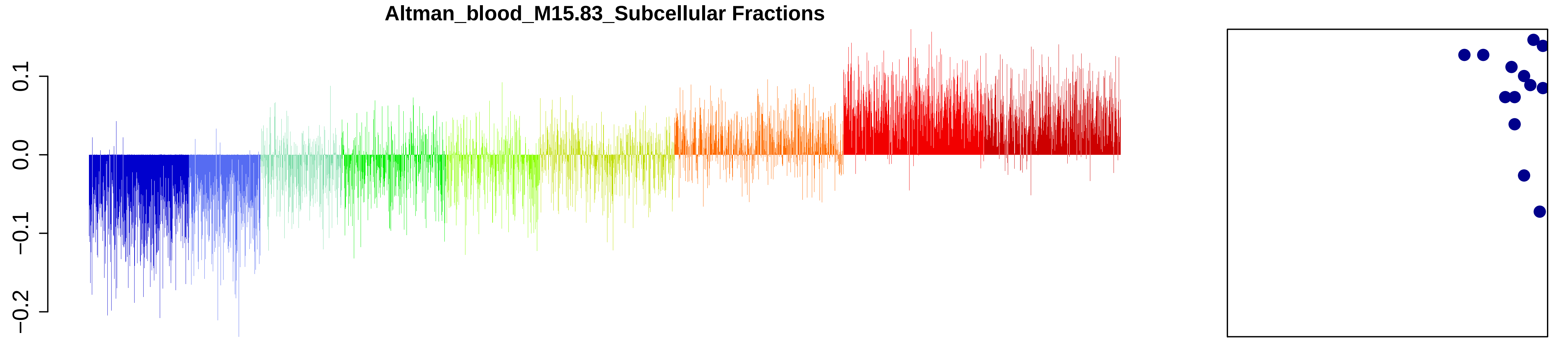
Altman_blood_M15.83_Subcellular Fractions
0.1
0.0
−0.1
−0.2

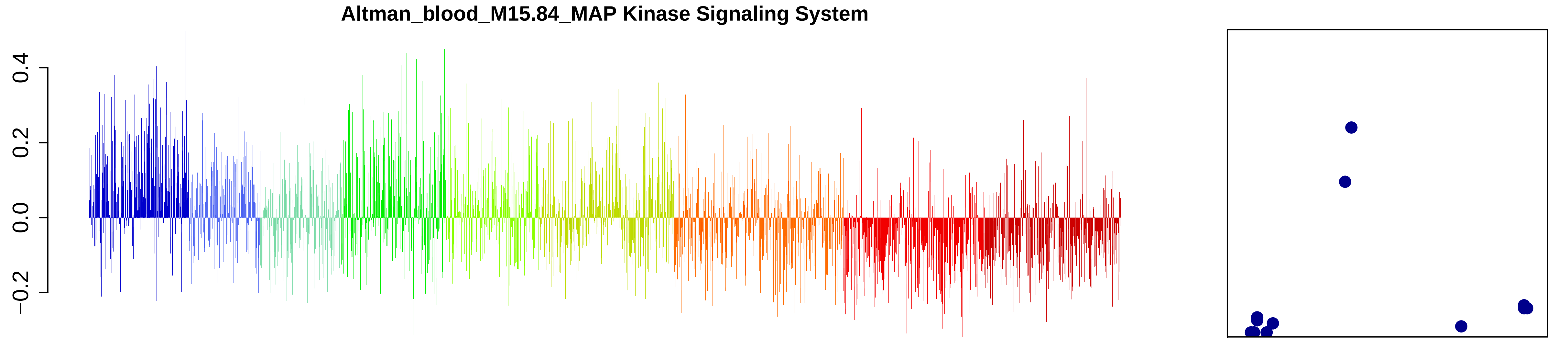
Altman_blood_M15.84_MAP Kinase Signaling System
0.4
0.2
0.0
−0.2

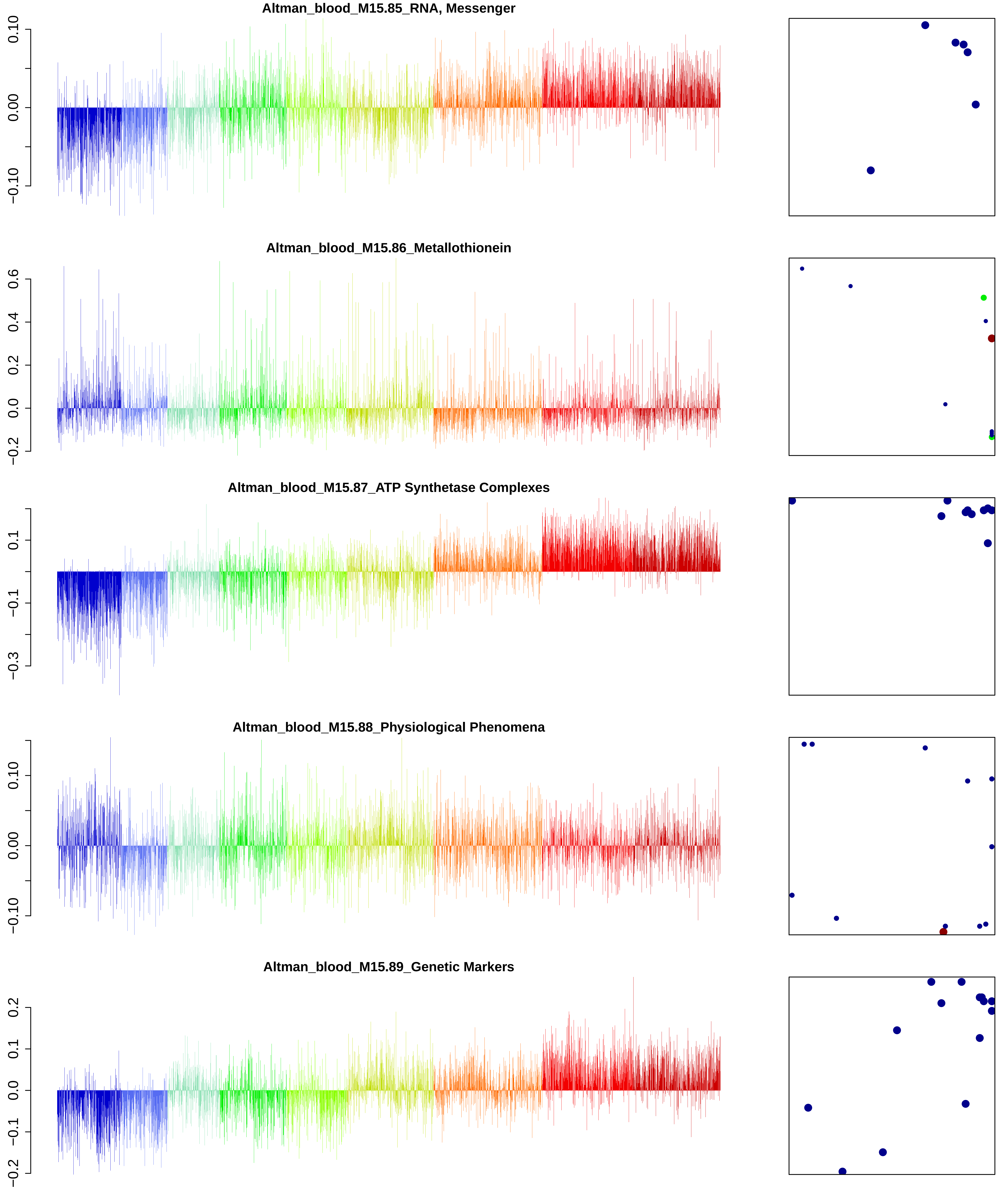
Altman_blood_M15.85_RNA, Messenger
0.10
0.00
−0.10
Altman_blood_M15.86_Metallothionein
0.6
0.4
0.2
0.0
−0.2
Altman_blood_M15.87_ATP Synthetase Complexes
0.1
−0.1
−0.3
Altman_blood_M15.88_Physiological Phenomena
0.10
0.00
−0.10
Altman_blood_M15.89_Genetic Markers
0.2
0.1
0.0
−0.1
−0.2

Altman_blood_M15.90_Hypoxia

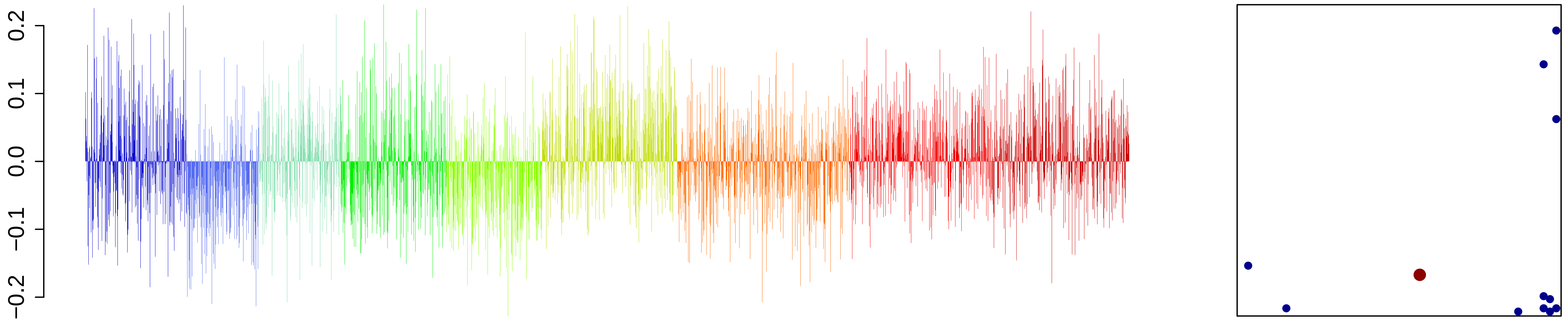

Altman_blood_M15.91_Biotransformation

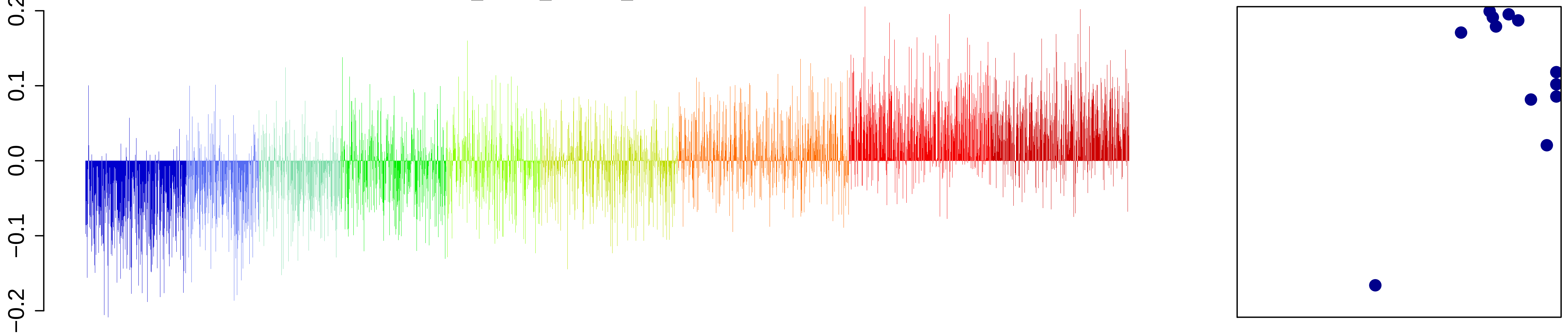

Altman_blood_M15.92_Ribosomal Protein S6 Kinases

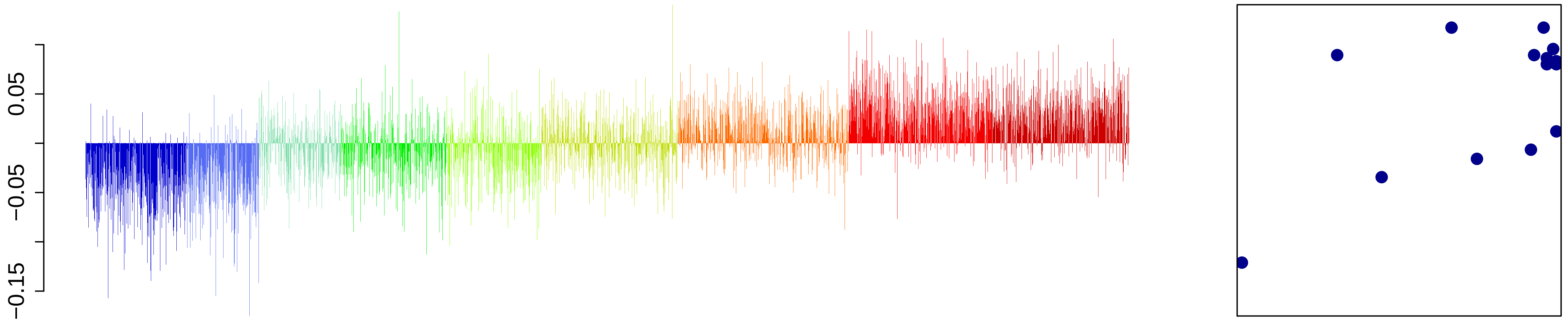

Altman_blood_M15.93_Nucleotides

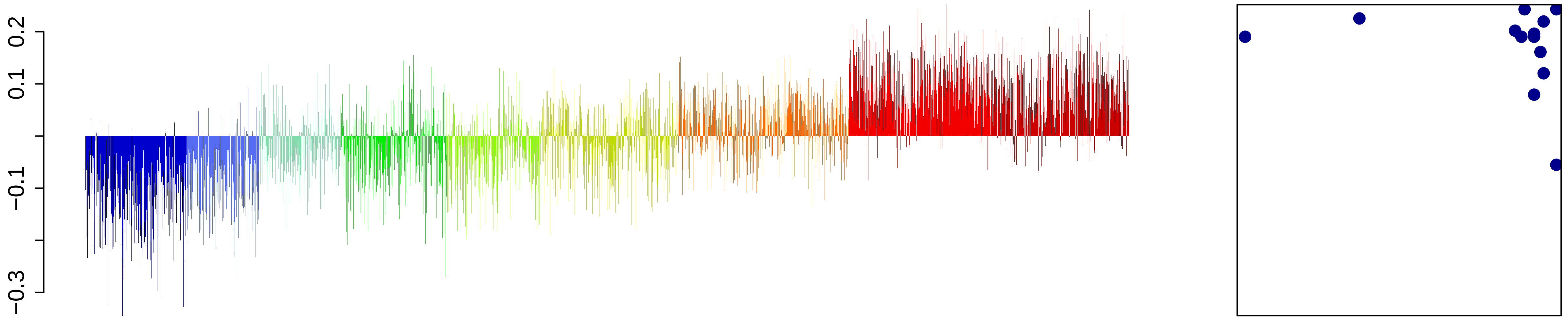

Altman_blood_M15.94_Recombination, Genetic

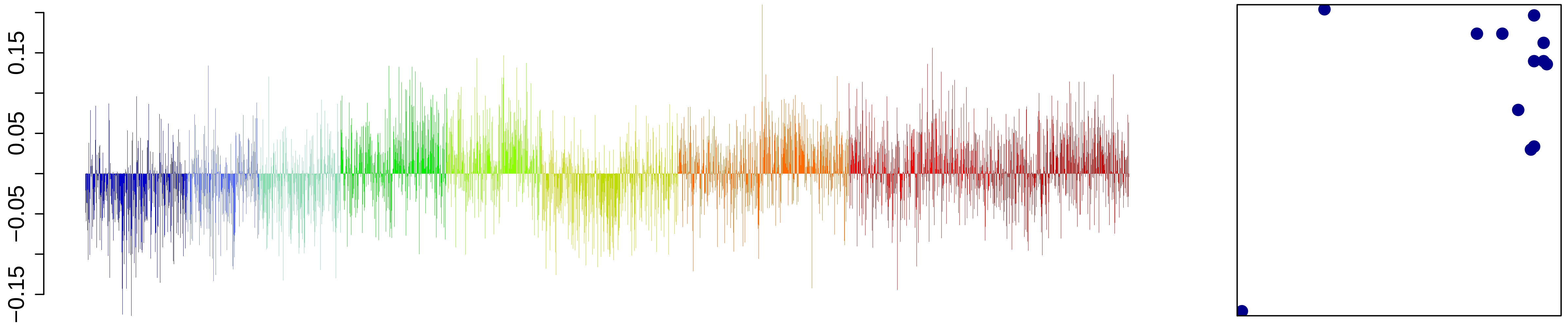

Altman_blood_M15.95_Prostaglandin E2

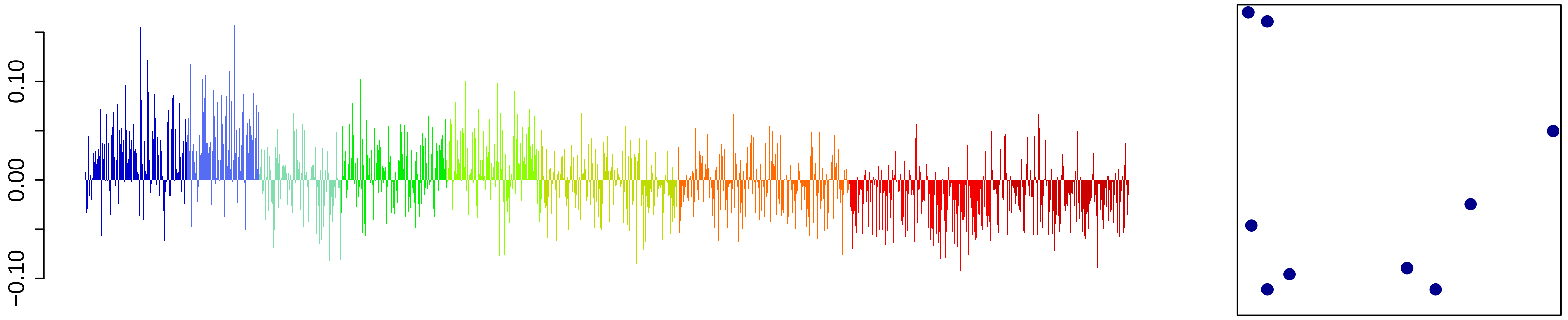

Altman_blood_M15.96_Biotransformation

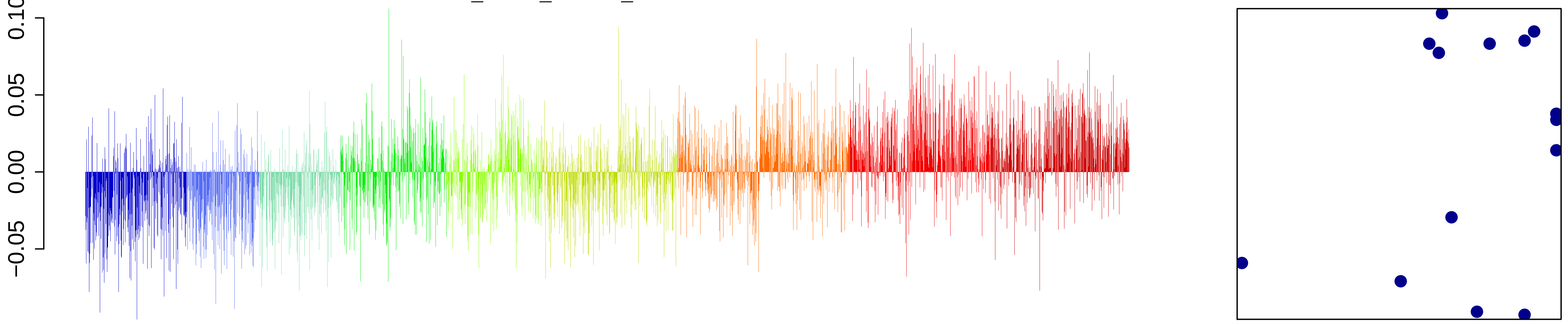

Altman_blood_M15.97_Biotransformation

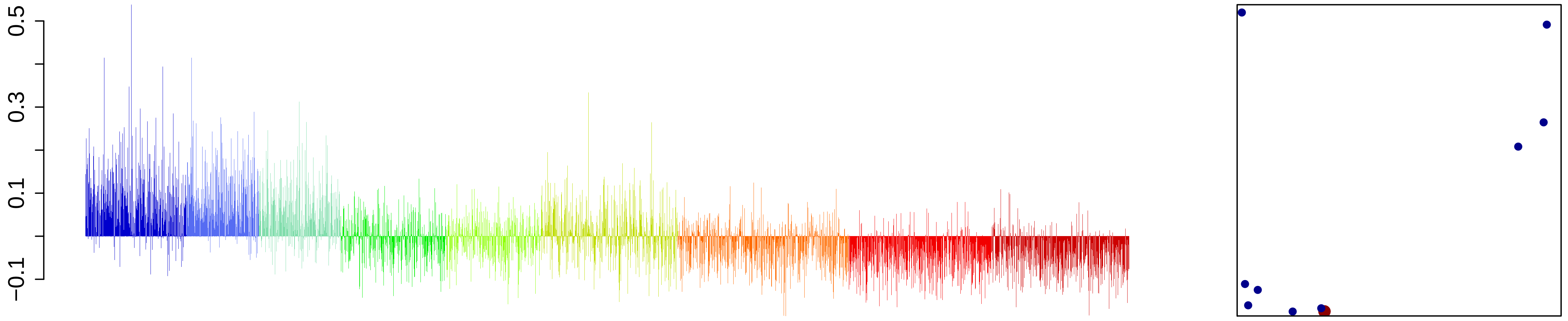

Altman_blood_M15.98_Introns

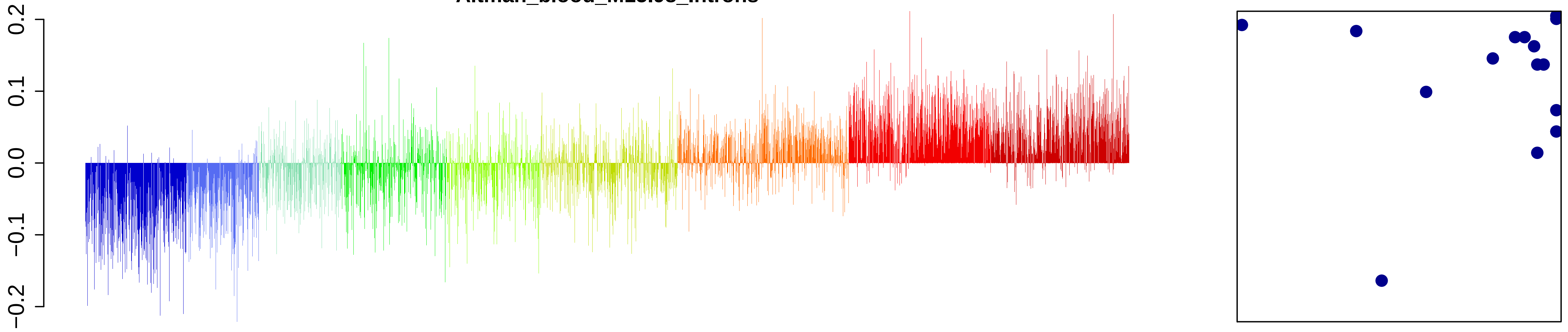

Altman_blood_M15.99_Protein Structure, Secondary

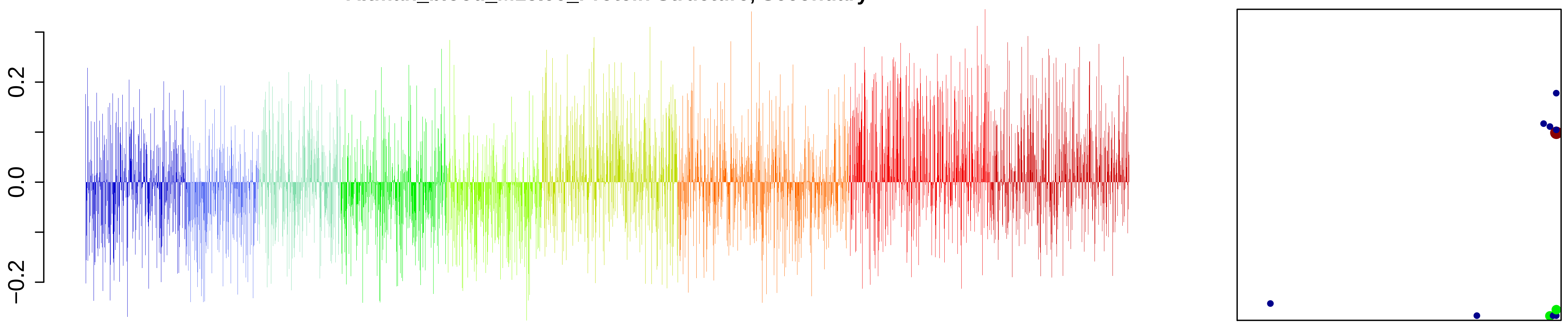

Altman_blood_M16.10_Nucleic Acid Hybridization

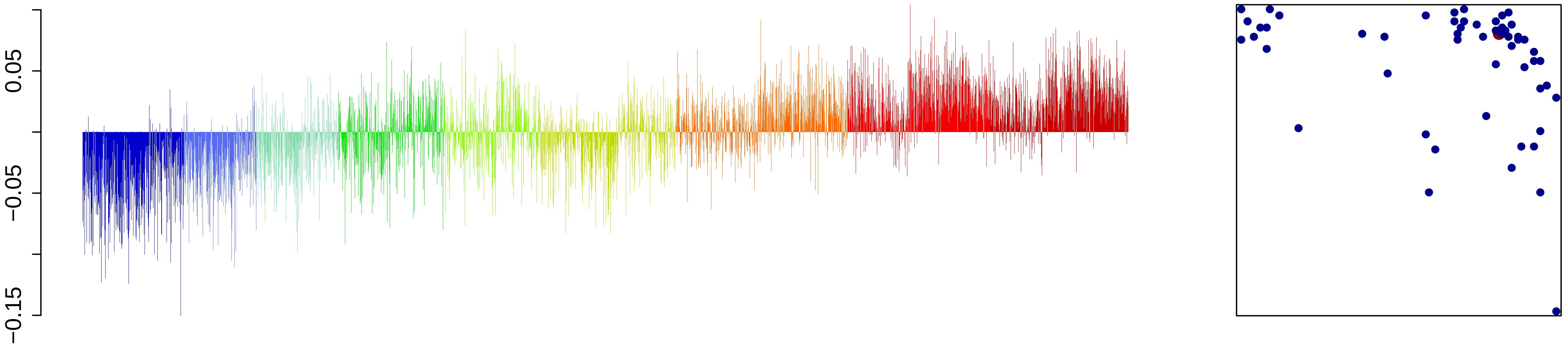

Altman_blood_M16.11_Hot Temperature

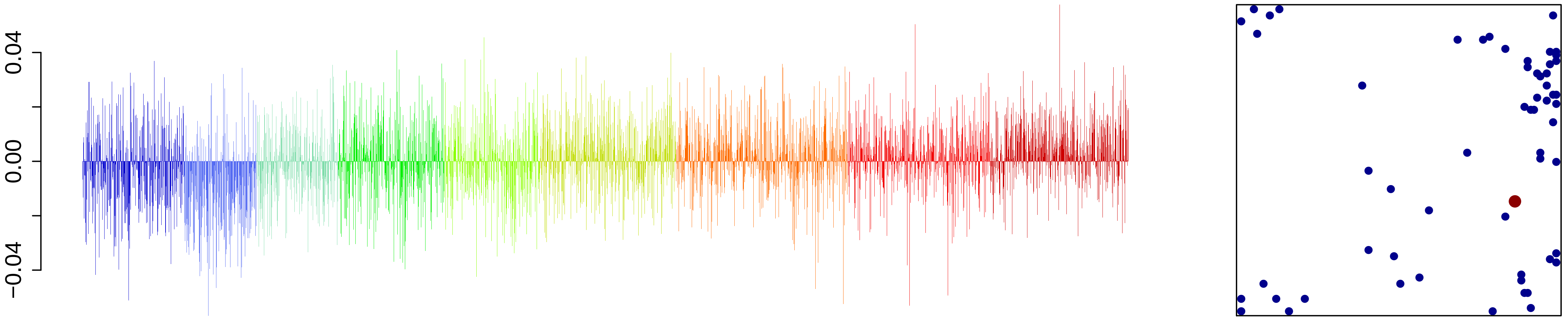

Altman_blood_M16.12_Genome

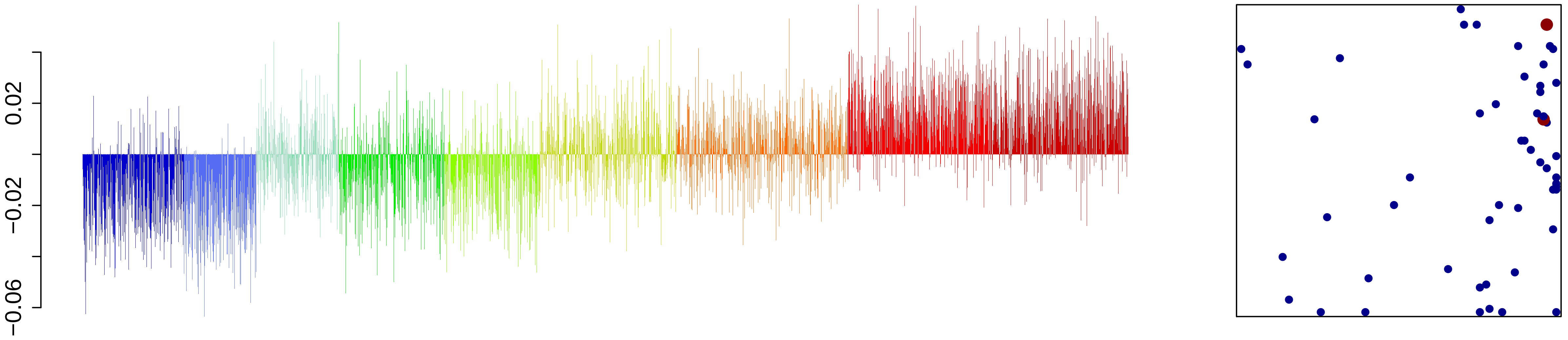

Altman_blood_M16.13_Colonic Diseases

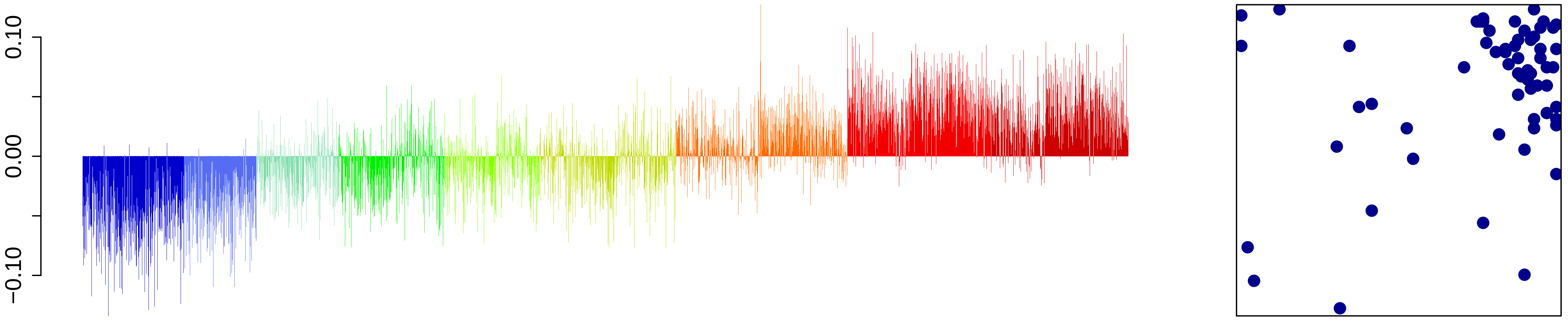

Altman_blood_M16.14_Transgenes

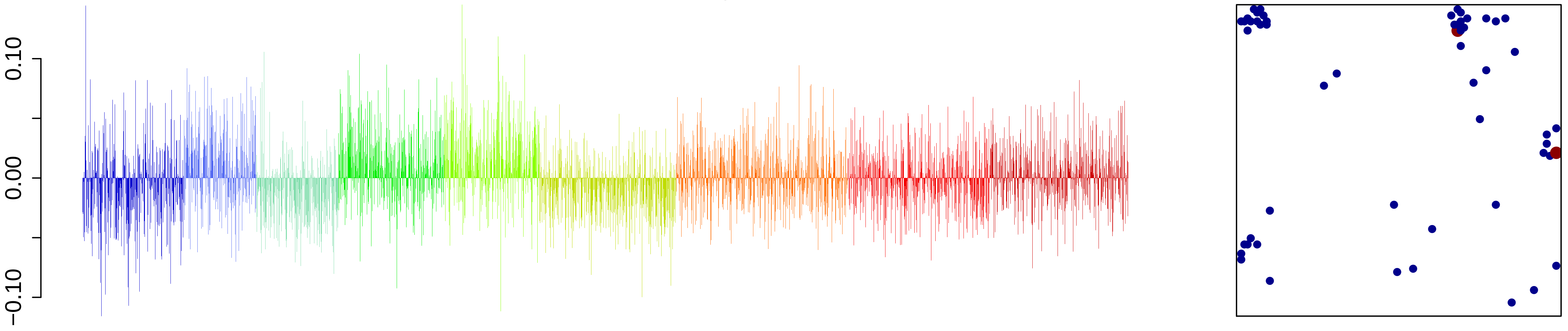

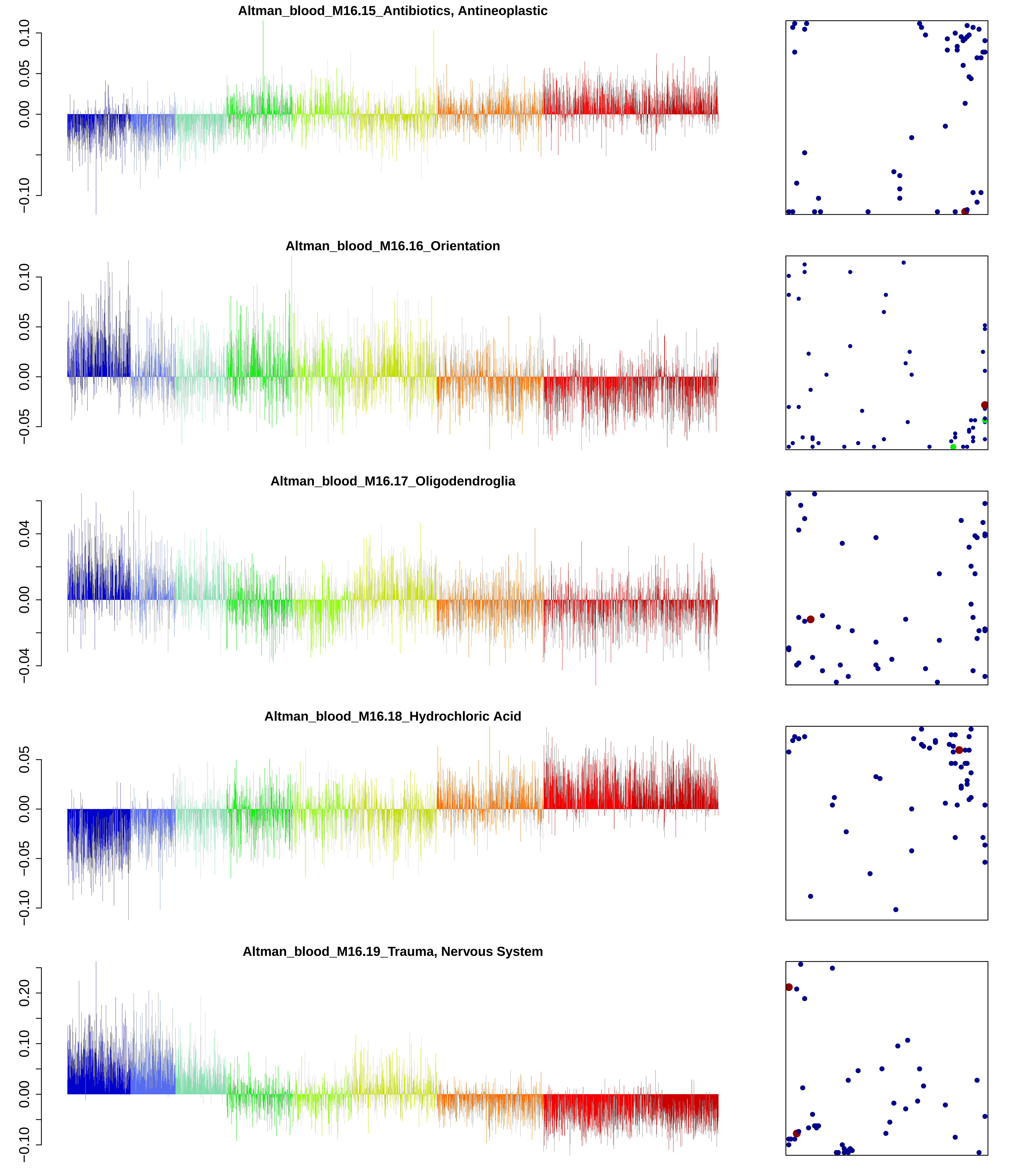
Altman_blood_M16.15_Antibiotics, Antineoplastic
−0.10
0.00
0.05
0.10
Altman_blood_M16.16_Orientation
−0.05
0.00
0.05
0.10
Altman_blood_M16.17_Oligodendroglia
−0.04
0.00
0.04
Altman_blood_M16.18_Hydrochloric Acid
−0.10
−0.05
0.00
0.05
Altman_blood_M16.19_Trauma, Nervous System
−0.10
0.00
0.10
0.20

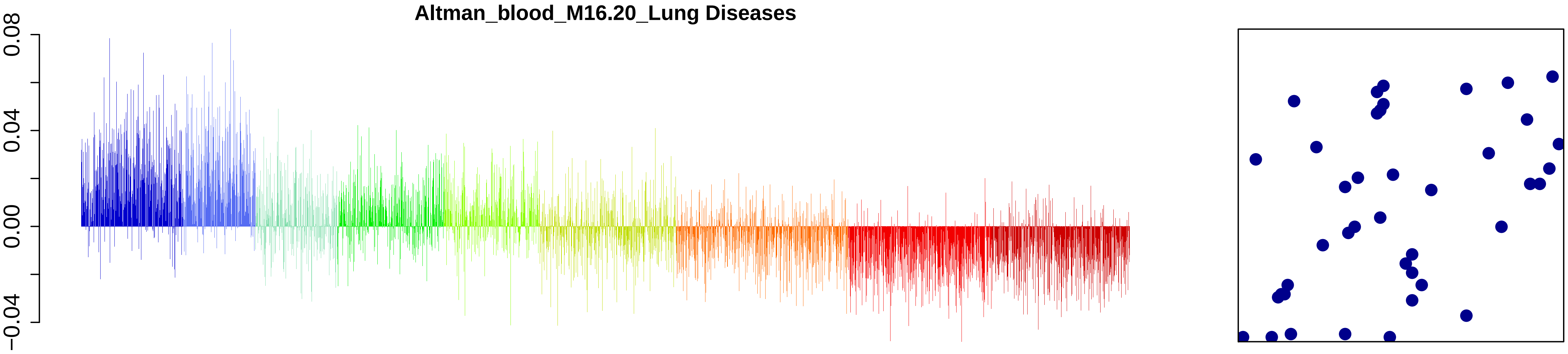
Altman_blood_M16.20_Lung Diseases
0.08
0.04
0.00
−0.04

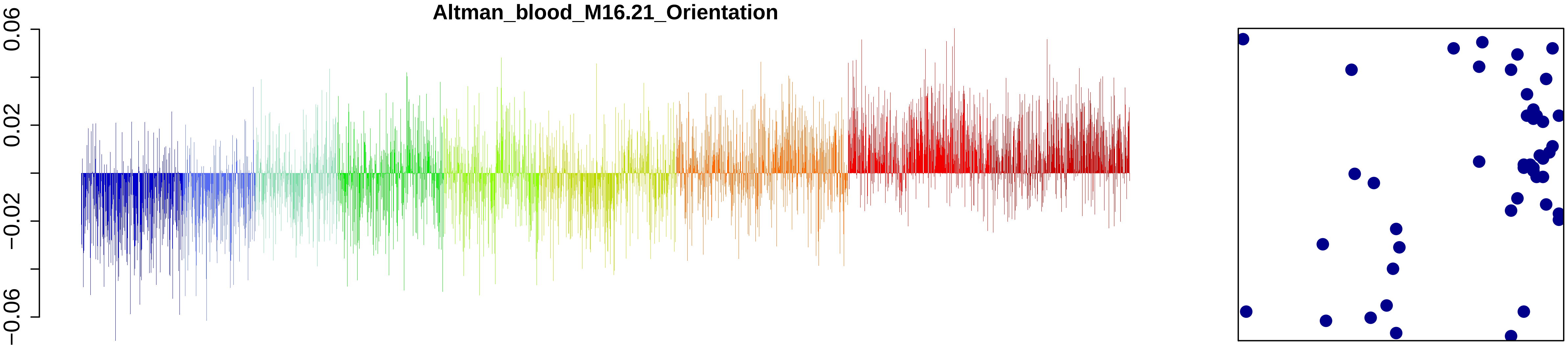
Altman_blood_M16.21_Orientation
0.06
0.02
−0.02
−0.06

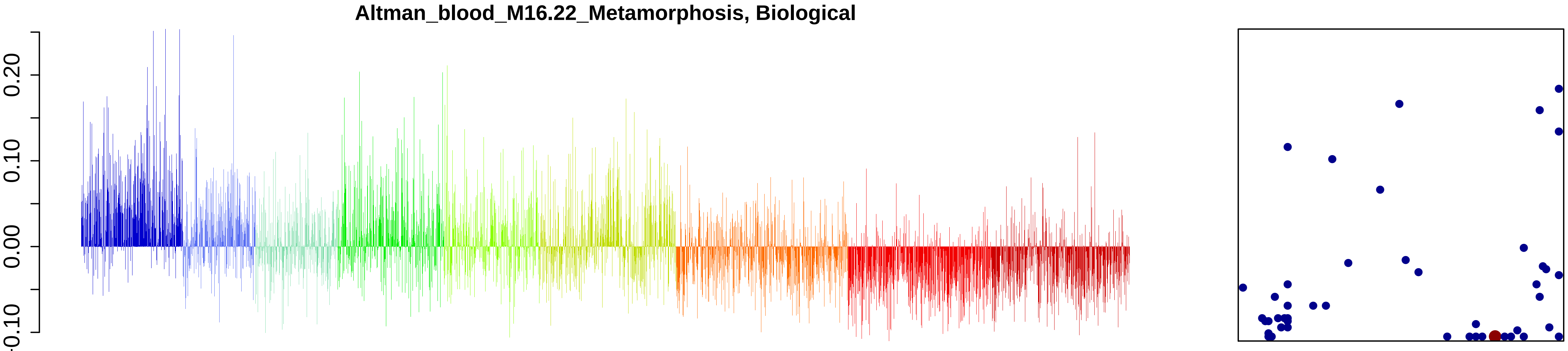
Altman_blood_M16.22_Metamorphosis, Biological
0.20
0.10
0.00
−0.10

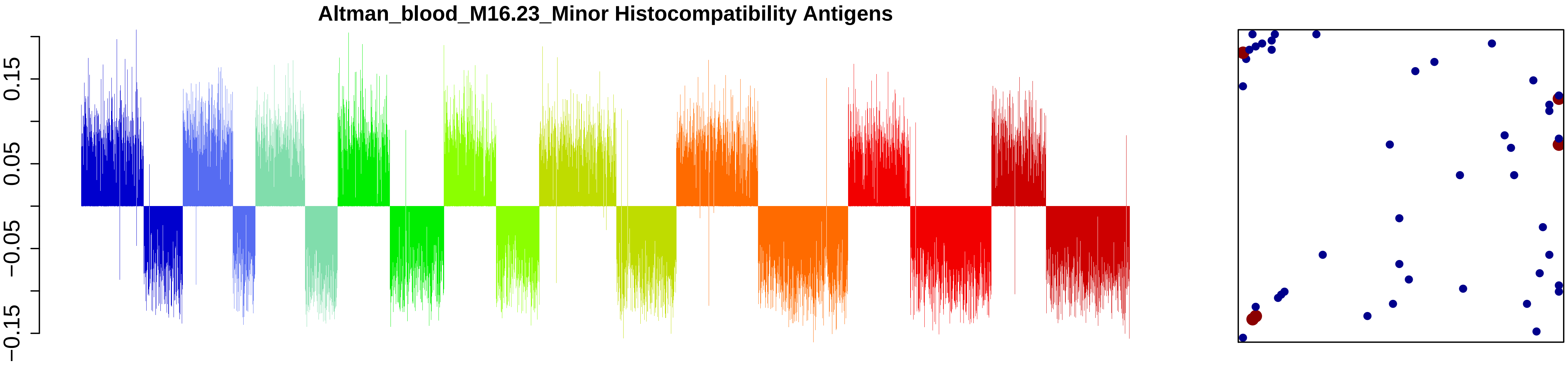
Altman_blood_M16.23_Minor Histocompatibility Antigens
0.15
0.05
−0.05
−0.15

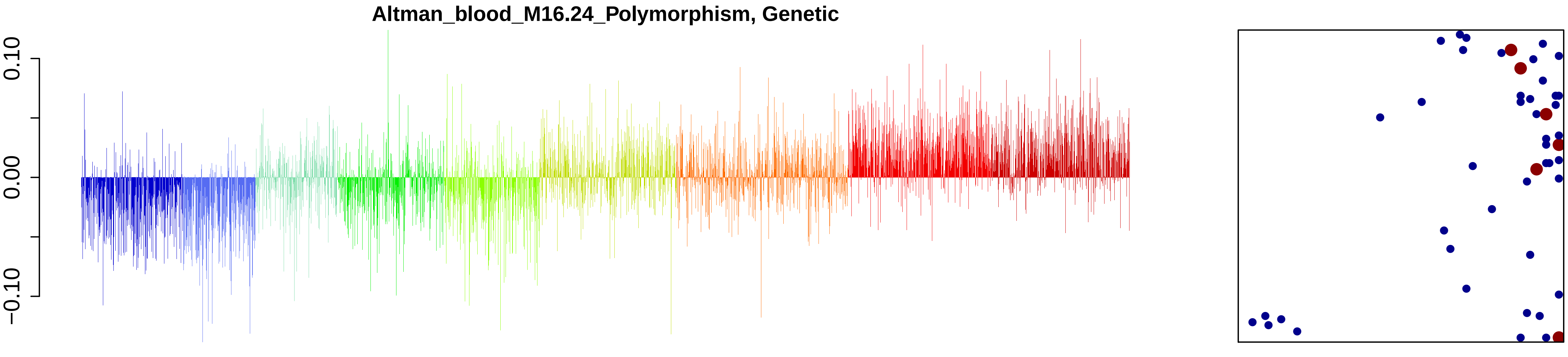
Altman_blood_M16.24_Polymorphism, Genetic
0.10
0.00
−0.10

Altman_blood_M16.25_Alternative Splicing

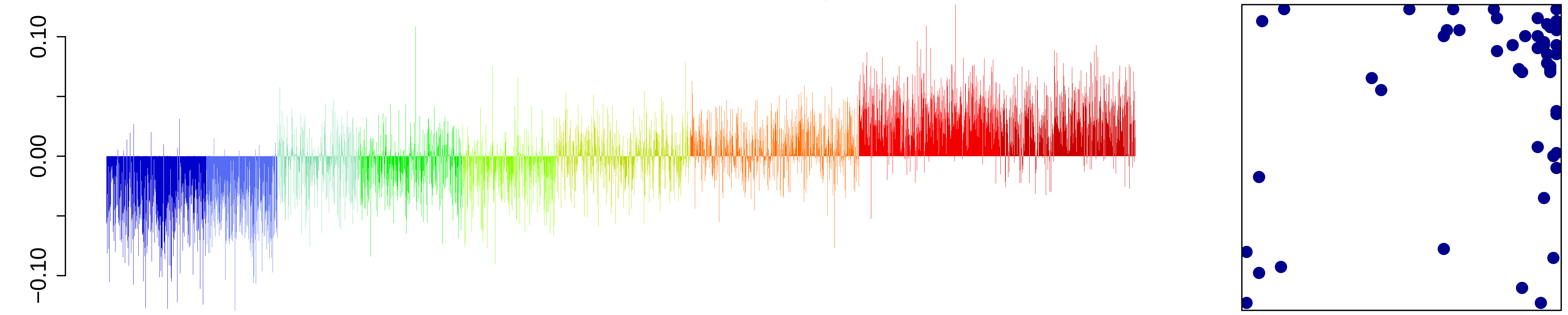

Altman_blood_M16.26_Protein Interaction Maps

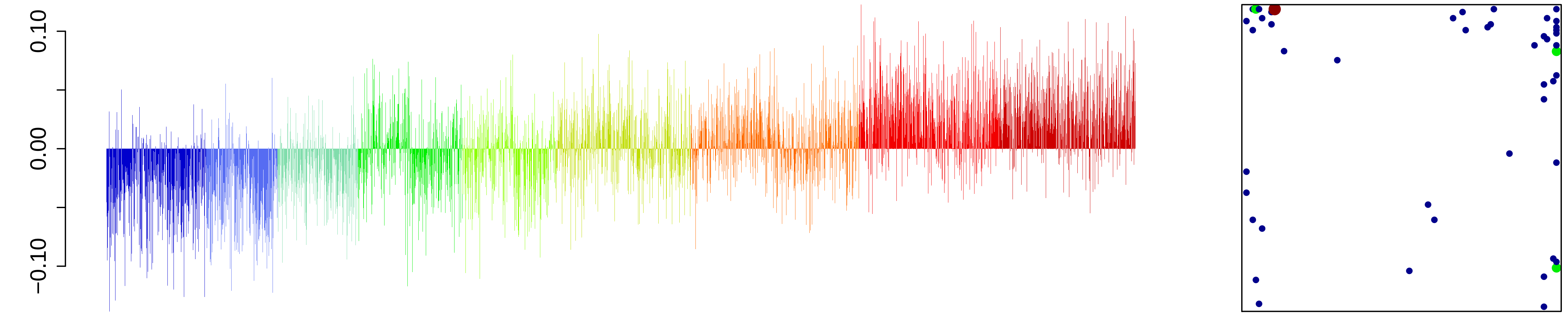

Altman_blood_M16.27_Epithelial−Mesenchymal Transition

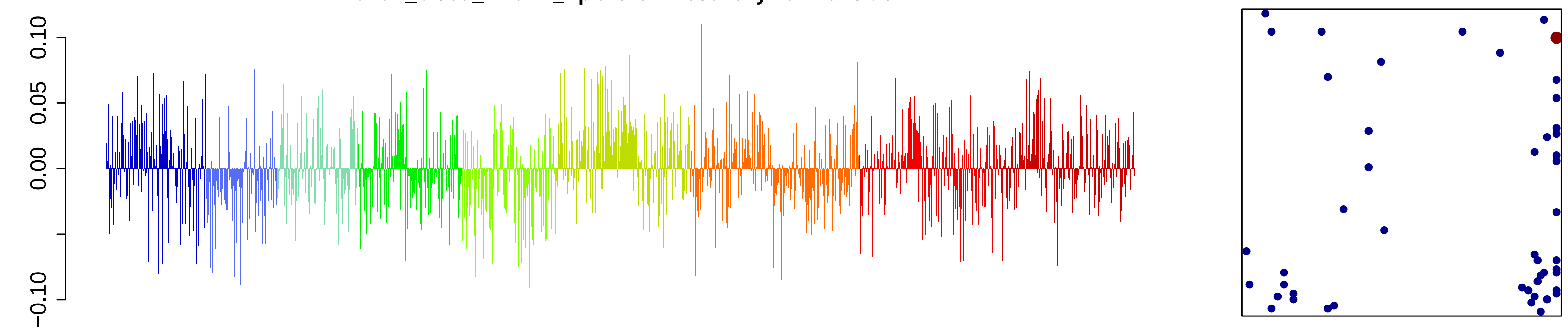

Altman_blood_M16.28_Attention

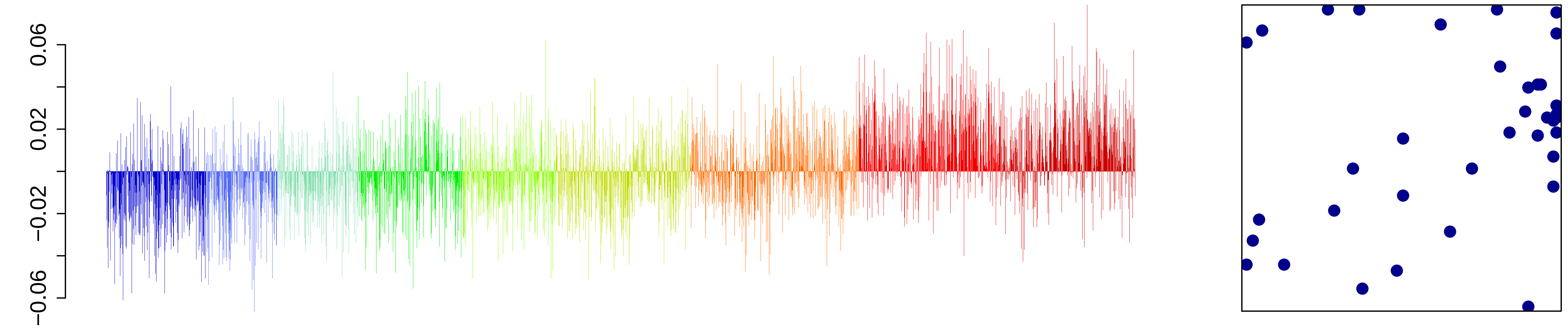

Altman_blood_M16.29_Biotin

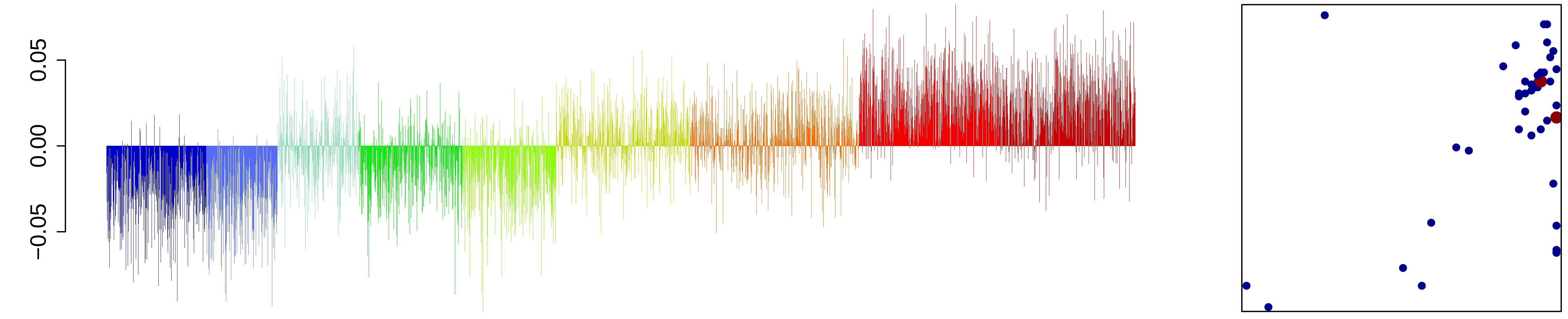

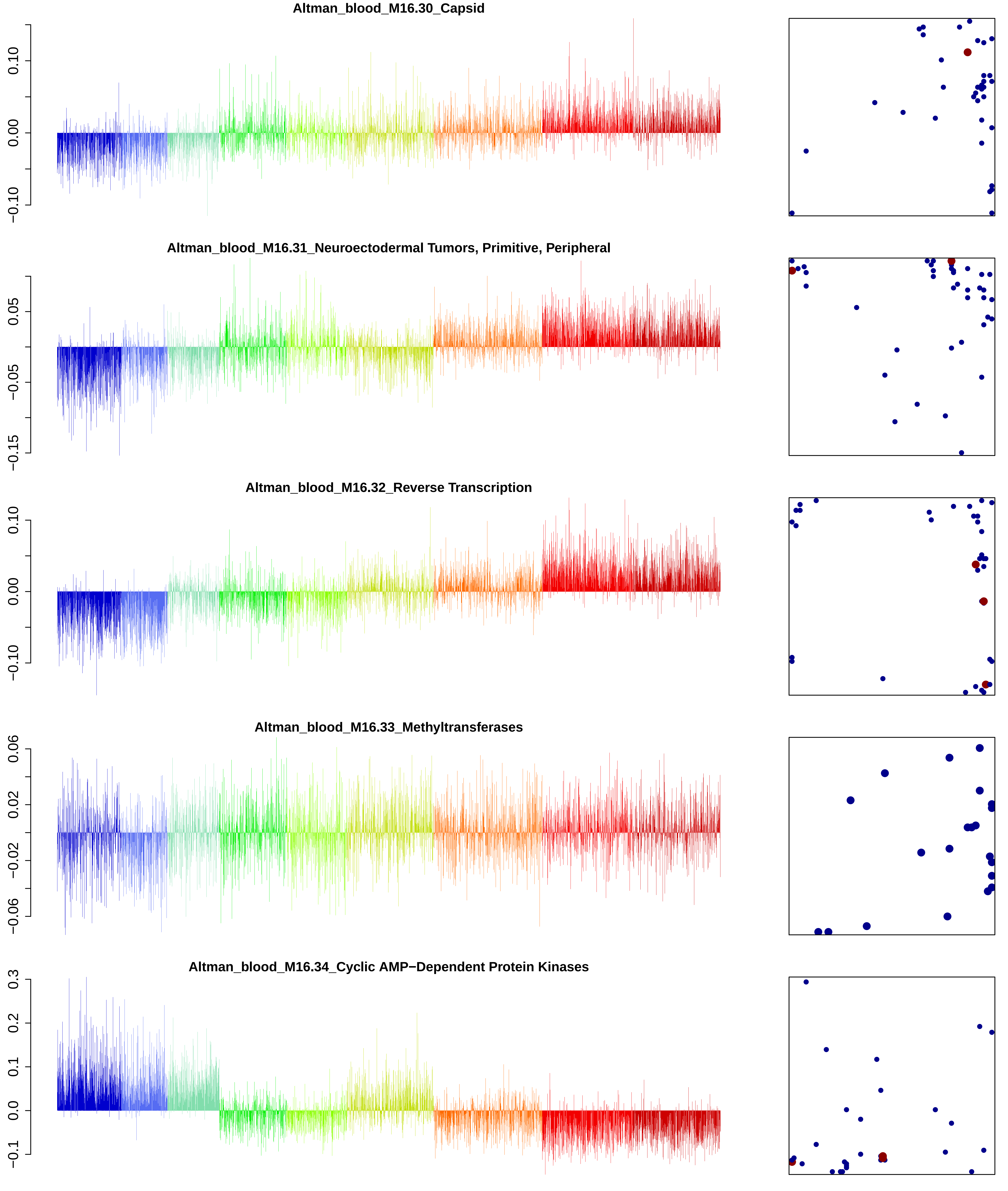
Altman_blood_M16.30_Capsid
0.10
0.00
−0.10
Altman_blood_M16.31_Neuroectodermal Tumors, Primitive, Peripheral
0.05
−0.05
−0.15
Altman_blood_M16.32_Reverse Transcription
0.10
0.00
−0.10
Altman_blood_M16.33_Methyltransferases
0.06
0.02
−0.02
−0.06
Altman_blood_M16.34_Cyclic AMP−Dependent Protein Kinases
0.3
0.2
0.1
0.0
−0.1

Altman_blood_M16.35_Casein Kinases

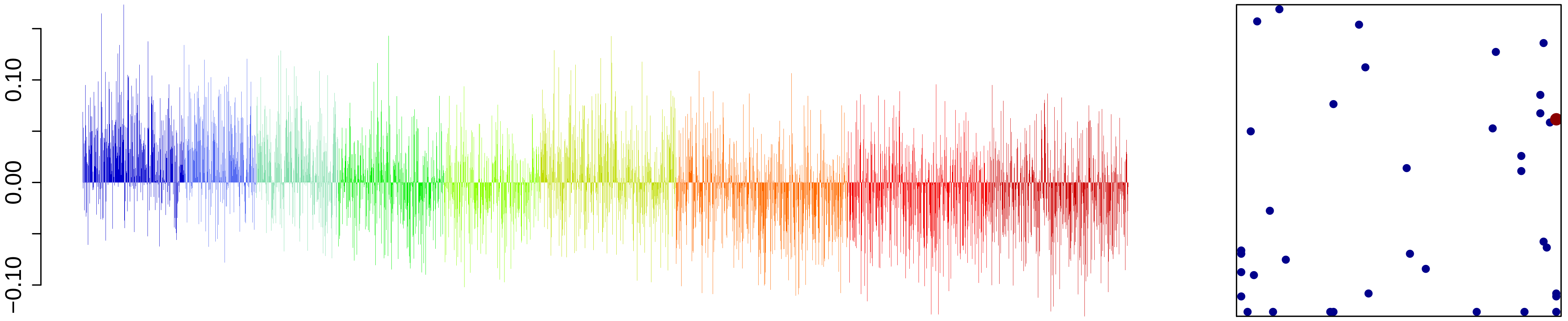

Altman_blood_M16.36_Aldehyde Oxidoreductases

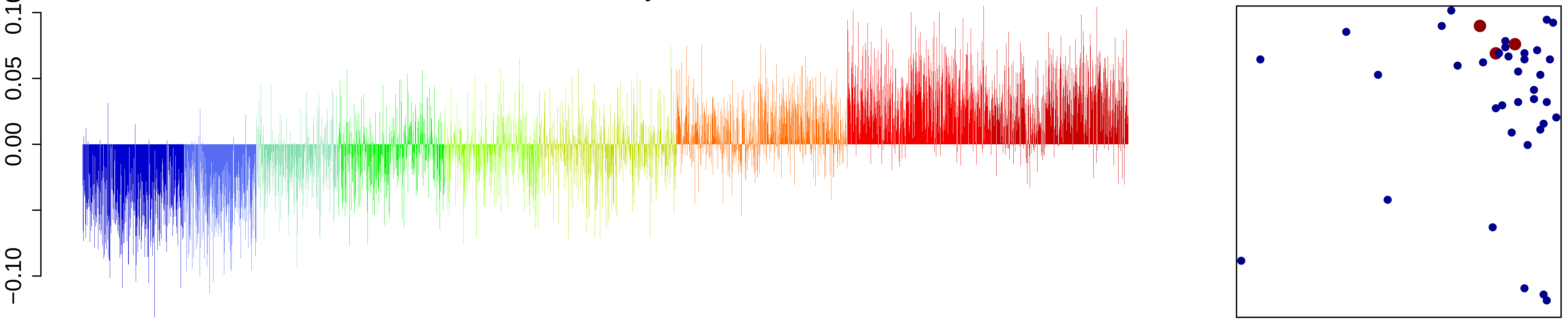

Altman_blood_M16.37_Chymotrypsin

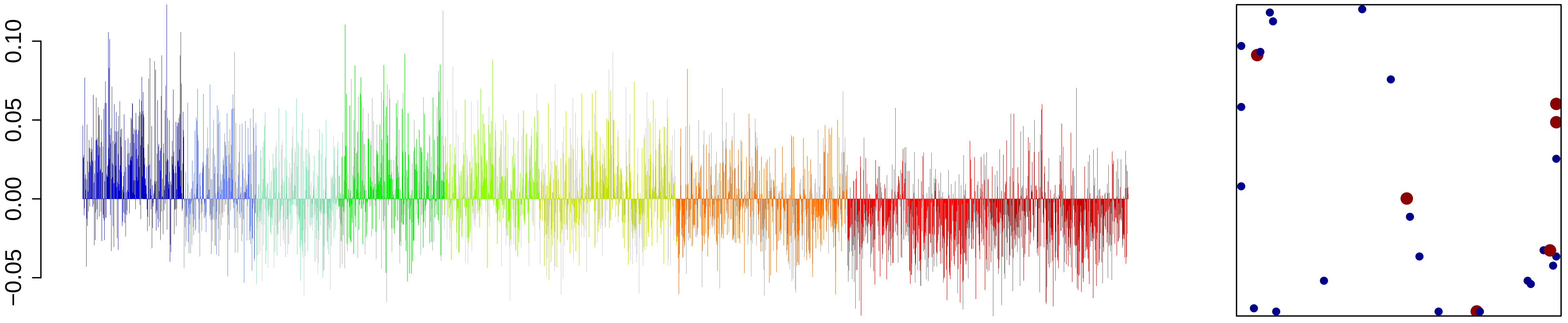

Altman_blood_M16.38_Tissues

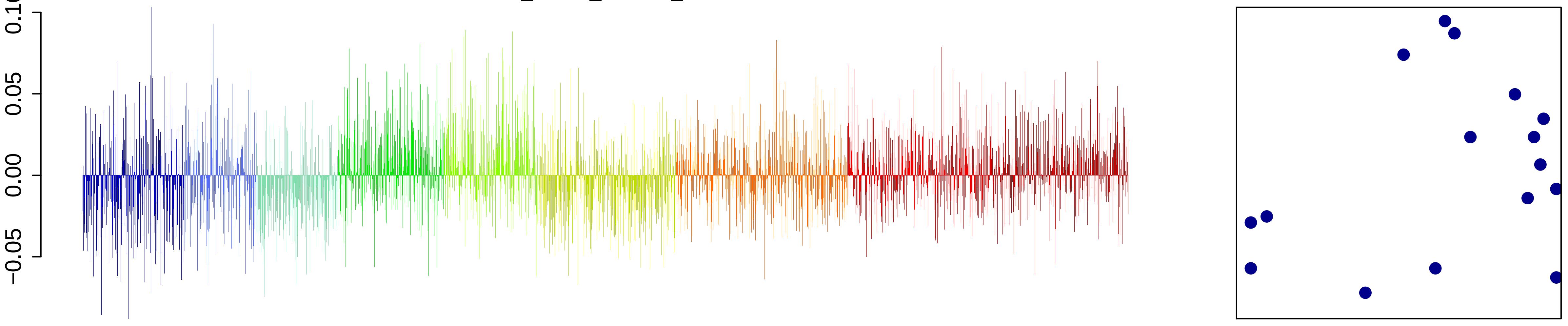

Altman_blood_M16.39_Limbic System

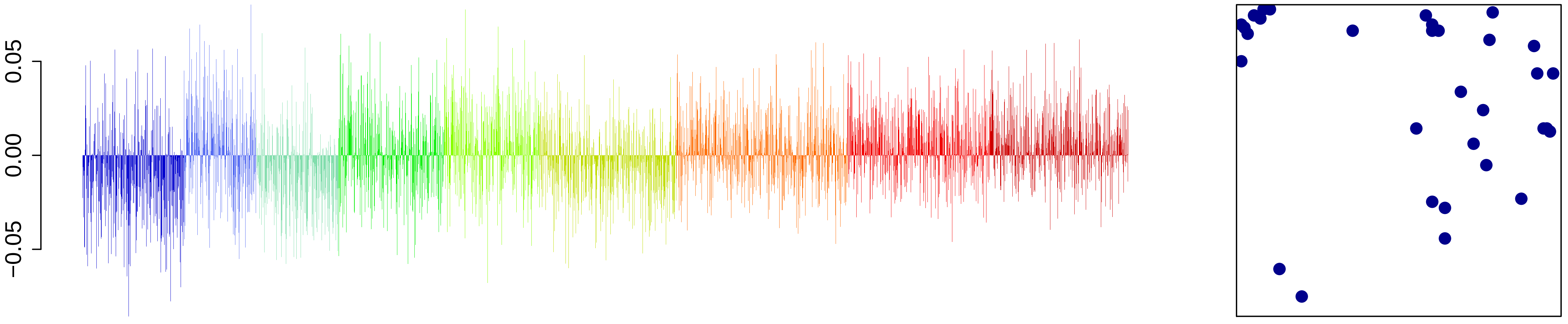

Altman_blood_M16.40_Protein Structure, Tertiary

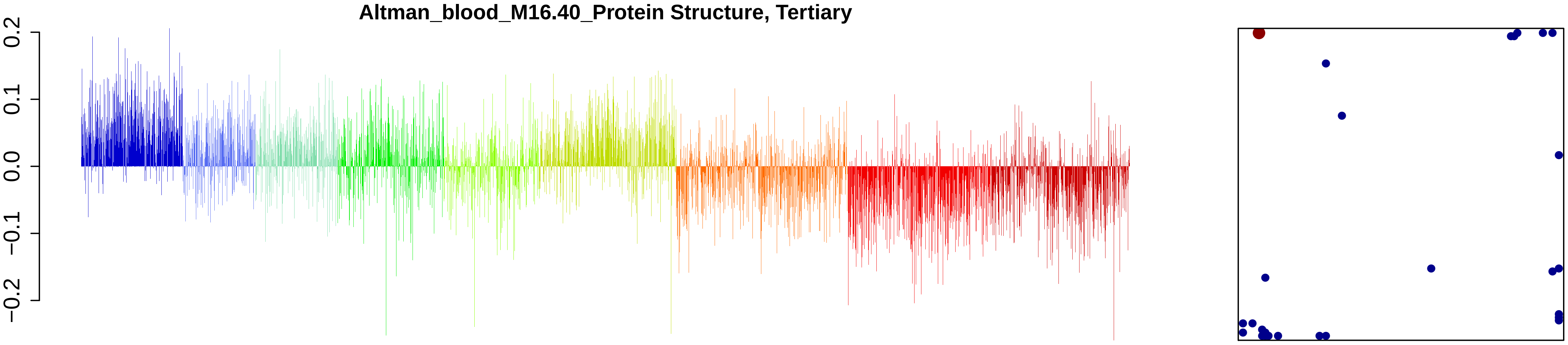

Altman_blood_M16.41_Phylogeny

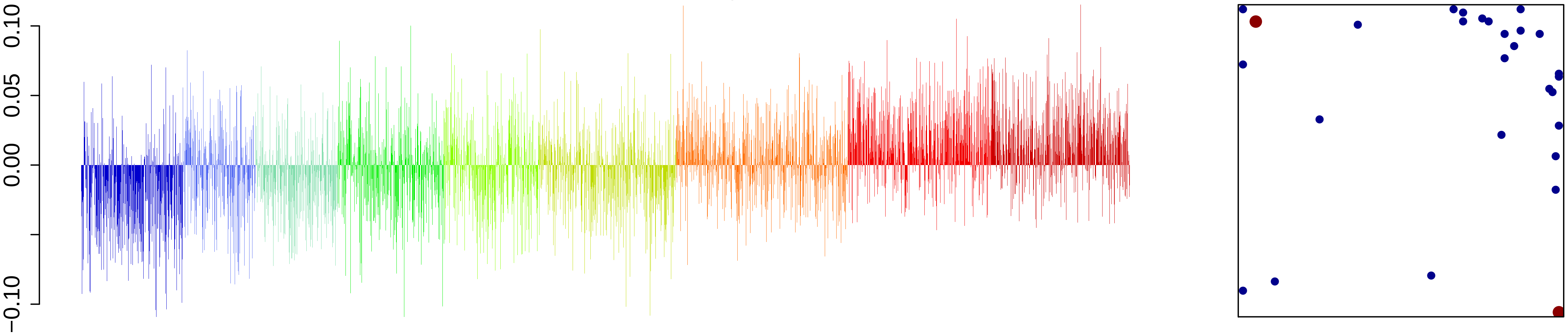

Altman_blood_M16.42_mRNA processing

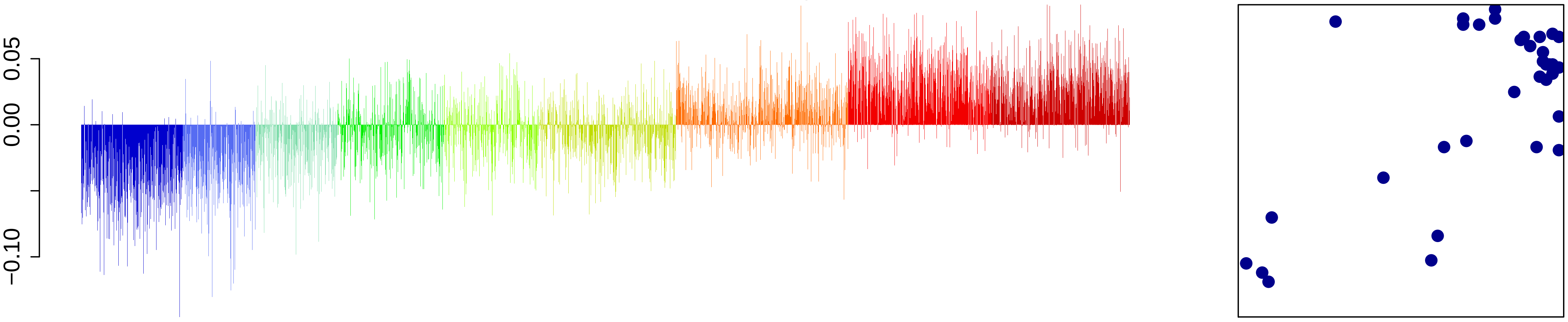

Altman_blood_M16.43_Genetic Predisposition to Disease

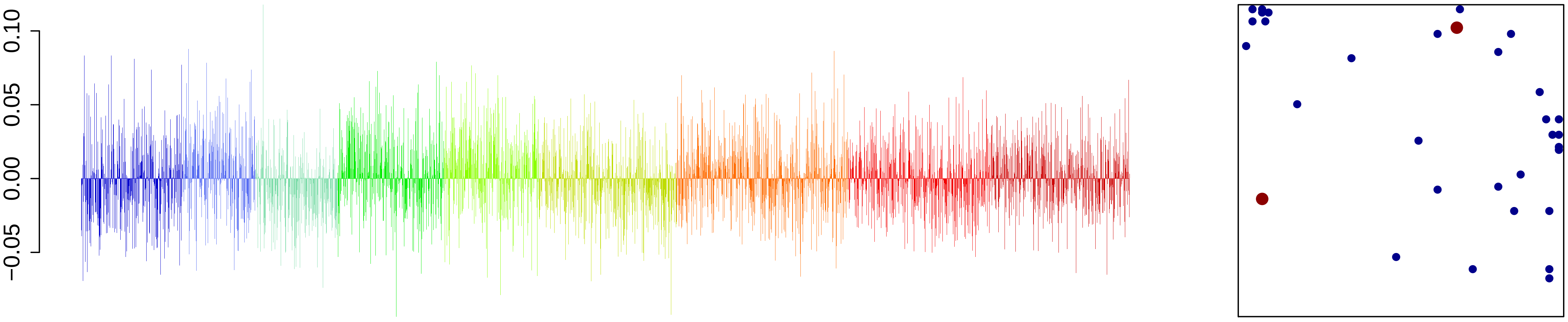

Altman_blood_M16.44_Mitogen−Activated Protein Kinase Phosphatases

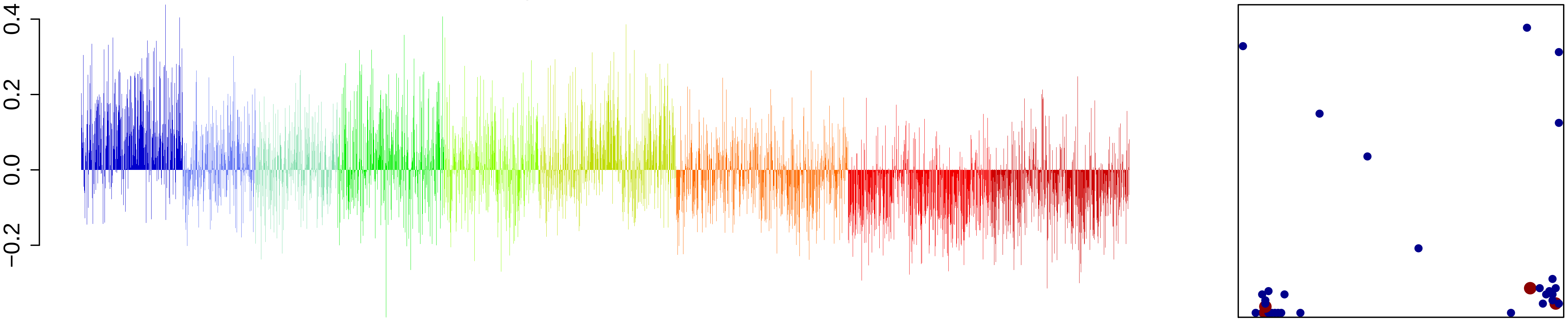

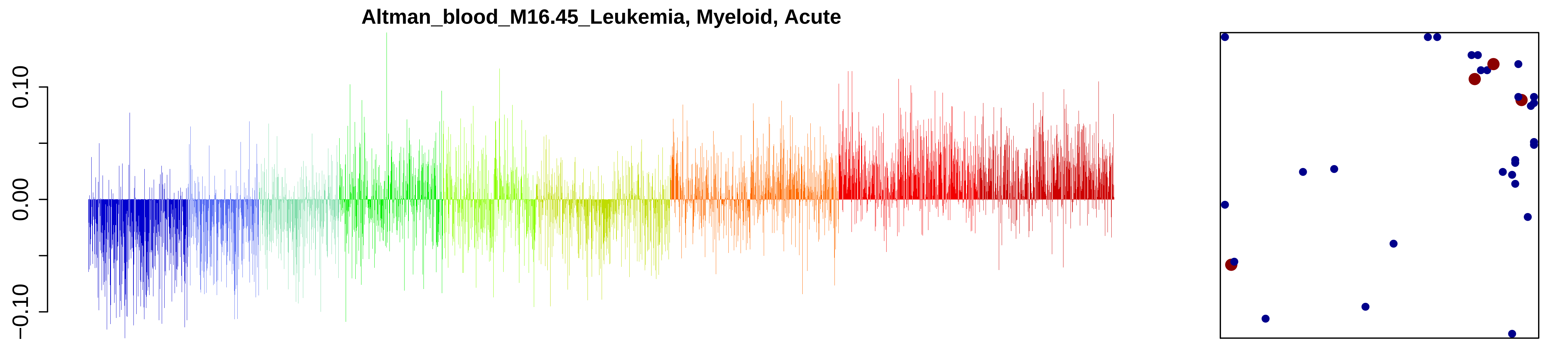
Altman_blood_M16.45_Leukemia, Myeloid, Acute
0.10
0.00
−0.10

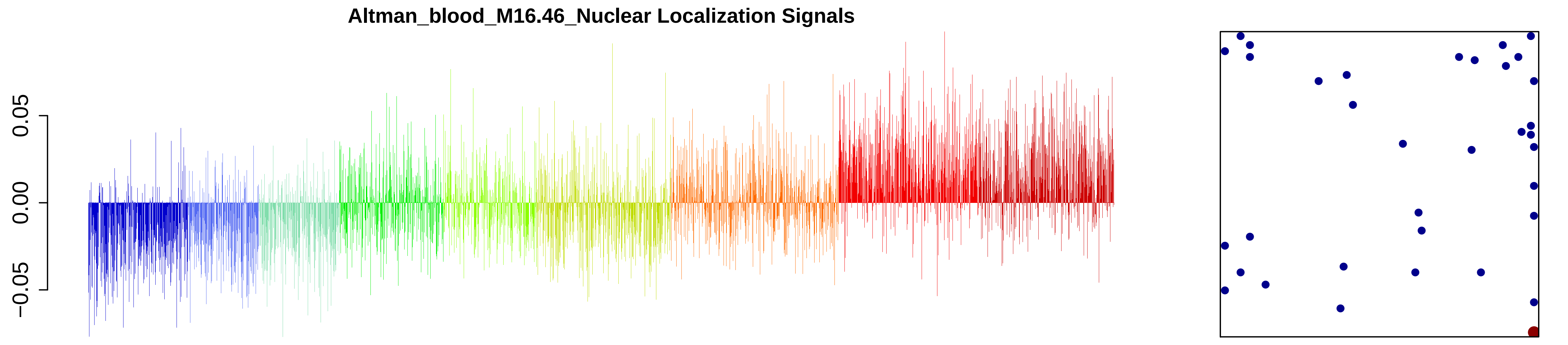
Altman_blood_M16.46_Nuclear Localization Signals
0.05
0.00
−0.05

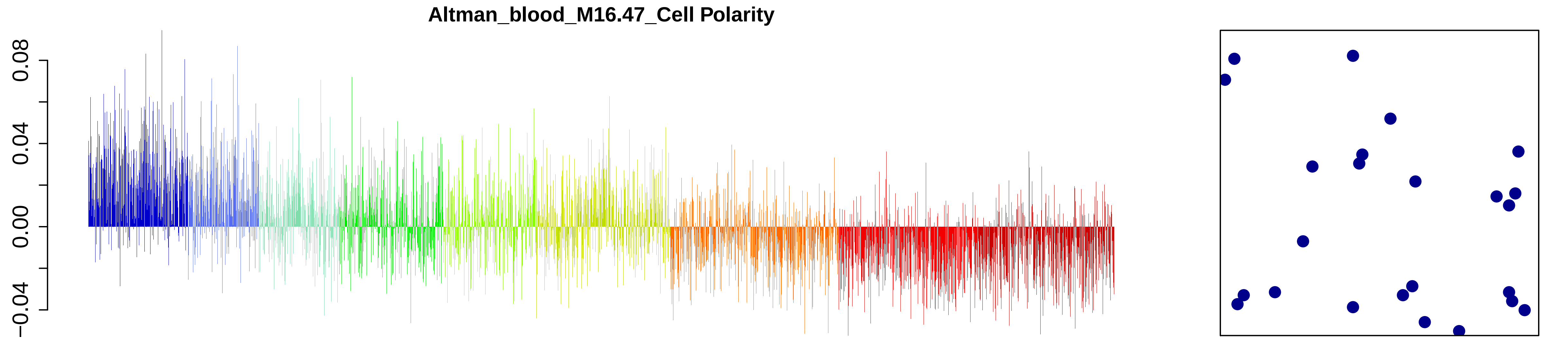
Altman_blood_M16.47_Cell Polarity
0.08
0.04
0.00
−0.04

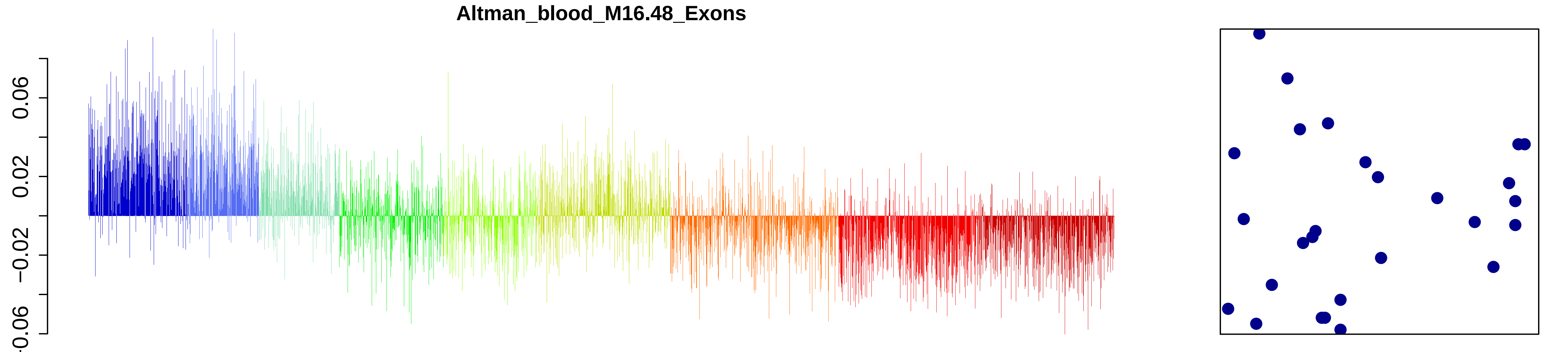
Altman_blood_M16.48_Exons
0.06
0.02
−0.02
−0.06

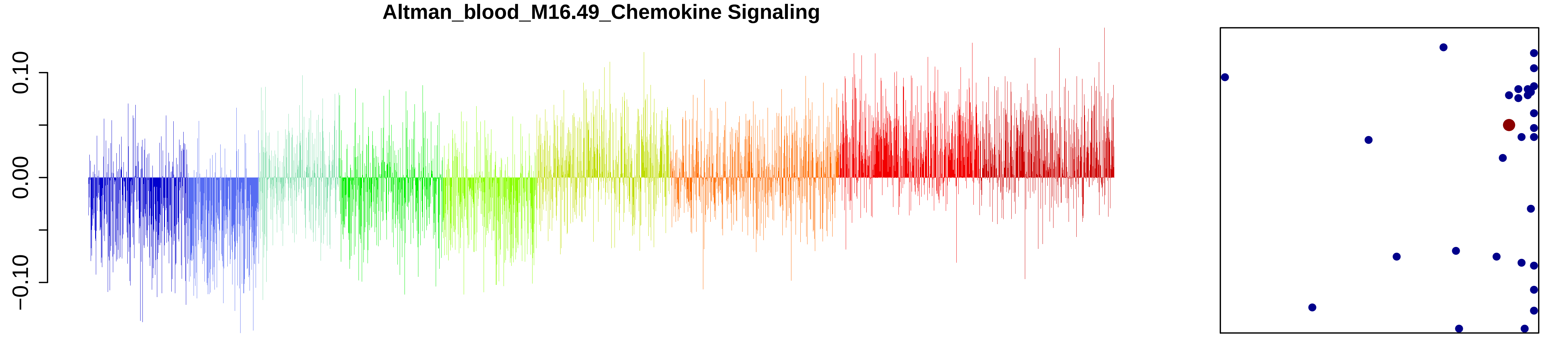
Altman_blood_M16.49_Chemokine Signaling
0.10
0.00
−0.10

Altman_blood_M16.50_Cell Movement

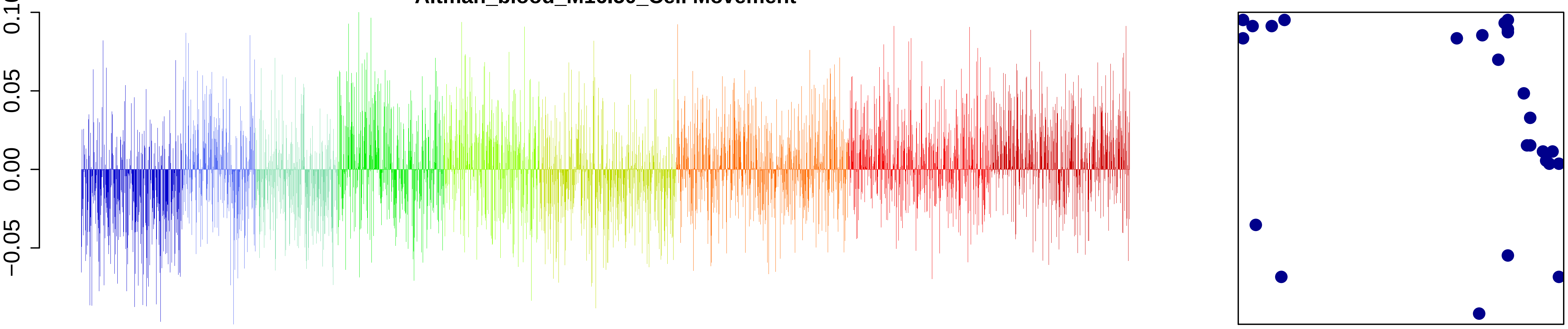

Altman_blood_M16.51_Binding Sites

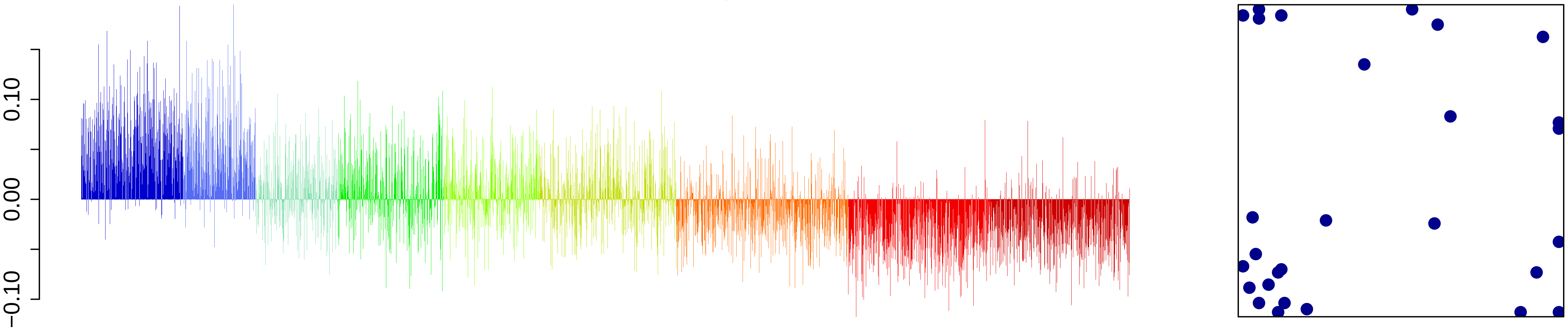

Altman_blood_M16.52_Chromosomes, Human, 6−12 and X

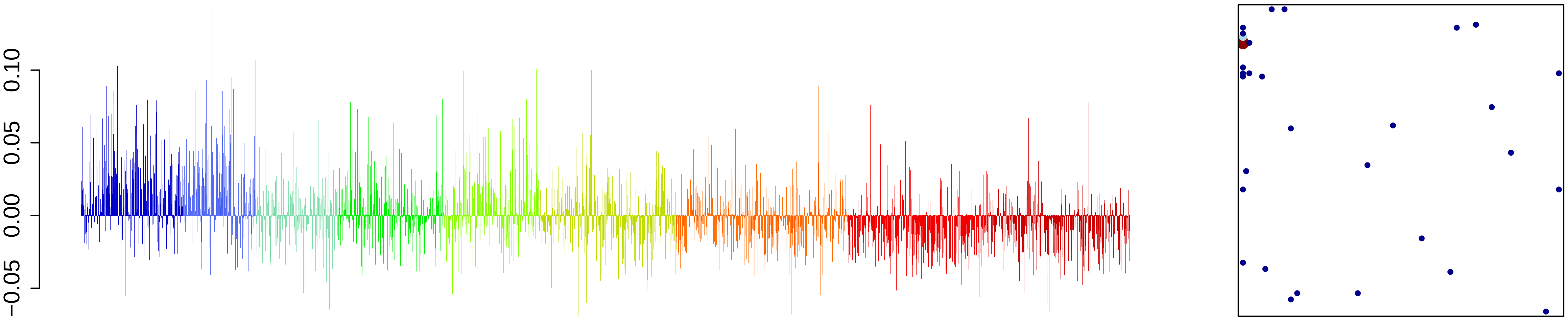

Altman_blood_M16.53_Genome

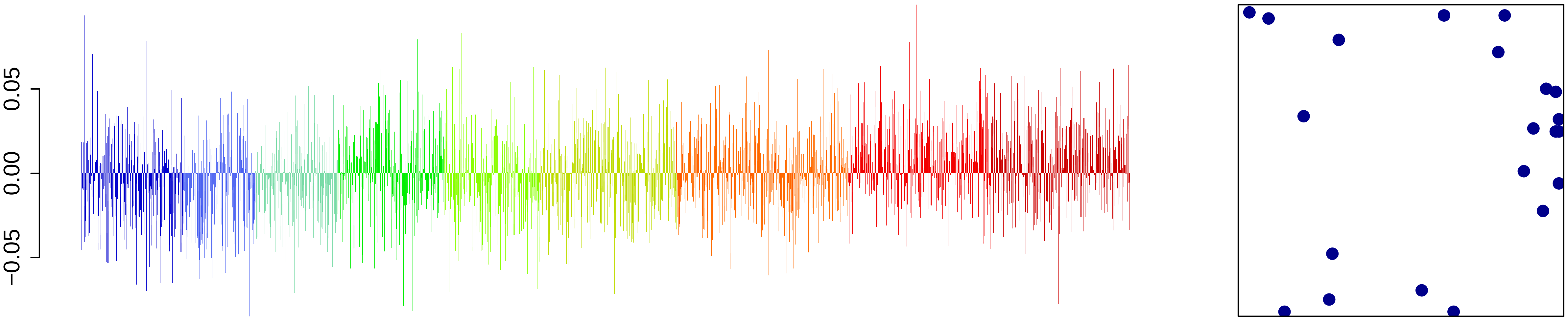

Altman_blood_M16.54_Genetic Phenomena

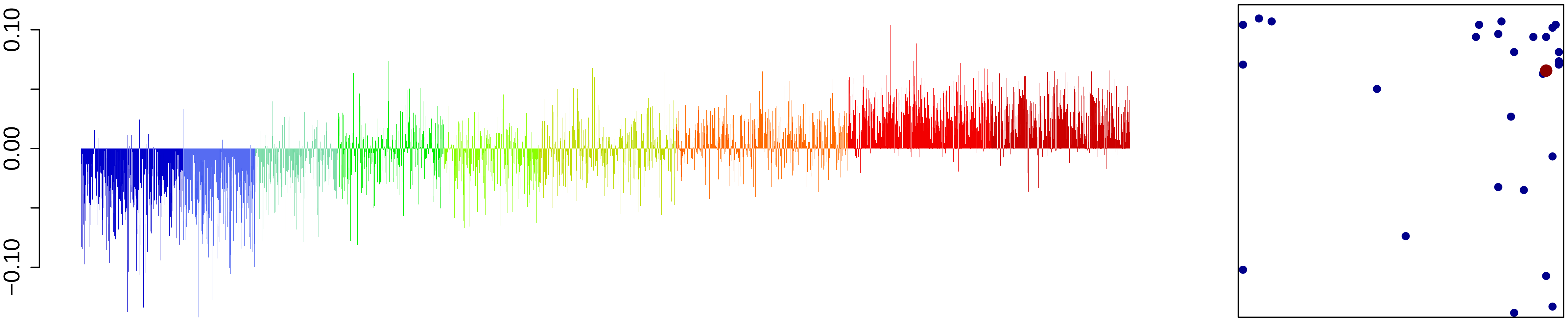

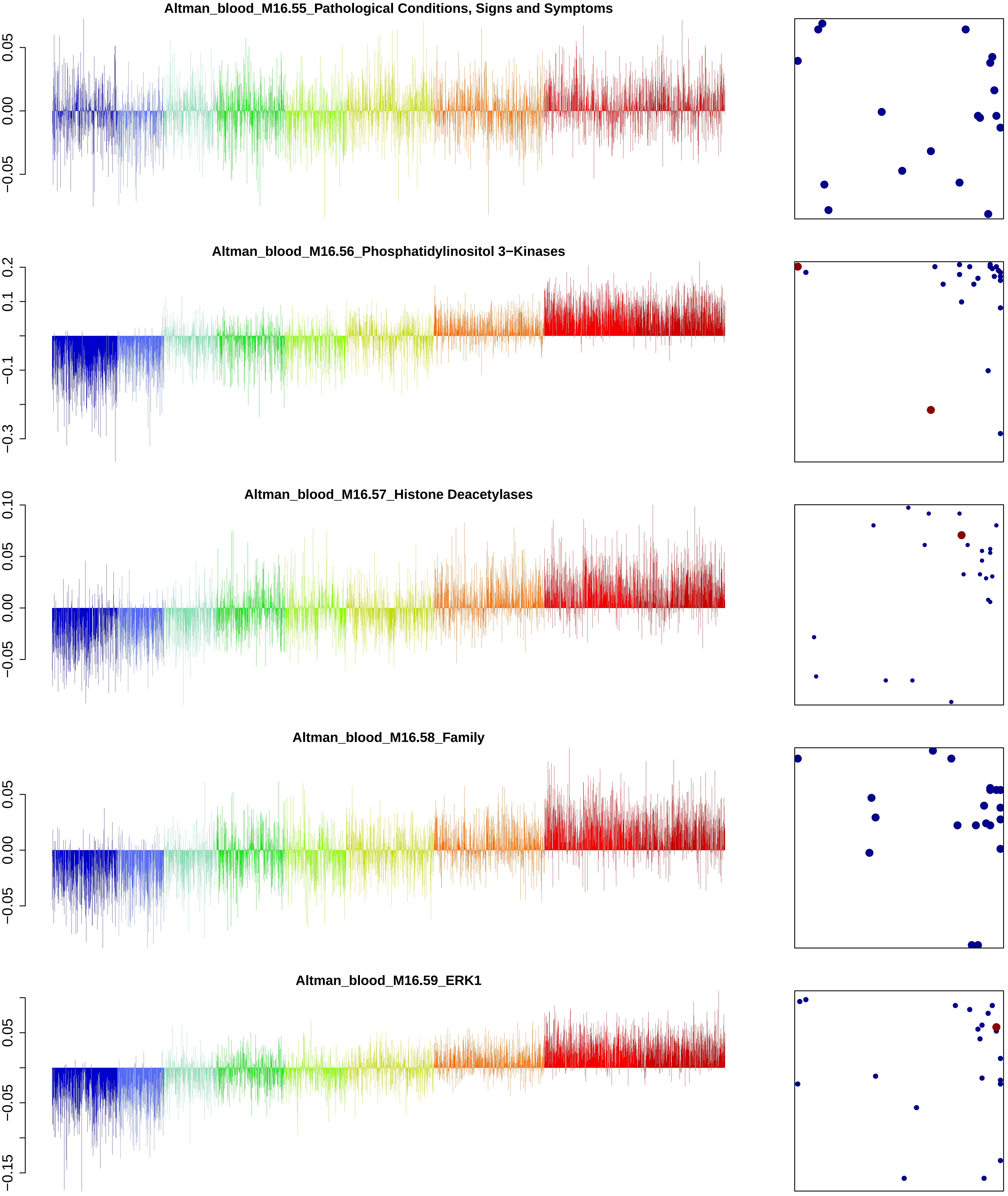
Altman_blood_M16.55_Pathological Conditions, Signs and Symptoms
0.05
0.00
−0.05
Altman_blood_M16.56_Phosphatidylinositol 3−Kinases
0.2
0.1
−0.1
−0.3
Altman_blood_M16.57_Histone Deacetylases
0.10
0.05
0.00
−0.05
Altman_blood_M16.58_Family
0.05
0.00
−0.05
Altman_blood_M16.59_ERK1
0.05
−0.05
−0.15

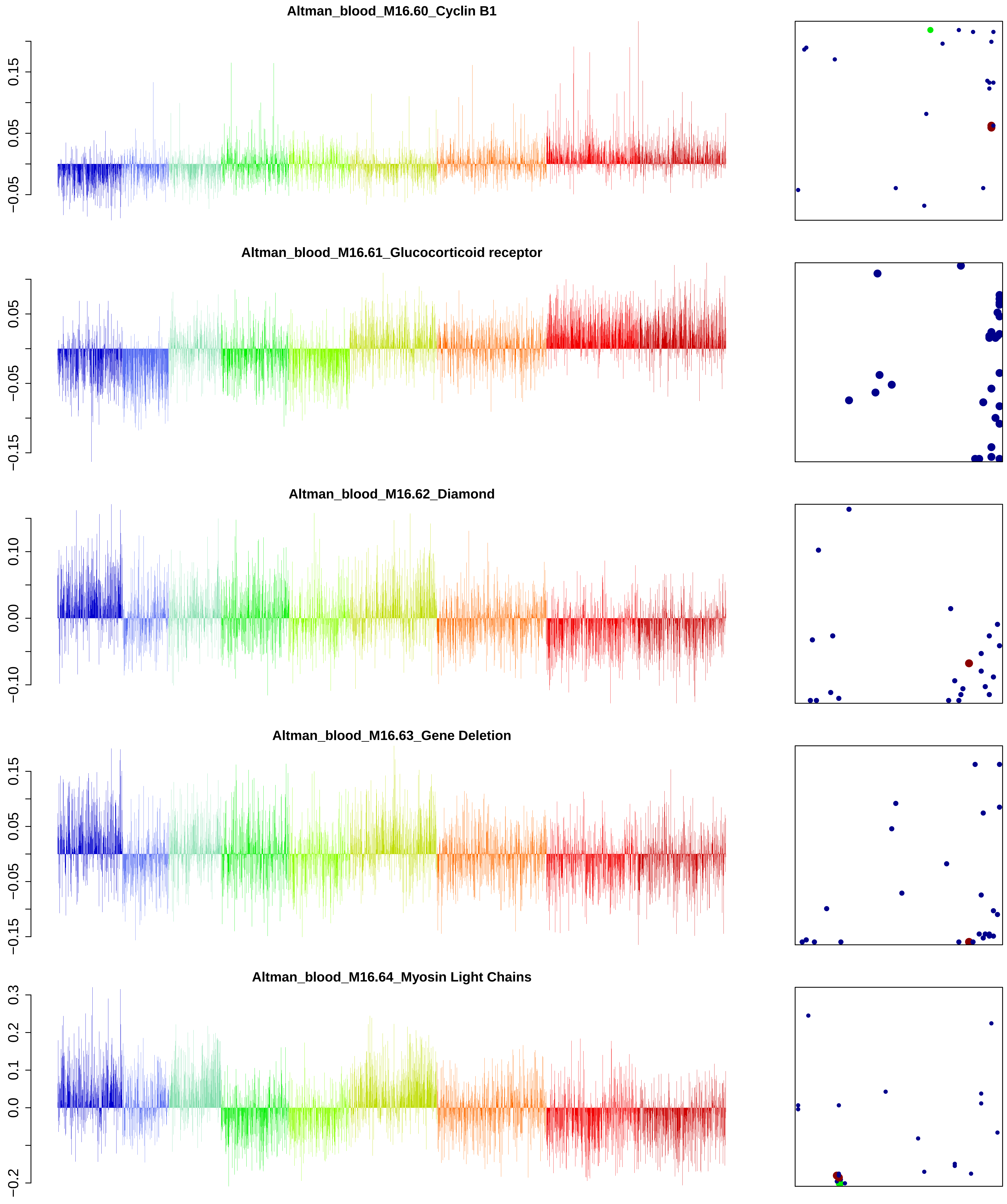
Altman_blood_M16.60_Cyclin B1
−0.05
0.05
0.15
Altman_blood_M16.61_Glucocorticoid receptor
−0.15
−0.05
0.05
Altman_blood_M16.62_Diamond
−0.10
0.00
0.10
Altman_blood_M16.63_Gene Deletion
−0.15
−0.05
0.05
0.15
Altman_blood_M16.64_Myosin Light Chains
−0.2
0.0
0.1
0.2
0.3

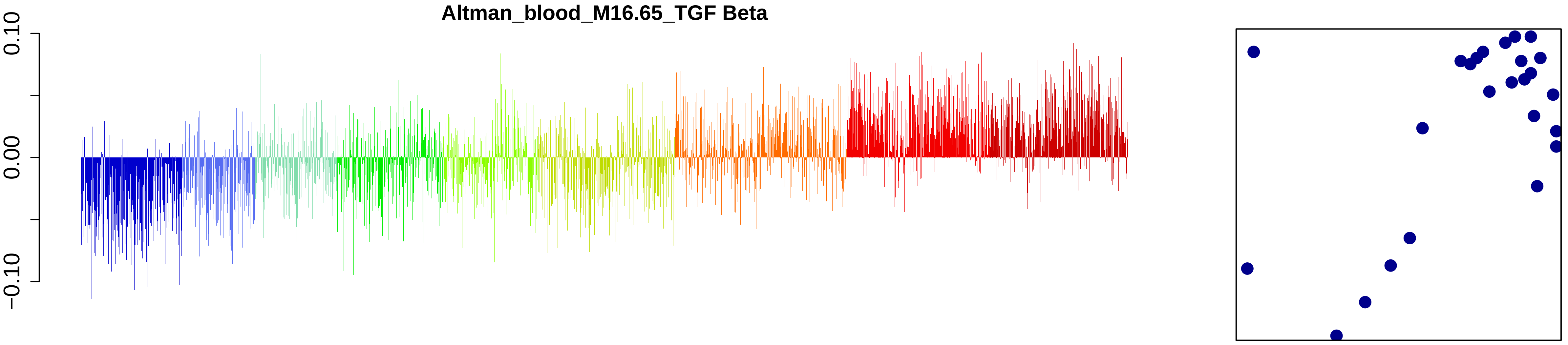
Altman_blood_M16.65_TGF Beta
0.10
0.00
−0.10

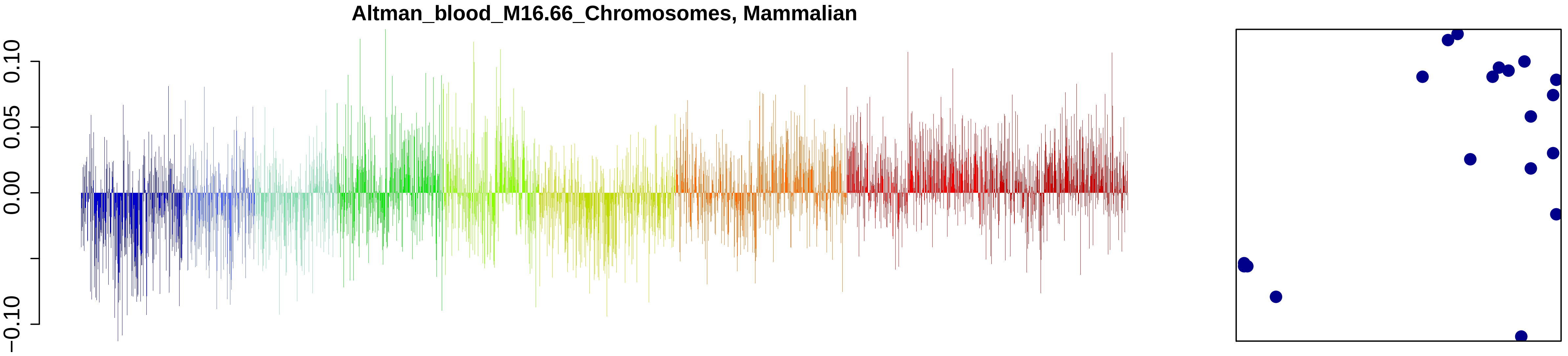
Altman_blood_M16.66_Chromosomes, Mammalian
0.10
0.05
0.00
−0.10

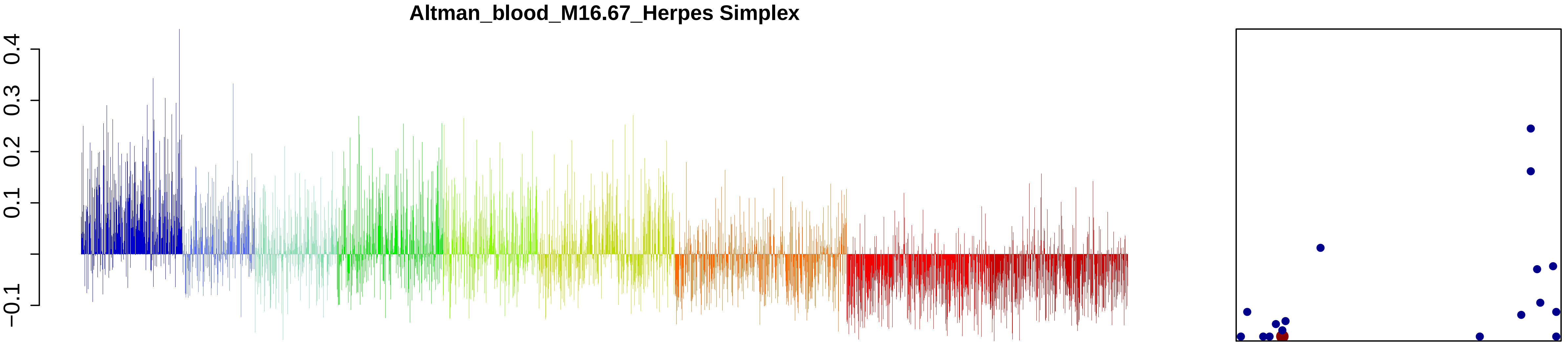
Altman_blood_M16.67_Herpes Simplex
0.4
0.3
0.2
0.1
−0.1

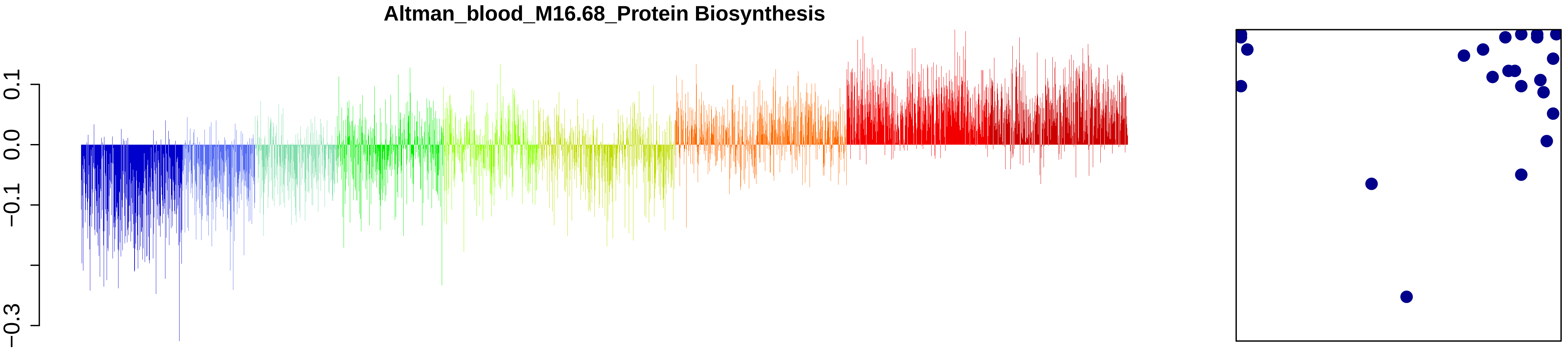
Altman_blood_M16.68_Protein Biosynthesis
0.1
0.0
−0.1
−0.3

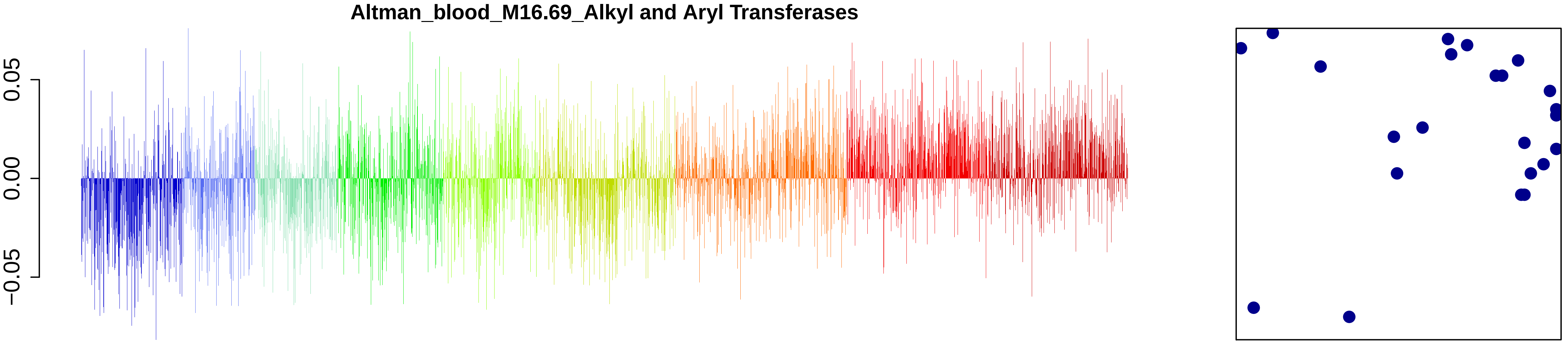
Altman_blood_M16.69_Alkyl and Aryl Transferases
0.05
0.00
−0.05

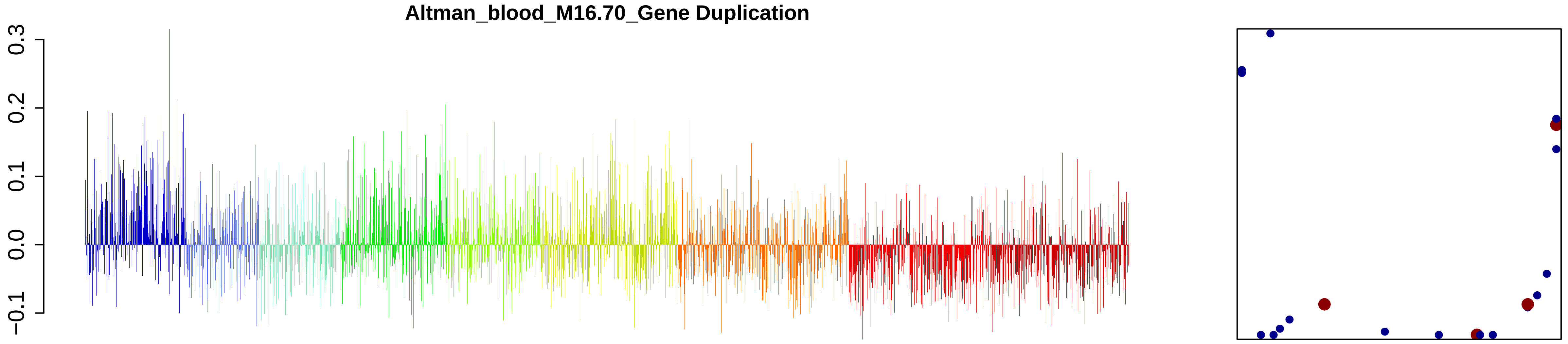
Altman_blood_M16.70_Gene Duplication
0.3
0.2
0.1
0.0
−0.1

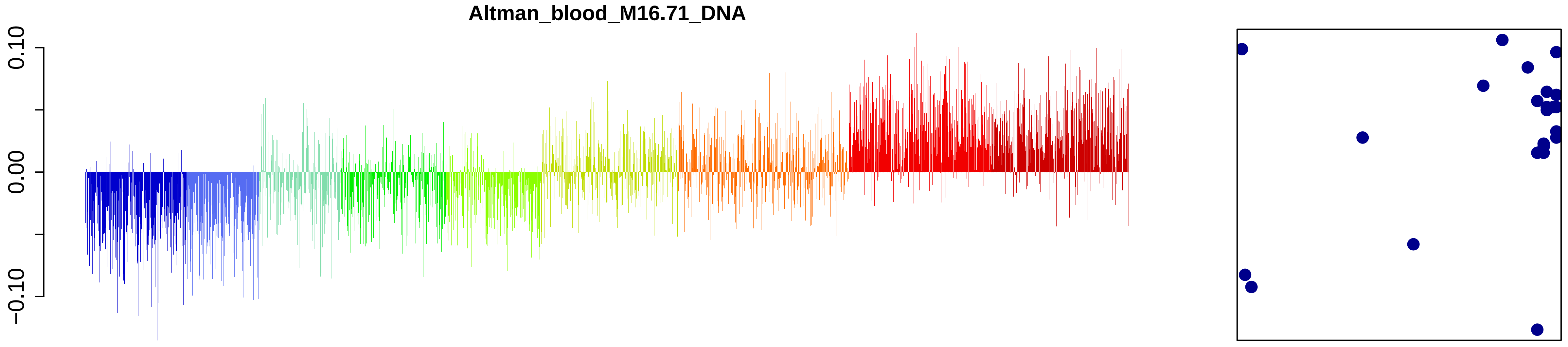
Altman_blood_M16.71_DNA
0.10
0.00
−0.10

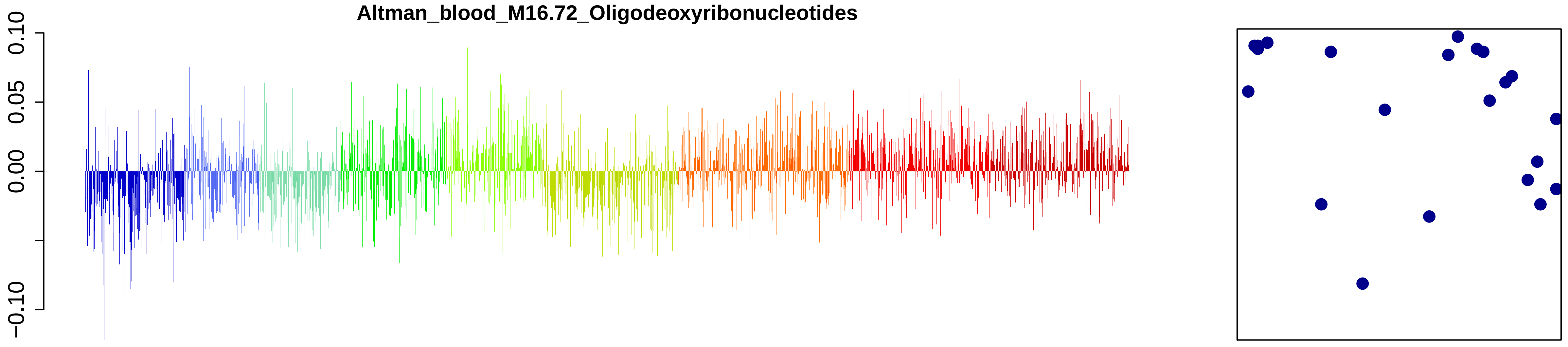
Altman_blood_M16.72_Oligodeoxyribonucleotides
0.10
0.05
0.00
−0.10

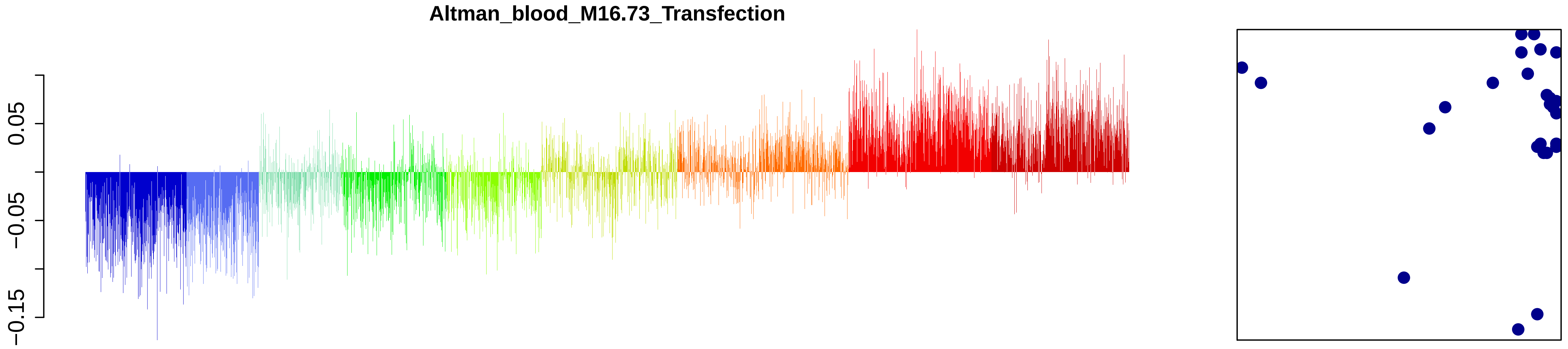
Altman_blood_M16.73_Transfection
0.05
−0.05
−0.15

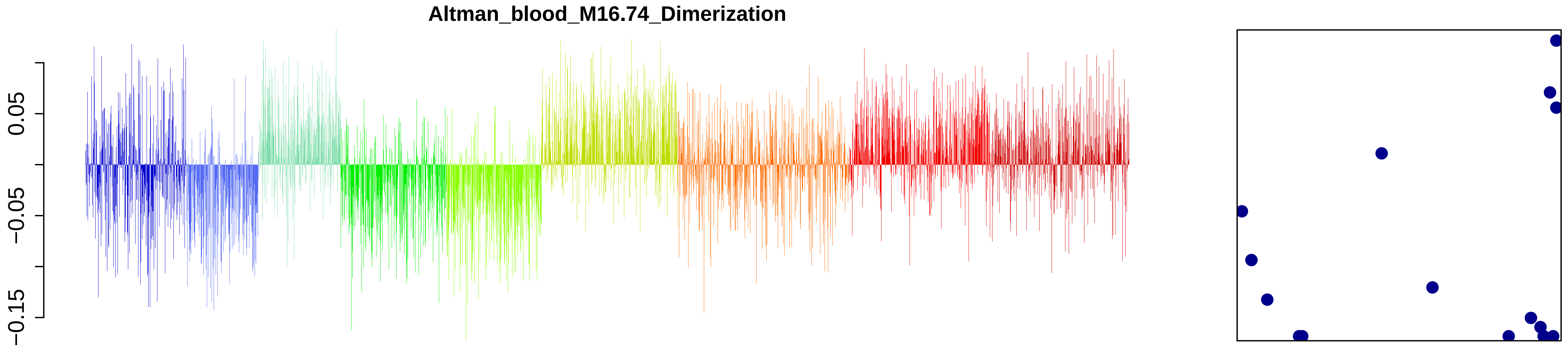
Altman_blood_M16.74_Dimerization
0.05
−0.05
−0.15

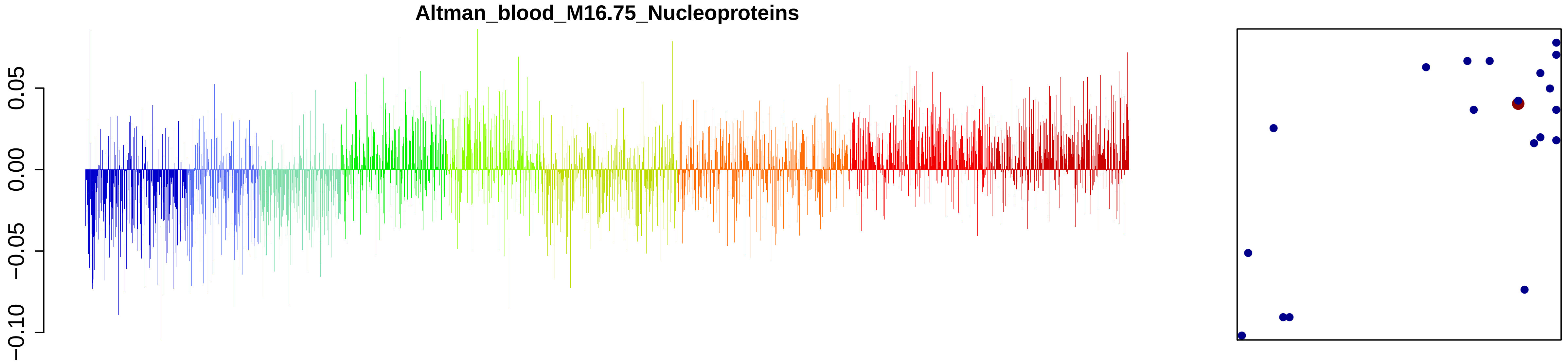
Altman_blood_M16.75_Nucleoproteins
0.05
0.00
−0.05
−0.10

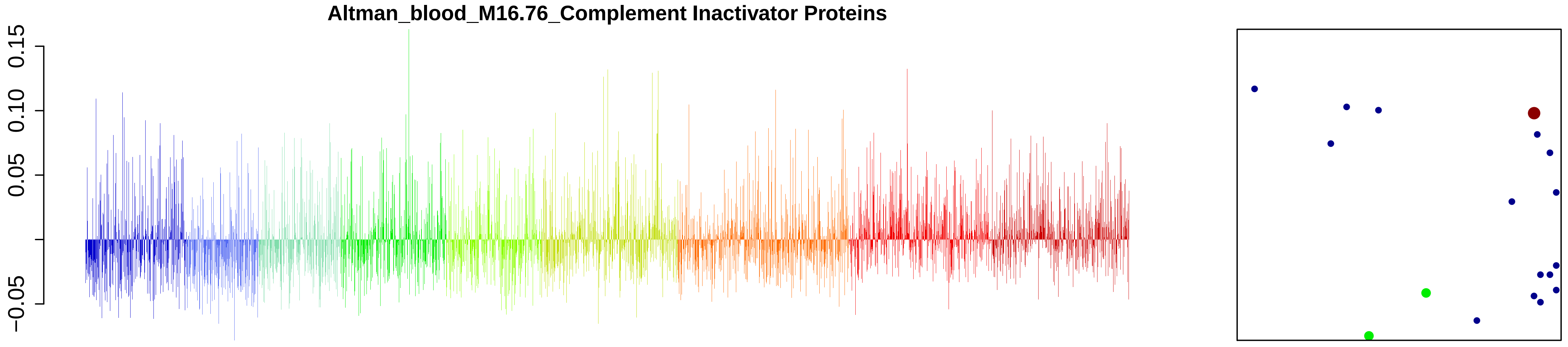
Altman_blood_M16.76_Complement Inactivator Proteins
0.15
0.10
0.05
−0.05

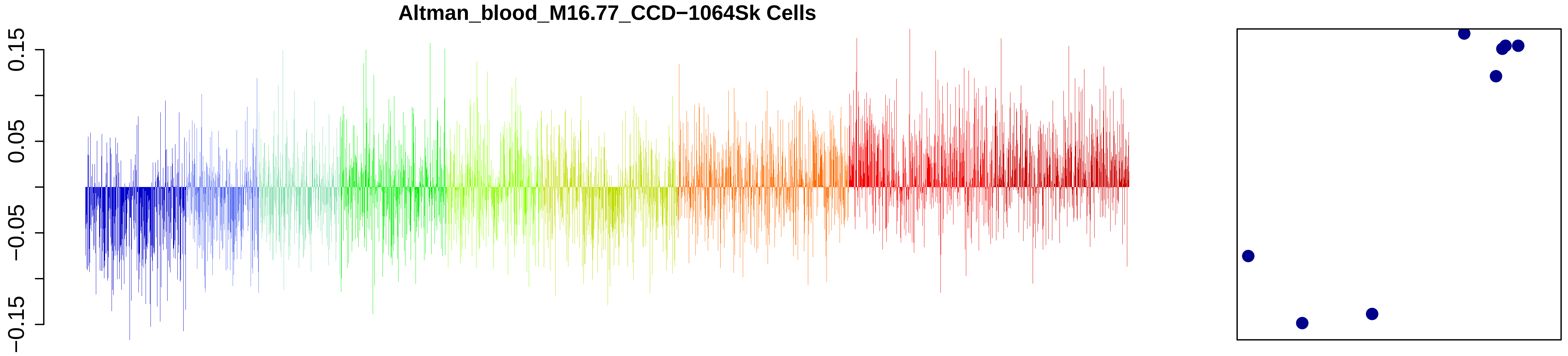
Altman_blood_M16.77_CCD−1064Sk Cells
0.15
0.05
−0.05
−0.15

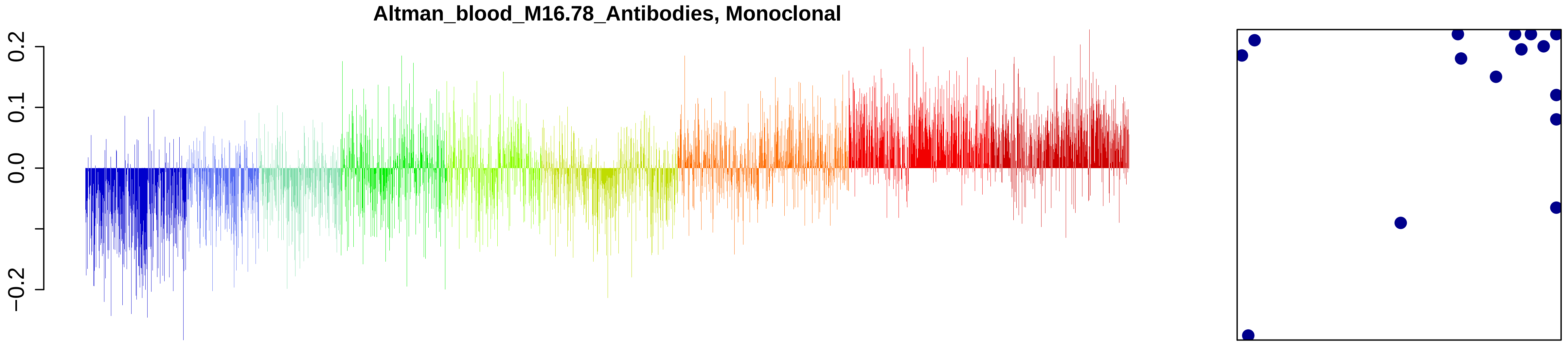
Altman_blood_M16.78_Antibodies, Monoclonal
0.2
0.1
0.0
−0.2

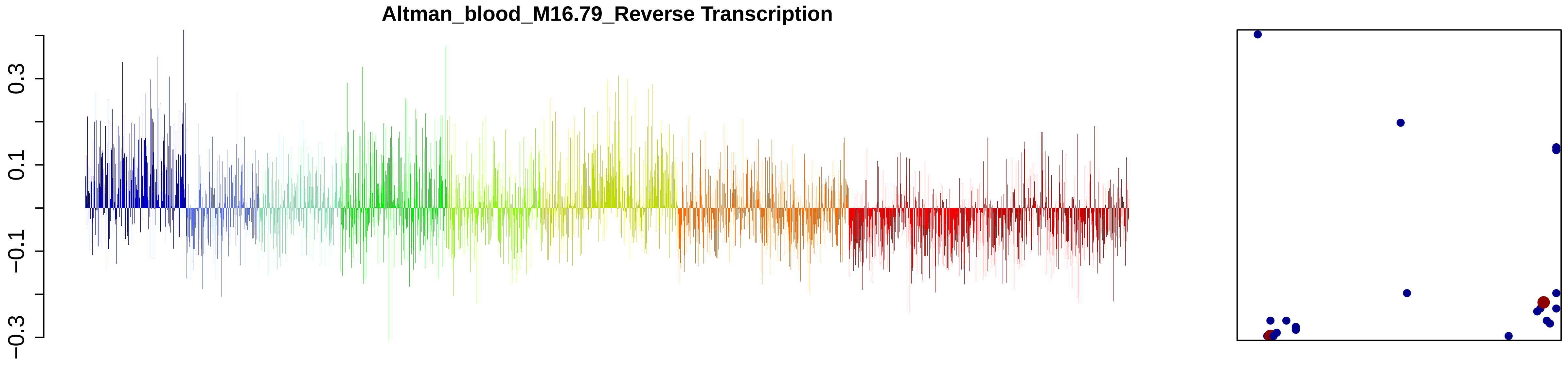
Altman_blood_M16.79_Reverse Transcription
0.3
0.1
−0.1
−0.3

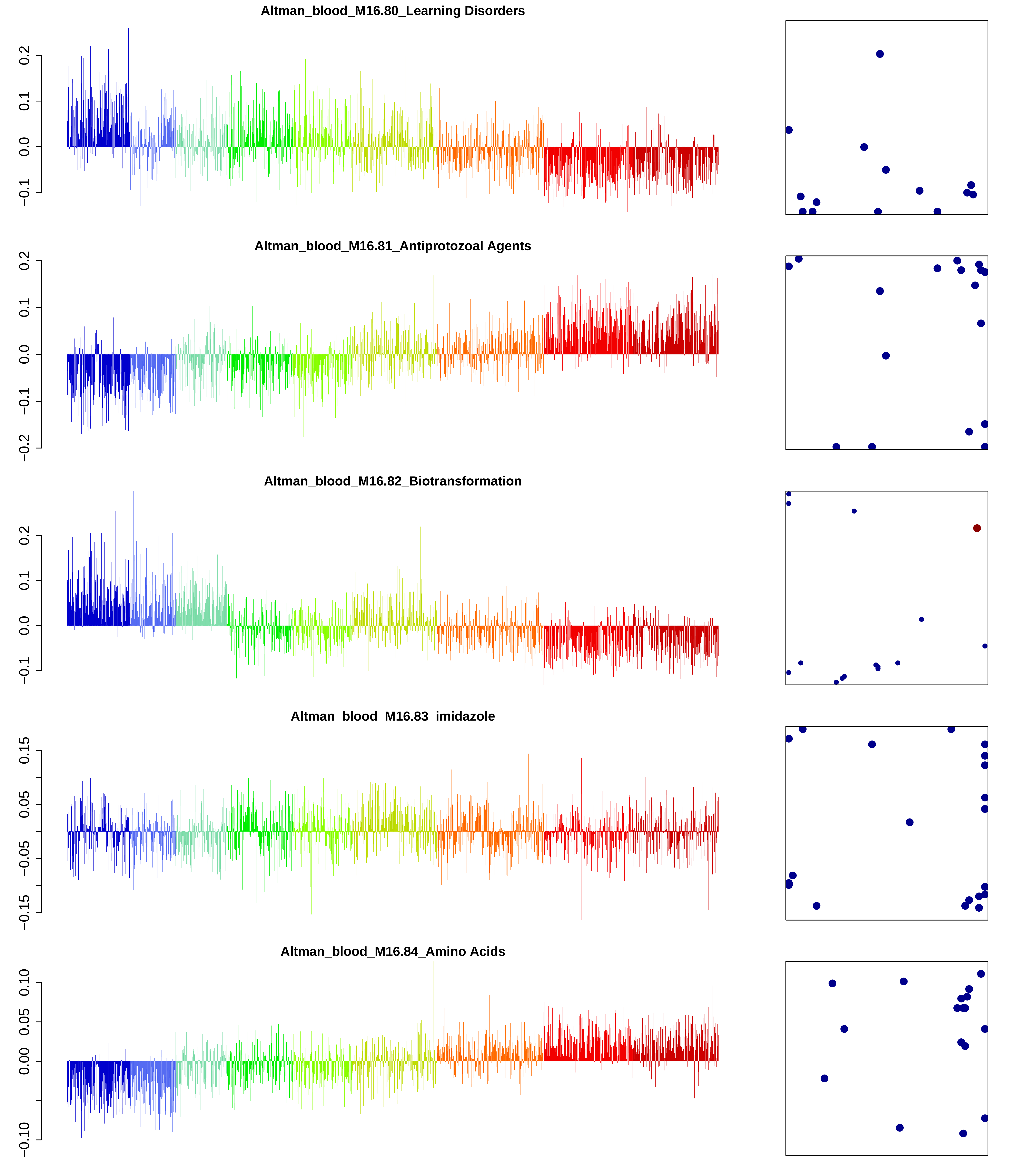
Altman_blood_M16.80_Learning Disorders
0.2
0.1
0.0
−0.1
Altman_blood_M16.81_Antiprotozoal Agents
0.2
0.1
0.0
−0.1
−0.2
Altman_blood_M16.82_Biotransformation
0.2
0.1
0.0
−0.1
Altman_blood_M16.83_imidazole
0.15
0.05
−0.05
−0.15
Altman_blood_M16.84_Amino Acids
0.10
0.05
0.00
−0.10

Altman_blood_M16.85_Xanthine

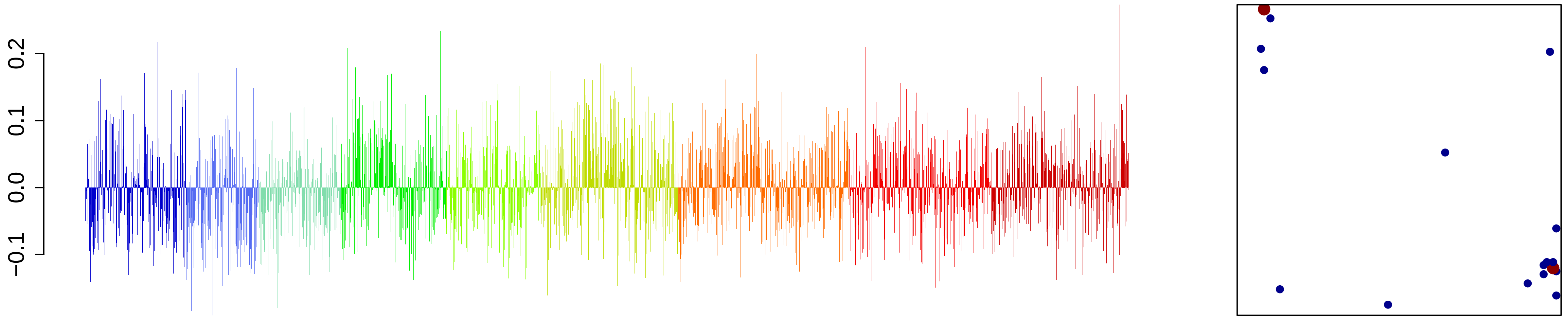

Altman_blood_M16.86_Oxygen

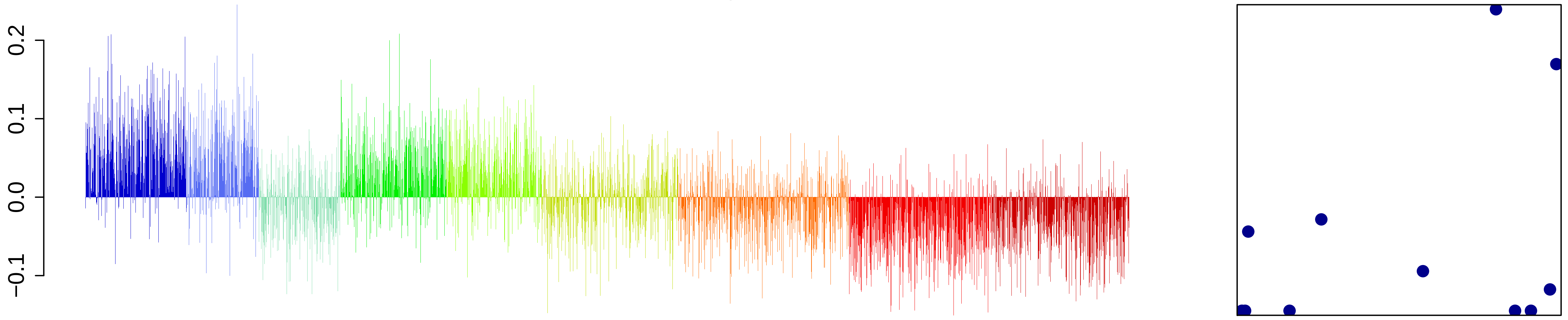

Altman_blood_M16.87_Cell Line

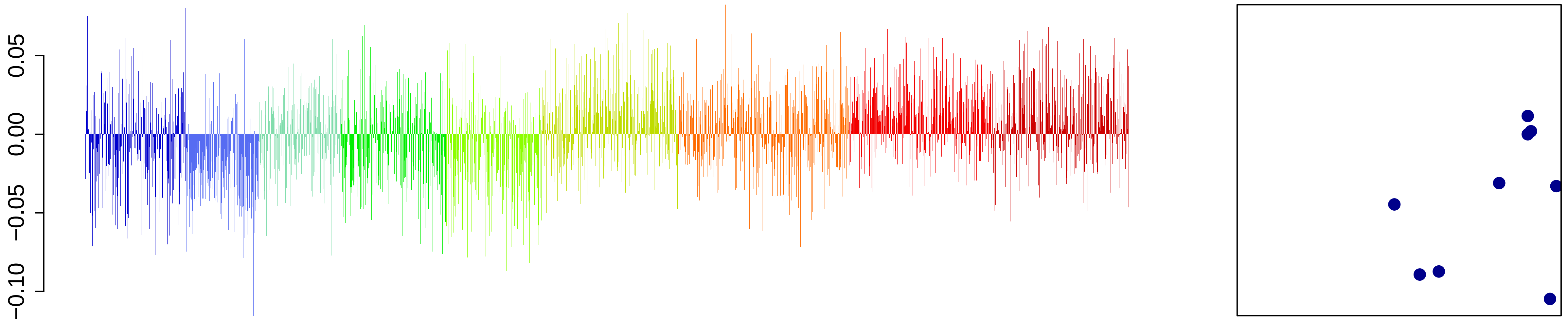

Altman_blood_M16.88_DNA, Single−Stranded

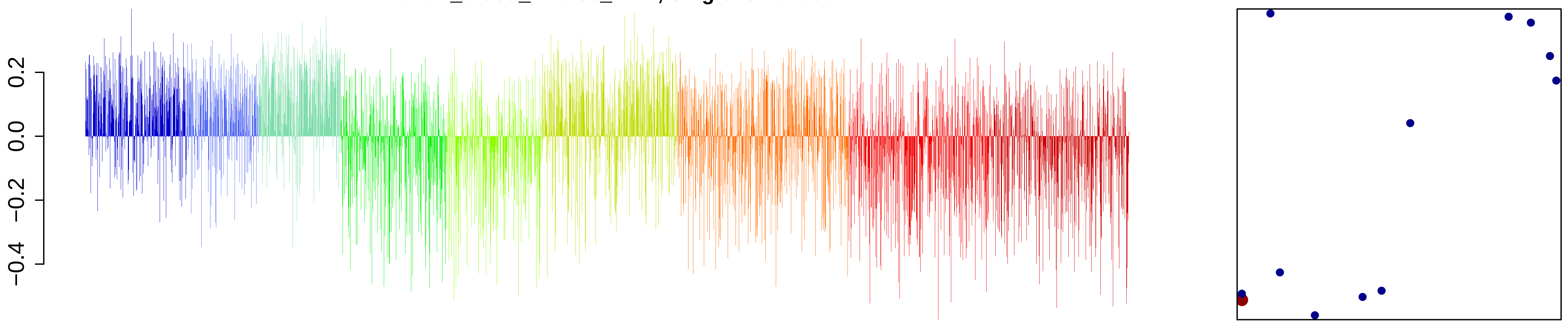

Altman_blood_M16.89_Sclerosis

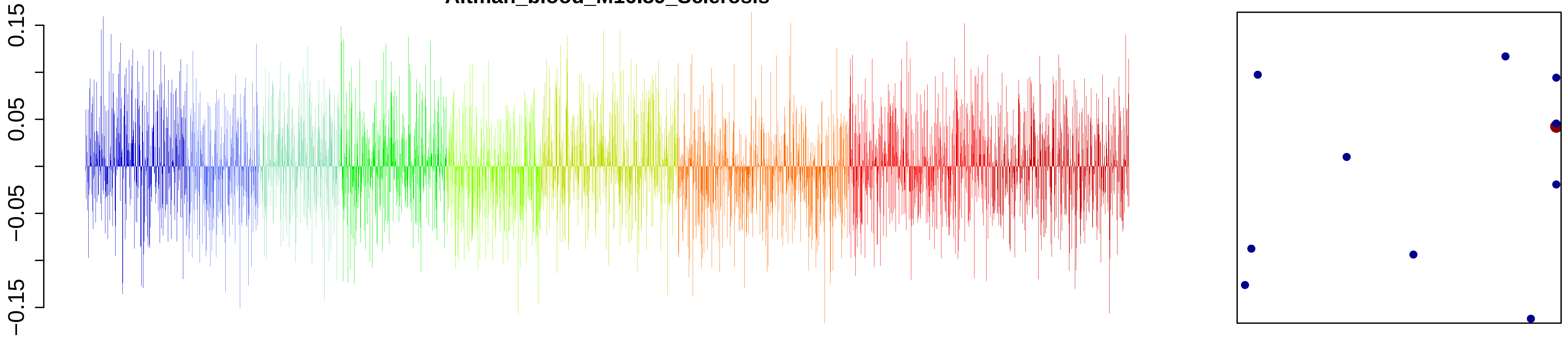

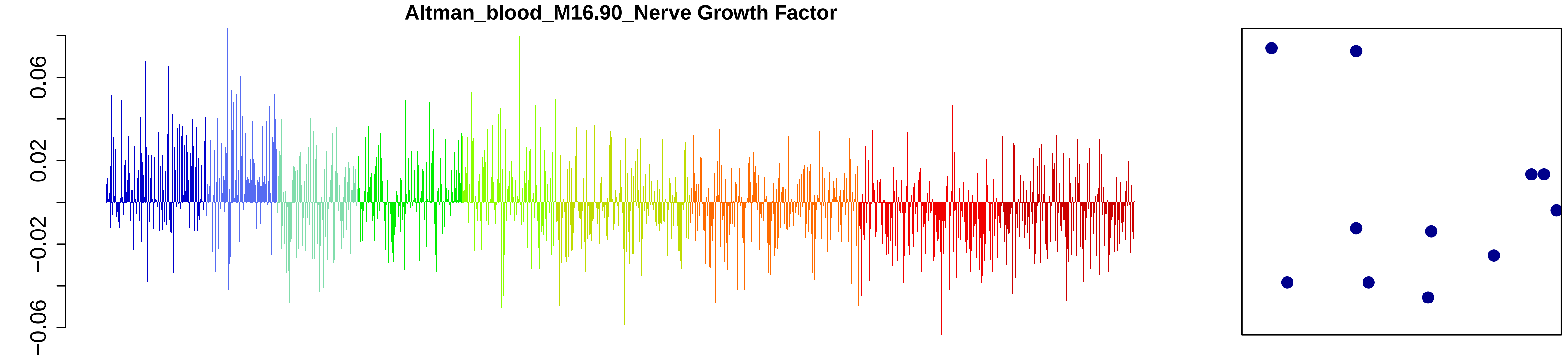
Altman_blood_M16.90_Nerve Growth Factor
0.06
0.02
−0.02
−0.06

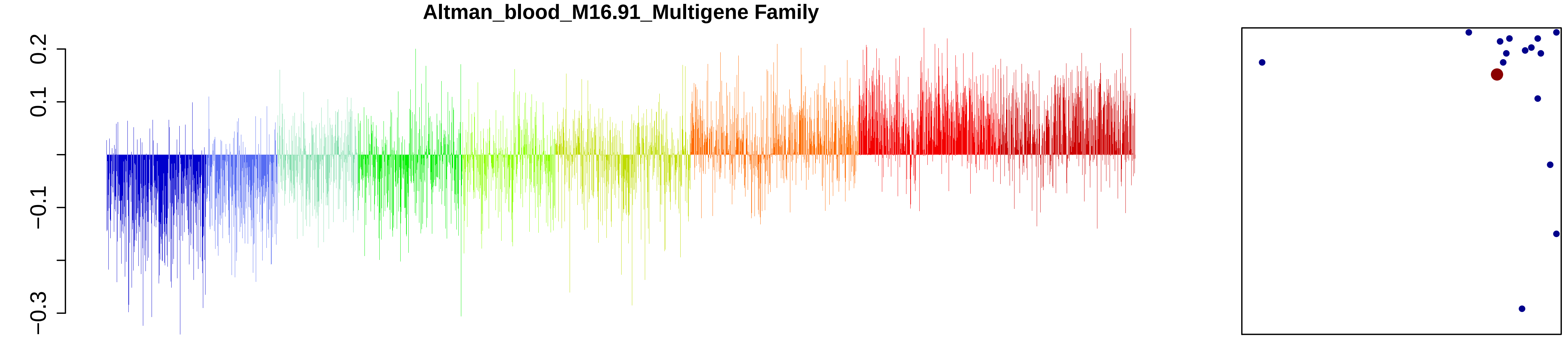
Altman_blood_M16.91_Multigene Family
0.2
0.1
−0.1
−0.3

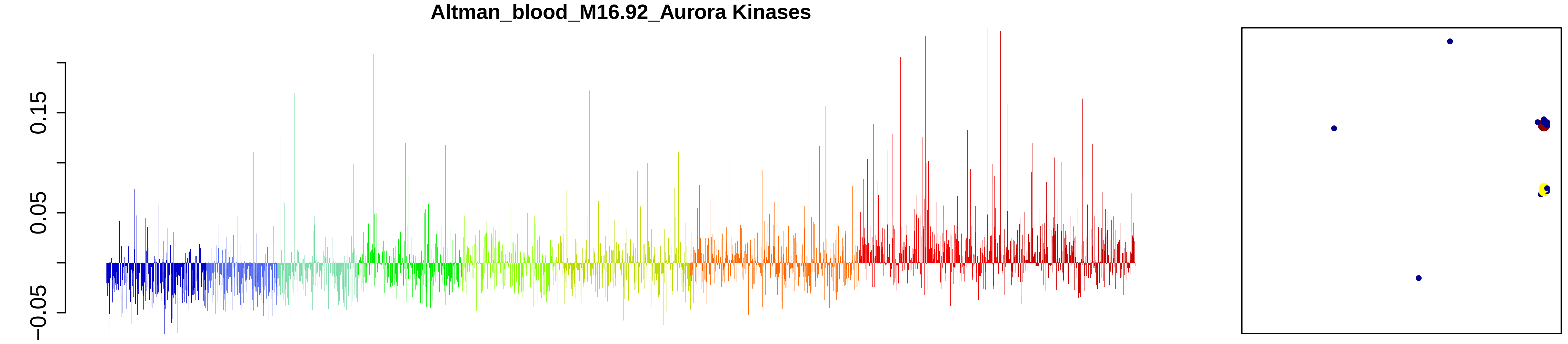
Altman_blood_M16.92_Aurora Kinases
0.15
0.05
−0.05

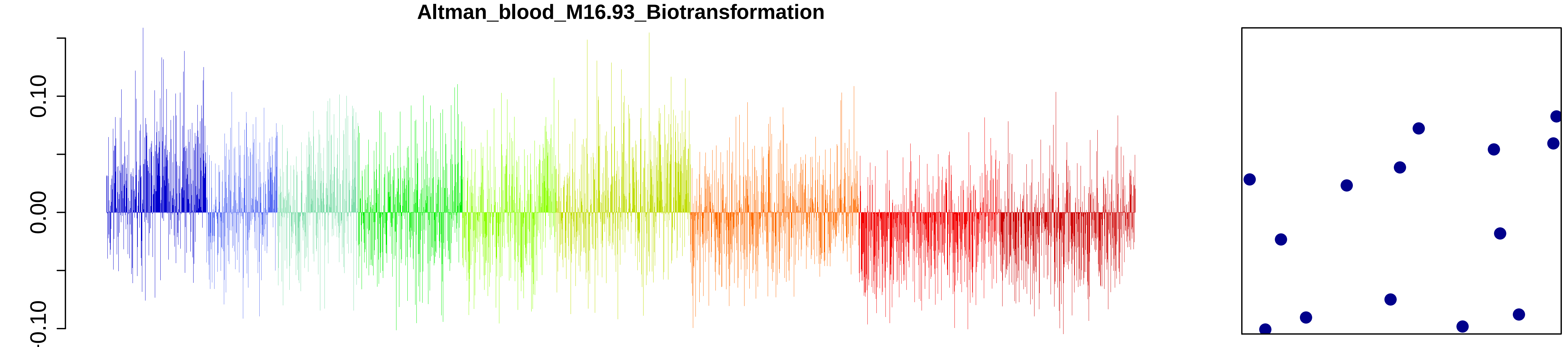
Altman_blood_M16.93_Biotransformation
0.10
0.00
−0.10

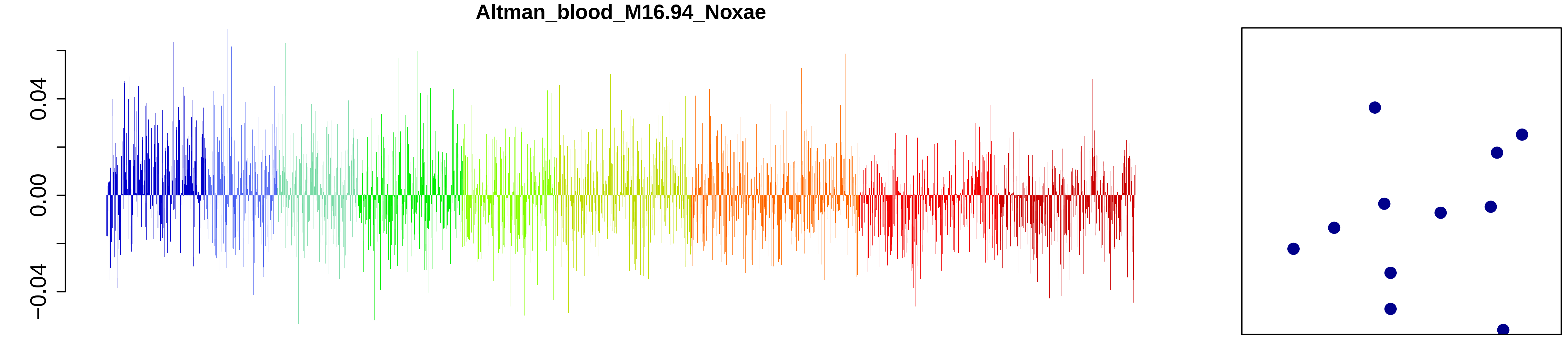
Altman_blood_M16.94_Noxae
0.04
0.00
−0.04

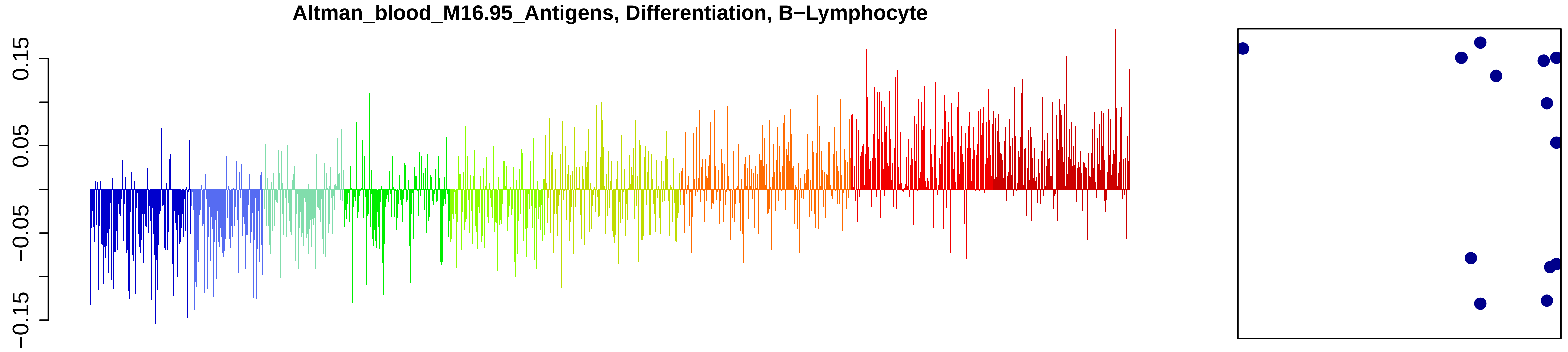
Altman_blood_M16.95_Antigens, Differentiation, B−Lymphocyte
0.15
0.05
−0.05
−0.15

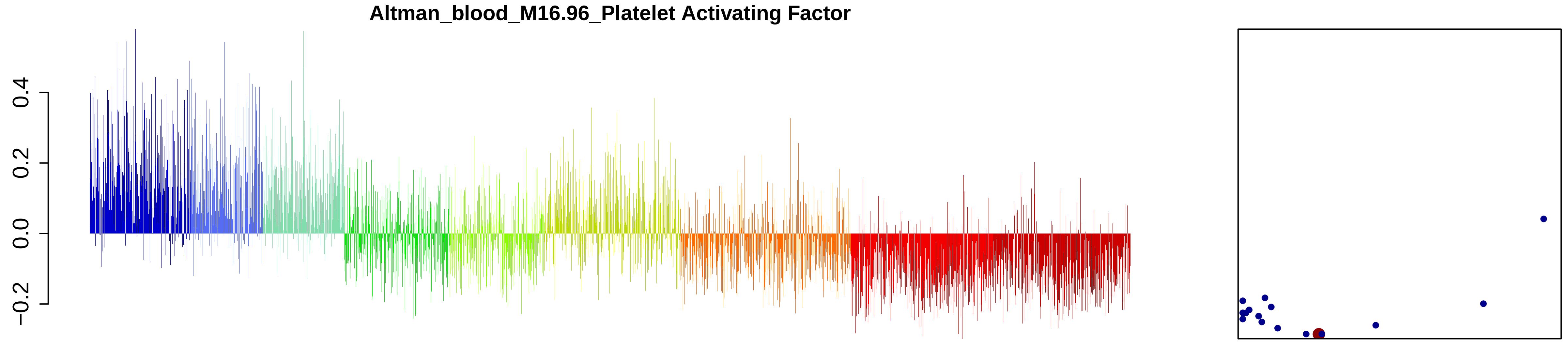
Altman_blood_M16.96_Platelet Activating Factor
0.4
0.2
0.0
−0.2

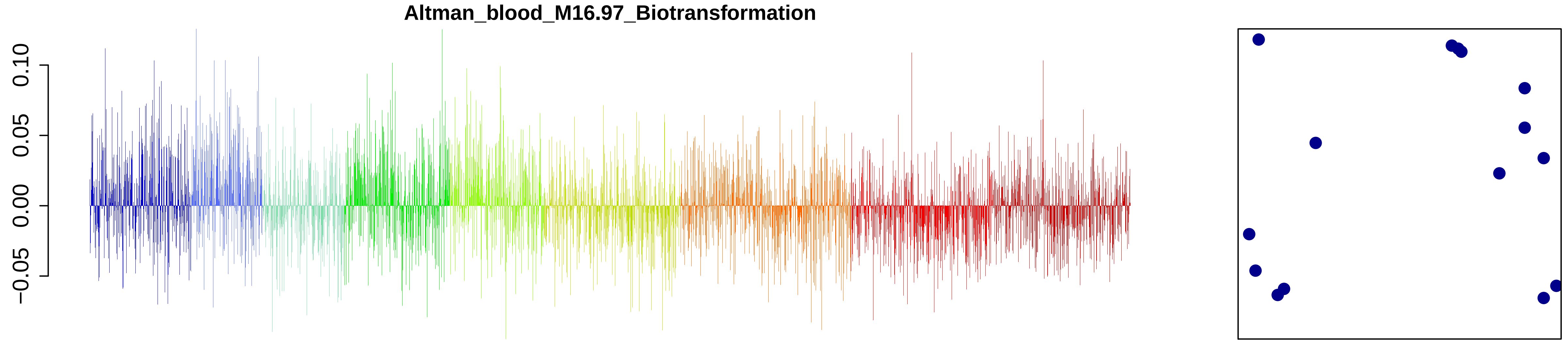
Altman_blood_M16.97_Biotransformation
0.10
0.05
0.00
−0.05

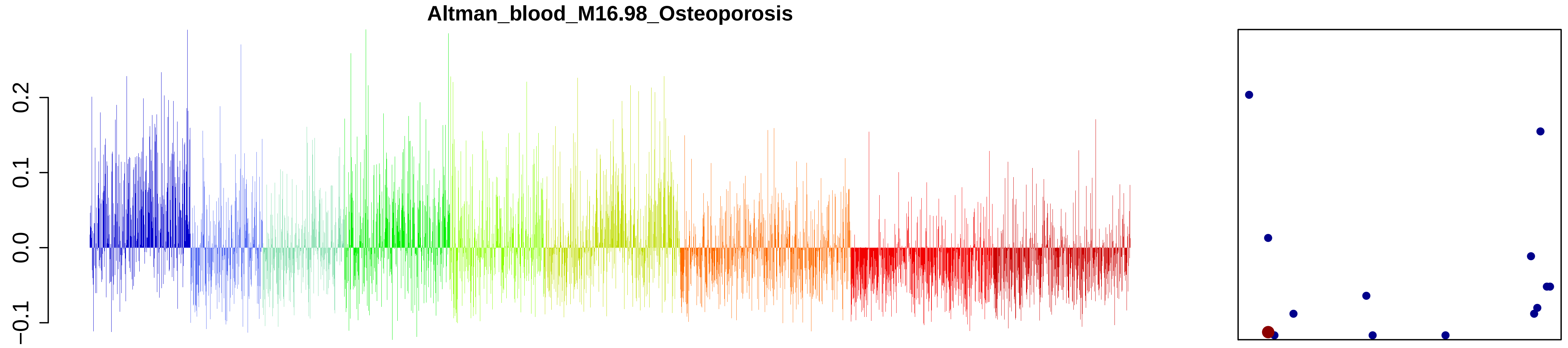
Altman_blood_M16.98_Osteoporosis
0.2
0.1
0.0
−0.1

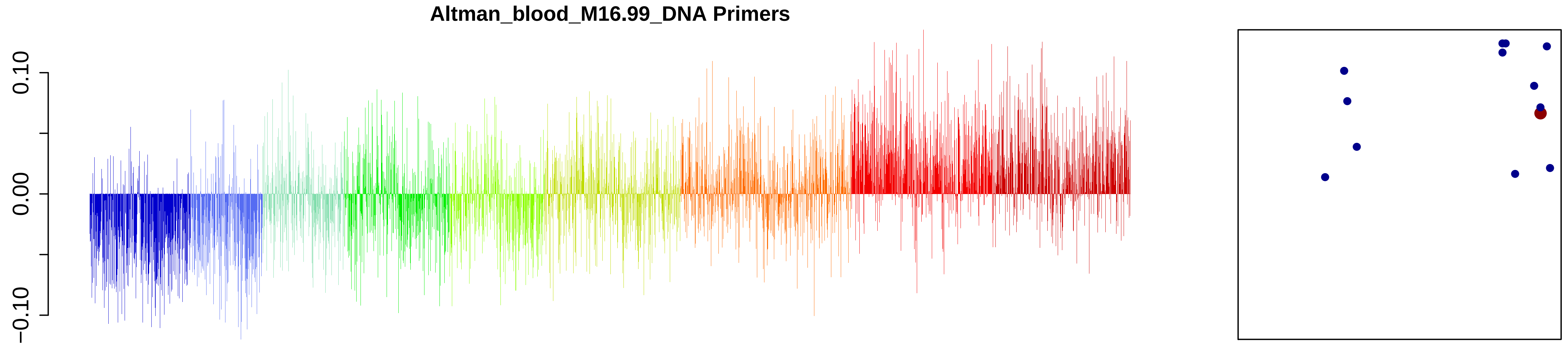
Altman_blood_M16.99_DNA Primers
0.10
0.00
−0.10

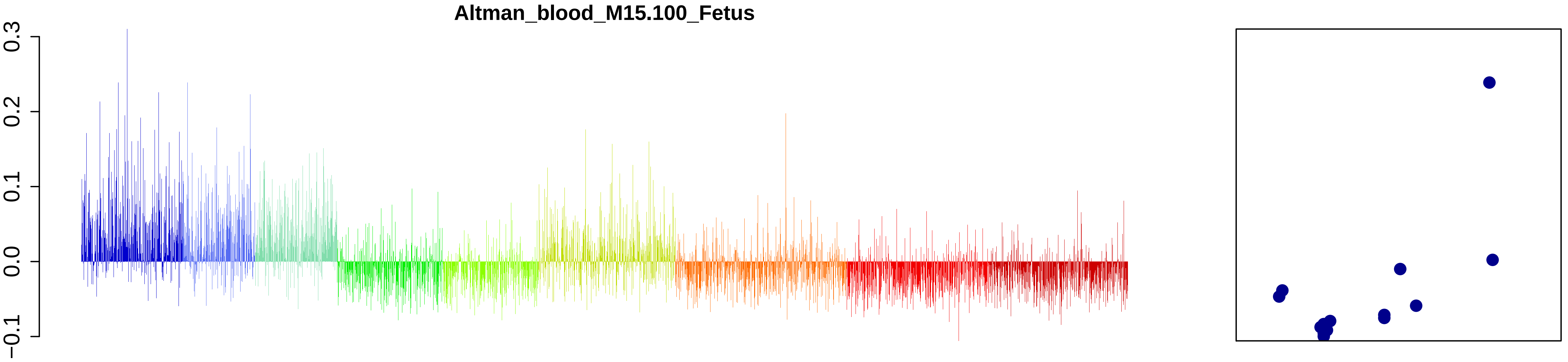
Altman_blood_M15.100_Fetus
0.3
0.2
0.1
0.0
−0.1

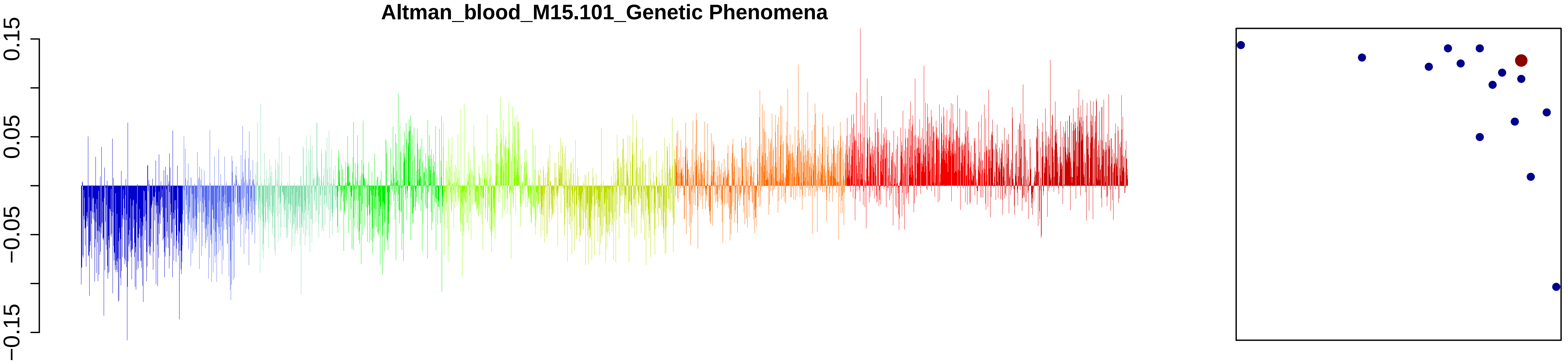
Altman_blood_M15.101_Genetic Phenomena
0.15
0.05
−0.05
−0.15

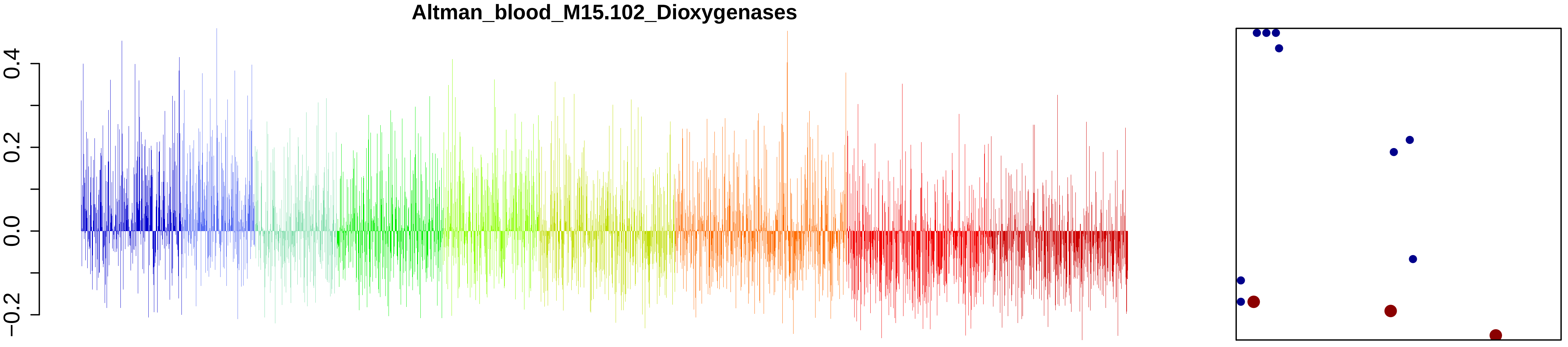
Altman_blood_M15.102_Dioxygenases
0.4
0.2
0.0
−0.2

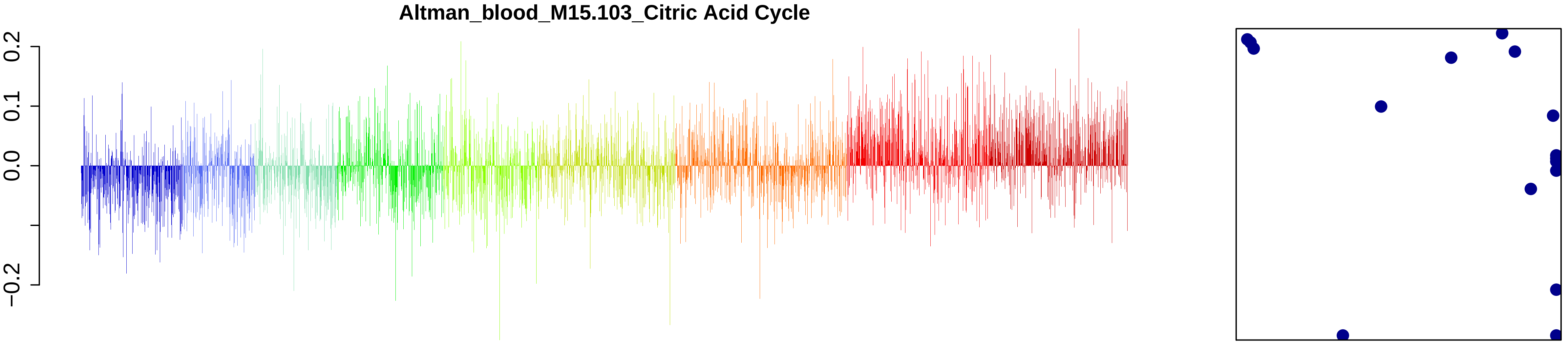
Altman_blood_M15.103_Citric Acid Cycle
0.2
0.1
0.0
−0.2

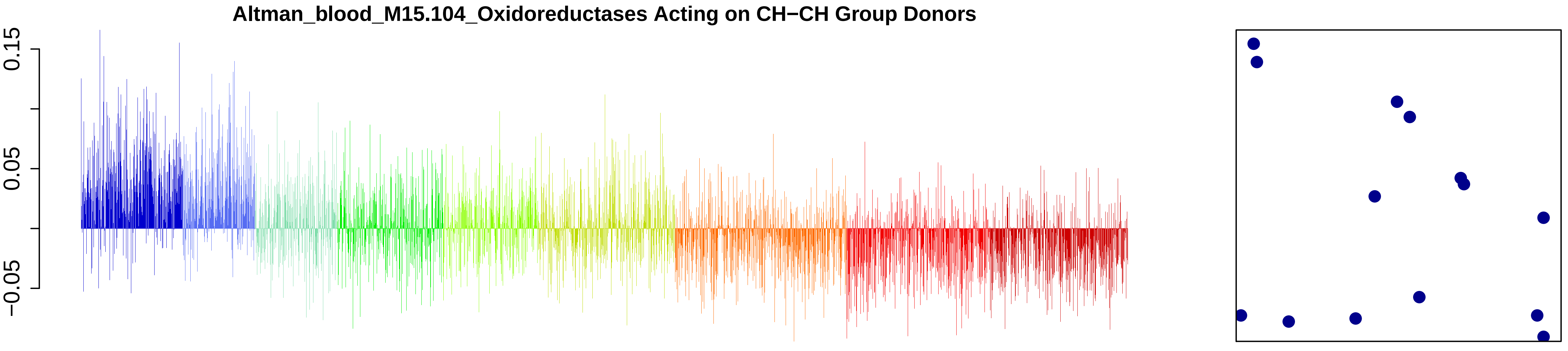
Altman_blood_M15.104_Oxidoreductases Acting on CH−CH Group Donors
0.15
0.05
−0.05

Altman_blood_M15.105_Metabolic Networks and Pathways

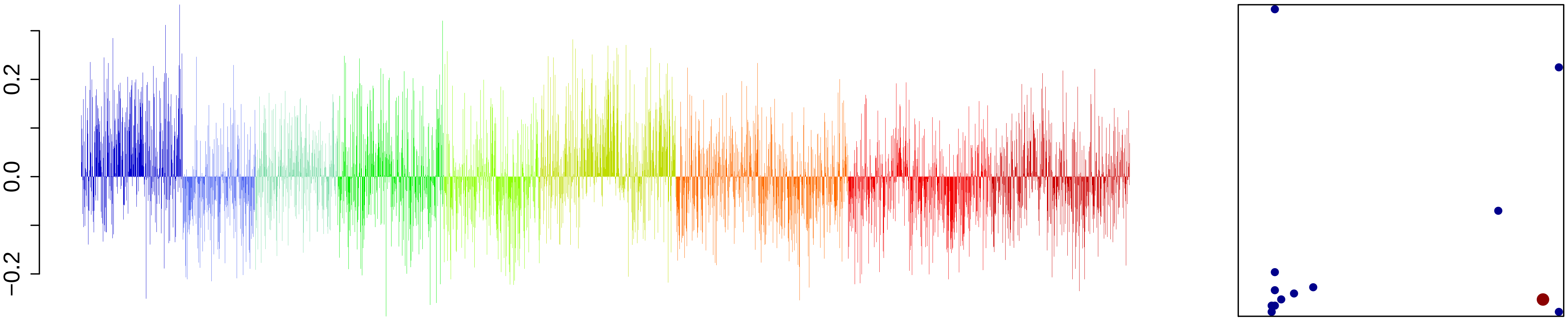

Altman_blood_M15.106_Buffers

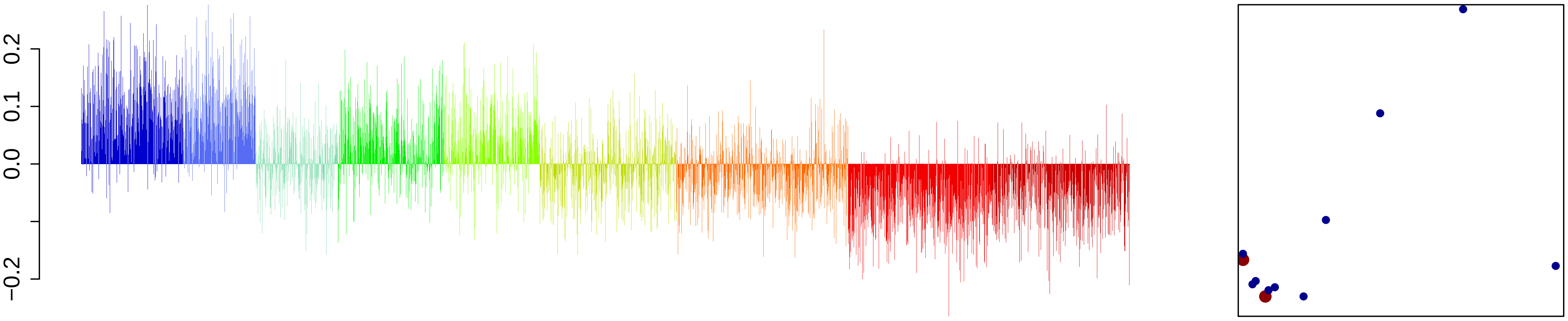

Altman_blood_M15.107_Biotransformation

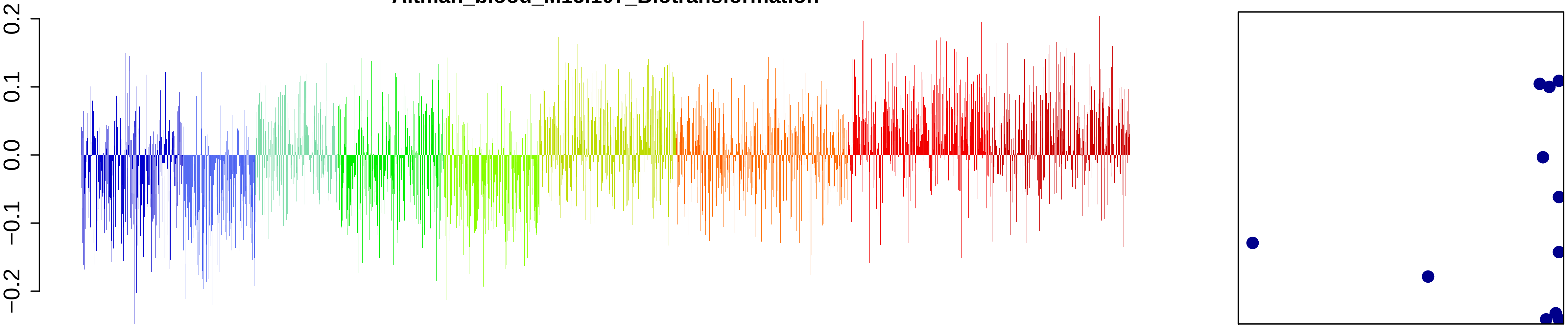

Altman_blood_M15.108_Cell Line, Transformed

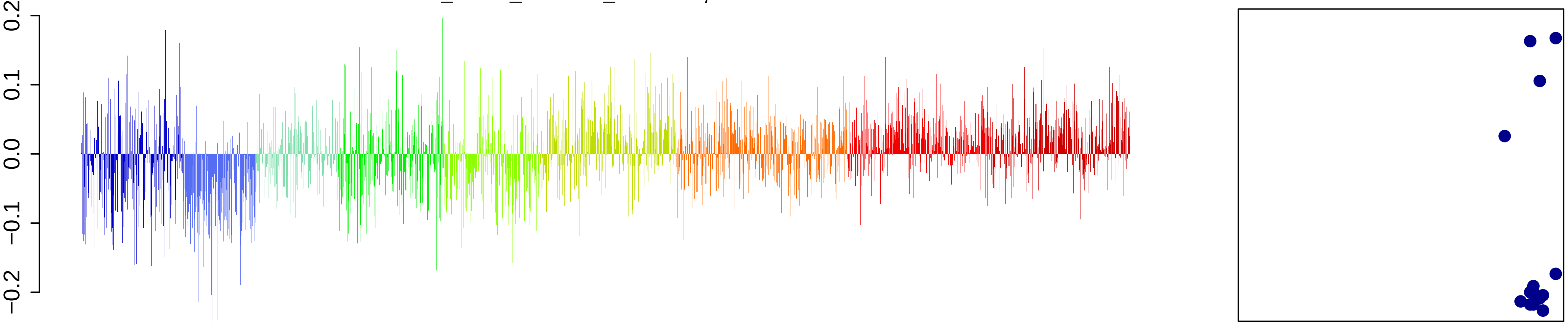

Altman_blood_M15.109_Etanercept

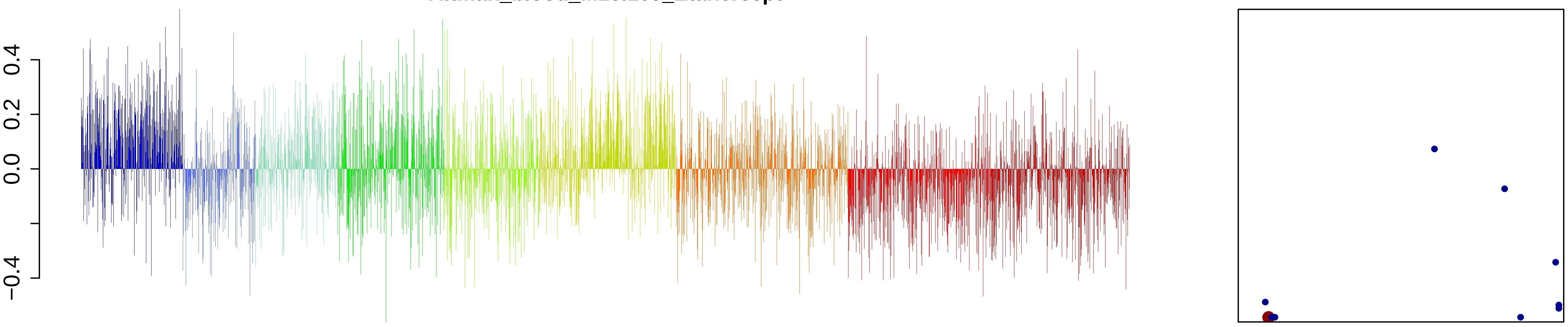

Altman_blood_M15.110_Polo−like Kinase

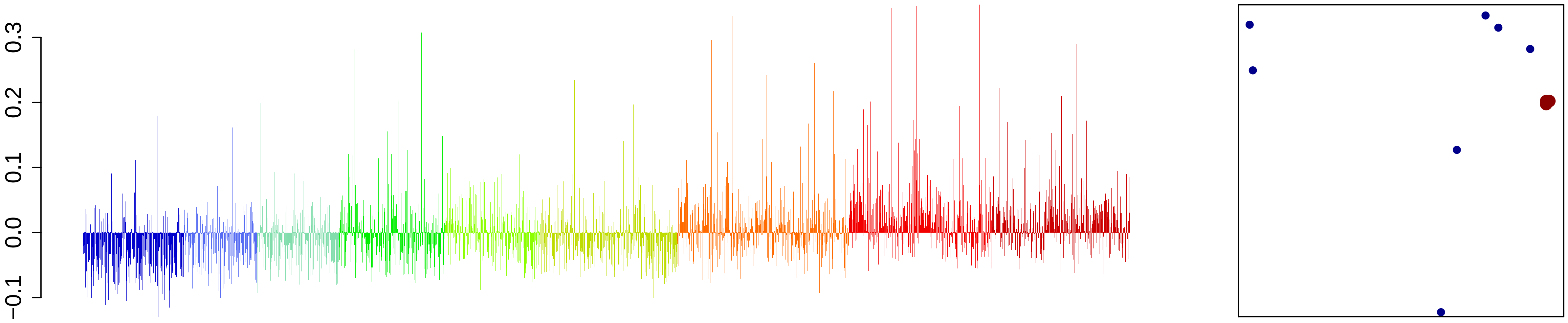

Altman_blood_M15.111_Docosahexaenoic Acids

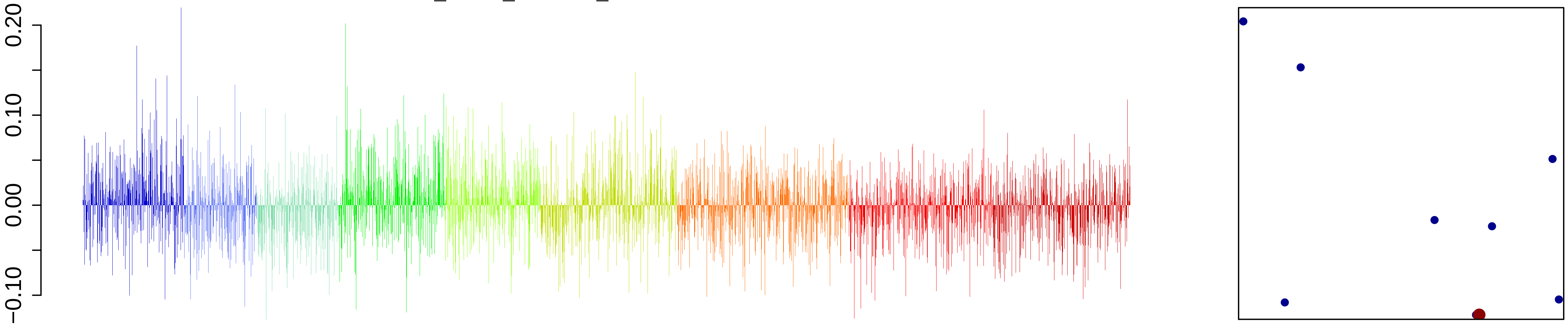

Altman_blood_M15.112_Karyopherins

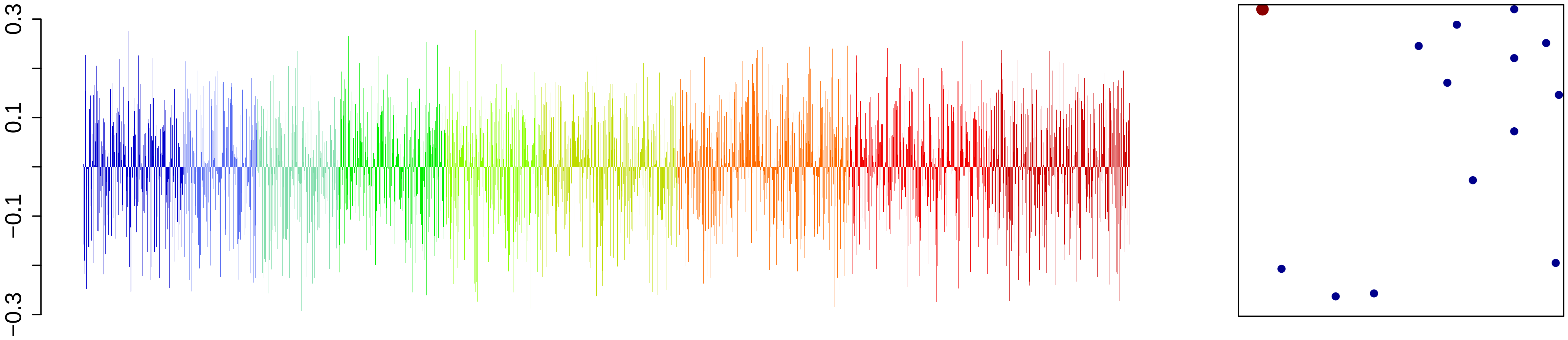

Altman_blood_M15.113_Antirheumatic Agents

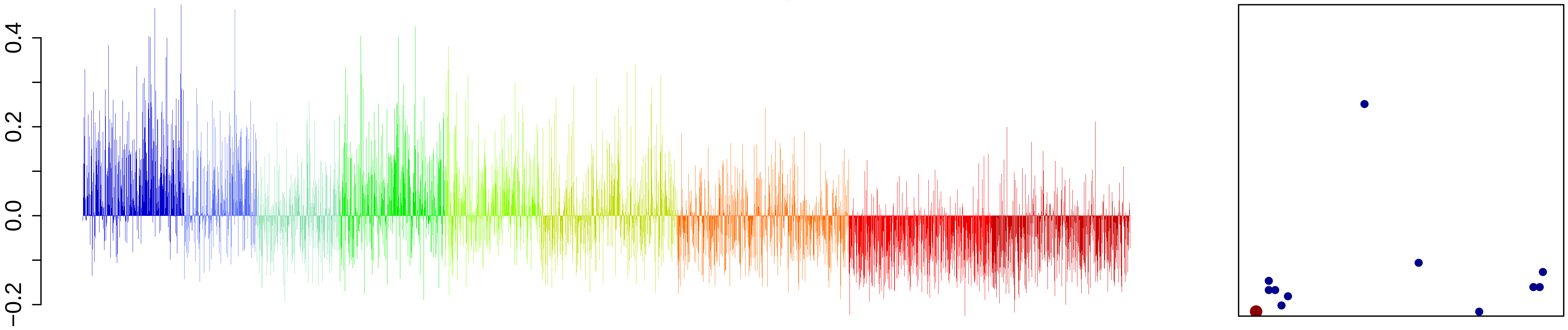

Altman_blood_M15.114_Indicators and Reagents

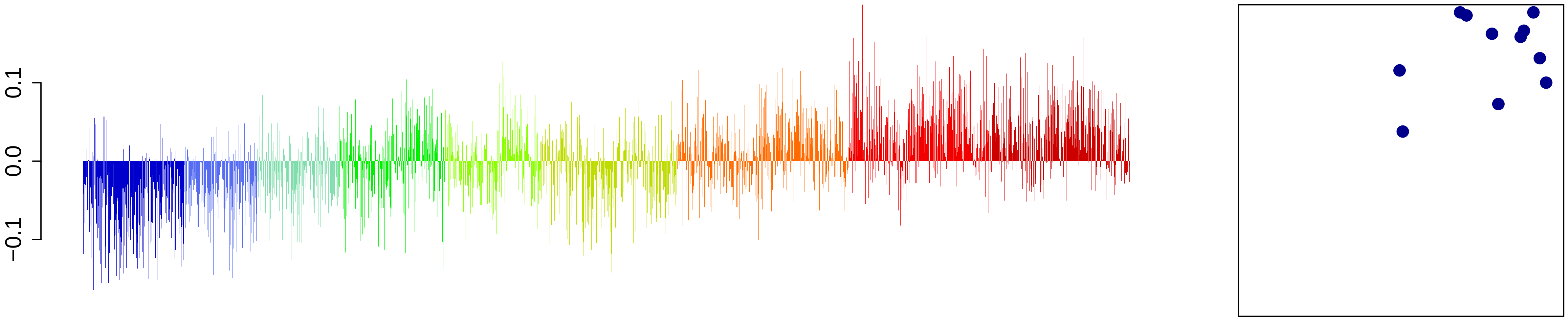

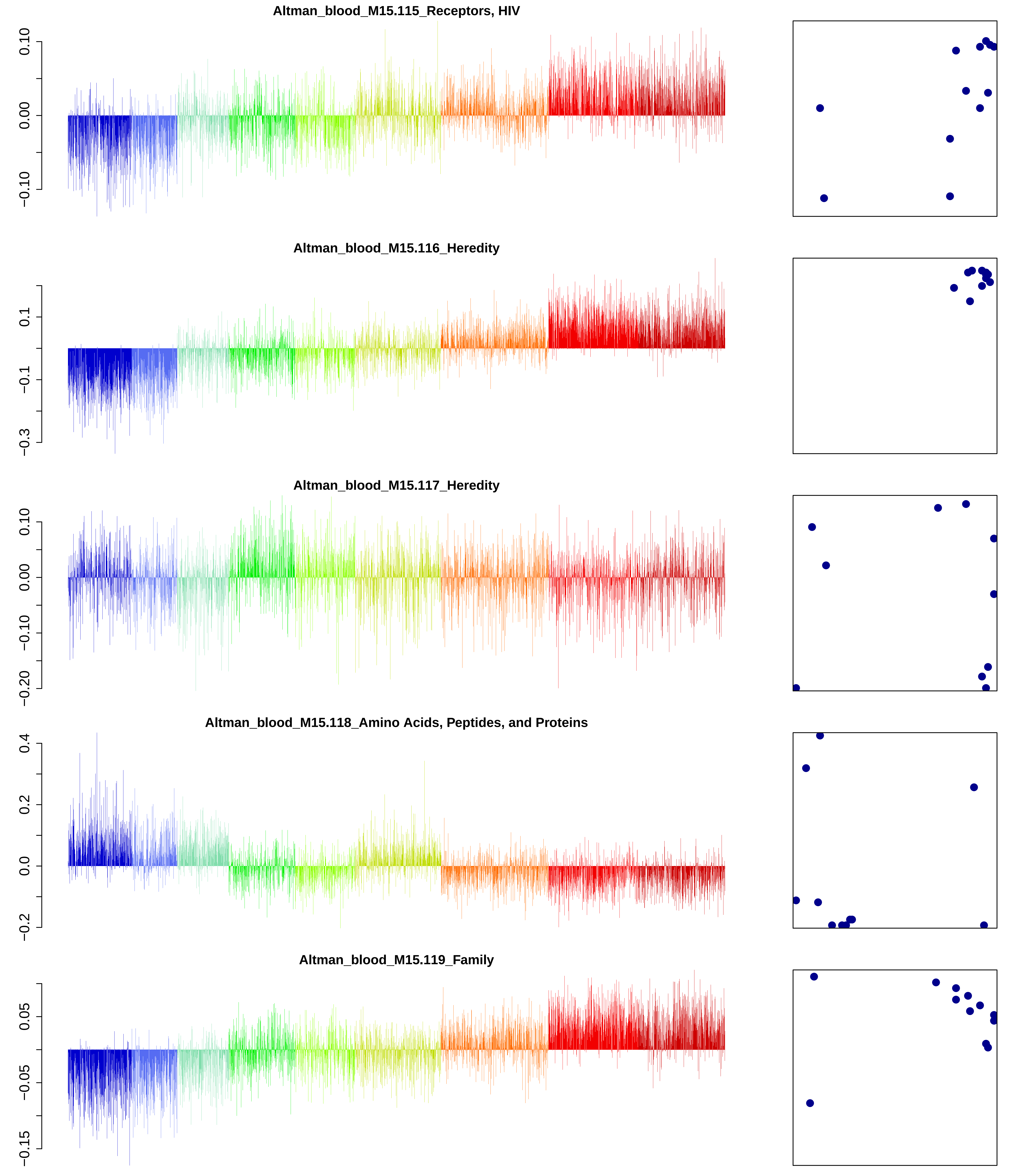

Altman_blood_M15.115_Receptors, HIV
0.10
0.00
−0.10
Altman_blood_M15.116_Heredity
0.1
−0.1
−0.3
Altman_blood_M15.117_Heredity
0.10
0.00
−0.10
−0.20
Altman_blood_M15.118_Amino Acids, Peptides, and Proteins
0.4
0.2
0.0
−0.2
Altman_blood_M15.119_Family
0.05
−0.05
−0.15

Altman_blood_M15.120_Amino Acids, Peptides, and Proteins

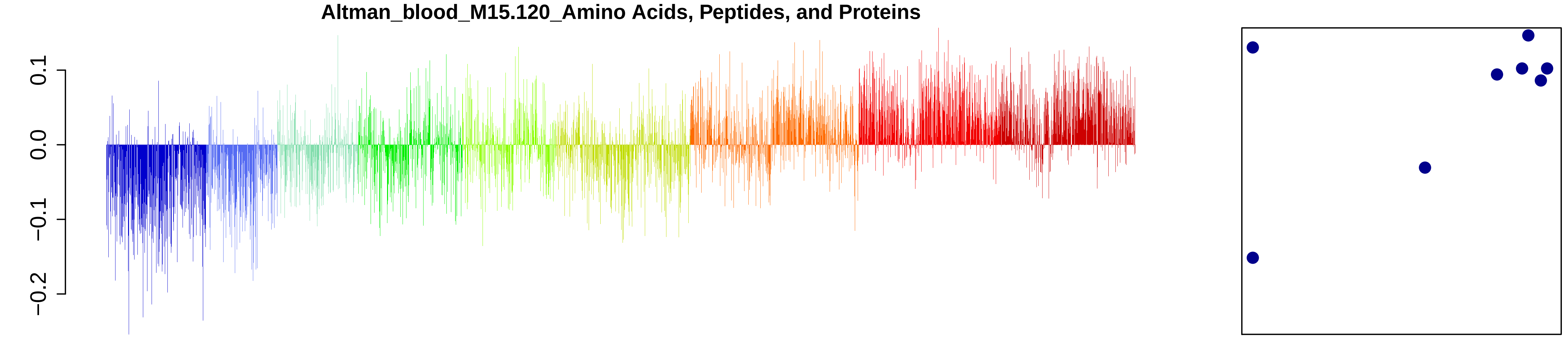

Altman_blood_M15.121_SN12C Cells

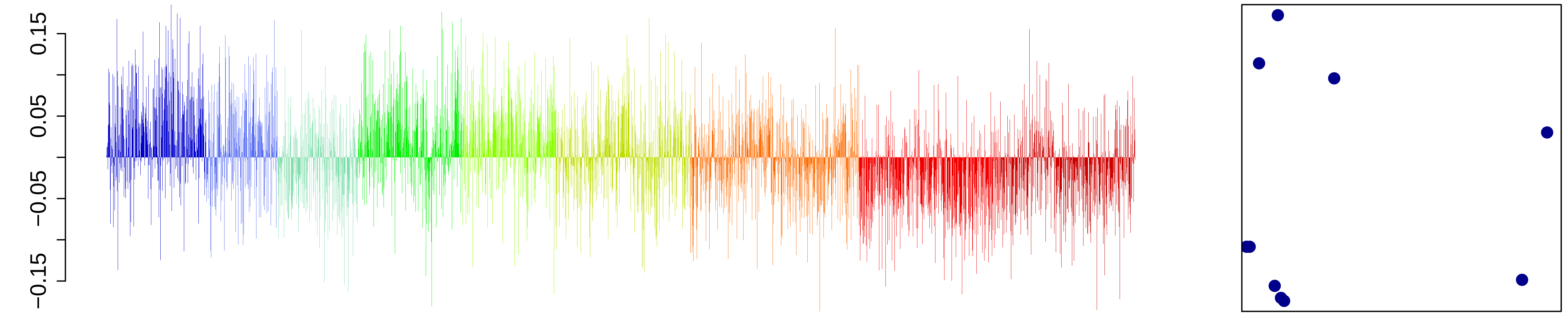

Altman_blood_M15.122_Proteins

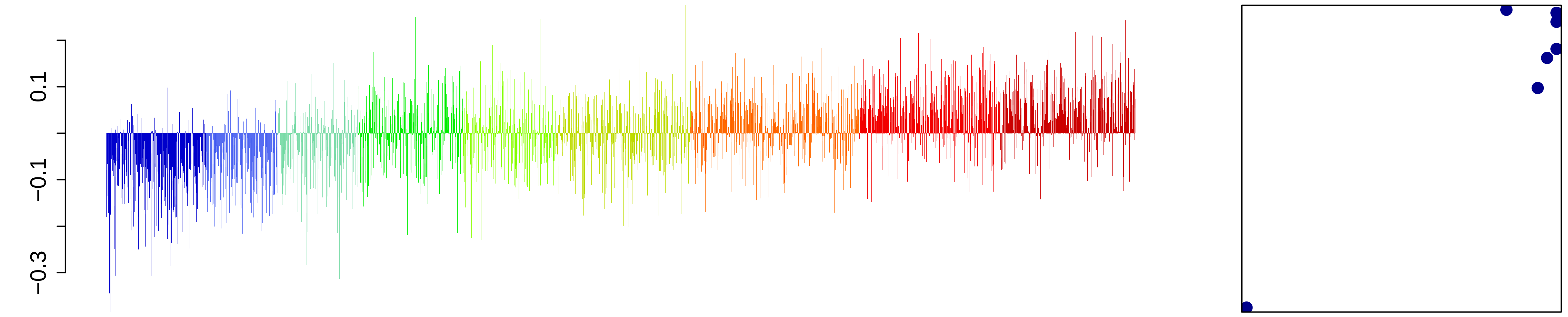

Altman_blood_M15.123_Cell Death

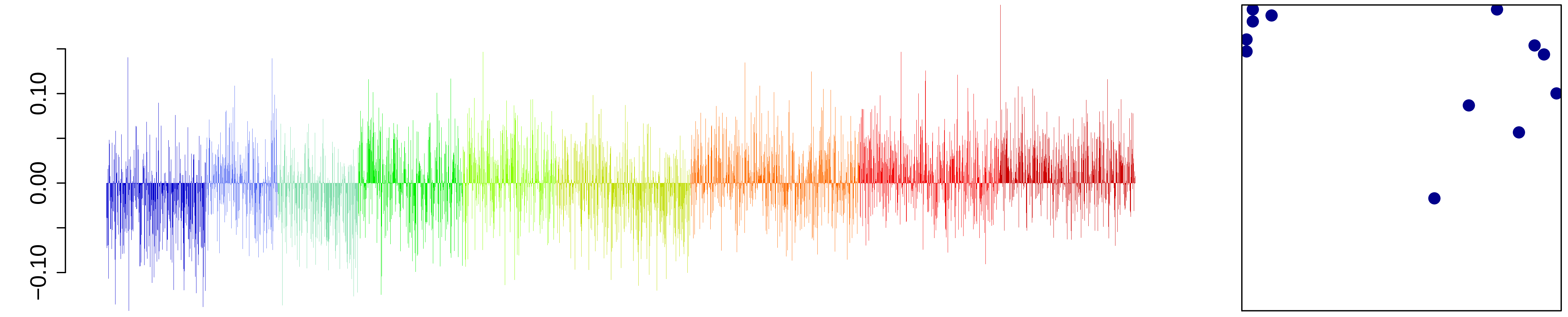

Altman_blood_M15.124_Piperidones

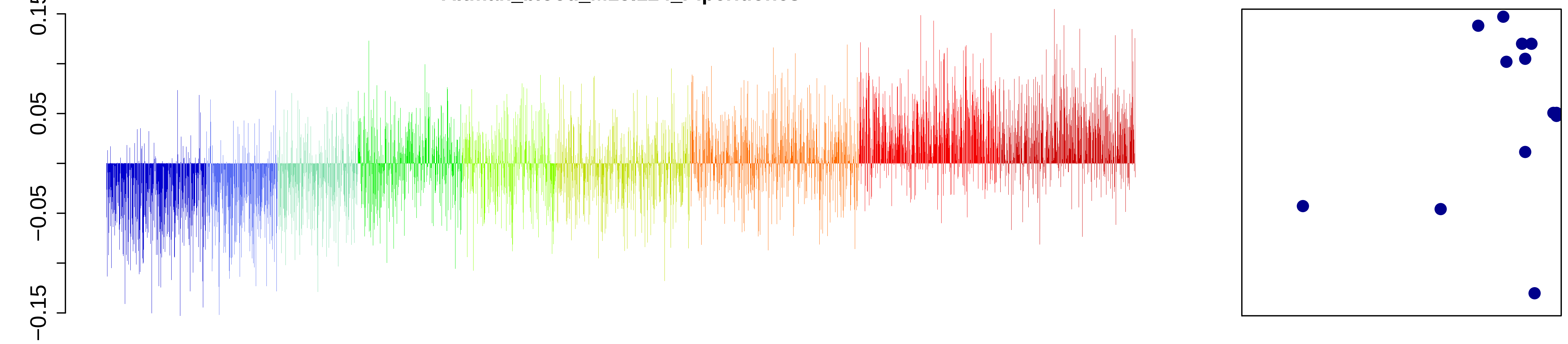

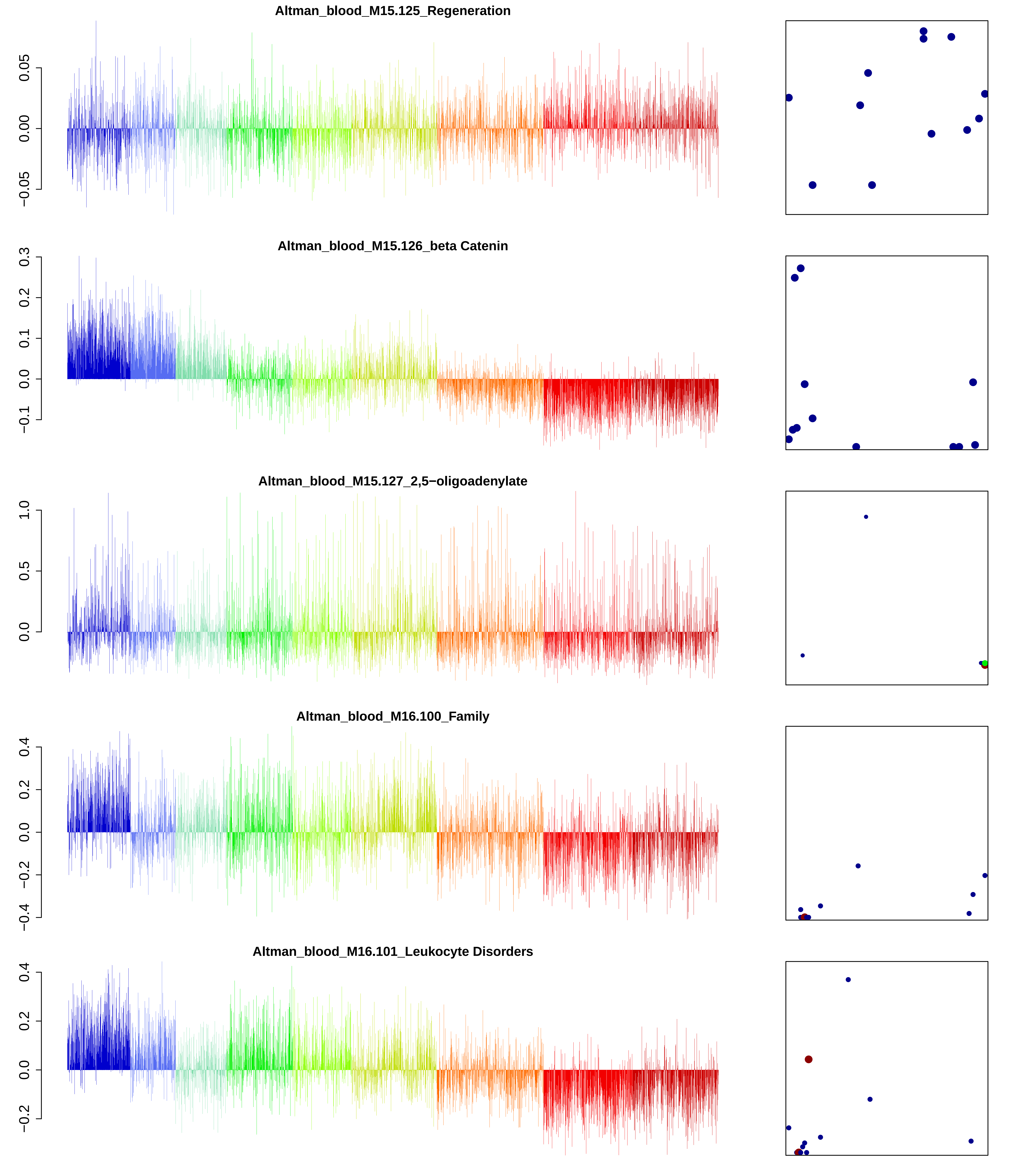
Altman_blood_M15.125_Regeneration
0.05
0.00
−0.05
Altman_blood_M15.126_beta Catenin
0.3
0.2
0.1
0.0
−0.1
Altman_blood_M15.127_2,5−oligoadenylate
1.0
0.5
0.0
Altman_blood_M16.100_Family
0.4
0.2
0.0
−0.2
−0.4
Altman_blood_M16.101_Leukocyte Disorders
0.4
0.2
0.0
−0.2

Altman_blood_M16.102_Imides

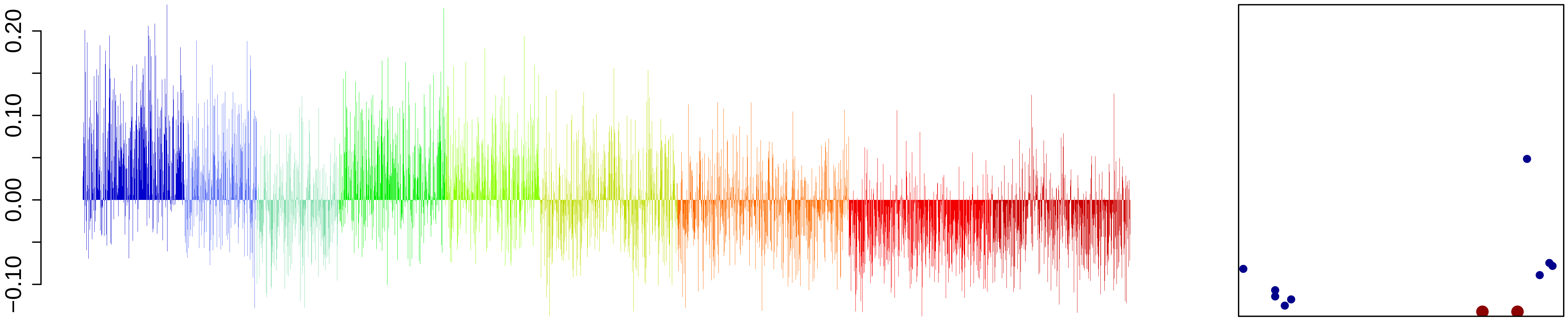

Altman_blood_M16.103_Biotransformation

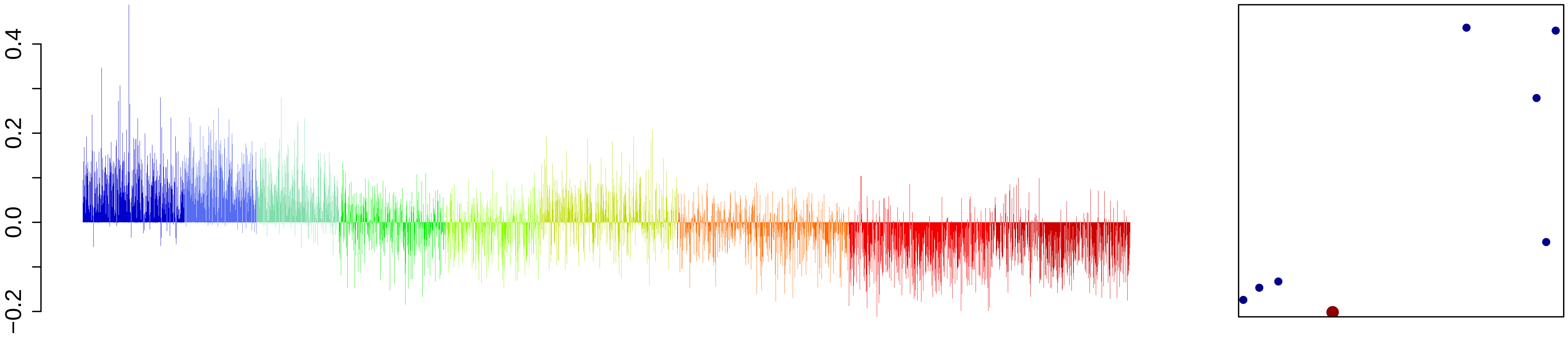

Altman_blood_M16.104_Cell Cycle

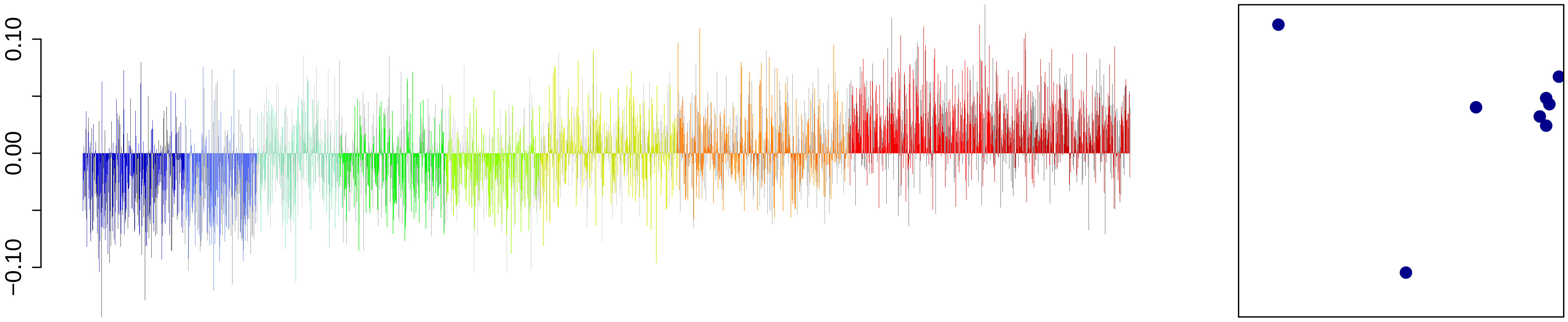

Altman_blood_M16.105_Tocopherols

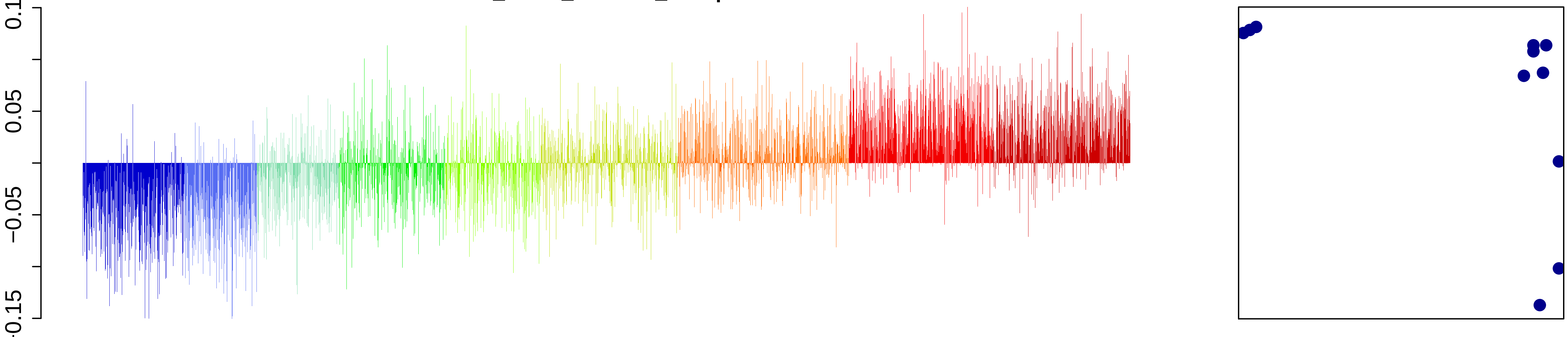

Altman_blood_M16.106_Base Sequence

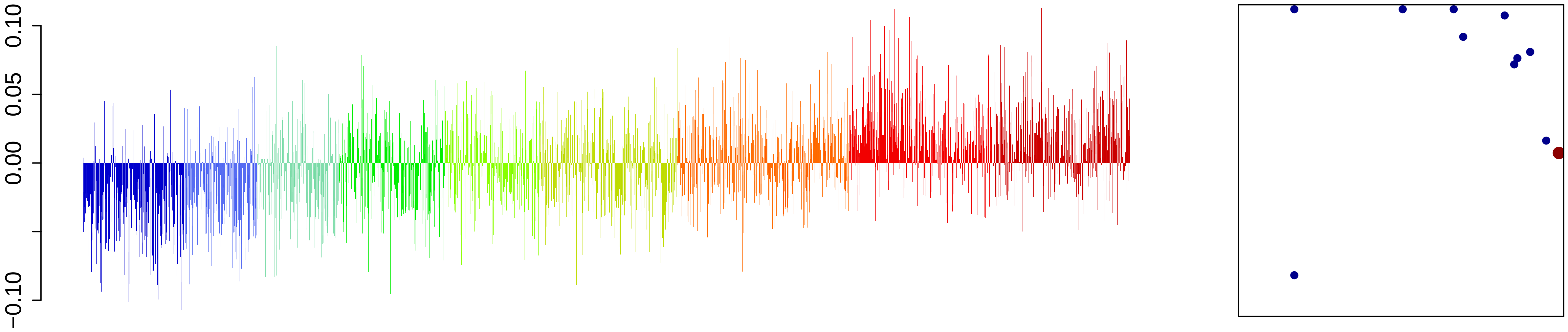

Altman_blood_M16.107_Cardiomyopathies

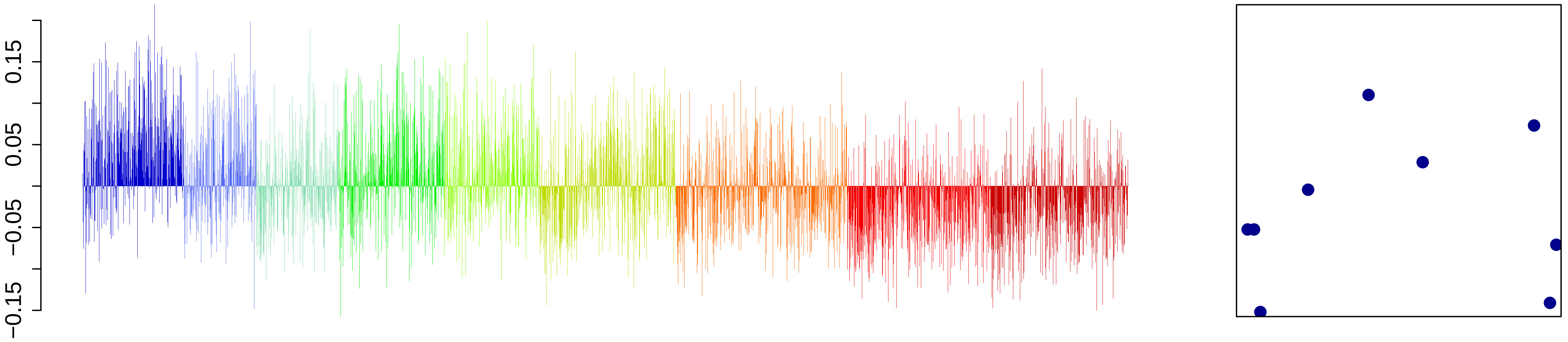

Altman_blood_M16.108_Epithelial Cells

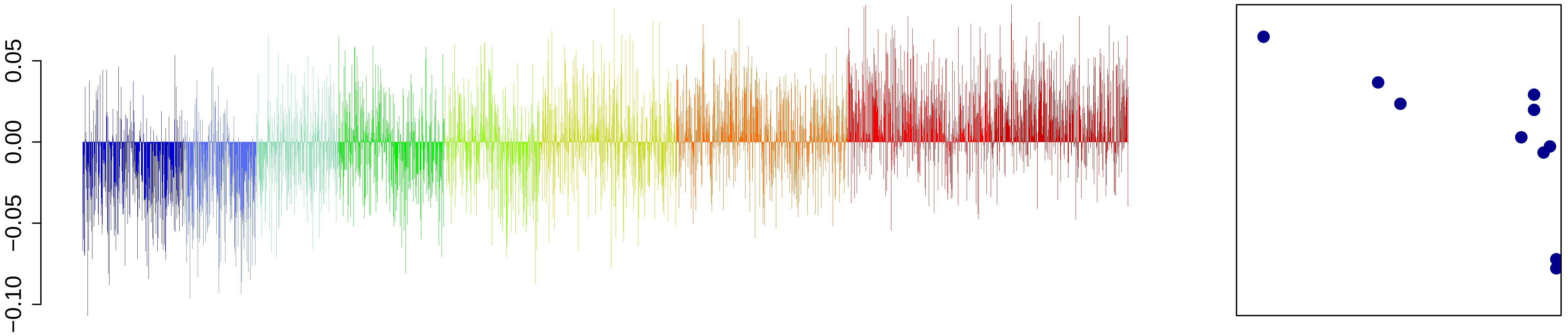

Altman_blood_M16.109_Cardiomyopathy, Dilated

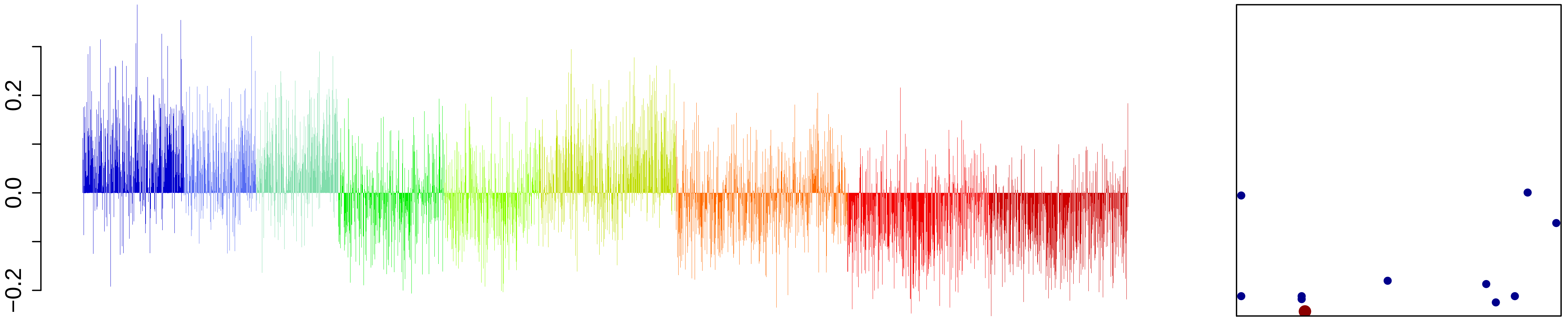

Altman_blood_M16.110_SN12C Cells

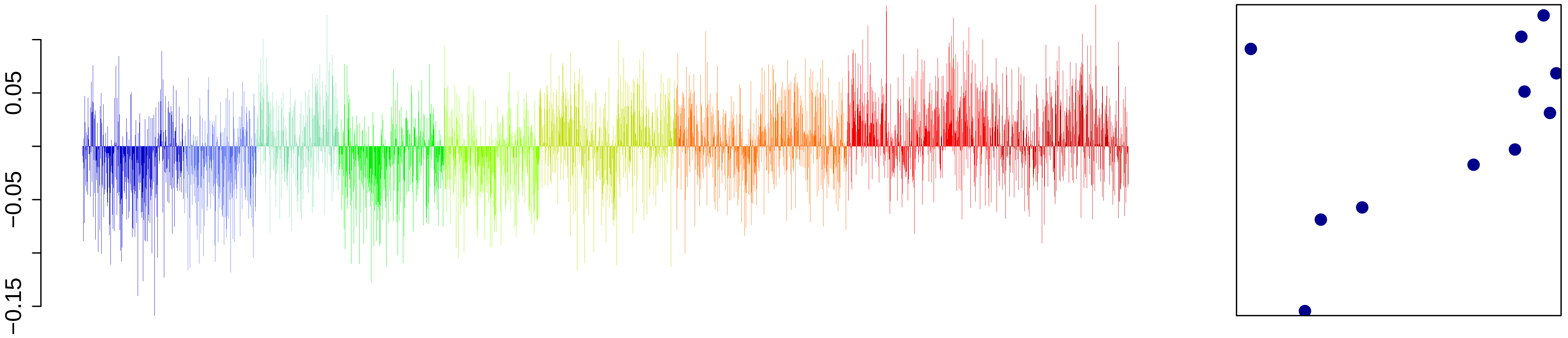

Altman_blood_M16.111_Dimerization

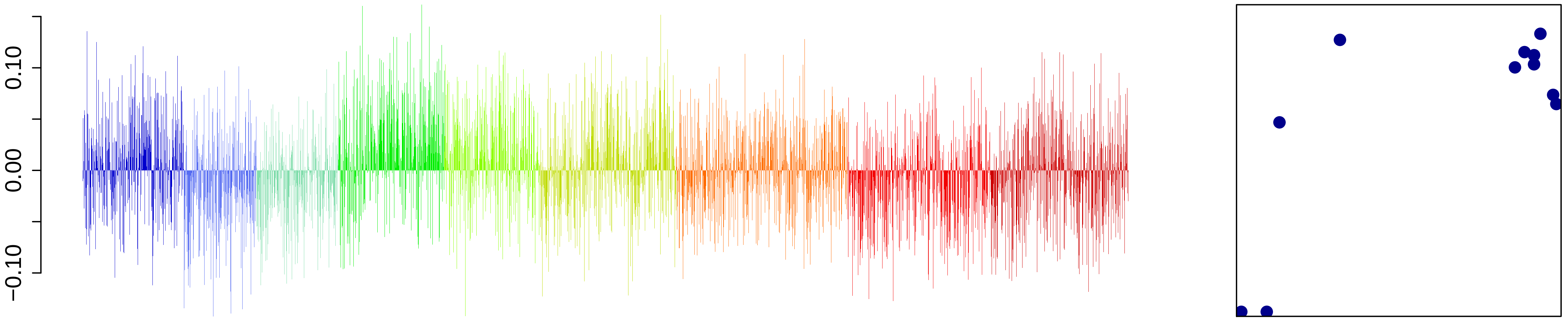

Supplement: Supplementary file 5 [file Data_Sheet_5.pdf]
